# Review of genus-group names in the family Tenebrionidae (Insecta, Coleoptera)

**DOI:** 10.3897/zookeys.1050.64217

**Published:** 2021-07-26

**Authors:** Patrice Bouchard, Yves Bousquet, Rolf L. Aalbu, Miguel A. Alonso-Zarazaga, Ottó Merkl, Anthony E. Davies

**Affiliations:** 1 Canadian National Collection of Insects, Arachnids and Nematodes, Agriculture and Agri-Food Canada, 960 Carling Avenue, Ottawa, Ontario, K1A 0C6, Canada Agriculture and Agri-Food Canada Ottawa Canada; 2 Gatineau, Quebec, Canada Unaffiliated Gatineau Canada; 3 California Academy of Sciences, Department of Entomology, 55 Music Concourse Drive, Golden Gate Park, San Francisco, California, 94118, USA California Academy of Sciences San Francisco United States of America; 4 Collection of Entomology, Museo Nacional de Ciencias Naturales (CSIC), José Gutiérrez Abascal, 2, E-28006, Madrid, Spain Museo Nacional de Ciencias Naturales Madrid Spain; 5 Hungarian Natural History Museum, Department of Zoology, H-1088 Baross u. 13, Budapest, Hungary Hungarian Natural History Museum Budapest Hungary

**Keywords:** Beetles, catalogue, classification, darkling beetles, distribution, nomenclature, publication dates

## Abstract

A review of genus-group names for darkling beetles in the family Tenebrionidae (Insecta: Coleoptera) is presented. A catalogue of 4122 nomenclaturally available genus-group names, representing 2307 valid genera (33 of which are extinct) and 761 valid subgenera, is given. For each name the author, date, page number, gender, type species, type fixation, current status, and first synonymy (when the name is a synonym) are provided. Genus-group names in this family are also recorded in a classification framework, along with data on the distribution of valid genera and subgenera within major biogeographical realms. A list of 535 unavailable genus-group names (e.g., incorrect subsequent spellings) is included. Notes on the date of publication of references cited herein are given, when known.

The following genera and subgenera are made available for the first time: *Anemiadena* Bouchard & Bousquet, **subgen. nov.** (in *Cheirodes* Gené, 1839), *Armigena* Bouchard & Bousquet, **subgen. nov.** (in *Nesogena* Mäklin, 1863), *Debeauxiella* Bouchard & Bousquet, **subgen. nov.** (in *Hyperops* Eschscholtz, 1831), *Hyperopsis* Bouchard & Bousquet, **subgen. nov.** (in *Hyperops* Eschscholtz, 1831), *Linio* Bouchard & Bousquet, **subgen. nov.** (in *Nilio* Latreille, 1802), *Matthewsotys* Bouchard & Bousquet, **gen. nov.**, *Neosolenopistoma* Bouchard & Bousquet, **subgen. nov.** (in *Eurynotus* W. Kirby, 1819), *Paragena* Bouchard & Bousquet, **subgen. nov.** (in *Nesogena* Mäklin, 1863), *Paulianaria* Bouchard & Bousquet, **gen. nov.**, *Phyllechus* Bouchard & Bousquet, **gen. nov.**, *Prorhytinota* Bouchard & Bousquet, **subgen. nov.** (in *Rhytinota* Eschscholtz, 1831), *Pseudorozonia* Bouchard & Bousquet, **subgen. nov.** (in *Rozonia* Fairmaire, 1888), *Pseudothinobatis* Bouchard & Bousquet, **gen. nov.**, *Rhytinopsis* Bouchard & Bousquet, **subgen. nov.** (in *Thalpophilodes* Strand, 1942), *Rhytistena* Bouchard & Bousquet, **subgen. nov.** (in *Rhytinota* Eschscholtz, 1831), *Spinosdara* Bouchard & Bousquet, **subgen. nov.** (in *Osdara* Walker, 1858), *Spongesmia* Bouchard & Bousquet, **subgen. nov.** (in *Adesmia* Fischer, 1822), and *Zambesmia* Bouchard & Bousquet, **subgen. nov.** (in *Adesmia* Fischer, 1822).

The names *Adeps* Gistel, 1857 and *Adepsion* Strand, 1917 **syn. nov.** [= *Tetraphyllus* Laporte & Brullé, 1831], *Asyrmatus* Canzoneri, 1959 **syn. nov.** [= *Pystelops* Gozis, 1910], *Euzadenos* Koch, 1956 **syn. nov.** [= *Selenepistoma* Dejean, 1834], *Gondwanodilamus* Kaszab, 1969 **syn. nov.** [= *Conibius* J.L. LeConte, 1851], *Gyrinodes* Fauvel, 1897 **syn. nov.** [= *Nesotes* Allard, 1876], *Helopondrus* Reitter, 1922 **syn. nov.** [= *Horistelops* Gozis, 1910], *Hybonotus* Dejean, 1834 **syn. nov.** [= *Damatris* Laporte, 1840], *Iphthimera* Reitter, 1916 **syn. nov.** [= *Metriopus* Solier, 1835], *Lagriomima* Pic, 1950 **syn. nov.** [= *Neogria* Borchmann, 1911], *Orphelops* Gozis, 1910 **syn. nov.** [= *Nalassus* Mulsant, 1854], *Phymatium* Billberg, 1820 **syn. nov.** [= *Cryptochile* Latreille, 1828], *Prosoblapsia* Skopin & Kaszab, 1978 **syn. nov.** [= *Genoblaps* Bauer, 1921], and *Pseudopimelia* Gebler, 1859 **syn. nov.** [= *Lasiostola* Dejean, 1834] are established as new synonyms (valid names in square brackets). *Anachayus* Bouchard & Bousquet, **nom. nov.** is proposed as a replacement name for *Chatanayus* Ardoin, 1957, *Genateropa* Bouchard & Bousquet, **nom. nov.** as a replacement name for *Apterogena* Ardoin, 1962, *Hemipristula* Bouchard & Bousquet, **nom. nov.** as a replacement name for *Hemipristis* Kolbe, 1903, *Kochotella* Bouchard & Bousquet, **nom. nov.** as a replacement name for *Millotella* Koch, 1962, *Medvedevoblaps* Bouchard & Bousquet, **nom. nov.** as a replacement name for *Protoblaps* G.S. Medvedev, 1998, and *Subpterocoma* Bouchard & Bousquet, **nom. nov.** is proposed as a replacement name for *Pseudopimelia* Motschulsky, 1860. *Neoeutrapela* Bousquet & Bouchard, 2013 is downgraded to a subgenus (**stat. nov.**) of *Impressosora* Pic, 1952. *Anchomma* J.L. LeConte, 1858 is placed in Stenosini: Dichillina (previously in Pimeliinae: Anepsiini); *Entypodera* Gerstaecker, 1871, *Impressosora* Pic, 1952 and *Xanthalia* Fairmaire, 1894 are placed in Lagriinae: Lagriini: Statirina (previously in Lagriinae: Lagriini: Lagriina); *Loxostethus* Triplehorn, 1962 is placed in Diaperinae: Diaperini: Diaperina (previously in Diaperinae: Diaperini: Adelinina); *Periphanodes* Gebien, 1943 is placed in Stenochiinae: Cnodalonini (previously in Tenebrioninae: Helopini); *Zadenos* Laporte, 1840 is downgraded to a subgenus (**stat. nov.**) of the older name *Selenepistoma* Dejean, 1834.

The type species [placed in square brackets] of the following available genus-group names are designated for the first time: *Allostrongylium* Kolbe, 1896 [*Allostrongyliumsilvestre* Kolbe, 1896], *Auristira* Borchmann, 1916 [*Auristiraoctocostata* Borchmann, 1916], *Blapidocampsia* Pic, 1919 [*Campsiapallidipes* Pic, 1918], *Cerostena* Solier, 1836 [*Cerostenadeplanata* Solier, 1836], *Coracostira* Fairmaire, 1899 [*Coracostiraarmipes* Fairmaire, 1899], *Dischidus* Kolbe, 1886 [*Helopssinuatus* Fabricius, 1801], *Eccoptostoma* Gebien, 1913 [*Taraxidesruficrus* Fairmaire, 1894], *Ellaemus* Pascoe, 1866 [*Emcephalussubmaculatus* Brême, 1842], *Epeurycaulus* Kolbe, 1902 [*Epeurycaulusaldabricus* Kolbe, 1902], *Euschatia* Solier, 1851 [*Euschatiaproxima* Solier, 1851], *Heliocaes* Bedel, 1906 [*Blaps emarginata* Fabricius, 1792], *Hemipristis* Kolbe, 1903 [*Hemipristisukamia* Kolbe, 1903], *Iphthimera* Reitter, 1916 [*Stenocararuficornis* Solier, 1835], *Isopedus* Stein, 1877 [*Helopstenebrioides* Germar, 1813], *Malacova* Fairmaire, 1898 [*Malacovabicolor* Fairmaire, 1898], *Modicodisema* Pic, 1917 [*Disemasubopaca* Pic, 1912], *Peltadesmia* Kuntzen, 1916 [*Metriopusplatynotus* Gerstaecker, 1854], *Phymatium* Billberg, 1820 [*Pimeliamaculata* Fabricius, 1781], *Podoces* Péringuey, 1886 [*Podocesgranosula* Péringuey, 1886], *Pseuduroplatopsis* Pic, 1913 [*Borchmanniajavana* Pic, 1913], *Pteraulus* Solier, 1848 [*Pteraulussulcatipennis* Solier, 1848], *Sciaca* Solier, 1835 [*Hylithusdisctinctus* Solier, 1835], *Sterces* Champion, 1891 [*Stercesviolaceipennis* Champion, 1891] and *Teremenes* Carter, 1914 [*Tenebriolongipennis* Hope, 1843].

Evidence suggests that some type species were misidentified. In these instances, information on the misidentification is provided and, in the following cases, the taxonomic species actually involved is fixed as the type species [placed in square brackets] following requirements in Article 70.3 of the International Code of Zoological Nomenclature: *Accanthopus* Dejean, 1821 [*Tenebriovelikensis* Piller & Mitterpacher, 1783], *Becvaramarygmus* Masumoto, 1999 [*Dietysusnodicornis* Gravely, 1915], *Heterophaga* Dejean, 1834 [*Opatrumlaevigatum* Fabricius, 1781], *Laena* Dejean, 1821, [*Scaurusviennensis* Sturm, 1807], *Margus* Dejean, 1834 [*Colydiumcastaneum* Herbst, 1797], *Pachycera* Eschscholtz, 1831 [*Tenebriobuprestoides* Fabricius, 1781], *Saragus* Erichson, 1842 [*Celibecostata* Solier, 1848], *Stene* Stephens, 1829 [*Colydiumcastaneum* Herbst, 1797], *Stenosis* Herbst, 1799 [*Tageniaintermedia* Solier, 1838] and *Tentyriopsis* Gebien, 1928 [*Tentyriopsispertyi* Gebien, 1940].

The following First Reviser actions are proposed to fix the precedence of names or nomenclatural acts (rejected name or act in square brackets): *Stenosisciliaris* Gebien, 1920 as the type species for *Afronosis* G.S. Medvedev, 1995 [*Stenosisleontjevi* G.S. Medvedev, 1995], *Alienoplonyx* Bremer, 2019 [*Alienolonyx*], *Amblypteraca* Mas-Peinado, Buckley, Ruiz & García-París, 2018 [*Amplypteraca*], *Caenocrypticoides* Kaszab, 1969 [*Caenocripticoides*], *Deriles* Motschulsky, 1872 [*Derilis*], *Eccoptostira* Borchmann, 1936 [*Ecoptostira*], †*Eodromus* Haupt, 1950 [†*Edromus*], *Eutelus* Solier, 1843 [*Lutelus*], *Euthriptera* Reitter, 1893 [*Enthriptera*], *Meglyphus* Motschulsky, 1872 [*Megliphus*], *Microtelopsis* Koch, 1940 [*Extetranosis* Koch, 1940, *Hypermicrotelopsis* Koch, 1940], *Neandrosus* Pic, 1921 [*Neoandrosus*], *Nodosogylium* Pic, 1951 [*Nodosogilium*], *Notiolesthus* Motschulsky, 1872 [*Notiolosthus*], *Pseudeucyrtus* Pic, 1916 [*Pseudocyrtus*], *Pseudotrichoplatyscelis* Kaszab, 1960 [*Pseudotrichoplatynoscelis* and *Pseudotrichoplatycelis*], *Rhydimorpha* Koch, 1943 [*Rhytimorpha*], *Rhophobas* Motschulsky, 1872 [*Rophobas*], *Rhyssochiton* Gray, 1831 [*Ryssocheton* and *Ryssochiton*], *Sphaerotidius* Kaszab, 1941 [*Spaerotidius*], *Stira* Agassiz, 1846 (Mollusca) [*Stira* Agassiz, 1846 (Coleoptera)], *Sulpiusoma* Ferrer, 2006 [*Sulpiosoma*] and *Taenobates* Motschulsky, 1872 [*Taeniobates*].

Supporting evidence is provided for the conservation of usage of *Cyphaleus* Westwood, 1841 nomen protectum over *Chrysobalus* Boisduval, 1835 nomen oblitum.

## Introduction

The last world catalogue of darkling beetles in the family Tenebrionidae Latreille, 1802 (Insecta: Coleoptera) was published by Gebien (1937a, 1938a, 1939, 1940, 1941, 1942a, 1943, 1948) more than 70 years ago. At the time, entire lineages were treated by coleopterists as separate families (e.g., Alleculidae Laporte, 1840, Lagriidae Latreille, 1825 (1820), Nilionidae Oken, 1843, Cossyphodidae Wasmann, 1899) and were therefore excluded from Gebien’s “Katalog der Tenebrioniden”. Since then, important studies on the relationships of taxa classified within Tenebrionidae and their close relatives, based on morphological (e.g., Doyen and Tschinkel 1982) and molecular data (e.g., Kergoat et al. 2014b; Kamiński et al. 2021b), have led to significant improvements in our understanding of the limits of the family and the relationships of major clades within the family. In addition, several taxa traditionally included in Tenebrionidae have now been transferred into closely related families (Table 1).

**Table 1.** Genus-group names transferred from Tenebrionidae to other families. An example of the current placement of each genus is given in the “Source” column.

Genus	Current placement	Source
*Acotulus* Reitter, 1891	Zopheridae	Schuh (2020: 68)
†*Adelidium* Tillyard, 1918	Coleoptera *incertae sedis*	Nabozhenko and Bukejs (2021: 55)
*Aegialites* Mannerheim, 1853	Salpingidae	Lawrence et al. (2010b: 722)
*Ageonoma* Pascoe, 1866	Zopheridae	Foley and Ivie (2008b: 37)
*Agnathus* Germar, 1818	Pyrochroidae	Young and Pollock (2010: 715)
*Anaplopus* Blackburn, 1890	Pythidae	Pollock (2010b: 709)
*Antarcticodomus* Brookes, 1951	Salpingidae	Lawrence et al. (2010b: 722)
*Apelta* Montrouzier 1864	Corylophidae	Fauvel (1903: 289)
*Aposyla* Pascoe, 1862	Boridae	Lawrence and Pollock (1994: 37)
*Archeocrypticus* Kaszab, 1964	Archeocrypticidae	Gimmel et al. (2018: 275)
*Arthopus* Sharp, 1876	Ulodidae	Leschen et al. (2016: 468)
*Bancous* Pic, 1946	Erotylidae	Skelley and Alonso-Zarazaga (2003: 107)
*Boros* Herbst, 1797	Boridae	Bouchard et al. (2011: 444)
*Brachyhelops* Fairmaire, 1885	Chrysomelidae	Reid (2014: 248)
*Brouniphylax* Strand, 1943	Ulodidae	Leschen et al. (2016: 468)
*Caanthus* Champion, 1894	Zopheridae	Ślipiński and Lawrence (1997: 372)
*Calophthalmus* J. Thomson, 1860	Mycteridae	Pollock (2010a: 693, as *Stilpnonotus*)
*Chalcodrya* Redtenbacher, 1867	Chalcodryidae	Lawrence and Leschen (2010: 567)
*Chanopterus* Boheman, 1858	Promecheilidae	Lawrence et al. (2010a: 563)
*Chitoniscus* C.O. Waterhouse, 1875	Promecheilidae	Kulzer (1963: 602)
†*Cistelites* Heer, 1864	Coleoptera *incertae sedis*	Nabozhenko (2019: 8)
*Cleteus* Fairmaire, 1906	Zopheridae	Freude (1974: 258)
*Coeloderes* Mulsant and Rey, 1859	Zopheridae	Bedel (1887: 199)
*Cotulades* Pascoe, 1860	Zopheridae	Ślipiński and Lawrence (2010: 549)
*Cycloderus* Solier, 1851	Pyrochroidae	Young and Pollock (2010: 715)
*Dacoderus* J.L. LeConte, 1859	Salpingidae	Bouchard et al. (2011: 446)
*Darwinella* Enderlein, 1912	Promecheilidae	Lawrence et al. (2010a: 563)
*Deridea* Westwood, 1875	Meloidae	Bouchard et al. (2011: 437)
*Diacalla* Pascoe, 1863	Anthicidae	Chandler (2010: 730)
*Diacallina* Champion, 1916	Anthicidae	Chandler (2010: 730)
*Dipsaconia* Pascoe, 1860	Ulodidae	Leschen et al. (2016: 468)
*Docalis* Pascoe, 1860	Zopheridae	Ślipiński and Lawrence (2010: 549)
*Egestriomima* Champion, 1916	Anthicidae	Chandler (2010: 730)
*Elascus* Pascoe, 1860	Zopheridae	Ślipiński and Lawrence (1999: 33)
*Endophloeus* Dejean, 1834	Zopheridae	Schuh (2020: 71)
*Enhypnon* Carter, 1919	Zopheridae	Turco et al. (2013: 371)
*Enneboeopsis* Champion, 1894	Archeocrypticidae	Gimmel et al. (2018: 275)
*Enneboeus* Waterhouse, 1878	Archeocrypticidae	Gimmel et al. (2018: 276)
*Eucistela* Carter, 1922	Pyrochroidae	Bousquet et al. (2015: 131)
*Eurypus* Kirby, 1819	Mycteridae	Pollock (2010a: 693)
*Exohadrus* Broun, 1893	Ulodidae	Leschen et al. (2016: 468)
*Falsoxanthalia* Pic,1934	Tetratomidae	Nikitsky (2020: 44)
*Ganyme* Pascoe, 1869	Ulodidae	Leschen et al. (2016: 468)
†*Helopides* Roemer, 1876	Cupedidae *incertae sedis*	Kirejtshuk et al. (2016: 146)
*Hymaea* Pascoe, 1869	Phloeostichidae	Bouchard et al. (2011: 362)
*Hydromedion* Waterhouse, 1875	Promecheilidae	Lawrence et al. (2010a: 563)
*Ictistygna* Pascoe, 1866	Anthicidae	Chandler (2010: 730)
*Ictistygnina* Champion, 1916	Anthicidae	Chandler (2010: 730)
*Ischyomius* Chevrolat, 1878	Pythidae	Pollock (2010b: 709)
*Latometus* Erichson, 1842	Zopheridae	Ślipiński and Lawrence (2010: 556)
*Loboglossa* Solier, 1851	Mycteridae	Pollock (2010a: 693)
*Malacodrya* Sharp, 1886	Promecheilidae	Lawrence et al. (2020: 28)
*Megazopherus* Casey, 1907	Zopheridae	Bousquet et al. (2018: 19)
*Melytra* Pascoe, 1869	Promecheilidae	Lawrence et al. (2010a: 563)
†*Menephiloides* Fujiyama, 1973	Coleoptera *incertae sedis*	Nabozhenko and Bukejs (2021: 55)
*Meralius* Casey, 1907	Zopheridae	Bousquet et al. (2018: 19)
*Meryx* Latreille, 1802	Ulodidae	Leschen et al. (2016: 468)
†*Mesothoris* Tillyard, 1916	Cupedidae *incertae sedis*	Kirejtshuk et al. (2016: 148)
*Micruloma* Carter, 1919	Cerylonidae	Doyen et al. (1990: 238)
*Mnionophilus* Carter, 1919	Zopheridae	Turco et al. (2013: 371)
*Mnionychus* Carter, 1926	Zopheridae	Lawrence and Ślipiński (2013: 12)
*Mylops* Fairmaire, 1884	Promecheilidae	Lawrence et al. (2010a: 563, as *Hydromedion*)
*Neboissianus* Kaszab, 1981	Archeocrypticidae	Gimmel et al. (2018: 276)
*Noserinus* Casey, 1907	Zopheridae	Foley and Ivie (2008: 18)
*Noserodes* Casey, 1907	Zopheridae	Bousquet et al. (2018: 19)
*Noserus* J.L. LeConte, 1862	Zopheridae	Bousquet et al. (2018: 19)
*Nosoderma* Solier, 1841	Zopheridae	Bousquet et al. (2018: 19)
*Notocerastes* Carter, 1926	Ulodidae	Leschen et al. (2016: 468)
*Notolea* Carter, 1915	Promecheilidae	Lawrence et al. (2010a: 566)
*Ocholissa* Pascoe, 1863	Salpingidae	Lawrence et al. (2010b: 724)
*Onysius* Broun, 1886	Promecheilidae	Lawrence et al. (2020: 33)
*Parahelops* Waterhouse, 1875	Promecheilidae	Lawrence et al. (2010a: 563)
*Paraphylax* Broun, 1880	Ulodidae	Leschen et al. (2016: 468)
*Parenneboeus* Kaszab, 1981	Archeocrypticidae	Gimmel et al. (2018: 276)
*Perimylops* Müller 1884	Promecheilidae	Lawrence et al. (2010a: 563)
*Phaennis* Champion, 1894	Ulodidae	Leschen et al. (2016: 469)
*Phaeogala* Fairmaire, 1896	Mycteridae	Pollock (2010a: 693)
*Phellopsis* J.L. LeConte, 1862	Zopheridae	Bousquet et al. (2018: 19)
*Philpottia* Broun, 1915	Chalcodryidae	Lawrence and Leschen (2010: 567)
*Phloeodes* J.L. LeConte, 1862	Zopheridae	Bousquet et al. (2018: 19)
*Phloeopsidius*Gebien, 1925	Zopheridae	Ivie et al. (2016: 780)
*Phycosecis* Pascoe, 1875	Phycosecidae	Bouchard et al. (2011: 349)
*Promecheilus* Solier, 1851	Promecheilidae	Lawrence et al. (2010a: 563)
*Pseudenneboeus* Kaszab, 1981	Archeocrypticidae	Gimmel et al. (2018: 277)
*Pseudonosoderma* Heyden, 1885	Zopheridae	Foley and Ivie (2008a: 8)
*Psilonycha* Fåhraeus, 1870	Scraptiidae	Bousquet et al. (2015: 131)
*Pteroderes* Germain, 1894	Ulodidae	Leschen et al. (2016: 469)
*Pycnidium* Erichson, 1847	Leiodidae	A. Newton (pers. comm.)
*Rhyssopera* Pascoe, 1860	Ulodidae	Leschen et al. (2016: 468)
*Rygmodus* White, 1846	Hydrophilidae	Bouchard et al. (2011: 158)
*Scoriaderma* Fairmaire, 1894	Zopheridae	Foley and Ivie (2008b: 21)
*Sesaspis* Casey, 1907	Zopheridae	Bousquet et al. (2018: 19)
*Sirrhas* Champion, 1893	Promecheilidae	Lawrence et al. (2010a: 563)
*Sivacrypticus* Kaszab, 1964	Archeocrypticidae	Gimmel et al. (2018: 277)
*Stilpnonotus* Gray, 1832	Mycteridae	Pollock (2010a: 693)
*Sympiezocera* P.H. Lucas, 1851	Cerambycidae	Danilevsky and Lin (2020: 211)
*Synercticus* Newman, 1842	Boridae	Pollock (2010: 699)
*Synopticus* J. Thomson, 1858	Tenebrionoidea *incertae sedis*	Bremer (2013a: 72)
*Syrphetodes* Broun, 1875	Ulodidae	Leschen et al. (2016: 469)
*Szekessya* Kaszab, 1955	Salpingidae	Lawrence et al. (2010b: 727)
*Tarphiomimus* Wollaston, 1873	Zopheridae	Ślipiński and Lawrence (2010: 553)
*Trachelolagria* Pic, 1941	Cleridae	Merkl (2004: 285)
*Trachyderas* Philippi and Philippi, 1864	Ulodidae	Leschen et al. (2016: 469)
*Trachyderastes* Kaszab, 1982	Ulodidae	Leschen et al. (2016: 469)
*Tretothorax* Lea, 1911	Salpingidae	Lawrence et al. (2010b: 722)
*Trictenotoma* Gray, 1831	Trictenotomidae	Bouchard et al. (2011: 444)
*Ulodes* Erichson, 1942	Ulodidae	Leschen et al. (2016: 469)
*Ulodica* Pascoe, 1769	Ulodidae	Leschen et al. (2016: 469)
†*Ulomites* Tillyard, 1916	Coleoptera *incertae sedis*	Martin (2010: 939)
*Uloporus* Casey, 1889	Archeocrypticidae	Gimmel et al. (2018: 276)
*Usechimorpha* Blaisdell, 1929	Zopheridae	Ślipiński and Lawrence (2010: 552)
*Usechus* Motschulsky, 1845	Zopheridae	Bousquet et al. (2018: 19)
*Verodes* Casey, 1907	Zopheridae	Bousquet et al. (2018: 19)
*Wattianus* Kaszab, 1981	Archeocrypticidae	Gimmel et al. (2018: 278)
*Zopherinus* Casey, 1907	Zopheridae	Bousquet et al. (2018: 19)
*Zopherodes* Casey, 1907	Zopheridae	Bousquet et al. (2018: 19)
*Zopherosis* White, 1859	Zopheridae	Ślipiński and Lawrence (2010: 549)
*Zopherus* Laporte, 1840	Zopheridae	Bousquet et al. (2018: 19)

With more than 30 000 described species worldwide (RLA, unpubl. data) and many new species described each year, the family Tenebrionidae has been described as “hyperdiverse” (Kergoat et al. 2014a). This overwhelming diversity, combined with significant changes in classification over the last decades, has hindered the production of recent catalogues at a world scale. Although some catalogues that cover large geographic areas have been published (e.g., Matthews and Bouchard 2008; Bousquet et al. 2018; Iwan et al. 2020), the nomenclatural information contained therein often differs in scope and major regional gaps of knowledge (e.g., fauna in the Afrotropical and Neotropical biogeographic realms) still remain.

The main objective of this publication is to provide a nomenclatural review of all genus-group names in the family Tenebrionidae. A table that includes an up-to-date synthesis of the classification of available genus-group names, along with the distribution of each valid genus and subgenus within the world’s major biogeographical realms, is included. Following bibliographic research, dating of references cited in this work is provided (whenever data is available) in order to establish the priority of genus-group names and therefore promote their nomenclatural stability in the future.

## Methods

### Nomenclatural data

All nomenclaturally available genus-group names in the family Tenebrionidae are listed alphabetically. For each name the author, year of publication, page number, gender (in square brackets: M = masculine, F = feminine, N = neuter), type species, typification, current status, and first synonymy (when the name is a synonym) are provided. The type species and type species fixation given for unjustified emendations and replacement names are identical to those of the available names they replace (International Commission on Zoological Nomenclature (henceforth ICZN) 1999, Article 67.8) and are listed following “Type species [automatic]:”. We follow previous authors (e.g., Alonso-Zarazaga and Lyal 2009; Bousquet et al. 2015, 2018) in considering type-species designations in R. Lucas (1920) potentially valid when a single species is listed under a particular genus-group name. According to ICZN (1999, Article 67.2.5) “a nominal species is deemed not to be originally included if it was doubtfully or conditionally included, or was cited as a species inquirenda, or as a species incertae sedis.” For this catalogue, any nominal species associated with a new genus with a question mark “?” was deemed not to be originally included. In accordance with Article 11.9.3.2 (ICZN 1999) we used the correct spelling of the species name (i.e., in agreement with the gender of the generic name) for all species-group name combinations. The author of each new synonym or new placement is given in square brackets (e.g., “[RLA]”) in the alphabetical list of available genus-group names. We included all genus-group names known to us up to the date of publication of this article.

While the identification of the type species is generally assumed to be correct (ICZN 1999, Article 70.1), evidence suggests that the type species of a small number of genus-group names were misidentified. In these cases, we include information about the misidentification and select the species (either the nominal species previously cited as the type species or the taxonomic species actually involved) that will best serve stability and universality in our opinion (ICZN 1999, Article 70.3). If we select the taxonomic species actually involved as type species, an action that could not have been taken under previous editions of the International Code of Zoological Nomenclature and that must be accompanied with a reference to Article 73 (ICZN 1999), it is given in the format “**fixed herein** (ICZN 1999, Article 70.3) as *Tenebriovelikensis* Piller and Mitterpacher, 1783, misidentified as *Tenebriocaraboides* Linnaeus, 1758 in the original designation by monotypy in Dejean (1821).”

In some cases, we have noted that the original combination of the accepted name of the type species is a primary homonym of an older species name (e.g., *Helopstenebrioides* Germar, 1813, the type species of *Isopedus* Stein, 1877, is a junior primary homonym of *Helopstenebrioides* Palisot de Beauvois, 1812). We refrain proposing replacement names for two raisons: first, the status of some senior homonyms, which in most cases involved pre-1820 names, is uncertain; second, in some cases the two homonyms belong to taxa in use but the names apply to taxa not considered congeneric after 1899, and the Commission (ICZN 1999: Article 23.9.5) mandates that the author(s) must not automatically replace the junior homonym but instead should refer to the Commission for a ruling. We hope that experts on the taxonomy of the taxa involved will look at all evidence and propose solutions to remove the homonymies as needed.

Genus-group names encountered in the literature that are not nomenclaturally available are listed in Appendix 1. Reasons for assigning the status of “nomenclaturally unavailable” for these names include: name proposed as an unmodified vernacular word, not appropriately latinized (ICZN 1999, Article 11.3, Recommendation 11A); name not used as valid when proposed (ICZN 1999, Article 11.5); name first published as a synonym and not treated before 1961 as an available name and adopted as the name of a taxon or treated as a senior homonym (ICZN 1999, Article 11.6); name published before 1931 without a description, a definition or an indication (Article 12.1); name proposed as a replacement name for an unavailable name (ICZN 1999, Article 12.2.3); name published after 1930 without a description, a definition or a bibliographic reference to such a published statement (ICZN 1999, Article 13.1); name published after 1930 without a type species (ICZN 1999, Article 13.3); replacement name published after 1930, for a name without valid typification, without designating a type species (ICZN 1999, Article 13.3.1); new nominal genus or subgenus published in a combined description with a new species not marked by “gen. nov., spec. nov.” or an equivalent expression (ICZN 1999, Article 13.4); name published after 1999 and not explicitly indicated as intentionally new (ICZN 1999, Article 16.1); rejected alternative original spelling (ICZN 1999, Article 24.2.3); original spelling corrected in the same work (ICZN 1999, Article 32.5.1.1); incorrect subsequent spelling that is not in prevailing usage (ICZN 1999, Article 33.3).

The symbol for fossil (†) and the acronym for plate (pl.) were used when relevant. The following initials were necessary to distinguish different authors with an identical family name: F.M. Brown, K.W. Brown, W. Kirby, W.F. Kirby, J.E. LeConte, J.L. LeConte, P.H. Lucas, R. Lucas, W.J. MacLeay, W.S. MacLeay, G.S. Medvedev, L.N. Medvedev, G.-A. Olivier, E. Olivier, F. Soldati, L. Soldati, C.G. Thomson, J. Thomson, C.O. Waterhouse, F.H. Waterhouse, G.R. Waterhouse. Note that the honorary title “Tjan-Schansky” was added by the Emperor of All Russia Nikolay II to the surname of A.P. Semenov’s father (and all his family members) in 1906 (M. Nabozhenko, pers. comm.). We use the author name “Semenov” for works published up to the end of 1906 and “Semenov-Tjan-Shansky” for those published after 1906. We also follow Bessudnova (2012: 102) and use the author’s surname “Fischer” for G. Fischer’s works up to the end of 1832 and use “Fischer von Waldheim” from 1833, the year he became a member of the Russian nobility.

Original literature for species-group names (i.e., type species) was verified; however, those works are not included in the “References” section because they are not the principal focus of this article, to conserve space and because they are, for the most part, available in recent publications. When the author(s) of a scientific name is different from the author(s) of the publication in which the name was proposed, the authorship of the scientific name is given in the format “Gray in Griffith and Pidgeon, 1832” (ICZN, Article 50.1.1 and Recommendation 51E) and only the reference pertaining to the whole work is given in the “References” section. Titles of references using a non-Latin alphabet were translated in English.

The name *Xylotinus*, as used by Sturm (1826: 59) to establish *Xylotinusflabellicornis* Sturm, 1826, the type species of *Rhipidandrus* J.L. LeConte, 1862, is considered an incorrect subsequent spelling for *Xyletinus* Latreille, 1810 [Coleoptera: Anobiidae] and is therefore not included here as an available genus-group name in Tenebrionidae (see Schwarz and Barber 1914: 175).

Two new synonymies were proposed in the fascicle on Coleoptera of the ‘Nomenclator Zoologicus’ by Agassiz (1846a). These synonymies are credited here to “Erichson in Agassiz” since Agassiz, who was not a beetle expert, acknowledged the contribution of the Coleoptera specialist W.F. Erichson, on the fascicle's title page, in the production of his catalogue.

### Classification

The classification presented in Table 2 is a synthesis starting from works on Tenebrionidae family-group names (e.g., Bouchard et al. 2005, 2011; Bouchard and Bousquet 2020a) with additional data published in recent regional catalogues (e.g., Matthews and Bouchard 2008; Bousquet et al. 2018; Iwan et al. 2020), on fossils (Nabozhenko 2019), as well as on the diversity, taxonomy and phylogenetics of various clades (e.g., Bremer and Lillig 2014; Aalbu et al. 2017, 2018; Kamiński et al. 2019a, b, c, 2021b; Matthews and Lawrence 2019; Lumen et al. 2020; Lillig and Bremer 2021; Gearner et al. 2021). Only nomenclaturally available names are included in Table 2 wherein the valid genus-group names are listed in alphabetical order under each valid family-group name and the synonyms (preceded by “=”) are given in chronological order. Valid subgenera are given following the acronym “SG”.

### Distribution

**Figure 1.**
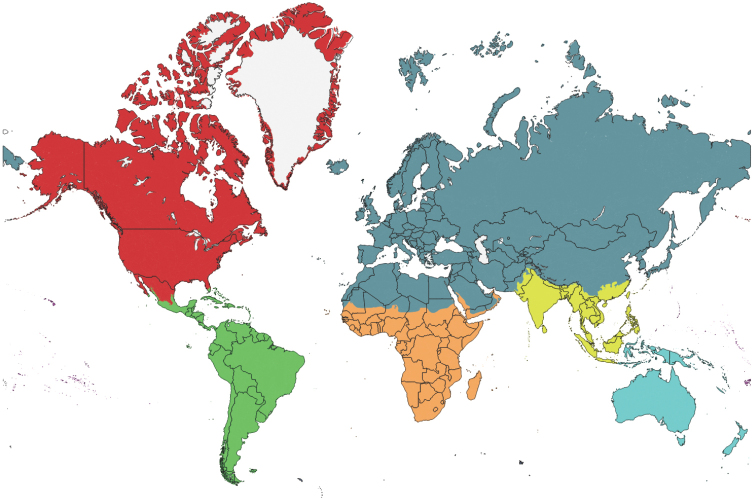
Map showing the biogeographic realms used to record the distribution of each valid genus and subgenus of Tenebrionidae (see Table 2). Blue (dark): Palaearctic; blue (pale): Australasia; green: Neotropic; orange: Afrotropic; red: Nearctic; yellow: Indo-Malay (see Olson et al. (2001: fig. 1) for the boundaries of the Oceania biogeographic realm). Figures 2–5 show closeup maps of boundary areas. The Antarctic realm is excluded since none of the valid Tenebrionidae genera and subgenera are distributed therein.

**Figure 2.**
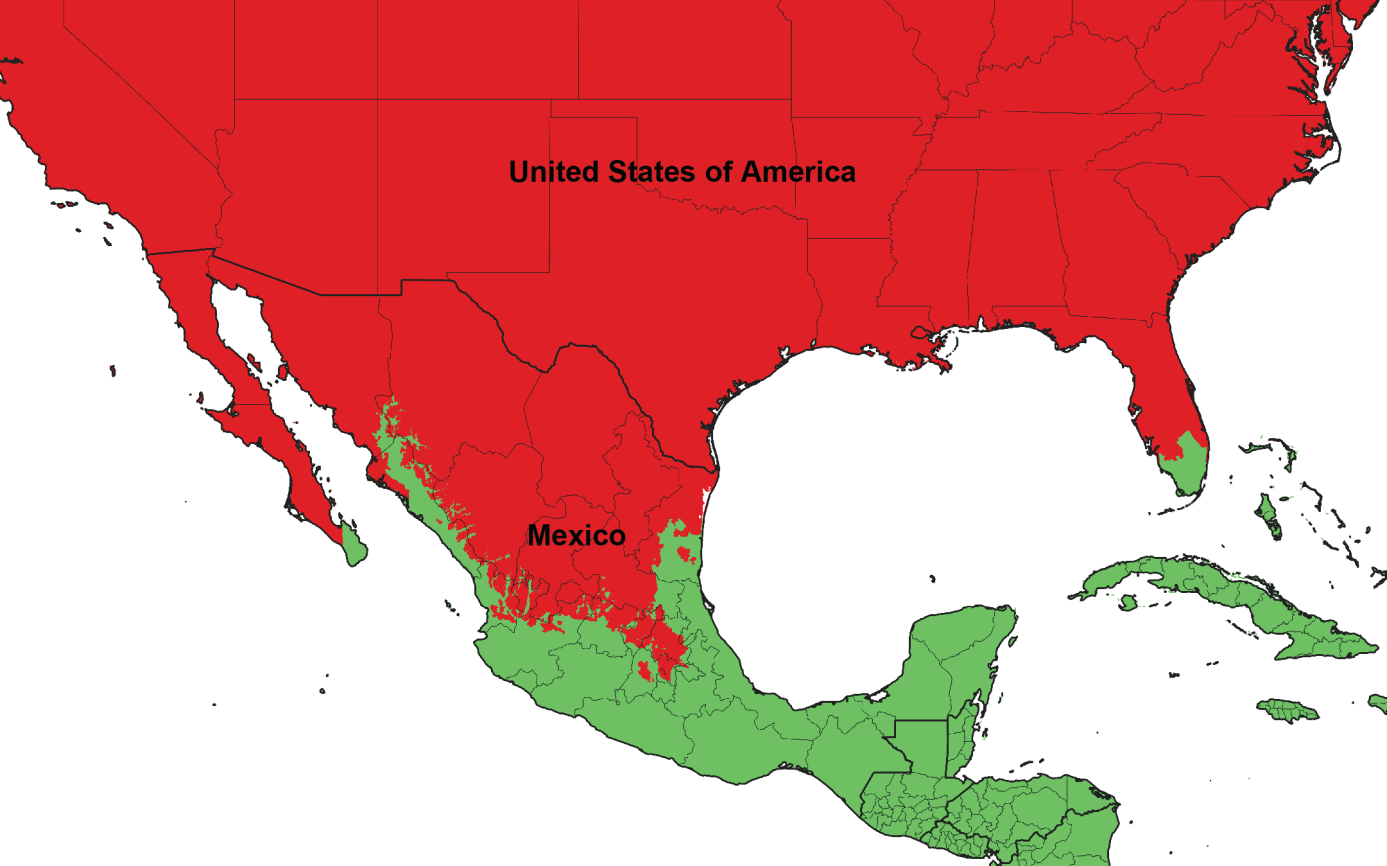
Closeup map showing the boundary between the Nearctic and Neotropic biogeographic realms. Countries with two biogeographic realms are named.

**Figure 3.**
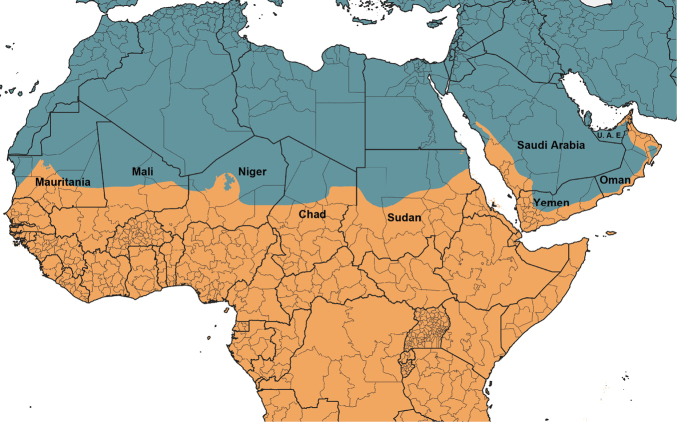
Closeup map showing the boundary between the Palaearctic and Afrotropic biogeographic realms. Countries with two biogeographic realms are named.

**Figure 4.**
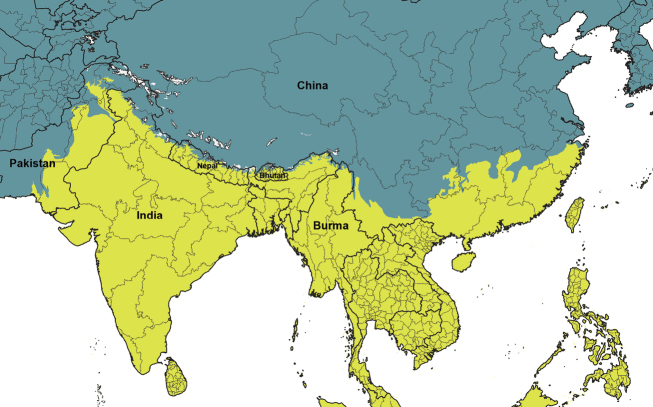
Closeup map showing the boundary between the Palaearctic and Indo-Malay biogeographic realms. Countries with two biogeographic realms are named.

**Figure 5.**
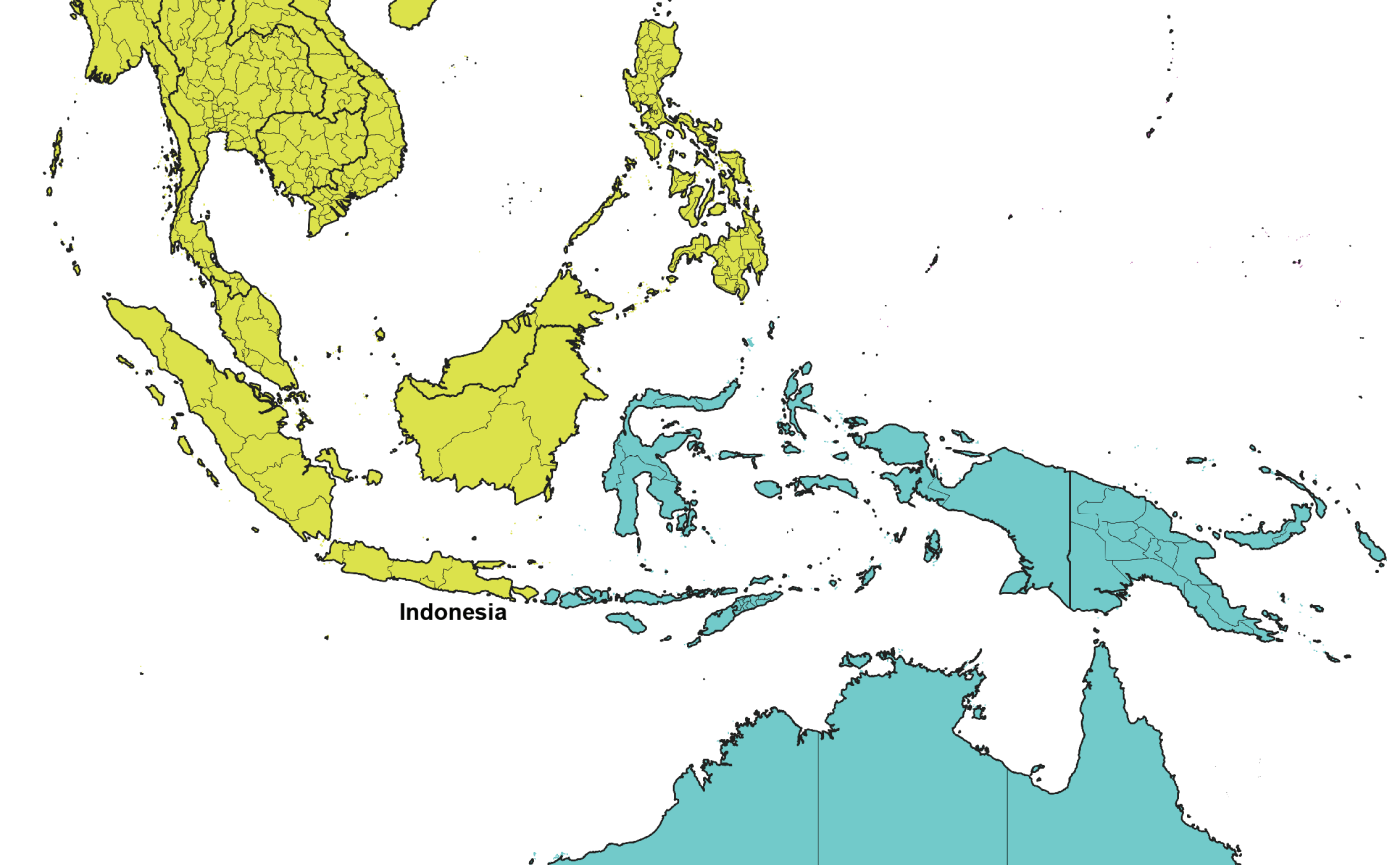
Closeup map showing the boundary between the Indo-Malay and Australasia biogeographic realms. Countries with two biogeographic realms are named.

The world’s major biogeographical realms, as defined by Olson et al. (2001), were used to record the overall distribution of each valid genus and subgenus (Table 2). The maps showing the borders of each realm (Figs 1–5) were produced with the free and open source software QGIS (version 3.10) using the layer Terrestrial Ecoregions of the World (WWF 2012).

### Publication dates

We tried to establish the date of publication of works listed in this catalogue as accurately as possible. In the References section, publication dates are listed after each reference in square brackets “[]” and preceded by DP (i.e., date of publication), unless only the year was found. These dates are either specific dates of publication (e.g., extracted from the works themselves or the journal wrappers, title pages, footers) or are the earliest known publication dates (e.g., extracted from recording journals or accounts of society meetings). In the latter case, the date is preceded by the word “by.” Sources of information for the dates of publication from societies, recording journals, and works are mentioned below.

#### Societies

**Acad Nat Sci Phil**: Academy of Natural Sciences, Philadelphia (Philadelphia, USA) [Proceedings];

**Acad Sci Fr**: Académie des Sciences (Paris, France) [Compte Rendus Hebdomadaires];

**Acad Sci St. Peters**: Académie Impériale des Sciences de St.-Pétersbourg (Saint Petersburg, Russia) [Bulletin Scientifique; Bulletin de la Classe historico-philologique];

**Amer Ant Soc**: American Antiquarian Society (Worcester, USA) [Proceedings];

**Amer Ent Soc**: American Entomological Society (Philadelphia, USA) [Proceedings];

**Amer Phil Soc**: American Philosophical Society (Philadelphia, USA) [Proceedings];

**Bost Soc Nat Hist**: Boston Society of Natural History (Boston, USA) [Proceedings];

**Ent Soc Lond**: Entomological Society of London (London, UK) [Transactions, Proceedings];

**Ent Ver Stettin**: Entomologischer Verein zu Stettin (Stettin, Germany [now Poland]) [Vereinsangelegenheiten];

**Nederl Ent Ver**: Nederlandsche Entomologische Vereniging (Leiden, Netherlands) [Inhouds-Opgave van Werken];

**Roy Soc Queensl**: Royal Society of Queensland (Brisbane, Australia) [Proceedings];

**Soc Ent Belg**: Société Entomologique de Belgique (Brussels, Belgium) [Annales, Comptes-Rendus];

**Soc Ent Fr**: Société Entomologique de France (Paris, France) [Annales, Bulletin];

**Soc Imp Nat Mosc**: Société Impériale des Naturalistes de Moscou (Moscow, Russia) [Bulletin].

#### Recording journals

**Allg Bibl Deutsch**: Allgemeine Bibliographie für Deutschland (Leipzig, Germany);

**Bibl Belg**: Bibliographie de la Belgique (Brussels, Belgium);

**Bibl Fr**: Bibliographie de la France (Paris, France);

**Bull Nord**: Bulletin du Nord, journal scientifique et littéraire, contenant: des mémoires et notices, des analyses et extraits d’ouvrages nouveaux; des variétés et mélanges, des annonces bibliographiques, etc., etc. (Moscow, Russia);

**Ent Nachr**: Entomologische Nachrichten (Berlin, Germany);

**Lit Centrbl**: Literarisches Centralblatt für Deutschland (Leipzig, Germany);

**Lit Ztg**: Literarische Zeitung (Berlin, Germany);

**Nat Nov**: Naturae Novitates (Berlin, Germany);

**Naturaliste**: Le Naturaliste (Paris, France);

**Pet Nouv Ent**: Petites Nouvelles Entomologiques (Paris, France);

**Rev Coleopt**: Revue Coléoptérologique (Brussels, Belgium);

**Zool Rec**: Zoological Record (London, UK).

#### Works reporting dates of publication

The following works included important information for dating the references: Kielmeyer and Jäger (1835), Guérin-Méneville (1838, 1858), Lefèvre (1885, 1895), Sharp (1892a), Sclater (1893), Oshanin (1910), Wheeler (1912), Sherborn (1934), Duncan (1937), Peavot (1937), F.H. Waterhouse (1937), ICZN (1957, 1958, 1999), F.M. Brown (1964), Raphael (1970), Cowan (1976), Kerzhner (1984), Hayek (1989), Baker (1996), Evenhuis (1997a, b, 2002, 2003, 2012, 2015, 2019a, b), Nagel and Schmidlin (2014), Merkl et al. (2008), Bouchard et al. (2011), Fujioka (2011), Bousquet (2016a, b, 2017), Williams (2017).

## Results

### Overall diversity and distribution

The family Tenebrionidae contains 4122 nomenclaturally available genus-group names (see Table 2 and List of available genus-group names in Tenebrionidae Latreille, 1802 below). Of the 2307 valid genera, 33 are extinct. A total of 761 subgenera are currently used as valid. The subfamily Pimeliinae contains the highest number of valid genera (n = 578) followed by Stenochiinae (n = 394), Tenebrioninae (n = 349), Blaptinae (n = 300), Lagriinae (n = 273), Alleculinae (n = 231) and Diaperinae (n = 128). The other four subfamilies contain few genera (Phrenapatinae = 28, Zolodininae = 3, Kuhitangiinae = 2 and Nilioninae = 1). Twenty valid genera could not be confidently placed in a subfamily and are included here as Tenebrionidae incertae sedis. The Afrotropic biogeographical realm is the most diverse with 1022 valid extant genus-group taxa (755 genera, 267 subgenera; see Table 2). The Palaearctic, Indo-Malay, and Neotropic realms follow with 925 (545 genera, 380 subgenera), 610 (501, 109) and 518 (428, 90) valid extant genus-group taxa respectively. The lowest number of valid extant genus-group taxa are in Australasia with 348 (310 genera, 38 subgenera), the Nearctic with 279 (206, 73) and Oceania with 62 (52, 10).

**Table 2.** List of nomenclaturally available genus-group names in the family Tenebrionidae presented in a classification framework. The presence of each valid extant genus and subgenus within a major biogeographical realm is indicated with the symbol “X” (usage of italics for the same symbol “*X*” indicates that a genus or subgenus is represented only by adventive species in a particular realm). * = status undetermined.

**Genus-group names**		**Nearctic**	**Neotropic**	**Palaearctic**	**Afrotropic**	**Indo-Malay**	**Australasia**	**Oceania**
**Pimeliinae Latreille, 1802**								
**Adelostomini Solier, 1834**								
*Acanthioides* Fairmaire, 1894					X			
*Acestophanus* Koch, 1950					X			
= *Acestus* Haag-Rutenberg, 1875								
*Adelostoma* Duponchel, 1827				X	X			
SGAdelostoma Duponchel, 1827				X	X			
= *Polyscopus* Waltl, 1835								
SGOmandelostoma Purchart, 2017					X			
SGPsaryphulum Koch, 1952					X			
SGZarudnionymus Semenov & Bogatchev, 1947				X	X			
= *Falsaspila* Koch, 1952								
= *Adelostomoides* Carl, 1991								
*Argasidus* Péringuey, 1899					X			
*Arthrochora* Gebien, 1938					X			
*Aspilomorpha* Koch, 1952					X			
*Basilewskyum* Koch, 1952					X			
*Brachymoschium* Fairmaire, 1896					X			
*Carinosella* Purchart, 2010					X			
*Cimicia* Fairmaire, 1891					X			
*Cimicichora* Koch, 1952					X			
*Cimiciopsis* Koch, 1952					X			
*Eurychora* Thunberg, 1789					X			
*Eurychorula* Koch, 1952					X			
*Eutichus* Haag-Rutenberg, 1875					X			
*Geophanus* Haag-Rutenberg, 1875					X			
*Herpsis* Haag-Rutenberg, 1875					X			
*Lepidochora* Koch, 1952					X			
*Lycanthropa* J. Thomson, 1860					X			
= *Zygas* Pascoe, 1866								
*Machlopsis* Pomel, 1871				X	X			
SGHidrosella Koch, 1952				X	X			
SGMachlopsis Pomel, 1871				X	X			
= *Hidrosis* Haag-Rutenberg, 1875								
*Phytolostoma* Koch, 1952					X			
*Platyphanus* Koch, 1952					X			
*Platysemodes* Strand, 1935					X			
= *Platysemus* Haag-Rutenberg, 1875								
*Pogonobasis* Solier, 1837				X	X			
= *Peristeptus* Haag-Rutenberg, 1875								
*Pogonocanta* Koch, 1952					X			
*Prunaspila* Koch, 1950					X			
= *Aspila* Fåhraeus, 1870								
*Psaryphis* Erichson, 1843					X			
*Serrichora* Koch, 1952					X			
*Smiliophanus* Koch, 1950					X			
= *Smiliotus* Haag-Rutenberg, 1875								
*Steptochora* Koch, 1952					X			
*Stips* Koch, 1950					X			
= *Steira* Westwood, 1837								
= *Stira* Agassiz, 1846								
*Stipsostoma* Koch, 1952					X			
*Symphochora* Koch, 1952					X			
**Adesmiini Lacordaire, 1859**								
*Adesmia* Fischer von Waldheim, 1822				X	X	X		
SGAdesmia Fischer von Waldheim, 1822				X	X			
= *Sarachus* Gistel, 1848								
SGAdesmina Reitter, 1916				X	X	X		
SGMacradesmia Kaszab, 1959				X	X			
= *Macradesmia* Löbl & Merkl, 2020								
SGMacropoda Solier, 1835					X			
SGMacropodesmia Löbl & Merkl, 2020					X			
SGOteroscelis Solier, 1835				X	X			
= *Heteroscelis* Agassiz, 1846								
SGOteroscelopsis Löbl & Merkl, 2020				X				
SGPhysosterna Dejean, 1834					X			
SGSomaladesmia Koch, 1944					X			
SGSpongesmia Bouchard & Bousquet, **subgen. nov.**					X			
SGZambesmia Bouchard & Bousquet, **subgen. nov.**					X			
*Alogenius* Gebien, 1910					X			
SGAequigula Penrith, 1979					X			
SGAlogenius Gebien, 1910					X			
= *Pedionomus* Haag-Rutenberg, 1875								
*Epiphysa* Dejean, 1834					X			
*Eustolopus* Gebien, 1938					X			
= *Entinopoda* Gebien, 1938								
*Metriopus* Solier, 1835					X			
SGCeradesmia Gebien, 1920					X			
SGCoeladesmia Reitter, 1916					X			
= *Peltadesmia* Kuntzen, 1916								
SGMetriopus Solier, 1835					X			
= *Iphthimera* Reitter, 1916								
*Onymacris* Allard, 1885					X			
*Orientocara* Koch, 1952					X			
*Physadesmia* Penrith, 1979					X			
*Renatiella* Koch, 1944					X			
= *Spongesmima* Koch, 1944								
*Stenocara* Solier, 1835					X			
SGArenacara Penrith, 1979					X			
SGCauricara Penrith, 1979					X			
SGStenocara Solier, 1835					X			
*Stenodesia* Reitter, 1916					X			
= *Cephaladesmia* Gebien, 1920								
= *Karroocara* Koch, 1952								
**Akidini Billberg, 1820**								
*Akis* Herbst, 1799				X				
= *Acidia* Illiger, 1804								
= *Stenopsis* Rafinesque, 1815								
= *Acis* Billberg, 1820								
*Cyphogenia* Solier, 1837				X				
SGCyphogenia Solier, 1837				X				
= *Eocyphogenia* G.S. Medvedev, 1968								
SGLechriomus Morawitz, 1865				X				
*Morica* Dejean, 1834				X				
*Sarothropus* Kraatz, 1865				X				
*Solskyia* Solsky, 1881				X				
**Anepsiini LeConte, 1862**								
*Anepsius* LeConte, 1851		X						
*Batuliodes* Casey, 1907		X						
*Batuliomorpha* Doyen, 1987		X						
*Batulius* LeConte, 1851		X						
**Asidini Fleming, 1821**								
*Afrasida* Wilke, 1922					X			
SGAfrasida Wilke, 1922					X			
SGArchasida Wilke, 1922					X			
*Alphasida* Escalera, 1905				X				
SGAlphasida Escalera, 1905				X				
SGBetasida Reitter, 1917				X				
= *Subalphasida* Escalera, 1928								
SGElongasida Escalera, 1906				X				
= *Cribrasida* Reitter, 1917								
= *Pseudoelongasida* Escalera, 1922								
SGGlabrasida Escalera, 1910				X				
= *Aplanasida* Reitter, 1917								
= *Aulonasida* Reitter, 1917								
= *Durasida* Reitter, 1917								
= *Gymnetasida* Reitter, 1917								
= *Melambasida* Reitter, 1917								
= *Mimelasida* Reitter, 1917								
= *Pedarasida* Reitter, 1917								
SGGranasida Reitter, 1917				X				
SGMachlasida Escalera, 1907				X				
= *Protomachlasida* Escalera, 1928								
*Amachla* Koch, 1962					X			
*Andremiopsis* Chatanay, 1913					X			
*Andremius* Fairmaire, 1903					X			
*Ardamimicus* Smith, 2013		X						
*Asida* Latreille, 1802				X				
SGAsida Latreille, 1802				X				
= *Dolichasida* Reitter, 1917								
= *Euryasida* Reitter, 1917								
= *Leptasida* Reitter, 1917								
= *Insulasida* Escalera, 1922								
= *Rugasida* Escalera, 1922								
SGGlobasida Escalera, 1905				X				
SGGracilasida Escalera, 1905				X				
= *Planasida* Escalera, 1907								
= *Trachasida* Reitter, 1917								
= *Granulasida* Escalera, 1922								
= *Pseudoplanasida* Escalera, 1921								
SGPeltasida Reitter, 1917				X				
SGPolasida Reitter, 1917				X				
= *Opatrasida* Escalera, 1922								
*Asidesthes* Fairmaire, 1900					X			
*Asidomorpha* Koch, 1962					X			
*Bartolozzia* Ferrer, 1998					X			
*Cardigenius* Solier, 1836			X					
SGCardigenius Solier, 1836			X					
= *Cardiogenius* Agassiz, 1846								
SGEllidoneus Wilke, 1922			X					
*Craniotus* LeConte, 1851		X						
*Cryptasida* Koch, 1962					X			
*Euryprosternum* Chatanay, 1914					X			
*Ferveoventer* Smith, 2013		X						
*Heterasida* Casey, 1912		X						
*Kochotella* Bouchard & Bousquet, **nom. nov.**					X			
= *Millotella* Koch, 1962								
*Leptasida* Chatanay, 1914					X			
*Litasida* Casey, 1912		X						
*Machla* Herbst, 1799					X			
= *Machloplasta* Wilke, 1922								
= *Pseudomachla* Wilke, 1922								
*Machleida* Fåhraeus, 1870					X			
= *Machloida* Rye, 1873								
*Machlomorpha* Péringuey, 1899					X			
SGAsidomachla Wilke, 1922					X			
SGMachlomorpha Péringuey, 1899					X			
*Machlophila* Wilke, 1924					X			
*Micrasida* Smith, 2013		X						
*Microschatia* Solier, 1836		X						
= *Pycnonotida* Casey, 1912								
= *Acroschatia* Wilke, 1922								
*Oxyge* Chatanay, 1914					X			
*Pelecyphorus* Solier, 1836		X						
SGAstrotus LeConte, 1858		X						
SGPelecyphorus Solier, 1836		X						
SGPleisiasida Smith, 2013		X						
= *Parasida* Casey, 1912								
SGPoliorcetes Champion, 1884		X						
SGSicharbas Champion, 1884		X						
SGStenosides Solier, 1836		X						
= *Pactostoma* LeConte, 1858								
= *Ologlyptus* Lacordaire, 1858								
SGUcalegon Champion, 1884		X						
SGZaleucus Champion, 1892		X						
= *Zamolxis* Champion, 1884								
*Philolithus* Lacordaire, 1858		X						
SGGlyptasida Casey, 1912		X						
SGGonasida Casey, 1912		X						
SGHerthasida Wilke, 1922		X						
SGPhilolithus Lacordaire, 1858		X						
SGTisamenes Champion, 1884		X						
*Prosodidius* Fairmaire, 1903					X			
*Pseudasida* Fairmaire, 1895					X			
*Saeculum* Kamiński, Kanda & Smith, 2021					X			
*Scotinesthes* Fairmaire, 1895					X			
= *Parecatus* Fairmaire, 1900								
*Scotinus* W. Kirby, 1819			X					
*Stenomorpha* Solier, 1836		X						
SGAsidina Casey, 1912		X						
SGAsidopsis Casey, 1912		X						
SGBothrasida Casey, 1912		X						
SGMegasida Casey, 1912		X						
SGNotiasida Casey, 1912		X						
SGPlatasida Casey, 1912		X						
SGPycnomorpha Motschulsky, 1870		X						
SGStenomorpha Solier, 1836		X						
= *Euschides* LeConte, 1851								
= *Psilomera* Motschulsky, 1870								
SGStethasida Casey, 1912		X						
SGTrichiasida Casey, 1912		X						
*Tamatasida* Koch, 1962					X			
**Boromorphini Skopin, 1978**								
*Boromorphus* Wollaston, 1854				X				
**Branchini LeConte, 1862**								
*Anectus* Horn, 1867			X					
*Branchus* LeConte, 1862		X	X					
*Oxinthas* Champion, 1884			X					
**Caenocrypticini Koch, 1958**								
*Caenocrypticoides* Kaszab, 1969			X					
*Caenocrypticus* Gebien, 1920					X			
SGCaenocapicus Endrödy-Younga, 1996					X			
SGCaenocrypticus Gebien, 1920					X			
= *Thorictophasis* Koch, 1950								
SGCryptocarpes Koch, 1952					X			
= *Lornamus* Koch, 1952								
SGPhyloradix Endrödy-Younga, 1996					X			
SGPsammotopulus Endrödy-Younga, 1996					X			
SGVernayella Koch, 1958					X			
**Ceratanisini Gebien, 1937**								
*Ceratanisus* Gemminger, 1870				X				
= *Anisocerus* Faldermann, 1837								
= *Apolites* Jacquelin du Val, 1861								
= *Haemerophygus* Baudi di Selve, 1876								
= *Seidlitzellus* Reitter, 1920								
= *Idastrandiella* Strand, 1929								
*Tenebriocephalon* Pic, 1925						X		
= *Klapperichia* Kaszab, 1954								
**Cnemeplatiini Jacquelin du Val, 1861**								
**Actizetina Watt, 1992**								
*Actizeta* Pascoe, 1875							X	
**Alaudina Aalbu, Caterino & Smith, 2018**								
*Alaudes* Horn, 1870		X						
**Cnemeplatiina Jacquelin du Val, 1861**								
*Cnemeplatia* Costa, 1847				X	X	X		
= *Autocera* Wollaston, 1857								
= *Cnemoplatia* Wollaston, 1865								
*Lepidocnemeplatia* Bousquet & Bouchard, 2018		X	X			X		
*Philhammus* Fairmaire, 1871				X	X			
SGPhilhamellus Kaszab, 1962				X				
SGPhilhammus Fairmaire, 1871				X	X			
= *Psilachnopus* Reitter, 1901								
= *Canariella* Uyttenboogaart, 1929								
**Rondoniellina Ferrer & Moragues, 2000**								
*Durandius* Kaszab, 1970						X		
*Rondoniella* Kaszab, 1970						X		
**Thorictosomatina Watt, 1992**								
*Thorictosoma* Lea, 1919							X	
*Wattiana* Matthews & Lawrence, 2005							X	
**Cnemodinini Gebien, 1910**								
*Cnemodinus* Cockerell, 1906		X						
= *Cnemodus* Horn, 1870								
**Coniontini G.R. Waterhouse, 1858**								
*Coelus* Eschscholtz, 1829		X						
= *Coelomorpha* Casey, 1890								
= *Pseudocoelus* Casey, 1908								
*Coniontis* Eschscholtz, 1829		X						
= *Coelotaxis* Horn, 1876								
= *Coniontellus* Casey, 1890								
= *Brachyontis* Casey, 1908								
= *Coniontides* Casey, 1908								
= *Crypticomorpha* Casey, 1908								
*Conisattus* Casey, 1895		X						
*Eusattus* LeConte, 1851		X						
= *Conipinus* LeConte, 1862								
= *Discodemus* LeConte, 1862								
= *Eusattodes* Casey, 1908								
= *Megasattus* Casey, 1908								
= *Nesostes* Casey, 1908								
= *Sphaeriontis* Casey, 1908								
= *Coelosattus* Blaisdell, 1927								
**Cossyphodini Wasmann, 1899**								
**Cossyphodina Wasmann, 1899**								
*Cossyphodes* Westwood, 1851				X	X			
= *Allocossyphodes* Andreae, 1961								
= *Hypercossyphodes* Andreae, 1961								
= *Metacossyphodes* Andreae, 1961								
= *Pachycossyphodes* Andreae, 1961								
*Paramellops* Andreae, 1961					X			
**Cossyphoditina Basilewsky, 1950**								
*Cossyphodites* Brauns, 1901					X			
**Esemephina Steiner, 1980**								
*Esemephe* Steiner, 1980			X					
**Paramellonina Andreae, 1961**								
*Cossyphodinus* Wasmann, 1899					X	X		
*Paramellon* C.O. Waterhouse, 1882				X	X	X		
**Cossyphodini** *incertae sedis*								
*Mimocossyphus* Pic, 1923				X				
**Cryptochilini Solier, 1841**								
**Calognathina Lacordaire, 1859**								
*Calognathus* Guérin-Méneville, 1836					X			
= *Callignathus* Agassiz, 1846								
**Cryptochilina Solier, 1841**								
*Cerasoma* Endrödy-Younga, 1989					X			
*Cryptochile* Latreille, 1828					X			
= *Phymatium* Billberg, 1820								
= *Cryptotrophus* Gistel, 1848								
*Cychrochile* Koch, 1953					X			
*Epipagus* Haag-Rutenberg, 1872					X			
*Horatoma* Solier, 1841					X			
= *Horatomodes* Haag-Rutenberg, 1872								
= *Saccophorus* Haag-Rutenberg, 1872								
= *Saccophorella* Strand, 1935								
= *Parapachynotela* Koch, 1952								
*Horatomella* Penrith & Endrödy-Younga, 1994					X			
*Orientochile* Penrith & Endrödy-Younga, 1994					X			
*Pachynotelus* Solier, 1841					X			
= *Fossilochile* Koch, 1952								
**Homebiina Endrödy-Younga, 1989**								
*Homebius* Endrödy-Younga, 1989					X			
**Vansoniina Koch, 1955**								
*Vansonium* Koch, 1950					X			
**Cryptoglossini LeConte, 1862**								
*Asbolus* LeConte, 1851		X						
*Cryptoglossa* Solier, 1837		X						
= *Centrioptera* Mannerheim, 1843								
= *Oochila* LeConte, 1862								
= *Amblycyphus* Motschulsky, 1870								
*Schizillus* Horn, 1874		X						
**Edrotini Lacordaire, 1859**								
*Armalia* Casey, 1907		X	X					
*Arthroconus* Solier, 1851			X					
= *Gymnognathus* Solier, 1851								
*Ascelosodis* Redtenbacher, 1868				X		X		
*Auchmobius* LeConte, 1851		X						
*Chilometopon* Horn, 1874		X						
= *Prometopion* Casey, 1907								
*Cryptadius* LeConte, 1851		X						
*Ditaphronotus* Casey, 1907			X					
*Edrotes* LeConte, 1851		X						
SGEdrotes LeConte, 1851		X						
= *Hedrotes* Gemminger, 1870								
SGOdrotes La Rivers, 1947		X						
*Emmenastrichus* Horn, 1894		X						
*Emmenides* Casey, 1907		X						
*Eremocantor* Smith & Wirth, 2016		X						
*Eschatomoxys* Blaisdell, 1935		X						
*Eurymetopon* Eschscholtz, 1831		X						
= *Eurymetopum* Agassiz, 1846								
*Falsoarthroconus* Kaszab, 1978			X					
*Garridoa* Marcuzzi, 1985			X					
*Hylithus* Guérin-Méneville, 1834			X					
= *Sciaca* Solier, 1835								
*Hylocrinus* Casey, 1907		X	X					
SGHylocrinus Casey, 1907		X	X					
SGLocrodes Casey, 1907		X	X					
SGParavius Casey, 1907		X						
*Kocakia* Kaszab, 1985			X					
= *Idiopsis* Kaszab, 1981								
*Koneus* Giraldo-Mendoza & Flores, 2019			X					
*Melanastus* Casey, 1907		X						
*Mencheres* Champion, 1884			X					
*Mesabates* Champion, 1884		X						
*Mesabatodes* Casey, 1907		X						
*Metoponium* Casey, 1907		X						
SGMetoponiopsis Casey, 1907		X						
SGMetoponium Casey, 1907		X						
*Micrarmalia* Casey, 1907			X					
*Micromes* Casey, 1907		X						
*Orthostibia* Blaisdell, 1923		X						
*Oxygonodera* Casey, 1907		X						
*Pachacamacius* Flores & Giraldo-Mendoza, 2019			X					
*Paraguania* Marcuzzi, 1953			X					
*Pescennius* Champion, 1884		X						
*Pimeliopsis* Champion, 1892		X						
*Posides* Champion, 1884		X						
*Prohylithus* Kaszab, 1964			X					
*Sechuranus* Flores & Giraldo-Mendoza, 2019			X					
*Soemias* Champion, 1884		X						
*Steriphanides* Casey, 1907		X						
*Steriphanus* Casey, 1907		X						
*Stibia* Horn, 1870		X						
= *Eutriorophus* Casey, 1924								
*Stictodera* Casey, 1907		X						
*Stomion* G.R. Waterhouse, 1845			X					
= *Stomium* Agassiz, 1846								
*Telabis* Casey, 1890		X						
*Telaponium* Blaisdell, 1923		X						
*Texaponium* Thomas, 1984		X						
*Tlascalinus* Casey, 1907		X						
*Trichiotes* Casey, 1907		X						
*Trientoma* Solier, 1835			X					
*Trimytantron* Ardoin, 1977			X					
= *Bielawskia* Marcuzzi, 1985								
*Trimytis* LeConte, 1851		X						
= *Pimalius* Casey, 1907								
*Triorophus* LeConte, 1851		X						
*Triphalopsis* Blaisdell, 1923		X						
*Triphalopsoides* Doyen, 1990			X					
*Triphalus* LeConte, 1866		X						
*Troglogeneion* Aalbu, 1985		X						
*Vizcainyx* Aalbu & Smith, 2020		X						
**Elenophorini Solier, 1837**								
**Elenophorina Solier, 1837**								
*Leptoderis* Billberg, 1820				X				
= *Elenophorus* Dejean, 1821								
= *Helenophorus* Gemminger, 1870								
**Megelenophorina Ferrer, 2015**								
*Megelenophorus* Gebien, 1910			X					
= *Cacicus* Dejean, 1834								
*Psammetichus* Latreille, 1828			X					
**Epitragini Blanchard, 1845**								
*Aspidolobus* Redtenbacher, 1868			X					
*Bothrotes* Casey, 1907		X	X					
*Conoecus* Horn, 1885		X						
*Cyrtomius* Casey, 1907			X					
SGCyrtomius Casey, 1907			X					
SGGrandicyrtomius Freude, 1967			X					
*Ecnomosternum* Gebien, 1928			X					
*Epitragella* Kulzer, 1958			X					
*Epitragodes* Casey, 1890		X	X					
*Epitragopsis* Casey, 1907			X					
*Epitragosoma* Brown & Triplehorn, 2002		X						
*Epitragus* Latreille, 1802			X					
SGEpitragus Latreille, 1802			X					
= *Lygophilus* Rafinesque, 1815								
= *Aethales* Dejean, 1834								
SGGobretus Freude, 1967			X					
SGSimilepitragus Freude, 1967			X					
*Eunotiodes* Casey, 1907			X					
*Geoborus* Blanchard, 1842			X					
= *Deroplatus* Solier, 1851								
*Hemasodes* Casey, 1907			X					
*Hypselops* Solier, 1851			X					
*Kaszabus* Freude, 1967			X					
*Lobometopon* Casey, 1907		X	X					
*Metopoloba* Casey, 1907		X						
*Nyctopetus* Guérin-Méneville, 1831			X					
*Omopheres* Casey, 1907			X					
SGMicroomopheres Freude, 1993			X					
SGOmopheres Casey, 1907			X					
*Ortheolus* Casey, 1907			X					
*Parepitragus* Casey, 1907			X					
*Pechalius* Casey, 1907		X	X					
= *Epitragoma* Casey, 1907								
*Pectinepitragus* Pic, 1927			X					
*Penaus* Freude, 1968			X					
*Phegoneus* Casey, 1907		X	X					
SGPectphegoneus Freude, 1968			X					
SGPhegoneus Casey, 1907		X	X					
SGSchoeniphegoneus Freude, 1968			X					
*Phitophilus* Guérin-Méneville, 1831			X					
*Polemiotus* Casey, 1907		X	X					
*Pseudortheolus* Freude, 1968			X					
*Pseudothinobatis* Bouchard & Bousquet, **gen. nov.**			X					
*Schoenicus* LeConte, 1866		X	X					
*Stictodere* Gebien, 1928			X					
= *Stictoderia* Gebien, 1937								
*Tapinocomus* Gebien, 1928			X					
*Tydeolus* Champion, 1884			X					
**Erodiini Billberg, 1820**								
*Ammodoides* Lesne, 1915					X			
*Ammozoides* Kaszab, 1979				X				
*Ammozoum* Semenov, 1891				X				
*Amnodeis* Miller, 1858				X				
*Anodesis* Solier, 1834					X			
*Apentanodes* Reitter, 1914				X		X		
SGApentanodes Reitter, 1914				X				
SGRasphytus Kulzer, 1956				X		X		
*Arthrodeis* Solier, 1834				X	X			
SGApentanes Reitter, 1914				X				
SGArthrodeis Solier, 1834				X	X			
= *Arthrodes* Agassiz, 1846								
SGArthrodinus Reitter, 1900				X				
SGKocheria Antoine, 1946				X	X			
*Arthrodibius* Lesne, 1915				X	X			
SGArthrodibius Lesne, 1915					X			
SGErodibius Löbl, Bouchard, Merkl & Bousquet, 2020				X	X			
SGHelioarthrodibius Koch, 1960					X			
*Arthrodion* Lesne, 1915					X			
*Arthrodosis* Reitter, 1900				X				
*Arthrodygmus* Reitter, 1914						X		
*Arthrohyalosis* Kaszab, 1979				X				
*Arthrohyalus* Koch, 1943				X				
*Bulbulus* Lesne, 1915				X				
*Capricephalius* Koch, 1943				X				
*Diaphanidus* Reitter, 1900				X				
SGDiaphanidus Reitter, 1900				X				
= *Globularthrodosis* Kaszab, 1979								
SGPseudodiaphanidus Bogatchev, 1950				X				
*Diodontes* Solier, 1834					X			
*Erodinus* Reitter, 1900*								
*Erodiontes* Reitter, 1914				X				
= *Iranarthrodosis* Kaszab, 1959								
*Erodius* Fabricius, 1775				X				
SGDimeriseis Solier, 1834				X				
SGDirosis Miller, 1858				X				
SGEodirosis Kwieton, 1980				X				
SGErodius Fabricius, 1775				X				
= *Cephacerus* Rafinesque, 1815								
= *Acantophorus* Billberg, 1820								
= *Herodius* Agassiz, 1846								
SGZophoserodius Reitter, 1914				X				
*Farsarthrosis* Kaszab, 1979				X				
*Foleya* Peyerimhoff, 1916				X				
*Histeromimus* Gahan, 1895				X				
*Histeromorphus* Kraatz, 1865					X			
*Hyalarthrodosis* Kaszab, 1979				X				
*Hyalerodius* Kaszab, 1979				X				
*Iranerodius* Kaszab, 1959				X				
*Leptonychoides* Schawaller, 1990				X				
*Leptonychus* Chevrolat, 1833				X				
*Piestognathoides* Kaszab, 1981				X				
*Piestognathus* P.H. Lucas, 1858				X				
*Somalammodes* Koch, 1943					X			
*Spyrathus* Kraatz, 1865				X		X		
**Evaniosomini Lacordaire, 1859**								
*Achanius* Erichson, 1847			X					
SGAchanius Erichson, 1847			X					
SGAmbigatus Fairmaire, 1892			X					
*Aryenis* Bates, 1868			X					
*Chorasmius* Bates, 1868			X					
*Evaniosomus* Guérin-Méneville, 1834			X					
= *Nochelius* Gistel, 1848								
*Evelina* J. Thomson, 1860			X					
*Melaphorus* Guérin-Méneville, 1834			X					
= *Stenholma* Solier, 1835								
= *Raptor* Gistel, 1848								
= *Melanophorus* Lacordaire, 1859								
*Oppenheimeria* Koch, 1952					X			
*Vaniosus* Kulzer, 1956			X					
**Falsomycterini Gebien, 1910**								
*Falsomycterus* Pic, 1907			X					
*Pteroctenus* Kirsch, 1866			X					
**Idisiini G.S. Medvedev, 1973**								
*Idisia* Pascoe, 1866				X				
**Klewariini Gebien, 1910**								
*Klewaria* Reitter, 1910				X				
**Lachnogyini Seidlitz, 1894**								
**Lachnodactylina Reitter, 1904**								
*Lachnodactylus* Seidlitz, 1898				X				
= *Lachnopus* Seidlitz, 1894								
**Lachnogyina Seidlitz, 1894**								
*Lachnogya* Ménétriés, 1849				X		X		
**Netuschiliina Ferrer & Yvinec, 2004**								
*Netuschilia* Reitter, 1904				X				
**Leptodini Lacordaire, 1859**								
*Leptodes* Dejean, 1834				X				
SGLeptodes Dejean, 1834				X				
= *Leptodinopsis* Kaszab, 1959								
SGLeptodopsis Haag-Rutenberg, 1879				X				
SGMesoleptodes G.S. Medvedev & Iljina, 2007				X				
SGParaleptodes G.S. Medvedev, 1967				X				
SGProleptodes G.S. Medvedev, 1967				X				
*Tapenopsis* Solier, 1843				X				
= *Tapinopsis* Agassiz, 1846								
**Nycteliini Solier, 1834**								
*Auladera* Solier, 1836			X					
= *Aulacodera* Agassiz, 1846								
*Callyntra* Solier, 1836			X					
*Entomobalia* Flores & Triplehorn, 2002			X					
*Entomoderes* Solier, 1836			X					
*Epipedonota* Solier, 1836			X					
*Gyriosomus* Guérin-Méneville, 1834			X					
= *Brachygenius* Dejean, 1836								
*Mitragenius* Solier, 1836			X					
*Nyctelia* Berthold, 1827			X					
= *Nyctelius* Guérin-Méneville, 1827								
= *Nyctelioma* Casey, 1908								
*Patagonogenius* Flores, 1999			X					
*Pilobalia* Burmeister, 1875			X					
*Psectrascelis* Solier, 1836			X					
= *Cerostena* Solier, 1836								
= *Stenocera* Agassiz, 1846								
*Scelidospecta* Kulzer, 1954			X					
**Nyctoporini Lacordaire, 1859**								
*Nyctoporis* Eschscholtz, 1831		X						
= *Emeax* Pascoe, 1866								
= *Enneacoides* Fairmaire, 1881								
**Phrynocarenini Gebien, 1928**								
*Phrynocarenum* Gebien, 1928			X					
= *Pseudoscotobius* Kulzer, 1955								
**Physogasterini Lacordaire, 1859**								
*Entomochilus* Gay & Solier, 1843			X					
*Philorea* Erichson, 1834			X					
= *Polpocara* Solier, 1843								
*Physogaster* Lacordaire, 1830			X					
*Physogasterinus* Kaszab, 1981			X					
*Pimelosomus* Burmeister, 1875			X					
**Pimeliini Latreille, 1802**								
*Afghanopachys* Kwieton, 1978				X				
*Allotadzhikistania* Bogatchev, 1960				X				
*Apatopsis* Semenov, 1891				X				
*Argyradelpha* G.S. Medvedev, 2005				X				
*Argyrophana* Semenov, 1889				X				
*Astorthocnemis* Lillig & Pavlíček, 2002				X				
*Balachowskya* Peyerimhoff, 1928				X				
*Bogatshevia* G.S. Medvedev & Iwan, 2006				X				
= *Achaemenes* Bogatchev, 1949								
*Cyclocnera* Leo, 2018				X				
*Diesia* Fischer von Waldheim, 1820				X				
= *Diesiola* Skopin, 1961								
*Dietomorpha* Reymond, 1938				X				
*Earophanta* Semenov, 1903				X				
= *Earophila* Semenov, 1903								
= *Earophilina* Strand, 1917								
*Euryostola* Reitter, 1893				X				
*Euthriptera* Reitter, 1893				X				
*Gedeon* Reiche & Saulcy, 1857				X				
*Habrobates* Semenov, 1903				X				
*Habrochiton* Semenov, 1907				X				
*Homopsis* Semenov, 1893				X				
*Idiesa* Reitter, 1893				X				
*Iranolasiostola* Pierre, 1968				X				
*Iranopachyscelis* Pierre, 1968				X				
*Kawiria* Schuster, 1935				X				
*Lasiostola* Dejean, 1834				X				
SGCentrocnemita Strand, 1935				X				
= *Centrocnemis* Kraatz, 1882								
SGLasiocnema G.S. Medvedev, 1993				X				
SGLasiograna G.S. Medvedev, 1993				X				
SGLasiostola Dejean, 1834				X				
= *Pseudopimelia* Gebler, 1859								
*Leucolaephus* P.H. Lucas, 1859				X				
= *Mecopisthopus* Karsch, 1881								
*Mantichorula* Reitter, 1889				X				
*Meladiesia* Reitter, 1909				X				
*Ocnera* Fischer von Waldheim, 1822				X				
= *Brachycyphus* Gebler, 1859								
*Pachylodera* Quedenfeldt, 1890				X				
*Pachyscelina* Kwieton, 1978				X				
*Pachyscelis* Solier, 1836				X				
SGPachyscelis Solier, 1836				X				
= *Brachyscelis* Dejean, 1834								
SGParapachyscelis Kwieton, 1978				X				
*Paraplatyope* Löbl, Bouchard, Merkl & Bousquet, 2020				X				
*Pelorocnemis* Solsky, 1876				X				
*Phymatiotris* Solier, 1836				X				
= *Graecopachys* Skopin, 1968								
*Pimelia* Fabricius, 1775				X	X	X		
SGAmblyptera Solier, 1836				X				
SGAmblypteraca Mas-Peinado, Buckley, Ruiz & García-París, 2018				X				
SGAphanaspis Wollaston, 1864				X				
SGCamphonota Solier, 1836				X				
= *Eurypimelia* Reitter, 1915								
SGChaetotoma Motschulsky, 1860				X				
SGEcphoroma Solier, 1836				X				
SGHispanomelia Mas-Peinado, Buckley, Ruiz & García-París, 2018				X				
SGIberomelia Mas-Peinado, Buckley, Ruiz & García-París, 2018				X				
SGItalomelia Mas-Peinado, Buckley, Ruiz & García-París, 2018				X				
SGMagrebmelia Mas-Peinado, Buckley, Ruiz & García-París, 2018				X				
SGMassadraamelia Mas-Peinado, Buckley, Ruiz & García-París, 2018				X				
SGMelanostola Solier, 1836				X				
= *Balius* Gistel, 1848								
SGPimelia Fabricius, 1775				X	X	X		
= *Pimidia* Rafinesque, 1815								
= *Agelarches* Gistel, 1848								
= *Doderoella* Schuster, 1926								
SGPseudamblyptera Pierre, 1985				X				
*Pimeliocnera* Reitter, 1909				X				
*Pimelipachys* Skopin, 1962				X				
*Pisterotarsa* Motschulsky, 1860				X				
= *Sympiezocnemis* Solsky, 1876								
= *Piesterotarsa* Sénac, 1884								
*Platyesia* Skopin, 1971				X				
*Platyope* Fischer von Waldheim, 1820				X				
*Podhomala* Solier, 1836				X				
SGPodhomala Solier, 1836				X				
= *Podomala* Agassiz, 1846								
= *Uriela* Reitter, 1887								
= *Pterocomodes* Reitter, 1901								
SGUrielina Reitter, 1888				X				
*Polpogenia* Solier, 1836					X			
*Prionotheca* Dejean, 1834				X	X			
*Przewalskia* Semenov, 1893				X				
*Pseudopachyscelis* Skopin, 1968				X				
*Pseudoplatyope* Pierre, 1964				X				
*Pseudopodhomala* Schuster, 1938				X				
= *Pseudopodhomalina* Kaszab, 1960								
= *Gedrosia* Bogatchev, 1961								
*Pseudostorthocnemis* Gridelli, 1952				X				
*Pterocoma* Dejean, 1834				X				
SGDzhungaropterocoma Skopin, 1974				X				
SGEupterocoma Skopin, 1974				X				
SGHemipterocoma Skopin, 1974				X				
SGMesopterocoma Skopin, 1974				X				
SGMongolopterocoma Skopin, 1974				X				
SGNeopterocoma Skopin, 1974				X				
SGPachypterocoma Skopin, 1974				X				
SGParapterocoma Skopin, 1974				X				
SGPoopterocoma Skopin, 1974				X				
SGPropterocoma Skopin, 1974				X				
SGPseudopterocoma Skopin, 1974				X				
SGPterocoma Dejean, 1834				X				
SGSubpterocoma Bouchard & Bousquet, **nom. nov.**				X				
= *Pseudopimelia* Motschulsky, 1860								
*Pterolasia* Solier, 1836				X	X			
*Scelace* Marseul, 1887				X				
= *Pachyscelodes* Sénac, 1887								
*Spectrocnera* Kwieton, 1981					X			
*Stalagmoptera* Solsky, 1876				X				
= *Arnoldiola* Semenov & Bogatchev, 1940								
*Sternocnera* Skopin, 1964				X				
*Sternodes* Fischer von Waldheim, 1837				X				
*Sternoplax* Frivaldszky, 1890				X				
SGMesosternoplax Skopin, 1973				X				
SGPachysternoplax Skopin, 1973				X				
SGParasternoplax Skopin, 1973				X				
SGPseudosternoplax Skopin, 1973				X				
SGSternoplax Frivaldszky, 1890				X				
= *Pseudeuthriptera* Bogatchev & Kryzhanovskii, 1955								
*Sternotrigon* Skopin, 1973				X				
*Storthocnemis* Karsch, 1881				X	X			
*Tadzhikistania* Bogatchev, 1960				X				
*Thriptera* Solier, 1836				X	X			
*Trachyderma* Latreille, 1828				X	X		*X*	
SGAtrachyderma Skopin, 1962				X				
SGTrachyderma Latreille, 1828				X	X		*X*	
*Trigonocnera* Reitter, 1893				X				
*Trigonopachys* Skopin, 1968				X				
*Trigonoscelis* Dejean, 1834				X				
SGChinotrigon Skopin, 1973				X				
SGEchinotrigon Skopin, 1973				X				
SGTrigonoscelis Dejean, 1834				X				
*Waterhousia* Skopin, 1973				X				
= *Heinrichesia* Carl, 2000								
**Praociini Eschscholtz, 1829**								
*Antofagapraocis* Flores, 2000			X					
*Asidelia* Fairmaire, 1905			X					
*Calymmophorus* Solier, 1841			X					
= *Calymmatophorus* Gemminger, 1870								
*Eutelocera* Solier, 1841			X					
= *Euteleocera* Agassiz, 1846								
*Falsopraocis* Kulzer, 1958			X					
*Gyrasida* Koch, 1962			X					
*Neopraocis* Kulzer, 1958			X					
*Parapraocis* Flores & Giraldo, 2020			X					
*Patagonopraocis* Flores & Chani-Posse, 2005			X					
*Pilobaloderes* Kulzer, 1958			X					
*Platesthes* G.R. Waterhouse, 1845			X					
*Platyholmus* Dejean, 1834			X					
= *Platyolmus* Burmeister, 1875								
= *Edrotopus* Haag-Rutenberg, 1877								
*Praocidia* Fairmaire, 1904			X					
*Praocis* Eschscholtz, 1829			X					
SGAnthrasomus Guérin-Méneville, 1834			X					
= *Anthracosomus* Agassiz, 1846								
SGFilotarsus Solier, 1841			X					
SGHemipraocis Flores & Pizarro-Araya, 2014			X					
SGMesopraocis Flores & Pizarro-Araya, 2014			X					
SGOrthogonoderes Solier, 1841			X					
= *Aulacus* Gray, 1832								
= *Eurygona* Laporte, 1840								
SGPostpraocis Flores & Pizarro-Araya, 2014			X					
SGPraocida Flores & Pizarro-Araya, 2014			X					
SGPraocis Eschscholtz, 1829			X					
= *Arctylus* Dejean, 1834								
SGPraonoda Flores & Pizarro-Araya, 2014			X					
*Thylacoderes* Solier, 1843			X					
**Sepidiini Eschscholtz, 1829**								
**Hypomelina Koch, 1955**								
*Argenticrinis* Louw, 1979					X			
*Bombocnodulus* Koch, 1955					X			
*Brinckia* Koch, 1962					X			
*Hypomelus* Solier, 1843					X			
*Iugidorsum* Louw, 1979					X			
*Sulcipectus* Louw, 1979					X			
*Trachynotidus* Péringuey, 1899					X			
*Triangulipenna* Louw, 1979					X			
*Uniungulum* Koch, 1962					X			
**Molurina Solier, 1834**								
*Amiantus* Fåhraeus, 1870					X			
*Arturium* Koch, 1951					X			
*Brachyphrynus* Fairmaire, 1882					X			
*Chiliarchum* Koch, 1953					X			
*Dichtha* Haag-Rutenberg, 1871					X			
*Distretus* Haag-Rutenberg, 1871					X			
SGDistretus Haag-Rutenberg, 1871					X			
SGPerdistretus Koch, 1953					X			
*Euphrynus* Fairmaire, 1897					X			
*Glyptophrynus* Fairmaire, 1899					X			
*Huilamus* Koch, 1953					X			
*Melanolophus* Fairmaire, 1882					X			
*Moluris* Latreille, 1802					X			
= *Physodera* Solier, 1843								
*Ocnodes* Fåhraeus, 1870					X			
= *Phanerotoma* Solier, 1843								
= *Phanerotomea* Koch, 1958								
*Phrynocolus* Lacordaire, 1859					X			
SGPhrynocolopsis Koch, 1951					X			
SGPhrynocolus Lacordaire, 1859					X			
= *Cryptogenius* Solier, 1843								
SGSpinophrynus Koch, 1951					X			
*Phrynophanes* Koch, 1951					X			
*Physophrynus* Fairmaire, 1882					X			
*Psammodes* W. Kirby, 1819					X			
= *Piesomera* Solier, 1843								
= *Psammodophysis* Péringuey, 1899								
= *Parmularia* Koch, 1955								
*Psammophanes* Lesne, 1922					X			
SGPsammolophus Koch, 1953					X			
SGPsammophanes Lesne, 1922					X			
SGPsammophrynopsis Koch, 1953					X			
SGPsammophrynus Koch, 1953					X			
SGPsammostretus Koch, 1953					X			
SGPsammotyriopsis Koch, 1953					X			
SGSomalarabes Koch, 1953					X			
*Psammoryssus* Kolbe, 1886					X			
*Psammotyria* Koch, 1953					X			
*Stridulomus* Koch, 1955					X			
*Tarsocnodes* Gebien, 1920					X			
*Tibiocnodes* ​Gearner & Kamiński, 2021					X			
*Toktokkus* Kamiński & Gearner, 2020					X			
*Tuberocnodes* ​Gearner & Kamiński, 2021					X			
**Oxurina Koch, 1955**								
*Decoriplus* Louw, 1979					X			
*Miripronotum* Louw, 1979					X			
*Namibomodes* Koch, 1952					X			
*Oxura* W. Kirby, 1819					X			
= *Oxyura* Agassiz, 1846								
*Palpomodes* Koch, 1952					X			
SGPalpomodes Koch, 1952					X			
SGPygmaeodes Koch, 1952					X			
*Pterostichula* Koch, 1952					X			
SGPterostichula Koch, 1952					X			
SGRipicolodes Koch, 1952					X			
*Stenethmus* Gebien, 1937					X			
*Synhimba* Koch, 1952					X			
**Sepidiina Eschscholtz, 1829**								
*Dimoniacis* Koch, 1958					X			
*Echinotus* Solier, 1843					X			
*Peringueyia* Koch, 1958					X			
*Sepidiopsis* Gestro, 1892					X			
*Sepidiostenus* Fairmaire, 1884					X			
= *Sepidiacis* Fairmaire, 1884								
*Sepidium* Fabricius, 1775				X	X			
= *Espidium* Rafinesque, 1815								
*Vieta* Laporte, 1840				X	X			
= *Dymonus* Solier, 1843								
= *Divieta* Reitter, 1914								
*Vietomorpha* Fairmaire, 1887					X			
**Trachynotina Koch, 1955**								
*Cyrtoderes* Dejean, 1834					X			
= *Phligra* Laporte, 1840								
*Epairopsis* Koch, 1955					X			
*Ethmus* Haag-Rutenberg, 1873					X			
SGEthmomerus Koch, 1954					X			
SGEthmophobes Koch, 1954					X			
SGEthmus Haag-Rutenberg, 1873					X			
= *Tynthlobia* Fairmaire, 1888								
*Histrionotus* Koch, 1955					X			
*Microphligra* Koch, 1955					X			
*Ossiporis* Pascoe, 1866					X			
= *Epairops* Fåhraeus, 1870								
*Oxycerus* Koch, 1955					X			
*Somaticus* Hope, 1841					X			
SGAcromaticus Koch, 1955					X			
SGBechuanitis Koch, 1955					X			
SGCeromelaephus Koch, 1955					X			
SGClinocranion Solier, 1843					X			
SGDiacis Koch, 1955					X			
SGSomaticus Hope, 1841					X			
= *Gonopterus* Solier, 1843								
SGTracheloeum Hope, 1841					X			
SGTrachyderes Koch, 1955					X			
SGTrichotrachys Koch, 1955					X			
SGTrichotrichus Koch, 1955					X			
SGTropitrachys Koch, 1955					X			
*Trachynotus* Latreille, 1828					X			
= *Hipomelus* Dejean, 1834								
*Trichethmus* Gebien, 1937					X			
**Stenosini Schaum, 1859 (1834)**								
**Araeoschizina Casey, 1907**								
*Araeoschizus* LeConte, 1851		X						
**Dichillina Reitter, 1916**								
*Afghanillus* Kaszab, 1960				X				
*Anchomma* LeConte, 1858		X						
*Aspidocephalus* Motschulsky, 1839				X				
*Dichillus* Jacquelin du Val, 1860				X				
SGDichillesthes Reitter, 1916				X				
SGDichillinus Reitter, 1916				X				
SGDichillocerus Reitter, 1916				X				
SGDichillodontus Reitter, 1916				X				
SGDichillomessor Reitter, 1916				X		X		
SGDichillus Jacquelin du Val, 1860				X				
SGMyrmecodichillus Kaszab, 1960				X				
SGPushtunillus G.S. Medvedev, 1995				X				
*Discopleurus* Lacordaire, 1859			X					
= *Pleurophorus* Solier, 1851								
*Herbertfranzia* Kaszab, 1973						X		
*Herbertfranziella* Kaszab, 1973				X		X		
*Hexagonochilus* Solier, 1851			X					
*Indochillus* Koch, 1941						X		
*Microtelus* Solier, 1838				X				
*Nepalofranziella* Fouquè, 2013						X		
*Oogaster* Faldermann, 1837				X				
*Pseudethas* Fairmaire, 1896				X		X		
SGPseudethas Fairmaire, 1896				X		X		
= *Schizillus* Wasmann, 1899								
= *Dischizillus* Wasmann, 1902								
SGStenillus Blair, 1927				X				
*Pseudochillus* Fouquè, 2015						X		
SGKaszabochillus Fouquè, 2015						X		
SGMicropseudochillus Fouquè, 2015						X		
SGPseudochillus Fouquè, 2015						X		
*Reitterella* Semenov, 1891				X				
**Harvengiina Ferrer, 2004**								
*Harvengia* Ferrer, 2004						X		
**Platamodina Reitter, 1900**								
*Fitzsimonsium* Koch, 1962					X			
= *Fitzsimonsia* Koch, 1955								
*Microblemma* Semenov, 1889				X				
*Platamodes* Ménétriés, 1849				X				
**Stenosina Schaum, 1859 (1834)**								
*Anethas* Jakobson, 1924					X			
SGAfrethas Koch, 1962					X			
SGAnethas Jakobson, 1924					X			
= *Pseudethas* Fairmaire, 1898								
SGTetrethas Koch, 1962					X			
*Caribanosis* Nabozhenko, Kirejtshuk, Merkl, Varela, Aalbu & Smith, 2016			X					
*Ecnomoderes* Gebien, 1928			X					
*Ethas* Pascoe, 1862						X		
*Eutagenia* Reitter, 1886				X				
*Gebieniella* Koch, 1940						X		
*Grammicus* G.R. Waterhouse, 1845			X					
*Indostola* G.S. Medvedev, 1991						X		
*Itampolis* Koch, 1962					X			
*Microtelopsis* Koch, 1940				X		X		
SGExtetranosis Koch, 1940						X		
SGHypermicrotelopsis Koch, 1940				X				
SGMicrotelopsis Koch, 1940						X		
SGTetranosis G.S. Medvedev, 1995				X		X		
*Mitotagenia* Reitter, 1916				X	X			
*Perdicus* Fairmaire, 1899					X			
*Renefouqueosis* Aalbu, Smith, Kanda & Bouchard, 2017			X					
*Schizaraeus* Kulzer, 1955			X					
*Schusteriella* Koch, 1940					X			
*Stenosethas* Kaszab, 1975						X		
*Stenosis* Herbst, 1799				X	X	X		
SGAfronosis G.S. Medvedev, 1995					X			
SGBurmanosis G.S. Medvedev, 1995						X		
SGIndianosis Koch, 1941						X		
SGStenosidops Koch, 1940					X			
SGStenosis Herbst, 1799				X	X	X		
= *Tagenia* Latreille, 1802								
*Tagenostola* Reitter, 1916				X		X		
*Tetranillus* Wasmann, 1899				X		X		
*Timosmithus* Ardoin, 1974					X			
**Typhlusechina Casey, 1907**								
*Typhlusechus* Linell, 1897		X						
**Stenosini *incertae sedis***								
†*Miostenosis* Wickham, 1913								
**Tentyriini Eschscholtz, 1831**								
*Abigopsis* Escalera, 1914				X				
*Afrinus* Fairmaire, 1888					X			
SGAfrinus Fairmaire, 1888					X			
= *Nerina* Lacordaire, 1859								
SGGynandrocera Gebien, 1920					X			
SGPalpafrina Koch, 1950					X			
*Alcinoeta* Strand, 1929				X				
= *Alcinoe* Ménétriés, 1849								
= *Allodengitha* Bogatchev, 1963								
*Amblycarenum* Gebien, 1910				X				
= *Amblycara* Fairmaire, 1893								
*Ammogiton* Peyerimhoff, 1920				X				
*Anatolica* Eschscholtz, 1831				X				
SGAnatolica Eschscholtz, 1831				X				
SGEurepipleura Bogdanov-Katjkov, 1915				X				
*Aphrotus* Péringuey, 1904					X			
= *Xenus* Péringuey, 1899								
*Archinamibia* Koch, 1952					X			
*Asphaltesthes* Kraatz, 1865					X			
SGAsphaltesthes Kraatz, 1865					X			
SGTagenesthes Koch, 1941					X			
*Broomium* Koch, 1950					X			
*Calyptopsis* Solier, 1835				X				
= *Choristopsis* Kraatz, 1865								
*Cantopipleurus* Koch, 1943					X			
*Capnisiceps* Chatanay, 1914					X			
*Catomulus* Reitter, 1897				X				
*Cimipsa* Peyerimhoff, 1911				X				
= *Cirta* Gemminger, 1870								
*Colposcelis* Dejean, 1834				X				
SGColposcelis Dejean, 1834				X				
SGColposceloides Schuster, 1940				X				
SGColposcythis Reitter, 1889				X				
SGScelocolpis Reitter, 1900				X				
SGTurcmenicola Bogatchev, 1952				X				
*Colposphena* Semenov, 1889				X				
*Craniosphena* Koch, 1962					X			
*Cychrachna* Koch, 1950					X			
*Cyphostethe* Marseul, 1867				X	X			
SGApterocyphostethe Kaszab, 1962				X				
SGCyphostethe Marseul, 1867				X	X			
= *Asphena* Semenov, 1889								
SGCyphostethoides Löbl & Merkl, 2020				X				
SGDerostethe Koch, 1950					X			
SGHimastethe Koch, 1950					X			
SGTrichostethe Koch, 1950					X			
*Dailognatha* Steven, 1828				X				
= *Delognatha* Agassiz, 1846								
*Dengitha* Reitter, 1887				X				
*Derosphaerius* Westwood, 1881					X			
SGApterosphaeria Koch, 1950					X			
SGDerosphaerius Westwood, 1881					X			
= *Derostrophus* Fairmaire, 1888								
= *Oatesius* Westwood, 1889								
SGEpityria Koch, 1950					X			
*Dichomma* Solier, 1835				X				
*Dividiopsa* Koch, 1944					X			
*Epitrichia* Gebler, 1859				X				
*Eschatostena* Keleinikova, 1977				X				
*Eulipus* Wollaston, 1864				X				
= *Mogadoria* Escalera, 1905								
*Eusyntelia* C.O. Waterhouse, 1881					X			
*Falsocatomulus* Pic, 1914				X				
= *Microhionthis* Blair, 1923								
*Freudeia* Kaszab, 1961						X		
*Freyitia* Koch, 1943						X		
*Girardius* L. Soldati, 2009				X				
*Gnathosia* Fischer von Waldheim, 1821				X				
= *Capnisa* Dejean, 1836								
*Gnophota* Erichson, 1843					X			
*Hegeter* Latreille, 1802				X	X			
SGHegeter Latreille, 1802				X	X			
SGHomalapipleurus Español, 1957				X				
SGPseudotalpophila Reitter, 1900				X				
*Hegeterocara* Reitter, 1900				X				
= *Fourtaus* Pic, 1921								
*Herlesa* Reitter, 1897				X				
*Hionthis* Miller, 1861				X				
*Homala* Eschscholtz, 1831					X			
= *Omala* Agassiz, 1846								
*Homalinota* Koch, 1950					X			
= *Homalopsis* Lesne, 1922								
*Homoeonota* Fairmaire, 1882				X	X			
= *Isonota* Fairmaire, 1887								
*Hyonthosoma* Reitter, 1900				X				
= *Hionthisoma* Gebien, 1937								
*Hyperops* Eschscholtz, 1831				X	X	X		
SGBelutschistanops Löbl, Bouchard, Merkl & Bousquet, 2020				X	X	X		
SGDebeauxiella Bouchard & Bousquet, **subgen. nov.**						X		
SGHyperops Eschscholtz, 1831				X	X	X		
= *Pachycera* Eschscholtz, 1831								
= *Tetromma* Dejean, 1834								
= *Oedenocera* Reiche, 1862								
SGHyperopsis Bouchard & Bousquet, **subgen. nov.**						X		
SGPachycerops Koch, 1943						X		
*Hypsosoma* Ménétriés, 1854				X				
*Imatismus* Dejean, 1834				X	X	X		
SGHimatismus Erichson, 1843					X			
SGImatismus Dejean, 1834				X	X	X		
= *Curimosphena* Gebien, 1920								
*Kokeniella* Reitter, 1906						X		
*Leptosphena* Semenov, 1891				X				
*Megagenius* Solier, 1835				X				
*Melanochrus* Wollaston, 1864				X				
= *Melasmocara* Reitter, 1900								
*Melaxumia* Reitter, 1895				X				
*Mesostena* Eschscholtz, 1831				X	X			
SGMesostena Eschscholtz, 1831				X	X			
= *Comphosida* Macquart, 1850								
SGMesostenopa Kraatz, 1865				X	X			
SGPlatystena Koch, 1940					X			
SGSaxistena Löbl & Merkl, 2020				X	X			
*Micipsa* P.H. Lucas, 1855				X	X			
SGCirsa P.H. Lucas, 1857				X				
= *Cyrta* Lacordaire, 1859								
SGMicipsa P.H. Lucas, 1855				X	X			
*Microdera* Eschscholtz, 1831				X				
SGAmicrodera Kaszab, 1966				X				
SGDordanea Reitter, 1887				X				
= *Adordanea* Reitter, 1897								
SGFalsomicrodera Kaszab, 1966				X				
SGIliodera Skopin, 1961				X				
SGMicrodera Eschscholtz, 1831				X				
= *Rhostax* Fischer von Waldheim, 1844								
SGTentyrodera Koch, 1943				X				
*Microderopsis* Haag-Rutenberg, 1876					X			
*Namaquaeon* Koch, 1950					X			
*Namibismus* Koch, 1952					X			
*Neognathosia* Kaszab, 1959				X				
*Nerinodon* Koch, 1952					X			
*Nothrocerus* Fairmaire, 1887					X			
*Orostegastopsis* Koch, 1962					X			
*Oterophloeus* Desbrochers des Loges, 1881				X				
= *Tynteria* Reitter, 1897								
*Oxycara* Solier, 1835				X	X	X		
SGOxycara Solier, 1835				X	X	X		
= *Emmenastus* Motschulsky, 1845								
= *Melancrus* Reiche & Saulcy, 1857								
= *Crypticoides* Fairmaire, 1898								
SGPleuroxycara Koch, 1959					X			
SGSymphoxycara Koch, 1943				X	X			
*Oxycarops* Reitter, 1900				X				
*Pachychila* Eschscholtz, 1831				X				
SGAnebacis Peyerimhoff, 1927				X				
SGHegeteromorpha Escalera, 1913				X				
SGNeocisba Reitter, 1900				X				
= *Neacisba* Peyerimhoff, 1927								
SGPachychila Eschscholtz, 1831				X				
= *Acisba* Dejean, 1834								
= *Lophoma* Solier, 1835								
= *Pachychile* Lacordaire, 1859								
SGPachychilina Reitter, 1900				X				
SGTentyromorpha Escalera, 1913				X				
= *Tentyriomorpha* Peyerimhoff, 1927								
*Paivaea* Wollaston, 1864				X				
= *Paivea* Scudder, 1882								
*Parabigopsis* Español, 1946				X				
*Paracirta* Schuster, 1930				X				
*Paulianesthes* Koch, 1962					X			
*Phaeotribon* Kraatz, 1865				X	X			
*Prochoma* Solier, 1835				X		X		
SGOxypistoma Löbl, Bouchard, Merkl & Bousquet, 2020				X		X		
SGProchoma Solier, 1835				X				
*Psammocryptus* Kraatz, 1865				X				
*Psammoica* Solier, 1835				X				
= *Psammoeca* Agassiz, 1846								
*Rhammatodes* Haag-Rutenberg, 1876					X			
SGAngoleantus Koch, 1952					X			
SGRhammatodes Haag-Rutenberg, 1876					X			
= *Euleantus* Haag-Rutenberg, 1876								
= *Tagenodes* Haag-Rutenberg, 1876								
= *Asbolius* Fairmaire, 1902								
*Rhomaleus* Chatanay, 1915				X	X			
*Rhytinota* Eschscholtz, 1831					X	X		
SGNemapus Solier, 1835						X		
= *Melarachnica* Kraatz, 1865								
SGProrhytinota Bouchard & Bousquet, **subgen. nov.**					X			
SGRhydimorpha Koch, 1943						X		
SGRhytinota Eschscholtz, 1831					X			
= *Rhytidonota* Agassiz, 1846								
= *Axumia* Reiche, 1848								
SGRhytistena Bouchard & Bousquet, **subgen. nov.**					X			
SGSphenariopsis Kraatz, 1865						X		
*Rozonia* Fairmaire, 1888					X			
SGPseudorozonia Bouchard & Bousquet, **subgen. nov.**					X			
SGRozonia Fairmaire, 1888					X			
*Scelosodis* Solier, 1835				X				
= *Cratopus* Eschscholtz, 1831								
= *Sceleoides* Agassiz, 1846								
= *Abiga* Guérin-Méneville, 1860								
= *Sceleodis* Gemminger, 1870								
*Schweinfurthia* Andres, 1922				X	X			
*Scythis* Kraatz, 1865				X				
= *Semenovonymus* Bogatchev, 1946								
= *Megascythis* Keleinikova, 1963								
*Scytosoma* Reitter, 1895				X				
= *Scythodonta* Reitter, 1897								
*Sinoecia* Chatanay, 1914				X				
*Sphenaria* Ménétriés, 1849				X				
*Stegastopsis* Kraatz, 1865				X				
= *Ohyonthis* Reitter, 1898								
*Stenosida* Solier, 1835						X	*X*	
= *Notioscythis* Fairmaire, 1883								
= *Aprosphaena* Reitter, 1916								
*Syachis* Bates, 1879				X				
= *Orocina* Reitter, 1897								
*Tamena* Reitter, 1900				X				
*Tentyria* Latreille, 1802				X				
= *Heliodromus* Brullé, 1832								
*Tentyrina* Reitter, 1900				X	X			
= *Tentyriina* Peyerimhoff, 1907								
*Tentyronota* Reitter, 1900				X				
*Thalpobia* Fairmaire, 1871				X				
= *Micipsina* Reitter, 1900								
*Thalpophilodes* Strand, 1942					X			
SGKaszabiella Koch, 1943					X			
SGRhytinopsis Bouchard & Bousquet, **subgen. nov.**					X			
SGThalpophilodes Strand, 1942					X			
= *Thalpophila* Solier, 1835								
*Thraustocolus* Kraatz, 1866				X	X			
SGLeptoderops Löbl, Bouchard, Merkl & Bousquet, 2020				X	X			
SGProthraustocola Kaszab, 1957				X				
SGThraustocolus Kraatz, 1866				X				
= *Calobamon* Kraatz, 1865								
= *Ibnsaudia* Koch, 1941								
*Trichosphaena* Reitter, 1916				X	X			
*Uyttenboogaartia* Koch, 1943				X				
**Thinobatini Lacordaire, 1859**								
*Cordibates* Kulzer, 1956			X					
*Thinobatis* Eschscholtz, 1831			X					
**Trilobocarini Lacordaire, 1859**								
*Derosalax* Gebien, 1926			X					
*Eremoecus* Lacordaire, 1859			X					
*Peltolobus* Lacordaire, 1859			X					
= *Megalophrys* G.R. Waterhouse, 1845								
= *Alhuena* Kulzer, 1956								
*Salax* Guérin-Méneville, 1834			X				*X*	
= *Pilioloba* Erichson, 1846								
*Trilobocara* Solier, 1851			X					
= *Edrotinus* Fairmaire, 1904								
= *Orthonychius* Gebien, 1926								
**Vacronini Gebien, 1910**								
*Alaephus* Horn, 1870		X						
= *Vacronus* Casey, 1907								
*Eupsophulus* Cockerell, 1906		X						
= *Eupsophus* Horn, 1870								
*Exangeltus* Blackburn, 1897							X	
*Lixionica* Blackburn, 1896							X	
**Zophosini Solier, 1834**								
*Zophosis* Latreille, 1802				X	X	X		
SGAnacardiosis Endrödy-Younga, 1986					X			
SGAnisosis Deyrolle, 1867					X			
SGCalosis Deyrolle, 1867					X			
SGCardiosis Deyrolle, 1867				X	X			
SGCaroliphosis Penrith, 1981					X			
SGCarpiella Koch, 1962					X			
SGCerosis Gebien, 1920					X			
SGCheirosis Deyrolle, 1867				X	X			
= *Chirosis* Gemminger, 1870								
SGDactylocalcar Gebien, 1938					X			
SGDignathosis Koch, 1958					X			
SGGahanosis Penrith, 1983					X			
SGGyrosis Gebien, 1920					X			
SGHeliophosis Koch, 1952					X			
SGHesseosis Koch, 1958					X			
SGHologenosis Deyrolle, 1867				X	X			
SGLatipleurosis Penrith, 1977					X			
SGMicrosis Koch, 1958					X			
SGMyrmecophosis Koch, 1958					X			
SGNamaphosis Penrith, 1981					X			
SGOccidentophosis Penrith, 1977					X			
SGOculosis Penrith, 1977				X	X			
SGOnychosis Deyrolle, 1867					X			
SGOphthalmosis Deyrolle, 1867				X	X			
SGPlanirostrosis Penrith, 1977					X			
SGPropemicrosis Penrith, 1981					X			
SGProtocalosis Penrith, 1977					X			
SGProtodactylus Koch, 1952					X			
= *Zophosodactylus* Koch, 1962								
SGSabulophosis Penrith, 1977					X			
SGScopulophosis Penrith, 1977					X			
SGSeptentriophosis Penrith, 1982				X	X	X		
SGSulcosis Penrith, 1977					X			
SGTarsosis Gebien, 1920					X			
SGZophosis Latreille, 1802					X			
= *Calcarosis* Penrith, 1977								
= *Predactylosis* Penrith, 1977								
**Zolodininae Watt, 1975**								
†*Praezolodinus* Bao, 2020								
*Tanylypa* Pascoe, 1869							X	
*Zolodinus* Blanchard, 1853							X	
**Lagriinae Latreille, 1825 (1820)**								
**Adeliini W. Kirby, 1828**								
*Adelium* W. Kirby, 1819							X	
= *Dystalica* Pascoe, 1869								
= *Rues* Casey, 1891								
= *Tropidopterus* Cazurro Ruiz, 1897								
*Adelodemus* Haag-Rutenberg, 1878							X	
= *Apostethus* Pascoe, 1882								
*Adelozotypus* Kaszab, 1982							X	
*Aoupinia* Matthews, 2003							X	
*Apasis* Pascoe, 1869							X	
*Apocryphodes* Matthews, 1998							X	
*Arcothymus* Pascoe, 1866							X	
*Bellendenum* Matthews, 1998							X	
*Blepegenes* Pascoe, 1868							X	
= *Ceradelium* Preudhomme de Borre, 1868								
*Bluops* Carter, 1914							X	
*Bolusculus* Matthews, 1998							X	
*Brycopia* Pascoe, 1869							X	
= *Dinoria* Pascoe, 1869								
*Cardiothorax* Motschulsky, 1860							X	
= *Thoracophorus* Hope, 1841								
= *Atryphodes* Pascoe, 1866								
= *Otrintus* Pascoe, 1866								
*Coripera* Pascoe, 1866							X	
*Cymbeba* Pascoe, 1866							X	
*Daedrosis* Bates, 1868							X	
= *Macroperas* Carter, 1914								
*Diaspirus* Matthews, 1998							X	
*Dicyrtodes* Matthews, 1998							X	
*Diemenoma* Matthews, 1998							X	
*Dorrigonum* Matthews, 1998							X	
*Epomidus* Matthews, 1998							X	
*Exadelium* Watt, 1992							X	
*Gondvanadelium* Kaszab, 1981			X					
*Isopteron* Hope, 1841							X	
= *Cestrinus* Erichson, 1842								
= *Isopterum* Agassiz, 1846								
= *Apatelus* Mulsant & Rey, 1859								
= *Prionotus* Mulsant & Rey, 1859								
= *Achora* Pascoe, 1869								
= *Priothorax* Gebien, 1910								
*Kaszabadelium* Watt, 1992							X	
*Leptogastrus* W.J. MacLeay, 1872							X	
*Licinoma* Pascoe, 1869			X				X	
*Mesopatrum* Broun, 1893							X	
*Montaguea* Kaszab, 1982							X	
*Monteithium* Matthews, 1998							X	
*Neoadelium* Carter, 1908							X	
= *Pseudadelium* Kaszab, 1982								
*Nolicima* Matthews, 1998							X	
*Nototrintus* Carter, 1924							X	
*Ozotypoides* Kaszab, 1982							X	
*Penadelium* Matthews, 1998			X					
*Periatrum* Sharp, 1886							X	
*Pheloneis* Pascoe, 1866							X	
= *Amarosoma* Redtenbacher, 1868								
*Pseudobyrsax* Kaszab, 1982							X	
*Pseudocilibe* Kaszab, 1982							X	
*Pseudopatrum* Sharp, 1886							X	
= *Mitua* Hope, 1848								
*Seirotrana* Pascoe, 1866							X	
*Stenadelium* Watt, 1992							X	
*Valdivium* Matthews, 1998			X					
*Wattadelium* Emberson, 2000							X	
= *Edalus* Broun, 1893								
*Yarranum* Matthews, 1998							X	
*Zeadelium* Watt, 1992							X	
**Belopini Reitter, 1917**								
*Adelonia* Laporte, 1840		X	X					
SGAdelonia Laporte, 1840		X	X					
= *Merotemnus* Horn, 1870								
= *Rhacius* Champion, 1885								
= *Ostorius* Fairmaire, 1889								
SGLatorhascius Pic, 1925			X					
*Centorus* Mulsant, 1854				X	X			
SGBelopomerus Reitter, 1920				X				
SGBelopus Gebien, 1911				X	X			
= *Calcar* Dejean, 1821								
= *Hemeralopius* Gistel, 1848								
SGCentorus Mulsant, 1854				X				
SGNanocalcar Skopin, 1974				X				
*Doyenia* Matthews & Lawrence, 2005							X	
*Euclarkia* Lea, 1919							X	
*Eulea* Carter, 1937							X	
*Exeniotis* Pascoe, 1871			X					
*Kershawia* Lea, 1905							X	
*Rhypasma* Pascoe, 1862			X					
= *Derosimus* Fairmaire, 1904								
*Thoseus* Pic, 1925						X		
†*Yantaroxenos* Nabozhenko, Kirejtshuk & Merkl, 2016								
**Chaerodini Doyen, Matthews & Lawrence, 1990**								
*Chaerodes* White, 1846							X	
= *Choerodes* Gemminger, 1870								
*Sphargeris* Pascoe, 1860							X	
**Cossyphini Latreille, 1802**								
*Cossyphus* G.-A. Olivier, 1791				X	X	X	*X*	
SGAcontodactylus Desbrochers des Loges, 1894				X	X	X	*X*	
= *Paracossyphus* Viñolas & Cartagena, 2005								
SGCossyphus G.-A. Olivier, 1791				X	X	X		
*Endustomus* Brême, 1842					X			
= *Endostomus* Gemminger, 1870								
= *Endostostomus* Jakobson, 1914								
**Eschatoporiini Blaisdell, 1906**								
*Eschatoporis* Blaisdell, 1906		X						
**Goniaderini Lacordaire, 1859**								
*Acropachia* Mäklin, 1875			X					
*Anaedus* Blanchard, 1842		X	X	X	X	X		
= *Aspisoma* Duponchel & Chevrolat, 1841								
= *Anaedes* Agassiz, 1846								
= *Aspidosoma* Agassiz, 1846								
*Gamaxus* Bates, 1868			X					
*Goniadera* Perty, 1832			X					
SGAemymone Bates, 1868			X					
SGGoniadera Perty, 1832			X					
= *Goniodera* Agassiz, 1846								
SGOpatresthes Gebien, 1928			X					
*Lyprochelyda* Fairmaire, 1899					X			
= *Basanaedus* Pic, 1917								
*Microanaedus* Pic, 1923					X			
*Microgoniadera* Pic, 1917			X					
*Microlyprops* Kaszab, 1939						X		
*Myrmecopeltoides* Kaszab, 1973			X					
*Paratenetus* Spinola, 1845		X	X					
= *Lagriola* Kirsch, 1874								
= *Storthephora* Mäklin, 1875								
*Pengalenganus* Pic, 1917						X	X	
*Phobelius* Blanchard, 1842			X					
*Phymatestes* Pascoe, 1866			X					
= *Phymatodes* Dejean, 1834								
*Prateus* LeConte, 1862		X	X					
*Pseudanaedus* Gebien, 1921					X			
*Pseudolyprops* Fairmaire, 1882							X	
= *Trichulodes* Carter, 1914								
*Spinadaenus* Pic, 1921						X		
*Tithassa* Pascoe, 1860			X					
*Xanthicles* Champion, 1886			X					
†**Gonialaenini Nabozhenko, Bukejs & Telnov, 2019**								
†*Gonialaena* Nabozhenko, Bukejs & Telnov, 2019								
**Laenini Seidlitz, 1895**								
*Afrolaena* Endrödy-Younga & Schawaller, 2002					X			
*Borneolaena* Schawaller, 1998						X		
*Chaetyllus* Pascoe, 1860			X					
= *Polytropus* Kirsch, 1866								
= *Rhicnodus* Fairmaire, 1892								
*Enigmatica* Ferrer, 2005					X			
*Grabulax* Kanda, 2016			X					
*Hovadelium* Ardoin, 1962					X			
*Hypolaenopsis* Masumoto, 2001				X				
*Laena* Dejean, 1821				X		X		
= *Catolaena* Reitter, 1900								
= *Psilolaena* Heller, 1923								
= *Ebertius* Jedlička, 1965								
*Lucidolaena* Endrödy-Younga & Schawaller, 2002					X			
*Mimolaena* Ardoin, 1962					X			
*Nepalolaena* Schawaller, 2001						X		
*Prolaena* Kaszab, 1980						X		
*Rhacolaena* Kaszab, 1979						X		
**Lagriini Latreille, 1825 (1820)**								
**Lagriina Latreille, 1825 (1820)**								
*Acerogria* Borchmann, 1936							X	
= *Birolagria* Pic, 1956								
*Acritolagria* Borchmann, 1916					X			
*Acutogria* Merkl, 1988							X	
*Adosogria* Borchmann, 1936					X			
*Adynata* Fåhraeus, 1870				X	X			
= *Microlagria* Seidlitz, 1898								
*Alagria* Borchmann, 1916					X			
= *Lagriostira* Kolbe, 1902								
*Allogria* Borchmann, 1916					X			
*Anotoma* Borchmann, 1936					X			
*Aulonogria* Borchmann, 1929						X		
*Bequaertiella* Pic, 1914					X			
*Bothrichara* Borchmann, 1916							X	
*Bothrionota* Borchmann, 1936						X		
*Bothynogria* Borchmann, 1916				X		X		
*Calogria* Borchmann, 1916							X	
*Ceratoma* Borchmann, 1916*								
*Cerogria* Borchmann, 1911				X	X	X	X	
SGCerogria Borchmann, 1911				X	X	X	X	
= *Cerogriodes* Borchmann, 1941								
= *Aeschrocera* Chen & Chou, 1996								
SGDrepanomela Borchmann, 1936							X	
SGParogria Borchmann, 1936					X			
*Cerostira* Borchmann, 1942					X			
= *Allocera* Borchmann, 1916								
*Chilenolagria* Pic, 1936			X					
*Chrysolagria* Seidlitz, 1898				X	X			
*Costiferolagria* Pic, 1915						X		
= *Auristira* Borchmann, 1916								
*Ctenogria* Borchmann, 1916						X		
*Derolagria* Borchmann, 1916					X			
*Ecnocera* Borchmann, 1936					X			
*Ecnolagria* Borchmann, 1916							X	
SGEcnolagria Borchmann, 1916							X	
SGOnocera Borchmann, 1936							X	
*Emydodes* Pascoe, 1860			X					
*Falsolagria* Pic, 1927			X					
*Flabellolagria* Pic, 1927					X			
*Gronophora* Borchmann, 1916							X	
*Helogria* Borchmann, 1916						X		
*Kaindilagria* Merkl, 1988							X	
*Lagria* Fabricius, 1775			*X*	X	X	X	X	
SGAmmocera Borchmann, 1941				X		X		
SGApteronympha Seidlitz, 1898				X	X			
SGGrandelagria Pic, 1940					X			
SGLagria Fabricius, 1775			*X*	X	X	X	X	
= *Lachna* Billberg, 1820								
SGLagriella Borchmann, 1916					X	X		
*Lagriopsis* Borchmann, 1916							X	
*Lopholagria* Borchmann, 1916					X			
SGLopholagria Borchmann, 1916					X			
SGMokalagria Pic, 1953					X			
*Lorona* Borchmann, 1936						X		
*Mallogria* Borchmann, 1936					X			
*Metriolagria* Merkl, 1987							X	
*Mimolagria* Pic, 1927			X					
*Minasius* Pic, 1932			X					
*Neogria* Borchmann, 1911						X		
= *Lagriomima* Pic, 1950 **syn. nov.**								
*Nothogria* Borchmann, 1916							X	
*Odontogria* Borchmann, 1936						X		
*Oreogria* Merkl, 1988							X	
*Oroptera* Borchmann, 1916							X	
*Phaedogria* Borchmann, 1936						X		
*Physogria* Borchmann, 1916					X			
*Physolagria* Fairmaire, 1891					X			
*Porrolagria* Kolbe, 1883					X			
= *Lagrimina* Fairmaire, 1894								
*Pseudolagria* Champion, 1917			X					
*Rhaibogria* Borchmann, 1936					X			
*Ruandania* Pic, 1955					X			
*Schevodera* Borchmann, 1936						X		
*Schevogria* Borchmann, 1936					X			
*Sphinctoderus* Fairmaire, 1903						X		
*Stenolagria* Merkl, 1987							X	
*Sulcolagria* Pic, 1955					X			
*Tomogria* Merkl, 1988							X	
*Xenocerogria* Merkl, 2007				X		X		
= *Xenocera* Borchmann, 1936								
*Xenogena* Borchmann, 1936					X			
*Xenolagria* Merkl, 1987							X	
**Statirina Blanchard, 1845**								
*Anisostira* Borchmann, 1915				X		X		
*Arthromacra* W. Kirby, 1837		X		X		X		
= *Macrarthra* Agassiz, 1846								
*Arunogria* Merkl, 1991						X		
*Astatira* Borchmann, 1921			X					
*Barsenis* Pascoe, 1887			X					
SGBarsenis Pascoe, 1887			X					
SGMicrodisema Pic, 1917			X					
SGModicodisema Pic, 1917			X					
SGNemostiromorpha Pic, 1917			X					
= *Adisema* Borchmann, 1936								
*Borchmannia* Pic, 1912						X		
SGBorchmannia Pic, 1912						X		
SGPseuduroplatopsis Pic, 1913						X		
*Bothriostira* Borchmann, 1936					X			
*Casnonidea* Fairmaire, 1882					X	X	X	
SGCasnonidea Fairmaire, 1882					X	X	X	
= *Synatractus* W.J. MacLeay, 1887								
= *Falsocasnonidea* Pic, 1934								
SGPilosocasnonidea Pic, 1934						X		
*Chlorophila* Semenov, 1891				X		X		
*Colparthrum* Kirsch, 1866			X					
SGColparthrum Kirsch, 1866			X					
SGPseudocolparthrum Borchmann, 1916			X					
*Costatosora* Pic, 1934					X			
*Cylindrosora* Borchmann, 1936						X		
*Cylindrostira* Borchmann, 1936						X		
*Davaona* Borchmann, 1930						X		
*Derostira* Fairmaire, 1897					X			
*Diorhychina* Borchmann, 1936					X			
*Disema* Mäklin, 1875			X					
*Disemorpha* Pic, 1917			X					
*Donaciolagria* Pic, 1914				X		X		
*Dysodera* Borchmann, 1936					X			
*Dysopinus* Borchmann, 1936						X		
*Eccoptostira* Borchmann, 1936					X			
*Entypodera* Gerstaecker, 1871					X			
= *Loubacantus* Bonadona, 1959								
*Epicydes* Champion, 1889			X					
SGCybostira Borchmann, 1936			X					
SGEpicydes Champion, 1889			X					
*Exostira* Borchmann, 1925						X		
*Falsonemostira* Pic, 1917						X		
*Gebienia* Borchmann, 1921			X					
*Hosohamudama* Masumoto, 1988						X		
*Hypostatira* Fairmaire, 1889			X					
*Hysterarthron* J. Thomson, 1864						X		
*Impressosora* Pic, 1952					X			
SGImpressosora Pic, 1952					X			
SGNeoeutrapela Bousquet & Bouchard, 2013					X			
= *Eutrapela* Dejean, 1834								
*Isocera* Borchmann, 1909			X					
= *Isotoma* Blanchard, 1842								
*Lagriodema* Borchmann, 1930						X		
*Lagriogonia* Fairmaire, 1891				X				
*Lagriomima* Pic, 1934						X	X	
*Lagriostira* Fairmaire, 1883					X			
*Leptinostethus* Borchmann, 1936					X			
*Leptosora* Borchmann, 1936					X			
*Lobophilomorphus* Pic, 1911					X			
*Lophophyllus* Fairmaire, 1887					X			
*Macrocasnonidea* Pic, 1934						X		
*Macrolagria* Lewis, 1895				X				
*Malaiseum* Borchmann, 1941						X		
*Meniscophorus* Champion, 1889			X					
*Merklia* Chen, 1997						X		
*Meropria* Borchmann, 1921			X					
*Mimoborchmania* Pic, 1934						X		
*Mimuroplatopsis* Borchmann, 1936					X			
*Natalostira* Pic, 1913					X			
*Nevermanniella* Borchmann, 1936			X					
*Ocularisora* Pic, 1934					X			
*Odontocerostira* Merkl, 2007				X		X		
= *Odontocera* Chen & Yuan, 1996								
*Othryades* Champion, 1889			X					
*Pachystira* Chen, 1997						X		
*Piciella* Borchmann, 1936			X					
*Pseudeutrapela* Pic, 1952					X			
*Pseudocasnonidea* Borchmann, 1936						X		
*Pseudostira* Fairmaire, 1903					X			
*Rhagostira* Borchmann, 1936					X			
*Rhaibodera* Borchmann, 1921			X					
*Rhosaces* Champion, 1889			X					
*Robustosora* Pic, 1954					X			
*Rouyerus* Pic, 1911						X		
SGBorneostira Pic, 1912						X		
SGRouyerus Pic, 1911						X		
*Sipolisia* Fairmaire, 1889			X					
*Sora* Walker, 1859				X	X	X	X	
SGHirsutosora Pic, 1934						X		
SGNemostiropsis Borchmann, 1936						X		
SGSora Walker, 1859				X	X	X	X	
= *Nemostira* Fairmaire, 1869								
*Sphragidophorus* Champion, 1889			X					
*Splichalia* Reitter, 1913				X				
*Staius* Fairmaire, 1896					X			
SGDerostirostaius Borchmann, 1936					X			
SGStaius Fairmaire, 1896					X			
*Statira* Lepeletier & Audinet-Serville, 1828		X	X					
SGFoveostatira Pic, 1918			X					
SGPleurostira Borchmann, 1921			X					
SGSpinostatira Pic, 1918			X					
= *Hoplostira* Borchmann, 1921								
SGStatira Lepeletier & Audinet-Serville, 1828		X	X					
SGXenostira Borchmann, 1921			X					
*Statiropsis* Borchmann, 1912			X					
*Strongylagria* Pic, 1915				X				
*Taiwanolagria* Masumoto, 1988				X		X		
*Thoracostira* Borchmann, 1936			X					
*Uroplatopsis* Champion, 1889			X					
*Xanthalia* Fairmaire, 1894				X	X	X		
= *Xanthia* Fairmaire, 1893								
= *Heterogria* Fairmaire, 1896								
= *Lagriocera* Fairmaire, 1896								
= *Wallardilagria* Pic, 1910								
= *Pachylagria* Borchmann, 1912								
= *Lagriodes* Borchmann, 1930								
= *Diversogria* Pic, 1954								
*Xenostethus* Bates, 1868					X			
= *Coracostira* Fairmaire, 1899								
**Lupropini Lesne, 1926**								
*Antennoluprops* Schawaller, 2007					X			
*Ardoiniellus* Schawaller, 2013					X			
*Bolitrium* Gebien, 1914						X		
*Capeluprops* Schawaller, 2011					X			
*Coxelinus* Fairmaire, 1869					X			
*Curtolyprops* Pic, 1917					X			
*Dichastops* Gerstaecker, 1871					X			
*Enicmosoma* Gebien, 1922					X			
= *Enicmonota* Ardoin, 1958								
*Falsotithassa* Pic, 1934						X		
= *Derispiolina* Kaszab, 1979								
*Indenicmosoma* Ardoin, 1964				X		X		
*Iscanus* Fauvel, 1904							X	X
= *Araucaricola* Lea, 1929								
*Kuschelus* Kaszab, 1982							X	
*Lorelus* Sharp, 1876			X				X	X
= *Lorelopsis* Champion, 1896								
*Luprops* Hope, 1833				X	X	X	X	
= *Oligorus* Dejean, 1834								
= *Syggona* Fåhraeus, 1870								
= *Etazeta* Fairmaire, 1889								
= *Syngona* Rye, 1873								
*Mesotretis* Bates, 1872							X	
*Microcalcar* Pic, 1925					X			
*Micropedinus* Lewis, 1894				X		X	X	
= *Notoprataeus* Carter, 1924								
*Mimocellus* Wasmann, 1904					X			
*Paralorelopsis* Marcuzzi, 1994			X					
*Sphingocorse* Gebien, 1921					X	X		
*Spinolagriella* Pic, 1955					X			
= *Spinorhacus* Kaszab, 1969								
*Spinolyprops* Pic, 1917				X	X	X		
*Terametus* Motschulsky, 1869					X			
**Pycnocerini Lacordaire, 1859**								
*Aediatorix* Bates, 1868						X		
= *Sipirocus* Fairmaire, 1896								
*Amorphochirus* Gebien, 1904					X			
*Calostegia* Westwood, 1843					X			
= *Calostega* Gemminger, 1870								
= *Apristopus* Kolbe, 1903								
*Catamerus* Fairmaire, 1887					X			
*Chirocharis* Kolbe, 1903					X			
*Chiroscelis* Lamarck, 1804					X			
*Gabonisca* Fairmaire, 1894					X			
= *Gabonia* Fairmaire, 1894								
*Hemipristula* Bouchard & Bousquet, **nom. nov.**					X			
= *Hemipristis* Kolbe, 1903								
*Malayoscelis* Schawaller, 2003						X		
*Metallonotus* Gray, 1832					X			
= *Aspidosternum* Mäklin, 1867								
*Passalocharis* Koch, 1954					X			
*Pezodontus* Dejean, 1834					X			
= *Odontopus* Silbermann, 1833								
= *Odontopezus* Alluaud, 1889								
*Pheugonius* Fairmaire, 1899						X		
*Prioproctus* Kolbe, 1903					X			
*Prioscelides* Kolbe, 1889					X			
*Prioscelis* Hope, 1841					X			
= *Iphius* Dejean, 1834								
= *Bovius* Gistel, 1848								
*Pristophilus* Kolbe, 1903					X			
*Pycnocerus* Westwood, 1844					X			
SGDinoscelis Gerstaecker, 1854					X			
SGPycnocerus Westwood, 1844					X			
= *Pachylocerus* Hope, 1841								
*Stratodemus* Gebien, 1921					X			
**Lagriinae incertae sedis**								
*Asiopus* Sharp, 1892			X					
*Pseudesarcus* Champion, 1913			X					
**Nilioninae Oken, 1843**								
*Nilio* Latreille, 1802			X					
SGLinio Bouchard & Bousquet, **subgen. nov.**			X					
SGMicronilio Pic, 1936			X					
SGNilio Latreille, 1802			X					
**Phrenapatinae Solier, 1834**								
**Archaeoglenini Watt, 1975**								
*Archaeoglenes* Broun, 1893			X	X	X	X	X	X
*Sepilokus* Iwan & Raś, 2020						X		
**Penetini Lacordaire, 1859**								
*Afrotagalus* Gebien, 1942					X			
*Archeophthora* Kaszab, 1978			X				X	
*Clamoris* Gozis, 1886		X	X	X		X		
= *Phtora* Mulsant, 1854								
= *Phthora* Champion, 1893								
*Cleolaus* Champion, 1886			X					
*Daochus* Champion, 1886			X					
*Dioedus* LeConte, 1862		X	X	X	X	X	X	X
= *Arrhabaeus* Champion, 1886								
= *Brachycilibe* Carter, 1911								
= *Tagalus* Gebien, 1914								
*Endroeditagalus* Schawaller & Bouchard, 2019					X			
*Exechophthalmus* Ardoin, 1974					X			
*Falsotagalus* Kaszab, 1977							X	
*Leleupium* Kaszab, 1956					X			
*Madagassa* Koch, 1950					X			
= *Pycna* Fairmaire, 1894								
*Molion* Champion, 1886			X					
*Nanotagalus* Gebien, 1942					X			
*Neotagalus* Kaszab, 1955								X
*Peneta* Lacordaire, 1859			X					
*Pseudophthora* Kaszab, 1970						X	X	X
SGFalsophthora Kaszab, 1977								X
SGPseudophthora Kaszab, 1970						X	X	
*Pycnochilus* C.O. Waterhouse, 1879					X			
*Scolytocaulus* Fairmaire, 1896					X	X	X	
= *Platycilibe* Carter, 1911								
= *Picnotagalus* Kaszab, 1939								
*Tagalinus* Kaszab, 1977							X	
*Tagalopsis* Kaszab, 1955								X
*Taiwanotagalus* Masumoto, 1982						X		
*Telchis* Champion, 1886			X					
*Zypoetes* Champion, 1893			X			X	X	
**Phrenapatini Solier, 1834**								
*Delognatha* Lacordaire, 1859			X					
*Phrenapates* Gray, 1831			X					
**Phrenapatinae *incertae sedis***								
*Aphtora* Bates, 1872							X	
**Kuhitangiinae G.S. Medvedev, 1962**								
**Foranotini Nabozhenko & Sadeghi, 2017**								
*Foranotum* Nabozhenko & Sadeghi, 2017				X				
**Kuhitangiini G.S. Medvedev, 1962**								
*Kuhitangia* G.S. Medvedev, 1962				X				
**Blaptinae Leach, 1815**								
**Amphidorini LeConte, 1862**								
*Eleodes* Eschscholtz, 1829		X	X					
SGAmphidora Eschscholtz, 1829		X						
SGArdeleodes Blaisdell, 1937		X						
SGBlapylis Horn, 1870		X						
= *Eleodopsis* Blaisdell, 1939								
SGCaverneleodes Triplehorn, 1975		X						
SGChaseleodes Thomas, 2015			X					
SGCratidus LeConte, 1862		X						
SGDiscogenia LeConte, 1866		X						
SGEleodes Eschscholtz, 1829		X	X					
= *Elaeodes* Gemminger, 1870								
SGHeteropromus Blaisdell, 1909		X						
SGLitheleodes Blaisdell, 1909		X						
SGMelaneleodes Blaisdell, 1909		X	X					
SGMetablapylis Blaisdell, 1909		X						
SGOmegeleodes Triplehorn & Thomas, 2012		X	X					
SGPromus LeConte, 1862		X	X					
SGPseudeleodes Blaisdell, 1909		X						
= *Trichoderulus* Blaisdell, 1923								
SGSteneleodes Blaisdell, 1909		X	X					
= *Xysta* Eschscholtz, 1829								
= *Holeleodes* Blaisdell, 1937								
SGTricheleodes Blaisdell, 1909		X						
*Eleodimorpha* Blaisdell, 1909		X						
*Embaphion* Say, 1824		X						
*Lariversius* Blaisdell, 1947		X						
*Neobaphion* Blaisdell, 1925		X						
*Nycterinus* Eschscholtz, 1829			X					
*Trogloderus* LeConte, 1879		X						
**Blaptini Leach, 1815**								
**Blaptina Leach, 1815**								
*Ablapsis* Reitter, 1887				X				
*Blaps* Fabricius, 1775		*X*		X	X	X	*X*	
SGArenoblaps G.S. Medvedev, 1999				X				
SGBlaps Fabricius, 1775		*X*		X	X	X	*X*	
= *Leptomorpha* Faldermann, 1835								
= *Agroblaps* Motschulsky, 1860								
= *Blapimorpha* Motschulsky, 1860								
= *Blapisa* Motschulsky, 1860								
= *Lithoblaps* Motschulsky, 1860								
= *Platyblaps* Motschulsky, 1860								
= *Rhizoblaps* Motschulsky, 1860								
= *Uroblaps* Motschulsky, 1860								
= *Leptocolena* Allard, 1880								
= *Acanthoblaps* Reitter, 1889								
= *Blapidurus* Fairmaire, 1891								
= *Blapidium* Bauer, 1921								
= *Hypoblaps* Kolbe, 1928								
= *Mesooblaps* Kolbe, 1928								
= *Notoblaps* Kolbe, 1928								
= *Opisthoblaps* Kolbe, 1928								
= *Nanoblaps* Semenov & Bogatchev, 1936								
SGDineria Motschulsky, 1860				X				
= *Laraliprosodes* Bogatchev, 1947								
SGGenoblaps Bauer, 1921				X		X		
= *Prosoblapsia* Skopin & Kaszab, 1978								
*Caraboblaps* Bauer, 1921*								
*Coelocnemodes* Bates, 1879						X		
= *Neoblaps* Ren & Li, 2001								
*Dila* Fischer von Waldheim, 1844				X				
= *Caenoblaps* König, 1906								
*Dilablaps* Bogatchev, 1976				X				
*Holoblaps* Bauer, 1921*								
*Hoplitoblaps* Fairmaire, 1889						X		
*Medvedevia* Chigray, 2019				X				
*Medvedevoblaps* Bouchard & Bousquet, **nom. nov.**				X				
= *Protoblaps* G.S. Medvedev, 1998								
*Nalepa* Reitter, 1887				X				
*Periblaps* Bauer, 1921*								
*Protoblaps* Bauer, 1921*								
*Thaioblaps* Masumoto, 1989						X		
*Thaumatoblaps* Kaszab & G.S. Medvedev, 1984				X				
**Gnaptorina G.S. Medvedev, 2001**								
*Gnaptor* Brullé, 1831				X				
SGGnaptor Brullé, 1831				X				
SGPlesiognaptor Chigray, Nabozhenko & Keskin, 2015				X				
**Gnaptorinina G.S. Medvedev, 2001**								
*Agnaptoria* Reitter, 1887				X				
*Asidoblaps* Fairmaire, 1886				X		X		
*Belousovia* G.S. Medvedev, 2007				X				
*Blaptogonia* G.S. Medvedev, 1998				X		X		
*Colasia* Koch, 1965				X				
*Gnaptorina* Reitter, 1887				X		X		
SGAustroptorina Bai, Li & Ren, 2020						X		
SGBoreoptorina G.S. Medvedev, 2009				X				
SGGnaptorina Reitter, 1887				X				
SGHesperoptorina G.S. Medvedev, 2009				X		X		
*Itagonia* Reitter, 1887				X				
*Montagona* G.S. Medvedev, 1998				X		X		
*Nepalindia* G.S. Medvedev, 1998						X		
*Pseudognaptorina* Kaszab, 1977				X		X		
*Sintagona* G.S. Medvedev, 1998				X				
*Tagonoides* Fairmaire, 1886				X				
*Viettagona* G.S. Medvedev & Merkl, 2003						X		
**Prosodina Skopin, 1960**								
*Prosodes* Eschscholtz, 1829				X		X		
SGDilopersina Reitter, 1909				X				
SGDiprosodes Reitter, 1909				X				
SGEuryprosodes Reitter, 1909				X				
SGFerganoprosodes G.S. Medvedev, 1997				X				
SGGebleria Motschulsky, 1846				X				
= *Aulonoscelis* Reitter, 1896								
SGHypoprosodes Reitter, 1909				X				
SGIndoprosodes G.S. Medvedev, 2003						X		
SGIranosodes G.S. Medvedev, 1996				X				
SGLyprosodes Reitter, 1909				X				
SGMegaprosodes Reitter, 1909				X				
= *Altiprosodes* G.S. Medvedev, 1997								
SGMeropersina Reitter, 1909				X				
SGMesoprosodes G.S. Medvedev, 1995				X				
SGMontiprosodes G.S. Medvedev, 2001				X				
SGOliprosodes Reitter, 1909				X				
SGPeltarium Fischer von Waldheim, 1844				X				
= *Blaposodes* Skopin, 1960								
SGPlanoprosodes G.S. Medvedev, 2005				X				
SGProsodella Reitter, 1909				X				
= *Paraprosodes* Reitter, 1909								
SGProsodes Eschscholtz, 1829				X				
= *Nyctipates* Gebler, 1841								
= *Eupomeca* Solier, 1848								
= *Blaptoprosodes* Reitter, 1909								
= *Lioprosodes* Reitter, 1909								
= *Platyprosodes* Reitter, 1909								
= *Prosodila* Reitter, 1909								
= *Pseudoprosodes* Reitter, 1909								
SGProsodestes Reitter, 1909				X				
SGProsodinia Reitter, 1909				X				
SGProsodopria Reitter, 1909				X				
= *Nyctipates* Solier, 1848								
SGProsodoscelis Reitter, 1909				X				
SGProsodura Reitter, 1909				X				
SGTangiprosodes G.S. Medvedev, 2005				X				
SGUroprosodes Reitter, 1909				X				
*Tagona* Fischer von Waldheim, 1820				X				
**Remipedellina Semenov, 1907**								
*Remipedella* Semenov, 1907				X				
**Dendarini Mulsant & Rey, 1854**								
**Dendarina Mulsant & Rey, 1854**								
*Bioplanes* Mulsant, 1854				X				
*Dendarophylan* Español, 1945				X				
*Dendarus* Dejean, 1821				X				
SGDendaroscelis Reitter, 1904				X				
SGDendarus Dejean, 1821				X				
= *Pandarus* Dejean, 1834								
= *Microdendarus* Escalera, 1944								
= *Reitterellus* Escalera, 1944								
SGPandarinus Mulsant & Rey, 1854				X				
SGParoderus Mulsant & Rey, 1854				X				
= *Dichromma* Mulsant & Rey, 1855								
SGRhizalemus Reitter, 1904				X				
SGRizalus Mulsant & Rey, 1854				X				
= *Rhizalus* Gebien, 1938								
*Heliopates* Dejean, 1834				X				
SGHeliocrates Reitter, 1904				X				
SGHeliopates Dejean, 1834				X				
= *Heliocaes* Bedel, 1906								
= *Heliopathes* Gebien, 1938								
*Litoboriolus* Español, 1945				X				
*Litororus* Reitter, 1904				X				
*Meglyphus* Motschulsky, 1872					X			
*Microphylacinus* Iwan, Kamiński & Aalbu, 2011					X			
*Micrositus* Mulsant & Rey, 1854				X				
*Neoisocerus* Bouchard, Lawrence, Davies & Newton, 2005				X				
= *Isocerus* Dejean, 1821								
*Phylacinus* Fairmaire, 1896					X			
*Phylan* Sturm, 1826				X				
SGEumicrositus Viñolas, 1990				X				
SGMeladeras Mulsant & Rey, 1854				X				
SGMeladocrates Reitter, 1904				X				
SGPhylan Sturm, 1826				X				
= *Heliophilus* Dejean, 1821								
= *Phylax* Brullé, 1832								
= *Olocrates* Mulsant, 1854								
SGPlatyolus Mulsant & Rey, 1854				X				
*Phylanmania* Ferrer, 2013				X				
*Pythiopus* Koch, 1953					X			
**Melambiina Mulsant & Rey, 1854**								
*Allophylax* Bedel, 1906				X				
SGAllophylax Bedel, 1906				X				
= *Neophylax* Bedel, 1906								
= *Melambiophylax* Schuster, 1922								
SGLitoboromimus Koch, 1948				X				
SGPhylaximon Koch, 1948				X				
*Bermejoina* Español, 1944				X				
*Gridelliopus* Koch, 1956					X			
*Guildia* Antoine, 1957				X				
*Hadroderus* Koch, 1956					X			
*Haemodus* Gebien, 1943					X			
= *Haemus* Péringuey, 1904								
*Hanstroemium* Koch, 1953					X			
*Hoplarion* Mulsant & Rey, 1854				X				
SGAtlasion Koch, 1948				X				
= *Megatlasion* Koch, 1948								
SGGlyptariobius Koch, 1948				X				
SGHoplariobius Reitter, 1904				X				
SGHoplarion Mulsant & Rey, 1854				X				
SGMentariobius Koch, 1948				X				
SGSaharoplarion Koch, 1948				X				
*Lasioderus* Mulsant & Rey, 1854					X			
*Litoborus* Mulsant & Rey, 1854				X				
SGLitoborus Mulsant & Rey, 1854				X				
SGParalitoborus Antoine, 1931				X				
*Melambius* Mulsant & Rey, 1854				X				
SGHadromelambius Koch, 1948				X				
SGHoplambius Reitter, 1904				X				
SGMelambatlasus Koch, 1948				X				
SGMelambius Mulsant & Rey, 1854				X				
*Melansis* Wollaston, 1864				X				
*Melasmana* Strand, 1935				X				
SGHeliomelasma Koch, 1948				X				
SGMelasmana Strand, 1935				X				
= *Melasma* Wollaston, 1864								
*Minorus* Mulsant & Rey, 1854					X			
*Orarabion* Leo & Liberto, 2011					X			
*Oreomelasma* Español, 1975				X				
*Otinia* Antoine, 1942				X				
SGAntoineius Koch, 1948				X				
SGOrophylaxus Koch, 1948				X				
SGOtinia Antoine, 1942				X				
*Peyerimhoffius* Koch, 1948				X				
*Psammoardoinellus* Leo, 1981				X				
*Pseudemmallus* Koch, 1956					X			
*Silvestriellum* Koch, 1956					X			
*Selenepistoma* Dejean, 1834					X			
SGSelenepistoma Dejean, 1834					X			
= *Euzadenos* Koch, 1956								
SGSerridenos Koch, 1956					X			
SGZadenos Laporte, 1840					X			
*Tragardhus* Koch, 1956					X			
SGMitragardhus Koch, 1956					X			
SGTragardhus Koch, 1956					X			
*Zoutpansbergia* Koch, 1956					X			
**Opatrini Brullé, 1832**								
**Ammobiina Desbrochers des Loges, 1902**								
*Adavius* Mulsant & Rey, 1859				X		X		
= *Udebra* Reitter, 1896								
*Ammidium* Erichson, 1843					X			
= *Eremonomus* Wollaston, 1861								
= *Ecripsis* Pascoe, 1866								
*Ammobius* Guérin-Méneville, 1844				X		X		
= *Ammophtorus* Lacordaire, 1859								
*Amphithrixoides* Bouchard & Löbl, 2008				X				
= *Amphithrix* Español, 1953								
*Arabammobius* Grimm & Lillig, 2020				X	X			
*Asiocaedius* G.S. Medvedev & Nepesova, 1985				X				
= *Pseudocaedius* G.S. Medvedev, 1966								
*Brachyidium* Fairmaire, 1883						X	X	X
= *Cnemodasus* Gebien, 1914								
*Caediexis* Lebedev, 1932				X				
*Caedius* Blanchard, 1845				X	X	X	X	
= *Isarida* Pascoe, 1866								
*Clitobius* Mulsant & Rey, 1859				X	X			
= *Halonomus* Wollaston, 1861								
= *Apithesis* C.O. Waterhouse, 1881								
= *Apteroclitobius* Koch, 1960								
*Coeloecetes* Blair, 1929						X		
*Corinta* Koch, 1950					X			
*Cornopterus* Koch, 1950					X			
*Cyptus* Gerstaecker, 1871				X	X			
= *Neocaedius* Pierre, 1972								
*Diaderma* Koch, 1960					X			
*Dilamus* Jacquelin du Val, 1861				X	X			
SGChoresmolamus G.S. Medvedev, 1978				X				
SGDilamus Jacquelin du Val, 1861				X	X			
SGOchrolamus Reitter, 1904				X	X			
*Emmalus* Erichson, 1843					X			
= *Emmallus* Agassiz, 1846								
= *Uzagaria* Ancey, 1881								
*Falsammidium* Koch, 1960					X			
*Falsocaedius* Español, 1943				X				
*Freyula* Koch, 1959					X			
*Hadrodes* Wollaston, 1877				X				
*Helenomelas* Ardoin, 1972				X				
*Mateuina* Español, 1944				X				
*Messoricolum* Koch, 1960					X			
*Moragacinella* Español, 1954				X				
= *Moralesia* Kaszab, 1944								
*Nesocaedius* Kolbe, 1915				X		X		
*Perithrix* Fairmaire, 1879				X				
*Platyprocnemis* Español & Lindberg, 1963					X			
*Plesioderes* Mulsant & Rey, 1859					X	X		
= *Epeurycaulus* Kolbe, 1902								
*Prodilamus* Ardoin, 1969				X	X			
*Proscheimus* Desbrochers des Loges, 1881				X				
*Psammestus* Reichardt, 1936				X				
*Pseudoleichenum* Ardoin, 1972					X			
*Raynalius* Chatanay, 1912					X			
*Tarphiophasis* Wollaston, 1877					X			
*Trigonopoda* Gebien, 1914						X		
*Weisea* Semenov, 1891				X				
**Blapstinina Mulsant & Rey, 1853**								
*Aconobius* Casey, 1895		X						
*Ammodonus* Mulsant & Rey, 1859		X	X					
= *Pseudonomus* Fairmaire, 1884								
= *Scaptes* Champion, 1886								
= *Trichotoides* Marcuzzi, 1954								
*Austrocaribius* Marcuzzi, 1954			X					
*Blapstinus* Dejean, 1821		X	X					*X*
= *Heteropus* Laporte, 1840								
= *Pedonoeces* G.R. Waterhouse, 1845								
= *Pedoeces* Agassiz, 1846								
= *Tessaromma* Boheman, 1858								
= *Aspidius* Mulsant & Rey, 1859								
= *Lachnoderes* Mulsant & Rey, 1859								
= *Lodinus* Mulsant & Rey, 1859								
= *Mecysmus* Horn, 1870								
*Cenophorus* Mulsant & Rey, 1859			X					
*Conibiosoma* Casey, 1890		X						
*Conibius* LeConte, 1851		X	X					*X*
= *Euconibius* Casey, 1895								
= *Gondwanodilamus* Kaszab, 1969 **syn. nov.**								
= *Ooconibius* Casey, 1895								
*Cybotus* Casey, 1890			X					
*Diastolinus* Mulsant & Rey, 1859			X					
= *Sellio* Mulsant & Rey, 1859								
= *Ctesicles* Champion, 1896								
*Goajiria* Ivie & Hart, 2016			X					
*Hummelinckia* Marcuzzi, 1954			X					
*Nevisia* Marcuzzi, 1986			X					
*Nocibiotes* Casey, 1895		X						
*Notibius* LeConte, 1851		X						
*Platylus* Mulsant & Rey, 1859			X					
*Tonibiastes* Casey, 1895		X						
*Tonibius* Casey, 1895		X						
*Trichoton* Hope, 1841		X	X					
= *Epilasium* Erichson, 1842								
= *Trichotum* Agassiz, 1846								
= *Bycrea* Pascoe, 1868								
*Ulus* Horn, 1870		X	X					
*Xerolinus* Ivie & Hart, 2016			X					
**Heterotarsina Blanchard, 1845**								
*Diphyrrhynchus* Fairmaire, 1849				X	X	X	X	X
= *Acanthosternus* Montrouzier, 1860								
= *Abantis* Fairmaire, 1892								
= *Abantiades* Fairmaire, 1894								
= *Neoabantis* Gebien, 1910								
*Heterocheira* Dejean, 1836					X		X	
= *Heterochira* Agassiz, 1846								
*Heterotarsus* Latreille, 1829				X	X	X		
= *Helopimorphus* Desbrochers des Loges, 1881								
= *Hopatropteron* Reitter, 1889								
= *Oubanghinum* Pic, 1933								
*Scymena* Pascoe, 1866							X	
**Neopachypterina Bouchard, Löbl & Merkl, 2007**								
*Amblysphagus* Fairmaire, 1896					X	X		
= *Trachymetus* Reitter, 1904								
†*Eupachypterus* Kirejtshuk, Nabozhenko & Nel, 2010								
*Neopachypterus* Bouchard, Löbl & Merkl, 2007				X		X		
= *Pachypterus* P.H. Lucas, 1847								
*Pseudolamus* Fairmaire, 1874				X				
**Opatrina Brullé, 1832**								
*Anatrum* Reichardt, 1936				X				
*Brachyesthes* Fairmaire, 1868				X		X		
*Caediomorpha* Blackburn, 1888							X	
*Ephalus* LeConte, 1862		X	X					
= *Pseudephalus* Casey, 1924								
*Eumylada* Reitter, 1904				X				
*Falsolobodera* Kaszab, 1967				X				
*Gonocephalum* Solier, 1834		*X*		X	X	X	X	X
SGGonocephalum Solier, 1834		*X*		X	X	X	X	X
= *Dasus* Motschulsky, 1845								
= *Megadasus* Reitter, 1904								
= *Hasticollinum* Kaszab, 1939								
SGMyladanesthes Skopin, 1961				X				
SGOpatropis Reitter, 1904				X	X	X		
*Hadrophasis* Ferrer, 1992					X			
*Jintaium* Ren, 1999				X				
*Melanesthes* Dejean, 1834				X				
SGLesbidana Reitter, 1904				X				
SGMelanesthes Dejean, 1834				X				
= *Hemitrichestes* Reitter, 1904								
= *Miglica* Reitter, 1904								
SGMongolesthes Reitter, 1904				X				
SGOpatronesthes Reitter, 1904				X				
*Melanocoma* Wollaston, 1868					X			
*Mesomorphus* Miedel, 1880				X	X	X	X	X
= *Pentholasius* Reitter, 1904								
= *Hopatromorpha* Blackburn, 1907								
*Myladina* Reitter, 1889				X				
*Opatroides* Brullé, 1832		*X*		X	X	X		
= *Hopatroides* Agassiz, 1846								
*Opatrum* Fabricius, 1775				X				
SGColpopatrum Reitter, 1904				X				
SGColpophorus Mulsant & Rey, 1859				X				
= *Colpophorinus* Escalera, 1914								
SGOpatrum Fabricius, 1775				X				
= *Hopatrum* Agassiz, 1846								
= *Thoracon* Gistel, 1848								
*Penthicinus* Reitter, 1896				X				
*Penthicus* Faldermann, 1836				X	X			
SGAllomyladion Bogatchev, 1972				X				
SGAulonolcus Reitter, 1904				X				
SGDiscotus Reitter, 1904				X	X			
SGMyladion Reitter, 1887				X				
= *Penthicomelanesthes* Bogatchev, 1972								
SGPenthicus Faldermann, 1836				X				
= *Lobodera* Mulsant & Rey, 1859								
= *Lobothorax* Gemminger, 1870								
= *Penthomegus* Reitter, 1904								
= *Stonavus* Reitter, 1904								
SGPseudopenthicinus Bogatchev, 1972				X				
*Phelopatrum* Marseul, 1876				X				
= *Pseudadrus* Fairmaire, 1897								
*Polycoelogastridion* Reichardt, 1936				X		X		
*Reichardtiellina* Kaszab, 1982				X				
= *Reichardtiella* Kaszab, 1942								
*Scleropatroides* Löbl & Merkl, 2003				X	X	X		
*Scleropatrum* Reitter, 1887				X		X		
= *Monatrum* Reichardt, 1936								
*Sinorus* Mulsant & Revelière, 1861				X				
*Sobas* Pascoe, 1863							X	
= *Trigonotarsus* Hope, 1843								
= *Pseudocaedius* Blackburn, 1890								
*Socotropatrum* Koch, 1970					X			
*Tidiguinia* Español, 1959				X				
*Trichosternum* Wollaston, 1861					X			
= *Trichopodus* Mulsant & Rey, 1859								
= *Japetus* Reitter, 1904								
*Wolladrus* Iwan & Kamiński, 2016				X				
= *Hadrus* Wollaston, 1854								
**Sclerina Lacordaire, 1859**								
*Eurycaulus* Fairmaire, 1868				X	X			
SGEurycaulinus Koch, 1937				X				
SGEurycaulus Fairmaire, 1868				X	X			
= *Ammotrypes* Fairmaire, 1879								
= *Scleronimon* Reitter, 1904								
= *Scleronopsis* Koch, 1935								
†*Palaeosclerum* Nabozhenko & Kirejtshuk, 2017								
*Platynosum* Mulsant & Rey, 1859				X				
= *Melanimon* Motschulsky, 1845								
*Sclerum* Dejean, 1834				X	X	X		
= *Scleron* Hope, 1841								
= *Anticlia* Gistel, 1848								
= *Chlamydion* Gistel, 1848								
**Stizopodina Lacordaire, 1859**								
*Adoryacus* Koch, 1963					X			
*Amathobius* Gebien, 1920					X			
*Blacodatus* Koch, 1963					X			
*Blenosia* Laporte, 1840					X			
= *Blacodes* Duponchel, 1842								
*Calaharena* Koch, 1963					X			
*Crististibes* Koch, 1963					X			
*Eichleria* Kamiński, 2015					X			
*Eremostibes* Koch, 1963					X			
*Helibatus* Mulsant & Rey, 1859					X			
= *Ennychius* Fåhraeus, 1870								
*Luebbertia* Koch, 1963					X			
*Microstizopus* Koch, 1963					X			
*Namazopus* Koch, 1963					X			
*Nemanes* Fairmaire, 1888					X			
*Parastizopus* Gebien, 1938					X			
= *Ennychiatus* Koch, 1963								
*Periloma* Gebien, 1938					X			
*Planostibes* Gemminger, 1870					X			
= *Planodes* Mulsant & Rey, 1859								
*Psammogaster* Koch, 1953					X			
*Sphaerostibes* Koch, 1963					X			
*Stizopus* Erichson, 1843					X			
= *Doryagus* Pascoe, 1887								
*Sulpius* Fairmaire, 1906					X			
*Syntyphlus* Koch, 1953					X			
**Opatrini *incertae sedis***								
*Hovarygmus* Fairmaire, 1898					X			
*Pachymastus* Fairmaire, 1896					X			
*Pocadiopsis* Fairmaire, 1896						X		
*Scleroides* Fairmaire, 1883							X	
*Trigonopilus* Fairmaire, 1893						X		
**Pedinini Eschscholtz, 1829**								
**Helopinina Lacordaire, 1859**								
*Amatodes* Dejean, 1834					X			
SGAmatodes Dejean, 1834					X			
= *Oncosoma* Westwood, 1843								
= *Oncoosoma* Gebien, 1911								
SGConophthalmus Quedenfeldt, 1885					X			
SGStrophiamixa Robiche, 2005					X			
= *Strophia* Robiche, 2004								
*Ametrocera* Fåhraeus, 1870					X			
= *Idricus* Fairmaire, 1888								
*Anaxius* Fåhraeus, 1870					X			
*Aptila* Fåhraeus, 1870					X			
*Asidodema* Koch, 1958					X			
*Blastarnodes* Koch, 1958					X			
*Diestecopus* Solier, 1848					X			
= *Blastarnus* Fairmaire, 1897								
*Drosochrus* Erichson, 1843				X	X			
SGDesertosochrus Koch, 1958					X			
SGDrosochrus Erichson, 1843				X	X			
SGHelopinus Solier, 1848				X	X			
= *Pteraulus* Solier, 1848								
= *Emyon* Gerstaecker, 1854								
*Micrantereus* Solier, 1848				X	X			
= *Solenomerus* Fåhraeus, 1870								
*Nicandra* Fairmaire, 1888					X			
SGCalous Koch, 1958					X			
SGHeteronicandra Koch, 1958					X			
SGNicandra Fairmaire, 1888					X			
SGOncotopsis Koch, 1958					X			
*Oncopteryx* Gebien, 1943					X			
= *Oncopterus* Fairmaire, 1887								
*Piscicula* Robiche, 2004					X			
*Psectes* Hesse, 1935					X			
**Leichenina Mulsant, 1854**								
*Apsheronellus* Bogatchev, 1967				X				
= *Microleichenum* G.S. Medvedev, 1973								
*Leichenum* Dejean, 1834		*X*	*X*	X	X	X	X	
= *Lichenum* Agassiz, 1846								
= *Endothina* Carter, 1924								
**Pedinina Eschscholtz, 1829**								
*Cabirutus* Strand, 1929				X		X		
SGCabirutus Strand, 1929				X				
= *Cabirus* Mulsant & Rey, 1853								
= *Asiobirus* G.S. Medvedev, 1968								
= *Dentibirus* G.S. Medvedev, 1968								
SGNeocabirutus Kulzer, 1964						X		
*Colpotinus* Fairmaire, 1891				X				
*Loensus* R. Lucas, 1920					X	X		
SGLoensus R. Lucas, 1920					X			
= *Pedinopsis* Gebien, 1910								
SGPseudopedinus Ardoin, 1969						X		
*Pedinus* Latreille, 1797				X		X		
SGBlindus Mulsant & Rey, 1853				X		X		
SGColpotus Mulsant & Rey, 1853				X				
SGPedinus Latreille, 1797				X				
= *Vadalus* Mulsant & Rey, 1853								
= *Pedinulus* Seidlitz, 1893								
**Platynotini Mulsant & Rey, 1853**								
**Eurynotina Mulsant & Rey, 1854**								
*Byrrhoncus* Koch, 1954					X			
*Capidium* Koch, 1954					X			
*Colophonesthes* Koch, 1954					X			
*Eurynotus* W. Kirby, 1819					X			
SGBiolus Mulsant & Rey, 1854					X			
SGEurynotus W. Kirby, 1819					X			
SGNeosolenopistoma Bouchard & Bousquet, **subgen. nov.**					X			
*Heteropsectropus* Kaszab, 1941					X			
*Hirtograbies* Koch, 1954					X			
*Isoncophallus* Koch, 1954					X			
*Menederes* Solier, 1848					X			
SGAnamenederes Koch, 1954					X			
SGMenederes Solier, 1848					X			
*Menederopsis* Koch, 1954					X			
= *Archinamaqua* Schawaller, 2012								
*Ograbies* Péringuey, 1899					X			
*Oncotus* Blanchard, 1845					X			
SGCilioncotus Koch, 1954					X			
SGMenoncotus Koch, 1954					X			
SGOncotus Blanchard, 1845					X			
SGQuadroncotus Koch, 1954					X			
*Phaleriderma* Koch, 1954					X			
*Phylacastus* Fairmaire, 1897					X			
*Psectrapus* Solier, 1848					X			
= *Psectropus* Gemminger, 1870								
*Schyzoschelus* Koch, 1954					X			
*Stridigula* Koch, 1954					X			
**Platynotina Mulsant & Rey, 1853**								
*Adamus* Iwan, 1997						X		
*Alaetrinus* Iwan, 1995		X	X					
*Amblychirus* Koch, 1956					X			
*Anchophthalmops* Koch, 1956		*X*			X			
= *Platykochius* Iwan, 2002								
*Anchophthalmus* Gerstaecker, 1854					X			
SGAnchophthalmus Gerstaecker, 1854					X			
= *Oxythorax* Fåhraeus, 1870								
= *Oncotiphallops* Koch, 1956								
SGKochogaster Kamiński & Raś, 2011					X			
= *Cosmogaster* Koch, 1956								
*Angolositus* Koch, 1955					X			
= *Aberlencus* Iwan, 2002								
= *Platymedvedevia* Iwan & Banaszkiewicz, 2007								
*Anomalipus* Guérin-Méneville, 1831					X			
= *Heteroscelis* Latreille, 1828								
= *Ectatocnemis* Horn, 1867								
= *Acmoeus* Fåhraeus, 1870								
= *Apodemus* Fåhraeus, 1870								
*Atrocrates* Koch, 1956					X			
*Atrocrypticanus* Iwan, 1999					X			
*Bantodemus* Koch, 1955					X			
*Clastopus* Fairmaire, 1898					X			
= *Hovademulus* Iwan, 1996								
*Claudegirardius* Iwan, 1999					X			
*Colpotinoides* Kaszab, 1975					X			
*Crypticanus* Fairmaire, 1897					X			
*Doyenus* Iwan, 1996					X			
*Ectateus* Koch, 1956					X			
*Eleoselinus* Kamiński, 2014					X			
*Eucolus* Mulsant & Rey, 1853						X		
= *Indeucolus* Kaszab, 1975								
*Eviropodus* Koch, 1956					X			
*Glyptopteryx* Gebien, 1910					X			
= *Microselinus* Koch, 1956								
= *Quadrideres* Koch, 1956								
= *Synquadrideres* Iwan, 2003								
*Gonopus* Latreille, 1828					X			
SGAgonopus Gebien, 1920					X			
SGGonopus Latreille, 1828					X			
*Hovademus* Iwan, 1996					X			
*Lechius* Iwan, 1995					X			
*Madobalus* Fairmaire, 1901					X			
*Melanocratus* Fairmaire, 1895					X			
*Melanopterus* Mulsant & Rey, 1854					X			
*Menearchus* Carter, 1920					X			
*Monodius* Koch, 1956					X			
*Nesopatrum* Gebien, 1921					X			
*Notocorax* Dejean, 1834						X		
= *Platydendarus* Kaszab, 1975								
*Opatrinus* Dejean, 1821			X					
= *Hopatrinus* Agassiz, 1846								
= *Dema* Gistel, 1848								
*Parabantodemus* Iwan, 2000					X			
*Paraselinus* Kamiński, 2013					X			
*Penthicoides* Fairmaire, 1896						X		
*Phallocentrion* Koch, 1956					X			
*Phymatoplata* Koch, 1956					X			
*Platyburak* Iwan, 1990						X		
*Platyburmanicus* Iwan, 2003						X		
*Platycolpotus* Iwan, 1997						X		
*Platynotoides* Kaszab, 1975						X		
*Platynotus* Fabricius, 1801						X		
= *Ixalus* Gistel, 1848								
*Pokryszkiella* Iwan, 1996					X			
*Pseudoblaps* Guérin-Méneville, 1834				X		X		
= *Nyctalops* Gistel, 1848								
*Pseudonotocorax* Iwan, 1997						X		
*Pteroselinus* Kamiński, 2015					X			
*Rugoplatynotus* Kaszab, 1975						X		
*Schelodontes* Koch, 1956					X			
= *Lawrenceus* Iwan, 1998								
= *Platycharlesus* Iwan, 1998								
= *Warchalowskiellus* Iwan, 1998								
*Sebastianus* Iwan, 1996					X			
*Selinopodus* Koch, 1956					X			
*Selinus* Mulsant & Rey, 1853					X			
*Stenogonopus* Gebien, 1938					X			
*Styphacus* Fairmaire, 1901					X			
*Trigonopus* Mulsant & Rey, 1853					X			
*Upembarus* Koch, 1956					X			
SGPseudoselinus Iwan, 2002					X			
SGUpembarus Koch, 1956					X			
*Zidalus* Mulsant & Rey, 1853				X	X			
= *Zodinus* Mulsant & Rey, 1853								
= *Apterozidalus* Ardoin, 1965								
*Zophodes* Fåhraeus, 1870					X			
**Platyscelidini Lacordaire, 1859**								
*Bioramix* Bates, 1879				X				
SGBioramix Bates, 1879				X				
SGCardiochianalus Kaszab, 1940				X				
SGChianalus Bates, 1879				X				
= *Botiras* Fairmaire, 1891								
SGEuryhelops Reitter, 1902				X				
= *Cardiobioramix* Kaszab, 1940								
SGFaustia Kraatz, 1882				X				
SGLeipopleura Seidlitz, 1893				X				
SGNudoplatyscelis Kaszab, 1940				X				
SGOvalobioramix Egorov, 2004				X				
SGPlanoplatyscelis Kaszab, 1940				X				
= *Pleioplatyscelis* Kaszab, 1940								
SGPlatynoscelis Kraatz, 1882				X				
SGTrichochianalus Kaszab, 1940				X				
SGTricholeipopleura Kaszab, 1940				X				
SGTrichoplatyscelis Reinig, 1931				X				
= *Pseudotrichoplatyscelis* Kaszab, 1960								
*Microplatyscelis* Kaszab, 1940				X				
*Myatis* Bates, 1879				X				
*Oodescelis* Motschulsky, 1845				X				
SGAcutoodescelis Kaszab, 1940				X				
SGClavatoodescelis Kaszab, 1940				X				
= *Oblongoodescelis* Kaszab, 1940								
SGConvexoodescelis Egorov, 2004				X				
SGLonguloodescelis Kaszab, 1940				X				
= *Trichoodescelis* Kaszab, 1940								
SGMontanoodescelis Egorov, 2004				X				
SGOodescelis Motschulsky, 1845				X				
= *Oodeoscelis* Agassiz, 1846								
SGOvaloodescelis Kaszab, 1940				X				
SGPlanoodescelis Egorov, 2004				X				
SGSpinoodescelis Kaszab, 1940				X				
SGSplenoodescelis Egorov, 2004				X				
SGTruncatoodescelis Kaszab, 1940				X				
*Platyscelis* Latreille, 1818				X				
SGOblongoplatyscelis Kaszab, 1940				X				
SGParaplatyscelis Kaszab, 1940				X				
SGPlatyscelis Latreille, 1818				X				
= *Kaszaboscelis* Löbl & Merkl, 2003								
SGPleiopleura Seidlitz, 1893				X				
*Somocoelia* Heyden & Kraatz, 1882				X				
*Somocoeloplatys* Skopin, 1968				X				
*Trichomyatis* Schuster, 1931				X				
= *Trichoplatynoscelis* Kaszab, 1940								
**Blaptinae *incertae sedis***								
*Stenolamus* Gebien, 1920					X			
**Tenebrioninae Latreille, 1802**								
**Acropteronini Doyen, 1989**								
*Acropteryx* Gistel, 1831			X					
= *Acropteron* Perty, 1832								
= *Sphenosoma* Dejean, 1834								
= *Acropterum* Agassiz, 1846								
= *Arthroplatus* Solier, 1851								
**Alphitobiini Reitter, 1917**								
*Alphitobius* Stephens, 1829		*X*	*X*	*X*	X	X	*X*	
= *Heterophaga* Dejean, 1834								
= *Cryptops* Solier, 1851								
= *Proselytus* Fåhraeus, 1870								
= *Microphyes* W.J. MacLeay, 1872								
= *Latetribolium* Lepesme, 1943								
†*Alphitopsis* Kirejtshuk, Nabozhenko & Nel, 2011								
*Ardoinia* Kaszab, 1969					X			
*Diaclina* Jacquelin du Val, 1861				X	X	X	X	
*Epipedodema* Gebien, 1921					X			
*Guanobius* Grimm, 2008						X		
*Hoplopeltis* Fairmaire, 1894						X		
*Peltoides* Laporte, 1833					X			
SGMicropeltoides Pic, 1916					X			
SGPeltoides Laporte, 1833					X			
= *Oopiestus* Chevrolat, 1833								
**Amarygmini Gistel, 1848**								
*Alienoplonyx* Bremer, 2019						X		
*Alymon* Pascoe, 1866					X			
= *Ghaleca* Péringuey, 1899								
*Amarygmus* Dalman, 1823				X		X	X	X
SGAmarygmus Dalman, 1823				X		X	X	X
= *Dietysus* Pascoe, 1866								
= *Elixota* Pascoe, 1866								
= *Eurypera* Pascoe, 1870								
= *Dictysus* Rye, 1874								
= *Aphyllocerus* Fairmaire, 1881								
= *Anacycus* Fairmaire, 1896								
= *Platolenes* Gebien, 1914								
= *Pseudamarygmus* Pic, 1915								
= *Apelina* Saha, 1988								
= *Plesiamarygmus* Masumoto, 1989								
SGBecvaramarygmus Masumoto, 1999						X		
SGCornugeton Bremer, 2016							X	
SGDryadigmus Bremer, 2007						X		
SGHyperamarygmus Kaszab, 1964							X	
SGOogeton Kaszab, 1941						X		
SGPhaenogeton Bremer, 2016							X	
SGPodamarygmus Carter, 1928						X		
SGPyanirygmus Pic, 1915						X		
SGVarogeton Bremer, 2014						X		
*Asthenochirus* Fairmaire, 1885					X			
*Asyleptus* Péringuey, 1896					X			
= *Barlacus* Fairmaire, 1900								
= *Termitonebria* Wasmann, 1925								
= *Falsozialeus* Pic, 1951								
*Atropsorodes* Ardoin, 1963					X			
*Axynaon* Blackburn, 1897							X	
= *Catopherus* Carter, 1918								
*Azarelius* Fairmaire, 1892						X		
*Bunamarygmus* Masumoto, 1988						X		
*Cantaloubeus* Ardoin, 1959					X			
*Caudamarygmus* Bremer, 2001						X		
*Cephalamarygmus* Bremer, 2001						X		
*Cerysia* Bremer, 2001						X	X	
*Chalcoplonyx* Ardoin, 1963					X			
*Chalcopteroides* Gebien, 1948							X	
= *Chalcopterus* Blessig, 1861								
*Cheiroplus* Ardoin, 1963					X			
*Cleognathus* Gebien, 1921					X			
*Coccimarygmus* Ardoin, 1966					X			
SGCoccimarygmus Ardoin, 1966					X			
SGLeiochromimus Ardoin, 1966					X			
*Crypsinous* Fairmaire, 1891					X			
= *Cryptadius* Fairmaire, 1894								
= *Ubangia* Gebien, 1914								
*Cymatothes* Dejean, 1834		X	X					
= *Physignathus* Gistel, 1834								
= *Pyanisia* Laporte, 1840								
= *Cymatodes* Agassiz, 1846								
*Dalmanius* Bremer, 2001						X		
*Dasyplonyx* Bremer, 2014						X		
*Dichotymus* Fairmaire, 1891					X			
*Erycastus* Fairmaire, 1897					X			
*Euglyptonotus* Gestro, 1901					X			
*Eulytus* C.O. Waterhouse, 1882					X			
*Eumolpamarygmus* Pic, 1923						X		
*Eumolparamarygmus* Bremer, 2006						X		
*Eupezoplonyx* Pic, 1922						X		
*Eupezus* Dejean, 1834					X			
*Euspinamarygmus* Masumoto, 1989						X		
*Fahraeus* Ardoin, 1963					X			
*Falsastenochirus* Pic, 1938					X			
= *Falsasthenochirus* Ardoin, 1965								
*Falsoplonyx* Ardoin, 1963					X			
*Falsosynopticus* Pic, 1936					X			
*Garambanus* Ardoin, 1964					X			
*Gonocnemis* J. Thomson, 1858					X	X		
= *Acastus* Péringuey, 1896								
*Gonocnemocistela* Pic, 1935					X			
*Hesseodes* Ardoin, 1963					X			
*Hoplobrachium* Fairmaire, 1886					X	X		
= *Holobrachium* Gebien, 1905								
= *Cephaloplonyx* Pic, 1922								
*Hoplonyx* J. Thomson, 1858					X			
SGHoplonyx J. Thomson, 1858					X			
= *Hoplochirus* Scudder, 1882								
SGHyloplonyx Ardoin, 1963					X			
SGNataloplonyx Ardoin, 1963					X			
SGNemoplonyx Ardoin, 1963					X			
*Hypamarygmus* Gebien, 1904					X			
*Insolitoplonyx* Bremer, 2014						X		
*Isopteroplonyx* Bremer, 2006							X	
*Javamarygmus* Pic, 1928						X		
*Lemoultia* Chatanay, 1913					X			
*Lobatopezus* Pic, 1952						X		
*Luzonoplonyx* Bremer, 2009						X		
*Macrosynopticus* Pic, 1922						X		
= *Cyrtostrongylium* Blair, 1929								
*Megacantha* Westwood, 1843					X			
*Meracantha* W. Kirby, 1837		X						
= *Falacer* Laporte, 1840								
= *Physocoelus* Haldeman, 1850								
*Meroxys* Ardoin, 1963					X			
*Mimosynopticus* Pic, 1922					X			
*Neoplonyx* Ardoin, 1963					X			
*Nepaloplonyx* Bremer, 2014						X		
*Nesioticus* Westwood, 1843					X			
*Oplocheirus* Lacordaire, 1859					X			
*Overlaetia* Pic, 1937					X			
*Paragonocnemis* Kraatz, 1899					X	X		
SGBorneogonocnemis Pic, 1936						X		
SGLycogonocnemis Pic, 1915					X			
SGMicrogonocnemis Pic, 1936					X			
SGParagonocnemis Kraatz, 1899					X			
*Paramarygmus* Quedenfeldt, 1885					X			
SGMeracanthoides Linell, 1896					X			
SGParamarygmus Quedenfeldt, 1885					X			
= *Pareupezus* Kolbe, 1889								
*Pilosoplonyx* Bremer, 2014						X		
*Pimelionotus* Ardoin, 1963					X			
*Platypsorodes* Ardoin, 1963					X			
*Plegacerus* Gebien, 1921					X			
*Plesiophthalmus* Motschulsky, 1857		*X*		X		X		
SGChaeroplonyx Bremer, 2014						X		
SGCyriogeton Pascoe, 1871						X		
SGEumolpocyriogeton Pic, 1922						X		
SGInspinogeton Pic, 1937						X		
SGOpacoplonyx Bremer, 2014				X		X		
SGPlesiophthalmus Motschulsky, 1857		*X*		X		X		
SGSpinamarygmus Pic, 1915						X		
*Plinthochrous* Fairmaire, 1891					X			
= *Lycoscelis* Blair, 1929								
*Podacamptus* Ardoin, 1964					X			
*Pontianacus* Fairmaire, 1898						X		
*Pseudalymon* Ardoin, 1969					X			
*Pseudoogeton* Masumoto, 1989				X		X		
*Psilocastus* Ardoin, 1963					X			
*Psoroderes* Ardoin, 1962					X			
*Psorodes* Dejean, 1834					X			
= *Acanthomera* Latreille, 1828								
= *Acanthomerus* Agassiz, 1846								
*Psorophodes* Ardoin, 1963					X			
*Pterodes* Ardoin, 1963					X			
*Pubamarygmus* Pic, 1915							X	
*Reichenspergeria* Wasmann, 1921						X		
*Seorsoplonyx* Bremer, 2010						X		
*Singapura* Gebien, 1925						X		
*Spathulipezus* Gebien, 1921							X	
*Spinodietysus* Pic, 1927						X		
*Stemmoderus* Spinola, 1842					X			
= *Stemmatoderus* Agassiz, 1846								
*Sylvanoplonyx* Bremer, 2010						X		
*Timogebienus* Ardoin, 1963					X			
*Trichamarygmus* Carter, 1913							X	
*Umslatus* Péringuey, 1899					X			
*Vutsimus* Péringuey, 1899					X			
*Ziaelas* Fairmaire, 1892						X		
**Apocryphini Lacordaire, 1859**								
*Apocrypha* Eschscholtz, 1831		X	X					
= *Compsomorphus* Solier, 1851								
*Diplocyrtus* Quedenfeldt, 1887				X				
*Plastica* C.O. Waterhouse, 1903			X					
*Pseudapocrypha* Champion, 1886			X					
**Bolitophagini W. Kirby, 1837**								
*Afrobyrsax* Ardoin, 1973					X			
*Atasthalomorpha* Miyatake, 1964				X				
*Atasthalus* Pascoe, 1871						X		
*Boletoxenus* Motschulsky, 1858				X		X		
= *Bolitoxenus* Gemminger, 1870								
*Bolitolaemus* Gebien, 1921					X			
*Bolitonaeus* Lewis, 1894				X		X		
*Bolitophagiella* Miyatake, 1964				X				
*Bolitophagus* Illiger, 1798		X		X				
= *Boletophagus* Agassiz, 1846								
*Bolitotherus* Candèze, 1861		X						
= *Phellidius* LeConte, 1862								
*Bolitotrogus* Miyatake, 1964				X		X		
*Byrsax* Pascoe, 1860				X		X	X	
*Eleates* Casey, 1886		X						
*Eledona* Latreille, 1797				X				
= *Heledona* Agassiz, 1846								
*Eledonoprius* Reitter, 1911				X				
*Lanhsia* Shibata, 1980						X		
*Megeleates* Casey, 1895		X						
*Microbolitonaeus* Grimm, 2014						X		
*Microatasthalus* Ando, 2010						X		
*Parabolitophagus* Miyatake, 1964				X				
†*Proteleates* Wickham,1914								
*Rhipidandrus* LeConte, 1862		X	X	X	X	X	X	X
= *Xyloborus* Motschulsky, 1860								
= *Eutomus* Lacordaire, 1865								
= *Heptaphylla* Friedenreich, 1883								
= *Cherostus* C.O. Waterhouse, 1894								
= *Bolitopertha* Gebien, 1910								
*Sumbawia* Gebien, 1925						X		
**Centronopini Doyen, 1989**								
*Centronopus* Solier, 1848		X	X					
SGCentronopus Solier, 1848			X					
= *Centropus* Jakobson, 1914								
SGMenechides Motschulsky, 1872		X	X					
= *Scotobates* Rye, 1877								
= *Pyres* Champion, 1885								
*Scotobaenus* LeConte, 1859		X						
*Tauroceras* Hope, 1841			X					
= *Tauroceropedus* Pic, 1913								
**Cerenopini Horn, 1870**								
*Argoporis* Horn, 1870		X						
= *Threnus* Motschulsky, 1870								
*Cerenopus* LeConte, 1851		X						
**Dissonomini G.S. Medvedev, 1968**								
*Bradyus* Dejean, 1834				X				
= *Aphaleria* Reitter, 1896								
*Dissonomus* Jacquelin du Val, 1861				X				
SGDissonomus Jacquelin du Val, 1861				X				
= *Heterophylus* Mulsant & Rey, 1859								
SGEudissonomus G.S. Medvedev, 1968				X				
SGNeodissonomus G.S. Medvedev, 1968				X				
SGParadissonomus G.S. Medvedev, 1968				X				
**Eulabini Horn, 1870**								
*Apsena* LeConte, 1862		X						
*Epantius* LeConte, 1851		X						
*Eulabis* Eschscholtz, 1829		X						
= *Heterarthron* Gistel, 1848								
**Falsocossyphini Ferrer, 2006**								
*Blatticephalus* Heller, 1918					X			
= *Catobleps* Blair, 1918								
*Falsocossyphus* Pic, 1916						X		
*Microblattellus* Ferrer, 2006						X		
**Heleini Fleming, 1821**								
**Asphalina Matthews & Lawrence, 2005**								
*Asphalus* Pascoe, 1868							X	
*Bassianus* Matthews & Doyen, 1989							X	
*Meneristes* Pascoe, 1869							X	
= *Asiris* Motschulsky, 1872								
*Sloanea* Carter, 1916							X	
**Cyphaleina Lacordaire, 1859**								
*Agastenes* R. Lucas, 1920							X	
= *Agasthenes* Bates, 1873								
= *Batessia* Ponting, 2018								
*Aglypta* Gebien, 1908							X	
= *Onoglypta* Carter, 1926								
*Amarygmimus* Bates, 1873							X	
= *Amarygmomimus* Rye, 1875								
*Amphianax* Bates, 1873							X	
*Atoreuma* Gebien, 1941							X	
= *Toreuma* Carter, 1913								
= *Eutoreuma* Carter, 1914								
*Bolbophanes* Carter, 1913							X	
= *Pseudobolbophanes* Kulzer, 1954								
*Byallius* Pascoe, 1869							X	
*Cyphaleus* Westwood, 1841							X	
= *Chrysobalus* Boisduval, 1835								
= *Chartopteryx* Westwood, 1841								
= *Oremasis* Pascoe, 1866								
= *Altes* Pascoe, 1869								
= *Anausis* Bates, 1873								
= *Apomestris* Bates, 1873								
= *Trisilus* Haag-Rutenberg, 1878								
*Hemicyclus* Westwood, 1841							X	
= *Cyclophanes* Carter, 1913								
*Mithippia* Pascoe, 1869							X	
*Mitrothorax* Carter, 1914							X	
= *Ctimene* Bates, 1873								
= *Mitrephorus* Carter, 1913								
= *Timeneca* Carter, 1914								
*Nyctozoilus* Guérin-Méneville, 1831							X	
= *Sphenogenius* Solier, 1848								
= *Onosterrhus* Pascoe, 1866								
= *Hypocilibe* Bates, 1872								
= *Aethalides* Bates, 1873								
= *Ononyctus* Carter, 1914								
*Olisthaena* Erichson, 1842							X	
= *Decialma* Pascoe, 1869								
= *Hectus* Pascoe, 1869								
= *Aphectus* Carter, 1926								
*Onotrichus* Carter, 1911							X	
*Pachycoelia* Boisduval, 1835							X	
= *Lepispilus* Westwood, 1841								
= *Lepidospilus* Agassiz, 1846								
= *Tyndarisus* Pascoe, 1869								
*Paraphanes* W.J. MacLeay, 1887							X	
*Phanechloros* Matthews & Bouchard, 2008							X	
= *Chlorophanes* Matthews, 1992								
*Platyphanes* Westwood, 1849							X	
= *Opigenia* Pascoe, 1869								
= *Laonicus* Haag-Rutenberg, 1878								
*Prophanes* Westwood, 1849							X	
= *Lygestira* Pascoe, 1866								
= *Maerodes* C.O. Waterhouse, 1877								
= *Moerodes* Rye, 1879								
*Styrus* Bates, 1873							X	
**Heleina Fleming, 1821**								
*Boreosaragus* Matthews, 1993							X	
*Brises* Pascoe, 1869							X	
= *Ephidonius* Pascoe, 1869								
*Camponotiphilus* Lea, 1914							X	
*Celibe* Boisduval, 1835							X	
*Cillibus* Matthews, 1993							X	
*Dysarchus* Pascoe, 1866							X	
= *Saragodinus* Bates, 1872								
= *Saragella* Carter, 1937								
*Edylius* Champion, 1894							X	
*Emcephalus* W. Kirby, 1828							X	
= *Encephalus* Agassiz, 1846								
= *Ellaemus* Pascoe, 1866								
= *Encara* Gemminger, 1870								
= *Euhelaeus* Gebien, 1921								
*Helea* Latreille, 1804							X	
= *Soradeus* Rafinesque, 1815								
= *Elaeus* Gemminger, 1870								
*Mimopeus* Pascoe, 1866							X	
*Ospidus* Pascoe, 1866							X	
*Pterohelaeus* Brême, 1842							X	
= *Barytipha* Pascoe, 1869								
= *Pterelaeus* Gemminger, 1870								
= *Pezohelaeus* Gebien, 1921								
*Saragus* Erichson, 1842							X	
= *Cyclosattus* Casey, 1892								
*Sympetes* Pascoe, 1866							X	
*Trichosaragus* Blackburn, 1890							X	
**Heleini *incertae sedis***								
*Cerodolus* Sharp, 1886							X	
*Pseudhelops* Guérin-Méneville, 1841							X	
**Helopini Latreille, 1802**								
**Cylindrinotina Español, 1956**								
*Armenohelops* Nabozhenko, 2002				X				
*Asialassus* Nabozhenko & Ando, 2018				X		X		
*Ceratopelius* Antoine, 1963				X				
*Cylindrinotus* Faldermann, 1837				X				
= *Cylindronotus* Agassiz, 1846								
= *Stenomacidius* Seidlitz, 1895								
*Ectromopsis* Antoine, 1949				X				
*Eustenomacidius* Nabozhenko, 2006				X				
SGCaucasohelops Nabozhenko, 2006				X				
SGEustenomacidius Nabozhenko, 2006				X				
*Gunarus* Gozis, 1886				X				
*Idahelops* Keskin & Nabozhenko, 2012				X				
*Microdocnemis* Nabozhenko & Keskin, 2010				X				
*Nalassus* Mulsant, 1854		X		X		X		
SGCaucasonotus Nabozhenko, 2000				X				
SGHoristelops Gozis, 1910				X				
= *Helopondrus* Reitter, 1922								
SGNalassus Mulsant, 1854		X		X		X		
= *Helopocerodes* Reitter, 1922								
SGNipponalassus Nabozhenko & Ando, 2018				X				
*Odocnemis* Allard, 1876				X				
SGHeloponotus Reitter, 1922				X				
SGOdocnemis Allard, 1876				X				
= *Omaleis* Allard, 1876								
= *Isopedus* Stein, 1877								
= *Homalus* Rye, 1878								
= *Homaleis* Rye, 1879								
= *Odontocnemis* Rye, 1878								
*Pseudoprobaticus* Nabozhenko, 2001				X				
*Reitterohelops* Skopin, 1960				X				
*Stenomax* Allard, 1876				X				
SGPystelops Gozis, 1910				X				
= *Asyrmatus* Canzoneri, 1959 **syn. nov.**								
SGStenomax Allard, 1876				X				
*Stygohelops* Leo & Liberto, 2003				X				
*Taurohelops* Keskin & Nabozhenko, 2015				X				
*Turkmenohelops* G.S. Medvedev, 1987				X				
*Turkonalassus* Keskin, Nabozhenko & Alpagut-Keskin, 2017				X				
*Xanthohelops* Nabozhenko, 2006				X				
*Xanthomus* Mulsant, 1854				X				
*Zophohelops* Reitter, 1902				X				
SGZophohelops Reitter, 1902				X				
= *Euryhelops* Reitter, 1902								
SGZophondrus Nabozhenko, 2014				X				
**Enoplopodina Reitter, 1917**								
*Accanthopus* Dejean, 1821				X				
= *Enoplopus* Solier, 1848								
**Helopina Latreille, 1802**								
*Adelphinus* Fairmaire & Coquerel, 1866				X				
SGAdelphinops Reitter, 1922				X				
SGAdelphinus Fairmaire & Coquerel, 1866				X				
*Allardius* Ragusa, 1898				X				
= *Pseudoparablops* Heyden, 1908								
*Apterotarpela* Kaszab, 1954						X		
*Catomus* Allard, 1876				X				
SGCatomodontus Löbl & Merkl, 2020				X				
SGCatomus Allard, 1876				X	X			
= *Catomidius* Seidlitz, 1895								
SGMontanocatomus Nabozhenko, 2006				X				
SGSinocatomus Nabozhenko, 2006				X				
†*Cryptohelops* Nabozhenko & Kirejtshuk, 2014								
*Deretus* Gahan, 1900					X			
*Entomogonus* Solier, 1848				X				
SGDelonurops Reitter, 1922				X				
= *Macrophanes* Iablokoff-Khnzorian, 1957								
SGEntomogonus Solier, 1848				X				
SGEutelogonus Reitter, 1922				X				
*Erionura* Reitter, 1903				X				
*Euboeus* Boieldieu, 1865				X				
SGEuboeus Boieldieu, 1865				X				
= *Probaticus* Seidlitz, 1895								
SGHelopidoxus Reitter, 1922				X				
SGHelopostygnus Antoine, 1949				X				
SGHelopotrichus Reitter, 1922				X				
SGPelorinus Vauloger de Beaupré, 1900				X				
*Hedyphanes* Fischer von Waldheim, 1820				X				
SGGranulophanes Nabozhenko, 2013				X				
SGHedyphanes Fischer von Waldheim, 1820				X				
= *Coelophanes* Iablokoff-Khnzorian, 1964								
SGMicrohedyphanes Nabozhenko & Lillig, 2013				X				
*Helops* Fabricius, 1775		X	X	X				
= *Hypulus* Rafinesque, 1815								
= *Anteros* Laporte, 1840								
= *Stenotrichus* LeConte, 1862								
= *Biomorphus* Motschulsky, 1872								
= *Coscinoptilix* Allard, 1876								
= *Mesohelops* Reitter, 1922								
*Italohelops* Español, 1961				X				
*Mamorina* Antoine, 1951				X				
*Nautes* Pascoe, 1866		X	X					
*Neohelops* Dajoz, 2001		X						
*Nephodinus* Gebien, 1943				X				
SGNephodinus Gebien, 1943				X				
= *Nephodes* Blanchard, 1845								
SGParanephodes Antoine, 1955				X				
*Nesotes* Allard, 1876				X				
SGHelopogonus Reitter, 1922				X				
SGNesotes Allard, 1876				X				
= *Diastixus* Allard, 1876								
= *Gyrinodes* Fauvel, 1897 **syn. nov.**								
*Nipponohelops* Masumoto, Ando & Akita, 2006				X				
*Physohelops* Schuster, 1937				X				
*Raiboscelis* Allard, 1876				X				
= *Hipponome* Laporte, 1840								
= *Rhaebosceles* Rye, 1878								
*Sabularius* Escalera, 1914				X				
*Socotraphanes* Nabozhenko, 2019					X			
*Stenohelops* Reitter, 1922				X				
SGHelopelius Reitter, 1922				X				
= *Stenomaleis* Español, 1957								
SGStenohelops Reitter, 1922				X				
= *Gunarellus* Reitter, 1922								
SG †*Stenolassus* Nabozhenko, Chigray & Bukejs, 2020								
*Tarpela* Bates, 1870		X	X					
= *Lamperos* Allard, 1876								
**Helopini *incertae sedis***								
*Dolphus* Blanchard, 1847			X					
*Erulipothydemus* Pic, 1918						X		
*Helopidesthes* Fairmaire, 1895					X			
*Microcatomus* Pic, 1925					X			
**Melanimonini Seidlitz, 1894 (1854)**								
*Cheirodes* Gené, 1839		X		X	X		*X*	
SGAnemiadena Bouchard & Bousquet, **subgen. nov.**					X			
SGCheirodes Gené, 1839		X		X	X		*X*	
= *Anemia* Laporte, 1840								
= *Chirodes* Agassiz, 1846								
SGHistiaea Fairmaire, 1892				X	X			
SGPseudanemia Wollaston, 1864				X	X			
= *Ammidanemia* Reitter, 1904								
SGSpinanemia Löbl, Bouchard, Merkl & Bousquet, 2020				X	X			
SGTrichanemia Ardoin, 1971					X			
*Dolamara* Reichardt, 1935				X				
*Melanimon* Steven, 1828				X				
= *Microzoum* Dejean, 1834								
= *Microzoon* Hope, 1841								
= *Fundulus* Gistel, 1848								
**Metaclisini Steiner, 2016**								
*Metaclisa* Jacquelin du Val, 1861		X	X	X		X		
= *Amarantha* Motschulsky, 1860								
= *Tharsus* LeConte, 1862								
**Palorini Matthews, 2003**								
*Astalbus* Fairmaire, 1900					X			
*Austropalorus* Halstead, 1967							X	
*Eutermicola* Lea, 1916							X	
*Palorinus* Blair, 1930						X	X	
*Paloropsis* Masumoto & Grimm, 2004						X		
*Palorus* Mulsant, 1854		*X*	*X*	X	X	X	X	X
= *Caenocorse* C.G. Thomson, 1859								
= *Eba* Pascoe, 1863								
= *Platyotus* Gerstaecker, 1871								
= *Circomus* Fleischer, 1900								
= *Stenopalorus* Blair, 1930								
*Platycotylus* Olliff, 1883					X	X	X	
= *Thurea* Ferrer, 1998								
*Prolabrus* Fairmaire, 1897					X			
*Pseudeba* Blackburn, 1903							X	
*Ulomina* Baudi di Selve, 1876		*X*	*X*	X	X	X	*X*	X
= *Coelopalorus* Blair, 1930								
*Ulomotypus* Broun, 1886							X	
†*Vabole* Alekseev & Nabozhenko, 2015								
**Paoligenini Ferrer, 2013**								
*Paoligena* Pic, 1928					X			
**Praeugenini De Moor, 1970**								
*Anarmostodera* Fairmaire, 1897					X			
*Dysgena* Mäklin, 1863					X			
*Miltoprepes* Gerstaecker, 1871					X			
= *Anephyctus* Fairmaire, 1891								
*Nesogena* Mäklin, 1863					X			
SGArmigena Bouchard & Bousquet, **subgen. nov.**					X			
SGHeterogena Froussart, 1961					X			
SGNesogena Mäklin, 1863					X			
= *Bradygena* Fairmaire, 1903								
SGParagena Bouchard & Bousquet, **subgen. nov.**					X			
*Phaeostolus* Fairmaire, 1884					X			
*Praeugena* Laporte, 1840					X			
= *Adelphus* Dejean, 1834								
= *Praogena* Agassiz, 1846								
= *Lamprobothris* Fairmaire, 1887								
= *Tactoderus* Fairmaire, 1892								
= *Ergenna* Fairmaire, 1897								
*Pseudopraeugena* De Moor, 1970					X			
**Rhysopaussini Wasmann, 1896**								
*Mimoxenotermes* Pic, 1931						X		
*Rhysopaussus* Wasmann, 1896						X		
*Rhyzodina* Chevrolat, 1873					X			
SGApistocerus Fairmaire, 1899					X			
SGEurhysodina Wasmann, 1921					X			
SGRhyzodina Chevrolat, 1873					X			
= *Rhysodina* Wasmann, 1921								
*Xenotermes* Wasmann, 1896						X		
**Scaurini Billberg, 1820**								
*Carchares* Pascoe, 1887					X			
= *Podoces* Péringuey, 1886								
*Cephalostenus* Solier, 1838				X				
= *Stenocephalus* Agassiz, 1846								
*Herpiscius* Solier, 1838					X			
*Scaurus* Fabricius, 1775				X	X			
= *Scauris* Rafinesque, 1815								
**Scotobiini Solier, 1838**								
*Ammophorus* Guérin-Méneville, 1831			X					
= *Selenomma* Dejean, 1836								
*Diastoleus* Solier, 1838			X					
*Emmallodera* Blanchard, 1842			X					
*Leptynoderes* Solier, 1838			X					
*Pumiliofossorum* Silvestro & Giraldo-Mendoza, 2015			X					
*Scotobius* Germar, 1823			X					
= *Gonogenius* Solier, 1838								
**Tenebrionini Latreille, 1802**								
*Ariarathus* Fairmaire, 1891				X		X		
= *Teneatopus* Reitter, 1920								
*Athrodactyla* Klug, 1833					X			
*Bius* Dejean, 1834		X		X				
= *Bia* Hope, 1841								
= *Dendroscopius* Gistel, 1848								
*Bouchardandrus* Steiner, 2016		X						
*Bremerus* Ferrer, 2004					X			
*Cedrosius* Fairmaire, 1902					X			
*Falsocalcar* Pic, 1925					X			
*Gridellia* Kammerer, 2006					X			
= *Villiersia* Gridelli, 1951								
*Hipalmus* Bates, 1870			X					
= *Lobetas* Motschulsky, 1872								
*Idiobates* Casey, 1891		X						
*Microzophobas* Pic, 1944			X					
*Neatus* LeConte, 1862		X		X				
*Neozophobas* Ferrer, 2006			X					
*Paratoxicum* Champion, 1894							X	
= *Schizophthalmotribolium* Kaszab, 1940								
*Phanerops* Solier, 1851			X					
*Rhinandrus* LeConte, 1866		X	X					
= *Exerestus* Bates, 1870								
= *Proderops* Fairmaire, 1873								
*Satanocalcar* Pic, 1925					X			
*Tenebrio* Linnaeus, 1758		*X*	*X*	X	X	X	*X*	
SGAfrotenebrio Gridelli, 1951					X			
SGCruracurvamtenebrio Robiche, 2019					X			
SGMegatenebrio Gridelli, 1951					X			
SGTenebrio Linnaeus, 1758		*X*	*X*	X	X	X	*X*	
= *Menedrio* Motschulsky, 1872								
= *Tenebrionellus* Crotch, 1874								
*Trichotenebrio* Ardoin, 1962					X			
*Zophobas* Dejean, 1834		X	X		X			
SGMacrozophobas Pic, 1913		X	X					
SGZophobas Dejean, 1834			X		X			
= *Pythonissus* Gistel, 1834								
**Titaenini Fauvel, 1905**								
*Artystona* Bates, 1874							X	
*Callismilax* Bates, 1874							X	
*Demtrius* Broun, 1895							X	
*Partystona* Watt, 1992							X	
*Titaena* Erichson, 1842							X	
**Toxicini Oken, 1843**								
**Dysantina Gebien, 1922**								
*Calymmus* Montrouzier, 1860							X	
*Cylindrosia* Gebien, 1922					X			
*Diceroderes* Solier, 1841			X					
= *Prosomenes* Blanchard, 1845								
*Dysantes* Pascoe, 1869					X	X		
= *Eudysantes* Bouchard, Lawrence, Davies & Newton, 2005								
*Ilyxerus* Pascoe, 1866							X	
*Mychestes* Pascoe, 1870							X	
*Opostirus* Kirsch, 1865			X					
*Orcopagia* Pascoe, 1868							X	
*Ozolais* Pascoe, 1866			X					
*Wattius* Kaszab, 1982			X					
**Nycteropina Lacordaire, 1859**								
*Chalcostylus* Fairmaire, 1898					X			
*Macellocerus* Solier, 1848					X			
= *Dolichoderus* Klug, 1833								
= *Dillacerus* Solier, 1835								
= *Stierlinius* Forel, 1893								
*Nycteropus* Klug, 1833					X			
**Toxicina Oken, 1843**								
*Cryphaeus* Klug, 1833				X	X	X	X	
= *Anthracias* Dejean, 1834								
= *Ardelio* Gistel, 1848								
*Epitoxicum* Bates, 1873						X		
*Taiwanocryphaeus* Masumoto, 1996						X		
*Toxicum* Latreille, 1802				X		X	X	
SGMutiloxicum Nabozhenko & Ivanov, 2018				X				
SGToxicum Latreille, 1802				X		X	X	
= *Trestonia* Rafinesque, 1815								
**Trachelostenini Lacordaire, 1859**								
*Leaus* Matthews & Lawrence, 1992							X	
*Myrmecodema* Gebien, 1943			X					
= *Myrmecosoma* Germain, 1855								
*Trachelostenus* Solier, 1851			X					
**Triboliini Gistel, 1848**								
*Aesymnus* Champion, 1886			X					
*Hypogena* Dejean, 1834		X	X					
= *Ulosonia* Laporte, 1840								
*Latheticus* C.O. Waterhouse, 1880		*X*	*X*	*X*	*X*	X	*X*	
*Lyphia* Mulsant & Rey, 1859		*X*		X	X	X	X	
= *Lindia* Blackburn, 1888								
*Metulosonia* Bates, 1873			X					
*Mycotrogus* Horn, 1870		X	X					
*Platybolium* Blair, 1938						X		
*Spelaebiosis* Bousquet & Bouchard, 2018			X					
= *Orghidania* Ardoin, 1977								
= *Ardoinia* Özdikmen, 2005								
*Tribolium* W.S. MacLeay, 1825		X	X	X	X	X	X	X
SGAphanotus LeConte, 1862		X						
SGTribolium W.S. MacLeay, 1825		X	X	X	X	X	X	X
= *Stene* Stephens, 1832								
= *Margus* Dejean, 1834								
= *Tenebrioloma* Gebien, 1910								
= *Leanum* Uyttenboogaart, 1934								
*Xenogloeus* Wollaston, 1861					X			
**Ulomini Blanchard, 1845**								
*Achthosus* Pascoe, 1863							X	
*Alegoria* Laporte, 1840			X					
= *Hylonoma* Macquart, 1850								
*Antimachus* Gistel, 1829			X					
= *Ceratupis* Perty, 1830								
*Apteruleda* Gebien, 1928			X					
*Apteruloma* Gebien, 1928			X					
*Basanopsis* Gebien, 1914						X		
*Brachypophlaeus* Fairmaire, 1897				X	X	X		
= *Leptoscapha* Fairmaire, 1886								
= *Stenoscapha* Fairmaire, 1885								
*Cenoscelis* Wollaston, 1868				X	X	X	X	
SGAptereutochia Kaszab, 1980						X		
SGCenoscelis Wollaston, 1868				X	X	X	X	
*Cneocnemis* Gebien, 1914				X		X	X	
*Curtopeltoides* Pic, 1916						X		
*Donisiellus* Bremer, 1992					X			
*Eutochia* LeConte, 1862		X	X					
= *Aniara* Melsheimer 1853:139								
= *Delopygus* LeConte, 1866								
= *Aniarus* Gemminger, 1870								
= *Holaniara* Fairmaire, 1871								
*Macruloma* Pic, 1921						X		
*Metabolocerus* Bates, 1873			X					
*Microcenoscelis* Schawaller, 2015					X			
*Neopsectropus* Kaszab, 1941					X			
*Neooligocara* Guerrero, Vidal & Moore, 2007			X					
*Oligocara* Solier, 1848			X					
*Pheres* Champion, 1886			X					
*Pycnuloma* Fairmaire, 1896						X		
*Scotochares* Boheman, 1858								X
*Semieutochia* Kaszab, 1980						X		
*Typhluloma* Lea, 1912							X	
*Uleda* Laporte, 1840			X					
*Uloma* Dejean, 1821		X	X	X	X	X	X	X
SGApterulomoides Kaszab, 1982							X	
SGUloma Dejean, 1821		X	X	X	X	X	X	X
= *Prioscelida* White, 1846								
= *Melasia* Perroud & Mulsant, 1856								
*Ulomimus* Bates, 1873				X		X		
= *Pseuduloma* Fairmaire, 1893								
**Tenebrioninae *incertae sedis***								
*Anophthalmolamus* Ferrer, 1993				X				
*Hangaya* Matthews & Merkl, 2015							X	
*Penichrus* Champion, 1885			X					
**Alleculinae Laporte, 1840**								
**Alleculini Laporte, 1840**								
**Alleculina Laporte, 1840**								
*Aeanes* Champion, 1893			X					
*Alethia* Champion, 1888		X	X					
*Allecula* Fabricius, 1801			X	X	X	X		
SGAllecula Fabricius, 1801			X	X	X	X		
SGAlleculina Pic, 1954					X			
SGDietopsis Solier, 1835			X					
*Alogista* Fåhraeus, 1870					X			
*Amaropsis* Champion, 1893			X					
*Anognathena* Ando, 2017						X		
*Anthracula* Fairmaire, 1897						X		
*Atoichus* Carter, 1915							X	
*Barbora* Novák, 2020						X		
*Barycistela* Blackburn, 1891							X	
*Blepusa* Westwood, 1842			X					
*Bobina* Novák, 2015				X		X		
*Bobisthes* Novák, 2019						X		
*Bolbostetha* Fairmaire, 1896						X		
= *Alleculodes* Borchmann, 1925								
*Borbochara* Novák, 2009						X		
*Borbonalia* Novák, 2014				X		X		
*Borborella* Novák, 2020						X		
*Borboresthes* Fairmaire, 1897				X		X		
*Charisius* Champion, 1888			X					
= *Narses* Champion, 1888								
*Chitwania* Novák, 2015						X		
*Cistelampra* Fairmaire, 1897					X			
*Cisteloida* Fairmaire, 1882						X		
*Cistelopsis* Fairmaire, 1896				X		X		
*Cteisodella* Novák, 2020						X		
*Cteisodes* Borchmann, 1932						X		
*Dimorphochilus* Borchmann, 1908							X	
*Diopoenus* Champion, 1888			X					
*Doranalia* Novák, 2020				X		X		
*Dorota* Novák, 2018						X		
*Erzika* Novák, 2020						X		
*Eucaliga* Fairmaire & Germain, 1861			X					
*Euomma* Boheman, 1858							X	
= *Apellatus* Pascoe, 1863								
*Evaostetha* Novák, 2008						X		
*Fifina* Novák, 2018						X		
*Fifinoides* Novák, 2020						X		
*Gerdacula* Novák, 2015						X		
*Havanalia* Novák, 2020				X				
*Hemicistela* Blackburn, 1891							X	
*Homotrysis* Pascoe, 1866							X	
= *Hybrenia* Pascoe, 1866								
*Houaphanica* Novák, 2020						X		
*Hymenalia* Mulsant, 1856				X	X	X		
*Hymenorus* Mulsant, 1852		X	X	X		X		
*Impressallecula* Pic, 1951					X			
*Indricula* Novák, 2016						X		
*Jaklia* Novák, 2010						X		
*Jophon* Champion, 1895							X	
†*Jurallecula* L.N. Medvedev, 1969								
*Knausia* Fall, 1931		X						
*Kombacula* Novák, 2012						X		
*Ksukolcula* Novák, 2017						X		
*Latacula* Campbell, 1971			X					
*Lepturidea* Fauvel, 1862							X	
= *Atractus* Boisduval, 1835								
= *Aethyssius* Pascoe, 1863								
= *Chromomaea* Pascoe, 1866								
= *Alcmeonis* Bates, 1868								
= *Anaxo* Bates, 1868								
= *Licymnius* Bates, 1868								
= *Neoatractus* Borchmann, 1909								
*Liodocistela* Pic, 1930						X		
*Litopous* Matthews, 2012							X	
*Lobopoda* Solier, 1835		X	X					
SGFlavipoda Campbell, 1966			X					
SGGlabrilobopoda Campbell, 1966			X					
SGLobopoda Solier, 1835		X	X					
SGMesolobopoda Campbell, 1966		X	X					
SGMonoloba Solier, 1835			X					
*Loriculoides* Novák, 2020						X		
= *Loricula* Novák, 2016								
*Lycula* Campbell, 1976			X					
*Macrocistelopsis* Pic, 1956							X	
*Madreallecula* Kanda, 2013		X						
*Magdanalia* Novák, 2020				X				
*Makicula* Novák, 2012						X		
*Matthewsotys* Bouchard & Bousquet, **gen. nov.**							X	
*Menes* Champion, 1888			X					
*Menoeceus* Champion, 1888		X	X					
*Metistete* Pascoe, 1866							X	
= *Lisa* Haag-Rutenberg, 1879								
*Microcistelopsis* Pic, 1922							X	
	= *Microcistela* Pic, 1919							
*Microstenogena* Pic, 1924					X			
*Microsthes* Novák, 2011						X		
*Mimopraogena* Pic, 1952					X			
*Mycetocharina* Seidlitz, 1890				X				
SGAlleculopsis Semenov, 1894				X				
SGMycetocharina Seidlitz, 1890				X				
= *Caristela* Fairmaire, 1894								
*Mycetocula* Novák, 2015						X		
*Narsodes* Campbell, 1976			X					
*Nesogenomorpha* Pic, 1917					X			
*Neocistela* Borchmann, 1909							X	
= *Pseudocistela* Blackburn, 1891								
*Netopha* Fairmaire, 1893				X		X		
*Nikomenalia*Dubrovina, 1975				X		X		
*Nocar* Blackburn, 1891							X	
*Notacula* Campbell, 1971			X					
*Notocistela* Carter, 1915							X	
*Nypsius* Champion, 1895							X	
*Obesacula* Campbell, 1971			X					
*Ommatochara* Borchmann, 1932						X		
*Ommatophorus* W.J. MacLeay, 1872							X	
*Omocula* Borchmann, 1937			X					
*Oocistela* Borchmann, 1908							X	
= *Melaps* Carter, 1908								
*Oracula* Novák, 2019					X			
SGDuocula Novák, 2019					X			
SGOracula Novák, 2019					X			
*Orchesiolobopoda* Pic, 1919			X					
*Palpichara* Borchmann, 1932						X		
*Palpicula* Novák, 2018						X		
*Parahymenorus* Campbell, 1971			X					
*Pemanoa* Buck, 1955							X	
*Petrostetha* Novák, 2008						X		
*Phediodes* Campbell, 1976			X					
*Phedius* Champion, 1888		X	X					
*Pitholaus* Champion, 1888			X					
*Pizura* Novák, 2016						X		
*Platyallecula* Blair, 1935					X			
*Polyidus* Champion, 1888			X					
*Potocula* Novák, 2012						X		
*Prionalia* Novák, 2020				X				
*Prionychus* Solier, 1835				X				
= *Eryx* Stephens, 1832								
= *Pelops* Gistel, 1834								
*Pseudocistelopsis* Novák, 2018						X		
*Psis* Novák, 2019						X		
*Punctacula* Campbell, 1971			X					
*Scaletomerus* Blackburn, 1891							X	
= *Otys* Champion, 1895								
*Scaphinion* Matthews, 2012							X	
*Simarus* Borchmann, 1909							X	
= *Ismarus* Haag-Rutenberg, 1878								
*Socotralia* Novák, 2007					X			
*Spinecula* Novák, 2019						X		
*Stenochidus* LeConte, 1862		X						
*Stenogena* Fairmaire, 1895					X			
*Stilbocistela* Borchmann, 1932						X		
*Synallecula* Kolbe, 1883					X			
*Tanychilus* Newman, 1838							X	
*Taxes* Champion, 1895							X	
*Telesicles* Champion, 1888		X						
*Temnes* Champion, 1888			X					
*Theatetes* Champion, 1888			X					
*Upinella* Mulsant, 1857				X		X		
SGThornella Novák, 2019						X		
SGTibinella Novák, 2019				X		X		
SGUpinella Mulsant, 1856				X		X		
*Vietnalia* Novák, 2021						X		
*Zizu* Novák, 2019						X		
**Gonoderina Seidlitz, 1896**								
*Andrimus* Casey, 1891		X						
*Androchirus* LeConte, 1862		X						
*Asiomira*Dubrovina, 1973				X				
= *Kirgisomira* Weise, 1974								
*Brachycula* Fairmaire, 1906					X			
†*Calcarocistela* Nabozhenko, 2016								
*Capnochroa* LeConte, 1862		X						
*Chromatia* LeConte, 1862		X						
*Copistethus* Seidlitz, 1890				X				
*Cornucistela* Campbell, 1980				X				
*Cryptomysia* Pic, 1954					X			
*Eubalia* Laporte, 1840					X			
= *Plesia* Klug, 1833								
= *Cacoplesia* Fairmaire, 1898								
*Gerandryus* Rottenberg, 1873				X				
= *Parablops* Rottenberg, 1871								
*Gonodera* Mulsant, 1856				X				
*Helopsisomira* Pic, 1952					X			
*Isomira* Mulsant, 1856		X	X	X	X	X		
SGApteromira Weise, 1974				X				
SGDanielomira Weise, 1974				X				
SGHeteromira Hölzel, 1958				X				
SGIsomira Mulsant, 1856		X	X	X		X		
= *Tedinus* Casey, 1891								
SGMucheimira Novák, 2016				X		X		
SGParaisomiraDubrovina, 1982				X				
SGPubeirosoma Pic, 1954					X			
*Kralia* Novák, 2013				X				
*Malaymira* Novák, 2020						X		
*Micrisomira* Pic, 1930						X		
*Microcistela* Pic, 1904				X				
*Onychomira* Campbell, 1984		X						
*Paracistela* Borchmann, 1941				X		X		
*Piccula* Bousquet & Bouchard, 2015					X			
= *Gerardia* Pic, 1954								
*Pseudocistela* Crotch, 1874		X	X	X	X	X	X	X
*Pseudohymenalia* Novák, 2008				X		X		
*Viriathus* Fairmaire, 1902					X			
**Mycetocharina Gistel, 1848**								
*Caulostena* Fairmaire, 1896					X			
*Cylindrothorus* Solier, 1843					X			
SGCylindrothorus Solier, 1843					X			
= *Othelecta* Pascoe, 1866								
SGMicrothelecta Pic, 1952					X			
*Hymenochara* Campbell, 1978		X						
*Labetis* C.O. Waterhouse, 1879								X
*Mycetochara* Guérin-Méneville, 1827		X		X				
SGErnocharis C.G. Thomson, 1859		X		X				
= *Stigmatoma* LeConte, 1862								
SGMycetochara Guérin-Méneville, 1827		X		X				
= *Mycetophila* Gyllenhal, 1810								
= *Mycetocharis* Gyllenhal, 1827								
= *Mycetochares* Latreille, 1829								
= *Bolithophilus* Gistel, 1832								
SGOculochara Novák, 2020				X				
SGPterna Reitter, 1884				X				
†*Mycetocharoides* Schaufuss, 1889								
**Xystropodina Solier, 1835**								
*Anamphidora* Casey, 1924		X						
*Cteisa* Solier, 1835			X					
*Dasytoxystropus* Pic, 1921			X					
*Erxias* Champion, 1888			X					
*Lystronychus* Latreille, 1829		X	X					
SGLystronychus Latreille, 1829		X	X					
SGXystronia Solier, 1835			X					
*Microprostenus* Pic, 1921			X					
*Prostenus* Klug, 1829			X					
= *Mecocerus* Solier, 1835								
*Scotobiopsis* Brèthes, 1910			X					
*Tucumana* Gebien, 1911			X					
= *Eustenia* Fairmaire, 1905								
*Xystropus* Solier, 1835			X					
**Alleculini *incertae sedis***								
*Omedes* Broun, 1893							X	
*Xylochus* Broun, 1880							X	
*Zomedes* Watt, 1992							X	
**Cteniopodini Solier, 1835**								
†*Amberophlus* Novák & Háva, 2019								
*Balassogloa* Semenov, 1891				X				
*Cistelina* Seidlitz, 1896				X		X		
*Cistelomorpha* Redtenbacher, 1868				X		X		
*Cnecosochara* Reitter, 1913				X				
*Cteniopinus* Seidlitz, 1896				X		X		
SGCteniopinus Seidlitz, 1896				X		X		
SGLechinius Blair, 1922						X		
= *Lechinius* Borchmann, 1930								
*Cteniopus* Solier, 1835				X				
SGCtenioposomus Reitter, 1906				X				
SGCteniopus Solier, 1835				X				
= *Cistela* Fabricius, 1775								
= *Cistella* Gistel, 1848								
= *Telacis* Poey, 1854								
= *Sarandonyx* Gozis, 1881								
SGRhinobarus Reitter, 1906				X				
*Diastanus* Fairmaire, 1902					X			
*Falsomophlus* Pic, 1925						X		
*Gastrhaema* Jacquelin du Val, 1863				X				
*Heliomophlus* Reitter, 1906				X				
*Heliostrhaema* Reitter, 1890				X				
*Heliotaurus* Mulsant, 1856				X	X			
SGAtlasotaurus Bouyon, 2011				X				
SGHeliotaurus Mulsant, 1856				X	X			
SGJulogenius Reitter, 1906				X				
*Holdhausia* Reitter, 1906				X				
*Hypocistela* Bates, 1879				X				
*Megischia* Solier, 1835				X				
*Megischina* Reitter, 1906				X				
*Nesotaurus* Fairmaire, 1896					X			
*Omophlina* Reitter, 1890				X				
*Omophlus* Dejean, 1834				X				
SGEuomophlus Iablokoff-Khnzorian, 1983				X				
SGMicromophlus Znojko, 1950				X				
SGOdontomophlus Seidlitz, 1896				X				
= *Pleuromophlus* Reitter, 1906								
SGOmophlus Dejean, 1834				X				
SGPaurodontomophlus Muche, 1979				X				
SGPhibalus Gistel, 1856				X				
= *Paromophlus* Iablokoff-Khnzorian, 1983								
*Petria* Semenov, 1894				X				
†*Platycteniopus* Chang, Nabozhenko, Pu, Xu, Jia, Li, 2016								
*Podonta* Solier, 1835				X				
= *Pododonta* Agassiz, 1846								
*Podontinus* Seidlitz, 1896				X				
*Proctenius* Reitter, 1890				X				
†*Sinocistela* Zhang, 1989								
*Stenerophlina* Reitter, 1906				X				
*Steneryx* Reitter, 1890				X				
*Tripolicryptus* Strand, 1929				X				
= *Brachycryptus* Quedenfeldt, 1891								
**Alleculinae *incertae sedis***								
*Alogistopsis* Borchmann, 1943					X			
*Amorphopoda* Fåhraeus, 1870					X			
*Apalmia* Fairmaire, 1896						X		
*Aptericula* Borchmann, 1937					X			
*Asticostena* Fairmaire, 1897						X		
*Bancocistela* Pic, 1947					X			
*Bearnicistela* Pic, 1909						X		
*Borchmannius* Bousquet & Bouchard, 2015					X			
= *Glyptothorax* Borchmann, 1937								
*Borneocistela* Pic, 1922						X		
*Bratyna* Westwood, 1875					X			
= *Morocaulus* Fairmaire, 1899								
*Buxela* Fairmaire, 1894						X		
*Cistelodema* Borchmann, 1932						X		
*Compsocula* Fairmaire, 1898					X			
*Costallecula* Pic, 1954					X			
*Dioxycula* Fairmaire, 1896						X		
*Ectatocera* Fåhraeus, 1870					X			
*Ectenostoma* Fåhraeus, 1870					X			
*Eutrapelodes* Borchmann, 1929					X			
*Falsopsilonycha* Pic, 1930					X			
*Flabellalogista* Pic, 1954					X			
*Helopsallecula* Pic, 1936					X			
*Homoropsis* Fairmaire, 1886					X			
*Hovacula* Fairmaire, 1898					X			
*Idatius* Fairmaire, 1906					X			
*Isomiropsis* Borchmann, 1942					X			
*Lagriallecula* Pic, 1920					X			
*Macrocistela* Pic, 1941					X			
*Mayidicistela* Pic, 1954					X			
*Microamarygmus* Pic, 1915						X		
*Mimocistela* Borchmann, 1938					X			
*Omolepta* Fåhraeus, 1870					X			
*Pseudomorocaulus* Pic, 1915					X			
*Rhipidonyx* Reitter, 1876						X		
*Seydelicistela* Pic, 1954					X			
*Stenerula* Fairmaire, 1875					X			
*Stenogenomorpha* Pic, 1919					X			
*Strongyallecula* Pic, 1955					X			
**Diaperinae Latreille, 1802**								
**Crypticini Brullé, 1832**								
*Apteroseriscius* Koch, 1950					X			
*Araeopselaphus* Gebien, 1921					X			
*Capicrypticus* Koch, 1950					X			
*Cechenosternum* Gebien, 1921				X	X	X		
*Crypticus* Latreille, 1816				X				
SGCrypticopsis Antoine, 1945				X				
SGCrypticus Latreille, 1816				X				
SGPlatycrypticus Español, 1952				X				
= *Ulomoides* Escalera, 1927								
SGSeriscius Motschulsky, 1845				X				
*Ellipsodes* Wollaston, 1854			*X*	X	X	X	X	X
SGAnthrenopsis Koch, 1950			*X*	X	X	X	X	X
SGEllipsodes Wollaston, 1854				X				
*Gondwanocrypticus* Español, 1955		X	X					
*Lamprocrypticus* Español, 1950				X				
*Lineocrypticus* Koch, 1950					X			
*Microcrypticus* Gebien, 1921					X	X		
SGCrypticocatops Kaszab, 1975						X		
SGMicrocrypticus Gebien, 1921					X			
*Myrmecocatops* Wasmann, 1897					X			
*Oochrotus* P.H. Lucas, 1852				X				
*Poecilocrypticus* Gebien, 1928		X	X					
*Pseudoseriscius* Español, 1950				X	X			
SGAustraloseriscius Koch, 1950					X			
SGPseudoseriscius Español, 1950				X				
**Diaperini Latreille, 1802**								
**Adelinina LeConte, 1862**								
*Adelina* Dejean, 1835		X	X	X		X	X	
= *Doliema* Pascoe, 1860								
= *Schedarosus* Reitter, 1876								
*Alphitophagus* Stephens, 1832		*X*		X		X	*X*	
= *Phyletes* Redtenbacher, 1845								
*Arabcynaeus* Schawaller, 2009				X				
*Cynaeus* LeConte, 1862		X		*X*		*X*		
*Doliodesmus* Spilman, 1967		X						
*Doliopines* Horn, 1894		X						
*Gnatocerus* Thunberg, 1814		X	X	X	X	X	*X*	
SGEchocerus Horn, 1870		X	X	X	X	X		
SGGnatocerus Thunberg, 1814		X	X	X	X	X	*X*	
= *Cerandria* Dejean, 1834								
= *Gnathocerus* Agassiz, 1846								
= *Sicinus* Champion, 1886								
*Iccius* Champion, 1886		X	X					
*Mophis* Champion, 1886			X					
*Neoplateia* Marcuzzi, 1986			X					
*Palembomimus* Matthews & Lawrence, 2005							X	
*Sitophagus* Mulsant, 1854		X	X					
**Diaperina Latreille, 1802**								
*Basides* Motschulsky, 1873				X		X	X	
= *Ischnodactylus* Chevrolat, 1877								
*Ceropria* Laporte & Brullé, 1831			*X*	X	X	X	X	
= *Epilampus* Dejean, 1834								
= *Epilamprus* Gistel, 1848								
= *Dictysomorphus* Pic, 1921								
*Cissides* Chatanay, 1915					X			
*Coelopleurum* Gebien, 1921					X			
*Cosmonota* Blanchard, 1842			X					
*Cyclobiomorphus* Pic, 1916						X		
*Cyclobium* Pic, 1916						X		
*Diaperis* Geoffroy, 1762		X	X	X		X		
= *Allophasia* Pascoe, 1871								
*Espagnolina* Kaszab, 1965						X		
*Exapinaeus* Pascoe, 1882			X					
*Falsocosmonota* Kaszab, 1962						X		
*Gargilius* Fairmaire, 1891					X			
*Gressittiola* Kaszab, 1955							X	
*Heterophylus* Klug, 1833					X			
= *Heterophyllus* Gemminger, 1870								
*Hoplaspis* Motschulsky, 1858						X		
*Lelegeis* Champion, 1886			X					
*Liodema* Horn, 1870		X	X					
*Louwerensia* Kaszab, 1964							X	
*Loxostethus* Triplehorn, 1962			X					
*Neomida* Latreille, 1829		X	X	X		X		
= *Oplocephala* Laporte & Brullé, 1831								
= *Arrhenoplita* W. Kirby, 1837								
= *Hoplocephala* Agassiz, 1846								
= *Evoplus* LeConte, 1866								
*Paniasis* Champion, 1886			X					
= *Pseudapsida* Kulzer, 1961								
*Pentaphyllus* Dejean, 1821		X	X	X	X	X	X	X
= *Iphicorynus* Jacquelin du Val, 1861								
*Phayllus* Champion, 1886			X					
*Platydema* Laporte & Brullé, 1831		X	X	X	X	X	X	X
= *Typhobia* Pascoe, 1869								
= *Histeropsis* Chevrolat, 1878								
= *Anisocara* Gebien, 1925								
*Platydemoides* Kaszab, 1980						X		
*Pseudobasides* Pic, 1916						X		
*Saptine* Champion, 1886			X					
*Sciophagus* Sharp, 1885							X	X
= *Pachycerus* Montrouzier, 1860								
*Stenoscapha* Bates, 1873			X					
*Stomylus* Fåhraeus, 1870					X			
= *Pselaphidion* Gebien, 1921								
*Ulomoides* Blackburn, 1888			*X*	X		X	X	X
= *Palembus* Casey, 1891								
= *Martianus* Fairmaire, 1893								
= *Tenebriomimus* Kolbe, 1901								
= *Phayllidius* Gebien, 1922								
*Yamatotakeru* Ando, 2015				X		X		
**Diaperini *incertae sedis***								
*Pelleas* Bates, 1872				X				
**Ectychini Doyen, Matthews & Lawrence, 1990**								
*Ectyche* Pascoe, 1869							X	
*Micrectyche* Bates, 1873							X	
**Gnathidiini Gebien, 1921**								
**Anopidiina Jeannel & Paulian, 1945**								
*Anopidium* Jeannel & Paulian, 1945					X			
*Caecophloeus* Dajoz, 1972			X					
*Cryptozoon* Schaufuss, 1882			X					
*Mauritianopidium* Dajoz, 1977					X			
*Menimopsis* Champion, 1896			X					
= *Caecomenimopsis* Kaszab, 1970								
*Nanocaecus* Schawaller & Purchart, 2012					X			
*Neanopidium* Dajoz, 1975			X					
*Paralyreus* Grouvelle, 1918					X			
*Paranopidium* Dajoz, 1974					X			
*Peyrierasia* Dajoz, 1975					X			
*Prototyrtaeus* Spiessberger & Ivie, 2020			X					
*Pseudanopidium* Dajoz, 1974					X			
*Sphaerognathium* Dajoz, 1975			X					
*Tyrtaeus* Champion, 1913			X		X		X	
**Gnathidiina Gebien, 1921**								
*Anommabates* Koch, 1956					X			
*Caecochares* Koch, 1956					X			
*Enanea* Lewis, 1894				X		X		
*Gnathidium* Gebien, 1921					X			
*Menimus* Sharp, 1876				X		X	X	X
SGMenimus Sharp, 1876				X		X	X	X
= *Ceramba* Fauvel, 1904								
= *Paita* Fauvel, 1904								
= *Microcilibe* Carter, 1919								
= *Tjikoraia* Pic, 1921								
= *Neomenimus* Kaszab, 1939								
SGSinomenimus G.S. Medvedev, 2007						X		
*Micropeneta* Pic, 1921						X		
= *Menimoides* Kaszab, 1946								
*Pseudoenanea* Pic, 1924						X		
*Sakaiomenimus* Ando, 2003				X				
*Szentivanya* Kaszab, 1958							X	
*Taiwanomenimus* Masumoto, Akita & Lee, 2019						X		
**Gnathidiini *incertae sedis***								
*Betschia* Dajoz, 1980					X			
*Mireanopidium* Dajoz, 1977					X			
**Hyociini G.S. Medvedev & Lawrence, 1982**								
**Brittonina G.S. Medvedev & Lawrence, 1986**								
*Brittona* G.S. Medvedev & Lawrence, 1986							X	
*Magela* G.S. Medvedev & Lawrence, 1986							X	
**Hyociina G.S. Medvedev & Lawrence, 1982**								
*Csiro* G.S. Medvedev & Lawrence, 1984							X	
SGCsiro G.S. Medvedev & Lawrence, 1984							X	
SGMillstreamia G.S. Medvedev & Lawrence, 1984							X	
*Hyocis* Pascoe, 1866							X	
SGHyocis Pascoe, 1866							X	
SGNannohyocis G.S. Medvedev & Lawrence, 1983							X	
SGNeohyocis G.S. Medvedev & Lawrence, 1983							X	
*Parahyocis* Kaszab, 1955							X	X
**Uptonina G.S. Medvedev & Lawrence, 1986**								
*Uptona* G.S. Medvedev & Lawrence, 1986							X	
**Hypophlaeini Billberg, 1820**								
*Cheilopoma* Murray, 1867					X			
*Corticeus* Piller & Mitterpacher, 1783		X	X	X	X	X	X	X
*SG Alienophloeus* Bremer, 2018			X					
SGCorticeus Piller & Mitterpacher, 1783		X	X	X	X	X		X
= *Hypophlaeus* Fabricius, 1790								
= *Paraphloeus* Seidlitz, 1894								
= *Syncolydium* Kolbe, 1897								
SGMetacorticeus Bremer & Lillig, 2017						X	X	
SGNeglectophloeus Bremer & Lillig, 2017			X			X	X	X
SGPogonophloeus Bremer, 1998		X		X	X	X		
SGSeorsophloeus Bremer, 1998					X	X		
SGStenophloeus Blair, 1921			X		X	X	X	
= *Cnemophloeus* Bremer, 1998								
SGTylophloeus Bremer, 1998		X	X	X		X		
*Ischnarthron* Gebien, 1921					X			
*Myonophloeus* Bremer & Lillig, 2017			X					
*Pogonoxenus* Wasmann, 1899					X			
*Typhlophloeus* Jeannel & Paulian, 1945				X	X			
**Leiochrinini Lewis, 1894**								
*Ades* Guérin-Méneville, 1857				X	X	X	X	X
= *Leiochrodes* Westwood, 1883								
*Crypsis* C.O. Waterhouse, 1877				X		X		
= *Leiochrotina* Westwood, 1883								
*Derispia* Lewis, 1894				X		X	X	
*Derispiella* Kaszab, 1961						X		
*Derispiola* Kaszab, 1946						X		
*Leiochrinus* Westwood, 1883				X		X	X	
*Leiochrodinus* Kaszab, 1961						X		
*Leiochrodontes* Kaszab, 1946						X		
*Leiochrota* Westwood, 1883						X		
*Pimplema* Pascoe, 1887						X	X	
= *Hades* J. Thomson, 1860								
*Stethotrypes* Gebien, 1914						X		
= *Leichrodomorphus* Pic, 1921								
**Myrmechixenini Jacquelin du Val, 1858**								
*Myrmechixenus* Chevrolat, 1835		*X*		X	X	X	*X*	
= *Myrmecoxenus* Märkel, 1844								
= *Myrmechoxenus* Aubé, 1850								
**Phaleriini Blanchard, 1845**								
*Emypsara* Pascoe, 1866				X				
= *Callicomus* Motschulsky, 1860								
= *Obenbergeria* Strand, 1929								
*Halammobia* Semenov, 1901				X				
*Pachyphaleria* Gebien, 1920					X			
*Paranemia* Heyden, 1892				X				
= *Taklamakania* Ferrer & Yvinec, 2004								
= *Taclamacanius* Ferrer & Yvinec, 2005								
*Phaleria* Latreille, 1802		X	X	X	X	X	*X*	
SGEpiphaleria Lewis, 1894				X	X	X	X	
SGEremophaleria Español, 1951				X				
SGNeophaleria Español, 1963					X			
SGPhaleria Latreille, 1802		X	X	X			*X*	
= *Uria* Gistel, 1848								
= *Sepedonastes* Gistel, 1856								
= *Halophalerus* Crotch, 1874								
= *Phalerisida* Kulzer, 1959								
= *Atahualpina* Español, 1960								
*Phaleromela* Reitter, 1916		X		X				
*Phtora* Germar, 1836				X	X			
SGClypeophtora F. Soldati & L. Soldati, 2003				X				
SGPhtora Germar, 1836				X	X			
= *Cataphronetis* P.H. Lucas, 1846								
= *Pseudostene* Wollaston, 1861								
= *Phthora* Gemminger, 1870								
**Scaphidemini Reitter, 1922**								
*Basanus* Lacordaire, 1859				X		X		
*Laoscapha* Schawaller, 2016						X		
†*Palaeobasanus* Nabozhenko & Kirejtshuk, 2020								
*Pseudoscaphidema* Pic, 1926				X				
*Scaphidema* Redtenbacher, 1848		X		X		X		
= *Nelites* LeConte, 1850								
= *Microbasanus* Pic, 1921								
*Spiloscapha* Bates, 1873				X		X	X	
**Trachyscelini Blanchard, 1845**								
*Macrotrachyscelis* Pic, 1925					X			
*Taiwanotrachyscelis* Masumoto, Akita & Lee, 2012						X		
*Trachyscelis* Latreille, 1809		*X*	*X*	X	X	X	X	
**Diaperinae *incertae sedis***								
*Triplehornia* Matthews & Lawrence, 2005							X	
**Stenochiinae W. Kirby, 1837**								
**Cnodalonini Oken, 1843**								
*Acanthobas* Gebien, 1928			X					
*Acanthocamaria* Gebien, 1919			X					
*Achariotheca* Kaszab, 1970							X	
*Achrostus* Fairmaire, 1891					X			
*Actanorie* Bates, 1879					X			
= *Callimaria* Fairmaire, 1888								
*Aesthetus* C.O. Waterhouse, 1890			X					
*Agymnonyx* Gebien, 1921							X	
*Ahexaroptrum* Kaszab, 1960						X		
*Ainu* Lewis, 1894				X		X		
SGAinu Lewis, 1894				X		X		
SGErulipus Fairmaire, 1903						X		
*Alcyonotus* Pascoe, 1882					X			
= *Adonicus* Fairmaire, 1891								
= *Sterces* Champion, 1891								
*Alobates* Motschulsky, 1872		X						
*Amarsenes* Bates, 1879					X			
*Amenophis* J. Thomson, 1858					X			
SGAmenophis J. Thomson, 1858					X			
= *Hemerobates* Kolbe, 1884								
= *Praostetha* Fairmaire, 1897								
= *Pseudamenophis* Pic, 1916								
SGDeriles Motschulsky, 1872					X			
= *Anadischidus* Kolbe, 1897								
*Anachayus* Bouchard & Bousquet, **nom. nov.**					X			
= *Chatanayus* Ardoin, 1957								
*Andocamaria* Masumoto, 1993						X		
*Androsus* Gebien, 1921						X	X	
*Anisophaedis* Ando, 1993						X		
*Annamosdara* Kaszab, 1941						X		
*Apsida* Lacordaire, 1859		X	X					
= *Hapsida* Gemminger, 1870								
*Aptereucyrtus* Gebien, 1922						X		
*Apterobrachys* Kaszab, 1986							X	
*Apteromaia* Kulzer, 1952						X		
*Apteromerus* Blair, 1928							X	X
*Apterophenus* Gebien, 1921							X	X
*Apterotheca* Gebien, 1921							X	
= *Austropeus* Carter, 1924								
= *Caxtonana* Buck, 1960								
*Argobrachium* Fairmaire, 1899					X			
= *Aphelus* Gebien, 1921								
*Argutiolana* Robiche, 2001					X			
= *Wahlbergylium* Ferrer, 2011								
*Artactes* Pascoe, 1868						X		
= *Macroartactes* Pic, 1924								
*Asbolodes* Fairmaire, 1892						X		
*Asbolodomimus* Pic, 1921						X		
= *Allopezus* Gebien, 1922								
*Asididius* Fairmaire, 1869					X			
*Asidobothris* Fairmaire, 1886					X			
*Asopidiopsis* Kaszab, 1955								X
*Asopis* Haag-Rutenberg, 1878								X
*Astathmetus* Bates, 1874			X					
*Augolesthus* Motschulsky, 1872						X		
= *Chrysomaia* Kulzer, 1952								
*Baratus* Fairmaire, 1897						X		
*Becvarius* Masumoto, 1998						X		
*Biroum* Kaszab, 1956						X		
*Blapida* Perty, 1830			X					
= *Rhyssochiton* Gray, 1831								
= *Metonites* Gistel, 1848								
*Borneocamaria* Pic, 1917						X		
= *Homoeogenus* C.O. Waterhouse, 1882								
= *Homoeocamaria* Blair, 1919								
= *Krollus* R. Lucas, 1920								
*Borneosphaerotus* Grimm, 2015						X		
*Borneosphena* Purchart & Grimm, 2016						X		
*Borneosynopticus* Grimm, 2015						X		
*Bothynocara* Gebien, 1928			X					
*Bothynocephalus* Doyen, 1988			X					
*Bradymerus* Perroud & Montrouzier, 1865				X	X	X	X	X
= *Isostira* Pascoe, 1870								
= *Bradynocerus* Fairmaire, 1883								
= *Pseudobradymerus* Pic, 1926								
= *Planibates* Kaszab, 1939								
*Bradysphaerotus* Kaszab, 1986							X	
*Brasilius* Gebien, 1928			X					
*Brosimapsida* Ferrer & Ødegaard, 2005			X					
*Byzacnus* Pascoe, 1866					X			
*Calabosca* Fairmaire, 1894						X		
= *Ascalabus* Fairmaire, 1893								
*Calydonella* Doyen, 1995			X					
*Calydoniomorpha* Pic, 1917			X					
*Calydonis* Pascoe, 1882			X					
*Camaria* Lepeletier & Audinet-Serville, 1828			X					
= *Truncatocamaria* Pic, 1922								
*Camarimena* Motschulsky, 1863						X		
= *Sinopium* Pascoe, 1866								
= *Espitomorphus* Pic, 1921								
*Camariocropterum* Pic, 1920			X					
*Camariodes* Fairmaire, 1869					X			
= *Tinophyllus* Fairmaire, 1869								
*Camariomorpha* Pic, 1915						X		
= *Methistamena* Gebien, 1919								
*Campolene* Pascoe, 1863							X	
*Campsia* Lepeletier & Audinet-Serville, 1828			X					
SGBlapidocampsia Pic, 1919			X					
SGCampsia Lepeletier & Audinet-Serville, 1828			X					
= *Celox* Gistel, 1848								
*Campsiomorpha* Pic, 1917				X		X		
*Camptobrachys* Kaszab, 1941						X		
*Carabelops* Fairmaire, 1899					X			
= Agraecus Fairmaire, 1900								
*Caracasa* Pic, 1921			X					
*Cataphanus* Gebien, 1921							X	
*Catapiestus* Perty, 1831						X		
= *Plateia* Laporte, 1840								
*Celebesa* Pic, 1921							X	
*Cephalothydemus* Pic, 1923						X		
*Cerandrosus* Gebien, 1921							X	
*Cerocamptus* Gebien, 1919						X		
*Chaetopsia* Gebien, 1925						X		
*Chalcocyclus* Fairmaire, 1884					X			
*Chalcopauliana* Ardoin, 1961					X			
*Charianus* Bates, 1879					X			
*Chariotheca* Pascoe, 1860							X	X
= *Chariothes* Carter, 1914								
*Chemolanus* Bates, 1879					X			
*Chlorocamma* Bates, 1873							X	
*Choastes* Champion, 1893			X					
= *Choaspes* Champion, 1885								
*Chrysopeplus* Gebien, 1942							X	
= *Leiopeplus* Broun, 1893								
*Cibdelis* Mannerheim, 1843		X						
= *Scotera* Motschulsky, 1845								
*Cleomis* Fairmaire, 1892						X		
= *Pseudeucyrtus* Pic, 1916								
= *Oyanus* Pic, 1921								
*Cnephalura* Doyen, 1988			X					
*Cnodalon* Latreille, 1797			X					
= *Cnodalum* Agassiz, 1846								
*Coelocnemis* Mannerheim, 1843		X						
*Coelometopus* Solier, 1848				X				
*Cophodema* Gebien, 1943			X					
= *Cophosoma* Gebien, 1928								
*Cryptobates* Fairmaire, 1882						X		
*Cryptobatoides* Kaszab, 1941						X		
*Cryptobrachys* Kaszab, 1941						X		
*Cryptostenophanes* Kaszab, 1941						X		
*Csikiola* Kaszab, 1955								X
*Cuemus* Bouchard, 2000							X	
*Cybopiestes* Reitter, 1917				X				
*Cyclonesus* Fairmaire, 1896						X		
*Cyrtosoma* Perty, 1830			X					
*Cyrtotyche* Pascoe, 1866					X			
*Cyrtotyctus* Kolbe, 1897					X			
*Damatris* Laporte, 1840					X			
= *Hybonotus* Dejean, 1834								
= *Malacova* Fairmaire, 1898								
*Danodema* Gebien, 1925					X			
*Dechiustes* Blair, 1940								X
*Dentatoploedipus* Kaszab, 1984						X		
*Deplanchesia* Fauvel, 1860			X					
*Derosphaerus* J. Thomson, 1858				X	X	X	X	
= *Euphron* Dejean, 1834								
= *Encyalesthus* Motschulsky, 1860								
= *Pachyurgus* LeConte, 1862								
= *Cholipus* Pascoe, 1866								
= *Notiolesthus* Motschulsky, 1872								
= *Neandrosus* Pic, 1921								
= *Falsoencyalesthus* Pic, 1923								
*Diachoriops* Ando, 2020				X		X	X	X
= *Schizomma* Gebien, 1921								
*Dinomus* Brême, 1842			X					
*Diopethes* Pascoe, 1882			X					
*Dioscoridemus* Koch, 1970					X			
*Drocleana* Bates, 1879					X			
*Eccoptostoma* Gebien, 1913					X			
= *Ogoueum* Pic, 1923								
*Ectomopsis* Fairmaire, 1905			X					
*Elomosda* Bates, 1870			X					
*Epicalla* Lacordaire, 1859			X					
*Episopus* Bates, 1873							X	
*Eremobatodes* Gebien, 1943					X			
= *Eremobates* Gebien, 1921								
*Espites* Pascoe, 1882							X	
*Eucyrtus* Lacordaire, 1859						X		
= *Microeucyrtus* Pic, 1926								
*Euhemicera* Ando, 1996						X		
*Euphloeus* Pascoe, 1887						X		
*Eutelonodolinus* Robiche, 2007					X			
*Eutelonotus* Fairmaire, 1902					X			
= *Eutelus* Solier, 1843								
= *Nodotelus* Koch, 1950								
*Euthysternum* Chatanay, 1915					X			
*Exocolena* Gebien, 1914						X		
*Falsandrosus* Kaszab, 1980						X		
*Falsobates* Kaszab, 1941						X		
*Falsobrachys* Kulzer, 1954						X		
*Falsocamaria* Pic, 1917				X		X		
= *Eucamaria* Gebien, 1919								
*Falsocamariodes* Ardoin, 1956					X			
*Falsodiopethes* Pic, 1924			X					
*Falsonannocerus* Pic, 1947					X			
*Falsoperichilus* Pic, 1920					X			
*Falsozotypus* Kaszab, 1980						X		
*Foochounus* Pic, 1921						X		
= *Chariophenus* Blair, 1929								
= *Anobriomaia* Kaszab, 1941								
= *Microcameria* Ren, 1998								
*Gaurobates* Gebien, 1928			X					
*Gauromaia* Pascoe, 1866						X		
SGFalsogauromaia Pic, 1921						X		
SGGauromaia Pascoe, 1866						X		
= *Cephaleucyrtus* Pic, 1923								
*Gebienella* Kaszab, 1941						X		
*Gebienocamaria* Masumoto, 1993						X		
*Gigantopigeus* Kaszab, 1984						X		
*Girardocamaria* Masumoto, 1993						X		
*Glyptotus* LeConte, 1858		X	X					
*Gnesis* Pascoe, 1866						X		
= *Tromosternus* Harold, 1876								
*Gonespites* Gebien, 1921							X	
*Gonospa* Champion, 1886			X					
*Graptopezus* Gebien, 1921							X	
*Haplandrus* LeConte, 1862		X						
*Haporema* Fairmaire, 1892					X			
*Hegemona* Laporte, 1840			X					
= *Eucamptus* Germar, 1842								
= *Eusarca* Chevrolat, 1845								
*Heliofugus* Guérin-Méneville, 1831			X					
SGCollariheliofugus Freude, 1960			X					
SGHeliofugus Guérin-Méneville, 1831			X					
= *Heliosteres* Hope, 1841								
= *Heliophygus* Agassiz, 1846								
= *Euschatia* Solier, 1851								
SGInscutoheliofugus Freude, 1960			X					
SGRugosiheliofugus Freude, 1960			X					
*Hemicera* Laporte & Brullé, 1831						X		
SGHemicera Laporte & Brullé, 1831						X		
= *Chrysolinoides* Jolivet, 1951								
SGNanohemicera Pic, 1923						X		
*Hemimmedia* Gebien, 1928			X					
*Hesiodus* Champion, 1885			X					
*Hexarhopalus* Fairmaire, 1891				X		X		
SGHexarhopalus Fairmaire, 1891				X		X		
= *Hexarhoptrum* Fairmaire, 1894								
= *Laosocryptobates* Pic, 1928								
= *Apteroleprocaulus* Kaszab, 1983								
SGLeprocaulus Fairmaire, 1896						X		
= *Pseudocaelophus* Pic, 1922								
= *Pseudoderosphaerus* Pic, 1922								
*Hicetaon* Champion, 1885			X					
*Holobrachys* Fairmaire, 1869					X			
*Hoploedipinus* Kaszab, 1984						X		
*Hoploedipus* Fairmaire, 1898						X		
*Hyboproctus* Kolbe, 1897					X			
*Hydissus* Pascoe, 1869							X	
= *Hydisus* Scudder, 1882								
*Hypaulax* Bates, 1868							X	
= *Chileone* Bates, 1868								
*Hypocalis* Dejean, 1834					X			
*Hypovinsonia* Ardoin, 1961					X			
*Ilus* Champion, 1885			X					
*Immedia* Pascoe, 1882			X					
*Iphthiminus* Spilman, 1973		X		X				
*Iphthimulus* Reitter, 1920				X				
*Irianobates* Kaszab, 1986							X	
*Isaminas* Champion, 1887			X					
= *Pteroglymmius* Gebien, 1928								
*Isicerdes* Champion, 1885			X					
*Isopus* Montrouzier, 1860							X	
*Kabakoviella* Kaszab, 1980						X		
*Kaszaba* Matthews & Doyen, 1989							X	
*Lenkous* Kaszab, 1973			X					
*Lepidocaulinus* Schawaller, Masumoto & Merkl, 2013						X		
*Leprocaulinus* Kaszab, 1982						X		
= *Pigeocaulinus* Kaszab, 1984								
*Lomocnemis* Gebien, 1921							X	
*Lordodera* Gebien, 1921					X			
*Lycidioides* Ando, 2003						X		
*Macropachylesthus* Pic, 1923						X		
*Macrostethus* Wollaston, 1854				X				
*Mahena* Gebien, 1922					X			
*Malayaplamius* Masumoto, 1986						X		
*Malaysphena* Bečvář & Purchart, 2008						X		
*Maracia* Gebien, 1919			X					
*Mariepskopia* Schawaller, 2012					X			
*Mechanetes* C.O. Waterhouse, 1887						X		
= *Diabolicobates* Pic, 1930								
*Melobrachys* Kaszab, 1960						X		
*Menandris* Haag-Rutenberg, 1878								X
*Menephilus* Mulsant, 1854				X		X		X
*Merinus* LeConte, 1862		X						
*Metisopus* Bates, 1873							X	X
*Micreuphlaeus* Fairmaire, 1897						X		
*Microbradymerus* Schawaller, 1999						X		
*Micromenandris* Kaszab, 1955								X
*Microphenus* Gebien, 1921							X	
*Microsphaerotus* Pic, 1928						X		
*Misolampidius* Solsky, 1875				X		X		
= *Ptilonix* Allard, 1877								
*Misolampomorphus* Kaszab, 1941						X		
*Misolampus* Latreille, 1806				X				
*Mitys* Champion, 1885			X					
*Moeon* Champion, 1886			X					
*Mophon* Champion, 1886			X					
*Moromelas* Fairmaire, 1898					X			
*Morphostenophanes* Pic, 1925				X		X		
= *Promorphostenophanes* Kaszab, 1960								
*Mrazius* Pic, 1925			X					
*Mylaris* Pallas, 1781			X					
= *Iphthinus* Dejean, 1834								
= *Cecrops* Gistel, 1834								
= *Nyctobates* Guérin-Méneville, 1834								
= *Iphthimus* Gemminger, 1870								
*Nannalcyon* Koch, 1950					X			
= *Nannocerus* Fairmaire, 1887								
*Necrobioides* Fairmaire, 1882						X		
*Neoplamius* Löbl, Bouchard, Merkl & Bousquet, 2020						X		
*Neoporphyrhyba* Ardoin, 1956					X			
*Neotheca* Carter, 1930							X	
*Nesocyrtosoma* Marcuzzi, 1976			X					
= †*Hesiodobates* Kaszab & Schawaller, 1984								
= *Pachycyrtosoma* Marcuzzi, 1999								
= *Serrania* Garrido, 2003								
*Nesosphaerotus* Gebien, 1921					X			
= *Nesophaerotus* Ardoin, 1962								
*Nuptis* Motschulsky, 1872			X					
*Oeatus* Champion, 1885			X					
*Oectosis* Pascoe, 1869							X	
*Oedemutes* Pascoe, 1860						X		
SGOedemutes Pascoe, 1860						X		
SGTamdaous Pic, 1923						X		
*Oenopion* Champion, 1885		X	X					
*Omolipus* Pascoe, 1860							X	
*Osdara* Walker, 1858						X	X	
SGOsdara Walker, 1858						X		
SGSpinosdara Bouchard & Bousquet, **subgen. nov.**						X	X	
*Osdaroides* Kaszab, 1980						X		
*Osternus* Fairmaire, 1895					X			
SGMicrocalydonis Pic, 1923					X			
SGOsternus Fairmaire, 1895					X			
*Othryoneus* Champion, 1886			X					
*Oxidates* Champion, 1886			X					
*Ozaenimorphus* Fairmaire, 1882					X			
*Ozotypus* Pascoe, 1862						X		
*Pachylesthus* Fairmaire, 1897						X		
*Papuamisolampus* Kaszab, 1986							X	
*Paramisolampidius* Merkl & Masumoto, 2020				X		X		
*Parimmedia* Gebien, 1928			X					
*Paroeatus* Gebien, 1928			X					
*Paulianaria* Bouchard & Bousquet, **gen. nov.**					X			
*Perichilus* Quedenfeldt, 1885					X			
*Periphanodes* Gebien, 1943						X		
= *Periphanes* Fairmaire, 1882								
*Pezomaia* Kulzer, 1952						X		
*Pezophenus* Gebien, 1921							X	
*Phaedis* Pascoe, 1866						X		
= *Pseudeumolpus* Kraatz, 1880								
= *Phaedeucyrtus* Pic, 1916								
= *Microgauromaia* Pic, 1921								
*Phenus* Gebien, 1921							X	
*Phymaeus* Pascoe, 1883						X		
*Picocamaria* Masumoto, 1993						X		
*Pigeostrongylium* Kaszab, 1984						X		
*Pigeus* Gebien, 1919						X		
= *Pseudocamarimena* Pic, 1923								
*Piloxys* Fairmaire, 1895					X			
*Plamius* Fairmaire, 1896				X		X		
= *Cnemandrosus* Gebien, 1927								
*Platycrepis* Lacordaire, 1859						X		
*Poeciltoides* Fairmaire, 1896					X			
*Polopinus* Casey, 1924		X						
*Polposipus* Solier, 1848					X			
= *Pulposipes* Gemminger, 1870								
= *Dysceladus* C.O. Waterhouse, 1875								
*Polypleurus* Eschscholtz, 1831		X						
*Ponapeida* Kulzer, 1957								X
*Porphyrhyba* Fairmaire, 1877					X			
= *Porphyrohyba* Rye, 1879								
= *Hybocaulus* Fairmaire, 1895								
*Postandrosus* Kulzer, 1951						X		
*Priocamaria* Gebien, 1919			X					
*Promethis* Pascoe, 1869				X	X	X	X	X
= *Mederis* Motschulsky, 1872								
= *Pediris* Motschulsky, 1872								
= *Setenis* Motschulsky, 1872								
= *Pseudobates* Fairmaire, 1882								
*Proscorus* Fairmaire, 1901					X			
*Pseudabax* Kraatz, 1880						X		
*Pseudamarsenes* Ardoin, 1955					X			
*Pseudandrosus* Kulzer, 1951						X		
*Pseudhadrus* Kolbe, 1910					X			
= *Paradrus* Jakobson, 1924								
*Pseudimmedia* Kulzer, 1958			X					
*Pseudisopus* Kulzer, 1957								X
*Pseudoblapida* Pic, 1917			X					
SGBlapidocamaria Pic, 1919			X					
SGPseudoblapida Pic, 1917			X					
*Pseudocamaria* Bates, 1879					X			
*Pseudochrysomela* Pic, 1925						X		
*Pseudoderiles* Gebien, 1928			X					
*Pseudonautes* Fairmaire, 1892				X		X		
= *Thydemus* Lewis, 1894								
*Pseudoperichilus* Pic, 1921					X			
*Pseudopigeus* Kaszab, 1984						X		
*Pseudothryoneus* Pic, 1921			X					
*Psydocamaria* Pic, 1923						X		
*Psydomorphus* Pic, 1921						X		
*Psydus* Pascoe, 1868						X		
*Rehumius* Fairmaire, 1893						X		
= *Melobates* Kaszab, 1941								
*Rhopalobates* Fairmaire, 1897						X		
*Robustocamaria* Pic, 1922						X		
= *Neocamaria* Kulzer, 1954								
*Rhophobas* Motschulsky, 1872						X		
*Sadanaria* Ando & Ichiyanagi, 2009						X		
*Saziches* Champion, 1886			X					
*Scotaeus* Hope, 1834						X		
*Scotoderus* Perroud & Montrouzier, 1865							X	X
= *Dechius* Pascoe, 1866								
= *Pelecypalpus* Hinton, 1947								
*Scutopiloxys* Pic, 1924					X			
*Simalura* Gebien, 1914				X		X		
= *Microhemicera* Pic, 1921								
*Sophrobates* Fairmaire, 1889			X					
*Sphaerocaulus* Fairmaire, 1869					X			
*Sphaeromatris* Fairmaire, 1899					X			
*Sphaerotidius* Kaszab, 1941						X		
*Sphaerotus* W. Kirby, 1819			X					
*Spheneuphloeus* Kaszab, 1941						X		
*Sphenolampidius* Kaszab, 1941						X		
*Sphenosdara* Kaszab, 1941						X		
*Spinepicalla* Pic, 1921			X					
*Spinoderosphaerus* Pic, 1922						X		
= *Spinogauromaia* Pic, 1922								
*Srilanka* Kaszab, 1980						X		
*Steneucyrtus* Fairmaire, 1896						X		
= *Pseudochariotheca* Pic, 1934								
*Stenochinus* Motschulsky, 1860				X	X	X		
= *Dicraeus* Marseul, 1876								
= *Brachypilium* Fairmaire, 1896								
= *Dicraeosis* Gebien, 1911								
*Stenophanes* Solsky, 1876				X				
*Stenothesilea* Kulzer, 1951						X	X	
*Sternomaia* Kulzer, 1952						X		
*Sthenoboea* Champion, 1885			X					
*Strepsius* Fairmaire, 1896					X			
*Styphloeus* Kaszab, 1941						X		
*Suarezius* Fairmaire, 1895					X			
*Sulpiusoma* Ferrer, 2006					X			
*Sundon* Pic, 1923						X		
*Sycophantes* Kirsch, 1866			X					
*Sycophantomorphus* Pic, 1924			X					
*Tabarus* Gebien, 1921							X	
*Taichius* Ando, 1996						X		
*Taiwanomenephilus* Masumoto, 1986						X		
*Tanchirus* Fairmaire, 1897						X		
*Taphrosoma* Kirsch, 1866			X					
= *Orobychus* Pascoe, 1868								
*Taraxides* C.O. Waterhouse, 1876					X			
= *Dischidus* Kolbe, 1886								
*Tearchus* Kraatz, 1880						X		
= *Heteromerotylus* Pic, 1921								
*Teles* Mulsant & Godart, 1876				X				
*Telethrus* Pascoe, 1882			X					
*Telleus* Fairmaire, 1904			X					
*Temnoaphelus* Ferrer, 1988					X			
*Temnophthalmus* Gebien, 1921					X			
*Tenebriocamaria* Pic, 1919			X					
*Tenebriopsis* Gebien, 1928			X					
*Tenesis* Duvivier, 1892					X			
*Tentyriopsis* Gebien, 1928			X					
*Tetragonomenes* Chevrolat, 1878				X		X	X	
= *Tetragonomecus* Rye, 1880								
= *Obriomaia* Gebien, 1927								
= *Falsoaugolesthus* Masumoto, 1993								
*Tetraphyllus* Laporte & Brullé, 1831				X		X		
= *Adeps* Gistel, 1857								
= *Addia* Lewis, 1894								
= *Adepsion* Strand, 1917								
*Thecacerus* Lacordaire, 1859			X					
*Thesilea* Haag-Rutenberg, 1878						X	X	X
*Thettea* Bates, 1879					X			
*Thydemorphus* Pic, 1918						X		
*Tonkinius* Fairmaire, 1903						X		
*Toxocnema* Fåhraeus, 1870					X			
*Trichodamatris* Chatanay, 1915					X			
*Uenomisolampidius* Masumoto, 1996						X		
*Upis* Fabricius, 1792		X		X				
*Xanthobates* Gebien, 1928			X					
*Xantusiella* Kaszab, 1941						X		
*Xenius* Champion, 1886			X					
*Xylopinus* LeConte, 1862		X						
= *Taenobates* Motschulsky, 1872								
*Zabroideus* Fairmaire, 1894				X				
*Zophius* Dejean, 1834					X			
*Zophophilus* Fairmaire, 1881						X	X	
= *Sphenothorax* Gebien, 1906								
= *Teremenes* Carter, 1914								
**Stenochiini W. Kirby, 1837**								
*Agissopterus* Fairmaire, 1884			X					
*Asemogena* Péringuey, 1904					X			
*Azonoderus* Harold, 1879					X			
*Bionesus* Fairmaire, 1879								X
*Bremerianus* Masumoto & Bečvář, 2005						X		
*Cuphotes* Champion, 1887			X					
= *Spheniscus* W. Kirby, 1819								
= *Eucosmus* Gistel, 1848								
= *Phygoscotus* Schulz, 1902								
*Dauresia* Ferrer, 2001					X			
*Dicyrtus* Duponchel, 1844			X					
*Diestesoma* Péringuey, 1904					X			
*Dorelogena* Péringuey, 1904					X			
*Elasmocerella* Strand, 1935			X					
= *Elasmocera* Mäklin, 1867								
*Epiplecta* Mäklin, 1867			X					
*Eucrossoscelis* Nakane, 1963						X		
*Eutherama* Carter, 1914							X	
*Falsocuphotes* Pic, 1918			X					
*Falsonotostrongylium* Kaszab, 1955								X
*Falsostrongylium* Pic, 1915			X					
*Flabellostrongylium* Pic, 1938			X					
*Freudella* Ardoin, 1961					X			
*Genateropa* Bouchard & Bousquet, **nom. nov.**					X			
= *Apterogena* Ardoin, 1962								
*Heterostrongylium* Kaszab, 1977							X	
*Holostrongylium* Kaszab, 1977						X	X	
*Hoplostrongylium* Ardoin, 1965					X			
*Hyperchalca* Fairmaire, 1869					X			
SGHyperchalca Fairmaire, 1869					X			
SGMacrohyperchalca Pic, 1935					X			
*Lophocnemis* Mäklin, 1867						X	X	
= *Pseudostrongylium* Kraatz, 1880								
= *Enganodia* Fairmaire, 1898								
= *Mimothydemus* Pic, 1923								
*Mentes* Champion, 1893			X					
*Microtocerus* Pic, 1918			X					
*Mictopsis* Fairmaire, 1899					X			
*Miotodera* Fairmaire, 1901					X			
= *Cyphelops* Fairmaire, 1901								
*Nodosogylium* Pic, 1951					X			
*Oenomia* Pascoe, 1883			X					
*Oploptera* Chevrolat, 1844			X					
SGOploptera Chevrolat, 1844			X					
= *Otocerus* Mäklin, 1867								
= *Hoploptera* Gemminger, 1870								
SGPlicatocerus Pic, 1918			X					
*Otoceromorphus* Pic, 1915			X					
*Parastrongylium* Kaszab, 1977							X	
*Phyllechus* Bouchard & Bousquet, **gen. nov.**						X		
*Phymatosoma* Laporte & Brullé, 1831						X		
*Platyesthus* Mäklin, 1878			X					
*Poecilesthus* Dejean, 1834			X					
= *Dinax* Gistel, 1848								
= *Diestica* Pascoe, 1868								
*Pseudogena* Fairmaire, 1899					X			
*Pseudotocerus* Champion, 1888			X					
*Psilonesogena* Bates, 1879					X			
= *Asthenopoda* Chatanay, 1915								
*Saitostrongylium* Masumoto, 1996						X		
*Strongylacanthus* Brèthes, 1925			X					
*Strongylium* W. Kirby, 1819		X	X	X	X	X	X	X
SGAfrostrongylium Robiche, 2019					X			
SGStrongylium W. Kirby, 1819		X	X	X	X	X	X	X
= *Stenochia* W. Kirby, 1819								
= *Gentinadis* Laporte, 1840								
= *Saerangodes* Gistel, 1848								
= *Anomoearthrum* Mäklin, 1867								
= *Coelolophus* Mäklin, 1867								
= *Xanthothopeia* Mäklin, 1867								
= *Xanthothopia* Gemminger, 1870								
= *Styrax* Westwood, 1875								
= *Messalia* Pascoe, 1883								
= *Strongyliastrum* Fairmaire, 1894								
= *Eustrongylium* Kolbe, 1895								
= *Allostrongylium* Kolbe, 1896								
= *Ebenolus* Fairmaire, 1897								
= *Zuercheria* Reitter, 1908								
= *Crossoscelis* Gebien, 1914								
= *Notostrongylium* Carter, 1915								
= *Pedostrongylium* Pic, 1916								
= *Falsolophocnemis* Pic, 1917								
= *Gibbostrongylium* Pic, 1917								
= *Microstrongylium* Pic, 1917								
= *Poecilesthostrongylium* Pic, 1918								
= *Reminius* Casey, 1924								
= *Mimogoueum* Pic, 1952								
*Theresea* Pic, 1917						X		
*Uenostrongylium* Masumoto, 1999						X		
**Talanini Champion, 1887 (1883)**								
*Talanus* Jacquelin du Val, 1857			X					
= *Dignamptus* LeConte, 1878								
**Stenochiinae *incertae sedis***								
†*Anthracohelops* Haupt, 1950								
†*Caryosoma* Haupt, 1950								
†*Eodromus* Haupt, 1950								
†*Mimohelops* Haupt, 1950								
†*Parakeleusticus* Haupt, 1950								
†*Pseudohelops* Haupt, 1950								
†*Pyrochalcaspis* Haupt, 1950								
**Tenebrionidae *incertae sedis***								
*Afrohelops* Schawaller, 2012					X			
*Allotriocochabambia* Faúndez, Rider & Carvajal, 2014			X					
= *Cochabambia* Marcuzzi, 1985								
*Ancylopoma* Pascoe, 1871			X					
*Baryscelis* Boisduval, 1835							X	
*Camarothelops* Kolbe, 1910					X			
†*Cretaceites* Wang, 1997								
†*Eoallognosis* Haupt, 1950								
†*Eocallidium* Haupt, 1950								
†*Eohelaeus* Haupt, 1950								
*Erelus* Mulsant & Rey, 1853				X				
*Gnathelops* Gebien, 1922					X			
*Homocyrtus* Dejean, 1834			X					
= *Cyphonotus* Guérin-Méneville, 1831								
= *Schlinkus* R. Lucas, 1920								
*Macrophtalmus* Montrouzier, 1855							X	
= *Macrophthalmata* Strand, 1935								
†*Paropiophorus* Haupt, 1950								
*Physciolagria* Pic, 1930					X			
†*Protoplatycera* Wickham, 1914								
*Pygidiphorus* Mulsant, 1856				X				
†*Rhinohelaeites* Haupt, 1950								
†*Tagenopsis* Heer, 1864								
†*Tenebrionites* Cockerell, 1920								

### List of available genus-group names in Tenebrionidae Latreille, 1802

*Abantiades* Fairmaire, 1894f: 395 [M]. Type species [automatic]: *Abantisaenescens* Fairmaire, 1892, by monotypy. Status: junior synonym of *Diphyrrhynchus* Fairmaire, 1849 in Blaptinae: Opatrini: Heterotarsina. Note: replacement name for *Abantis* Fairmaire, 1892; junior homonym of *Abantiades* Herrich-Schäffer, 1856 [Lepidoptera].

*Abantis* Fairmaire, 1892a: 109 [F]. Type species: *Abantisaenescens* Fairmaire, 1892, by monotypy. Status: junior synonym of *Diphyrrhynchus* Fairmaire, 1849 in Blaptinae: Opatrini: Heterotarsina. Synonymy: Gebien (1938a: 407). Note: junior homonym of *Abantis* Hoppfer, 1855 [Lepidoptera].

*Aberlencus* Iwan, 2002b: 559 [M]. Type species: *Aberlencusangolensis* Iwan, 2002, by original designation. Status: junior synonym of *Angolositus* Koch, 1955 in Blaptinae: Platynotini: Platynotina. Synonymy: Kamiński (2015a: 90).

*Abiga* Guérin-Méneville, 1860: clxxxix [F]. Type species: *Abigahumilis* Guérin-Méneville, 1860, by subsequent designation (Löbl et al. 2008a: 40). Status: junior synonym of *Scelosodis* Solier, 1835 in Pimeliinae: Tentyriini. Synonymy: Guérin-Méneville (1862: 376).

*Abigopsis* Escalera, 1914: 277 [F]. Type species: *Scelosodisustus* Fairmaire, 1879, by subsequent designation (Gebien 1937a: 612). Status: valid genus in Pimeliinae: Tentyriini.

*Ablapsis* Reitter, 1887a: 368 [F]. Type species: *Blaps compressipes* Reitter, 1887, by monotypy. Status: valid genus in Blaptinae: Blaptini: Blaptina.

*Acanthioides* Fairmaire, 1894e: 320 [F]. Type species: *Acanthioidesasperula* Fairmaire, 1894, by monotypy. Status: valid genus in Pimeliinae: Adelostomini.

*Acanthobas* Gebien, 1928: 168, 175 [M]. Type species: *Acanthobasangusticollis* Gebien, 1928, by monotypy. Status: valid genus in Stenochiinae: Cnodalonini.

*Acanthoblaps* Reitter, 1889a: 687 [F]. Type species: *Acanthoblapsdentitibia* Reitter, 1889, by monotypy. Status: junior synonym of *Blaps* Fabricius, 1775 in Blaptinae: Blaptini: Blaptina. Synonymy: Gebien (1910b: 226).

*Acanthocamaria* Gebien, 1919: 26, 31 [F]. Type species: *Acanthocamariabrunneoopaca* Gebien, 1919, by monotypy. Status: valid genus in Stenochiinae: Cnodalonini.

*Acanthomera* Latreille, 1828: 580 [F]. Type species: *Pimeliadentipes* Fabricius, 1787, by monotypy. Status: senior synonym of *Psorodes* Dejean, 1834 in Tenebrioninae: Amarygmini. Note: junior homonym of *Acanthomera* Wiedemann, 1821 [Diptera].

*Acanthomerus* Agassiz, 1846b: 2 [M]. Type species [automatic]: *Pimeliadentipes* Fabricius, 1787, by monotypy. Status: junior synonym of *Psorodes* Dejean, 1834 in Tenebrioninae: Amarygmini. Note: unjustified emendation of *Acanthomera* Latreille, 1828, not in prevailing usage.

*Acanthosternus* Montrouzier, 1860: 289 [M]. Type species: *Acanthosternushalorageos* Montrouzier, 1860, by monotypy. Status: junior synonym of *Diphyrrhynchus* Fairmaire, 1849 in Blaptinae: Opatrini: Heterotarsina. Synonymy: Bates (1872a: 97).

*Acantophorus* Billberg, 1820: 32 [M]. Type species: *Erodiusgibbus* Fabricius, 1775, by subsequent designation (Bouchard and Bousquet 2020b: 7). Status: junior synonym of *Erodius* Fabricius, 1775 in Pimeliinae: Erodiini. Synonymy: Bouchard and Bousquet (2020b: 7).

*Acastus* Péringuey, 1896: 177 [M]. Type species: *Acastusagrestis* Péringuey, 1896, by subsequent designation (Ardoin 1963a: 87). Status: junior synonym of *Gonocnemis* J. Thomson, 1858 in Tenebrioninae: Amarygmini. Synonymy: Gebien (1913: 76).

*Accanthopus* Dejean, 1821: 71 [M]. Type species: **fixed herein** (ICZN 1999, Article 70.3) as *Tenebriovelikensis* Piller & Mitterpacher, 1783, misidentified as *Tenebriocaraboides* Linnaeus, 1758 in the original designation by monotypy in Dejean (1821). Status: valid genus in Tenebrioninae: Helopini: Enoplopodina. Note: the type species “*Blaps caraboides* Germ.” was first established by monotypy; Seidlitz (1895: 681) first noted that *Tenebriocaraboides* Linnaeus of Germar (1817) was identical to *Helopsdentipes* Rossi, 1790, currently a junior synonym of *Tenebriovelikensis* Piller & Mitterpacher, 1783; we follow currently accepted concepts (e.g., Löbl et al. 2008b: 246) and fix the type species according to the requirements of Article 70.3.2 (ICZN 1999); the nominal species *Tenebriocaraboides* Linnaeus, 1758 is a valid species in the genus *Cychrus* Fabricius, 1794 [Coleoptera: Carabidae].

*Acerogria* Borchmann, 1936: 19, 137 [F]. Type species: *Cerogriadohrni* Borchmann, 1911, by original designation. Status: valid genus in Lagriinae: Lagriini: Lagriina.

*Acestophanus* Koch, 1950a: 67 [M]. Type species [automatic]: *Acestuselongatus* Haag-Rutenberg, 1875, by subsequent designation (R. Lucas 1920: 69). Status: valid genus in Pimeliinae: Adelostomini. Note: replacement name for *Acestus* Haag-Rutenberg, 1875.

*Acestus* Haag-Rutenberg, 1875b: 4, 56 [M]. Type species: *Acestuselongatus* Haag-Rutenberg, 1875, by subsequent designation (R. Lucas 1920: 69). Status: senior synonym of *Acestophanus* Koch, 1950 in Pimeliinae: Adelostomini. Note: junior homonym of *Acestus* Leidy, 1851 [Annelida].

*Achaemenes* Bogatchev, 1949: 39 [M]. Type species: *Achaemenesvillosus* Bogatchev, 1949 (= *Thripterabogatchevi* Kwieton, 1982), by original designation. Status: senior synonym of *Bogatshevia* G.S. Medvedev & Iwan, 2006 in Pimeliinae: Pimeliini. Note: junior homonym of *Achaemenes* Stål, 1866 [Hemiptera].

*Achanius* Erichson, 1847a: 118 [M]. Type species: *Achaniusanthicoides* Erichson, 1847, by monotypy. Status: valid genus and subgenus in Pimeliinae: Evaniosomini. Note: transferred from Edrotini (where it was previously placed by Doyen (1994: 500)) by Flores and Aballay (2015: 167).

*Achariotheca* Kaszab, 1970a: 273 [F]. Type species: *Achariothecabaloghi* Kaszab, 1970, by original designation. Status: valid genus in Stenochiinae: Cnodalonini.

*Achora* Pascoe, 1869: 279 [F]. Type species: *Asidaserricollis* Hope, 1843 (= *Cestrinusobscurus* Erichson, 1842), by monotypy. Status: junior synonym of *Isopteron* Hope, 1841 in Lagriinae: Adeliini. Synonymy: Gemminger in Gemminger and Harold (1870: 1929, with *Prionotus* Mulsant & Rey, 1859, a junior synonym of *Isopteron* Hope, 1841); Matthews (1998: 788).

*Achrostus* Fairmaire, 1891b: 256 [M]. Type species: *Achrostusrufonitens* Fairmaire, 1891, by monotypy. Status: valid genus in Stenochiinae: Cnodalonini.

*Achthosus* Pascoe, 1863a: 42 [M]. Type species: *Achthosuswestwoodii* Pascoe, 1863, by monotypy. Status: valid genus in Tenebrioninae: Ulomini.

*Acidia* Illiger, 1804: 79 [F]. Type species [automatic]: *Pimeliareflexa* Fabricius, 1775, by subsequent designation (Latreille 1810: 429). Status: junior synonym of *Akis* Herbst, 1799 in Pimeliinae: Akidini. Note: unjustified emendation of *Akis* Herbst, 1799, not in prevailing usage.

*Acis* Billberg, 1820: 32 [F]. Type species [automatic]: *Pimeliareflexa* Fabricius, 1775, by subsequent designation (Latreille 1810: 429). Status: junior synonym of *Akis* Herbst, 1799 in Pimeliinae: Akidini. Note: unjustified emendation of *Akis* Herbst, 1799, not in prevailing usage.

*Acisba* Dejean, 1834: 185 [F]. Type species: *Tentyriacribrosa* Besser, 1832 (= *Pimeliapunctata* Fabricius, 1798), by subsequent designation (Löbl et al. 2008a: 40). Status: junior synonym of *Pachychila* Eschscholtz, 1831 in Pimeliinae: Tentyriini. Synonymy: Solier (1835b: 288). Note: the original combination of the accepted name of the type species, *Pimeliapunctata* Fabricius, 1798, is a junior primary homonym of *Pimeliapunctata* Thunberg, 1787.

*Acmoeus* Fåhraeus, 1870: 293 [M]. Type species: *Anomalipuselephas* Fåhraeus, 1870, by monotypy. Status: junior synonym of *Anomalipus* Guérin-Méneville, 1831 in Blaptinae: Platynotini: Platynotina. Synonymy: Gebien (1938a: 409).

*Aconobius* Casey, 1895: 617 [M]. Type species: *Conibiosomalaciniata* Casey, 1892, by original designation. Status: valid genus in Blaptinae: Opatrini: Blapstinina.

*Acontodactylus* Desbrochers des Loges, 1894: 5 [M]. Type species: *Cossyphusminutissimus* Laporte, 1840, by original designation. Status: valid subgenus of *Cossyphus* G.-A. Olivier, 1791 in Lagriinae: Cossyphini.

*Acritolagria* Borchmann, 1916a: 48, 98 [F]. Type species: *Lagriafulvopilosa* Fairmaire, 1887, by subsequent designation (Borchmann 1936: 166). Status: valid genus in Lagriinae: Lagriini: Lagriina.

*Acromaticus* Koch, 1955a: 143 [M]. Type species: *Sepidiumstriatum* Quensel, 1806 (= *Sepidiumstriatum* Thunberg, 1787), by original designation. Status: valid subgenus of *Somaticus* Hope, 1841 in Pimeliinae: Sepidiini: Trachynotina.

*Acropachia* Mäklin, 1875: 656 [F]. Type species: *Acropachiabifoveolata* Mäklin, 1875, by monotypy. Status: valid genus in Lagriinae: Goniaderini.

*Acropteron* Perty, 1832: 64 [N]. Type species: *Acropteronrufipes* Perty, 1832 (= *Acropteryxrufipes* Gistel, 1831), by subsequent designation (Hope 1841: 133). Status: junior synonym of *Acropteryx* Gistel, 1831 in Tenebrioninae: Acropteronini. Synonymy: Gistel (1857: 551, synonymy of the type species), Martins and Pereira (1966: 159, as “*Acropteroxysrufipes* Distl., 1831”).

*Acropterum* Agassiz, 1846b: 6 [N]. Type species [automatic]: *Acropteronrufipes* Perty, 1832 (= *Acropteryxrufipes* Gistel, 1831), by subsequent designation (Hope 1841: 133). Status: junior synonym of *Acropteryx* Gistel, 1831 in Tenebrioninae: Acropteronini. Note: unjustified emendation of *Acropteron* Perty, 1832, not in prevailing usage.

*Acropteryx* Gistel, 1831: 308 [F]. Type species: *Acropteryxrufipes* Gistel, 1831, by original designation. Status: valid genus in Tenebrioninae: Acropteronini. Note: genus previously included in Erotylidae: Languriinae (see Leschen and Węgrzynowicz 1998: 234); reversal of precedence cannot be used to conserve the usage of *Acropteron* Perty, 1832 because *Acropteryx* was used as valid after 1899 (e.g., Blackwelder 1945: 427).

*Acroschatia* Wilke, 1922: 269 [F]. Type species: *Microschatiarobusta* Horn, 1893, by original designation. Status: junior synonym of *Microschatia* Solier, 1836 in Pimeliinae: Asidini. Synonymy: K.W. Brown and Doyen (1992: 546).

*Actanorie* Bates, 1879a: 289 [M]. Type species: *Camariaundaticollis* Fairmaire, 1875, by original designation. Status: valid genus in Stenochiinae: Cnodalonini.

*Actizeta* Pascoe, 1875: 214 [F]. Type species: *Actizetaammobioides* Pascoe, 1875 (= *Actizetaalbata* Pascoe, 1875), by original designation. Status: valid genus in Pimeliinae: Cnemeplatiini: Actizetina.

*Acutogria* Merkl, 1988a: 137 [F]. Type species: *Acutogriafalcata* Merkl, 1988, by original designation. Status: valid genus in Lagriinae: Lagriini: Lagriina.

*Acutoodescelis* Kaszab, 1940b: 941, 951 [F]. Type species: *Platyscelispunctatissima* Fairmaire, 1886, by original designation. Status: valid subgenus of *Oodescelis* Motschulsky, 1845 in Blaptinae: Platyscelidini.

*Adamus* Iwan, 1997: 255 [M]. Type species: *Platynotusmicrositoides* Kaszab, 1975, by original designation. Status: valid genus in Blaptinae: Platynotini: Platynotina.

*Adavius* Mulsant & Rey, 1859a: 126, 138 [M]. Type species: *Adaviusclavipes* Mulsant & Rey, 1859, by monotypy. Status: valid genus in Blaptinae: Opatrini: Ammobiina.

*Addia* Lewis, 1894: 465 [F]. Type species: *Addiascatebrae* Lewis, 1894, by monotypy. Status: junior synonym of *Tetraphyllus* Laporte & Brullé, 1831 in Stenochiinae: Cnodalonini. Synonymy: Masumoto (1982a: 23, probable); Ando (1991: 64, confirmed).

*Adelina* Dejean, 1835: 315 [F]. Type species: *Cucujusplanus* Fabricius, 1801, by monotypy. Status: valid genus in Diaperinae: Diaperini: Adelinina.

*Adelium* W. Kirby, 1819a: 420 [N]. Type species: *Adeliumcalosomoides* W. Kirby, 1819, by subsequent designation (Meyer 1844: 180). Status: valid genus in Lagriinae: Adeliini. Note: the type species previously recognized for this genus was *Carabusporcatus* Fabricius, 1775, by subsequent designation by Lacordaire (1859b: 438).

*Adelodemus* Haag-Rutenberg, 1878: 100 [M]. Type species: *Adelodemusasperulus* Haag-Rutenberg, 1878 (= *Cestrinussqualidus* W.J. MacLeay, 1872), by monotypy. Status: valid genus in Lagriinae: Adeliini.

*Adelonia* Laporte, 1840: 221 [F]. Type species: *Ulomafiliforme* Laporte, 1840, by monotypy. Status: valid genus and subgenus in Lagriinae: Belopini.

*Adelostoma* Duponchel, 1827: 342 [N]. Type species: *Adelostomasulcatum* Duponchel, 1827, by monotypy. Status: valid genus and subgenus in Pimeliinae: Adelostomini.

*Adelostomoides* Carl, 1991: 24 [M]. Type species: *Adelostomagrande* Haag-Rutenberg, 1879, by monotypy. Status: junior synonym of *Zarudnionymus* Semenov-Tjan-Shansky & Bogatchev, 1947 in Pimeliinae: Adelostomini. Synonymy: Purchart (2007: 240).

*Adelozotypus* Kaszab, 1982b: 224 [M]. Type species: *Adelozotypusnovaecaledoniae* Kaszab, 1982, by original designation. Status: valid genus in Lagriinae: Adeliini.

*Adelphinops* Reitter, 1922b: 168, 169 [M]. Type species: *Adelphinusordubadensis* Reitter, 1890, by monotypy. Status: valid subgenus of *Adelphinus* Fairmaire & Coquerel, 1866 in Tenebrioninae: Helopini: Helopina.

*Adelphinus* Fairmaire & Coquerel, 1866: 44 [M]. Type species: *Eutrapelasuturalis* P.H. Lucas, 1847, by monotypy. Status: valid genus and subgenus in Tenebrioninae: Helopini: Helopina.

*Adelphus* Dejean, 1834: 208 [M]. Type species: *Helopsmarginatus* Fabricius, 1792, by subsequent designation (Bousquet and Bouchard 2013a: 58). Status: senior synonym of *Praeugena* Laporte, 1840 in Tenebrioninae: Praeugenini. Synonymy: Chevrolat (1847b: 458). Note: nomen oblitum (see Bouchard and Bousquet, 2020b: 6).

*Adeps* Gistel, 1857: 63 [M]. Type species: *Amarygmuspaykullii* Dalman, 1823, by original designation. Status: junior synonym of *Tetraphyllus* Laporte & Brullé, 1831 in Stenochiinae: Cnodalonini. Synonymy: **new synonym** [PB]. Note: *Adeps* Gistel, 1857, and its replacement name *Adepsion* Strand, 1917, have been forgotten in the literature; the type species of *Adeps* is currently included in the genus *Tetraphyllus* Laporte & Brullé, 1831 and for that reason Gistel’s name is considered a junior synonym of *Tetraphyllus*; junior homonym of *Adeps* Gistel, 1848 [Crustacea].

*Adepsion* Strand, 1917: 90 [N]. Type species [automatic]: *Amarygmuspaykullii* Dalman, 1823, by original designation. Status: junior synonym of *Tetraphyllus* Laporte & Brullé, 1831 in Stenochiinae: Cnodalonini. Synonymy: **new synonym** [PB]. Note: replacement name for *Adeps* Gistel, 1857.

*Ades* Guérin-Méneville, 1857: 277 [M]. Type species: *Adeshemisphericus* Guérin-Méneville, 1857, by monotypy. Status: valid genus in Diaperinae: Leiochrinini.

*Adesmia* Fischer, 1822: 153 [F]. Type species: *Adesmialongipes* Fischer, 1822 (= *Pimeliaanomala* Fischer, 1820), by monotypy. Status: valid genus and subgenus in Pimeliinae: Adesmiini.

*Adesmina* Reitter, 1916a: 5, 29 [F]. Type species: *Adesmiaarabica* Reitter, 1916, by subsequent designation (Löbl et al. 2008b: 122). Status: valid subgenus of *Adesmia* Fischer, 1822 in Pimeliinae: Adesmiini.

*Adisema* Borchmann, 1936: pl. 7 [F]. Type species: *Disemaambigua* Mäklin, 1875, by monotypy. Status: junior synonym of *Nemostiromorpha* Pic, 1917 in Lagriinae: Lagriini: Statirina.

*Adonicus* Fairmaire, 1891b: 258 [M]. Type species: *Adonicuspurpuripennis* Fairmaire, 1891, by monotypy. Status: junior synonym of *Alcyonotus* Pascoe, 1882 in Stenochiinae: Cnodalonini. Synonymy: Gebien (1905: 255).

*Adordanea* Reitter, 1897b: 229 [F]. Type species: *Microderasubseriata* Reitter, 1889, by monotypy. Status: junior synonym of *Dordanea* Reitter, 1887 in Pimeliinae: Tentyriini. Synonymy: Kaszab (1966: 304), G.S. Medvedev (1990: 112).

*Adoryacus* Koch, 1963: 34 [M]. Type species: *Stizopusrotundicollis* Fairmaire, 1897, by original designation. Status: valid genus in Blaptinae: Opatrini: Stizopodina.

*Adosogria* Borchmann, 1936: 16, 63 [F]. Type species: *Lagriabennigseni* Borchmann, 1909, by original designation. Status: valid genus in Lagriinae: Lagriini: Lagriina.

*Adynata* Fåhraeus, 1870: 330 [F]. Type species: *Adynatatricolor* Fåhraeus, 1870, by subsequent designation (Borchmann 1936: 23). Status: valid genus in Lagriinae: Lagriini: Lagriina.

*Aeanes* Champion, 1893a: 566 [M]. Type species: *Aeanesangusticollis* Champion, 1893, by monotypy. Status: valid genus in Alleculinae: Alleculini: Alleculina.

*Aediatorix* Bates, 1868: 315 [M]. Type species: *Aediatorixjansoni* Bates, 1868, by monotypy. Status: valid genus in Lagriinae: Pycnocerini.

*Aemymone* Bates, 1868: 314 [F]. Type species: *Goniaderacariosa* Bates, 1868, by original designation. Status: valid subgenus of *Goniadera* Perty, 1832 in Lagriinae: Goniaderini. Note: combined description of a new genus and single new species (ICZN 1999, Article 12.2.6).

*Aequigula* Penrith, 1979: 20, 23 [F]. Type species: *Alogeniushughesae* Penrith, 1979, by original designation. Status: valid subgenus of *Alogenius* Gebien, 1910 in Pimeliinae: Adesmiini.

*Aeschrocera* Chen & Chou, 1996: 265 [F]. Type species: *Cerogriabrunneocollis* Chen & Chou, 1996 (= *Cerogriadiversicornis* Pic, 1933), by original designation. Status: junior synonym of *Cerogria* Borchmann, 1911 in Lagriinae: Lagriini: Lagriina. Synonymy: Merkl (2007: 262).

*Aesthetus* C.O. Waterhouse, 1890: 552 [M]. Type species: *Aesthetustuberculatus* C.O. Waterhouse, 1890, by monotypy. Status: valid genus in Stenochiinae: Cnodalonini.

*Aesymnus* Champion, 1886: 168 [M]. Type species: *Aesymnusnitidus* Champion, 1886, by monotypy. Status: valid genus in Tenebrioninae: Triboliini.

*Aethales* Dejean, 1834: 180 [M]. Type species: *Epitragusbrunnicornis* Latreille, 1811, by monotypy. Status: junior synonym of *Epitragus* Latreille, 1802 in Pimeliinae: Epitragini. Synonymy: Bousquet and Bouchard (2013a: 43). Note: as mentioned by Bousquet and Bouchard (2013a: 43) the type species *Epitragusbrunnicornis* Latreille was listed as a species incertae sedis in the genus *Epitragus* Latreille, 1802 by Freude (1967: 176).

*Aethalides* Bates, 1873d: 50 [M]. Type species: *Aethalidespunctipennis* Bates, 1873, by monotypy. Status: junior synonym of *Nyctozoilus* Guérin-Méneville, 1831 in Tenebrioninae: Heleini: Cyphaleina. Synonymy: Matthews (1992: 473).

*Aethyssius* Pascoe, 1863a: 45 [M]. Type species [automatic]: *Atractusviridis* Boisduval, 1835, by subsequent designation (Duponchel and Chevrolat 1841: 312). Status: junior synonym of *Lepturidea* Fauvel, 1862 in Alleculinae: Alleculini: Alleculina. Synonymy: Matthews and Bouchard (2008: 324). Note: replacement name for *Atractus* Boisduval, 1835.

*Afghanillus* Kaszab, 1960a: 1 [M]. Type species: *Afghanillusklapperichi* Kaszab, 1960, by original designation. Status: valid genus in Pimeliinae: Stenosini: Dichillina.

*Afghanopachys* Kwieton, 1978: 29, 30 [M]. Type species: *Pachyscelishaarlovi* Gridelli, 1954, by original designation. Status: valid genus in Pimeliinae: Pimeliini.

*Afrasida* Wilke, 1922: 260 [F]. Type species: *Asidacaryophyllea* Wiedemann, 1823, by original designation. Status: valid genus and subgenus in Pimeliinae: Asidini.

*Afrethas* Koch, 1962a: 23 [M]. Type species: *Stenosispusillima* Fairmaire, 1888, by original designation. Status: valid subgenus of *Anethas* Jakobson, 1924 in Pimeliinae: Stenosini: Stenosina.

*Afrinus* Fairmaire, 1888a: 189 [M]. Type species: *Afrinusgrandicornis* Fairmaire, 1888, by subsequent designation (R. Lucas 1920: 79). Status: valid genus and subgenus in Pimeliinae: Tentyriini. Note: the designation of *Afrinusstriolifrons* Fairmaire, 1888 as the type species of *Afrinus* Fairmaire, 1888 by Koch (1950b: 329) is invalid (ICZN 1999, Article 69.1).

*Afrobyrsax* Ardoin, 1973: 894 [M]. Type species: *Afrobyrsaxgirardi* Ardoin, 1973, by original designation. Status: valid genus in Tenebrioninae: Bolitophagini.

*Afrohelops* Schawaller, 2012a: 75 [M]. Type species: *Afrohelopskenyaensis* Schawaller, 2012, by original designation. Status: valid genus in Tenebrionidae: incertae sedis. Note: removed from the tribe Helopini and placed as Tenebrionidae incertae sedis by Nabozhenko (2018: 183).

*Afrolaena* Endrödy-Younga & Schawaller, 2002: 9, 11 [F]. Type species: *Afrolaenatibialis* Endrödy-Younga & Schawaller, 2002, by original designation. Status: valid genus in Lagriinae: Laenini.

*Afronosis* G.S. Medvedev, 1995a: 849, 859 [F]. Type species: *Stenosisciliaris* Gebien, 1920, by original designation. Status: valid subgenus of *Stenosis* Herbst, 1799 in Pimeliinae: Stenosini: Stenosina. Note: *Stenosisleontjevi* G.S. Medvedev, 1995 was listed as a second type species of this genus in the original publication (G.S. Medvedev, 1995: 859); we act as First Revisers and select *Stenosisciliaris* Gebien, 1920 as the type species for *Afronosis* G.S. Medvedev, 1995.

*Afrostrongylium* Robiche, 2019a: 83 [N]. Type species: *Strongyliumfrancoisi* Robiche, 2019, by original designation. Status: valid subgenus of *Strongylium* W. Kirby, 1819 in Stenochiinae: Stenochiini.

*Afrotagalus* Gebien, 1942b: 111, 120 [M]. Type species: *Afrotagaluseidmanni* Gebien, 1942, by original designation. Status: valid genus in Phrenapatinae: Penetini.

*Afrotenebrio* Gridelli, 1951: 223, 230 [M]. Type species: *Tenebrioguineensis* Imhoff, 1843, by original designation. Status: valid subgenus of *Tenebrio* Linnaeus, 1758 in Tenebrioninae: Tenebrionini.

*Agastenes* R. Lucas, 1920: 79 [M]. Type species [automatic]: *Agastheneswestwoodi* Bates, 1873, by monotypy. Status: valid genus in Tenebrioninae: Heleini: Cyphaleina. Note: unjustified emendation of *Agasthenes* Bates, 1873.

*Agasthenes* Bates, 1873e: 352 [M]. Type species: *Agastheneswestwoodi* Bates, 1873, by monotypy. Status: senior synonym of *Agastenes* R. Lucas, 1920 in Tenebrioninae: Heleini: Cyphaleina. Note: junior homonym of *Agasthenes* Förster, 1869 [Hymenoptera].

*Agelarches* Gistel, 1848a: x [M]. Type species [automatic]: *Pimeliaangulata* Fabricius, 1775, by subsequent designation (Hope 1841: 118). Status: junior synonym of *Pimelia* Fabricius, 1775 in Pimeliinae: Pimeliini. Note: unnecessary replacement name for *Pimelia* Fabricius, 1775.

*Agissopterus* Fairmaire, 1884a: 513 [M]. Type species: *Agissopterussemipunctatus* Fairmaire, 1884, by original designation. Status: valid genus in Stenochiinae: Stenochiini.

*Aglypta* Gebien, 1908a: 329 [F]. Type species: *Aglyptaoctocostata* Gebien, 1908, by monotypy. Status: valid genus in Tenebrioninae: Heleini: Cyphaleina.

*Agnaptoria* Reitter, 1887a: 364, 372 [F]. Type species: *Agnaptoriarubripes* Reitter, 1887, by monotypy. Status: valid genus in Blaptinae: Blaptini: Gnaptorinina.

*Agonopus* Gebien, 1920: 110 [M]. Type species: *Gonopuspuncticollis* Solier, 1848, by subsequent designation (Endrödy-Younga 2000: 7). Status: valid subgenus of *Gonopus* Latreille, 1828 in Blaptinae: Platynotini: Platynotina.

*Agraecus* Fairmaire, 1900a: 21 [M]. Type species: *Agraecuschalcoides* Fairmaire, 1900, by monotypy. Status: junior synonym of *Carabelops* Fairmaire, 1899 in Stenochiinae: Cnodalonini. Synonymy: Ardoin (1956b: 90).

*Agroblaps* Motschulsky, 1860c: 531 [F]. Type species: *Blaps fatidica* Sturm, 1807 (= *Blaps lethifera* Marsham, 1802), by subsequent designation (Nabozhenko 2008: 35). Status: junior synonym of *Blaps* Fabricius, 1775 in Blaptinae: Blaptini: Blaptina. Synonymy: Gemminger in Gemminger and Harold (1870: 1860).

*Agymnonyx* Gebien, 1921a: 325, 328 [M]. Type species: *Agymnonyxsulciventris* Gebien, 1921, by subsequent designation (Gebien 1942a: 334). Status: valid genus in Stenochiinae: Cnodalonini.

*Ahexaroptrum* Kaszab, 1960b: 291 [N]. Type species: *Ahexaroptrumhumeridens* Kaszab, 1960, by original designation. Status: valid genus in Stenochiinae: Cnodalonini.

*Ainu* Lewis, 1894: 479 [M]. Type species: *Ainutenuicornis* Lewis, 1894, by monotypy. Status: valid genus and subgenus in Stenochiinae: Cnodalonini.

*Akis* Herbst, 1799: 124 [F]. Type species: *Pimeliareflexa* Fabricius, 1775, by subsequent designation (Latreille 1810: 429). Status: valid genus in Pimeliinae: Akidini.

*Alaephus* Horn, 1870: 344, 346 [M]. Type species: *Alaephuspallidus* Horn, 1870, by monotypy. Status: valid genus in Pimeliinae: Vacronini.

*Alaetrinus* Iwan, 1995a: 14, 24 [M]. Type species: *Tenebriopullus* Sahlberg, 1823, by original designation. Status: valid genus in Blaptinae: Platynotini: Platynotina.

*Alagria* Borchmann, 1916a: 50, 183 [F]. Type species [automatic]: *Lagriostirahispida* Kolbe, 1902 (= *Lagriasubseriata* Reitter, 1880), by subsequent designation (Borchmann 1936: 224). Status: valid genus in Lagriinae: Lagriini: Lagriina. Note: replacement name for *Lagriostira* Kolbe, 1902.

*Alaudes* Horn, 1870: 361 [M]. Type species: *Alaudessingularis* Horn, 1870, by monotypy. Status: valid genus in Pimeliinae: Cnemeplatiini: Alaudina.

*Alcinoe* Ménétriés, 1849: 230 [F]. Type species: *Alcinoehelopioides* Ménétriés, 1849, by monotypy. Status: senior synonym of *Alcinoeta* Strand, 1929 in Pimeliinae: Tentyriini. Note: junior homonym of *Alcinoe* Rang, 1828 [Ctenophora].

*Alcinoeta* Strand, 1929: 23 [F]. Type species [automatic]: *Alcinoehelopioides* Ménétriés, 1849, by monotypy. Status: valid genus in Pimeliinae: Tentyriini. Note: replacement name for *Alcinoe* Ménétriés, 1849.

*Alcmeonis* Bates, 1868: 270 [F]. Type species: *Alcmeonispulchra* Bates, 1868, by monotypy. Status: junior synonym of *Lepturidea* Fauvel, 1862 in Alleculinae: Alleculini: Alleculina. Synonymy: Matthews and Bouchard (2008: 324, with *Aethyssius* Pascoe, 1863, a junior synonym of *Lepturidea* Fauvel, 1862).

*Alcyonotus* Pascoe, 1882: 35 [M]. Type species: *Alcyonotusiridescens* Pascoe, 1882, by monotypy. Status: valid genus in Stenochiinae: Cnodalonini.

*Alegoria* Laporte, 1840: 221 [F]. Type species: *Alegoriadilatata* Laporte, 1840, by monotypy. Status: valid genus in Tenebrioninae: Ulomini.

*Alethia* Champion, 1888: 417 [F]. Type species: *Alethiasallaei* Champion, 1888, by original designation. Status: valid genus in Alleculinae: Alleculini: Alleculina.

*Alhuena* Kulzer, 1956b: 912 [F]. Type species: *Alhuenapenai* Kulzer, 1956 (= *Peltolobuswaterhousei* Bates, 1873), by original designation. Status: junior synonym of *Peltolobus* Lacordaire, 1859 in Pimeliinae: Trilobocarini. Synonymy: Kulzer (1962: 100).

*Alienophloeus* Bremer, 2018: 45 [M]. Type species: *Corticeuspumilio* Bremer, 2018, by original designation. Status: valid subgenus of *Corticeus* Piller & Mitterpacher, 1783 in Diaperinae: Hypophlaeini.

*Alienoplonyx* Bremer, 2019: 59 [M]. Type species: *Alienoplonyxalleni* Bremer, 2019, by monotypy. Status: valid genus in Tenebrioninae: Amarygmini. Note: we act as First Revisers and reject the alternative original spelling *Alienolonyx*, used by Bremer (2019: 60).

*Allardius* Ragusa, 1898: 130 [M]. Type species: *Parablopsoculatus* Baudi di Selve, 1876, by monotypy. Status: valid genus in Tenebrioninae: Helopini: Helopina.

*Allecula* Fabricius, 1801b: 21 [F]. Type species: *Cistelamorio* Fabricius, 1787, by subsequent designation (Duponchel 1840: 283). Status: valid genus and subgenus in Alleculinae: Alleculini: Alleculina.

*Alleculina* Pic, 1954: 246 [F]. Type species: *Synalleculamacroceps* Pic, 1922, by original designation. Status: valid subgenus of *Allecula* Fabricius, 1801 in Alleculinae: Alleculini: Alleculina.

*Alleculodes* Borchmann, 1925: 335 [F]. Type species: *Alleculodesdiscrepans* Borchmann, 1925, by original designation. Status: junior synonym of *Bolbostetha* Fairmaire, 1896 in Alleculinae: Alleculini: Alleculina. Synonymy: Borchmann (1941a: 23).

*Alleculopsis* Semenov, 1894: 609 [F]. Type species: *Alleculopsisdeserticola* Semenov, 1894, by original designation. Status: valid subgenus of *Mycetocharina* Seidlitz, 1890 in Alleculinae: Alleculini: Alleculina.

*Allocera* Borchmann, 1916a: 49, 175 [F]. Type species: *Porrolagriasubaenea* Borchmann, 1908, by subsequent designation (Borchmann 1936: 227). Status: senior synonym of *Cerostira* Borchmann, 1942 in Lagriinae: Lagriini: Lagriina. Note: junior homonym of *Allocera* Sichel, 1866 [Hymenoptera].

*Allocossyphodes* Andreae, 1961: 204, 215 [M]. Type species: *Cossyphodesarnoldi* Brauns, 1925, by original designation. Status: junior synonym of *Cossyphodes* Westwood, 1851 in Pimeliinae: Cossyphodini: Cossyphodina. Synonymy: Schawaller (2013c: 362, implied by inclusion of *Cossyphodesarnoldi* Brauns, 1925 in Cossyphodes Westwood, 1851 without use of a subgenus rank).

*Allodengitha* Bogatchev, 1963: 57 [F]. Type species: *Allodengithadeserta* Bogatchev, 1963, by original designation. Status: junior synonym of *Alcinoeta* Strand, 1929 in Pimeliinae: Tentyriini. Synonymy: G.S. Medvedev (1989: 798).

*Allogria* Borchmann, 1916a: 48, 100 [F]. Type species: *Allogriaspinosa* Borchmann, 1916, by subsequent designation (Borchmann 1936: 74). Status: valid genus in Lagriinae: Lagriini: Lagriina.

*Allomyladion* Bogatchev, 1972: 626 [N]. Type species: *Penthicusporicollis* Reitter, 1896, by original designation. Status: valid subgenus of *Penthicus* Faldermann, 1836 in Tenebrioninae: Opatrini: Opatrina.

*Allopezus* Gebien, 1922a: 504 [M]. Type species: *Allopezusmiritarsis* Gebien, 1922, by monotypy. Status: junior synonym of *Asbolodomimus* Pic, 1921 in Stenochiinae: Cnodalonini. Synonymy: Gebien (1948: 543).

*Allophasia* Pascoe, 1871: 351 [F]. Type species: *Allophasiafryi* Pascoe, 1871, by monotypy. Status: junior synonym of *Diaperis* Geoffroy, 1762 in Diaperinae: Diaperini: Diaperina. Synonymy: Triplehorn and Brendell (1985: 14).

*Allophylax* Bedel, 1906b: 177 [M]. Type species: *Phylaxlittoralis* Mulsant, 1854 (= *Opatrumpicipes* G.-A. Olivier, 1812), by subsequent designation (Gebien 1938a: 412). Status: valid genus and subgenus in Blaptinae: Dendarini: Melambiina. Note: replacement name for *Neophylax* Bedel, 1906.

*Allostrongylium* Kolbe, 1896: 364 [N]. Type species: *Allostrongyliumsilvestre* Kolbe, 1896, by **present designation**. Status: junior synonym of *Strongylium* W. Kirby, 1819 in Stenochiinae: Stenochiini. Synonymy: Gebien (1948: 539, with *Anomoearthrum* Mäklin, 1867, a junior synonym of *Strongylium* W. Kirby, 1819).

*Allotadzhikistania* Bogatchev, 1960a: 43 [F]. Type species: *Allotadzhikistaniacomata* Bogatchev, 1960, by original designation. Status: valid genus in Pimeliinae: Pimeliini.

*Allotriocochabambia* Faúndez, Rider & Carvajal, 2014: 595 [F]. Type species [automatic]: *Cochabambiakulzeri* Marcuzzi, 1985, by monotypy. Status: valid genus in Tenebrionidae: incertae sedis. Note: replacement name for *Cochabambia* Marcuzzi, 1985; Marcuzzi (1985: 185) mentioned that his new genus *Cochabambia* could not be placed in “any given Neotropical tribe so far known” and is therefore included here in Tenebrionidae incertae sedis under its replacement name.

*Alobates* Motschulsky, 1872: 25 [M]. Type species: *Tenebriopensylvanicus* DeGeer, 1775, by original designation. Status: valid genus in Stenochiinae: Cnodalonini.

*Alogenius* Gebien, 1910a: 91 [M]. Type species [automatic]: *Metriopusfavosus* Erichson, 1843, by subsequent designation (Gebien 1937a: 661). Status: valid genus and subgenus in Pimeliinae: Adesmiini. Note: replacement name for *Pedionomus* Haag-Rutenberg, 1875.

*Alogista* Fåhraeus, 1870: 318 [F]. Type species: *Alogistaabnormis* Fåhraeus, 1870, by monotypy. Status: valid genus in Alleculinae: Alleculini: Alleculina.

*Alogistopsis* Borchmann, 1943: 53 [F]. Type species: *Alogistopsispilistriata* Borchmann, 1943, by monotypy. Status: valid genus in Alleculinae: incertae sedis.

*Alphasida* Escalera, 1905a: 380 [F]. Type species: *Asidagaditana* Escalera, 1905 (= *Alphasidatypica* Gebien 1937), by subsequent designation (Casey 1912: 78). Status: valid genus and subgenus in Pimeliinae: Asidini.

*Alphitobius* Stephens, 1829: 19 [M]. Type species: *Helopspicipes* Panzer, 1794 (= *Opatrumlaevigatum* Fabricius, 1781), by monotypy (see ICZN 1975, Opinion 1039). Status: valid genus in Tenebrioninae: Alphitobiini. Note: placed on the Official List of Generic Names in Zoology (ICZN 1975, Opinion 1039).

*Alphitophagus* Stephens, 1832b: 12 [M]. Type species: *Alphitophagusquadripustulatus* Stephens, 1832 (= *Diaperisbifasciata* Say, 1824), by monotypy. Status: valid genus in Diaperinae: Diaperini: Adelinina.

†*Alphitopsis* Kirejtshuk, Nabozhenko & Nel, 2011: 549 [F]. Type species: *Alphitopsisinitialis* Kirejtshuk, Nabozhenko & Nel, 2011, by original designation. Status: valid genus in Tenebrioninae: Alphitobiini. Note: described from Lower Cretaceous deposits (China).

*Altes* Pascoe, 1869: 288, 290 [M]. Type species: *Chartopteryxbinodosus* Pascoe, 1862, by original designation. Status: junior synonym of *Cyphaleus* Westwood, 1841 in Tenebrioninae: Heleini: Cyphaleina. Synonymy: Gemminger in Gemminger and Harold (1870: 1994, with *Chartopteryx* Westwood, 1841, a synonym of *Cyphaleus* Westwood, 1841); Matthews (1992: 490).

*Altiprosodes* G.S. Medvedev, 1997: 580 [M]. Type species: *Prosodeskuhistanica* G.S. Medvedev, 1996, by original designation. Status: junior synonym of *Megaprosodes* Reitter, 1909 in Blaptinae: Blaptini: Prosodina. Synonymy: G.S. Medvedev (2001: 83). Note: originally described as a section within a subgenus.

*Alymon* Pascoe, 1866a: 484 [M]. Type species: *Alymonprolatus* Pascoe, 1866, by monotypy. Status: valid genus in Tenebrioninae: Amarygmini.

*Amachla* Koch, 1962a: 117 [F]. Type species: *Machlasulcicollis* Fåhraeus, 1871, by original designation. Status: valid genus in Pimeliinae: Asidini.

*Amarantha* Motschulsky, 1860b: 141 [F]. Type species: *Amaranthaviridis* Motschulsky, 1860, by monotypy. Status: senior synonym of *Metaclisa* Jacquelin du Val, 1861 in Tenebrioninae: Metaclisini. Synonymy: Lewis (1891: 70). Note: nomen oblitum (see Bouchard et al. 2007: 393).

*Amaropsis* Champion, 1893a: 567 [F]. Type species: *Amaropsisannulicornis* Champion, 1893, by monotypy. Status: valid genus in Alleculinae: Alleculini: Alleculina.

*Amarosoma* Redtenbacher, 1868: 131 [N]. Type species: *Amarosomasimulans* Redtenbacher, 1868, by monotypy. Status: junior synonym of *Pheloneis* Pascoe, 1866 in Lagriinae: Adeliini. Synonymy: Pascoe (1876: 52).

*Amarsenes* Bates, 1879a: 297 [M]. Type species: *Tetraphyllusoblongocamelus* Fairmaire, 1877, by monotypy. Status: valid genus in Stenochiinae: Cnodalonini.

*Amarygmimus* Bates, 1873e: 354 [M]. Type species: *Amarygmimusduboulayi* Bates, 1873, by monotypy. Status: valid genus in Tenebrioninae: Heleini: Cyphaleina.

*Amarygmomimus* Rye, 1875: 290 [M]. Type species [automatic]: *Amarygmimusduboulayi* Bates, 1873, by monotypy. Status: junior synonym of *Amarygmimus* Bates, 1873 in Tenebrioninae: Heleini: Cyphaleina. Note: unjustified emendation of *Amarygmimus* Bates, 1873, not in prevailing usage.

*Amarygmus* Dalman, 1823: 60 [M]. Type species: *Chrysomelamicans* Fabricius, 1794, by subsequent designation (Gebien 1921a: 410). Status: valid genus and subgenus in Tenebrioninae: Amarygmini.

*Amathobius* Gebien, 1920: 129 [M]. Type species: *Amathobiusglyptopterus* Gebien, 1920, by subsequent designation (Koch 1963: 42). Status: valid genus in Blaptinae: Opatrini: Stizopodina.

*Amatodes* Dejean, 1834: 189 [F]. Type species: *Pimeliagemmata* Fabricius, 1801, by monotypy. Status: valid genus and subgenus in Blaptinae: Pedinini: Helopinina. Note: the original combination of the name of the type species, *Pimeliagemmata* Fabricius, 1801, is a junior primary homonym of *Pimeliagemmata* Herbst, 1799.

†*Amberophlus* Novák & Háva, 2019: 128 [M]. Type species: *Amberophlusniger* Novák & Háva, 2019, by original designation. Status: valid genus in Alleculinae: Cteniopodini. Note: described from Eocene Baltic amber.

*Ambigatus* Fairmaire, 1892b: 246 [M]. Type species: *Ambigatusrufonitens* Fairmaire, 1892, by subsequent designation (R. Lucas 1920: 87). Status: valid subgenus of *Achanius* Erichson, 1847 in Pimeliinae: Evaniosomini.

*Amblycara* Fairmaire, 1893a: cxlvii [N]. Type species: *Amblycarabiskrense* Fairmaire, 1893 (= *Melancrusalutaceum* Fairmaire, 1875), by monotypy. Status: senior synonym of *Amblycarenum* Gebien, 1910 in Pimeliinae: Tentyriini. Note: junior homonym of *Amblycara* Bergroth, 1891 [Hemiptera].

*Amblycarenum* Gebien, 1910a: 77 [N]. Type species [automatic]: *Amblycarabiskrense* Fairmaire, 1893 (= *Melancrusalutaceum* Fairmaire, 1875), by monotypy. Status: valid genus in Pimeliinae: Tentyriini. Note: replacement name for *Amblycara* Fairmaire, 1893.

*Amblychirus* Koch, 1956a: 87 [M]. Type species: *Trigonopusbrevior* Fairmaire, 1897, by original designation. Status: valid genus in Blaptinae: Platynotini: Platynotina.

*Amblycyphus* Motschulsky, 1870: 401 [M]. Type species: *Amblycyphusasperatus* Motschulsky, 1870 (= *Centriopterapectoralis* Blaisdell, 1921), by monotypy. Status: junior synonym of *Cryptoglossa* Solier, 1837 in Pimeliinae: Cryptoglossini. Synonymy: Aalbu et al. (1995: 483).

*Amblyptera* Solier, 1836: 195 [F]. Type species: *Pimeliascabrosa* Solier, 1836, by subsequent designation (Viñolas and Cartagena 2005: 267). Status: valid subgenus of *Pimelia* Fabricius, 1775 in Pimeliinae: Pimeliini.

*Amblypteraca* Mas-Peinado, Buckley, Ruiz & García-París, 2018: 543 [F]. Type species: *Pimeliarugosa* Fabricius, 1792 (= *Pimeliafairmairii* Kraatz, 1865), by original designation. Status: valid subgenus of *Pimelia* Fabricius, 1775 in Pimeliinae: Pimeliini. Note: we act as First Revisers and reject the alternative original spelling *Amplypteraca*, used by Mas-Peinado et al. (2018: 531, 543).

*Amblysphagus* Fairmaire, 1896a: 16 [M]. Type species: *Amblysphaguspachyderus* Fairmaire, 1896, by monotypy. Status: valid genus in Blaptinae: Opatrini: Neopachypterina.

*Amenophis* J. Thomson, 1858: 93 [M]. Type species: *Amenophisfairmairei* J. Thomson, 1858, by subsequent designation (Gebien 1941: 342). Status: valid genus and subgenus in Stenochiinae: Cnodalonini.

*Ametrocera* Fåhraeus, 1870: 260 [F]. Type species: *Ametroceraaurita* Fåhraeus, 1870, by subsequent designation (R. Lucas 1920: 88). Status: valid genus in Blaptinae: Pedinini: Helopinina.

*Amiantus* Fåhraeus, 1870: 279 [M]. Type species: *Amiantusgibbosus* Fåhraeus, 1870, by subsequent designation (Haag-Rutenberg 1871: 45). Status: valid genus in Pimeliinae: Sepidiini: Molurina.

*Amicrodera* Kaszab, 1966: 282, 292 [F]. Type species: *Microderalindbergi* Kaszab, 1966, by original designation. Status: valid subgenus of *Microdera* Eschscholtz, 1831 in Pimeliinae: Tentyriini.

*Ammidanemia* Reitter, 1904: 132 [F]. Type species: *Anemiafausti* Solsky, 1882 (= *Cheirodesbrevicollis* Wollaston, 1864), by subsequent designation (G.S. Medvedev 1990: 212). Status: junior synonym of *Pseudanemia* Wollaston, 1864 in Tenebrioninae: Melanimonini. Synonymy: Ardoin (1971: 359, through placement of the type species in the subgenusPseudanemia Wollaston, 1864).

*Ammidium* Erichson, 1843: 250 [N]. Type species: *Ammidiumciliatum* Erichson, 1843, by monotypy. Status: valid genus in Blaptinae: Opatrini: Ammobiina.

*Ammobius* Guérin-Méneville, 1844: 121 [M]. Type species: *Ammobiusrufus* Guérin-Méneville, 1844, by monotypy. Status: valid genus in Blaptinae: Opatrini: Ammobiina. Note: combined description of a new genus and a single new species (ICZN 1999, Article 12.2.6); the original combination of the name of the type species has been given as “*Trachyscelisrufus* Guérin-Méneville, 1844” in recent literature; however, we use the combination *Ammobiusrufus* here as it was used by Guérin-Méneville (1844: 560) in his ‘Table alphabétique des genres, sous-genres et espèces figurés ou décrits dans l’iconographie du règne animal. Insectes’.

*Ammocera* Borchmann, 1941a: 5 [F]. Type species: *Ammoceraunicolor* Borchmann, 1941, by original designation. Status: valid subgenus of *Lagria* Fabricius, 1775 in Lagriinae: Lagriini: Lagriina.

*Ammodoides* Lesne, 1915: 227, 233 [M]. Type species: *Arthrodeislateripunctatus* Fairmaire, 1890, by monotypy. Status: valid genus in Pimeliinae: Erodiini.

*Ammodonus* Mulsant & Rey, 1859a: 141, 143 [M]. Type species: *Opatrumfossor* J.L. LeConte, 1847, by monotypy. Status: valid genus in Blaptinae: Opatrini: Blapstinina. Note: transferred from Ammobiina by Lumen et al. (2020: 341).

*Ammogiton* Peyerimhoff, 1920: 326 [M]. Type species: *Ammogitonviberti* Peyerimhoff, 1920, by monotypy. Status: valid genus in Pimeliinae: Tentyriini.

*Ammophorus* Guérin-Méneville, 1831a: pl. 4 [M]. Type species: *Ammophorusperuvianus* Guérin-Méneville, 1831, by monotypy. Status: valid genus in Tenebrioninae: Scotobiini.

*Ammophtorus* Lacordaire, 1859a: 284 [M]. Type species [automatic]: *Ammobiusrufus* Guérin-Méneville, 1844, by monotypy. Status: junior synonym of *Ammobius* Guérin-Méneville, 1844 in Blaptinae: Opatrini: Ammobiina. Note: unnecessary replacement name for *Ammobius* Guérin-Méneville, 1844.

*Ammotrypes* Fairmaire, 1879a: 194 [M]. Type species: *Ammotrypescrenulicollis* Fairmaire, 1879, by monotypy. Status: junior synonym of *Eurycaulus* Fairmaire, 1868 in Blaptinae: Opatrini: Sclerina. Synonymy: Grimm (2005: 9); Löbl and Smetana (2010: 33).

*Ammozoides* Kaszab, 1979a: 91 [M]. Type species: *Ammozoumvalidicorne* Reitter, 1900, by original designation. Status: valid genus in Pimeliinae: Erodiini.

*Ammozoum* Semenov, 1891: 352 [N]. Type species: *Ammozoumhyalinum* Semenov, 1891, by monotypy. Status: valid genus in Pimeliinae: Erodiini.

*Amnodeis* Miller, 1858: 117 [M]. Type species: *Amnodeisgrandis* Miller, 1858, by subsequent designation (R. Lucas 1920: 90). Status: valid genus in Pimeliinae: Erodiini.

*Amorphochirus* Gebien, 1904a: 339 [M]. Type species: *Pycnocerushercules* Fairmaire, 1884, by monotypy. Status: valid genus in Lagriinae: Pycnocerini.

*Amorphopoda* Fåhraeus, 1870: 320 [F]. Type species: *Amorphopodaelateroides* Fåhraeus, 1870, by monotypy. Status: valid genus in Alleculinae: incertae sedis.

*Amphianax* Bates, 1873e: 350 [M]. Type species: *Amphianaxsubcoriaceus* Bates, 1873, by monotypy. Status: valid genus in Tenebrioninae: Heleini: Cyphaleina.

*Amphidora* Eschscholtz, 1829: 9 [F]. Type species: *Amphidoralittoralis* Eschscholtz, 1829, by monotypy. Status: valid subgenus of *Eleodes* Eschscholtz, 1829 in Blaptinae: Amphidorini. Note: Doyen and Lawrence (1979: 367) synonymized this genus with *Eleodes* Eschscholtz, 1829; however, Smith and Johnston in Bousquet et al. (2018: 143) used *Amphidora* as a valid subgenus of *Eleodes*.

*Amphithrix* Español, 1952b: 306 [F]. Type species: *Amphithrixpeyerimhoffi* Español, 1952, by original designation. Status: senior synonym of *Amphithrixoides* Bouchard & Löbl, 2008 in Blaptinae: Opatrini: Ammobiina. Note: junior homonym of *Amphithrix* Ragonot, 1893 [Lepidoptera].

*Amphithrixoides* Bouchard & Löbl, 2008: 39 [M]. Type species [automatic]: *Amphithrixpeyerimhoffi* Español, 1952, by original designation. Status: valid genus in Blaptinae: Opatrini: Ammobiina. Note: replacement name for *Amphithrix* Español, 1952.

*Anacardiosis* Endrödy-Younga, 1986: 210, 221 [F]. Type species: *Cardiosishamiltonuli* Koch, 1969, by original designation. Status: valid subgenus of *Zophosis* Latreille, 1802 in Pimeliinae: Zophosini.

*Anachayus* Bouchard & Bousquet, **new replacement name** [M]. Type species [automatic]: *Chemolanusvillosipes* Fairmaire, 1884, by original designation. Status: valid genus in Stenochiinae: Cnodalonini. Note: replacement name for *Chatanayus* Ardoin, 1957.

*Anacycus* Fairmaire, 1896a: 33 [M]. Type species: *Dietysusnavicularis* Fairmaire, 1894, by subsequent designation (Löbl et al. 2008: 215). Status: junior synonym of *Amarygmus* Dalman, 1823 in Tenebrioninae: Amarygmini. Synonymy: Blair (1929a: 245; with *Elixota* Pascoe, 1866, a junior synonym of *Amarygmus* Dalman, 1823).

*Anadischidus* Kolbe, 1897a: 241 [M]. Type species: *Nyctobatesiphthinoides* Quedenfeldt, 1885, by monotypy. Status: junior synonym of *Deriles* Motschulsky, 1872 in Stenochiinae: Cnodalonini. Synonymy: Gebien (1911a: 441).

*Anaedes* Agassiz, 1846b: 20 [M]. Type species [automatic]: *Anaeduspunctatissimus* Blanchard, 1842, by monotypy. Status: junior synonym of *Anaedus* Blanchard, 1842 in Lagriinae: Goniaderini. Note: unjustified emendation of *Anaedus* Blanchard, 1842, not in prevailing usage.

*Anaedus* Blanchard, 1842: pl. 14 [M]. Type species: *Anaeduspunctatissimus* Blanchard, 1842, by monotypy. Status: valid genus in Lagriinae: Goniaderini.

*Anamenederes* Koch, 1954a: 68 [M]. Type species: *Menederesdigitatus* Koch, 1954, by original designation. Status: valid subgenus of *Menederes* Solier, 1848 in Blaptinae: Platynotini: Eurynotina.

*Anamphidora* Casey, 1924: 330 [F]. Type species: *Anamphidoraparvula* Casey, 1924, by original designation. Status: valid genus in Alleculinae: Alleculini: Xystropodina.

*Anarmostodera* Fairmaire, 1897a: 114 [F]. Type species: *Anarmostoderacrassicornis* Fairmaire, 1897, by monotypy. Status: valid genus in Tenebrioninae: Praeugenini.

*Anatolica* Eschscholtz, 1831: 5, 7 [F]. Type species: *Tentyriasubquadrata* Tauscher, 1812, by subsequent designation (Gebien 1937a: 599). Status: valid genus and subgenus in Pimeliinae: Tentyriini.

*Anatrum* Reichardt, 1936: 84, 209 [N]. Type species: *Anatrumsongoricum* Reichardt, 1936, by original designation. Status: valid genus in Blaptinae: Opatrini: Opatrina.

*Anausis* Bates, 1873e: 355 [F]. Type species: *Anausismacleayi* Bates, 1873, by monotypy. Status: junior synonym of *Cyphaleus* Westwood, 1841 in Tenebrioninae: Heleini: Cyphaleina. Synonymy: Matthews (1992: 490).

*Anaxius* Fåhraeus, 1870: 307 [M]. Type species: *Anaxiusobesus* Fåhraeus, 1870, by monotypy. Status: valid genus in Blaptinae: Pedinini: Helopinina.

*Anaxo* Bates, 1868: 272 [F]. Type species: *Anaxobrevicornis* Bates, 1868, by monotypy. Status: junior synonym of *Lepturidea* Fauvel, 1862 in Alleculinae: Alleculini: Alleculina. Synonymy: Matthews and Bouchard (2008: 324, with *Aethyssius* Pascoe, 1863, a junior synonym of *Lepturidea* Fauvel, 1862).

*Anchomma* J.L. LeConte, 1858a: 63 [N]. Type species: *Anchommacostatum* J.L. LeConte, 1858, by monotypy. Status: valid genus in Pimeliinae: Stenosini: Dichillina. Note: **new placement** [RLA], previously included in Pimeliinae: Anepsiini; genus originally described in Tenebrionoidea: Zopheridae: Colydiinae.

*Anchophthalmops* Koch, 1956a: 173 [M]. Type species: *Anchophthalmopsbrevipleurum* Koch, 1956, by original designation. Status: valid genus in Blaptinae: Platynotini: Platynotina.

*Anchophthalmus* Gerstaecker, 1854: 533 [M]. Type species: *Anchophthalmussilphoides* Gerstaecker, 1854, by subsequent designation (Gebien 1938a: 298). Status: valid genus and subgenus in Blaptinae: Platynotini: Platynotina.

*Ancylopoma* Pascoe, 1871: 354 [N]. Type species: *Ancylopomapunctigerum* Pascoe, 1871, by monotypy. Status: valid genus in Tenebrionidae: incertae sedis. Note: see Bouchard and Bousquet (2020a: 102) for comments regarding the placement of this genus and its associated family-group name.

*Andocamaria* Masumoto, 1993b: 230, 232 [F]. Type species: *Campsiomorphaformosana* Pic, 1930, by original designation. Status: valid genus in Stenochiinae: Cnodalonini.

*Andremiopsis* Chatanay, 1913a: 406 [F]. Type species: *Andremiopsiscostata* Chatanay, 1913, by monotypy. Status: valid genus in Pimeliinae: Asidini.

*Andremius* Fairmaire, 1903b: 364 [M]. Type species: *Andremiuscrispatus* Fairmaire, 1903, by monotypy. Status: valid genus in Pimeliinae: Asidini.

*Andrimus* Casey, 1891: 155 [M]. Type species: *Cteniopusmurrayi* J.L. LeConte, 1866, by subsequent designation (R. Lucas 1920: 96). Status: valid genus in Alleculinae: Alleculini: Gonoderina.

*Androchirus* J.L. LeConte, 1862: 244 [M]. Type species: *Cistelafuscipes* Melsheimer, 1846 (= *Cistelaerythropa* W. Kirby, 1837), by original designation. Status: valid genus in Alleculinae: Alleculini: Gonoderina.

*Androsus* Gebien, 1921a: 325, 386 [M]. Type species: *Chariothecaviolacea* Pascoe, 1887, by original designation. Status: valid genus in Stenochiinae: Cnodalonini.

*Anebacis* Peyerimhoff, 1927: 4, 15 [F]. Type species: *Tentyriacribricollis* Fairmaire, 1875, by subsequent designation (Löbl et al. 2008a: 40). Status: valid subgenus of *Pachychila* Eschscholtz, 1831 in Pimeliinae: Tentyriini.

*Anectus* Horn, 1867: 399 [M]. Type species: *Anectusvestitus* Horn, 1867, by monotypy. Status: valid genus in Pimeliinae: Branchini.

*Anemia* Laporte, 1840: 218 [F]. Type species: *Anemiagranulata* Laporte, 1840, by monotypy. Status: junior synonym of *Cheirodes* Gené, 1839 in Tenebrioninae: Melanimonini. Synonymy: Marseul (1863: 183); Spilman (1973: 41).

*Anemiadena* Bouchard & Bousquet, **new subgenus** [F]. Type species: *Anemiaconvexa* Gestro, 1881, by **present designation**. Status: valid subgenus of *Cheirodes* Gené, 1839 in Tenebrioninae: Melanimonini. Note: Ardoin (1971: 362, 402) introduced the new subgenus name *Anemiadena* for two nominal species, but unfortunately did not designate a type species; the fact that *Anemiaconvexa* Gestro, 1881 was listed as the “type” of *Anemiadena* in the Zoological Record for the year 1971 (Anonymous in Commonwealth Institute of Entomology 1975) does not represent a valid type species designation since the nomenclatural act is anonymous (ICZN 1999, Article 14); the subgenusAnemiadena, which has been treated as valid since 1971, is therefore unavailable (ICZN 1999, Article 13.3); we hereby make the name available by selecting *Anemiaconvexa* Gestro, 1881 as type species and referring to Ardoin (1971: 362) for the character states that characterise and differentiate *Anemiadena*.

*Anephyctus* Fairmaire, 1891b: 257 [M]. Type species: *Anephyctushirtulus* Fairmaire, 1891, by monotypy. Status: junior synonym of *Miltoprepes* Gerstaecker, 1871 in Tenebrioninae: Praeugenini. Synonymy: Gridelli (1939: 78, through placement of the type species in *Miltoprepes* Gerstaecker, 1871).

*Anepsius* J.L. LeConte, 1851: 147 [M]. Type species: *Anepsiusdelicatulus* J.L. LeConte, 1851, by monotypy. Status: valid genus in Pimeliinae: Anepsiini.

*Anethas* Jakobson, 1924: 242 [M]. Type species [automatic]: *Pseudethaslongiceps* Fairmaire, 1898, by monotypy. Status: valid genus and subgenus in Pimeliinae: Stenosini: Stenosina. Note: replacement name for *Pseudethas* Fairmaire, 1898.

*Angoleantus* Koch, 1952a: 128, 133 [M]. Type species: *Rhammatodesstriatulus* Koch, 1941, by original designation. Status: valid subgenus of *Rhammatodes* Haag-Rutenberg, 1876 in Pimeliinae: Tentyriini.

*Angolositus* Koch, 1955c: 448 [M]. Type species: *Angolositussadabandeirus* Koch, 1955, by original designation. Status: valid genus in Blaptinae: Platynotini: Platynotina.

*Aniara* Melsheimer, 1853: 139 [F]. Type species: *Ulomapiceum* Melsheimer, 1846, by monotypy. Status: senior synonym of *Eutochia* J.L. LeConte, 1862 in Tenebrioninae: Ulomini. Note: name previously attributed to “Lacordaire, 1859” in the literature; junior homonym of *Aniara* Hope, 1838 [Coleoptera: Carabidae].

*Aniarus* Gemminger in Gemminger and Harold, 1870: 1964 [M]. Type species [automatic]: *Ulomapiceum* Melsheimer, 1846, by monotypy. Status: junior synonym of *Eutochia* J.L. LeConte, 1862 in Tenebrioninae: Ulomini. Note: unjustified emendation of *Aniara* Melsheimer, 1853, not in prevailing usage.

*Anisocara* Gebien, 1925e: 101 [N]. Type species: *Anisocaragynandromorphum* Gebien, 1925, by monotypy. Status: junior synonym of *Platydema* Laporte & Brullé, 1831 in Diaperinae: Diaperini: Diaperina. Synonymy: Schawaller (2004: 5).

*Anisocerus* Faldermann, 1837: 39 [M]. Type species: *Anisocerustristis* Faldermann, 1837, by monotypy. Status: senior synonym of *Ceratanisus* Gemminger, 1870 in Pimeliinae: Ceratanisini. Note: junior homonym of *Anisocerus* Audinet-Serville, 1835 [Coleoptera: Cerambycidae].

*Anisophaedis* Ando, 1993: 107 [M]. Type species: *Anisophaedisohkurai* Ando, 1993, by original designation. Status: valid genus in Stenochiinae: Cnodalonini.

*Anisosis* Deyrolle, 1867: 81, 232 [F]. Type species: *Anisosiscaudata* Deyrolle, 1867, by monotypy. Status: valid subgenus of *Zophosis* Latreille, 1802 in Pimeliinae: Zophosini. Note: the alternative original spelling *Urosis*, used by Deyrolle (1867: 81), was rejected by Dallas (1868: 265–266) who acted as First Reviser.

*Anisostira* Borchmann, 1915: 296 [F]. Type species: *Anisostiravaricolor* Borchmann, 1915 (= *Macrolagriarugipennis* Lewis, 1896), by original designation. Status: valid genus in Lagriinae: Lagriini: Statirina.

*Annamosdara* Kaszab, 1941a: 3, 30 [F]. Type species: *Annamosdaramultidentata* Kaszab, 1941, by original designation. Status: valid genus in Stenochiinae: Cnodalonini.

*Anobriomaia* Kaszab, 1941b: 67 [F]. Type species: *Anobriomaiasulcata* Kaszab, 1941, by original designation. Status: junior synonym of *Foochounus* Pic, 1921 in Stenochiinae: Cnodalonini. Synonymy: Kaszab (1983a: 134).

*Anodesis* Solier, 1834: 508, 594 [F]. Type species: *Anodesiscleryi* Solier, 1834, by monotypy. Status: valid genus in Pimeliinae: Erodiini.

*Anognathena* Ando in Ando et al., 2017: 148 [F]. Type species: *Anognathenaneraida* Ando, 2017, by original designation. Status: valid genus in Alleculinae: Alleculini: Alleculina.

*Anomalipus* Guérin-Méneville, 1831b: pl. 29 [M]. Type species: *Blapsdentipes* Fabricius, 1794, by monotypy. Status: valid genus in Blaptinae: Platynotini: Platynotina. Note: nomen protectum (see Bouchard and Bousquet 2020a: 101, 132); unjustified emendation of the original spelling *Anomalipes*, introduced by Lacordaire (1859a: 257), in prevailing usage and treated as a justified emendation (ICZN 1999, Article 33.2.3.1), see Bouchard et al. (2005: 511).

*Anommabates* Koch, 1956b: 84 [M]. Type species: *Anommabatespauliani* Koch, 1956, by original designation. Status: valid genus in Diaperinae: Gnathidiini: Gnathidiina.

*Anomoearthrum* Mäklin, 1867: 482 [N]. Type species: *Anomoearthrumdebile* Mäklin, 1867, by subsequent designation (Gebien 1948: 540). Status: junior synonym of *Strongylium* W. Kirby, 1819 in Stenochiinae: Stenochiini. Synonymy: Gemminger in Gemminger and Harold (1870: 2028).

*Anophthalmolamus* Ferrer, 1993: 122 [M]. Type species: *Anophthalmolamusfuerteventurae* Ferrer, 1993, by original designation. Status: valid genus in Tenebrioninae: incertae sedis.

*Anopidium* Jeannel & Paulian, 1945: 63 [N]. Type species: *Anopidiumelgonicum* Jeannel & Paulian, 1945, by original designation. Status: valid genus in Diaperinae: Gnathidiini: Anopidiina.Note: originally described in Tenebrionoidea: Zopheridae: Colydiinae.

*Anotoma* Borchmann, 1936: 18, 81 [F]. Type species: *Chrysolagriaheynei* Borchmann, 1915, by original designation. Status: valid genus in Lagriinae: Lagriini: Lagriina.

*Antennoluprops* Schawaller, 2007: 30 [M]. Type species: *Antennolupropsbremeri* Schawaller, 2007, by original designation. Status: valid genus in Lagriinae: Lupropini.

*Anteros* Laporte, 1840: 235 [M]. Type species: *Helopschalibeus* Rossi, 1790 (= *Tenebriocaeruleus* Linnaeus, 1758), by subsequent designation (Iablokoff-Khnzorian 1964: 303). Status: junior synonym of *Helops* Fabricius, 1775 in Tenebrioninae: Helopini: Helopina. Synonymy: Erichson in Agassiz (1846a: 12). Note: junior homonym of *Anteros* Hübner, 1819 [Lepidoptera].

*Anthracias* Dejean, 1834: 205 [M]. Type species: *Ulomacornutum* Fischer, 1823, by monotypy. Status: junior synonym of *Cryphaeus* Klug, 1833 in Tenebrioninae: Toxicini: Toxicina. Synonymy: Gebien (1921b: 238).

†*Anthracohelops* Haupt, 1950: 114, 128 [M]. Type species: *Anthracohelopsgigas* Haupt, 1950, by original designation. Status: valid genus in Stenochiinae: incertae sedis. Note: described from Middle Eocene deposits (Germany).

*Anthracosomus* Agassiz, 1846b: 26 [M]. Type species [automatic]: *Anthrasomuschevrolatii* Guérin-Méneville, 1834, by monotypy. Status: junior synonym of *Anthrasomus* Guérin-Méneville, 1834 in Pimeliinae: Praociini. Note: unjustified emendation of *Anthrasomus* Guérin-Méneville, 1834, not in prevailing usage.

*Anthracula* Fairmaire, 1897c: 236 [F]. Type species: *Anthraculalatifrons* Fairmaire, 1897, by monotypy. Status: valid genus in Alleculinae: Alleculini: Alleculina.

*Anthrasomus* Guérin-Méneville, 1834: 32 [M]. Type species: *Anthrasomuschevrolatii* Guérin-Méneville, 1834, by monotypy. Status: valid subgenus of *Praocis* Eschscholtz, 1829 in Pimeliinae: Praociini.

*Anthrenopsis* Koch, 1950c: 74 [F]. Type species: *Platydemascriptipennis* Fairmaire, 1875 (= *Basidesziczac* Motschulsky, 1873), by original designation. Status: valid subgenus of *Ellipsodes* Wollaston, 1854 in Diaperinae: Crypticini.

*Anticlia* Gistel, 1848a: x, 125 [F]. Type species [automatic]: *Opatrumorientale* Fabricius, 1775, by subsequent designation (Hope 1841: 110). Status: junior synonym of *Sclerum* Dejean, 1834 in Blaptinae: Opatrini: Sclerina. Note: unnecessary replacement name for *Sclerum* Dejean, 1834.

*Antimachus* Gistel, 1829: 1055 [M]. Type species: *Phalariafurcifera* Dalman, 1821, by monotypy. Status: valid genus in Tenebrioninae: Ulomini.

*Antofagapraocis* Flores, 2000b: 62, 68 [M]. Type species: *Falsopraocissubnudus* Kulzer, 1959, by original designation. Status: valid genus in Pimeliinae: Praociini.

*Antoineius* Koch, 1948: 418 [M]. Type species: *Micrositusjeanneli* Koch, 1945 (= *Melambiusinermis* Antoine, 1942), by original designation. Status: valid subgenus of *Otinia* Antoine, 1942 in Blaptinae: Dendarini: Melambiina.

*Aoupinia* Matthews, 2003a: 441 [F]. Type species: *Aoupiniapseudohelea* Matthews, 2003, by original designation. Status: valid genus in Lagriinae: Adeliini.

*Apalmia* Fairmaire, 1896a: 60 [F]. Type species: *Apalmiacerambycina* Fairmaire, 1896, by monotypy. Status: valid genus in Alleculinae: incertae sedis.

*Apasis* Pascoe, 1869: 139 [F]. Type species: *Apasishowittii* Pascoe, 1869, by monotypy. Status: valid genus in Lagriinae: Adeliini.

*Apatelus* Mulsant & Rey, 1859a: 88, 91 [M]. Type species: *Apatelushopei* Mulsant & Rey, 1859, by monotypy. Status: junior synonym of *Isopteron* Hope, 1841 in Lagriinae: Adeliini. Synonymy: Carter (1926: 123).

*Apatopsis* Semenov, 1891: 368 [F]. Type species: *Apatopsisgrombczewskii* Semenov, 1891, by subsequent designation (R. Lucas 1920: 107). Status: valid genus in Pimeliinae: Pimeliini.

*Apelina* Saha, 1988: 429 [F]. Type species: *Apelinakeralaensis* Saha, 1988, by original designation. Status: junior synonym of *Amarygmus* Dalman, 1823 in Tenebrioninae: Amarygmini. Synonymy: Bremer and Lillig (2014: 12).

*Apellatus* Pascoe, 1863a: 45 [M]. Type species: *Apellatuslateralis* Pascoe, 1863 (= *Apellatusamoenus* Pascoe, 1866), by monotypy. Status: junior synonym of *Euomma* Boheman, 1858 in Alleculinae: Alleculini: Alleculina. Synonymy: Pascoe (1866a: 491).

*Apentanes* Reitter, 1914a: 45, 51 [M]. Type species: *Arthrodeisoccidentalis* Fairmaire, 1868, by subsequent designation (Gebien 1937a: 536). Status: valid subgenus of *Arthrodeis* Solier, 1834 in Pimeliinae: Erodiini.

*Apentanodes* Reitter, 1914a: 46, 53 [M]. Type species: *Arthrodeisglobosus* Reiche & Saulcy, 1857, by subsequent designation (Gebien 1937a: 539). Status: valid genus and subgenus in Pimeliinae: Erodiini.

*Aphaleria* Reitter, 1896a: 235 [F]. Type species: *Aphaleriacapnisoides* Reitter, 1896 (= *Erodiuspygmaeus* Fischer, 1821), by monotypy. Status: junior synonym of *Bradyus* Dejean, 1834 in Tenebrioninae: Dissonomini. Synonymy: Löbl et al. (2008b: 240).

*Aphanaspis* Wollaston, 1864: 478 [F]. Type species: *Pimeliagranulicollis* Wollaston, 1864, by subsequent designation (Löbl et al. 2008a: 40). Status: valid subgenus of *Pimelia* Fabricius, 1775 in Pimeliinae: Pimeliini.

*Aphanotus* J.L. LeConte, 1862: 233 [M]. Type species: *Eulabisbrevicornis* J.L. LeConte, 1859, by original designation. Status: valid subgenus of *Tribolium* W.S. MacLeay, 1825 in Tenebrioninae: Triboliini.

*Aphectus* Carter, 1926: 127, 128 [M]. Type species [automatic]: *Hectusanthracinus* Pascoe, 1869, by monotypy. Status: junior synonym of *Olisthaena* Erichson, 1842 in Tenebrioninae: Heleini: Cyphaleina. Note: unnecessary replacement name for *Hectus* Pascoe, 1869.

*Aphelus* Gebien, 1921b: 62, 78 [M]. Type species: *Aphelussimplicicollis* Gebien, 1921, by subsequent designation (Gebien 1941: 341). Status: junior synonym of *Argobrachium* Fairmaire, 1899 in Stenochiinae: Cnodalonini. Synonymy: Ardoin (1961b: 31).

*Aphrotus* Péringuey, 1904: 252 [M]. Type species [automatic]: *Xenustricorniger* Péringuey, 1899, by monotypy. Status: valid genus in Pimeliinae: Tentyriini. Note: replacement name for *Xenus* Péringuey, 1899.

*Aphtora* Bates, 1872b: 265 [F]. Type species: *Aphtorarufipes* Bates, 1872, by monotypy. Status: valid genus in Phrenapatinae: incertae sedis. Note: Matthews and Lawrence (2019: 623) placed this genus in the subfamily Phrenapatinae (tribal position uncertain).

*Aphyllocerus* Fairmaire, 1881a: 348 [M]. Type species: *Aphyllocerusdecipiens* Fairmaire, 1881, by monotypy. Status: junior synonym of *Amarygmus* Dalman, 1823 in Tenebrioninae: Amarygmini. Synonymy: Gebien (1921a: 409).

*Apistocerus* Fairmaire, 1899a: 78 [M]. Type species: *Apistoceruswasmanni* Fairmaire, 1899, by monotypy. Status: valid subgenus of *Rhyzodina* Chevrolat, 1873 in Tenebrioninae: Rhysopaussini.

*Apithesis* C.O. Waterhouse, 1881: 476 [F]. Type species: *Apithesisobesa* C.O. Waterhouse, 1881, by monotypy. Status: junior synonym of *Clitobius* Mulsant & Rey, 1859 in Blaptinae: Opatrini: Ammobiina. Synonymy: Purchart and Kamiński (2017: 143).

*Aplanasida* Reitter, 1917a: 11, 30 [F]. Type species: *Asidabrevicosta* Solier, 1836, by monotypy. Status: junior synonym of *Glabrasida* Escalera, 1910 in Pimeliinae: Asidini. Synonymy: Viñolas and Cartagena (2005: 282).

*Apocrypha* Eschscholtz, 1831: 13 [F]. Type species: *Apocryphaanthicoides* Eschscholtz, 1831, by monotypy. Status: valid genus in Tenebrioninae: Apocryphini.

*Apocryphodes* Matthews, 1998: 704, 765 [M]. Type species: *Apocryphodesthompsoni* Matthews, 1998, by original designation. Status: valid genus in Lagriinae: Adeliini.

*Apodemus* Fåhraeus, 1870: 293 [M]. Type species: *Anomalipusplanus* Fåhraeus, 1870, by subsequent designation (Iwan 2002c: 234). Status: junior synonym of *Anomalipus* Guérin-Méneville, 1831 in Blaptinae: Platynotini: Platynotina. Synonymy: Gebien (1938a: 409). Note: junior homonym of *Apodemus* Kaup, 1829 [Mammalia].

*Apolites* Jacquelin du Val, 1861: 324 [M]. Type species: *Helopsmucoreus* Waltl, 1838, by monotypy. Status: senior synonym of *Ceratanisus* Gemminger, 1870 in Pimeliinae: Ceratanisini. Synonymy: Allard (1876b: ciii). Note: junior homonym of *Apolites* Sundevall, 1835 [Aves].

*Apomestris* Bates, 1873e: 357 [M]. Type species: *Apomestriswestwoodi* Bates, 1873, by monotypy. Status: junior synonym of *Cyphaleus* Westwood, 1841 in Tenebrioninae: Heleini: Cyphaleina. Synonymy: Carter (1926: 119, with *Altes* Pascoe, 1869, a junior synonym of *Cyphaleus* Westwood, 1841).

*Apostethus* Pascoe, 1882: 27 [M]. Type species: *Apostethusterrenus* Pascoe, 1882, by monotypy. Status: junior synonym of *Adelodemus* Haag-Rutenberg, 1878 in Lagriinae: Adeliini. Synonymy: Carter (1926: 131).

*Apristopus* Kolbe, 1903: 167 [M]. Type species: *Priosceliscrassicornis* Westwood, 1842, by monotypy. Status: junior synonym of *Calostegia* Westwood, 1843 in Lagriinae: Pycnocerini. Synonymy: Gebien (1904a: 169).

*Aprosphaena* Reitter, 1916c: 140, 142 [F]. Type species: *Aprosphaenaadriani* Reitter, 1916 (= *Tageniastriatopunctata* Wiedemann, 1821), by subsequent designation (Löbl et al. 2008a: 40). Status: junior synonym of *Stenosida* Solier, 1835 in Pimeliinae: Tentyriini. Synonymy: Blair (1935a: 103).

*Apsena* J.L. LeConte, 1862: 228 [F]. Type species: *Eulabispubescens* J.L. LeConte, 1851, by original designation. Status: valid genus in Tenebrioninae: Eulabini.

*Apsheronellus* Bogatchev, 1967: 157 [M]. Type species: *Apsheronellusarenarius* Bogatchev, 1967, by original designation. Status: valid genus in Blaptinae: Pedinini: Leichenina.

*Apsida* Lacordaire, 1859a: 309 [F]. Type species: *Apsidachrysomelina* Lacordaire, 1859, by original designation. Status: valid genus in Stenochiinae: Cnodalonini.

*Aptereucyrtus* Gebien, 1922a: 477 [M]. Type species: *Aptereucyrtushemichalceus* Gebien, 1922, by monotypy. Status: valid genus in Stenochiinae: Cnodalonini.

*Aptereutochia* Kaszab, 1980a: 190 [F]. Type species: *Eutochiaaptera* Kaszab, 1980, by original designation. Status: valid subgenus of *Cenoscelis* Wollaston, 1868 in Tenebrioninae: Ulomini. Note: this name was first published by Kaszab (1979a: 92) without a description, a definition, or a bibliographic reference to such a published statement (ICZN 1999, Article (13.1) and is therefore unavailable from that date.

*Aptericula* Borchmann, 1937: 219 [F]. Type species: *Aptericulanyassensis* Borchmann, 1937, by original designation. Status: valid genus in Alleculinae: incertae sedis.

*Apterobrachys* Kaszab, 1986: 295 [M]. Type species: *Apterobrachyswilhelminae* Kaszab, 1986, by original designation. Status: valid genus in Stenochiinae: Cnodalonini.

*Apteroclitobius* Koch, 1960: 391, 404 [M]. Type species: *Clitobiusgrandis* Fairmaire, 1896, by original designation. Status: junior synonym of *Clitobius* Mulsant & Rey, 1859 in Blaptinae: Opatrini: Ammobiina. Synonymy: Purchart and Kamiński (2017: 143).

*Apterocyphostethe* Kaszab, 1962a: 302 [F]. Type species: *Himatismuskoltzei* Reitter, 1895, by original designation. Status: valid subgenus of *Cyphostethe* Marseul, 1866 in Pimeliinae: Tentyriini.

*Apterogena* Ardoin, 1962a: 67 [F]. Type species: *Apterogenacanonnei* Ardoin, 1962, by original designation. Status: senior synonym of *Genateropa* Bouchard & Bousquet, **nom. nov.** in Stenochiinae: Stenochiini. Note: junior homonym of *Apterogena* Amyot, 1847 [Hemiptera].

*Apteroleprocaulus* Kaszab, 1983c: 182 [M]. Type species: *Leprocaulusmontanus* Kaszab, 1982, by original designation. Status: junior synonym of *Hexarhopalus* Fairmaire, 1891 in Stenochiinae: Cnodalonini. Synonymy: Bečvář and Purchart (2008: 39).

*Apteromaia* Kulzer, 1952: 719 [F]. Type species: *Eucyrtusovipennis* Gebien, 1913, by original designation. Status: valid genus in Stenochiinae: Cnodalonini.

*Apteromerus* Blair, 1928: 72 [M]. Type species: *Opatrinusconvexus* Fairmaire, 1849, by monotypy. Status: valid genus in Stenochiinae: Cnodalonini.

*Apteromira* Weise, 1974: 123 [F]. Type species: *Cistelaovulum* Kiesenwetter, 1863, by original designation. Status: valid subgenus of *Isomira* Mulsant, 1856 in Alleculinae: Alleculini: Gonoderina.

*Apteronympha* Seidlitz, 1898b: 336, 338 [F]. Type species: *Lagriarubida* Graells, 1858, by subsequent designation (Merkl 2004: 284). Status: valid subgenus of *Lagria* Fabricius, 1775 in Lagriinae: Lagriini: Lagriina.

*Apterophenus* Gebien, 1921a: 325, 342 [M]. Type species: *Apterophenusevanescens* Gebien, 1921, by subsequent designation (Gebien 1941: 1142). Status: valid genus in Stenochiinae: Cnodalonini.

*Apteroseriscius* Koch, 1950c: 64 [M]. Type species: *Pseudoserisciusespanoli* Koch, 1950, by original designation. Status: valid genus in Diaperinae: Crypticini.

*Apterosphaeria* Koch, 1950b: 298 [F]. Type species: *Derosphaeriusrugifrons* Fairmaire, 1888, by original designation. Status: valid subgenus of *Derosphaerius* Westwood, 1881 in Pimeliinae: Tentyriini.

*Apterotarpela* Kaszab, 1954: 262 [F]. Type species: *Apterotarpelaklapperichi* Kaszab, 1954, by original designation. Status: valid genus in Tenebrioninae: Helopini: Helopina.

*Apterotheca* Gebien, 1921a: 348 [F]. Type species: *Chariothecabesti* Blackburn, 1894, by subsequent designation (Gebien 1942a: 338). Status: valid genus in Stenochiinae: Cnodalonini.

*Apterozidalus* Ardoin, 1965b: 1315 [M]. Type species: *Apterozidalusroyi* Ardoin, 1965, by original designation. Status: junior synonym of *Zidalus* Mulsant & Rey, 1853 in Blaptinae: Platynotini: Platynotina. Synonymy: Iwan (1995b: 362).

*Apteruleda* Gebien, 1928: 134 [F]. Type species: *Apteruledauncipes* Gebien, 1928, by original designation. Status: valid genus in Tenebrioninae: Ulomini.

*Apteruloma* Gebien, 1928: 149 [N]. Type species: *Apterulomamagnum* Gebien, 1928, by original designation. Status: valid genus in Tenebrioninae: Ulomini.

*Apterulomoides* Kaszab, 1982d: 279 [M]. Type species: *Ulomarotundipenne* Kaszab, 1982, by monotypy. Status: valid subgenus of *Uloma* Dejean, 1821 in Tenebrioninae: Ulomini.

*Aptila* Fåhraeus, 1870: 258 [F]. Type species: *Aptilacostata* Fåhraeus, 1870, by subsequent designation (R. Lucas 1920: 115). Status: valid genus in Blaptinae: Pedinini: Helopinina.

*Arabammobius* Grimm & Lillig, 2020: 307 [M]. Type species: *Ammobiustarsalis* Grimm, 2012, by original designation. Status: valid genus in Blaptinae: Opatrini: Ammobiina.

*Arabcynaeus* Schawaller, 2009a: 164 [M]. Type species: *Arabcynaeusbremeri* Schawaller, 2009, by original designation. Status: valid genus in Diaperinae: Diaperini: Adelinina.

*Araeopselaphus* Gebien, 1921b: 10 [M]. Type species: *Araeopselaphusmyrmecophilus* Gebien, 1921, by monotypy. Status: valid genus in Diaperinae: Crypticini.

*Araeoschizus* J.L. LeConte, 1851: 138 [M]. Type species: *Araeoschizuscostipennis* J.L. LeConte, 1851, by monotypy. Status: valid genus in Pimeliinae: Stenosini: Araeoschizina.

*Araucaricola* Lea, 1929: 218 [F]. Type species: *Araucaricolaebenina* Lea, 1929, by monotypy. Status: junior synonym of *Iscanus* Fauvel, 1904 in Lagriinae: Lupropini. Synonymy: Kaszab (1982a: 59).

*Archaeoglenes* Broun, 1893a: 188 [M]. Type species: *Archaeoglenescostipennis* Broun, 1893, by monotypy. Status: valid genus in Phrenapatinae: Archaeoglenini.

*Archasida* Wilke, 1922: 261 [F]. Type species: *Afrasidainnotata* Wilke, 1922, by original designation. Status: valid subgenus of *Afrasida* Wilke, 1922 in Pimeliinae: Asidini.

*Archeophthora* Kaszab, 1978a: 166 [F]. Type species: *Brachycilibetasmanicum* Carter, 1919, by original designation. Status: valid genus in Phrenapatinae: Penetini.

*Archinamaqua* Schawaller, 2012b: 80 [F]. Type species: *Archinamaqualyleae* Schawaller, 2012 (= *Menederopsisconstricta* Koch, 1954), by original designation. Status: junior synonym of *Menederopsis* Koch, 1954 in Blaptinae: Platynotini: Eurynotina. Synonymy: Kamiński and Schawaller (2019: 293).

*Archinamibia* Koch, 1952a: 157 [F]. Type species: *Archinamibiapeezi* Koch, 1952, by original designation. Status: valid genus in Pimeliinae: Tentyriini.

*Arcothymus* Pascoe, 1866a: 476 [M]. Type species: *Arcothymuscoenosus* Pascoe, 1866 (= *Adeliumtriste* Montrouzier, 1860), by monotypy. Status: valid genus in Lagriinae: Adeliini. Note: as mentioned by Matthews and Bouchard (2008: 350) the type species was originally described from Australia in error; the genus *Arcothymus* Pascoe, 1866 is endemic to New Caledonia.

*Arctylus* Dejean, 1834: 180 [M]. Type species: *Praocispentagonus* Lacordaire, 1830, by monotypy. Status: junior synonym of *Praocis* Eschscholtz, 1829 in Pimeliinae: Praociini. Synonymy: Bousquet and Bouchard (2013a: 43).

*Ardamimicus* Smith, 2013: 601 [M]. Type species: *Ardamimicuscognatoi* Smith, 2013, by original designation. Status: valid genus in Pimeliinae: Asidini.

*Ardeleodes* Blaisdell, 1937: 128 [M]. Type species: *Eleodestibialis* Blaisdell, 1909, by original designation. Status: valid subgenus of *Eleodes* Eschscholtz, 1829 in Blaptinae: Amphidorini. Note: the alternative original spelling *Arpeleodes*, used by Blaisdell (1937: 128), was rejected by Gebien (1938a: 63) who acted as First Reviser.

*Ardelio* Gistel, 1848a: xi [M]. Type species [automatic]: *Ulomacornutum* Fischer, 1823, by monotypy. Status: junior synonym of *Cryphaeus* Klug, 1833 in Tenebrioninae: Toxicini: Toxicina. Note: unnecessary replacement name for *Anthracias* Dejean, 1834.

*Ardoinia* Kaszab, 1969a: 249 [F]. Type species: *Ardoiniadiaclinoides* Kaszab, 1969, by original designation. Status: valid genus in Tenebrioninae: Alphitobiini.

*Ardoinia* Özdikmen, 2005: 202 [F]. Type species [automatic]: *Orghidaniatorrei* Ardoin, 1977, by monotypy. Status: senior synonym of *Spelaebiosis* Bousquet & Bouchard, 2018 in Tenebrioninae: Triboliini. Note: replacement name for *Orghidania* Ardoin, 1977; junior homonym of *Ardoinia* Kaszab, 1969 [Coleoptera: Tenebrionidae: Tenebrioninae: Alphitobiini].

*Ardoiniellus* Schawaller, 2013a: 138 [M]. Type species: *Ardoiniellusmontanus* Schawaller, 2013, by original designation. Status: valid genus in Lagriinae: Lupropini.

*Arenacara* Penrith, 1979: 43, 46 [N]. Type species: *Stenocarabrunnipes* Haag-Rutenberg, 1877, by original designation. Status: valid subgenus of *Stenocara* Solier, 1835 in Pimeliinae: Adesmiini.

*Arenoblaps* G.S. Medvedev, 1999b: 400 [F]. Type species: *Blaps hiemalis* Semenov-Tjan-Shansky & Bogatchev, 1940, by original designation. Status: valid subgenus of *Blaps* Fabricius, 1775 in Blaptinae: Blaptini: Blaptina.

*Argasidus* Péringuey, 1899: 251 [M]. Type species: *Argasidussquamosus* Péringuey, 1899, by monotypy. Status: valid genus in Pimeliinae: Adelostomini.

*Argenticrinis* Louw, 1979: 99, 100 [M]. Type species: *Argenticrinishaackei* Louw, 1979 (= *Psammodeslossowi* Koch, 1952), by original designation. Status: valid genus in Pimeliinae: Sepidiini: Hypomelina.

*Argobrachium* Fairmaire, 1899d: 216 [N]. Type species: *Argobrachiumimpressifrons* Fairmaire, 1899, by monotypy. Status: valid genus in Stenochiinae: Cnodalonini.

*Argoporis* Horn, 1870: 325 [F]. Type species: *Cerenopuscostipennis* J.L. LeConte, 1851, by subsequent designation (Gebien 1937a: 797). Status: valid genus in Tenebrioninae: Cerenopini. Note: as pointed out by Bousquet et al. (2018: 97, 179), evidence shows that *Threnus* Motschulsky, 1870 was published before the currently accepted valid name *Argoporis* Horn, 1870; as *Threnus* Motschulsky, 1870 was used as valid after 1899 (e.g., Leng 1920: 224), reversal of precedence cannot be used to conserve usage of *Argoporis* Horn, 1870; an application to the ICZN is necessary to conserve usage of *Argoporis* Horn, 1870.

*Argutiolana* Robiche, 2001: 191 [F]. Type species: *Argutiolanamaguini* Robiche, 2001, by monotypy. Status: valid genus in Stenochiinae: Cnodalonini. Note: transferred from Tenebrioninae: Tenebrionini by Robiche (2019a: 97).

*Argyradelpha* G.S. Medvedev, 2005a: 306 [F]. Type species: *Argyradelphalopatini* G.S. Medvedev, 2005, by original designation. Status: valid genus in Pimeliinae: Pimeliini.

*Argyrophana* Semenov, 1889: 222, 224 [F]. Type species: *Argyrophanadeserti* Semenov, 1889, by monotypy. Status: valid genus in Pimeliinae: Pimeliini.

*Ariarathus* Fairmaire, 1891e: ccxi [M]. Type species: *Ariarathusulomoides* Fairmaire, 1891 (= *Tenebrioatronitens* Fairmaire, 1891), by monotypy. Status: valid genus in Tenebrioninae: Tenebrionini.

*Armalia* Casey, 1907: 289, 330 [F]. Type species: *Emmenastustexanus* J.L. LeConte, 1866, by original designation. Status: valid genus in Pimeliinae: Edrotini.

*Armenohelops* Nabozhenko, 2002a: 42 [M]. Type species: *Armenohelopsarmeniacus* Nabozhenko, 2002, by original designation. Status: valid genus in Tenebrioninae: Helopini: Cylindrinotina.

*Armigena* Bouchard & Bousquet, **new subgenus** [F]. Type species: *Nesogenatestaceipes* Fairmaire, 1868, by **present designation**. Status: valid subgenus of *Nesogena* Mäklin, 1863 in Tenebrioninae: Praeugenini. Note: first proposed by Froussart (1961: 60) without type species designation; the subgenusArmigena, which is currently used as valid, is therefore unavailable (ICZN 1999, Articles 13.3, 16.1); we hereby make the name available by selecting *Nesogenatestaceipes* Fairmaire, 1868 as type species and referring to Froussart (1961: 60) for the character states that characterise and differentiate *Armigena*.

*Arnoldiola* Semenov-Tjan-Shansky & Bogatchev, 1940: 201 [F]. Type species: *Arnoldiolapeculiaris* Semenov-Tjan-Shansky & Bogatchev, 1940 (= *Stalagmopteraruginota* Reitter, 1896), by original designation. Status: junior synonym of *Stalagmoptera* Solsky, 1876 in Pimeliinae: Pimeliini. Synonymy: Löbl et al. (2008b: 168). Note: junior homonym of *Arnoldiola* Strand, 1928 [Diptera].

*Arrhabaeus* Champion, 1886: 144 [M]. Type species: *Arrhabaeusconvexus* Champion, 1886, by monotypy. Status: junior synonym of *Dioedus* J.L. LeConte, 1862 in Phrenapatinae: Penetini. Synonymy: Kaszab (1977a: 314).

*Arrhenoplita* W. Kirby, 1837: 235 [F]. Type species: *Ips haemorrhoidalis* Fabricius, 1787, by original designation. Status: junior synonym of *Neomida* Latreille, 1829 in Diaperinae: Diaperini: Diaperina. Synonymy: Duponchel and Chevrolat (1841: 157).

*Artactes* Pascoe, 1868: xii [M]. Type species: *Artactesnigritarsis* Pascoe, 1868, by monotypy. Status: valid genus in Stenochiinae: Cnodalonini.

*Arthrochora* Gebien, 1938b: 75 [F]. Type species: *Arthrochoraarenicola* Gebien, 1938, by monotypy. Status: valid genus in Pimeliinae: Adelostomini.

*Arthroconus* Solier, 1851: 238 [M]. Type species: *Arthroconuspiceus* Solier, 1851 (= *Gymnognathusfuscus* Solier, 1851), by subsequent designation (Lacordaire 1859a: 67). Status: valid genus in Pimeliinae: Edrotini. Note: the First Reviser (*Arthroconus* Solier, 1851 versus *Gymnognathus* Solier, 1851) is Lacordaire (1859a: 67).

*Arthrodeis* Solier, 1834: 508, 513 [M]. Type species: *Arthrodeisrotundatus* Solier, 1834, by subsequent designation (Hope 1841: 114). Status: valid genus and subgenus in Pimeliinae: Erodiini.

*Arthrodes* Agassiz, 1846b: 35 [M]. Type species [automatic]: *Arthrodeisrotundatus* Solier, 1834, by subsequent designation (Hope 1841: 114). Status: junior synonym of *Arthrodeis* Solier, 1834 in Pimeliinae: Erodiini. Note: unjustified emendation of *Arthrodeis* Solier, 1834, not in prevailing usage.

*Arthrodibius* Lesne, 1915: 227, 235 [M]. Type species: *Arthrodeislaxepunctatus* Fairmaire, 1884, by subsequent designation (Sharp 1919: 119). Status: valid genus and subgenus in Pimeliinae: Erodiini.

*Arthrodinus* Reitter, 1900b: 299 [M]. Type species: *Erodiusobesus* Brullé, 1839, by subsequent designation (Reitter 1914a: 46). Status: valid subgenus of *Arthrodeis* Solier, 1834 in Pimeliinae: Erodiini.

*Arthrodion* Lesne, 1915: 227, 234 [N]. Type species: *Spyrathusafricanus* Fairmaire, 1882, by monotypy. Status: valid genus in Pimeliinae: Erodiini.

*Arthrodosis* Reitter, 1900b: 299 [F]. Type species: *Erodiusglobosus* Faldermann, 1837, by subsequent designation (R. Lucas 1920: 119). Status: valid genus in Pimeliinae: Erodiini. Note: the original combination of the accepted name of the type species, *Erodiusglobosus* Faldermann, 1837, is a junior primary homonym of *Erodiusglobosus* Thunberg, 1787.

*Arthrodygmus* Reitter, 1914a: 46 [M]. Type species: *Arthrodygmusfieberi* Reitter, 1914, by monotypy. Status: valid genus in Pimeliinae: Erodiini.

*Arthrohyalosis* Kaszab, 1979a: 75 [F]. Type species: *Arthrodosismostofii* Pierre, 1975, by original designation. Status: valid genus in Pimeliinae: Erodiini.

*Arthrohyalus* Koch, 1943a: 490, 503 [M]. Type species: *Arthrohyalussarcinipennis* Koch, 1943, by monotypy. Status: valid genus in Pimeliinae: Erodiini.

*Arthromacra* W. Kirby, 1837: 238 [F]. Type species: *Arthromacradonacioides* W. Kirby, 1837 (= *Lagriaaenea* Say, 1824), by monotypy. Status: valid genus in Lagriinae: Lagriini: Statirina.

*Arthroplatus* Solier, 1851: 246 [M]. Type species: *Arthroplatuspallipes* Solier, 1851, by monotypy. Status: junior synonym of *Acropteryx* Gistel, 1831 in Tenebrioninae: Acropteronini. Synonymy: Schaum (1852: 183, with *Acropteron* Perty, 1832, a junior synonym of *Acropteryx* Gistel, 1831).

*Arturium* Koch, 1951: 83 [N]. Type species: *Melanolophusater* C.O. Waterhouse, 1885, by original designation. Status: valid genus in Pimeliinae: Sepidiini: Molurina.

*Artystona* Bates, 1874: 104 [F]. Type species: *Titaenaerichsonii* White, 1846, by subsequent designation (R. Lucas 1920: 120). Status: valid genus in Tenebrioninae: Titaenini. Note: this name was introduced one year earlier by Bates (1873f: 473) but it is unavailable from that date since there is no description nor originally included species (ICZN 1999, Article 12.1).

*Arunogria* Merkl, 1991: 14 [F]. Type species: *Arunogriapubescens* Merkl, 1991, by original designation. Status: valid genus in Lagriinae: Lagriini: Statirina.

*Aryenis* Bates, 1868: 309 [F]. Type species: *Aryenisrufescens* Bates, 1868 (= *Statiraunicolor* Blanchard, 1843), by monotypy. Status: valid genus in Pimeliinae: Evaniosomini.

*Asbolius* Fairmaire, 1902a: 134 [M]. Type species: *Asboliusquadricollis* Fairmaire, 1902, by monotypy. Status: junior synonym of *Rhammatodes* Haag-Rutenberg, 1876 in Pimeliinae: Tentyriini. Synonymy: Blair (1935a: 103).

*Asbolodes* Fairmaire, 1892c: 52 [M]. Type species: *Asbolodeshumerosus* Fairmaire, 1892, by monotypy. Status: valid genus in Stenochiinae: Cnodalonini.

*Asbolodomimus* Pic, 1921d: 20 [M]. Type species: *Asbolodomimussubcarinatus* Pic, 1921, by monotypy. Status: valid genus in Stenochiinae: Cnodalonini.

*Asbolus* J.L. LeConte, 1851: 129 [M]. Type species: *Asbolusverrucosus* J.L. LeConte, 1851, by subsequent designation (Aalbu 2005: 721). Status: valid genus in Pimeliinae: Cryptoglossini. Note: the older name *Asbolus* Voet, 1793 [Coleoptera: Silphidae] was published in a work that did not include consistent application of binominal nomenclature and is therefore unavailable (ICZN 1999, Article 11.4).

*Ascalabus* Fairmaire, 1893b: 30 [M]. Type species: *Ascalabuspedinoides* Fairmaire, 1893, by monotypy. Status: senior synonym of *Calabosca* Fairmaire, 1894 in Stenochiinae: Cnodalonini. Note: junior homonym of *Ascalabus* Agassiz, 1846 [Pisces].

*Ascelosodis* Redtenbacher, 1868: 117 [F]. Type species: *Ascelosodisserripes* Redtenbacher, 1868, by monotypy. Status: valid genus in Pimeliinae: Edrotini.

*Asemogena* Péringuey, 1904: 281 [F]. Type species: *Asemogenasimplex* Péringuey, 1904, by subsequent designation (R. Lucas 1920: 121). Status: valid genus in Stenochiinae: Stenochiini.

*Asialassus* Nabozhenko & Ando, 2018: 311 [M]. Type species: *Helopscordicollis* Marseul, 1876, by original designation. Status: valid genus in Tenebrioninae: Helopini: Cylindrinotina.

*Asida* Latreille, 1802: 167 [F]. Type species: *Opatrumgriseum* Fabricius, 1781, by monotypy. Status: valid genus and subgenus in Pimeliinae: Asidini.

*Asidelia* Fairmaire, 1905: 296 [F]. Type species: *Asideliacontracta* Fairmaire, 1905, by monotypy. Status: valid genus in Pimeliinae: Praociini.

*Asidesthes* Fairmaire, 1900c: 246 [F]. Type species: *Asidesthesperrieri* Fairmaire, 1900, by monotypy. Status: valid genus in Pimeliinae: Asidini.

*Asididius* Fairmaire, 1869b: 236 [M]. Type species: *Asididiuscoquerelii* Fairmaire, 1869, by monotypy. Status: valid genus in Stenochiinae: Cnodalonini.

*Asidina* Casey, 1912: 76, 169 [F]. Type species: *Pelecyphorusparallelus* J.L. LeConte, 1851, by original designation. Status: valid subgenus of *Stenomorpha* Solier, 1836 in Pimeliinae: Asidini.

*Asidoblaps* Fairmaire, 1886d: 342 [F]. Type species: *Asidoblapsdavidis* Fairmaire, 1886, by subsequent designation (R. Lucas 1920: 122). Status: valid genus in Blaptinae: Blaptini: Gnaptorinina.

*Asidobothris* Fairmaire, 1886c: 72 [F]. Type species: *Asidobothrisclathrata* Fairmaire, 1886, by monotypy. Status: valid genus in Stenochiinae: Cnodalonini.

*Asidodema* Koch, 1958: 139 [F]. Type species: *Oncosomaalternicoste* Gebien, 1910, by original designation. Status: valid genus in Blaptinae: Pedinini: Helopinina.

*Asidomachla* Wilke, 1922: 262 [F]. Type species: *Asidabicostata* Fåhraeus, 1870, by original designation. Status: junior synonym of *Machlomorpha* Péringuey, 1899 in Pimeliinae: Asidini. Synonymy: Koch (1962a: 125).

*Asidomorpha* Koch, 1962a: 121 [F]. Type species: *Afrasidaprona* Wilke, 1922, by original designation. Status: valid genus in Pimeliinae: Asidini.

*Asidopsis* Casey, 1912: 77, 185 [F]. Type species: *Asidaopaca* Say, 1824, by original designation. Status: valid subgenus of *Stenomorpha* Solier, 1836 in Pimeliinae: Asidini.

*Asiobirus* G.S. Medvedev, 1968a: 170, 179 [M]. Type species: *Cabirusvalidipes* Reitter, 1891, by original designation. Status: junior synonym of *Cabirutus* Strand, 1929 in Blaptinae: Pedinini: Pedinina. Synonymy: Kamiński and Iwan (2017: 595).

*Asiocaedius* G.S. Medvedev & Nepesova, 1985: 138 [M]. Type species [automatic]: *Pseudocaediuskiseritzkii* G.S. Medvedev, 1966, by original designation. Status: valid genus in Blaptinae: Opatrini: Ammobiina. Note: replacement name for *Pseudocaedius* G.S. Medvedev, 1966.

*Asiomira* Dubrovina, 1973: 367 [F]. Type species: *Isomiraophthalmica* Seidlitz, 1896, by original designation. Status: valid genus in Alleculinae: Alleculini: Gonoderina. Note: changed from subgenus of *Isomira* Mulsant, 1856 to valid genus by Novák (2016a: 179).

*Asiopus* Sharp, 1892b: 43 [M]. Type species: *Asiopusopatroides* Sharp, 1892, by monotypy. Status: valid genus in Lagiinae: incertae sedis. Note: this little-known genus has been treated as a member of the subfamily Lagriinae (e.g., Fery 2013: 66).

*Asiris* Motschulsky, 1872: 24 [F]. Type species: *Asirisangulicollis* Motschulsky, 1872 (= *Tenebrioaustralis* Boisduval, 1835), by original designation. Status: junior synonym of *Meneristes* Pascoe, 1869 in Tenebrioninae: Heleini: Asphalina. Synonymy: C.O. Waterhouse (1876: 288).

*Asopidiopsis* Kaszab, 1955a: 511, 515 [F]. Type species: *Asopidiopsisovalis* Kaszab, 1955, by original designation. Status: valid genus in Stenochiinae: Cnodalonini.

*Asopis* Haag-Rutenberg, 1878: 104 [F]. Type species: *Asopissuavis* Haag-Rutenberg, 1878, by monotypy. Status: valid genus in Stenochiinae: Cnodalonini.

*Asphaltesthes* Kraatz, 1865: 80, 181 [F]. Type species: *Mesostenacostata* Erichson, 1843, by monotypy. Status: valid genus and subgenus in Pimeliinae: Tentyriini.

*Asphalus* Pascoe, 1868: xii [M]. Type species: *Asphalusebeninus* Pascoe, 1868, by monotypy. Status: valid genus in Tenebrioninae: Heleini: Asphalina.

*Asphena* Semenov, 1889: 218 [F]. Type species: *Asphenakomarowi* Semenov, 1889, by monotypy. Status: junior synonym of *Cyphostethe* Marseul, 1866 in Pimeliinae: Tentyriini. Synonymy: Reitter (1916c: 141).

*Aspidius* Mulsant & Rey, 1859: 123 [M]. Type species: *Blapspunctata* Fabricius, 1792, by subsequent designation (Bousquet et al. 2018: 195). Status: junior synonym of *Blapstinus* Dejean, 1821 in Blaptinae: Opatrini: Blapstinina. Synonymy: Champion (1885: 124).

*Aspidocephalus* Motschulsky, 1839: 63 [M]. Type species: *Aspidocephalusdesertus* Motschulsky, 1839, by monotypy. Status: valid genus in Pimeliinae: Stenosini: Dichillina. Note: unjustified emendation of the original spelling *Aspicephalus*, introduced by Lacordaire (1859a: 107), in prevailing usage and treated as a justified emendation (ICZN 1999, Article 33.2.3.1).

*Aspidolobus* Redtenbacher, 1868: 118 [M]. Type species: *Aspidolobuspiliger* Redtenbacher, 1868, by monotypy. Status: valid genus in Pimeliinae: Epitragini.

*Aspidosoma* Agassiz, 1846b: 36, 37 [N]. Type species [automatic]: *Aspisomafulvipenne* Duponchel & Chevrolat, 1841, by original designation. Status: junior synonym of *Anaedus* Blanchard, 1842 in Lagriinae: Goniaderini. Note: unjustified emendation of *Aspisoma* Duponchel & Chevrolat, 1841, not in prevailing usage.

*Aspidosternum* Mäklin, 1867: 500 [N]. Type species: *Tenebriocyaneus* Fabricius, 1794, by monotypy. Status: junior synonym of *Metallonotus* Gray, 1832 in Lagriinae: Pycnocerini. Synonymy: Alluaud (1889: xlvi).

*Aspila* Fåhraeus, 1870: 251 [F]. Type species: *Aspilabicostata* Fåhraeus, 1870, by monotypy. Status: senior synonym of *Prunaspila* Koch, 1950 in Pimeliinae: Adelostomini. Note: junior homonym of *Aspila* Stephens, 1834 [Lepidoptera].

*Aspilomorpha* Koch, 1952b: 33, 101 [F]. Type species: *Aspilomorphamediolobata* Koch, 1952, by original designation. Status: valid genus in Pimeliinae: Adelostomini.

*Aspisoma* Duponchel & Chevrolat, 1841: 240 [N]. Type species: *Aspisomafulvipenne* Duponchel & Chevrolat, 1841, by original designation. Status: senior synonym of *Anaedus* Blanchard, 1842 in Lagriinae: Goniaderini. Synonymy: Lacordaire (1859a: 396). Note: junior homonym of *Aspisoma* Laporte, 1833 [Coleoptera: Lampyridae].

*Astalbus* Fairmaire, 1900d: 484 [M]. Type species: *Astalbusscrobicollis* Fairmaire, 1900, by monotypy. Status: valid genus in Tenebrioninae: Palorini.

*Astathmetus* Bates, 1874: 23 [M]. Type species: *Astathmetusalienus* Bates, 1874, by monotypy. Status: valid genus in Stenochiinae: Cnodalonini. Note: this name was introduced one year earlier by Bates (1873f: 472) but it is unavailable from that date since there is no description nor originally included species (ICZN 1999, Article 12.1).

*Astatira* Borchmann, 1921: 218, 322 [F]. Type species: *Astatirahumeralis* Borchmann, 1921, by original designation. Status: valid genus in Lagriinae: Lagriini: Statirina.

*Asthenochirus* Fairmaire, 1885a: viii [M]. Type species: *Asthenochirusnigropunctatus* Fairmaire, 1885, by subsequent designation (R. Lucas 1920: 123). Status: valid genus in Tenebrioninae: Amarygmini. Note: redescribed as new by Fairmaire (1887b: 298).

*Asthenopoda* Chatanay, 1915a: 527, 544 [F]. Type species: *Asthenopodafragilis* Chatanay, 1915, by original designation. Status: junior synonym of *Psilonesogena* Bates, 1879 in Stenochiinae: Stenochiini. Synonymy: De Moor (1970: 9).

*Asticostena* Fairmaire, 1897c: 228 [F]. Type species: *Asticostenaalternata* Fairmaire, 1897, by monotypy. Status: valid genus in Alleculinae: incertae sedis.

*Astorthocnemis* Lillig & Pavlíček, 2002: 98 [F]. Type species: *Storthocnemissaudita* Koch, 1965, by original designation. Status: valid genus in Pimeliinae: Pimeliini.

*Astrotus* J.L. LeConte, 1858b: 19 [M]. Type species: *Microschatiacontorta* J.L. LeConte, 1853, by original designation. Status: valid subgenus of *Pelecyphorus* Solier, 1836 in Pimeliinae: Asidini.

*Asyleptus* Péringuey, 1896: 179 [M]. Type species: *Asyleptusfumosus* Péringuey, 1896, by monotypy. Status: valid genus in Tenebrioninae: Amarygmini.

*Asyrmatus* Canzoneri, 1959: 149 [M]. Type species: *Helopspiceus* Sturm, 1826, by original designation. Status: junior synonym of *Pystelops* Gozis, 1910 in Tenebrioninae: Helopini: Cylindrinotina. Synonymy: **new synonym** [YB]. Note: *Pystelops* Gozis, 1910 has been forgotten in the literature; its type species is currently included in the subgenusAsyrmatus Canzoneri, 1959 and for that reason Canzoneri’s name is considered a junior synonym of *Pystelops*; the original combination of the accepted name of the type species, *Helopspiceus* Sturm, 1826, is a junior primary homonym of *Helopspiceus* G.-A. Olivier, 1792.

*Atahualpina* Español, 1960: 113 [F]. Type species: *Atahualpinaperuviana* Español, 1960 (= *Phaleriasubparalella* Chevrolat, 1878), by original designation. Status: junior synonym of *Phaleria* Latreille, 1802 in Diaperinae: Phaleriini. Synonymy: Triplehorn (1991: 258).

*Atasthalomorpha* Miyatake, 1964: 68, 77 [F]. Type species: *Atasthalusdentifrons* Lewis, 1894, by original designation. Status: valid genus in Tenebrioninae: Bolitophagini.

*Atasthalus* Pascoe, 1871: 348 [M]. Type species: *Atasthalusspectrum* Pascoe, 1871, by monotypy. Status: valid genus in Tenebrioninae: Bolitophagini.

*Athrodactyla* Klug, 1833: 90 [F]. Type species: *Athrodactylaelongata* Klug, 1833, by subsequent designation (Hope 1841: 126). Status: valid genus in Tenebrioninae: Tenebrionini.

*Atlasion* Koch, 1948: 427 [N]. Type species: *Micrositusbedeli* Escalera, 1914, by original designation. Status: valid subgenus of *Hoplarion* Mulsant & Rey, 1854 in Blaptinae: Dendarini: Melambiina. Note: the First Reviser (*Atlasion* Koch, 1948 versus *Megatlasion* Koch, 1948) is Antoine (1957: 351).

*Atlasotaurus* Bouyon, 2011: 464 [M]. Type species: *Omophlusmaroccanus* P.H. Lucas, 1846, by original designation. Status: valid subgenus of *Heliotaurus* Mulsant, 1856 in Alleculinae: Cteniopodini.

*Atoichus* Carter, 1915a: 72 [M]. Type species: *Licymniusbicolor* Blackburn, 1893, by original designation. Status: valid genus in Alleculinae: Alleculini: Alleculina.

*Atoreuma* Gebien, 1941: 1132 [N]. Type species [automatic]: *Toreumacupreum* Carter, 1913, by monotypy. Status: valid genus in Tenebrioninae: Heleini: Cyphaleina. Note: replacement name for *Eutoreuma* Carter, 1914.

*Atrachyderma* Skopin, 1962: 228 [N]. Type species: *Pimeliasetosa* Faldermann, 1832, by original designation. Status: valid subgenus of *Trachyderma* Latreille, 1828 in Pimeliinae: Pimeliini. Note: *Pimeliasetosa*, the type species, has been attributed to Fischer in the literature; the species was described as “Pimeliasetosa Fald.” which, from the context, indicates that Faldermann was responsible for the description of the species.

*Atractus* Boisduval, 1835: 283 [M]. Type species: *Atractusviridis* Boisduval, 1835, by subsequent designation (Duponchel and Chevrolat 1841: 312). Status: senior synonym of *Lepturidea* Fauvel, 1862 in Alleculinae: Alleculini: Alleculina. Synonymy: Matthews and Bouchard (2008: 324). Note: junior homonym of *Atractus* Wagler, 1828 [Reptilia].

*Atrocrates* Koch, 1956a: 82 [M]. Type species: *Trigonopusplatyderus* Mulsant & Rey, 1853, by original designation. Status: valid genus in Blaptinae: Platynotini: Platynotina.

*Atrocrypticanus* Iwan, 1999a: 73 [M]. Type species: *Atrocrypticanusfraternus* Iwan, 1999, by original designation. Status: valid genus in Blaptinae: Platynotini: Platynotina.

*Atropsorodes* Ardoin, 1963a: 88 [M]. Type species: *Atropsorodesbarnardi* Ardoin, 1963, by original designation. Status: valid genus in Tenebrioninae: Amarygmini. Note: name published earlier by Ardoin (1962b: 969) without fixation of a type species in the original publication (ICZN 1999, Article 13.3) and is therefore unavailable from that date.

*Atryphodes* Pascoe, 1866a: 478 [M]. Type species [automatic]: *Thoracophoruswalckenaerii* Hope, 1841, by original designation. Status: junior synonym of *Cardiothorax* Motschulsky, 1860 in Lagriinae: Adeliini. Note: unnecessary replacement name for *Thoracophorus* Hope, 1841.

*Auchmobius* J.L. LeConte, 1851: 139 [M]. Type species: *Auchmobiussublaevis* J.L. LeConte, 1851, by monotypy. Status: valid genus in Pimeliinae: Edrotini.

*Augolesthus* Motschulsky, 1872: 34 [M]. Type species: *Augolesthuspurpureofasciatus* Motschulsky, 1872, by original designation. Status: valid genus in Stenochiinae: Cnodalonini.

*Aulacodera* Agassiz, 1846b: 40, 41 [F]. Type species [automatic]: *Nycteliacrenicosta* Guérin-Méneville, 1834, by subsequent designation (Gebien 1937a: 752). Status: junior synonym of *Auladera* Solier, 1836 in Pimeliinae: Nycteliini. Note: unjustified emendation of *Auladera* Solier, 1836, not in prevailing usage.

*Aulacus* Gray in Griffith and Pidgeon, 1832: 783 [M]. Type species: *Aulacuschilensis* Gray, 1832, by monotypy. Status: senior synonym of *Orthogonoderes* Solier, 1841 in Pimeliinae: Praociini. Synonymy: Kulzer (1958a: 81). Note: junior homonym of *Aulacus* Jurine, 1807 [Hymenoptera].

*Auladera* Solier, 1836: 307, 331 [F]. Type species: *Nycteliacrenicosta* Guérin-Méneville, 1834, by subsequent designation (Gebien 1937a: 752). Status: valid genus in Pimeliinae: Nycteliini.

*Aulonasida* Reitter, 1917a: 9, 19 [F]. Type species: *Asidachauveneti* Solier, 1836, by subsequent designation (F. Soldati 2008: 32). Status: junior synonym of *Glabrasida* Escalera, 1910 in Pimeliinae: Asidini. Synonymy: Viñolas and Cartagena (2005: 282).

*Aulonogria* Borchmann, 1929a: 9 [F]. Type species: *Lagriarugosa* Fabricius, 1801, by original designation. Status: valid genus in Lagriinae: Lagriini: Lagriina. Note: also published as new in Borchmann (1930a: 405, 424).

*Aulonolcus* Reitter, 1904: 168 [M]. Type species: *Pedinusaltaicus* Gebler, 1830, by monotypy. Status: valid subgenus of *Penthicus* Faldermann, 1836 in Blaptinae: Opatrini: Opatrina.

*Aulonoscelis* Reitter, 1896c: 173 [F]. Type species: *Platyscelishauseri* Reitter, 1895 (= *Dilaphilacoides* Fischer von Waldheim, 1844), by original designation. Status: junior synonym of *Gebleria* Motschulsky, 1846 in Blaptinae: Blaptini: Prosodina. Synonymy: G.S. Medvedev (2005b: 99).

*Auristira* Borchmann, 1916a: 47, 129 [F]. Type species: *Auristiraoctocostata* Borchmann, 1916, by **present designation**. Status: junior synonym of *Costiferolagria* Pic, 1915 in Lagriinae: Lagriini: Lagriina. Synonymy: Borchmann (1936: 176).

*Australoseriscius* Koch, 1950c: 65 [M]. Type species: *Crypticusexplorator* Gebien, 1920, by original designation. Status: valid subgenus of *Pseudoseriscius* Español, 1950 in Diaperinae: Crypticini.

*Austrocaribius* Marcuzzi, 1954: 18 [M]. Type species: *Austrocaribiusvenezuelensis* Marcuzzi, 1954, by monotypy. Status: valid genus in Blaptinae: Opatrini: Blapstinina.

*Austropalorus* Halstead, 1967a: 72, 129 [M]. Type species: *Austropalorusplanatus* Halstead, 1967, by original designation. Status: valid genus in Tenebrioninae: Palorini.

*Austropeus* Carter, 1924b: 543 [M]. Type species: *Austropeuspustulosus* Carter, 1924, by monotypy. Status: junior synonym of *Apterotheca* Gebien, 1921 in Stenochiinae: Cnodalonini. Synonymy: Bouchard (2002: 452).

*Austroptorina* Bai, Li & Ren, 2020: 166 [F]. Type species: *Gnaptorinalongicornis* Li & Ren, 2004, by original designation. Status: valid subgenus of *Gnaptorina* Reitter, 1887 in Blaptinae: Blaptini: Gnaptorinina.

*Autocera* Wollaston, 1857: 154 [F]. Type species: *Autoceralaticeps* Wollaston, 1857, by monotypy. Status: junior synonym of *Cnemeplatia* Costa, 1847 in Pimeliinae: Cnemeplatiini: Cnemeplatiina. Synonymy: Kraatz (1859: 75).

*Axumia* Reiche, 1850: pl. 22 [F]. Type species: *Axumiapraelonga* Reiche, 1850, by monotypy. Status: junior synonym of *Rhytinota* Eschscholtz, 1831 in Pimeliinae: Tentyriini. Synonymy: Kraatz (1865: 170).

*Axynaon* Blackburn, 1897a: 34 [N]. Type species: *Axynaonchampioni* Blackburn, 1897, by monotypy. Status: valid genus in Tenebrioninae: Amarygmini.

*Azarelius* Fairmaire, 1892d: vii [M]. Type species: *Azareliussculpticollis* Fairmaire, 1892, by monotypy. Status: valid genus in Tenebrioninae: Amarygmini. Note: transferred from Rhysopaussini to Amarygmini by Löbl and Smetana (2010: 34).

*Azonoderus* Harold, 1879: 125 [M]. Type species: *Azonoderustristis* Harold, 1879, by monotypy. Status: valid genus in Stenochiinae: Stenochiini.

*Balachowskya* Peyerimhoff, 1928: 61 [F]. Type species: *Balachowskyaportentosa* Peyerimhoff, 1928, by monotypy. Status: valid genus in Pimeliinae: Pimeliini.

*Balassogloa* Semenov, 1891: 372 [F]. Type species: *Balassogloasphenarioides* Semenov, 1891, by subsequent designation (R. Lucas 1920: 131). Status: valid genus in Alleculinae: Cteniopodini.

*Balius* Gistel, 1848a: xi, xiv [M]. Type species [automatic]: *Pimeliasimplex* Solier, 1836, by monotypy. Status: junior synonym of *Melanostola* Solier, 1836 in Pimeliinae: Pimeliini. Note: unnecessary replacement name for *Melanostola* Solier, 1836.

*Bancocistela* Pic, 1947: 151 [F]. Type species: *Bancocistelaivoirensis* Pic, 1947, by monotypy. Status: valid genus in Alleculinae: incertae sedis.

*Bantodemus* Koch, 1955c: 428 [M]. Type species: *Trigonopuslethaeus* Mulsant & Rey, 1853, by original designation. Status: valid genus in Blaptinae: Platynotini: Platynotina.

*Baratus* Fairmaire, 1897c: 233 [M]. Type species: *Baratuscrenulatus* Fairmaire, 1897, by monotypy. Status: valid genus in Stenochiinae: Cnodalonini.

*Barbora* Novák, 2020b: 462 [F]. Type species: *Barboracastanea* Novák, 2020, by original designation. Status: valid genus in Alleculinae: Alleculini: Alleculina.

*Barlacus* Fairmaire, 1900b: 45 [M]. Type species: *Barlacuscostulatus* Fairmaire, 1900 (= *Asyleptusfumosus* Péringuey, 1896), by monotypy. Status: junior synonym of *Asyleptus* Péringuey, 1896 in Tenebrioninae: Amarygmini. Synonymy: Ardoin (1962b: 957); Bremer (2013a: 77).

*Barsenis* Pascoe, 1887: 17 [F]. Type species: *Barsenisfulvipes* Pascoe, 1887, by monotypy. Status: valid genus and subgenus in Lagriinae: Lagriini: Statirina.

*Bartolozzia* Ferrer, 1998a: 369 [F]. Type species: *Bartolozziahispida* Ferrer, 1998, by original designation. Status: valid genus in Pimeliinae: Asidini.

*Barycistela* Blackburn, 1891: 327 [F]. Type species: *Barycistelarobusta* Blackburn, 1891, by monotypy. Status: valid genus in Alleculinae: Alleculini: Alleculina.

*Baryscelis* Boisduval, 1835: 253 [F]. Type species: *Baryscelislaticollis* Boisduval, 1835, by subsequent designation (Hope 1841: 126). Status: valid genus in Tenebrionidae: incertae sedis. Note: the type material of the two species included in this genus has not been located (Matthews and Bouchard 2008: 350) and therefore the identity of the genus is uncertain.

*Barytipha* Pascoe, 1869: 288, 292 [F]. Type species: *Barytiphasocialis* Pascoe, 1869, by monotypy. Status: junior synonym of *Pterohelaeus* Brême, 1842 in Tenebrioninae: Heleini: Heleina. Synonymy: Matthews (1993: 1041).

*Basanaedus* Pic, 1917d: 11 [M]. Type species: *Basanaedusluteomaculatus* Pic, 1917, by subsequent designation (Gebien 1941: 820). Status: junior synonym of *Lyprochelyda* Fairmaire, 1899 in Lagriinae: Goniaderini. Synonymy: Ardoin (1969a: 166).

*Basanopsis* Gebien, 1914a: 21 [F]. Type species: *Basanopsiscurvipes* Gebien, 1914, by monotypy. Status: valid genus in Tenebrioninae: Ulomini.

*Basanus* Lacordaire, 1859a: 306 [M]. Type species: *Basanusforticornis* Lacordaire, 1859, by monotypy. Status: valid genus in Diaperinae: Scaphidemini. Note: combined description of a new genus and single new species (ICZN 1999, Article 12.2.6).

*Basides* Motschulsky, 1873: 471 [M]. Type species: *Basidesbifasciatus* Motschulsky, 1873, by subsequent designation (Gebien 1925d: 560). Status: valid genus in Diaperinae: Diaperini: Diaperina. Note: *Basides* Motschulsky, 1873 was used as valid after 1899 (e.g., Gebien, 1911a: 381) and therefore reversal of precedence cannot be used to conserve usage of *Ischnodactylus* Chevrolat, 1877.

*Basilewskyum* Koch, 1952b: 30, 94 [N]. Type species: *Basilewskyumstenosinoide* Koch, 1952, by original designation. Status: valid genus in Pimeliinae: Adelostomini.

*Bassianus* Matthews & Doyen, 1989: 44 [M]. Type species: *Tenebriocolydioides* Erichson, 1842, by original designation. Status: valid genus in Tenebrioninae: Heleini: Asphalina.

*Batessia* Ponting, 2018: 131 [F]. Type species [automatic]: *Agastheneswestwoodi* Bates, 1873, by monotypy. Status: junior synonym of *Agastenes* R. Lucas, 1920 in Tenebrioninae: Heleini: Cyphaleina. Note: unnecessary replacement name for *Agasthenes* Bates, 1873.

*Batuliodes* Casey, 1907: 499 [M]. Type species: *Batuliusrotundicollis* J.L. LeConte, 1851, by original designation. Status: valid genus in Pimeliinae: Anepsiini.

*Batuliomorpha* Doyen, 1987: 359 [F]. Type species: *Batuliomorphacomata* Doyen, 1987, by original designation. Status: valid genus in Pimeliinae: Anepsiini.

*Batulius* J.L. LeConte, 1851: 148 [M]. Type species: *Batuliussetosus* J.L. LeConte, 1851, by subsequent designation (Casey 1907: 497). Status: valid genus in Pimeliinae: Anepsiini.

*Bearnicistela* Pic in E. Olivier and Pic, 1909: 139 [F]. Type species: *Bearnicistelaluteicolor* Pic, 1909, by monotypy. Status: valid genus in Alleculinae: incertae sedis.

*Bechuanitis* Koch, 1955a: 93 [F]. Type species: *Trachynotusbrucki* Haag-Rutenberg, 1873, by original designation. Status: valid subgenus of *Somaticus* Hope, 1841 in Pimeliinae: Sepidiini: Trachynotina.

*Becvaramarygmus* Masumoto, 1999c: 369 [M]. Type species: **fixed herein** (ICZN 1999, Article 70.3) as *Dietysusnodicornis* Gravely, 1915, misidentified as *Dietysusatricolor* Pic, 1922 in the original designation in Masumoto (1999). Status: valid subgenus of *Amarygmus* Dalman, 1823 in Tenebrioninae: Amarygmini. Note: the type species “*Dietysusatricolor* Pic” was first established by original designation; Bremer and Lillig (2014: 12, 49) first noted that *Dietysusatricolor* Pic of Masumoto (1999) was identical to *Dietysusnodicornis* Gravely, 1915; we follow the concept of Bremer and Lillig (2014: 12, 49) and fix the type species according to the requirements of Article 70.3.2 (ICZN 1999); the nominal species *Dietysusatricolor* Pic, 1922 is a junior synonym of *Amarygmusfilicornis* (Gravely, 1915); Iwan et al. (2020: 262) erroneously placed *Becvaramarygmus* Masumoto, 1999 as a junior synonym of the subgenusAmarygmus Dalman, 1823 (M. Lillig and H. Bremer, pers. comm. 2020).

*Becvarius* Masumoto, 1998: 207 [M]. Type species: *Becvariusstanislavi* Masumoto, 1998, by original designation. Status: valid genus in Stenochiinae: Cnodalonini.

*Bellendenum* Matthews, 1998: 704, 794 [N]. Type species: *Bellendenumgonyxuthum* Matthews, 1998, by original designation. Status: valid genus in Lagriinae: Adeliini.

*Belopomerus* Reitter, 1920a: 3 [M]. Type species: *Calcarzoufali* Reitter, 1915, by monotypy. Status: valid subgenus of *Centorus* Mulsant, 1854 in Lagriinae: Belopini.

*Belopus* Gebien, 1911a: 459 [M]. Type species [automatic]: *Tenebrioelongatus* Herbst, 1797, by monotypy. Status: valid subgenus of *Centorus* Mulsant, 1854 in Lagriinae: Belopini. Note: replacement name for *Calcar* Dejean, 1821; nomen protectum (see Bouchard and Bousquet 2020b: 6).

*Belousovia* G.S. Medvedev, 2007a: 157 [F]. Type species: *Belousoviahelenae* G.S. Medvedev, 2007, by original designation. Status: valid genus in Blaptinae: Blaptini: Gnaptorinina.

*Belutschistanops* Löbl, Bouchard, Merkl & Bousquet, 2020: 3 [M]. Type species: *Hyperopsschusteri* Koch, 1940, by original designation. Status: valid subgenus of *Hyperops* Eschscholtz, 1831 in Pimeliinae: Tentyriini. Note: name first proposed by Koch (1943a: 523, 552) without a type species originally included (ICZN 1999, Article 13.3); Löbl et al. (2008a: 40) designated *Hyperopsschusteri* Koch, 1940 as the type species of Koch’s name but did not explicitly indicate the genus-group name as intentionally new (ICZN 1999, Article 16.1).

*Bequaertiella* Pic, 1914a: 486 [F]. Type species: *Bequaertiellacoerulescens* Pic, 1914, by monotypy. Status: valid genus in Lagriinae: Lagriini: Lagriina.

*Bermejoina* Español, 1944: 12 [F]. Type species: *Bermejoinaaiunica* Español, 1944, by original designation. Status: valid genus in Blaptinae: Dendarini: Melambiina.

*Betasida* Reitter, 1917a: 8, 11 [F]. Type species: *Asidaluctuosa* Boisduval, 1835, by subsequent designation (Viñolas and Cartagena 2005: 110). Status: valid subgenus of *Alphasida* Escalera, 1905 in Pimeliinae: Asidini. Note: some authors (e.g., F. Soldati 2008: 32) have used *Asidaargenteolimbata* Escalera, 1901 as type species for this genus; however, the older designation by Viñolas and Cartagena (2005: 110) should stand since *Asidaluctuosa* Boisduval, 1835 was one of the two originally included nominal species in *Betasida*.

*Betschia* Dajoz, 1980: 135 [F]. Type species: *Betschiaminuta* Dajoz, 1980, by original designation. Status: valid genus in Diaperinae: Gnathidiini: incertae sedis. Note: transferred from Tenebrionoidea: Zopheridae (as “Colydiidae”) by Ivie and Ślipiński (1990: 18); placed in “Gnathidiini incertae sedis” by Schawaller and Purchart (2012: 312).

*Bia* Hope, 1841: 132 [F]. Type species [automatic]: *Trogossitathoracica* Fabricius, 1792, by monotypy. Status: junior synonym of *Bius* Dejean, 1834 in Tenebrioninae: Tenebrionini. Note: unjustified emendation of *Bius* Dejean, 1834, not in prevailing usage.

*Bielawskia* Marcuzzi, 1985: 179 [F]. Type species: *Bielawskiacubana* Marcuzzi, 1985 (= *Trimytantrondecui* Ardoin, 1977), by monotypy. Status: junior synonym of *Trimytantron* Ardoin, 1977 in Pimeliinae: Edrotini. Synonymy: Marcuzzi (1998: 153).

*Biolus* Mulsant & Rey, 1854: 25 [M]. Type species: *Eurynotusasperipennis* Mulsant & Rey, 1854 (= *Helopsgranulatus* Fabricius, 1787), by subsequent designation (Koch 1953a: 276). Status: valid subgenus of *Eurynotus* W. Kirby, 1819 in Blaptinae: Platynotini: Eurynotina.

*Biomorphus* Motschulsky, 1872: 38 [M]. Type species: *Biomorphustuberculatus* Motschulsky, 1872 (= *Amphidoraattenuata* J.L. LeConte, 1851), by original designation. Status: junior synonym of *Helops* Fabricius, 1775 in Tenebrioninae: Helopini: Helopina. Synonymy: Aalbu et al. (1995: 485).

*Bionesus* Fairmaire, 1879b: 70 [M]. Type species: *Bionesuscinereosparsus* Fairmaire, 1879, by monotypy. Status: valid genus in Stenochiinae: Stenochiini.

*Bioplanes* Mulsant, 1854: 144 [M]. Type species: *Bioplanesmeridionalis* Mulsant, 1854, by monotypy. Status: valid genus in Blaptinae: Dendarini: Dendarina.

*Bioramix* Bates, 1879b: 478 [M]. Type species: *Bioramixovalis* Bates, 1879, by subsequent designation (Kaszab 1940a: 175). Status: valid genus and subgenus in Blaptinae: Platyscelidini. Note: nomenclatural stability in this genus is threatened by the discovery of an older type species designation (*Bioramixpamirensis* Bates, 1879, by subsequent designation by R. Lucas (1920: 138), which is currently included in the valid subgenusPlanoplatyscelis Kaszab, 1940); we recommend that an application be submitted to the International Commission on Zoological Nomenclature to maintain the type species designation proposed by Kaszab (1940a: 175); taxon also described as a new taxon with the same species by Bates (1890: 69).

*Birolagria* Pic, 1956: 86 [F]. Type species: *Birolagriacicatricosa* Pic, 1956 (= *Cerogriaoriunda* Borchmann, 1924), by monotypy. Status: junior synonym of *Acerogria* Borchmann, 1936 in Lagriinae: Lagriini: Lagriina. Synonymy: Merkl (1987: 124).

*Biroum* Kaszab, 1956a: 104 [N]. Type species: *Biroumparadoxum* Kaszab, 1956, by original designation. Status: valid genus in Stenochiinae: Cnodalonini.

*Bius* Dejean, 1834: 205 [M]. Type species: *Trogossitathoracicus* Fabricius, 1792, by monotypy. Status: valid genus in Tenebrioninae: Tenebrionini.

*Blacodatus* Koch, 1963: 42 [M]. Type species: *Blacodesvertagus* Mulsant & Rey, 1859, by monotypy. Status: valid genus in Blaptinae: Opatrini: Stizopodina.

*Blacodes* Duponchel, 1842a: 590 [M]. Type species: *Pedinussulcatus* Laporte, 1840, by monotypy. Status: junior synonym of *Blenosia* Laporte, 1840 in Blaptinae: Opatrini: Stizopodina. Synonymy: Erichson (1843: 245). Note: name previously attributed to Blanchard (1845: 13) in the literature.

*Blapida* Perty, 1830: 58 [F]. Type species: *Blapidaokeni* Perty, 1830, by monotypy. Status: valid genus in Stenochiinae: Cnodalonini.

*Blapidium* Bauer, 1921: 231 [N]. Type species: *Blaps ocreata* Allard, 1880, by subsequent designation (Nabozhenko and Chigray 2020: 10). Status: junior synonym of *Blaps* Fabricius, 1775 in Blaptinae: Blaptini: Blaptina. Synonymy: Nabozhenko and Chigray (2020: 10). Note: originally proposed without included nominal species; Kolbe (1928: 200), by including four species in association with this name, was the first author to subsequently and expressly included nominal species in *Blapidium* (ICZN 1999, Article 67.2.2).

*Blapidocamaria* Pic, 1919a: 7 [F]. Type species: *Pseudoblapidaincostata* Pic, 1919, by monotypy. Status: valid subgenus of *Pseudoblapida* Pic, 1917 in Stenochiinae: Cnodalonini.

*Blapidocampsia* Pic, 1919b: 3 [F]. Type species: *Campsiapallidipes* Pic, 1918, by **present designation**. Status: valid subgenus of *Campsia* Lepeletier & Audinet-Serville, 1828 in Stenochiinae: Cnodalonini.

*Blapidurus* Fairmaire, 1891d: xcvi [M]. Type species: *Blapiduruscrassicornis* Fairmaire, 1891, by monotypy. Status: junior synonym of *Blaps* Fabricius, 1775 in Blaptinae: Blaptini: Blaptina. Synonymy: Champion (1895: 48).

*Blapimorpha* Motschulsky, 1860c: 531 [F]. Type species: *Blapsreflexa* Gebler, 1832, by subsequent designation (Nabozhenko 2008: 35). Status: junior synonym of *Blaps* Fabricius, 1775 in Blaptinae: Blaptini: Blaptina. Synonymy: Gemminger in Gemminger and Harold (1870: 1860).

*Blapisa* Motschulsky, 1860c: 530 [F]. Type species: *Blaps jaegeri* Hummel, 1827 (= *Tenebriomortisagus* Linnaeus, 1758), by subsequent designation (Nabozhenko 2008: 35). Status: junior synonym of *Blaps* Fabricius, 1775 in Blaptinae: Blaptini: Blaptina. Synonymy: Gemminger in Gemminger and Harold (1870: 1860).

*Blaposodes* Skopin, 1960a: 47 [M]. Type species: *Blaps baerii* Fischer von Waldheim, 1842, by original designation. Status: junior synonym of *Peltarium* Fischer von Waldheim, 1844 in Blaptinae: Blaptini: Blaptina. Synonymy: Nabozhenko and Chigray (2020: 10).

*Blaps* Fabricius, 1775: 254 [F]. Type species: *Tenebriomortisagus* Linnaeus, 1758, by subsequent designation (Latreille 1810: 429). Status: valid genus and subgenus in Blaptinae: Blaptini: Blaptina.

*Blapstinus* Dejean, 1821: 66 [M]. Type species: *Blapspunctata* Fabricius, 1792, by monotypy. Status: valid genus in Blaptinae: Opatrini: Blapstinina.

*Blaptogonia* G.S. Medvedev, 1998a: 186 [F]. Type species: *Tagonoidescostulata* Fairmaire, 1901, by original designation. Status: valid genus in Blaptinae: Blaptini: Gnaptorinina.

*Blaptoprosodes* Reitter, 1909a: 120 [M]. Type species: *Prosodesmucronata* Reitter, 1893, by original designation. Status: junior synonym of *Prosodes* Eschscholtz, 1829 in Blaptinae: Blaptini: Prosodina. Synonymy: G.S. Medvedev (2001: 54).

*Blapylis* Horn, 1870: 304, 315 [F]. Type species: *Eleodescordata* Eschscholtz, 1829, by subsequent designation (Bousquet et al. 2018: 143). Status: valid subgenus of *Eleodes* Eschscholtz, 1829 in Blaptinae: Amphidorini.

*Blastarnodes* Koch, 1958: 154 [M]. Type species: *Blastarnodesherero* Koch, 1958, by original designation. Status: valid genus in Blaptinae: Pedinini: Helopinina.

*Blastarnus* Fairmaire, 1897f: 132 [M]. Type species: *Blastarnusgrallator* Fairmaire, 1897, by subsequent designation (Oustalet 1898: 972). Status: junior synonym of *Diestecopus* Solier, 1848 in Blaptinae: Pedinini: Helopinina. Synonymy: Koch (1958: 152).

*Blatticephalus* Heller, 1918: 377 [M]. Type species: *Blatticephalusadelotopus* Heller, 1918, by monotypy. Status: valid genus in Tenebrioninae: Falsocossyphini.

*Blenosia* Laporte, 1840: 209 [F]. Type species: *Pedinussulcatus* Laporte, 1840, by subsequent designation (R. Lucas 1920: 140). Status: valid genus in Blaptinae: Opatrini: Stizopodina.

*Blepegenes* Pascoe, 1868: xii [M]. Type species: *Blepegenesaruspex* Pascoe, 1868, by monotypy. Status: valid genus in Lagriinae: Adeliini.

*Blepusa* Westwood, 1842: 69 [F]. Type species: *Blepusacostata* Westwood, 1842, by monotypy. Status: valid genus in Alleculinae: Alleculini: Alleculina.

*Blindus* Mulsant & Rey, 1853b: 206 [M]. Type species: *Pedinusstrigosus* Faldermann, 1835, by monotypy. Status: valid subgenus of *Pedinus* Latreille, 1797 in Blaptinae: Pedinini: Pedinina.

*Bluops* Carter, 1914a: 227 [M]. Type species: *Bluopsverrucosus* Carter, 1914, by monotypy. Status: valid genus in Lagriinae: Adeliini.

*Bobina* Novák, 2015a: 125 [F]. Type species: *Bobinajendeki* Novák, 2015, by original designation. Status: valid genus in Alleculinae: Alleculini: Alleculina.

*Bobisthes* Novák, 2019a: 178 [M]. Type species: *Bobisthesbellator* Novák, 2019, by original designation. Status: valid genus in Alleculinae: Alleculini: Alleculina.

*Bogatshevia* G.S. Medvedev & Iwan, 2006: 618 [F]. Type species [automatic]: *Achaemenesvillosus* Bogatchev, 1949 (= *Thripterabogatchevi* Kwieton, 1982), by original designation. Status: valid genus in Pimeliinae: Pimeliini. Note: replacement name for *Achaemenes* Bogatchev, 1949 (as “*Achaemenus*”).

*Bolbophanes* Carter, 1913a: 86 [M]. Type species: *Paraphanesdumbrelli* Lea, 1895, by subsequent designation (Gebien 1941: 1133). Status: valid genus in Tenebrioninae: Heleini: Cyphaleina.

*Bolbostetha* Fairmaire, 1896c: 117 [F]. Type species: *Bolbostethasoleata* Fairmaire, 1896, by subsequent designation (Novák and Pettersson 2008: 320). Status: valid genus in Alleculinae: Alleculini: Alleculina.

*Boletophagus* Agassiz, 1846b: 48 [M]. Type species [automatic]: *Silphareticulata* Linnaeus, 1767, by subsequent designation (C.G. Thomson 1859: 115). Status: junior synonym of *Bolitophagus* Illiger, 1798 in Tenebrioninae: Bolitophagini. Note: unjustified emendation of *Bolitophagus* Illiger, 1798, not in prevailing usage.

*Boletoxenus* Motschulsky, 1858a: 63 [M]. Type species: *Boletoxenusgibber* Motschulsky, 1858, by monotypy. Status: valid genus in Tenebrioninae: Bolitophagini.

*Bolithophilus* Gistel, 1832: 137 [M]. Type species [automatic]: *Cistelascapularis* Illiger, 1805 (= *Cistelahumeralis* Fabricius, 1787), by subsequent designation (Westwood 1838: 32). Status: junior synonym of *Mycetochara* Guérin-Méneville, 1827 in Alleculinae: Alleculini: Mycetocharina. Note: replacement name for *Mycetophila* Gyllenhal, 1810.

*Bolitolaemus* Gebien, 1921b: 23 [M]. Type species: *Bolitolaemuscatenulatus* Gebien, 1921, by monotypy. Status: valid genus in Tenebrioninae: Bolitophagini.

*Bolitonaeus* Lewis, 1894: 387 [M]. Type species: *Bolitonaeusmergae* Lewis, 1894, by original designation. Status: valid genus in Tenebrioninae: Bolitophagini.

*Bolitopertha* Gebien, 1910c: 379 [F]. Type species: *Bolitoperthanovemcostata* Gebien, 1910 (= *Bolitophagusborbonicus* Fairmaire, 1880), by monotypy. Status: junior synonym of *Rhipidandrus* J.L. LeConte, 1862 in Tenebrioninae: Bolitophagini. Synonymy: Gebien (1922b: 268, with *Cherostus* C.O. Waterhouse, 1894, a junior synonym of *Rhipidandrus* J.L. LeConte, 1862).

*Bolitophagiella* Miyatake, 1964: 68, 71 [F]. Type species: *Bolitophaguspannosus* Lewis, 1894, by original designation. Status: valid genus in Tenebrioninae: Bolitophagini.

*Bolitophagus* Illiger, 1798: 100 [M]. Type species: *Silphareticulata* Linnaeus, 1767, by subsequent designation (C.G. Thomson 1859: 115). Status: valid genus in Tenebrioninae: Bolitophagini. Note: as mentioned by Bouchard and Bousquet (2020b: 8) nomenclatural stability in this genus is threatened by the discovery of an older type species designation (*Opatrumagricola* Herbst, 1783, by subsequent designation by Curtis (1836: pl. 586), which is currently the type species of the valid genus *Eledona* Latreille, 1797); we recommend that an application be submitted to the International Commission on Zoological Nomenclature to maintain the type species designation proposed by C.G. Thomson (1859: 115).

*Bolitotherus* Candèze, 1861: 367 [M]. Type species: *Bolitophaguscornutus* Fabricius, 1801, by subsequent designation (J.L. LeConte 1862: 236). Status: valid genus in Tenebrioninae: Bolitophagini. Note: the younger species name *Bolitophaguscornutus* Fabricius, 1801 was given priority over the older synonym *Opatrumbifurcum* Fabricius, 1798 by the ICZN (2019, Opinion 2438).

*Bolitotrogus* Miyatake, 1964: 68, 80 [M]. Type species: *Bolitotroguskurosonis* Miyatake, 1964, by original designation. Status: valid genus in Tenebrioninae: Bolitophagini.

*Bolitoxenus* Gemminger in Gemminger and Harold, 1870: 1946 [M]. Type species [automatic]: *Boletoxenusgibber* Motschulsky, 1858, by monotypy. Status: junior synonym of *Boletoxenus* Motschulsky, 1858 in Tenebrioninae: Bolitophagini. Note: unjustified emendation of *Boletoxenus* Motschulsky, 1858, not in prevailing usage.

*Bolitrium* Gebien, 1914b: 390 [N]. Type species: *Bolitriumcrenulicolle* Gebien, 1914, by monotypy. Status: valid genus in Lagriinae: Lupropini.

*Bolusculus* Matthews, 1998: 704, 798 [M]. Type species: *Bolusculusarcanus* Matthews, 1998, by original designation. Status: valid genus in Lagriinae: Adeliini.

*Bombocnodulus* Koch, 1955a: 36 [M]. Type species: *Psammodescrinicollis* Haag- Rutenberg, 1879, by monotypy. Status: valid genus in Pimeliinae: Sepidiini: Hypomelina.

*Borbochara* Novák, 2009: 259 [F]. Type species: *Borbocharabicolor* Novák, 2009, by original designation. Status: valid genus in Alleculinae: Alleculini: Alleculina.

*Borbonalia* Novák, 2014: 136 [F]. Type species: *Borbonaliabrancuccii* Novák, 2014, by original designation. Status: valid genus in Alleculinae: Alleculini: Alleculina.

*Borborella* Novák, 2020a: 196 [F]. Type species: *Borborellahergovitsi* Novák, 2020, by original designation. Status: valid genus in Alleculinae: Alleculini: Alleculina.

*Borboresthes* Fairmaire, 1897d: 253 [F]. Type species: *Alleculacruralis* Marseul, 1876, by original designation. Status: valid genus in Alleculinae: Alleculini: Alleculina. Note: see Löbl and Smetana (2013: 39) for comments on the gender of this name.

*Borchmannia* Pic in Borchmann and Pic, 1912: 35 [F]. Type species: *Borchmannialineaticeps* Pic, 1912, by subsequent designation (R. Lucas 1920: 144). Status: valid genus and subgenus in Lagriinae: Lagriini: Statirina.

*Borchmannius* Bousquet & Bouchard in Bousquet et al., 2015: 142 [M]. Type species [automatic]: *Glyptothoraxpilosus* Borchmann, 1937, by original designation. Status: valid genus in Alleculinae: incertae sedis. Note: replacement name for *Glyptothorax* Borchmann, 1937.

*Boreoptorina* G.S. Medvedev, 2009: 417 [F]. Type species: *Gnaptorinacordicollis* G.S. Medvedev, 1998, by original designation. Status: junior synonym of *Hesperoptorina* G.S. Medvedev, 2009 in Blaptinae: Blaptini: Gnaptorinina. Synonymy and First Reviser action (*Boreoptorina* G.S. Medvedev, 2009 versus *Hesperoptorina* G.S. Medvedev, 2009) by Li et al. (2021: 245).

*Boreosaragus* Matthews, 1993: 1040, 1067 [M]. Type species: *Saraguslugubris* Lea, 1897, by original designation. Status: valid genus in Tenebrioninae: Heleini: Heleina.

*Borneocamaria* Pic, 1917e: 17 [F]. Type species: *Borneocamariaatra* Pic, 1917, by monotypy. Status: valid genus in Stenochiinae: Cnodalonini.

*Borneocistela* Pic, 1922d: 18 [F]. Type species: *Borneocisteladiversicornis* Pic, 1922, by monotypy. Status: valid genus in Alleculinae: incertae sedis.

*Borneogonocnemis* Pic, 1936b: 16 [F]. Type species: *Borneogonocnemisruficolor* Pic, 1936, by monotypy. Status: valid subgenus of *Paragonocnemis* Kraatz, 1899 in Tenebrioninae: Amarygmini.

*Borneolaena* Schawaller, 1998: 2 [F]. Type species: *Borneolaenariedeli* Schawaller, 1998, by original designation. Status: valid genus in Lagriinae: Laenini.

*Borneosphaerotus* Grimm, 2015: 218 [M]. Type species: *Borneosphaerotussantubongicus* Grimm, 2015, by original designation. Status: valid genus in Stenochiinae: Cnodalonini.

*Borneosphena* Purchart & Grimm, 2016: 522 [F]. Type species: *Borneosphenafouquei* Purchart & Grimm, 2016, by original designation. Status: valid genus in Stenochiinae: Cnodalonini.

*Borneostira* Pic, 1912: 53 [F]. Type species: *Rouyerusbrevilineatus* Pic, 1912, by monotypy. Status: valid subgenus of *Rouyerus* Pic, 1911 in Lagriinae: Lagriini: Statirina.

*Borneosynopticus* Grimm, 2015: 219 [M]. Type species: *Borneosynopticustubericollis* Grimm, 2015, by original designation. Status: valid genus in Stenochiinae: Cnodalonini.

*Boromorphus* Wollaston, 1854: 492 [M]. Type species: *Boromorphusmaderae* Wollaston, 1854 (= *Borostagenioides* P.H. Lucas, 1846), by monotypy. Status: valid genus in Pimeliinae: Boromorphini.

*Bothrasida* Casey, 1912: 76, 122 [F]. Type species: *Asidaclathrata* Champion, 1884, by original designation. Status: valid subgenus of *Stenomorpha* Solier, 1836 in Pimeliinae: Asidini.

*Bothrichara* Borchmann, 1916a: 48, 104 [F]. Type species: *Lagriapulchella* Guérin-Méneville, 1830, by subsequent designation (Borchmann 1936: 68). Status: valid genus in Lagriinae: Lagriini: Lagriina.

*Bothrionota* Borchmann, 1936: 16, 66 [F]. Type species: *Bothrionotafoveata* Borchmann, 1930, by original designation. Status: valid genus in Lagriinae: Lagriini: Lagriina.

*Bothriostira* Borchmann, 1936: 239, 473 [F]. Type species: *Bothriostiracylindracea* Borchmann, 1936, by original designation. Status: valid genus in Lagriinae: Lagriini: Statirina.

*Bothrotes* Casey, 1907: 379 [M]. Type species: *Epitraguscanaliculatus* Say, 1824, by original designation. Status: valid genus in Pimeliinae: Epitragini.

*Bothynocara* Gebien, 1928: 167, 170 [N]. Type species: *Bothynocaratenuepunctatum* Gebien, 1928, by original designation. Status: valid genus in Stenochiinae: Cnodalonini.

*Bothynocephalus* Doyen, 1988: 315 [M]. Type species: *Bothynocephaluscristatus* Doyen, 1988, by original designation. Status: valid genus in Stenochiinae: Cnodalonini.

*Bothynogria* Borchmann, 1916a: 128 [F]. Type species: *Bothynogriacalcarata* Borchmann, 1916, by monotypy. Status: valid genus in Lagriinae: Lagriini: Lagriina.

*Botiras* Fairmaire, 1891d: xcviii [M]. Type species: *Botirasstriatellus* Fairmaire, 1891, by subsequent designation (R. Lucas 1920: 145). Status: junior synonym of *Chianalus* Bates, 1879 in Blaptinae: Platyscelidini. Synonymy: Blair (1923a: 283).

*Bouchardandrus* Steiner, 2016: 543 [M]. Type species: *Haplandrusconcolor* J.L. LeConte, 1866, by original designation. Status: valid genus in Tenebrioninae: Tenebrionini.

*Bovius* Gistel, 1848a: ix [M]. Type species [automatic]: *Tenebrioserratus* Fabricius, 1775, by monotypy. Status: junior synonym of *Prioscelis* Hope, 1841 in Lagriinae: Pycnocerini. Note: unnecessary replacement name for *Iphius* Dejean, 1834.

*Brachycilibe* Carter, 1911a: 207 [F]. Type species: *Brachycilibeantennata* Carter, 1911, by monotypy. Status: junior synonym of *Dioedus* J.L. LeConte, 1862 in Phrenapatinae: Penetini. Synonymy: Kaszab (1978a: 164).

*Brachycryptus* Quedenfeldt, 1891: 129 [M]. Type species: *Brachycryptustripolitanus* Quedenfeldt, 1891, by monotypy. Status: senior synonym of *Tripolicryptus* Strand, 1929 in Alleculinae: Cteniopodini. Note: junior homonym of *Brachycryptus* C.G. Thomson, 1873 [Hymenoptera].

*Brachycula* Fairmaire, 1906: 278 [F]. Type species: *Brachyculaquadrivittata* Fairmaire, 1906, by monotypy. Status: valid genus in Alleculinae: Alleculini: Gonoderina. Note: the alternative original spelling *Brachicula*, used by Fairmaire (1906: 278), was rejected by Bousquet et al. (2015: 143) who acted as the First Revisers.

*Brachycyphus* Gebler, 1859: 473. Type species: *Pimeliaimbricata* Fischer, 1820, by monotypy. Status: junior synonym of *Ocnera* Fischer, 1822 in Pimeliinae: Pimeliini. Synonymy: Gemminger in Gemminger and Harold (1870: 1888).

*Brachyesthes* Fairmaire, 1868: 490 [F]. Type species: *Melanesthespilosellus* Marseul, 1866, by monotypy. Status: valid genus in Blaptinae: Opatrini: Opatrina.

*Brachygenius* Dejean, 1836: 206 [M]. Type species [automatic]: *Nyctelialuczotii* Guérin-Méneville, 1831, by subsequent designation (Duponchel 1845b: 449). Status: junior synonym of *Gyriosomus* Guérin-Méneville, 1834 in Pimeliinae: Nycteliini. Note: unnecessary replacement name for *Gyriosomus* Guérin-Méneville, 1834.

*Brachyidium* Fairmaire, 1883a: 33 [N]. Type species: *Brachyidiumbreviusculum* Fairmaire, 1883, by monotypy. Status: valid genus in Blaptinae: Opatrini: Ammobiina.

*Brachymoschium* Fairmaire, 1896b: 348 [N]. Type species: *Brachymoschiumparvitarse* Fairmaire, 1896, by monotypy. Status: valid genus in Pimeliinae: Adelostomini.

*Brachyontis* Casey, 1908: 82, 141 [F]. Type species: *Coniontisglobulina* Casey, 1895, by monotypy. Status: junior synonym of *Coniontis* Eschscholtz, 1829 in Pimeliinae: Coniontini. Synonymy: Aalbu et al. (2002: 487).

*Brachyphrynus* Fairmaire in Fairmaire et al., 1882: 71 [M]. Type species: *Brachyphrynusspissicornis* Fairmaire, 1882, by monotypy. Status: valid genus in Pimeliinae: Sepidiini: Molurina.

*Brachypilium* Fairmaire, 1896a: 23 [N]. Type species: *Brachypiliumsculpturatum* Fairmaire, 1896, by monotypy. Status: junior synonym of *Stenochinus* Motschulsky, 1860 in Stenochiinae: Cnodalonini. Synonymy: Löbl et al. (2008b: 349).

*Brachypophlaeus* Fairmaire, 1897a: 113 [M]. Type species: *Hypophlaeusdimidiatipennis* Fairmaire, 1880, by monotypy. Status: valid genus in Tenebrioninae: Ulomini.

*Brachyscelis* Dejean, 1834: 179 [F]. Type species: *Pimeliamusiva* Ménétriés, 1832, by subsequent designation (Bouchard et al. 2007: 388). Status: senior synonym of *Pachyscelis* Solier, 1836 in Pimeliinae: Pimeliini. Synonymy: Bouchard et al. (2007: 388). Note: the date of publication of the name *Brachyscelis* Germar, 1834 (Coleoptera: Chrysomelidae) is unknown whereas *Brachyscelis* Dejean was published by 30 June 1834; nomen oblitum (see Bouchard et al. 2007: 388).

*Bradygena* Fairmaire, 1903c: 211 [F]. Type species: *Nesogenaviolacea* Fairmaire, 1903 (= *Nesogenaepiscopalis* Fairmaire, 1875), by subsequent designation (Froussart 1961: 91). Status: junior synonym of *Nesogena* Mäklin, 1863 in Tenebrioninae: Praeugenini. Synonymy: Gebien (1948: 549).

*Bradymerus* Perroud & Montrouzier, 1865: 110 [M]. Type species: *Bradymerustuberculatus* Perroud & Montrouzier, 1865, by monotypy. Status: valid genus in Stenochiinae: Cnodalonini.

*Bradynocerus* Fairmaire, 1883a: 36 [M]. Type species: *Bradynocerusaulacopterus* Fairmaire, 1883, by monotypy. Status: junior synonym of *Bradymerus* Perroud & Montrouzier, 1865 in Stenochiinae: Cnodalonini. Synonymy: Gebien (1939: 751).

*Bradysphaerotus* Kaszab, 1986: 293 [M]. Type species: *Bradysphaerotuspapuanus* Kaszab, 1986, by original designation. Status: valid genus in Stenochiinae: Cnodalonini.

*Bradyus* Dejean, 1834: 190 [M]. Type species: *Erodiuspygmaeus* Fischer, 1821, by monotypy. Status: valid genus in Tenebrioninae: Dissonomini.

*Branchus* J.L. LeConte, 1862: 222 [M]. Type species: *Branchusfloridanus* J.L. LeConte, 1862, by monotypy. Status: valid genus in Pimeliinae: Branchini.

*Brasilius* Gebien, 1928: 169, 178 [M]. Type species: *Nyctobatesexaratus* Fairmaire, 1889, by original designation. Status: valid genus in Stenochiinae: Cnodalonini.

*Bratyna* Westwood, 1875: 228 [F]. Type species: *Bratynaapicalis* Westwood, 1875, by monotypy. Status: valid genus in Alleculinae: incertae sedis.

*Bremerianus* Masumoto & Bečvář, 2005: 418 [M]. Type species: *Bremerianuscameronensis* Masumoto & Bečvář, 2005, by original designation. Status: valid genus in Stenochiinae: Stenochiini.

*Bremerus* Ferrer, 2004a: 234 [M]. Type species: *Bremerusfrankkochi* Ferrer, 2004, by original designation. Status: valid genus in Tenebrioninae: Tenebrionini.

*Brinckia* Koch, 1962b: 117 [F]. Type species: *Psammodesdebilis* Péringuey, 1899, by original designation. Status: valid genus in Pimeliinae: Sepidiini: Hypomelina.

*Brises* Pascoe, 1869: 145 [M]. Type species: *Brisestrachynotoides* Pascoe, 1869, by monotypy. Status: valid genus in Tenebrioninae: Heleini: Heleina. Note: the First Reviser (*Brises* Pascoe, 1869 versus *Ephidonius* Pascoe, 1869) is Carter (1914b: 46).

*Brittona* G.S. Medvedev & Lawrence, 1986: 574 [F]. Type species: *Brittonaminuta* G.S. Medvedev & Lawrence, 1986, by original designation. Status: valid genus in Diaperinae: Hyociini: Brittonina.

*Broomium* Koch, 1950b: 338, 342 [N]. Type species: *Broomiumnudum* Koch, 1950, by original designation. Status: valid genus in Pimeliinae: Tentyriini.

*Brosimapsida* Ferrer & Ødegaard, 2005: 640 [F]. Type species: *Brosimapsidagonospoides* Ferrer & Ødegaard, 2005, by original designation. Status: valid genus in Stenochiinae: Cnodalonini.

*Brycopia* Pascoe, 1869: 141 [F]. Type species: *Brycopiapilosella* Pascoe, 1869, by monotypy. Status: valid genus in Lagriinae: Adeliini. Note: the First Reviser (*Brycopia* Pascoe, 1869 versus *Dinoria* Pascoe, 1869) is Carter (1920a: 238).

*Bulbulus* Lesne, 1915: 227, 240 [M]. Type species: *Arthrodeisbyrrhiformis* Fairmaire, 1892, by monotypy. Status: valid genus in Pimeliinae: Erodiini.

*Bunamarygmus* Masumoto, 1988a: 127 [M]. Type species: *Bunamarygmusthailandicus* Masumoto, 1988, by original designation. Status: valid genus in Tenebrioninae: Amarygmini.

*Burmanosis* G.S. Medvedev, 1995a: 845, 859 [F]. Type species: *Stenosiskaszabi* G.S. Medvedev, 1995, by original designation. Status: valid subgenus of *Stenosis* Herbst, 1799 in Pimeliinae: Stenosini: Stenosina.

*Buxela* Fairmaire, 1894a: 28 [F]. Type species: *Buxelasordescens* Fairmaire, 1894, by monotypy. Status: valid genus in Alleculinae: incertae sedis.

*Byallius* Pascoe, 1869: 42 [M]. Type species: *Byalliusreticulatus* Pascoe, 1869, by monotypy. Status: valid genus in Tenebrioninae: Heleini: Cyphaleina.

*Bycrea* Pascoe, 1868: xii [F]. Type species: *Bycreavillosa* Pascoe, 1868, by monotypy. Status: junior synonym of *Trichoton* Hope, 1841 in Blaptinae: Opatrini: Blapstinina. Synonymy: Kamiński et al. (2019c: 360).

*Byrrhoncus* Koch, 1954a: 49 [M]. Type species: *Byrrhoncusmonticola* Koch, 1954, by original designation. Status: valid genus in Blaptinae: Platynotini: Eurynotina.

*Byrsax* Pascoe, 1860a: 42 [M]. Type species: *Byrsaxcoenosus* Pascoe, 1860 (= *Boletophagusgibbifer* Wesmael, 1836), by monotypy. Status: valid genus in Tenebrioninae: Bolitophagini. Note: genus originally described in Tenebrionoidea: Zopheridae: Colydiinae.

*Byzacnus* Pascoe, 1866a: 469 [M]. Type species: *Byzacnuspicticollis* Pascoe, 1866, by monotypy. Status: valid genus in Stenochiinae: Cnodalonini.

*Cabirus* Mulsant & Rey, 1853b: 148, 223 [M]. Type species: *Cabirusminutissimus* Mulsant & Rey, 1853, by subsequent designation (Gebien 1938a: 314). Status: senior synonym of *Cabirutus* Strand, 1929 in Blaptinae: Pedinini: Pedinina. Note: junior homonym of *Cabirus* Hübner, 1819 [Lepidoptera].

*Cabirutus* Strand, 1929: 24 [M]. Type species [automatic]: *Cabirusminutissimus* Mulsant & Rey, 1853, by subsequent designation (Gebien 1938a: 314). Status: valid genus and subgenus in Blaptinae: Pedinini: Pedinina. Note: replacement name for *Cabirus* Mulsant & Rey, 1853.

*Cacicus* Dejean, 1834: 182 [M]. Type species: *Elenophorusamericanus* Lacordaire, 1830, by monotypy. Status: senior synonym of *Megelenophorus* Gebien, 1910 in Pimeliinae: Elenophorini: Megelenophorina. Note: junior homonym of *Cacicus* Lacépède, 1799 [Aves].

*Cacoplesia* Fairmaire, 1898a: 237 [F]. Type species [automatic]: *Plesiamelanura* Klug, 1833, by subsequent designation (Bousquet et al. 2015: 137). Status: junior synonym of *Eubalia* Laporte, 1840 in Alleculinae: Alleculini: Gonoderina. Synonymy: Borchmann (1909a: 713). Note: replacement name for *Plesia* Klug, 1833.

*Caecochares* Koch, 1956b: 91 [M]. Type species: *Caecocharesgrjebinei* Koch, 1956, by original designation. Status: valid genus in Diaperinae: Gnathidiini: Gnathidiina.

*Caecomenimopsis* Kaszab, 1970b: 198 [F]. Type species: *Caecomenimopsisleleupi* Kaszab, 1970, by original designation. Status: junior synonym of *Menimopsis* Champion, 1896 in Diaperinae: Gnathidiini: Anopidiina. Synonymy: Peck (1990: 370).

*Caecophloeus* Dajoz, 1972: 278 [M]. Type species: *Caecophloeusfranzi* Dajoz, 1972, by original designation. Status: valid genus in Diaperinae: Gnathidiini: Anopidiina.

*Caediexis* Lebedev, 1932: 125 [F]. Type species: *Caediexisarenicola* Lebedev, 1932, by monotypy. Status: valid genus in Blaptinae: Opatrini: Ammobiina.

*Caediomorpha* Blackburn, 1888: 272 [F]. Type species: *Caediomorphaaustralis* Blackburn, 1888 (= *Morychusheteromerus* King, 1869), by monotypy. Status: valid genus in Blaptinae: Opatrini: Opatrina. Note: moved from the subtribe Ammobiina to Opatrina by Lumen et al. (2020: 346).

*Caedius* Blanchard, 1845: 13 [M]. Type species: *Opatrumsphaeroides* Hope, 1843, by subsequent designation (Lacordaire 1859a: 262). Status: valid genus in Blaptinae: Opatrini: Ammobiina. Note: *Caedius* is an incorrect subsequent spelling of the original spelling *Coedius*, first used by Lacordaire (1859a: 261), and in prevailing usage; *Caedius* is deemed to be the correct original spelling (ICZN 1999, Article 33.3.1).

*Caenoblaps* König, 1906: 24 [F]. Type species: *Caenoblapsdifformis* König, 1906, by monotypy. Status: junior synonym of *Dila* Fischer von Waldheim, 1844 in Blaptinae: Blaptini: Blaptina. Synonymy: Chigray et al. (2019: 3).

*Caenocapicus* Endrödy-Younga, 1996: 15, 19 [M]. Type species: *Caenocrypticuscapensis* Endrödy-Younga, 1996, by original designation. Status: valid subgenus of *Caenocrypticus* Gebien, 1920 in Pimeliinae: Caenocrypticini.

*Caenocorse* C.G. Thomson, 1859: 117 [F]. Type species: *Hypophlaeusdepressus* Fabricius, 1790, by original designation. Status: junior synonym of *Palorus* Mulsant, 1854 in Tenebrioninae: Palorini. Synonymy: Bedel (1906a: 92).

*Caenocrypticoides* Kaszab, 1969b: 322 [M]. Type species: *Caenocrypticoidesloksai* Kaszab, 1969, by original designation. Status: valid genus in Pimeliinae: Caenocrypticini. Note: we act as First Revisers and reject the alternative original spelling *Caenocripticoides*, used by Kaszab (1969b: 324).

*Caenocrypticus* Gebien, 1920: 139 [M]. Type species: *Caenocrypticusuncinatus* Gebien, 1920, by monotypy. Status: valid genus and subgenus in Pimeliinae: Caenocrypticini.

*Calabosca* Fairmaire, 1894f: 395 [F]. Type species [automatic]: *Ascalabuspedinoides* Fairmaire, 1893, by monotypy. Status: valid genus in Stenochiinae: Cnodalonini. Note: replacement name for *Ascalabus* Fairmaire, 1893.

*Calaharena* Koch, 1963: 64 [F]. Type species: *Calaharenadutoiti* Koch, 1963, by original designation. Status: valid genus in Blaptinae: Opatrini: Stizopodina.

*Calcar* Dejean, 1821: 67 [N]. Type species: *Tenebrioelongatus* Herbst, 1797, by monotypy. Status: senior synonym of *Belopus* Gebien, 1911 in Lagriinae: Belopini. Note: junior homonym of *Calcar* de Montfort, 1810 [Mollusca].

†*Calcarocistela* Nabozhenko in Nabozhenko et al., 2016a: 1421 [F]. Type species: *Calcarocistelakirejtshuki* Nabozhenko, 2016, by original designation. Status: valid genus in Alleculinae: Alleculini: Gonoderina. Note: described from Lower Cretaceous deposits (China).

*Calcarosis* Penrith, 1977: 21, 98 [F]. Type species: *Zophosismichaelis* Penrith, 1977, by original designation. Status: junior synonym of *Zophosis* Latreille, 1802 in Pimeliinae: Zophosini. Synonymy: Penrith (1981a: 22).

*Callicomus* Motschulsky, 1860d: 138 [M]. Type species: *Diaperisriederii* Faldermann, 1833, by subsequent designation (Jakobson 1924: 243). Status: senior synonym of *Emypsara* Pascoe, 1866 in Diaperinae: Phaleriini. Synonymy: Jakobson (1924: 243). Note: junior homonym of *Callicomus* Agassiz, 1846 [Coleoptera: Cerambycidae].

*Callignathus* Agassiz, 1846b: 58, 60 [M]. Type species [automatic]: *Calognathuschevrolatii* Guérin-Méneville, 1836, by monotypy. Status: junior synonym of *Calognathus* Guérin-Méneville, 1836 in Pimeliinae: Cryptochilini: Calognathina. Note: unjustified emendation of *Calognathus* Guérin-Méneville, 1836, not in prevailing usage.

*Callimaria* Fairmaire, 1888b: 12 [F]. Type species: *Callimariaimpressipennis* Fairmaire, 1888, by monotypy. Status: junior synonym of *Actanorie* Bates, 1879 in Stenochiinae: Cnodalonini. Synonymy: Ardoin (1956b: 90).

*Callismilax* Bates, 1874: 105 [F]. Type species: *Leptomorphaaenea* Montrouzier, 1860, by subsequent designation (Gebien 1942a: 333). Status: valid genus in Tenebrioninae: Titaenini. Note: Matthews and Lawrence (2019: 637) mentioned that this genus is not distinguishable from *Titaena* Erichson, 1842.

*Callyntra* Solier, 1836: 307, 335 [F]. Type species: *Nycteliamulticosta* Guérin-Méneville, 1834, by subsequent designation (Duponchel 1842b: 63). Status: valid genus in Pimeliinae: Nycteliini.

*Calobamon* Kraatz, 1865: 80, 105 [M]. Type species: *Calobamonleptoderus* Kraatz, 1865, by monotypy. Status: senior synonym of *Thraustocolus* Kraatz, 1866 in Pimeliinae: Tentyriini. Note: junior homonym of *Calobamon* Loew, 1850 [Diptera].

*Calognathus* Guérin-Méneville, 1836: pl. 172 [M]. Type species: *Calognathuschevrolatii* Guérin-Méneville, 1836, by monotypy. Status: valid genus in Pimeliinae: Cryptochilini: Calognathina.

*Calogria* Borchmann, 1916a: 48, 107 [F]. Type species: *Calogriacostata* Borchmann, 1916, by monotypy. Status: valid genus in Lagriinae: Lagriini: Lagriina.

*Calosis* Deyrolle, 1867: 81, 222 [F]. Type species: *Calosisamabilis* Deyrolle, 1867, by monotypy. Status: valid subgenus of *Zophosis* Latreille, 1802 in Pimeliinae: Zophosini. Note: the alternative original spelling *Calliosis* (p. 81) was corrected to *Calosis* in the “Errata” of the same work (p. 248), *Calosis* is considered to be the correct original spelling (ICZN 1999, Article 32.5.1.1).

*Calostega* Gemminger in Gemminger and Harold, 1870: 1991 [F]. Type species [automatic]: *Calostegiapurpuripennis* Westwood, 1843, by monotypy. Status: junior synonym of *Calostegia* Westwood, 1843 in Lagriinae: Pycnocerini. Note: unjustified emendation of *Calostegia* Westwood, 1843, not in prevailing usage.

*Calostegia* Westwood, 1843: 117 [F]. Type species: *Calostegiapurpuripennis* Westwood, 1843, by monotypy. Status: valid genus in Lagriinae: Pycnocerini. Note: redescribed as a new genus by Westwood (1844: 221, as “*Calostega*”).

*Calous* Koch, 1958: 151 [M]. Type species: *Blastarnusmichaelseni* Gebien, 1920, by original designation. Status: valid subgenus of *Nicandra* Fairmaire, 1888 in Blaptinae: Pedinini: Helopinina.

*Calydonella* Doyen, 1995: 8 [F]. Type species: *Calydonellalisa* Doyen, 1995, by original designation. Status: valid genus in Stenochiinae: Cnodalonini.

*Calydoniomorpha* Pic, 1917g: 19 [F]. Type species: *Calydoniomorphabrevicornis* Pic, 1917, by monotypy. Status: valid genus in Stenochiinae: Cnodalonini.

*Calydonis* Pascoe, 1882: 31 [F]. Type species: *Calydonisrefulgens* Pascoe, 1882, by subsequent designation (Gebien 1919: 141). Status: valid genus in Stenochiinae: Cnodalonini.

*Calymmatophorus* Gemminger in Gemminger and Harold, 1870: 1904 [M]. Type species [automatic]: *Praociscucullatus* Guérin-Méneville, 1834, by subsequent designation (R. Lucas 1920: 161). Status: junior synonym of *Calymmophorus* Solier, 1841 in Pimeliinae: Praociini. Note: unjustified emendation of *Calymmophorus* Solier, 1841 (as “*Calymmaphorus*”), not in prevailing usage.

*Calymmophorus* Solier, 1841a: 209, 245 [M]. Type species: *Praociscucullata* Guérin-Méneville, 1834, by subsequent designation (R. Lucas 1920: 161). Status: valid genus in Pimeliinae: Praociini. Note: two spellings were originally used, *Calymmaphorus* (pp. 209, 245, 246, 247, 250, 370) and *Galymmaphorus* (p. 247, in note); however, the incorrect subsequent spelling *Calymmophorus*, first introduced by Burmeister (1875: 494), is in prevailing usage and deemed to be the correct original spelling (ICZN 1999, Article 33.3.1).

*Calymmus* Montrouzier, 1860: 289 [M]. Type species: *Toxicumberardi* Montrouzier, 1860, by monotypy. Status: valid genus in Tenebrioninae: Toxicini: Dysantina. Note: the name *Calymmus* was listed as a synonym of *Toxicum* Latreille, 1802 by Montrouzier (1860: 289); it was treated before 1961 as an available name and adopted as the name of a taxon (e.g., Fauvel 1862: 148); therefore, *Calymmus* was made available from its first publication as a synonym (ICZN 1999, Article 11.6.1).

*Calyptopsis* Solier, 1835b: 253, 269 [F]. Type species: *Calyptopsisemondi* Solier, 1835 (= *Hegetercaraboides* Brullé, 1832), by monotypy. Status: valid genus in Pimeliinae: Tentyriini.

*Camaria* Lepeletier & Audinet-Serville, 1828: 454 [F]. Type species: *Camarianitida* Lepeletier & Audinet-Serville, 1828 (= *Tenebrionitens* G.-A. Olivier, 1795), by monotypy. Status: valid genus in Stenochiinae: Cnodalonini.

*Camarimena* Motschulsky, 1863: 473 [F]. Type species: *Camarimenaovicauda* Motschulsky, 1863, by monotypy. Status: valid genus in Stenochiinae: Cnodalonini.

*Camariocropterum* Pic, 1920a: 16 [N]. Type species: *Camariocropterumlaticeps* Pic, 1920, by monotypy. Status: valid genus in Stenochiinae: Cnodalonini.

*Camariodes* Fairmaire, 1869b: 232 [M]. Type species: *Camariodescoquerelii* Fairmaire, 1869 (= *Camariahelopioides* Klug, 1833), by monotypy. Status: valid genus in Stenochiinae: Cnodalonini. Note: the First Reviser (*Camariodes* Fairmaire, 1869 versus *Tinophyllus* Fairmaire, 1869) is Fairmaire (1886c: 75).

*Camariomorpha* Pic, 1915d: 7 [F]. Type species: *Camariomorphasingularis* Pic, 1915, by monotypy. Status: valid genus in Stenochiinae: Cnodalonini.

*Camarothelops* Kolbe, 1910: 30 [M]. Type species: *Camarothelopsbraueri* Kolbe, 1910, by monotypy. Status: valid genus in Tenebrionidae: incertae sedis. Note: removed from the tribe Helopini and placed as Tenebrionidae incertae sedis by Nabozhenko (2018: 183).

*Camphonota* Solier, 1836: 195 [F]. Type species: *Tenebriosubglobosus* Pallas, 1781, by subsequent designation (Lacordaire 1859a: 188). Status: valid subgenus of *Pimelia* Fabricius, 1775 in Pimeliinae: Pimeliini.

*Campolene* Pascoe, 1863a: 44 [F]. Type species: *Campolenenitida* Pascoe, 1863, by monotypy. Status: valid genus in Stenochiinae: Cnodalonini.

*Camponotiphilus* Lea, 1914: 257 [M]. Type species: *Camponotiphilusfimbricollis* Lea, 1914, by monotypy. Status: valid genus in Tenebrioninae: Heleini: Heleina.

*Campsia* Lepeletier & Audinet-Serville, 1828: 455 [F]. Type species: *Cnodalonirroratum* Dalman, 1823, by subsequent designation (Hope 1841: 133). Status: valid genus and subgenus in Stenochiinae: Cnodalonini.

*Campsiomorpha* Pic, 1917g: 19 [F]. Type species: *Camarialata* Pic, 1915, by subsequent designation (Gebien 1942a: 323). Status: valid genus in Stenochiinae: Cnodalonini.

*Camptobrachys* Kaszab, 1941a: 4, 24 [M]. Type species: *Camptobrachyssulcatus* Kaszab, 1941, by original designation. Status: valid genus in Stenochiinae: Cnodalonini.

*Canariella* Uyttenboogaart, 1929: 341, 342 [F]. Type species: *Canariellaarenapta* Uyttenboogaart, 1929 (= *Philhammussericans* Fairmaire, 1871), by monotypy. Status: junior synonym of *Philhammus* Fairmaire, 1871 in Pimeliinae: Cnemeplatiini: Cnemeplatiina. Synonymy: Uyttenboogaart (1940: 68). Note: junior homonym of *Canariella* Hesse, 1918 [Mollusca].

*Cantaloubeus* Ardoin, 1959b: 203 [M]. Type species: *Cantaloubeusviridis* Ardoin, 1959, by monotypy. Status: valid genus in Tenebrioninae: Amarygmini.

*Cantopipleurus* Koch, 1943a: 578 [M]. Type species: *Tentyriamesostenoides* Baudi di Selve, 1881, by monotypy. Status: valid genus in Pimeliinae: Tentyriini.

*Capeluprops* Schawaller, 2011: 271, 273 [M]. Type species: *Capelupropslaenoides* Schawaller, 2011, by original designation. Status: valid genus in Lagriinae: Lupropini.

*Capicrypticus* Koch, 1950c: 55 [M]. Type species: *Lamprocrypticuscapensis* Koch, 1950, by original designation. Status: valid genus in Diaperinae: Crypticini.

*Capidium* Koch, 1954a: 44 [N]. Type species: *Oncotustardus* Solier, 1848, by original designation. Status: valid genus in Blaptinae: Platynotini: Eurynotina.

*Capnisa* Dejean, 1836: 197 [F]. Type species: *Bradyuskarelini* Faldermann, 1836, by monotypy. Status: junior synonym of *Gnathosia* Fischer, 1821 in Pimeliinae: Tentyriini. Synonymy: Reitter (1896d: 129).

*Capnisiceps* Chatanay, 1914a: 215 [M]. Type species: *Capnisicepsmaindroni* Chatanay, 1914, by original designation. Status: valid genus in Pimeliinae: Tentyriini.

*Capnochroa* J.L. LeConte, 1862: 244 [F]. Type species: *Cistelafuliginosa* Melsheimer, 1846, by monotypy. Status: valid genus in Alleculinae: Alleculini: Gonoderina.

*Capricephalius* Koch, 1943a: 492, 508 [M]. Type species: *Arthrodibiusbazmanicus* Schuster, 1938, by monotypy. Status: valid genus in Pimeliinae: Erodiini.

*Carabelops* Fairmaire, 1899e: 534 [M]. Type species: *Carabelopsaenescens* Fairmaire, 1899, by monotypy. Status: valid genus in Stenochiinae: Cnodalonini.

*Caraboblaps* Bauer, 1921: 231 [F]. Type species: none designated. Status: undetermined taxon in Blaptinae: Blaptini: Blaptina. Note: this genus was described before 1931 (ICZN 1999, Article 12.1); however, we could not find any nominal species that were subsequently and expressly included in *Caraboblaps* and therefore no “originally included nominal species” could be used to fix the type species (ICZN 1999, Article 67.2.2).

*Caracasa* Pic, 1921d: 21 [F]. Type species: *Caracasaaeneipennis* Pic, 1921, by monotypy. Status: valid genus in Stenochiinae: Cnodalonini.

*Carchares* Pascoe, 1887: 12 [M]. Type species: *Carcharesmacer* Pascoe, 1887, by monotypy. Status: valid genus in Tenebrioninae: Scaurini.

*Cardigenius* Solier, 1836: 407, 492 [M]. Type species: *Cardigeniuslaticollis* Solier, 1836, by subsequent designation (Wilke 1922: 276). Status: valid genus and subgenus in Pimeliinae: Asidini.

*Cardiobioramix* Kaszab, 1940a: 154, 183 [M]. Type species: *Bioramixasidioides* Bates, 1879, by original designation. Status: junior synonym of *Euryhelops* Reitter, 1902 in Blaptinae: Platyscelidini. Synonymy: Nabozhenko and Egorov (2020: 266).

*Cardiochianalus* Kaszab, 1940a: 150, 202 [M]. Type species: *Botirassculptipennis* Fairmaire, 1891, by original designation. Status: valid subgenus of *Bioramix* Bates, 1879 in Blaptinae: Platyscelidini. Note: the alternative original spelling *Chardiochianalus*, used by Kaszab (1940a: 202), was rejected by Kaszab (1975a: 4) who acted as the First Reviser (ICZN 1999, Article 24.2.4).

*Cardiogenius* Agassiz, 1846b: 65, 66 [M]. Type species [automatic]: *Cardigeniuslaticollis* Solier, 1836, by subsequent designation (Wilke 1922: 276). Status: junior synonym of *Cardigenius* Solier, 1836 in Pimeliinae: Asidini. Note: unjustified emendation of *Cardigenius* Solier, 1836, not in prevailing usage.

*Cardiosis* Deyrolle, 1867: 81, 235 [F]. Type species: *Cardiosismouffleti* Deyrolle, 1867, by monotypy. Status: valid subgenus of *Zophosis* Latreille, 1802 in Pimeliinae: Zophosini.

*Cardiothorax* Motschulsky, 1860a: 67 [M]. Type species [automatic]: *Thoracophoruswalckenaerii* Hope, 1841, by original designation. Status: valid genus in Lagriinae: Adeliini. Note: replacement name for *Thoracophorus* Hope, 1841.

*Caribanosis* Nabozhenko, Kirejtshuk, Merkl, Varela, Aalbu & Smith, 2016d: 568 [F]. Type species: *Rhypasmaquisqueyanus* Garrido & Varela, 2011, by original designation. Status: valid genus in Pimeliinae: Stenosini: Stenosina.

*Carinosella* Purchart, 2010: 254 [F]. Type species: *Carinosellamaasaiorum* Purchart, 2010, by original designation. Status: valid genus in Pimeliinae: Adelostomini.

*Caristela* Fairmaire, 1894d: 311 [F]. Type species: *Caristelamegalops* Fairmaire, 1894, by monotypy. Status: junior synonym of *Mycetocharina* Seidlitz, 1890 in Alleculinae: Alleculini: Alleculina. Synonymy: Bedel (1897: 36).

*Caroliphosis* Penrith, 1981b: 114, 115 [F]. Type species: *Hesseosisadamantina* Koch, 1958, by original designation. Status: valid subgenus of *Zophosis* Latreille, 1802 in Pimeliinae: Zophosini.

*Carpiella* Koch, 1962b: 148 [F]. Type species: *Carpiellalatisternum* Koch, 1962, by original designation. Status: valid subgenus of *Zophosis* Latreille, 1802 in Pimeliinae: Zophosini.

†*Caryosoma* Haupt, 1950: 114, 125 [N]. Type species: *Caryosomarugosum* Haupt, 1950, by original designation. Status: valid genus in Stenochiinae: incertae sedis. Note: described from Middle Eocene deposits (Germany).

*Casnonidea* Fairmaire, 1882a: 264 [F]. Type species: *Casnonideaholomelaena* Fairmaire, 1882, by subsequent designation (Borchmann 1930a: 444). Status: valid genus and subgenus in Lagriinae: Lagriini: Statirina.

*Catamerus* Fairmaire, 1887b: 290 [M]. Type species: *Catamerusrevoili* Fairmaire, 1887, by monotypy. Status: valid genus in Lagriinae: Pycnocerini.

*Cataphanus* Gebien, 1921a: 325, 346 [M]. Type species: *Cataphanusquadraticollis* Gebien, 1921, by monotypy. Status: valid genus in Stenochiinae: Cnodalonini.

*Cataphronetis* P.H. Lucas, 1846: pl. 30 [F]. Type species: *Cataphronetislevaillantii* P.H. Lucas, 1846 (= *Phtoracrenata* Germar, 1836), by monotypy. Status: junior synonym of *Phtora* Germar, 1836 in Diaperinae: Phaleriini. Synonymy: Lacordaire (1859a: 335).

*Catapiestus* Perty, 1831: xxxviii [M]. Type species: *Catapiestuspiceus* Perty, 1831, by monotypy. Status: valid genus in Stenochiinae: Cnodalonini.

*Catobleps* Blair, 1918: 149 [M]. Type species: *Catoblepsblattoides* Blair, 1918 (= *Blatticephalusadelotopus* Heller, 1918), by original designation. Status: junior synonym of *Blatticephalus* Heller, 1918 in Tenebrioninae: Falsocossyphini. Synonymy: Blair (1919c: 102).

*Catolaena* Reitter, 1900a: 282 [F]. Type species: *Laenaturkestanica* Reitter, 1897, by subsequent designation (Löbl et al. 2008a: 41). Status: junior synonym of *Laena* Dejean, 1821 in Lagriinae: Laenini. Synonymy: Schawaller (2001b: 4).

*Catomidius* Seidlitz, 1895: 791, 792 [M]. Type species: *Hedyphanesrhynchophorus* Seidlitz, 1895, by subsequent designation (Nabozhenko 2008: 38). Status: junior synonym of *Catomus* Allard, 1876 in Tenebrioninae: Helopini: Helopina. Synonymy: Reitter (1922a: 7).

*Catomodontus* Löbl & Merkl in Löbl et al., 2020: 2 [M]. Type species: *Catomuscoronatus* Koch, 1935, by original designation. Status: valid subgenus of *Catomus* Allard, 1876 in Tenebrioninae: Helopini: Helopina. Note: the name was first proposed by Koch (1935: 107, 108) without fixation of a type species in the original publication (ICZN 1999, Article 13.3); Löbl and Merkl (2003: 251) designated *Catomuscoronatus* Koch, 1935 as the type species of Koch’s name but did not explicitly indicate the genus-group name as intentionally new (ICZN 1999, Article 16.1).

*Catomulus* Reitter, 1897a: 302 [M]. Type species: *Catomulussubaeneus* Reitter, 1897 (= *Oxycaraolcesii* Fairmaire, 1883), by monotypy. Status: valid genus in Pimeliinae: Tentyriini.

*Catomus* Allard, 1876a: 4 [M]. Type species: *Catomuspersicus* Allard, 1876, by subsequent designation (Gebien 1943: 409). Status: valid genus and subgenus in Tenebrioninae: Helopini: Helopina.

*Catopherus* Carter, 1918: 713 [M]. Type species: *Catopheruscorpulentus* Carter, 1918 (= *Axynaonchampioni* Blackburn, 1897), by monotypy. Status: junior synonym of *Axynaon* Blackburn, 1897 in Tenebrioninae: Amarygmini. Synonymy: Carter (1920a: 249).

*Caucasohelops* Nabozhenko, 2006: 816 [M]. Type species: *Eustenomacidiussvetlanae* Nabozhenko, 2006, by original designation. Status: valid subgenus of *Eustenomacidius* Nabozhenko, 2006 in Tenebrioninae: Helopini: Cylindrinotina.

*Caucasonotus* Nabozhenko, 2000: 107 [M]. Type species: *Cylindronotusdombaicus* Nabozhenko, 2000, by original designation. Status: valid subgenus of *Nalassus* Mulsant, 1854 in Tenebrioninae: Helopini: Cylindrinotina.

*Caudamarygmus* Bremer, 2001b: 88, 94 [M]. Type species: *Caudamarygmusnotabilis* Bremer, 2001, by original designation. Status: valid genus in Tenebrioninae: Amarygmini.

*Caulostena* Fairmaire, 1896b: 355 [F]. Type species: *Caulostenafoveicollis* Fairmaire, 1896, by monotypy. Status: valid genus in Alleculinae: Alleculini: Mycetocharina.

*Cauricara* Penrith, 1979: 7, 42 [N]. Type species: *Stenocaravelox* Péringuey, 1886, by original designation. Status: valid subgenus of *Stenocara* Solier, 1835 in Pimeliinae: Adesmiini.

*Caverneleodes* Triplehorn, 1975: 39 [M]. Type species: *Eleodeseasterlai* Triplehorn, 1975, by original designation. Status: valid subgenus of *Eleodes* Eschscholtz, 1829 in Blaptinae: Amphidorini.

*Caxtonana* Buck, 1960: 224 [F]. Type species: *Caxtonanacostata* Buck, 1960, by monotypy. Status: junior synonym of *Apterotheca* Gebien, 1921 in Stenochiinae: Cnodalonini. Synonymy: Bouchard (2002: 452).

*Cechenosternum* Gebien, 1921b: 9 [N]. Type species: *Cechenosternumnigromaculatum* Gebien, 1921, by subsequent designation (Gebien 1939: 748). Status: valid genus in Diaperinae: Crypticini.

*Cecrops* Gistel, 1834: 21 [M]. Type species: *Tenebriogigas* Linnaeus, 1763, by subsequent designation (Bousquet and Bouchard 2017: 131). Status: junior synonym of *Mylaris* Pallas, 1781 in Stenochiinae: Cnodalonini. Synonymy: Bousquet and Bouchard (2017: 131).

*Cedrosius* Fairmaire, 1902b: 332 [M]. Type species: *Cedrosiuscalosomoides* Fairmaire, 1902, by monotypy. Status: valid genus in Tenebrioninae: Tenebrionini.

*Celebesa* Pic, 1921d: 23 [F]. Type species: *Celebesaelongata* Pic, 1921, by subsequent designation (Riley 1923: 126). Status: valid genus in Stenochiinae: Cnodalonini.

*Celibe* Boisduval, 1835: 262 [F]. Type species: *Celibeaustralis* Boisduval, 1835, by subsequent designation (Watt 1968: 36). Status: valid genus in Tenebrioninae: Heleini: Heleina.

*Celox* Gistel, 1848a: 126 [F]. Type species [automatic]: *Cnodalonirroratum* Dalman, 1823, by subsequent designation (Hope 1841: 133). Status: junior synonym of *Campsia* Lepeletier & Audinet-Serville, 1828 in Stenochiinae: Cnodalonini. Note: unnecessary replacement name for *Campsia* Lepeletier & Audinet-Serville, 1828.

*Cenophorus* Mulsant & Rey, 1859a: 113 [M]. Type species: *Cenophorusviduus* Mulsant & Rey, 1859, by monotypy. Status: valid genus in Blaptinae: Opatrini: Blapstinina.

*Cenoscelis* Wollaston, 1868: 179 [F]. Type species: *Cenoscelistibialis* Wollaston, 1868 (= *Ulomapullum* Erichson, 1843), by monotypy. Status: valid genus and subgenus in Tenebrioninae: Ulomini.

*Centorus* Mulsant, 1854: 272 [M]. Type species: *Calcarprocerum* Mulsant, 1854, by monotypy. Status: valid genus and subgenus in Lagriinae: Belopini.

*Centrioptera* Mannerheim, 1843: 279 [F]. Type species: *Centriopteracaraboides* Mannerheim, 1843, by monotypy. Status: junior synonym of *Cryptoglossa* Solier, 1837 in Pimeliinae: Cryptoglossini. Synonymy: Aalbu et al. (2002: 486).

*Centrocnemis* Kraatz in Heyden and Kraatz, 1882: 330 [F]. Type species: *Centrocnemismollis* Kraatz, 1882, by monotypy. Status: senior synonym of *Centrocnemita* Strand, 1935 in Pimeliinae: Pimeliini. Note: junior homonym of *Centrocnemis* Signoret, 1852 [Hemiptera].

*Centrocnemita* Strand, 1935a: 284 [F]. Type species [automatic]: *Centrocnemismollis* Kraatz, 1882, by monotypy. Status: valid subgenus of *Lasiostola* Dejean, 1834 in Pimeliinae: Pimeliini. Note: replacement name for *Centrocnemis* Kraatz, 1882.

*Centronopus* Solier, 1848: 153, 154, 258 [M]. Type species: *Centronopusextensicollis* Solier, 1848 (= *Tenebriosuppressus* Say, 1835), by original designation. Status: valid genus and subgenus in Tenebrioninae: Centronopini.

*Centropus* Jakobson, 1914: 528 [M]. Type species [automatic]: *Centronopusextensicollis* Solier, 1848 (= *Tenebriosuppressus* Say, 1835), by original designation. Status: junior synonym of *Centronopus* Solier, 1848 in Tenebrioninae: Centronopini. Note: unjustified emendation of *Centronopus* Solier, 1848, not in prevailing usage; junior homonym of *Centropus* Illiger, 1811 [Aves].

*Cephacerus* Rafinesque, 1815: 113 [M]. Type species [automatic]: *Erodiusgibbus* Fabricius, 1775, by subsequent designation (Latreille 1810: 429). Status: junior synonym of *Erodius* Fabricius, 1775 in Pimeliinae: Erodiini. Note: unnecessary replacement name for *Erodius* Fabricius, 1775.

*Cephaladesmia* Gebien, 1920: 49, 61 [F]. Type species: *Cephaladesmiathomseni* Gebien, 1920, by monotypy. Status: junior synonym of *Stenodesia* Reitter, 1916 in Pimeliinae: Adesmiini. Synonymy: Penrith (1979: 70, 85).

*Cephalamarygmus* Bremer, 2001b: 92, 95 [M]. Type species: *Amarygmuspreangerensis* Pic, 1952, by original designation. Status: valid genus in Tenebrioninae: Amarygmini.

*Cephaleucyrtus* Pic, 1923c: 22 [M]. Type species: *Cephaleucyrtusviridicollis* Pic, 1923, by monotypy. Status: junior synonym of *Gauromaia* Pascoe, 1866 in Stenochiinae: Cnodalonini. Synonymy: Gebien (1941: 1144).

*Cephaloplonyx* Pic, 1922c: 12 [M]. Type species: *Cephaloplonyxopacus* Pic, 1922 (= *Helopsdentipes* Fabricius, 1781), by monotypy. Status: junior synonym of *Hoplobrachium* Fairmaire, 1886 in Tenebrioninae: Amarygmini. Synonymy: Gebien (1943: 922).

*Cephalostenus* Solier, 1838b: 160, 184 [M]. Type species: *Cephalostenusdejeanii* Solier, 1838 (= *Scauruselegans* Brullé, 1832), by subsequent designation (Hope 1841: 115). Status: valid genus in Tenebrioninae: Scaurini.

*Cephalothydemus* Pic, 1923c: 24 [M]. Type species: *Cephalothydemustheresae* Pic, 1923, by monotypy. Status: valid genus in Stenochiinae: Cnodalonini.

*Ceradelium* Preudhomme de Borre, 1868: 126 [N]. Type species: *Ceradeliumarmatum* Preudhomme de Borre, 1868 (= *Blepegenesaruspex* Pascoe, 1868), by monotypy. Status: junior synonym of *Blepegenes* Pascoe, 1868 in Lagriinae: Adeliini. Synonymy: Pascoe (1869: 41).

*Ceradesmia* Gebien, 1920: 49, 63 [F]. Type species: *Stenocaraalbicolle* Haag-Rutenberg, 1878, by original designation. Status: valid subgenus of *Metriopus* Solier, 1835 in Pimeliinae: Adesmiini.

*Ceramba* Fauvel, 1904: 206 [F]. Type species: *Cerambahydrovatina* Fauvel, 1904, by monotypy. Status: junior synonym of *Menimus* Sharp, 1876 in Diaperinae: Gnathidiini: Gnathidiina. Synonymy: Gebien (1940: 429).

*Cerandria* Dejean, 1834: 200 [F]. Type species: *Trogossitacornuta* Fabricius, 1798, by subsequent designation (Duponchel 1842b: 285). Status: junior synonym of *Gnatocerus* Thunberg, 1814 in Diaperinae: Diaperini: Adelinina. Synonymy: Erichson (1847a: 119).

*Cerandrosus* Gebien, 1921a: 325, 394 [M]. Type species: *Cerandrosusnodipennis* Gebien, 1921, by monotypy. Status: valid genus in Stenochiinae: Cnodalonini.

*Cerasoma* Endrödy-Younga, 1989: 113 [N]. Type species: *Cerasomacerasus* Endrödy-Younga, 1989, by original designation. Status: valid genus in Pimeliinae: Cryptochilini: Cryptochilina.

*Ceratanisus* Gemminger in Gemminger and Harold, 1870: 1818 [M]. Type species [automatic]: *Anisocerustristis* Faldermann, 1837, by monotypy. Status: valid genus in Pimeliinae: Ceratanisini. Note: replacement name for *Anisocerus* Faldermann, 1837.

*Ceratoma* Borchmann, 1916a: 49 [F]. Type species: none designated. Status: undetermined taxon in Lagriinae: Lagriini: Lagriina. Note: this genus was included in a key, which fulfils the criteria of availability for new genus-group names proposed before 1931 (ICZN 1999, Article 12.1); however, we could not find any nominal species that were subsequently and expressly included in *Ceratoma* and therefore no “originally included nominal species” could be used to fix the type species (ICZN 1999, Article 67.2.2).

*Ceratopelius* Antoine, 1963: 52, 54 [M]. Type species: *Ceratopeliusmussardi* Antoine, 1963, by original designation. Status: valid genus in Tenebrioninae: Helopini: Cylindrinotina.

*Ceratupis* Perty, 1830: 57 [F]. Type species: *Ceratupisnigerrima* Perty, 1830, by monotypy. Status: junior synonym of *Antimachus* Gistel, 1829 in Tenebrioninae: Ulomini. Synonymy: Lacordaire (1859a: 330).

*Cerenopus* J.L. LeConte, 1851: 143 [M]. Type species: *Cerenopusconcolor* J.L. LeConte, 1851, by subsequent designation (R. Lucas 1920: 173). Status: valid genus in Tenebrioninae: Cerenopini.

*Cerocamptus* Gebien, 1919: 28, 151 [M]. Type species: *Camariamalayana* Fairmaire, 1893, by original designation. Status: valid genus in Stenochiinae: Cnodalonini.

*Cerodolus* Sharp, 1886: 410 [M]. Type species: *Cerodoluschrysomeloides* Sharp, 1886, by monotypy. Status: valid genus in Tenebrioninae: Heleini: incertae sedis. Note: transferred from Titaenini by Matthews and Lawrence (2019: 628).

*Cerogria* Borchmann, 1911: 210 [F]. Type species: *Lagriaanisocera* Wiedemann, 1823, by subsequent designation (Borchmann 1936: 119). Status: valid genus and subgenus in Lagriinae: Lagriini: Lagriina. Note: as mentioned by Löbl et al. (2020: 5), discovery of the earlier designation of *Cerogriadohrni* Borchmann, 1911 as the type species of *Cerogria* (by subsequent designation by R. Lucas (1920: 174)) threatens the nomenclatural stability of this genus since *C.dohrni* is the type species of the valid genus *Acerogria* Borchmann, 1936; we recommend that an application be submitted to the International Commission on Zoological Nomenclature to maintain the type species designation proposed by Borchmann (1936: 119).

*Cerogriodes* Borchmann, 1941b: 25 [M]. Type species: *Cerogriaklapperichi* Borchmann, 1941, by monotypy. Status: junior synonym of *Cerogria* Borchmann, 1911 in Lagriinae: Lagriini: Lagriina. Synonymy: Merkl (2007: 262).

*Ceromelaephus* Koch, 1955a: 87 [M]. Type species: *Trachynotusbadeni* Haag-Rutenberg, 1873, by original designation. Status: valid subgenus of *Somaticus* Hope, 1841 in Pimeliinae: Sepidiini: Trachynotina.

*Ceropria* Laporte & Brullé, 1831: 332, 396 [F]. Type species: *Helopsindutus* Wiedemann, 1819, by subsequent designation (Duponchel 1844b: 359). Status: valid genus in Diaperinae: Diaperini: Diaperina.

*Cerosis* Gebien, 1920: 33, 35 [F]. Type species: *Cerosishereroensis* Gebien, 1920, by monotypy. Status: valid subgenus of *Zophosis* Latreille, 1802 in Pimeliinae: Zophosini.

*Cerostena* Solier, 1836: 307, 325 [F]. Type species: *Cerostenadeplanata* Solier, 1836, by **present designation**. Status: junior synonym of *Psectrascelis* Solier, 1836 in Pimeliinae: Nycteliini. Synonymy: Fairmaire (1876: 356).

*Cerostira* Borchmann, 1942a: 36 [F]. Type species [automatic]: *Porrolagriasubaenea* Borchmann, 1908, by subsequent designation (Borchmann 1936: 227). Status: valid genus in Lagriinae: Lagriina. Note: replacement name for *Allocera* Borchmann, 1916.

*Cerysia* Bremer, 2001a: 68 [F]. Type species: *Elixotalaevicornis* Blair, 1929, by original designation. Status: valid genus in Tenebrioninae: Amarygmini.

*Cestrinus* Erichson, 1842a: 172 [M]. Type species: *Cestrinustrivialis* Erichson, 1842, by subsequent designation (Blair 1919a: 529). Status: junior synonym of *Isopteron* Hope, 1841 in Lagriinae: Adeliini. Synonymy: Champion (1894a: 354), Doyen et al. (1990: 231).

*Chaerodes* White, 1846: 12 [M]. Type species: *Chaerodestrachyscelides* White, 1846, by monotypy. Status: valid genus in Lagriinae: Chaerodini.

*Chaeroplonyx* Bremer, 2014a: 36 [M]. Type species: *Plesiophthalmuskimanisensis* Masumoto, 2001, by original designation. Status: valid subgenus of *Plesiophthalmus* Motschulsky, 1857 in Tenebrioninae: Amarygmini.

*Chaetopsia* Gebien, 1925c: 567 [F]. Type species: *Chaetopsiaangusticollis* Gebien, 1925, by monotypy. Status: valid genus in Stenochiinae: Cnodalonini.

*Chaetotoma* Motschulsky, 1860c: 533 [F]. Type species: *Tenebriocephalotes* Pallas, 1781, by subsequent designation (Löbl et al. 2008a: 41). Status: valid subgenus of *Pimelia* Fabricius, 1775 in Pimeliinae: Pimeliini.

*Chaetyllus* Pascoe, 1860b: 122 [M]. Type species: *Chaetyllusanthicoides* Pascoe, 1860, by monotypy. Status: valid genus in Lagriinae: Laenini. Note: placed in the tribe Laenini by Kanda (2016: 562).

*Chalcocyclus* Fairmaire, 1884d: 74 [M]. Type species: *Chalcocyclusspeculifer* Fairmaire, 1884, by monotypy. Status: valid genus in Stenochiinae: Cnodalonini.

*Chalcopauliana* Ardoin, 1961a: 209 [F]. Type species: *Chalcopaulianavinsoni* Ardoin, 1961, by monotypy. Status: valid genus in Stenochiinae: Cnodalonini.

*Chalcoplonyx* Ardoin, 1963b: 308, 333 [M]. Type species: *Gonocnemisviridis* Kraatz, 1899, by original designation. Status: valid genus in Tenebrioninae: Amarygmini.

*Chalcopteroides* Gebien, 1948: 497 [M]. Type species [automatic]: *Chalcopterusiridicolor* Blessig, 1861, by subsequent designation (Gebien 1948: 497). Status: valid genus in Tenebrioninae: Amarygmini. Note: replacement name for *Chalcopterus* Blessig, 1861; *Chalcopteroides* was proposed earlier by Strand (1935b: 302); however, this name is unavailable because Strand did not designate a type species for the nominal taxon, a mandatory requirement for replacement name without valid typification proposed after 1930 (ICZN 1999; Article 13.3.1).

*Chalcopterus* Blessig, 1861: 103 [M]. Type species: *Chalcopterusiridicolor* Blessig, 1861, by subsequent designation (Gebien 1948: 497). Status: senior synonym of *Chalcopteroides* Gebien, 1948 in Tenebrioninae: Amarygmini. Note: junior homonym of *Chalcopterus* Reichenbach, 1852 [Aves].

*Chalcostylus* Fairmaire, 1898b: 409 [M]. Type species: *Chalcostylusperrieri* Fairmaire, 1898, by monotypy. Status: valid genus in Tenebrioninae: Toxicini: Nycteropina.

*Charianus* Bates, 1879a: 297 [M]. Type species: *Tetraphylluspurpuratus* Coquerel, 1851, by original designation. Status: valid genus in Stenochiinae: Cnodalonini.

*Chariophenus* Blair, 1929a: 239 [M]. Type species: *Chariophenuswasmanni* Blair, 1929, by original designation. Status: junior synonym of *Foochounus* Pic, 1921 in Stenochiinae: Cnodalonini. Synonymy: Schawaller and Ando (2009: 260).

*Chariotheca* Pascoe, 1860b: 125 [F]. Type species: *Chariothecacoruscans* Pascoe, 1860, by subsequent designation (Gebien 1942a: 335). Status: valid genus in Stenochiinae: Cnodalonini.

*Chariothes* Carter, 1914b: 78 [M]. Type species [automatic]: *Chariothecacoruscans* Pascoe, 1860, by subsequent designation (Gebien 1942a: 335). Status: junior synonym of *Chariotheca* Pascoe, 1860 in Stenochiinae: Cnodalonini. Note: unnecessary replacement name for *Chariotheca* Pascoe, 1860.

*Charisius* Champion, 1888: 421 [M]. Type species: *Charisiusfasciatus* Champion, 1888, by subsequent designation (R. Lucas 1920: 178). Status: valid genus in Alleculinae: Alleculini: Alleculina. Note: the First Reviser (*Charisius* Champion, 1888 versus *Narses* Champion, 1888) is Campbell (2014: 271).

*Chartopteryx* Westwood, 1841a: 43 [F]. Type species: *Chartopteryxchildrenii* Westwood, 1841, by monotypy. Status: junior synonym of *Cyphaleus* Westwood, 1841 in Tenebrioninae: Heleini: Cyphaleina. Synonymy and First Reviser action (*Chartopteryx* Westwood, 1841 versus *Cyphaleus* Westwood, 1841): Matthews (1992: 490).

*Chaseleodes* Thomas, 2015: 122 [F]. Type species: *Eleodescurta* Champion, 1884, by original designation. Status: valid subgenus of *Eleodes* Eschscholtz, 1829 in Blaptinae: Amphidorini.

*Chatanayus* Ardoin, 1957: 61 [M]. Type species: *Chemolanusvillosipes* Fairmaire, 1884, by original designation. Status: senior synonym of *Anachayus* Bouchard & Bousquet, **nom. nov.** in Stenochiinae: Cnodalonini. Note: junior homonym of *Chatanayus* Fleutiaux 1940 [Coleoptera: Elateridae].

*Cheilopoma* Murray, 1867: 20 [N]. Type species: *Cheilopomacastaneum* Murray, 1867, by monotypy. Status: valid genus in Diaperinae: Hypophlaeini.

*Cheirodes* Gené, 1839: 73 [M]. Type species: *Cheirodessardous* Gené, 1839, by monotypy. Status: valid genus and subgenus in Tenebrioninae: Melanimonini.

*Cheiroplus* Ardoin, 1963a: 129 [M]. Type species: *Cheiroplusfreyi* Ardoin, 1963, by monotypy. Status: valid genus in Tenebrioninae: Amarygmini. Note: the earlier usage of *Cheiroplus* by Ardoin (1962b: 970) is unavailable since it was published after 1930 without fixation of a type species in the original publication (ICZN 1999, Article 13.3).

*Cheirosis* Deyrolle, 1867: 81, 220 [F]. Type species: *Zophosisovata* Faldermann, 1837 (= *Pedinusacuminatus* Fischer, 1832), by monotypy. Status: valid subgenus of *Zophosis* Latreille, 1802 in Pimeliinae: Zophosini.

*Chemolanus* Bates, 1879a: 296 [M]. Type species: *Tetraphyllusconsobrinus* Fairmaire, 1868 (= *Camariaobscura* Klug, 1833), by original designation. Status: valid genus in Stenochiinae: Cnodalonini.

*Cherostus* C.O. Waterhouse, 1894: 68 [M]. Type species: *Cherostuswalkeri* C.O. Waterhouse, 1894, by subsequent designation (Barber 1914: 191). Status: junior synonym of *Rhipidandrus* J.L. LeConte, 1862 in Tenebrioninae: Bolitophagini. Synonymy: Gebien (1939: 762).

*Chianalus* Bates, 1879b: 479 [M]. Type species: *Chianaluscostipennis* Bates, 1879, by monotypy. Status: valid subgenus of *Bioramix* Bates, 1879 in Blaptinae: Platyscelidini.

*Chilenolagria* Pic, 1936a: 28 [F]. Type species: *Chilenolagrialimbata* Pic, 1936, by monotypy. Status: valid genus in Lagriinae: Lagriini: Lagriina.

*Chileone* Bates, 1868: 264 [F]. Type species: *Chileonedeyrollei* Bates, 1868, by monotypy. Status: junior synonym of *Hypaulax* Bates, 1868 in Stenochiinae: Cnodalonini. Synonymy: Carter (1914b: 46).

*Chiliarchum* Koch, 1953a: 263 [N]. Type species: *Molurisbertolonii* Guérin-Méneville, 1844, by original designation. Status: valid genus in Pimeliinae: Sepidiini: Molurina. Note: elevated to rank of genus (from subgenus of *Ocnodes* Fåhraeus, 1870) by Kamiński et al. (2020: 9, 17)

*Chilometopon* Horn, 1874: 31 [N]. Type species: *Trimytisabnormis* Horn, 1870, by subsequent designation (Casey 1907: 367). Status: valid genus in Pimeliinae: Edrotini.

*Chinotrigon* Skopin, 1973: 170 [M]. Type species: *Trigonoscelissublaevigata* Reitter, 1887, by original designation. Status: valid subgenus of *Trigonoscelis* Dejean, 1834 in Pimeliinae: Pimeliini.

*Chirocharis* Kolbe, 1903: 166, 179 [F]. Type species: *Chiroscelisaustralis* Westwood, 1845, by monotypy. Status: valid genus in Lagriinae: Pycnocerini.

*Chirodes* Agassiz, 1846b: 78, 81 [M]. Type species [automatic]: *Cheirodessardous* Gené, 1839, by monotypy. Status: junior synonym of *Cheirodes* Gené, 1839 in Tenebrioninae: Melanimonini. Note: unjustified emendation of *Cheirodes* Gené, 1839, not in prevailing usage.

*Chiroscelis* Lamarck, 1804: 261 [F]. Type species: *Chiroscelisbifenestra* Lamarck, 1804 (= *Tenebriodigitatus* Fabricius, 1801), by monotypy. Status: valid genus in Lagriinae: Pycnocerini.

*Chirosis* Gemminger in Gemminger and Harold, 1870: 1806 [F]. Type species [automatic]: *Zophosisovata* Faldermann, 1837 (= *Pedinusacuminatus* Fischer, 1832), by monotypy. Status: junior synonym of *Cheirosis* Deyrolle, 1867 in Pimeliinae: Zophosini. Note: unjustified emendation of *Cheirosis* Deyrolle, 1867, not in prevailing usage.

*Chitwania* Novák, 2015d: 91 [F]. Type species: *Chitwaniakejvali* Novák, 2015, by original designation. Status: valid genus in Alleculinae: Alleculini: Alleculina.

*Chlamydion* Gistel, 1848a: x [N]. Type species [automatic]: *Opatrumorientale* Fabricius, 1775, by subsequent designation (Hope 1841: 110). Status: junior synonym of *Sclerum* Dejean, 1834 in Blaptinae: Opatrini: Sclerina. Note: unnecessary replacement name for *Sclerum* Dejean, 1834.

*Chlorocamma* Bates, 1873e: 371 [N]. Type species: *Chlorocammacarenipenne* Bates, 1873 (= *Leptomorphasulcata* Montrouzier, 1860), by subsequent designation (Gebien 1942a: 755). Status: valid genus in Stenochiinae: Cnodalonini.

*Chlorophanes* Matthews, 1992: 451, 461 [M]. Type species: *Platyphanespunctipennis* Carter, 1911, by original designation. Status: senior synonym of *Phanechloros* Matthews & Bouchard, 2008 in Tenebrioninae: Heleini: Cyphaleina. Note: junior homonym of *Chlorophanes* Reichenbach, 1853 [Aves].

*Chlorophila* Semenov, 1891: 374 [F]. Type species: *Lagriaportschinskii* Semenov, 1891, by monotypy. Status: valid genus in Lagriinae: Lagriini: Statirina.

*Choaspes* Champion, 1885: 118 [M]. Type species: *Choaspespurpureus* Champion, 1885, by subsequent designation (Gebien 1941: 338). Status: senior synonym of *Choastes* Champion, 1893 in Stenochiinae: Cnodalonini. Note: junior homonym of *Choaspes* Moore, 1881 [Lepidoptera].

*Choastes* Champion, 1893a: 526 [M]. Type species [automatic]: *Choaspespurpureus* Champion, 1885, by subsequent designation (Gebien 1941: 338). Status: valid genus in Stenochiinae: Cnodalonini. Note: replacement name for *Choaspes* Champion, 1885.

*Choerodes* Gemminger in Gemminger and Harold, 1870: 1944 [M]. Type species [automatic]: *Chaerodestrachyscelides* White, 1846, by monotypy. Status: junior synonym of *Chaerodes* White, 1846 in Lagriinae: Chaerodini. Note: unjustified emendation of *Chaerodes* White, 1846, not in prevailing usage; junior homonym of *Choerodes* Leidy, 1852 [Mammalia] and *Choerodes* Guenée, 1857 [Lepidoptera].

*Cholipus* Pascoe, 1866a: 471 [M]. Type species: *Cholipusbrevicornis* Pascoe, 1866, by original designation. Status: junior synonym of *Derosphaerus* J. Thomson, 1858 in Stenochiinae: Cnodalonini. Synonymy: Mäklin (1863a: 552, with *Encyalesthus* Motschulsky, 1860, a junior synonym of *Derosphaerus* J. Thomson, 1858).

*Chorasmius* Bates, 1868: 310 [M]. Type species: *Evaniosomusprocerus* Erichson, 1847, by monotypy. Status: valid genus in Pimeliinae: Evaniosomini.

*Choresmolamus* G.S. Medvedev, 1978: 150 [M]. Type species: *Dilamusoxianus* G.S. Medvedev, 1978, by original designation. Status: valid subgenus of *Dilamus* Jacquelin du Val, 1861 in Blaptinae: Opatrini: Ammobiina.

*Choristopsis* Kraatz, 1865: 81, 227 [F]. Type species: *Choristopsiscaucasica* Kraatz, 1865, by monotypy. Status: junior synonym of *Calyptopsis* Solier, 1835 in Pimeliinae: Tentyriini. Synonymy: Reitter (1900c: 130).

*Chromatia* J.L. LeConte, 1862: 244 [F]. Type species: *Cistelaamoena* Say, 1824, by monotypy. Status: valid genus in Alleculinae: Alleculini: Gonoderina.

*Chromomaea* Pascoe, 1866a: 490 [F]. Type species: *Chromomaeapicta* Pascoe, 1866, by monotypy. Status: junior synonym of *Lepturidea* Fauvel, 1862 in Alleculinae: Alleculini: Alleculina. Synonymy: Fauvel (1905: 225).

*Chrysobalus* Boisduval, 1835: 267 [M]. Type species: *Chrysobalusfulgidipennis* Boisduval, 1835, by monotypy. Status: senior synonym of *Cyphaleus* Westwood, 1841 in Tenebrioninae: Heleini: Cyphaleina. Synonymy: Lacordaire (1859b: 409); although the identity of the type species is uncertain (Matthews 1992: 439, Matthews and Bouchard 2008: 350), it is probably a species of *Cyphaleus* Westwood, 1841. Note: nomen oblitum; we provide references to support the conservation of *Cyphaleus* Westwood, 1841 as the valid name for this genus (ICZN 1999, Article 23.9.1) in Appendix 2.

*Chrysolagria* Seidlitz, 1898b: 336, 339 [F]. Type species: *Lagriaviridipennis* Fabricius, 1798, by monotypy. Status: valid genus in Lagriinae: Lagriini: Lagriina.

*Chrysolinoides* Jolivet, 1951: 2 [M]. Type species: *Chrysolinoidesphilippinensis* Jolivet, 1951 (= *Hemicerairidicolor* Gebien, 1921), by monotypy. Status: junior synonym of *Hemicera* Laporte & Brullé, 1831 in Stenochiinae: Cnodalonini. Synonymy: Kulzer (1952: 764). Note: genus originally described in Chrysomeloidea: Chrysomelidae.

*Chrysomaia* Kulzer, 1952: 755, 756 [F]. Type species: *Eucyrtuscarbunculus* Fairmaire, 1885, by original designation. Status: junior synonym of *Augolesthus* Motschulsky, 1872 in Stenochiinae: Cnodalonini. Synonymy: Kaszab (1983a: 134).

*Chrysopeplus* Gebien, 1942a: 755 [M]. Type species [automatic]: *Helopsexpolitus* Broun, 1880, by original designation. Status: valid genus in Stenochiinae: Cnodalonini. Note: replacement name for *Leiopeplus* Broun, 1893.

*Cibdelis* Mannerheim, 1843: 282 [F]. Type species: *Cibdelisblaschkii* Mannerheim, 1843, by monotypy. Status: valid genus in Stenochiinae: Cnodalonini.

*Cilioncotus* Koch, 1954a: 41 [M]. Type species: *Oncotusnamaquanus* Koch, 1954, by original designation. Status: valid subgenus of *Oncotus* Blanchard, 1845 in Blaptinae: Platynotini: Eurynotina.

*Cillibus* Matthews, 1993: 1040, 1078 [M]. Type species: *Saragusblackburni* W.J. MacLeay, 1888, by original designation. Status: valid genus in Tenebrioninae: Heleini: Heleina.

*Cimicia* Fairmaire, 1891a: lxxxix [F]. Type species: *Cimiciaspinipes* Fairmaire, 1891, by monotypy. Status: valid genus in Pimeliinae: Adelostomini.

*Cimicichora* Koch, 1952b: 38 [F]. Type species: *Cimicichoracrenulata* Koch, 1952, by original designation. Status: valid genus in Pimeliinae: Adelostomini.

*Cimiciopsis* Koch, 1952b: 21, 87 [F]. Type species: *Cimiciopsiscastleae* Koch, 1952, by original designation. Status: valid genus in Pimeliinae: Adelostomini.

*Cimipsa* Peyerimhoff, 1911: 346 [F]. Type species: *Cimipsasergenti* Peyerimhoff, 1911, by monotypy. Status: valid genus in Pimeliinae: Tentyriini.

*Circomus* Fleischer, 1900: 236 [M]. Type species: *Hypophlaeussubdepressus* Wollaston, 1864, by monotypy. Status: junior synonym of *Palorus* Mulsant, 1854 in Tenebrioninae: Palorini. Synonymy: Löbl et al. (2008b: 276).

*Cirsa* P.H. Lucas, 1857: lvi [F]. Type species: *Cirsastriaticollis* P.H. Lucas, 1857, by monotypy. Status: valid subgenus of *Micipsa* P.H. Lucas, 1855 in Pimeliinae: Tentyriini.

*Cirta* Gemminger in Gemminger and Harold, 1870: 1831 [F]. Type species [automatic]: *Cirsastriaticollis* P.H. Lucas, 1857, by monotypy. Status: junior synonym of *Cirsa* P.H. Lucas, 1857 in Pimeliinae: Tentyriini. Note: unjustified emendation of *Cirsa* P.H. Lucas, 1857, not in prevailing usage.

*Cissides* Chatanay, 1915a: 475, 495 [M]. Type species: *Heterophyluspunctatissimus* Fairmaire, 1869, by original designation. Status: valid genus in Diaperinae: Diaperini: Diaperina.

*Cistela* Fabricius, 1775: 116 [F]. Type species: *Chrysomelasulphurea* Linnaeus, 1758, by subsequent designation (Latreille 1810: 429). Status: senior synonym of *Cteniopus* Solier, 1835 in Alleculinae: Cteniopodini. Note: junior homonym of *Cistela* Geoffroy, 1762 [Coleoptera: Byrrhidae]. Note: the older name *Cistela* Geoffroy, 1762 was suppressed for purposes of the Principle of Priority but not for those of the Principle of Homonymy (ICZN 1994a, Opinion 1754).

*Cistelampra* Fairmaire, 1897b: 386 [F]. Type species: *Cistelamprapurpurina* Fairmaire, 1897, by monotypy. Status: valid genus in Alleculinae: Alleculini: Alleculina.

*Cistelina* Seidlitz, 1896: 195 [F]. Type species: *Cisteladavidis* Fairmaire, 1878, by subsequent designation (R. Lucas 1920: 187). Status: valid genus in Alleculinae: Cteniopodini.

*Cistella* Gistel, 1848a: xi [F]. Type species [automatic]: *Chrysomelasulphurea* Linnaeus, 1758, by subsequent designation (Latreille 1810: 429). Status: junior synonym of *Cteniopus* Solier, 1835 in Alleculinae: Cteniopodini. Note: unjustified emendation of *Cistela* Fabricius, 1775, not in prevailing usage.

*Cistelodema* Borchmann, 1932a: 380 [F]. Type species: *Pseudocistelacyanea* Pic, 1930, by original designation. Status: valid genus in Alleculinae: incertae sedis.

*Cisteloida* Fairmaire, 1882a: 256 [F]. Type species: *Cisteloidacastanescens* Fairmaire, 1882, by monotypy. Status: valid genus in Alleculinae: Alleculini: Alleculina.

*Cistelomorpha* Redtenbacher, 1868: 134 [F]. Type species: *Cistelomorphastraminea* Redtenbacher, 1868, by subsequent designation (Borchmann 1932a: 358). Status: valid genus in Alleculinae: Cteniopodini.

*Cistelopsis* Fairmaire, 1896a: 39 [F]. Type species: *Cistelopsisrufina* Fairmaire, 1896, by subsequent designation (R. Lucas 1920: 187). Status: valid genus in Alleculinae: Alleculini: Alleculina.

*Clamoris* Gozis, 1886: 25 [F]. Type species [automatic]: *Phtoracrenata* Mulsant, 1854 (= *Clamorisinsurgens* Gozis, 1886), by monotypy. Status: valid genus in Phrenapatinae: Penetini. Note: replacement name for *Phtora* Mulsant, 1854.

*Clastopus* Fairmaire, 1898b: 407 [M]. Type species: *Clastopuseurynotoides* Fairmaire, 1898, by monotypy. Status: valid genus in Blaptinae: Platynotini: Platynotina.

*Claudegirardius* Iwan, 1999b: 372 [M]. Type species: *Claudegirardiusbertiae* Iwan, 1999, by original designation. Status: valid genus in Blaptinae: Platynotini: Platynotina.

*Clavatoodescelis* Kaszab, 1940b: 942, 974 [F]. Type species: *Platyscelismelas* Fischer, 1823, by original designation. Status: valid subgenus of *Oodescelis* Motschulsky, 1845 in Blaptinae: Platyscelidini. Note: the First Reviser (*Clavatoodescelis* Kaszab, 1940 versus *Oblongoodescelis* Kaszab, 1940) is Egorov (2020: 380).

*Cleognathus* Gebien, 1921b: 154 [M]. Type species: *Cleognathusprosternalis* Gebien, 1921, by monotypy. Status: valid genus in Tenebrioninae: Amarygmini.

*Cleolaus* Champion, 1886: 142 [M]. Type species: *Penetasommeri* Lacordaire, 1859, by original designation. Status: valid genus in Phrenapatinae: Penetini.

*Cleomis* Fairmaire, 1892c: 54 [M]. Type species: *Cleomisviolaceipes* Fairmaire, 1892, by subsequent designation (R. Lucas 1920: 189). Status: valid genus in Stenochiinae: Cnodalonini. Note: removed from synonymy with *Psydus* Pascoe, 1868 by Kaszab (1983b: 382).

*Clinocranion* Solier, 1843: 114 [N]. Type species: *Clinocranionspinosum* Solier, 1843, by subsequent designation (R. Lucas 1920: 190). Status: valid subgenus of *Somaticus* Hope, 1841 in Pimeliinae: Sepidiini: Trachynotina. Note: the alternative original spelling *Clynocranion*, used by Solier (1843: 4), was rejected by Koch (1955a: 70) who acted as the First Reviser.

*Clitobius* Mulsant & Rey, 1859c: 141 [M]. Type species: *Clitobiussabulicola* Mulsant & Rey, 1859 (= *Opatrumovatum* Erichson, 1843), by monotypy. Status: valid genus and subgenus in Blaptinae: Opatrini: Ammobiina. Note: the original combination of the accepted name of the type species, *Opatrumovatum* Erichson, 1843, is a junior primary homonym of *Opatrumovatum* Fabricius, 1801.

*Clypeophtora* F. Soldati & L. Soldati, 2003: 4 [F]. Type species: *Phtoratronqueti* F. Soldati & L. Soldati, 2003, by original designation. Status: valid subgenus of *Phtora* Germar, 1836 in Diaperinae: Phaleriini.

*Cnecosochara* Reitter, 1913: 660 [F]. Type species: *Cnecosocharapetriiformis* Reitter, 1913, by monotypy. Status: valid genus in Alleculinae: Cteniopodini.

*Cnemandrosus* Gebien, 1927: 42 [M]. Type species: *Eucyrtussemipurpureus* Fairmaire, 1896, by original designation. Status: junior synonym of *Plamius* Fairmaire, 1896 in Stenochiinae: Cnodalonini. Synonymy: Gebien (1941: 1143).

*Cnemeplatia* Costa, 1847: 146 [F]. Type species: *Cnemeplatiaatropos* Costa, 1847, by monotypy. Status: valid genus in Pimeliinae: Cnemeplatiini: Cnemeplatiina.

*Cnemodasus* Gebien, 1914b: 374 [M]. Type species: *Cnemodasusrectangulus* Gebien, 1914, by subsequent designation (Kaszab 1982b: 43). Status: junior synonym of *Brachyidium* Fairmaire, 1883 in Blaptinae: Opatrini: Ammobiina. Synonymy: Gebien (1921a: 492).

*Cnemodinus* Cockerell, 1906: 242 [M]. Type species [automatic]: *Cnemodustestaceus* Horn, 1870, by monotypy. Status: valid genus in Pimeliinae: Cnemodinini. Note: replacement name for *Cnemodus* Horn, 1870.

*Cnemodus* Horn, 1870: 266 [M]. Type species: *Cnemodustestaceus* Horn, 1870, by monotypy. Status: senior synonym of *Cnemodinus* Cockerell, 1906 in Pimeliinae: Cnemodinini. Note: junior homonym of *Cnemodus* Herrich-Schaeffer, 1850 [Hemiptera].

*Cnemophloeus* Bremer, 1998: 10, 13 [M]. Type species: *Hypophlaeusfilum* Fairmaire, 1893, by original designation. Status: junior synonym of *Stenophloeus* Blair, 1921 in Diaperinae: Hypophlaeini. Synonymy: Löbl et al. (2008b: 312).

*Cnemoplatia* Wollaston, 1865: 411 [F]. Type species [automatic]: *Cnemeplatiaatropos* Costa, 1847, by monotypy. Status: junior synonym of *Cnemeplatia* Costa, 1847 in Pimeliinae: Cnemeplatiini: Cnemeplatiina. Note: unjustified emendation of *Cnemeplatia* Costa, 1847, not in prevailing usage.

*Cneocnemis* Gebien, 1914c: 32 [F]. Type species: *Ulomahaemorrhoum* Fairmaire, 1893, by original designation. Status: valid genus in Tenebrioninae: Ulomini.

*Cnephalura* Doyen, 1988: 313 [F]. Type species: *Cnephaluraumbrata* Doyen, 1988, by original designation. Status: valid genus in Stenochiinae: Cnodalonini.

*Cnodalon* Latreille, 1797: 23 [N]. Type species: *Cnodalonviride* Latreille, 1804, by subsequent monotypy (Latreille 1804: 321). Status: valid genus in Stenochiinae: Cnodalonini. Note: originally proposed without included nominal species; in order to promote nomenclatural stability Bousquet and Bouchard (2019: 114, 116) recommended to the ICZN to use its plenary powers to designate *Cnodalonviride* Latreille, 1804 as the type species for the nominal genus *Cnodalon* Latreille, 1797, as currently accepted in the literature, after the recent discovery that this species was in fact not an originally included nominal species in the genus *Cnodalon*, and so not available as the type species.

*Cnodalum* Agassiz, 1846b: 91 [N]. Type species [automatic]: *Cnodalonviride* Latreille, 1804, by subsequent monotypy (Latreille 1804: 321). Status: junior synonym of *Cnodalon* Latreille, 1797 in Stenochiinae: Cnodalonini. Note: unjustified emendation of *Cnodalon* Latreille, 1797, not in prevailing usage; in a recent application to the ICZN to fix type species problems with the genus *Cnodalon* Latreille, 1797, Bousquet and Bouchard (2019: 116) recommended that *Cnodalum* Agassiz, 1846 be placed on the Official Index of Rejected and Invalid Generic Names in Zoology in order to avoid homonymy problems with the name *Cnodalum* Emeljanov, 1978 [Hemiptera], which is currently used as valid.

*Coccimarygmus* Ardoin, 1966: 185, 187 [M]. Type species: *Paramarygmusmorychoides* Fairmaire, 1894, by original designation. Status: valid genus and subgenus in Tenebrioninae: Amarygmini.

*Cochabambia* Marcuzzi, 1985: 184 [F]. Type species: *Cochabambiakulzeri* Marcuzzi, 1985, by monotypy. Status: senior synonym of *Allotriocochabambia* Faúndez, Rider & Carvajal, 2014 in Tenebrionidae: incertae sedis. Note: junior homonym of *Cochabambia* Pirán, 1959 [Hemiptera].

*Coeladesmia* Reitter, 1916a: 3 [F]. Type species: *Metriopusplatynotus* Gerstaecker, 1854, by subsequent designation (Gebien 1937a: 656). Status: valid subgenus of *Metriopus* Solier, 1835 in Pimeliinae: Adesmiini.

*Coelocnemis* Mannerheim, 1843: 280 [F]. Type species: *Coelocnemisdilaticollis* Mannerheim, 1843, by subsequent designation (R. Lucas 1920: 194). Status: valid genus in Stenochiinae: Cnodalonini.

*Coelocnemodes* Bates, 1879b: 474 [M]. Type species: *Coelocnemodesstoliczkanus* Bates, 1879, by monotypy. Status: valid genus in Blaptinae: Blaptini: Blaptina.

*Coeloecetes* Blair, 1929b: 384 [M]. Type species: *Coeloecetescavernicola* Blair, 1929, by monotypy. Status: valid genus in Blaptinae: Opatrini: Ammobiina.

*Coelolophus* Mäklin, 1867: 502 [M]. Type species: *Coelolophusbicarinatus* Mäklin, 1867, by subsequent designation (Löbl et al. 2008a: 41). Status: junior synonym of *Strongylium* W. Kirby, 1819 in Stenochiinae: Stenochiini. Synonymy: Gebien (1948: 519).

*Coelometopus* Solier, 1848: 154, 278 [M]. Type species: *Coelometopusclypeatus* Solier, 1848 (= *Blaps clypeata* Germar, 1813), by monotypy. Status: valid genus in Stenochiinae: Cnodalonini.

*Coelomorpha* Casey, 1890a: 182 [F]. Type species: *Coelomorphamaritima* Casey, 1890, by monotypy. Status: junior synonym of *Coelus* Eschscholtz, 1829 in Pimeliinae: Coniontini. Synonymy: Doyen (1972: 371).

*Coelopalorus* Blair, 1930: 135 [M]. Type species: *Palorusfoveicollis* Blair, 1930 (= *Ulominacarinata* Baudi di Selve, 1876), by monotypy. Status: junior synonym of *Ulomina* Baudi di Selve, 1876 in Tenebrioninae: Palorini. Synonymy: Scupola (2002: 186).

*Coelophanes* Iablokoff-Khnzorian, 1964: 309 [M]. Type species: *Hedyphanesimpressicollis* Faldermann, 1837 (= *Hedyphaneslaticollis* Fischer, 1832), by original designation. Status: junior synonym of *Hedyphanes* Fischer, 1820 in Tenebrioninae: Helopini: Helopina. Synonymy: Nabozhenko (2005: 351).

*Coelopleurum* Gebien, 1921b: 35 [N]. Type species: *Coelopleurumglabratum* Gebien, 1921, by monotypy. Status: valid genus in Diaperinae: Diaperini: Diaperina.

*Coelosattus* Blaisdell, 1927: 166 [M]. Type species: *Coelosattusfortineri* Blaisdell, 1927 (= *Eusattusdilatatus* J.L. LeConte, 1851), by monotypy. Status: junior synonym of *Eusattus* J.L. LeConte, 1851 in Pimeliinae: Coniontini. Synonymy: Doyen (1972: 373).

*Coelotaxis* Horn, 1876b: 200 [F]. Type species: *Coelotaxispunctulata* Horn, 1876, by subsequent designation (Gebien 1938a: 289). Status: junior synonym of *Coniontis* Eschscholtz, 1829 in Pimeliinae: Coniontini. Synonymy: Doyen (1972: 373).

*Coelus* Eschscholtz, 1829: 5 [M]. Type species: *Coelusciliatus* Eschscholtz, 1829, by monotypy. Status: valid genus in Pimeliinae: Coniontini.

*Colasia* Koch, 1965: 127, 131 [F]. Type species: *Colasiaakisoides* Koch, 1965, by monotypy. Status: valid genus in Blaptinae: Blaptini: Gnaptorinina.

*Collariheliofugus* Freude, 1960a: 125, 126 [M]. Type species: *Euschatiacollaris* Germain, 1855, by original designation. Status: valid subgenus of *Heliofugus* Guérin-Méneville, 1831 in Stenochiinae: Cnodalonini.

*Colophonesthes* Koch, 1954a: 48 [F]. Type species: *Colophonesthesmontisatri* Koch, 1954, by original designation. Status: valid genus in Blaptinae: Platynotini: Eurynotina.

*Colparthrum* Kirsch, 1866: 204 [N]. Type species: *Colparthrumgerstaeckeri* Kirsch, 1866, by monotypy. Status: valid genus and subgenus in Lagriinae: Lagriini: Statirina.

*Colpopatrum* Reitter, 1904: 148 [N]. Type species: *Opatrumasperipenne* Reitter, 1897, by monotypy. Status: valid subgenus of *Opatrum* Fabricius, 1775 in Blaptinae: Opatrini: Opatrina.

*Colpophorinus* Escalera, 1914: 336 [M]. Type species: *Opatrumgonocephaloides* Escalera, 1914, by monotypy. Status: junior synonym of *Colpophorus* Mulsant & Rey, 1859 in Blaptinae: Opatrini: Opatrina. Synonymy: Español (1954b: 317).

*Colpophorus* Mulsant & Rey, 1859c: 44 [M]. Type species: *Opatrumemarginatum* P.H. Lucas, 1847, by subsequent designation (Iwan and Löbl 2008: 269). Status: valid subgenus of *Opatrum* Fabricius, 1775 in Blaptinae: Opatrini: Opatrina.

*Colposcelis* Dejean, 1834: 185 [F]. Type species: *Tentyrialongicollis* Zubkov, 1833, by subsequent designation (Gebien 1937a: 598). Status: valid genus and subgenus in Pimeliinae: Tentyriini.

*Colposceloides* Schuster, 1940: 20 [M]. Type species: *Colposcelislicenti* Schuster, 1940, by monotypy. Status: valid subgenus of *Colposcelis* Dejean, 1834 in Pimeliinae: Tentyriini.

*Colposcythis* Reitter, 1889b: 113 [F]. Type species: *Colposcythiswalteri* Reitter, 1889, by monotypy. Status: valid subgenus of *Colposcelis* Dejean, 1834 in Pimeliinae: Tentyriini.

*Colposphena* Semenov, 1889: 217 [F]. Type species: *Sphenariabrevicollis* Solsky, 1882, by subsequent designation (Gebien 1937a: 577). Status: valid genus in Pimeliinae: Tentyriini.

*Colpotinoides* Kaszab, 1975b: 282, 354 [M]. Type species: *Pseudoblapsgebieni* Kaszab, 1942, by original designation. Status: valid genus in Blaptinae: Platynotini: Platynotina.

*Colpotinus* Fairmaire, 1891c: xvii [M]. Type species: *Colpotinussimulator* Fairmaire, 1891, by monotypy. Status: valid genus in Blaptinae: Pedinini: Pedinina.

*Colpotus* Mulsant & Rey, 1853b: 148, 208 [M]. Type species: *Colpotusstrigicollis* Mulsant & Rey, 1853, by subsequent designation (Gebien 1938a: 313). Status: valid subgenus of *Pedinus* Latreille, 1797 in Blaptinae: Pedinini: Pedinina.

*Comphosida* Macquart, 1850: 174 [F]. Type species: *Mesostenapunctipennis* Solier, 1835 (= *Pimeliaangustata* Fabricius, 1775), by monotypy. Status: junior synonym of *Mesostena* Eschscholtz, 1831 in Pimeliinae: Tentyriini. Synonymy: Lacordaire (1859a: 52).

*Compsocula* Fairmaire, 1898a: 236 [F]. Type species: *Stenogenaapicata* Fairmaire, 1896, by subsequent designation (R. Lucas 1920: 199). Status: valid genus in Alleculinae: incertae sedis. Note: placed in Alleculinae by Chatanay (1915a: 526).

*Compsomorphus* Solier, 1851: 208 [M]. Type species: *Compsomorphuselegans* Solier, 1851, by original designation. Status: junior synonym of *Apocrypha* Eschscholtz, 1831 in Tenebrioninae: Apocryphini. Synonymy: Lacordaire (1859b: 433).

*Conibiosoma* Casey, 1890b: 476 [N]. Type species: *Conibiuselongatus* Horn, 1870, by monotypy. Status: valid genus in Blaptinae: Opatrini: Blapstinina.

*Conibius* J.L. LeConte, 1851: 145 [M]. Type species: *Conibiusseriatus* J.L. LeConte, 1851, by subsequent designation (R. Lucas 1920: 199). Status: valid genus in Blaptinae: Opatrini: Blapstinina.

*Coniontellus* Casey, 1890b: 388 [M]. Type species: *Coniontisobesa* J.L. LeConte, 1851, by subsequent designation (Casey 1908: 57). Status: junior synonym of *Coniontis* Eschscholtz, 1829 in Pimeliinae: Coniontini. Synonymy: Doyen (1972: 373).

*Coniontides* Casey, 1908: 57, 78 [M]. Type species: *Coniontislata* J.L. LeConte, 1866, by original designation. Status: junior synonym of *Coniontis* Eschscholtz, 1829 in Pimeliinae: Coniontini. Synonymy: Doyen (1972: 373).

*Coniontis* Eschscholtz, 1829: 7 [F]. Type species: *Coniontisviatica* Eschscholtz, 1829, by subsequent designation (Casey 1908: 57). Status: valid genus in Pimeliinae: Coniontini.

*Conipinus* J.L. LeConte, 1862: 223 [M]. Type species: *Eusattusdubius* J.L. LeConte, 1851, by subsequent designation (Gebien 1938a: 284). Status: junior synonym of *Eusattus* J.L. LeConte, 1851 in Pimeliinae: Coniontini. Synonymy: J.L. LeConte (1866a: 60).

*Conisattus* Casey, 1895: 614 [M]. Type species: *Conisattusrectus* Casey, 1895, by monotypy. Status: valid genus in Pimeliinae: Coniontini.

*Conoecus* Horn, 1885: 159 [M]. Type species: *Conoecusovipennis* Horn, 1885, by monotypy. Status: valid genus in Pimeliinae: Epitragini.

*Conophthalmus* Quedenfeldt, 1885: 13 [M]. Type species: *Conophthalmussetulosus* Quedenfeldt, 1885, by monotypy. Status: valid subgenus of *Amatodes* Dejean, 1834 in Blaptinae: Pedinini: Helopinina. Note: see Robiche (2013: 522) for placement of the genus.

*Convexoodescelis* Egorov, 2004: 593 [F]. Type species: *Platyscelisbrevipennis* Kaszab, 1938, by original designation. Status: valid subgenus of *Oodescelis* Motschulsky, 1845 in Blaptinae: Platyscelidini.

*Cophodema* Gebien, 1943: 402 [F]. Type species [automatic]: *Cophosomahumeridens* Gebien, 1928, by monotypy. Status: valid genus in Stenochiinae: Cnodalonini. Note: replacement name for *Cophosoma* Gebien, 1928.

*Cophosoma* Gebien, 1928: 219, 222 [N]. Type species: *Cophosomahumeridens* Gebien, 1928, by monotypy. Status: senior synonym of *Cophodema* Gebien, 1943 in Stenochiinae: Cnodalonini. Note: junior homonym of *Cophosoma* Costa, 1849 [Hemiptera].

*Copistethus* Seidlitz, 1890: 524 [M]. Type species: *Cistelaspadix* Kiesenwetter, 1861, by monotypy. Status: valid genus in Alleculinae: Alleculini: Gonoderina.

*Coracostira* Fairmaire, 1899d: 220 [F]. Type species: *Coracostiraarmipes* Fairmaire, 1899, by **present designation**. Status: junior synonym of *Xenostethus* Bates, 1868 in Lagriinae: Lagriini: Statirina. Synonymy: Borchmann (1936: 460).

*Cordibates* Kulzer, 1956b: 909 [M]. Type species: *Cordibateschilensis* Kulzer, 1956, by original designation. Status: valid genus in Pimeliinae: Thinobatini.

*Corinta* Koch, 1950c: 36 [F]. Type species: *Corintalitoralis* Koch, 1950, by original designation. Status: valid genus in Blaptinae: Opatrini: Ammobiina.

*Coripera* Pascoe, 1866a: 483 [F]. Type species: *Adeliumdeplanatum* Boisduval, 1835, by original designation. Status: valid genus in Lagriinae: Adeliini.

*Cornopterus* Koch, 1950c: 40 [M]. Type species: *Cornopteruswykehami* Koch, 1950, by original designation. Status: valid genus in Blaptinae: Opatrini: Ammobiina.

*Cornucistela* Campbell, 1980: 133 [F]. Type species: *Cornucistelaserrata* Campbell, 1980, by original designation. Status: valid genus in Alleculinae: Alleculini: Gonoderina.

*Cornugeton* Bremer, 2016: 224 [M]. Type species: *Platolenesmonilicornis* Gebien, 1920, by original designation. Status: valid subgenus of *Amarygmus* Dalman, 1823 in Tenebrioninae: Amarygmini.

*Corticeus* Piller & Mitterpacher, 1783: 87 [M]. Type species: *Corticeusunicolor* Piller & Mitterpacher, 1783, by monotypy. Status: valid genus and subgenus in Diaperinae: Hypophlaeini.

*Coscinoptilix* Allard, 1876a: 15 [M]. Type species: *Coscinoptilixgracilicornis* Allard, 1876, by monotypy. Status: junior synonym of *Helops* Fabricius, 1775 in Tenebrioninae: Helopini: Helopina. Synonymy: Champion (1887: 312). Note: Allard’s original spelling *Coscinopter* is corrected to *Coscinoptilix* in the errata for volume 14 of ‘L’Abeille, Journal d’Entomologie’ (at the end of page 36 of the section “Table alphabétique”) and therefore *Coscinoptilix* is considered the correct original spelling (ICZN 1999, Article 32.5.1.1); see Bousquet et al. (2018: 183).

*Cosmogaster* Koch, 1956a: 164 [F]. Type species: *Anchophthalmusimpressicollis* Fairmaire, 1897, by original designation. Status: senior synonym of *Kochogaster* Kamiński & Raś, 2011 in Blaptinae: Platynotini: Platynotina. Note: junior homonym of *Cosmogaster* Faust, 1904 [Coleoptera: Curculionidae].

*Cosmonota* Blanchard, 1842: pl. 14 [F]. Type species: *Cosmonotaangustata* Blanchard, 1842, by subsequent designation (Gebien 1928: 127). Status: valid genus in Diaperinae: Diaperini: Diaperina.

*Cossyphodes* Westwood, 1851: 168 [M]. Type species: *Cossyphodeswollastonii* Westwood, 1851, by monotypy. Status: valid genus in Pimeliinae: Cossyphodini: Cossyphodina.

*Cossyphodinus* Wasmann, 1899a: 163 [M]. Type species: *Cossyphodinusindicus* Wasmann, 1899, by monotypy. Status: valid genus in Pimeliinae: Cossyphodini: Paramellonina.

*Cossyphodites* Brauns, 1901: 91 [M]. Type species: *Cossyphodeswoodrooffei* Péringuey, 1885, by monotypy. Status: valid genus in Pimeliinae: Cossyphodini: Cossyphoditina.

*Cossyphus* G.-A. Olivier, 1791: 121 [M]. Type species: *Lampyrisdepressa* Fabricius, 1781, by monotypy. Status: valid genus and subgenus in Lagriinae: Cossyphini.

*Costallecula* Pic, 1954: 235 [F]. Type species: *Costalleculaluteocostata* Pic, 1954, by monotypy. Status: valid genus in Alleculinae: incertae sedis.

*Costatosora* Pic, 1934: 32 [F]. Type species: *Nemostiradistincticornis* Pic, 1911, by monotypy. Status: valid genus in Lagriinae: Lagriini: Statirina.

*Costiferolagria* Pic, 1915b: 5 [F]. Type species: *Lagriasemialutacea* Pic, 1915, by monotypy. Status: valid genus in Lagriinae: Lagriini: Lagriina.

*Coxelinus* Fairmaire, 1869b: 202 [M]. Type species: *Coxelinusstricticollis* Fairmaire, 1869, by subsequent designation (Gebien 1941: 826). Status: valid genus in Lagriinae: Lupropini.

*Craniosphena* Koch, 1962a: 45, 54, 143 [F]. Type species: *Himatismusjusti* Fairmaire, 1880, by original designation. Status: valid genus in Pimeliinae: Tentyriini.

*Craniotus* J.L. LeConte, 1851: 142 [M]. Type species: *Craniotuspubescens* J.L. LeConte, 1851, by monotypy. Status: valid genus in Pimeliinae: Asidini.

*Cratidus* J.L. LeConte, 1862: 239 [M]. Type species: *Amphidoraosculans* J.L. LeConte, 1851, by monotypy. Status: valid subgenus of *Eleodes* Eschscholtz, 1829 in Blaptinae: Amphidorini.

*Cratopus* Eschscholtz, 1831: 5, 8 [M]. Type species: *Cratopuscastaneus* Eschscholtz, 1831, by monotypy. Status: senior synonym of *Scelosodis* Solier, 1835 in Pimeliinae: Tentyriini. Note: junior homonym of *Cratopus* Schönherr, 1826 [Coleoptera: Curculionidae].

†*Cretaceites* Wang, 1997: 203 [M]. Type species: *Cretaceitesjingxiensis* Wang, 1997, by original designation. Status: valid genus in Tenebrionidae: incertae sedis. Note: described from Lower Cretaceous deposits (China).

*Cribrasida* Reitter, 1917a: 11, 38 [F]. Type species: *Asidagrandipalpis* Allard, 1869, by subsequent designation (F. Soldati 2008: 32). Status: junior synonym of *Elongasida* Escalera, 1906 in Pimeliinae: Asidini. Synonymy: Viñolas and Cartagena (2005: 290).

*Crististibes* Koch, 1963: 62 [M]. Type species: *Planostibesbinodosus* Gebien, 1920, by monotypy. Status: valid genus in Blaptinae: Opatrini: Stizopodina.

*Crossoscelis* Gebien, 1914a: 52 [F]. Type species: *Crossoscelisclauda* Gebien, 1914, by monotypy. Status: junior synonym of *Strongylium* W. Kirby, 1819 in Stenochiinae: Stenochiini. Synonymy: Masumoto (1999a: 123).

*Cruracurvamtenebrio* Robiche, 2019b: 97 [M]. Type species: *Tenebriokamgangi* Robiche, 2019, by monotypy. Status: valid subgenus of *Tenebrio* Linnaeus, 1758 in Tenebrioninae: Tenebrionini.

*Cryphaeus* Klug, 1833: 89 [M]. Type species: *Cryphaeusaries* Klug, 1833, by monotypy. Status: valid genus in Tenebrioninae: Toxicini: Toxicina.

*Crypsinous* Fairmaire, 1891b: 262 [M]. Type species: *Crypsinousacutispina* Fairmaire, 1891, by monotypy. Status: valid genus in Tenebrioninae: Amarygmini.

*Crypsis* C.O. Waterhouse, 1877: 73 [F]. Type species: *Crypsisviolaceipennis* C.O. Waterhouse, 1877, by monotypy. Status: valid genus in Diaperinae: Leiochrinini.

*Cryptadius* Fairmaire, 1894f: 395 [M]. Type species [automatic]: *Crypsinousacutispina* Fairmaire, 1891, by monotypy. Status: junior synonym of *Crypsinous* Fairmaire, 1891 in Tenebrioninae: Amarygmini. Note: unnecessary replacement name for *Crypsinous* Fairmaire, 1891 (as “*Crypsinon*”); junior homonym of *Cryptadius* J.L. LeConte, 1851 [Coleoptera: Tenebrionidae: Pimeliinae: Edrotini].

*Cryptadius* J.L. LeConte, 1851: 140 [M]. Type species: *Cryptadiusinflatus* J.L. LeConte, 1851, by monotypy. Status: valid genus in Pimeliinae: Edrotini.

*Cryptasida* Koch, 1962a: 129 [F]. Type species: *Asidanamaqua* Péringuey, 1899, by original designation. Status: valid genus in Pimeliinae: Asidini.

*Crypticanus* Fairmaire, 1897f: 119 [M]. Type species: *Crypticanuscuneatus* Fairmaire, 1897 (= *Melanopterusedwarsii* Mulsant & Rey, 1854), by original designation. Status: valid genus in Blaptinae: Platynotini: Platynotina.

*Crypticocatops* Kaszab, 1975c: 102 [M]. Type species: *Platydemacatopoides* Fairmaire, 1896, by original designation. Status: valid subgenus of *Microcrypticus* Gebien, 1921 in Diaperinae: Crypticini.

*Crypticoides* Fairmaire, 1898d: 389 [M]. Type species: *Crypticoidesmellyi* Fairmaire, 1898, by monotypy. Status: junior synonym of *Oxycara* Solier, 1835 in Pimeliinae: Tentyriini. Synonymy: Koch (1960: 382).

*Crypticomorpha* Casey, 1908: 81, 140 [F]. Type species: *Coniontistenuis* Casey, 1908, by monotypy. Status: junior synonym of *Coniontis* Eschscholtz, 1829 in Pimeliinae: Coniontini. Synonymy: Aalbu et al. (2002: 487).

*Crypticopsis* Antoine, 1945: 270 [F]. Type species: *Crypticuscorticeus* Fairmaire, 1871, by original designation. Status: valid subgenus of *Crypticus* Latreille, 1816 in Diaperinae: Crypticini.

*Crypticus* Latreille, 1816: 298 [M]. Type species: *Helopsglaber* Fabricius, 1775 (= *Tenebrioquisquilius* Linnaeus, 1761), by monotypy. Status: valid genus and subgenus in Diaperinae: Crypticini.

*Cryptobates* Fairmaire, 1882a: 231 [M]. Type species: *Cryptobatesrubigineus* Fairmaire, 1882, by monotypy. Status: valid genus in Stenochiinae: Cnodalonini.

*Cryptobatoides* Kaszab, 1941a: 2, 15 [F]. Type species: *Cryptobatoidesopaca* Kaszab, 1941, by original designation. Status: valid genus in Stenochiinae: Cnodalonini.

*Cryptobrachys* Kaszab, 1941a: 4, 14 [M]. Type species: *Cryptobatescrassecostatus* Fairmaire, 1898, by monotypy. Status: valid genus in Stenochiinae: Cnodalonini.

*Cryptocarpes* Koch, 1952a: 191 [M]. Type species: *Cryptocarpeselongatus* Koch, 1952, by original designation. Status: valid subgenus of *Caenocrypticus* Gebien, 1920 in Pimeliinae: Caenocrypticini. Note: the First Reviser (*Cryptocarpes* Koch, 1952 versus *Lornamus* Koch, 1952) is Endrödy-Younga (1996: 21).

*Cryptochile* Latreille, 1828: 576 [F]. Type species: *Pimeliamaculata* Fabricius, 1781, by monotypy. Status: valid genus in Pimeliinae: Cryptochilini: Cryptochilina. Note: discovery of the older unused name *Phymatium* Billberg, 1820 threatens the validity of *Cryptochile* Latreille, 1828 and its associated family-group names currently used as valid; we recommend that an application be submitted to the International Commission on Zoological Nomenclature to conserve usage of *Cryptochile* Latreille, 1828.

*Cryptogenius* Solier, 1843: 37, 122 [M]. Type species: *Cryptogeniusdentatus* Solier, 1843, by original designation. Status: senior synonym of *Phrynocolus* Lacordaire, 1859 in Pimeliinae: Sepidiini: Molurina. Note: junior homonym of *Cryptogenius* Westwood, 1842 [Coleoptera: Hybosoridae].

*Cryptoglossa* Solier, 1837a: 638, 680 [F]. Type species: *Cryptoglossabicostata* Solier, 1837, by monotypy. Status: valid genus in Pimeliinae: Cryptoglossini.

†*Cryptohelops* Nabozhenko & Kirejtshuk, 2014: 68 [M]. Type species: *Cryptohelopsmenaticus* Nabozhenko & Kirejtshuk, 2014, by original designation. Status: valid genus in Tenebrioninae: Helopini: Helopina. Note: described from Middle-Upper Paleocene deposits (France).

*Cryptomysia* Pic, 1954: 260 [F]. Type species: *Cryptomysiaminor* Pic, 1954, by original designation. Status: valid genus in Alleculinae: Alleculini: Gonoderina.

*Cryptops* Solier, 1851: 235 [M]. Type species: *Cryptopsulomoides* Solier, 1851 (= *Tenebriodiaperinus* Panzer, 1797), by original designation. Status: junior synonym of *Alphitobius* Stephens, 1829 in Tenebrioninae: Alphitobiini. Synonymy: Gemminger in Gemminger and Harold (1870: 1962). Note: junior homonym of *Cryptops* Leach, 1814 [Chilopoda].

*Cryptostenophanes* Kaszab, 1941a: 5, 12 [M]. Type species: *Cryptostenophanesborneensis* Kaszab, 1941, by original designation. Status: valid genus in Stenochiinae: Cnodalonini.

*Cryptotrophus* Gistel, 1848a: xi [M]. Type species [automatic]: *Pimeliamaculata* Fabricius, 1781, by monotypy. Status: junior synonym of *Cryptochile* Latreille, 1828 in Pimeliinae: Cryptochilini: Cryptochilina. Note: unnecessary replacement name for *Cryptochile* Latreille, 1828.

*Cryptozoon* Schaufuss, 1882: 47 [N]. Type species: *Cryptozooncivile* Schaufuss, 1882, by subsequent designation (Bousquet et al. 2018: 284). Status: valid genus in Diaperinae: Gnathidiini: Anopidiina. Note: transferred from Colydiidae (see Doyen and Lawrence 1979: 366).

*Csikiola* Kaszab, 1955a: 493 [F]. Type species: *Csikiolathesileiformis* Kaszab, 1955, by original designation. Status: valid genus in Stenochiinae: Cnodalonini.

*Csiro* G.S. Medvedev & Lawrence, 1984: 563 [F]. Type species: *Hyocissubparallelus* Champion, 1894, by original designation. Status: valid genus and subgenus in Diaperinae: Hyociini: Hyociina.

*Cteisa* Solier, 1835a: 242 [F]. Type species: *Cteisahirta* Solier, 1835, by monotypy. Status: valid genus in Alleculinae: Alleculini: Xystropodina.

*Cteisodella* Novák, 2020f: 50 [F]. Type species: *Cteisodellaassamica* Novák, 2020, by original designation. Status: valid genus in Alleculinae: Alleculini: Alleculina.

*Cteisodes* Borchmann, 1932a: 307 [F]. Type species: *Cteisodessericea* Borchmann, 1932, by original designation. Status: valid genus in Alleculinae: Alleculini: Alleculina.

*Cteniopinus* Seidlitz, 1896: 200 [M]. Type species: *Cistelaaltaica* Gebler, 1830, by subsequent designation (R. Lucas 1920: 212). Status: valid genus and subgenus in Alleculinae: Cteniopodini.

*Ctenioposomus* Reitter, 1906b: 131 [M]. Type species: *Cteniopusfrater* Reitter, 1903, by subsequent designation (Novák and Pettersson 2008: 330). Status: valid subgenus of *Cteniopus* Solier, 1835 in Alleculinae: Cteniopodini. Note: the alternative original spelling *Ctenoposomus*, used by Reitter (1906b: 135), was rejected by Bousquet et al. (2015: 140) who acted as the First Revisers.

*Cteniopus* Solier, 1835: 245, 246 [M]. Type species: *Chrysomelasulphurea* Linnaeus, 1758, by subsequent designation (Westwood 1838: 32). Status: valid genus and subgenus in Alleculinae: Cteniopodini.

*Ctenogria* Borchmann, 1916a: 48, 101 [F]. Type species: *Ctenogriavermiculata* Borchmann, 1916, by original designation. Status: valid genus in Lagriinae: Lagriini: Lagriina.

*Ctesicles* Champion, 1896: 7 [M]. Type species: *Ctesiclesinsularis* Champion, 1896, by subsequent designation (R. Lucas 1920: 214). Status: junior synonym of *Diastolinus* Mulsant & Rey, 1859 in Blaptinae: Opatrini: Blapstinina. Synonymy: Ivie and Hart (2016: 468).

*Ctimene* Bates, 1873e: 359 [F]. Type species: *Ctimenebreweri* Bates, 1873, by monotypy. Status: senior synonym of *Mitrothorax* Carter, 1914 in Tenebrioninae: Heleini: Cyphaleina. Note: junior homonym of *Ctimene* Boisduval, 1832 [Lepidoptera].

*Cuemus* Bouchard, 2000: 96 [M]. Type species: *Cuemusmonteithi* Bouchard, 2000, by original designation. Status: valid genus in Stenochiinae: Cnodalonini.

*Cuphotes* Champion, 1887: 332 [F]. Type species [automatic]: *Spheniscuserotyloides* W. Kirby, 1819, by monotypy. Status: valid genus in Stenochiinae: Stenochiini. Note: replacement name for *Spheniscus* W. Kirby, 1819.

*Curimosphena* Gebien, 1920: 42, 43 [F]. Type species: *Himatismusvillosus* Haag-Rutenberg, 1870, by original designation. Status: junior synonym of *Imatismus* Dejean, 1834 in Pimeliinae: Tentyriini. Synonymy: Bouchard et al. (2007: 386).

*Curtolyprops* Pic, 1917a: 11 [M]. Type species: *Curtolypropslatipennis* Pic, 1917, by monotypy. Status: valid genus in Lagriinae: Lupropini.

*Curtopeltoides* Pic, 1916d: 14 [M]. Type species: *Curtopeltoidesrufescens* Pic, 1916, by subsequent designation (Gebien 1940: 1062). Status: valid genus in Tenebrioninae: Ulomini.

*Cybopiestes* Reitter, 1917b: 148, 150 [M]. Type species: *Cybopiestescsikii* Reitter, 1917, by monotypy. Status: valid genus in Stenochiinae: Cnodalonini.

*Cybostira* Borchmann, 1936: 429, 430 [F]. Type species: *Cybostiracaligata* Borchmann, 1936, by original designation. Status: valid subgenus of *Epicydes* Champion, 1889 in Lagriinae: Lagriini: Statirina.

*Cybotus* Casey, 1890b: 482 [M]. Type species: *Blapstinusestriatus* J.L. LeConte, 1878, by monotypy. Status: valid genus in Blaptinae: Opatrini: Blapstinina.

*Cychrachna* Koch, 1950b: 341, 361 [F]. Type species: *Cychrachnacarcharoides* Koch, 1950, by original designation. Status: valid genus in Pimeliinae: Tentyriini.

*Cychrochile* Koch, 1953b: 160 [F]. Type species: *Cychrochileerodioides* Koch, 1953, by original designation. Status: valid genus in Pimeliinae: Cryptochilini: Cryptochilina.

*Cyclobiomorphus* Pic, 1916c: 1 [M]. Type species: *Cyclobiomorphusundulatus* Pic, 1916, by subsequent designation (Gebien 1940: 420). Status: valid genus in Diaperinae: Diaperini: Diaperina.

*Cyclobium* Pic, 1916a: 11 [N]. Type species: *Cyclobiumvesiculiferum* Pic, 1916, by monotypy. Status: valid genus in Diaperinae: Diaperini: Diaperina.

*Cyclocnera* Leo, 2018: 50 [F]. Type species: *Cyclocneraazarovi* Leo, 2018, by original designation. Status: valid genus in Pimeliinae: Pimeliini.

*Cyclonesus* Fairmaire, 1896c: 104 [M]. Type species: *Cyclonesusparvicollis* Fairmaire, 1896, by monotypy. Status: valid genus in Stenochiinae: Cnodalonini.

*Cyclophanes* Carter, 1913a: 92 [M]. Type species: *Cyclophanesvariegatus* Carter, 1913, by subsequent designation (R. Lucas 1920: 217). Status: junior synonym of *Hemicyclus* Westwood, 1841 in Tenebrioninae: Heleini: Cyphaleina. Synonymy: Matthews (1992: 497).

*Cyclosattus* Casey, 1892: 710 [M]. Type species: *Eusattuswebsteri* Casey, 1891 (= *Celibecostata* Solier, 1848), by monotypy. Status: junior synonym of *Saragus* Erichson, 1842 in Tenebrioninae: Heleini: Heleina. Synonymy: Doyen et al. (1990: 244). Note: the type species was originally described from Colorado [United States of America] in error, Doyen et al. (1990: 244) pointed out that *Eusattuswebsteri* Casey, 1891 is a synonym of the Australian species *Celibecostata* Solier, 1848.

*Cylindrinotus* Faldermann, 1837: 73 [M]. Type species: *Cylindrinotuslugubris* Faldermann, 1837 (= *Helopsfemoratus* Faldermann, 1837), by subsequent designation (Gebien 1943: 425). Status: valid genus in Tenebrioninae: Helopini: Cylindrinotina. Note: Gebien (1943: 425) selected *Helopsfemoratus* Faldermann, 1837 as type species, which is not an originally included species in *Cylindrinotus*; however, since Gebien listed *Cylindrinotuslugubris* Faldermann, 1837 (a species originally included in *Cylindrinotus*) as a synonym of *Helopsfemoratus* Faldermann, 1837, he is deemed to have designated the latter as type species (ICZN 1999, Article 69.2.2); the original combination of the accepted name of the type species, *Helopsfemoratus* Faldermann, 1837, is a junior primary homonym of *Helopsfemoratus* Fabricius, 1798.

*Cylindronotus* Agassiz, 1846b: 111 [M]. Type species [automatic]: *Cylindrinotuslugubris* Faldermann, 1837 (= *Helopsfemoratus* Faldermann, 1837), by subsequent designation (Gebien 1943: 425). Status: junior synonym of *Cylindrinotus* Faldermann, 1837 in Tenebrioninae: Helopini: Cylindrinotina. Note: unjustified emendation of *Cylindrinotus* Faldermann, 1837, not in prevailing usage.

*Cylindrosia* Gebien, 1922b: 289 [F]. Type species: *Cylindrosiafoveifrons* Gebien, 1922, by monotypy. Status: valid genus in Tenebrioninae: Toxicini: Dysantina.

*Cylindrosora* Borchmann, 1936: 237, 375 [F]. Type species: *Soramacer* Borchmann, 1930, by original designation. Status: valid genus in Lagriinae: Lagriini: Statirina.

*Cylindrostira* Borchmann, 1936: 237, 374 [F]. Type species: *Casnonideacorporaali* Borchmann, 1929, by original designation. Status: valid genus in Lagriinae: Lagriini: Statirina.

*Cylindrothorus* Solier, 1843: 4, 50 [M]. Type species: *Cylindrothoruspilosus* Solier, 1843, by original designation. Status: valid genus and subgenus in Alleculinae: Alleculini: Mycetocharina.

*Cymatodes* Agassiz, 1846b: 112 [M]. Type species [automatic]: *Helopsundatus* Fabricius, 1792 (= *Erotylusnebulosus* Fabricius, 1781), by monotypy. Status: junior synonym of *Cymatothes* Dejean, 1834 in Tenebrioninae: Amarygmini. Note: unjustified emendation of *Cymatothes* Dejean, 1834, not in prevailing usage; the older name *Cymatodes* W. Kirby & Spence, 1828 [Coleoptera: Curculionidae] is not nomenclaturally available.

*Cymatothes* Dejean, 1834: 208 [M]. Type species: *Helopsundatus* Fabricius, 1792 (= *Erotylusnebulosus* Fabricius, 1781), by monotypy. Status: valid genus in Tenebrioninae: Amarygmini.

*Cymbeba* Pascoe, 1866a: 483 [F]. Type species: *Cymbebadissimilis* Pascoe, 1866, by monotypy. Status: valid genus in Lagriinae: Adeliini. Note: as mentioned by Matthews and Bouchard (2008: 350) the type species was erroneously described from Australia; the genus *Cymbeba* Pascoe, 1866 is endemic to New Caledonia.

*Cynaeus* J.L. LeConte, 1862: 233 [M]. Type species: *Platydemaangusta* J.L. LeConte, 1851, by original designation. Status: valid genus in Diaperinae: Diaperini: Adelinina.

*Cyphaleus* Westwood, 1841a: 43 [M]. Type species: *Helopsrugosus* Gray, 1831, by subsequent designation (Duponchel 1844a: 547). Status: valid genus in Tenebrioninae: Heleini: Cyphaleina. Note: nomen protectum (see Appendix 2); *Cyphaleus* is sometimes attributed to Hope (1841: 126) in the literature (e.g., Matthews 2019: 631); however, Hope did not describe the genus and there is no indication in his publication that “*Cyphaleusrugosus* Hope”; the only species he included in the genus is the same as *Helopsrugosus* Gray, 1831 (“*Cyphaleus* Hope” was treated as an unavailable name by Neave (1939: 939)); the First Reviser (*Cyphaleus* Westwood, 1841 versus *Chartopteryx* Westwood, 1841) is Matthews (1992: 490).

*Cyphelops* Fairmaire, 1901a: 73 [M]. Type species: *Cyphelopsinflatus* Fairmaire, 1901, by monotypy. Status: junior synonym of *Miotodera* Fairmaire, 1901 in Stenochiinae: Stenochiini. Synonymy: Chatanay (1915a: 526).

*Cyphogenia* Solier, 1837a: 638, 677 [F]. Type species: *Tenebrioauritus* Pallas, 1781, by monotypy. Status: valid genus and subgenus in Pimeliinae: Akidini.

*Cyphonotus* Guérin-Méneville, 1831a: pl. 5 [M]. Type species: *Cyphonotusdromedarius* Guérin-Méneville, 1831, by monotypy. Status: senior synonym of *Homocyrtus* Dejean, 1834 in Tenebrionidae: incertae sedis. Note: junior homonym of *Cyphonotus* Fischer, 1823 [Coleoptera: Scarabaeidae].

*Cyphostethe* Marseul, 1866: xxxix [F]. Type species: *Himatismusferrugineus* Marseul, 1866, by subsequent designation (Gebien 1937a: 573). Status: valid genus and subgenus in Pimeliinae: Tentyriini.

*Cyphostethoides* Löbl & Merkl in Löbl et al., 2020: 3 [M]. Type species: *Cyphostethebrunnea* Kaszab, 1962, by original designation. Status: valid subgenus of *Cyphostethe* Marseul, 1866 in Pimeliinae: Tentyriini. Note: name first proposed by Kaszab (1979c: 273) without fixation of a type species in the original publication (ICZN 1999, Article 13.3); Löbl and Merkl (2003: 244) designated *Cyphostethebrunnea* Kaszab, 1962 as the type species of Kaszab’s name but did not explicitly indicate the genus-group name as intentionally new (ICZN 1999, Article 16.1).

*Cyptus* Gerstaecker, 1871: 61 [M]. Type species: *Cyptusscabrosus* Gerstaecker, 1871, by monotypy. Status: valid genus in Blaptinae: Opatrini: Ammobiina.

*Cyriogeton* Pascoe, 1871: 356 [M]. Type species: *Cyriogetoninsignis* Pascoe, 1871, by monotypy. Status: valid subgenus of *Plesiophthalmus* Motschulsky, 1857 in Tenebrioninae: Amarygmini.

*Cyrta* Lacordaire, 1859: 724 [F]. Type species [automatic]: *Cirsastriaticollis* P.H. Lucas, 1857, by monotypy. Status: junior synonym of *Cirsa* P.H. Lucas, 1857 in Pimeliinae: Tentyriini. Note: unjustified emendation of *Cirsa* P.H. Lucas, 1857, not in prevailing usage.

*Cyrtoderes* Dejean, 1834: 181 [M]. Type species: *Sepidium lacunosum* Thunberg, 1784 (= *Tenebriocristatus* DeGeer, 1778), by subsequent designation (Bousquet and Bouchard 2013a: 45). Status: valid genus in Pimeliinae: Sepidiini: Trachynotina.

*Cyrtomius* Casey, 1907: 379 [M]. Type species: *Cyrtomiuscavicauda* Casey, 1907 (= *Epitragusplicatus* Champion, 1884), by original designation. Status: valid genus and subgenus in Pimeliinae: Epitragini.

*Cyrtosoma* Perty, 1830: 59 [N]. Type species: *Cyrtosomaunicolor* Perty, 1830, by monotypy. Status: valid genus in Stenochiinae: Cnodalonini.

*Cyrtostrongylium* Blair, 1929a: 246 [N]. Type species: *Cyrtostrongyliumrhysopaussoides* Blair, 1929 (= *Macrosynopticuscostatus* Pic, 1922), by monotypy. Status: junior synonym of *Macrosynopticus* Pic, 1922 in Tenebrioninae: Amarygmini. Synonymy: Pic (1930d: 12).

*Cyrtotyche* Pascoe, 1866a: 469 [F]. Type species: *Cyrtotychesatanas* Pascoe, 1866, by monotypy. Status: valid genus in Stenochiinae: Cnodalonini.

*Cyrtotyctus* Kolbe, 1897a: 241 [M]. Type species: *Cyrtotyctusosdaroides* Kolbe, 1897, by monotypy. Status: valid genus in Stenochiinae: Cnodalonini.

*Dactylocalcar* Gebien, 1938b: 46 [N]. Type species: *Dactylocalcarcaecus* Gebien, 1938, by monotypy. Status: valid subgenus of *Zophosis* Latreille, 1802 in Pimeliinae: Zophosini.

*Daedrosis* Bates, 1868: 266 [F]. Type species: *Daedrosiscrenatostriata* Bates, 1868, by subsequent designation (R. Lucas 1920: 224). Status: valid genus in Lagriinae: Adeliini.

*Dailognatha* Steven, 1828: 88 [F]. Type species: *Tentyriaaequalis* Tauscher, 1812, by monotypy. Status: valid genus in Pimeliinae: Tentyriini.

*Dalmanius* Bremer, 2001b: 90, 95 [M]. Type species: *Dalmaniusperegrinus* Bremer, 2001, by original designation. Status: valid genus in Tenebrioninae: Amarygmini.

*Damatris* Laporte, 1840: 224 [F]. Type species: *Tetraphyllusformosus* Laporte & Brullé, 1831, by monotypy. Status: valid genus in Stenochiinae: Cnodalonini.

*Danielomira* Weise, 1974: 119 [F]. Type species: *Isomiracantabrica* Weise, 1974, by original designation. Status: valid subgenus of *Isomira* Mulsant, 1856 in Alleculinae: Alleculini: Gonoderina.

*Danodema* Gebien, 1925c: 570 [F]. Type species: *Danodemasubcalva* Gebien, 1925, by monotypy. Status: valid genus in Stenochiinae: Cnodalonini.

*Daochus* Champion, 1886: 139 [M]. Type species: *Daochusmandibularis* Champion, 1886, by monotypy. Status: valid genus in Phrenapatinae: Penetini.

*Dasus* Motschulsky, 1845a: 78 [M]. Type species: *Opatrumfuscum* Herbst, 1793 (= *Opatrumrusticum* G.-A. Olivier, 1812), by original designation. Status: junior synonym of *Gonocephalum* Solier, 1834 in Blaptinae: Opatrini: Opatrina. Synonymy: Bedel (1894: 154).

*Dasyplonyx* Bremer, 2014a: 37 [M]. Type species: *Cyriogetonmaculosus* Pic, 1922, by original designation. Status: valid genus in Tenebrioninae: Amarygmini.

*Dasytoxystropus* Pic, 1921b: 12 [M]. Type species: *Dasytoxystropussubparallelus* Pic, 1921, by original designation. Status: valid genus in Alleculinae: Alleculini: Xystropodina.

*Dauresia* Ferrer, 2001: 187 [F]. Type species: *Dauresiamontisusti* Ferrer, 2001, by original designation. Status: valid genus in Stenochiinae: Stenochiini.

*Davaona* Borchmann, 1930a: 442, 524 [F]. Type species: *Casnonideaperforata* Borchmann, 1913, by original designation. Status: valid genus in Lagriinae: Lagriini: Statirina.

*Debeauxiella* Bouchard & Bousquet, **new subgenus** [F]. Type species: *Pachyceraangulophthalma* Koch, 1943, by **present designation**. Status: valid subgenus of *Hyperops* Eschscholtz, 1831 in Pimeliinae: Tentyriini. Note: Koch (1943a: 524, 546) introduced the new subgenus name *Debeauxiella* for three nominal species, but unfortunately did not designate a type species; the subgenusDebeauxiella, which has been treated as valid since 1943, is therefore unavailable (ICZN 1999, Article 13.3); we hereby make the name available by selecting *Pachyceraangulophthalma* Koch, 1943 as type species and referring to Koch (1943a: 524) for the character states that characterise and differentiate *Debeauxiella*.

*Dechius* Pascoe, 1866a: 455 [M]. Type species: *Dechiusaphodioides* Pascoe, 1866 (= *Tenebriocancellatus* Montrouzier, 1860), by monotypy. Status: junior synonym of *Scotoderus* Perroud & Montrouzier, 1865 in Stenochiinae: Cnodalonini. Synonymy: Bates (1873f: 473).

*Dechiustes* Blair, 1940: 137 [M]. Type species: *Dechiustescarolinensis* Blair, 1940, by original designation. Status: valid genus in Stenochiinae: Cnodalonini.

*Decialma* Pascoe, 1869: 288, 291 [F]. Type species: *Decialmatenuitarsis* Pascoe, 1869, by monotypy. Status: junior synonym of *Olisthaena* Erichson, 1842 in Tenebrioninae: Heleini: Cyphaleina. Synonymy: Carter (1913a: 62).

*Decoriplus* Louw, 1979: 117, 120 [M]. Type species: *Psammodespictus* Haag-Rutenberg, 1871, by original designation. Status: valid genus in Pimeliinae: Sepidiini: Oxurina.

*Delognatha* Agassiz, 1846b: 116, 118 [F]. Type species [automatic]: *Tentyriaaequalis* Tauscher, 1812, by monotypy. Status: junior synonym of *Dailognatha* Steven, 1828 in Pimeliinae: Tentyriini. Note: unjustified emendation of *Dailognatha* Steven, 1828, not in prevailing usage; suppressed for the purposes of both the Principle of Priority and the Principle of Homonymy and placed on the Official Index of Rejected and Invalid Generic Names in Zoology by the ICZN (2010b, Opinion 2250).

*Delognatha* Lacordaire, 1859a: 315 [F]. Type species: *Delognathalacordairei* Lacordaire, 1859, by subsequent designation (Gebien 1940: 756). Status: valid genus in Phrenapatinae: Phrenapatini. Note: the senior homonym *Delognatha* Agassiz, 1846, an unjustified emendation of *Dailognatha* Steven, 1828 [Coleoptera: Tenebrionidae: Pimeliinae: Tentyriini], was placed on the Official Index of Rejected and Invalid Generic Names in Zoology and suppressed for the purposes of both the Principle of Priority and the Principle of Homonymy by the ICZN (2010b, Opinion 2250).

*Delonurops* Reitter, 1922a: 24, 25 [M]. Type species: *Entomogonusclavimanus* Reitter, 1903, by subsequent designation (Iablokoff-Khnzorian 1964: 304). Status: valid subgenus of *Entomogonus* Solier, 1848 in Tenebrioninae: Helopini: Helopina.

*Delopygus* J.L. LeConte, 1866b: 129 [M]. Type species: *Delopyguscrenatus* J.L. LeConte, 1866, by monotypy. Status: junior synonym of *Eutochia* J.L. LeConte, 1862 in Tenebrioninae: Ulomini. Synonymy: Horn (1870: 372).

*Dema* Gistel, 1848a: xi [F]. Type species [automatic]: *Opatrumclathratum* Fabricius, 1787, by monotypy. Status: junior synonym of *Opatrinus* Dejean, 1821 in Blaptinae: Platynotini: Platynotina. Note: unnecessary replacement name for *Opatrinus*Dejean 1821.

*Demtrius* Broun, 1895: 243 [M]. Type species: *Demtriuscarinulatus* Broun, 1895, by monotypy. Status: valid genus in Tenebrioninae: Titaenini. Note: placed in Titaenini by Matthews (2003b: 13).

*Dendarophylan* Español, 1945: 312, 313 [M]. Type species: *Phylan pardoi* Español, 1945, by original designation. Status: valid genus in Blaptinae: Dendarini: Dendarina.

*Dendaroscelis* Reitter, 1904: 79 [F]. Type species: *Dendarusserripes* Reitter, 1904, by monotypy. Status: valid subgenus of *Dendarus* Dejean, 1821 in Blaptinae: Dendarini: Dendarina.

*Dendarus* Dejean, 1821: 65 [M]. Type species: *Helopstristis* Rossi, 1790, by subsequent designation (Blanchard 1844: pl. 48). Status: valid genus and subgenus in Blaptinae: Dendarini: Dendarina. Note: as pointed out by Silfverberg (1984: 59) and Löbl et al. (2008a: 41) the *Helopstristis* Herbst of Laporte (1840) (= *Pandaruscoarcticollis* Mulsant, 1854) was used as the type species of *Dendarus* (e.g., Gebien 1938a: 299) because *Helopstristis* Rossi, 1790 was treated as a nomen dubium; however, the *Helopstristis* Herbst of Laporte (1840), in addition to being nomenclaturally unavailable, was not originally included in *Dendarus* Dejean, 1821 and therefore cannot be used as the type species; the earliest, and valid, type species designation is that of Blanchard (1844: pl. 48, as “Pedinus (Dendarus) tristis Rossi”).

*Dendroscopius* Gistel, 1848a: 125 [M]. Type species [automatic]: *Trogossitathoracica* Fabricius, 1792, by monotypy. Status: junior synonym of *Bius* Dejean, 1834 in Tenebrioninae: Tenebrionini. Note: unnecessary replacement name for *Bius* Dejean, 1834.

*Dengitha* Reitter, 1887b: 516 [F]. Type species: *Dengithalutea* Reitter, 1887, by monotypy. Status: valid genus in Pimeliinae: Tentyriini.

*Dentatoploedipus* Kaszab, 1984: 355, 383 [M]. Type species: *Dentatoploedipussembilanicus* Kaszab, 1984, by original designation. Status: valid genus in Stenochiinae: Cnodalonini.

*Dentibirus* G.S. Medvedev, 1968a: 170, 204 [M]. Type species: *Heliopatespusillus* Ménétriés, 1849, by original designation. Status: junior synonym of *Cabirutus* Strand, 1929 in Blaptinae: Pedinini: Pedinina. Synonymy: Kamiński and Iwan (2017: 595).

*Deplanchesia* Fauvel, 1860: 310 [F]. Type species: *Deplanchesiametallescens* Fauvel, 1860, by monotypy. Status: valid genus in Stenochiinae: Cnodalonini.

*Deretus* Gahan, 1900: 10 [M]. Type species: *Deretusdenticollis* Gahan, 1900, by monotypy. Status: valid genus in Tenebrioninae: Helopini: Helopina.

*Deriles* Motschulsky, 1872: 24 [M]. Type species: *Upisexcavata* Herbst, 1797, by original designation. Status: valid subgenus of *Amenophis* J. Thomson, 1858 in Stenochiinae: Cnodalonini. Note: we act as First Revisers and reject the alternative original spelling *Derilis*, used by Motschulsky (1872: 27).

*Derispia* Lewis, 1894: 389 [F]. Type species: *Diaperismaculipennis* Marseul, 1876, by original designation. Status: valid genus in Diaperinae: Leiochrinini.

*Derispiella* Kaszab, 1961a: 357, 364 [F]. Type species: *Derispiellahingstoni* Kaszab, 1961, by original designation. Status: valid genus in Diaperinae: Leiochrinini.

*Derispiola* Kaszab, 1946a: 30, 115 [F]. Type species: *Derispiolafruhstorferi* Kaszab, 1946, by original designation. Status: valid genus in Diaperinae: Leiochrinini. Note: *Derispiola* was used earlier by Gebien (1940: 434) but is unavailable from that date because it was published without a description, a definition, or a bibliographic reference to such a published statement (ICZN 1999, Article 13.1).

*Derispiolina* Kaszab, 1979b: 279 [F]. Type species: *Derispiolinapterolomoides* Kaszab, 1979, by original designation. Status: junior synonym of *Falsotithassa* Pic, 1934 in Lagriinae: Lupropini. Synonymy: Schawaller (2000: 2).

*Derolagria* Borchmann, 1916a: 61 [F]. Type species: *Lagriaplicatula* Borchmann, 1909, by subsequent designation (Borchmann 1936: 151). Status: valid genus in Lagriinae: Lagriini: Lagriina.

*Deroplatus* Solier, 1851: 133 [M]. Type species: *Geoboruscostatus* Blanchard, 1842, by subsequent designation (Gebien 1937a: 563). Status: junior synonym of *Geoborus* Blanchard, 1842 in Pimeliinae: Epitragini. Synonymy: Lacordaire (1859a: 77).

*Derosalax* Gebien, 1926: 85 [M]. Type species: *Derosalaxbruchi* Gebien, 1926, by monotypy. Status: valid genus in Pimeliinae: Trilobocarini.

*Derosimus* Fairmaire, 1904a: 62 [M]. Type species: *Derosimusquadricollis* Fairmaire, 1904, by monotypy. Status: junior synonym of *Rhypasma* Pascoe, 1862 in Lagriinae: Belopini. Synonymy: Blair (1935a: 104).

*Derosphaerius* Westwood, 1881: 362 [M]. Type species: *Derosphaeriusanthracinus* Westwood, 1881, by monotypy. Status: valid genus and subgenus in Pimeliinae: Tentyriini.

*Derosphaerus* J. Thomson, 1858: 99 [M]. Type species: *Derosphaerusglobicollis* J. Thomson, 1858, by subsequent designation (R. Lucas 1920: 233). Status: valid genus in Stenochiinae: Cnodalonini. Note: nomen protectum (see Bouchard and Bousquet 2020b: 6).

*Derostethe* Koch, 1950b: 285 [F]. Type species: *Himatismuspunctatissimus* Haag-Rutenberg, 1870, by original designation. Status: valid subgenus of *Cyphostethe* Marseul, 1866 in Pimeliinae: Tentyriini.

*Derostira* Fairmaire, 1897b: 388 [F]. Type species: *Derostiracrenulipennis* Fairmaire, 1897, by monotypy. Status: valid genus in Lagriinae: Lagriini: Statirina.

*Derostirostaius* Borchmann, 1936: 477 [M]. Type species: *Derostiralaticollis* Pic, 1913, by original designation. Status: valid subgenus of *Staius* Fairmaire, 1896 in Lagriinae: Lagriini: Statirina. Note: first proposed by Pic (1934: 32) without original type species designation; it is therefore unavailable from that date (ICZN 1999, Article 13.3).

*Derostrophus* Fairmaire, 1888a: 186 [M]. Type species [automatic]: *Derosphaeriusanthracinus* Westwood, 1881, by monotypy. Status: junior synonym of *Derosphaerius* Westwood, 1881 in Pimeliinae: Tentyriini. Note: unnecessary replacement name for *Derosphaerius* Westwood, 1881.

*Desertosochrus* Koch, 1958: 149 [M]. Type species: *Drosochruspiligaster* Koch, 1958, by original designation. Status: valid subgenus of *Drosochrus* Erichson, 1843 in Blaptinae: Pedinini: Helopinina.

*Diabolicobates* Pic, 1930b: 30 [M]. Type species: *Diabolicobatescornutus* Pic, 1930 (= *Mechanetesplatitubera* Kaszab, 1941), by monotypy. Status: junior synonym of *Mechanetes* C.O. Waterhouse, 1887 in Stenochiinae: Cnodalonini. Synonymy: Kaszab (1941a: 43).

*Diachoriops* Ando, 2020: 8 [M]. Type species [automatic]: *Schizommacucumericola* Gebien, 1921, by original designation. Status: valid genus in Stenochiinae: Cnodalonini. Note: replacement name for *Schizomma* Gebien, 1921.

*Diacis* Koch, 1955a: 105 [M]. Type species: *Trachynotusregalis* Haag-Rutenberg, 1876, by original designation. Status: valid subgenus of *Somaticus* Hope, 1841 in Pimeliinae: Sepidiini: Trachynotina.

*Diaclina* Jacquelin du Val, 1861: 296 [F]. Type species: *Tenebriochrysomelinus* Herbst, 1799 (= *Tenebriotestudineus* Piller & Mitterpacher, 1783), by original designation. Status: valid genus in Tenebrioninae: Alphitobiini.

*Diaderma* Koch, 1960: 399, 405 [N]. Type species: *Diadermapunticum* Koch, 1960, by original designation. Status: valid genus in Blaptinae: Opatrini: Ammobiina.

*Diaperis* Geoffroy, 1762: 337 [F]. Type species: *Chrysomelaboleti* Linnaeus, 1758, by subsequent monotypy (Müller 1776: 74; see ICZN 1994a, Opinion 1754). Status: valid genus in Diaperinae: Diaperini: Diaperina. Note: originally proposed without included nominal species; placed on the Official List of Generic names in Zoology by the ICZN (1994a, Opinion 1754).

*Diaphanidus* Reitter, 1900b: 299, 301 [M]. Type species: *Arthrodeisantennatus* Reitter, 1894, by subsequent designation (R. Lucas 1920: 235). Status: valid genus and subgenus in Pimeliinae: Erodiini.

*Diaspirus* Matthews, 1998: 704, 759 [M]. Type species: *Diaspirusbellendenus* Matthews, 1998, by original designation. Status: valid genus in Lagriinae: Adeliini.

*Diastanus* Fairmaire, 1902b: 338 [M]. Type species: *Diastanusnitidiventris* Fairmaire, 1902, by monotypy. Status: valid genus in Alleculinae: Cteniopodini.

*Diastixus* Allard, 1876a: 5 [M]. Type species: *Helopspunctipennis* P.H. Lucas, 1846 (= *Helopsheteromorphus* P.H. Lucas, 1846), by subsequent designation (Nabozhenko 2008: 38). Status: junior synonym of *Nesotes* Allard, 1876 in Tenebrioninae: Helopini: Helopina. Synonymy: Antoine (1949: 134).

*Diastoleus* Solier, 1838b: 8, 67 [M]. Type species: *Scotobiuscollaris* Guérin-Méneville, 1834, by original designation. Status: valid genus in Tenebrioninae: Scotobiini.

*Diastolinus* Mulsant & Rey, 1859a: 70, 74 [M]. Type species: *Blapsclathrata* Fabricius, 1792, by subsequent designation (R. Lucas 1920: 236). Status: valid genus in Blaptinae: Opatrini: Blapstinina. Note: the First Revisers (*Diastolinus* Mulsant & Rey, 1859 versus *Sellio* Mulsant & Rey, 1859) are Ivie and Hart (2016: 468).

*Diceroderes* Solier, 1841b: 30, 46 [M]. Type species: *Diceroderesmexicanus* Solier, 1841, by original designation. Status: valid genus in Tenebrioninae: Toxicini: Dysantina.

*Dichastops* Gerstaecker, 1871: 63 [M]. Type species: *Dichastopssubaeneus* Gerstaecker, 1871, by monotypy. Status: valid genus in Lagriinae: Lupropini.

*Dichillesthes* Reitter, 1916d: 156 [F]. Type species: *Dichilluscordicollis* Reitter, 1886, by monotypy. Status: valid subgenus of *Dichillus* Jacquelin du Val, 1860 in Pimeliinae: Stenosini: Dichillina.

*Dichillinus* Reitter, 1916d: 156, 161 [M]. Type species: *Tageniapusilla* Ménétriés, 1849, by subsequent designation (G.S. Medvedev 1975: 599). Status: valid subgenus of *Dichillus* Jacquelin du Val, 1860 in Pimeliinae: Stenosini: Dichillina.

*Dichillocerus* Reitter, 1916d: 155, 167 [M]. Type species: *Hyperopspertusus* Kiesenwetter, 1861, by subsequent designation (G.S. Medvedev 1975: 603). Status: valid subgenus of *Dichillus* Jacquelin du Val, 1860 in Pimeliinae: Stenosini: Dichillina.

*Dichillodontus* Reitter, 1916d: 156, 166 [M]. Type species: *Dichillusadriani* Reitter, 1916, by monotypy. Status: valid subgenus of *Dichillus* Jacquelin du Val, 1860 in Pimeliinae: Stenosini: Dichillina.

*Dichillomessor* Reitter, 1916d: 155 [M]. Type species: *Dichillushimalayanus* Fairmaire, 1896, by subsequent designation (G.S. Medvedev 1975: 594). Status: valid subgenus of *Dichillus* Jacquelin du Val, 1860 in Pimeliinae: Stenosini: Dichillina.

*Dichillus* Jacquelin du Val, 1860: 253 [M]. Type species: *Tageniaminuta* Solier, 1838, by original designation. Status: valid genus and subgenus in Pimeliinae: Stenosini: Dichillina.

*Dichomma* Solier, 1835b: 253, 271 [N]. Type species: *Dichommamaillei* Solier, 1835 (= *Tentyriadardana* Steven, 1828), by subsequent designation (Jacquelin du Val 1860: 249). Status: valid genus in Pimeliinae: Tentyriini.

*Dichotymus* Fairmaire, 1891f: ccxcv [M]. Type species: *Dichotymusstriatipennis* Fairmaire, 1891, by monotypy. Status: valid genus in Tenebrioninae: Amarygmini.

*Dichromma* Mulsant & Rey, 1855: 94 [N]. Type species: *Pandarinusforaminosus* Mulsant & Rey, 1855, by monotypy. Status: junior synonym of *Paroderus* Mulsant & Rey, 1854 in Blaptinae: Dendarini: Dendarina. Synonymy: Reitter (1904: 89, through placement of the type species in *Paroderus* Mulsant & Rey, 1854).

*Dichtha* Haag-Rutenberg, 1871: 39 [F]. Type species: *Cryptogeniusinflatus* Gerstaecker, 1854, by original designation. Status: valid genus in Pimeliinae: Sepidiini: Molurina.

*Dicraeosis* Gebien, 1911a: 355 [F]. Type species [automatic]: *Dicraeusbacillus* Marseul, 1876, by monotypy. Status: junior synonym of *Stenochinus* Motschulsky, 1860 in Stenochiinae: Cnodalonini. Note: replacement name for *Dicraeus* Marseul, 1876.

*Dicraeus* Marseul, 1876: 103 [M]. Type species: *Dicraeusbacillus* Marseul, 1876, by monotypy. Status: junior synonym of *Stenochinus* Motschulsky, 1860 in Stenochiinae: Cnodalonini. Synonymy: Gebien (1925a: 79). Note: junior homonym of *Dicraeus* Loew, 1873 [Diptera].

*Dictysomorphus* Pic, 1921d: 24 [M]. Type species: *Dictysomorphusdentaticornis* Pic, 1921, by monotypy. Status: junior synonym of *Ceropria* Laporte & Brullé, 1831 in Diaperinae: Diaperini: Diaperina. Synonymy: Kaszab (1983a: 132).

*Dictysus* Rye, 1874: 281 [M]. Type species [automatic]: *Dietysusconfusus* Pascoe, 1866 (= *Cnodalonmetallicum* Perty, 1831), by monotypy. Status: junior synonym of *Amarygmus* Dalman, 1823 in Tenebrioninae: Amarygmini. Note: unjustified emendation for *Dietysus* Pascoe, 1866, not in prevailing usage.

*Dicyrtodes* Matthews, 1998: 706, 740 [M]. Type species: *Dicyrtodesarneius* Matthews, 1998, by original designation. Status: valid genus in Lagriinae: Adeliini.

*Dicyrtus* Duponchel, 1844a: 5 [M]. Type species: *Dicyrtusgibbosus* Duponchel, 1844, by monotypy. Status: valid genus in Stenochiinae: Stenochiini. Note: combined description of a new genus and single new species (ICZN 1999, Article 12.2.6).

*Diemenoma* Matthews, 1998: 707, 710 [F]. Type species: *Adeliumcommodum* Pascoe, 1869, by original designation. Status: valid genus in Lagriinae: Adeliini.

*Diesia* Fischer, 1820: pl. 14 [F]. Type species: *Diesiaquadridentata* Fischer, 1820, by subsequent designation (Hope 1841: 118). Status: valid genus in Pimeliinae: Pimeliini.

*Diesiola* Skopin, 1961a: 397, 398 [F]. Type species: *Diesiaquadridentata* Fischer, 1820, by original designation. Status: junior synonym of *Diesia* Fischer, 1820 in Pimeliinae: Pimeliini. Synonymy: Skopin (1971: 328).

*Diestecopus* Solier, 1848: 152, 194 [M]. Type species: *Diestecopuserodioides* Solier, 1848, by original designation. Status: valid genus in Blaptinae: Pedinini: Helopinina.

*Diestesoma* Péringuey, 1904: 271 [N]. Type species: *Diestesomapulchrum* Péringuey, 1904, by monotypy. Status: valid genus in Stenochiinae: Stenochiini.

*Diestica* Pascoe, 1868: xii [F]. Type species: *Diesticaviridipennis* Pascoe, 1868, by monotypy. Status: junior synonym of *Poecilesthus* Dejean, 1834 in Stenochiinae: Stenochiini. Synonymy: Gebien (1911b: 589).

*Dietomorpha* Reymond, 1938: 143 [F]. Type species: *Dietomorphapardalis* Reymond, 1938, by monotypy. Status: valid genus in Pimeliinae: Pimeliini.

*Dietopsis* Solier, 1835a: 236 [F]. Type species: *Alleculasolieri* Laporte, 1840, by subsequent monotypy (Laporte 1840: 243). Status: valid subgenus of *Allecula* Fabricius, 1801 in Alleculinae: Alleculini: Alleculina. Note: originally proposed without included nominal species; Laporte (1840: 243), by describing one new species *Alleculasolieri* Laporte, 1840 in association with the subgenus “*Dietopsis* Sol.”, was the first author to subsequently and expressly include nominal species in *Dietopsis* (ICZN 1999, Article 67.2.2).

*Dietysus* Pascoe, 1866a: 486 [M]. Type species: *Dietysusconfusus* Pascoe, 1866 (= *Cnodalonmetallicum* Perty, 1831), by monotypy. Status: junior synonym of *Amarygmus* Dalman, 1823 in Tenebrioninae: Amarygmini. Synonymy: Gebien (1921a: 409).

*Dignamptus* J.L. LeConte, 1878: 421 [M]. Type species: *Dignamptusstenochinus* J.L. LeConte, 1878, by subsequent designation (Bousquet et al. 2018: 325). Status: junior synonym of *Talanus* Jacquelin du Val, 1857 in Stenochiinae: Talanini. Synonymy: Champion (1887: 321).

*Dignathosis* Koch, 1958: 75 [F]. Type species: *Dignathosisarcana* Koch, 1958, by original designation. Status: valid subgenus of *Zophosis* Latreille, 1802 in Pimeliinae: Zophosini.

*Dila* Fischer von Waldheim, 1844: 111 [F]. Type species: *Blapslaevicollis* Gebler, 1841, by subsequent designation (Motschulsky 1860c: 530). Status: valid genus in Blaptinae: Blaptini: Blaptina.

*Dilablaps* Bogatchev, 1976: 98 [F]. Type species: *Dilablapsparadoxa* Bogatchev, 1976, by original designation. Status: valid genus in Blaptinae: Blaptini: Blaptina.

*Dilamus* Jacquelin du Val, 1861: 279 [M]. Type species: *Borosrufipes* P.H. Lucas, 1846, by original designation. Status: valid genus and subgenus in Blaptinae: Opatrini: Ammobiina.

*Dillacerus* Solier, 1835: 497 [M]. Type species: *Dillacerusduponti* Solier, 1835 (= *Dolichoderusacuminatus* Klug, 1833), by monotypy. Status: senior synonym of *Macellocerus* Solier, 1848 in Tenebrioninae: Toxicini: Nycteropina. Note: discovery of this forgotten name threatens the stability of the junior synonym *Macellocerus* Solier, 1848; although *Dillacerus* Solier, 1835 has not been used as valid in the literature after 1899, we could not find usage of *Macellocerus* Solier, 1848 in at least 25 works, published by at least ten authors in the immediately preceding 50 years, and therefore reversal of precedence cannot be used to treat *Dillacerus* Solier, 1835 as a nomen oblitum; we recommend that an application be submitted to the International Commission on Zoological Nomenclature to conserve usage of *Macellocerus* Solier, 1848, a genus that includes approximately 50 valid species.

*Dilopersina* Reitter, 1909a: 117 [F]. Type species: *Prosodesjakowlewi* Semenov, 1894, by original designation. Status: valid subgenus of *Prosodes* Eschscholtz, 1829 in Blaptinae: Blaptini: Prosodina.

*Dimeriseis* Solier, 1834: 530 [F]. Type species: *Erodiuslaevigatus* G.-A. Olivier, 1792, by subsequent designation (Löbl et al. 2008a: 41). Status: valid subgenus of *Erodius* Fabricius, 1775 in Pimeliinae: Erodiini.

*Dimoniacis* Koch, 1958: 44 [F]. Type species: *Dimoniacisjacksoni* Koch, 1958, by original designation. Status: valid genus in Pimeliinae: Sepidiini: Sepidiina.

*Dimorphochilus* Borchmann, 1908: 352 [M]. Type species: *Dimorphochilusapicalis* Borchmann, 1908, by subsequent designation (R. Lucas 1920: 240). Status: valid genus in Alleculinae: Alleculini: Alleculina.

*Dinax* Gistel, 1848a: xi [M]. Type species [automatic]: *Erotylusfasciatus* Fabricius, 1781, by subsequent designation (Hope 1841: 133). Status: junior synonym of *Poecilesthus* Dejean, 1834 in Stenochiinae: Stenochiini. Note: unnecessary replacement name for *Poecilesthus* Dejean, 1834.

*Dineria* Motschulsky, 1860c: 530 [F]. Type species: *Blapsconfusa* Ménétriés, 1832 (= *Blaps halophila* Fischer, 1820), by subsequent designation (G.S. Medvedev and Iwan 2006: 617). Status: valid subgenus of *Blaps* Fabricius, 1775 in Blaptinae: Blaptini: Blaptina.

*Dinomus* Brême, 1842a: 113 [M]. Type species: *Dinomusperforatus* Brême, 1842, by monotypy. Status: valid genus in Stenochiinae: Cnodalonini.

*Dinoria* Pascoe, 1869: 141 [F]. Type species: *Dinoriapicta* Pascoe, 1869, by monotypy. Status: junior synonym of *Brycopia* Pascoe, 1869 in Lagriinae: Adeliini. Synonymy: Carter (1920a: 238).

*Dinoscelis* Gerstaecker, 1854: 533 [F]. Type species: *Dinoscelispasserinii* Gerstaecker, 1854, by monotypy. Status: valid subgenus of *Pycnocerus* Westwood, 1841 in Lagriinae: Pycnocerini. Note: combined description of a new genus and single new species (ICZN 1999, Article 12.2.6).

*Diodontes* Solier, 1834: 508, 518 [M]. Type species: *Diodontesporcatus* Solier, 1834, by subsequent designation (Hope 1841: 114). Status: valid genus in Pimeliinae: Erodiini.

*Dioedus* J.L. LeConte, 1862: 238 [M]. Type species: *Dioeduspunctatus* J.L. LeConte, 1862, by monotypy. Status: valid genus in Phrenapatinae: Penetini.

*Diopethes* Pascoe, 1882: 32 [M]. Type species: *Diopethesarachnoides* Pascoe, 1882, by monotypy. Status: valid genus in Stenochiinae: Cnodalonini.

*Diopoenus* Champion, 1888: 445 [M]. Type species: *Diopoenuscompressicornis* Champion, 1888, by monotypy. Status: valid genus in Alleculinae: Alleculini: Alleculina.

*Diorhychina* Borchmann, 1936: 239, 472 [F]. Type species: *Diorhychinaperforata* Borchmann, 1936, by original designation. Status: valid genus in Lagriinae: Lagriini: Statirina.

*Dioscoridemus* Koch, 1970: 115 [M]. Type species: *Dioscoridemusvonhayekae* Koch, 1970, by original designation. Status: valid genus in Stenochiinae: Cnodalonini.

*Dioxycula* Fairmaire, 1896c: 115 [F]. Type species: *Dioxyculaaranea* Fairmaire, 1896, by monotypy. Status: valid genus in Alleculinae: incertae sedis.

*Diphyrrhynchus* Fairmaire, 1849: 445 [M]. Type species: *Diphyrrhynchuschalceus* Fairmaire, 1849, by monotypy. Status: valid genus in Blaptinae: Opatrini: Heterotarsina. Note: *Diphyrrhynchus* is an incorrect subsequent spelling of the original spelling *Diphyrhynchus*, first used by Gemminger in Gemminger and Harold (1870: 1955), and in prevailing usage; *Diphyrrhynchus* is deemed to be the correct original spelling (ICZN 1999, Article 33.3.1).

*Diplocyrtus* Quedenfeldt, 1887: 257 [M]. Type species: *Diplocyrtusfloccosus* Quedenfeldt, 1887, by monotypy. Status: valid genus in Tenebrioninae: Apocryphini.

*Diprosodes* Reitter, 1909a: 115 [M]. Type species: *Prosodesbiformis* Semenov, 1894, by subsequent designation (G.S. Medvedev 1997: 570). Status: valid subgenus of *Prosodes* Eschscholtz, 1829 in Blaptinae: Blaptini: Prosodina.

*Dirosis* Miller, 1858: 115 [F]. Type species: *Dirosisnervosa* Miller, 1858 (= *Erodiusservillei* Solier, 1834), by monotypy. Status: valid subgenus of *Erodius* Fabricius, 1775 in Pimeliinae: Erodiini.

*Dischidus* Kolbe, 1886: 297 [M]. Type species: *Helopssinuatus* Fabricius, 1801 (= *Tenebriolaevigatus* Fabricius, 1781), by **present designation**. Status: junior synonym of *Taraxides* C.O. Waterhouse, 1876 in Stenochiinae: Cnodalonini. Synonymy: Champion (1891: 637). Note: the original combination of the accepted name of the type species, *Tenebriolaevigatus* Fabricius, 1781, is a junior primary homonym of *Tenebriolaevigatus* Linnaeus, 1767.

*Dischizillus* Wasmann, 1902: 244 [M]. Type species [automatic]: *Schizillusrogersi* Wasmann, 1899, by monotypy. Status: junior synonym of *Pseudethas* Fairmaire, 1896 in Pimeliinae: Stenosini: Dichillina. Note: replacement name for *Schizillus* Wasmann, 1899.

*Discodemus* J.L. LeConte, 1862: 223 [M]. Type species: *Zophosisreticulata* Say, 1824, by monotypy. Status: junior synonym of *Eusattus* J.L. LeConte, 1851 in Pimeliinae: Coniontini. Synonymy: J.L. LeConte (1866a: 60).

*Discogenia* J.L. LeConte, 1866b: 117 [F]. Type species: *Eleodesscabricula* J.L. LeConte, 1858, by subsequent designation (Bousquet et al. 2018: 149). Status: valid subgenus of *Eleodes* Eschscholtz, 1829 in Blaptinae: Amphidorini.

*Discopleurus* Lacordaire, 1859a: 105 [M]. Type species [automatic]: *Pleurophorusquadricollis* Solier, 1851, by monotypy. Status: valid genus in Pimeliinae: Stenosini: Dichillina. Note: replacement name for *Pleurophorus* Solier, 1851.

*Discotus* Reitter, 1904: 161 [M]. Type species: *Opatrumdilectans* Faldermann, 1836, by subsequent designation (G.S. Medvedev 1990: 185). Status: valid subgenus of *Penthicus* Faldermann, 1836 in Blaptinae: Opatrini: Opatrina.

*Disema* Mäklin, 1875: 646 [F]. Type species: *Disemabimaculata* Mäklin, 1875, by subsequent designation (R. Lucas 1920: 244). Status: valid genus in Lagriinae: Lagriini: Statirina.

*Disemorpha* Pic, 1917f: 16 [F]. Type species: *Disemorphaciliata* Pic, 1917, by monotypy. Status: valid genus in Lagriinae: Lagriini: Statirina.

*Dissonomus* Jacquelin du Val, 1861: 280 [M]. Type species [automatic]: *Heliopatespicipes* Faldermann, 1837, by subsequent designation (Gebien 1938a: 397). Status: valid genus and subgenus in Tenebrioninae: Dissonomini. Note: replacement name for *Heterophylus* Mulsant & Rey, 1859.

*Distretus* Haag-Rutenberg, 1871: 42 [M]. Type species: *Molurisamplipennis* Fåhraeus, 1870, by subsequent designation (Rye 1873: 287). Status: valid genus and subgenus in Pimeliinae: Sepidiini: Molurina.

*Ditaphronotus* Casey, 1907: 289, 341 [M]. Type species: *Emmenastusfoveicollis* Champion, 1884, by original designation. Status: valid genus in Pimeliinae: Edrotini.

*Diversogria* Pic, 1954: 229 [F]. Type species: *Heterogrialepersonnei* Pic, 1954, by original designation. Status: junior synonym of *Xanthalia* Fairmaire, 1894 in Lagriinae: Lagriini: Statirina. Synonymy: Merkl (2004: 285).

*Dividiopsa* Koch, 1944a: 158 [F]. Type species: *Gnophotaangolensis* Harold, 1879, by monotypy. Status: valid genus in Pimeliinae: Tentyriini.

*Divieta* Reitter, 1914c: 390 [F]. Type species: *Vietacostata* Allard, 1874, by subsequent designation (Löbl et al. 2008a: 41). Status: junior synonym of *Vieta* Laporte, 1840 in Pimeliinae: Sepidiini: Sepidiina. Synonymy: Lesne (1922: 696).

*Doderoella* Schuster, 1926: 133 [F]. Type species: *Doderoellacyrenaica* Schuster, 1926, by monotypy. Status: junior synonym of *Pimelia* Fabricius, 1775 in Pimeliinae: Pimeliini. Synonymy: Kwieton (1982: 35).

*Dolamara* Reichardt, 1935: 251 [F]. Type species: *Melanimoncupreomicans* Reitter, 1915, by original designation. Status: valid genus in Tenebrioninae: Melanimonini.

*Dolichasida* Reitter, 1917a: 40, 58 [F]. Type species: *Asidamoraguezi* Schaufuss, 1879, by subsequent designation (Wilke 1922: 258). Status: junior synonym of *Asida* Latreille, 1802 in Pimeliinae: Asidini. Synonymy: Gebien (1937a: 733; with *Insulasida* Escalera, 1922, a junior synonym of *Asida* Latreille, 1802).

*Dolichoderus* Klug, 1833: 87 [M]. Type species: *Dolichoderusacuminatus* Klug, 1833, by monotypy. Status: senior synonym of *Macellocerus* Solier, 1848 in Tenebrioninae: Toxicini: Nycteropina. Note: junior homonym of *Dolichoderus* Lund, 1831 [Hymenoptera].

*Doliema* Pascoe, 1860a: 50 [N]. Type species: *Doliemaplatisoides* Pascoe, 1860, by monotypy. Status: junior synonym of *Adelina* Dejean, 1835 in Diaperinae: Diaperini: Adelinina. Synonymy: Fleutiaux and Sallé (1890: 428), Spilman (1973: 40).

*Doliodesmus* Spilman, 1967: 149 [M]. Type species: *Doliodesmuscharlesi* Spilman, 1967, by monotypy. Status: valid genus in Diaperinae: Diaperini: Adelinina.

*Doliopines* Horn, 1894: 427 [M]. Type species: *Doliopinescucujinus* Horn, 1894, by monotypy. Status: valid genus in Diaperinae: Diaperini: Adelinina.

*Dolphus* Blanchard, 1847: pl. 11 [M]. Type species: *Dolphusglobipennis* Blanchard, 1847, by monotypy. Status: valid genus in Tenebrioninae: Helopini: incertae sedis.

*Donaciolagria* Pic, 1914b: 14 [F]. Type species: *Donaciolagriaimpressipennis* Pic, 1914, by monotypy. Status: valid genus in Lagriinae: Lagriini: Statirina.

*Donisiellus* Bremer, 1992: 111, 113 [M]. Type species: *Donisiellusdecellei* Bremer, 1992, by original designation. Status: valid genus in Tenebrioninae: Ulomini.

*Doranalia* Novák, 2020c: 481 [F]. Type species: *Cistelarufipennis* Marseul, 1876, by original designation. Status: valid genus in Alleculinae: Alleculini: Alleculina.

*Dordanea* Reitter, 1887a: 357 [F]. Type species: *Dordaneaelegans* Reitter, 1887, by monotypy. Status: valid subgenus of *Microdera* Eschscholtz, 1831 in Pimeliinae: Tentyriini.

*Dorelogena* Péringuey, 1904: 280 [F]. Type species: *Dorelogenacastanea* Péringuey, 1904, by subsequent designation (R. Lucas 1920: 248). Status: valid genus in Stenochiinae: Stenochiini.

*Dorota* Novák, 2018c: 452 [F]. Type species: *Allecularufoposticalis* Pic, 1944, by original designation. Status: valid genus in Alleculina: Alleculini: Alleculina.

*Dorrigonum* Matthews, 1998: 708, 752 [N]. Type species: *Licinomaumbilicata* Carter, 1924, by original designation. Status: valid genus in Lagriinae: Adeliini.

*Doryagus* Pascoe, 1887: 12 [M]. Type species: *Doryagustalpa* Pascoe, 1887, by monotypy. Status: junior synonym of *Stizopus* Erichson, 1843 in Blaptinae: Opatrini: Stizopodina. Synonymy: Gebien (1920: 120).

*Doyenia* Matthews & Lawrence, 2005: 532 [F]. Type species: *Doyeniacrematogastri* Matthews & Lawrence, 2005, by original designation. Status: valid genus in Lagriinae: Belopini.

*Doyenus* Iwan, 1996: 385, 386 [M]. Type species: *Doyenusuncus* Iwan, 1996, by original designation. Status: valid genus in Blaptinae: Platynotini: Platynotina.

*Drepanomela* Borchmann, 1936: 119 [F]. Type species: *Lagriacribratula* Schaufuss, 1887, by original designation. Status: valid subgenus of *Cerogria* Borchmann, 1911 in Lagriinae: Lagriini: Lagriina.

*Drocleana* Bates, 1879a: 291 [F]. Type species: *Camariachalcoptera* Klug, 1833, by original designation. Status: valid genus in Stenochiinae: Cnodalonini.

*Drosochrus* Erichson, 1843: 243 [M]. Type species: *Drosochrusbrunnipes* Erichson, 1843, by subsequent designation (Gebien 1943: 910). Status: valid genus and subgenus in Blaptinae: Pedinini: Helopinina.

*Dryadigmus* Bremer, 2007: 26 [M]. Type species: *Amarygmuscechovskyi* Bremer, 2007, by original designation. Status: valid subgenus of *Amarygmus* Dalman, 1823 in Tenebrioninae: Amarygmini.

*Duocula* Novák, 2019f: 56 [F]. Type species: *Oraculaclara* Novák, 2019, by original designation. Status: valid subgenus of *Oracula* Novák, 2019 in Alleculinae: Alleculini: Alleculina.

*Durandius* Kaszab, 1970c: 115 [M]. Type species: *Durandiusardoini* Kaszab, 1970, by original designation. Status: valid genus in Pimeliinae: Cnemeplatiini: Rondoniellina.

*Durasida* Reitter, 1917a: 9, 14 [F]. Type species: *Tenebriosilphoides* Linnaeus, 1767, by subsequent designation (F. Soldati 2008: 32). Status: junior synonym of *Glabrasida* Escalera, 1910 in Pimeliinae: Asidini. Synonymy: Viñolas and Cartagena (2005: 282).

*Dymonus* Solier, 1843: 7, 121 [M]. Type species: *Sepidium vestitum* Guérin-Méneville, 1831, by original designation. Status: junior synonym of *Vieta* Laporte, 1840 in Pimeliinae: Sepidiini: Sepidiina. Synonymy: Lacordaire (1859a: 205).

*Dysantes* Pascoe, 1869: 31 [M]. Type species: *Dicerodereselongatus* Redtenbacher, 1868, by monotypy. Status: valid genus in Tenebrioninae: Toxicini: Dysantina. Note: this name is not a junior homonym of *Dysantes* Forster, 1869 [Hymenoptera], as previously reported in the literature, since the name in Coleoptera was published 1 January 1869 whereas the name in Hymenoptera was issued in May 1869 (see Bouchard and Bousquet 2020a: 101 for additional comments).

*Dysarchus* Pascoe, 1866a: 449 [M]. Type species: *Dysarchusodewahnii* Pascoe, 1866, by monotypy. Status: valid genus in Tenebrioninae: Heleini: Heleina.

*Dysceladus* C.O. Waterhouse, 1875: 411 [M]. Type species: *Dysceladustuberculatus* C.O. Waterhouse, 1875 (= *Polposipusherculeanus* Solier, 1848), by monotypy. Status: junior synonym of *Polposipus* Solier, 1848 in Stenochiinae: Cnodalonini. Synonymy: Gebien (1922b: 268, 311).

*Dysgena* Mäklin, 1863b: 558 [F]. Type species: *Dysgenalugubris* Mäklin, 1863, by subsequent designation (R. Lucas 1920: 252). Status: valid genus in Tenebrioninae: Praeugenini.

*Dysodera* Borchmann, 1936: 238, 470 [F]. Type species: *Dysoderamethneri* Borchmann, 1936, by original designation. Status: valid genus in Lagriinae: Lagriini: Statirina.

*Dysopinus* Borchmann, 1936: 236, 379 [M]. Type species: *Nemostiraserra* Borchmann, 1913, by original designation. Status: valid genus in Lagriinae: Lagriini: Statirina.

*Dystalica* Pascoe, 1869: 142 [F]. Type species: *Dystalicahomogenea* Pascoe, 1869, by monotypy. Status: junior synonym of *Adelium* W. Kirby, 1819 in Lagriinae: Adeliini. Synonymy: Matthews (1998: 777).

*Dzhungaropterocoma* Skopin, 1974b: 150 [F]. Type species: *Pterocomasubnuda* Reitter, 1897, by original designation. Status: valid subgenus of *Pterocoma* Dejean, 1834 in Pimeliinae: Pimeliini.

*Earophanta* Semenov, 1903b: 172 [F]. Type species [automatic]: *Platyopeserrata* Semenov, 1893, by original designation. Status: valid genus in Pimeliinae: Pimeliini. Note: replacement name for *Earophila* Semenov, 1903.

*Earophila* Semenov, 1903a: 9 [F]. Type species: *Platyopeserrata* Semenov, 1893, by original designation. Status: senior synonym of *Earophanta* Semenov, 1903 in Pimeliinae: Pimeliini. Note: junior homonym of *Earophila* Gumppenberg, 1887 [Lepidoptera].

*Earophilina* Strand, 1917: 99 [F]. Type species [automatic]: *Platyopeserrata* Semenov, 1893, by original designation. Status: junior synonym of *Earophanta* Semenov, 1903 in Pimeliinae: Pimeliini. Note: unnecessary replacement name for *Earophila* Semenov, 1903.

*Eba* Pascoe, 1863b: 122, 129 [F]. Type species: *Ebacerylonoides* Pascoe, 1863, by monotypy. Status: junior synonym of *Palorus* Mulsant, 1854 in Tenebrioninae: Palorini. Synonymy: Carter and Zeck (1937: 194). Note: transferred from “Tenebrionoidea: Colydiidae” by Carter and Zeck (1937: 194).

*Ebenolus* Fairmaire, 1897c: 227 [M]. Type species: *Ebenolusvernicatus* Fairmaire, 1897, by monotypy. Status: junior synonym of *Strongylium* W. Kirby, 1819 in Stenochiinae: Stenochiini. Synonymy: Kaszab (1977b: 29).

*Ebertius* Jedlička, 1965: 98 [M]. Type species: *Ebertiusnepalensis* Jedlička, 1965 (= *Laenalongipilis* Schuster, 1926), by original designation. Status: junior synonym of *Laena* Dejean, 1821 in Lagriinae: Laenini. Synonymy: Kaszab (1970d: 429, through placement of the type species in *Laena* Dejean, 1821). Note: originally described in the family Carabidae.

*Eccoptostira* Borchmann, 1936: 236, 377 [F]. Type species: *Nemostirarohdei* Pic, 1912, by original designation. Status: valid genus in Lagriinae: Lagriini: Statirina. Note: we act as First Revisers and reject the alternative original spelling *Ecoptostira*, used by Borchmann (1936: 236).

*Eccoptostoma* Gebien, 1913: 70 [N]. Type species: *Taraxidesruficrus* Fairmaire, 1894, by **present designation**. Status: valid genus in Stenochiinae: Cnodalonini. Note: redescribed as new by Gebien (1921b: 62, 82).

*Echinotrigon* Skopin, 1973: 170 [M]. Type species: *Trigonoscelisgranulata* Reitter, 1915, by original designation. Status: valid subgenus of *Trigonoscelis* Dejean, 1834 in Pimeliinae: Pimeliini.

*Echinotus* Solier, 1843: 30 [M]. Type species: *Sepidium spinicolle* Laporte, 1840, by original designation [p. 122]. Status: valid genus in Pimeliinae: Sepidiini: Sepidiina.

*Echocerus* Horn, 1870: 364, 366 [M]. Type species: *Trogossitamaxillosa* Fabricius, 1801, by monotypy. Status: valid subgenus of *Gnatocerus* Thunberg, 1814 in Diaperinae: Diaperini: Adelinina.

*Ecnocera* Borchmann, 1936: 22, 220 [F]. Type species: *Porrolagriagracilis* Borchmann, 1909, by original designation. Status: valid genus in Lagriinae: Lagriini: Lagriina.

*Ecnolagria* Borchmann, 1916a: 49, 139 [F]. Type species: *Lagriagrandis* Gyllenhal, 1817, by original designation. Status: valid genus and subgenus in Lagriinae: Lagriini: Lagriina.

*Ecnomoderes* Gebien, 1928: 109, 110 [M]. Type species: *Ecnomoderesbarbatus* Gebien, 1928, by subsequent designation (Gebien 1937a: 690). Status: valid genus in Pimeliinae: Stenosini: Stenosina.

*Ecnomosternum* Gebien, 1928: 104 [N]. Type species: *Ecnomosternumvermiculatum* Gebien, 1928, by monotypy. Status: valid genus in Pimeliinae: Epitragini.

*Ecphoroma* Solier, 1836: 195 [N]. Type species: *Pimeliahemisphaerica* Solier, 1836, by subsequent designation (Löbl et al. 2008b: 157). Status: valid subgenus of *Pimelia* Fabricius, 1775 in Pimeliinae: Pimeliini.

*Ecripsis* Pascoe, 1866a: 456 [F]. Type species: *Ecripsispubescens* Pascoe, 1866, by monotypy. Status: junior synonym of *Ammidium* Erichson, 1843 in Blaptinae: Opatrini: Ammobiina. Synonymy: Blair in Carter (1914c: 406). Note: this African genus was originally described from Australia in error (see Matthews and Bouchard 2008: 351).

*Ectateus* Koch, 1956a: 230 [M]. Type species: *Anchophthalmusmodestus* Fairmaire, 1887, by original designation. Status: valid genus in Blaptinae: Platynotini: Platynotina.

*Ectatocera* Fåhraeus, 1870: 325 [F]. Type species: *Ectatoceralongicornis* Fåhraeus, 1870, by monotypy. Status: valid genus in Alleculinae: incertae sedis.

*Ectatocnemis* Horn, 1867: 400 [F]. Type species: *Ectatocnemismultilineata* Horn, 1867, by monotypy. Status: junior synonym of *Anomalipus* Guérin-Méneville, 1831 in Blaptinae: Platynotini: Platynotina. Synonymy: Endrödy-Younga (1988: 19). Note: this taxon was originally described from Chile in error, it is native to South Africa (see Endrödy-Younga 1988: 19).

*Ectenostoma* Fåhraeus, 1870: 317 [N]. Type species: *Ectenostomanigriventris* Fåhraeus, 1870, by monotypy. Status: valid genus in Alleculinae: incertae sedis.

*Ectomopsis* Fairmaire, 1905: 299 [F]. Type species: *Ectomopsisbruchi* Fairmaire, 1905, by monotypy. Status: valid genus in Stenochiinae: Cnodalonini.

*Ectromopsis* Antoine, 1949: 125, 145 [F]. Type species: *Catomuspoliticollis* Allard, 1876, by original designation. Status: valid genus in Tenebrioninae: Helopini: Cylindrinotina.

*Ectyche* Pascoe, 1869: 143 [F]. Type species: *Ectycheerebea* Pascoe, 1869, by monotypy. Status: valid genus in Diaperinae: Ectychini. Note: “*Ectyche? nana* Pascoe, 1869” was doubtfully included in this genus by Pascoe (1869: 143) and is therefore not an originally included species (ICZN 1999: Article 67.2.5).

*Edalus* Broun, 1893b: 1159 [M]. Type species: *Edalusopacus* Broun, 1893, by subsequent designation (R. Lucas 1920: 255). Status: senior synonym of *Wattadelium* Emberson, 2000 in Lagriinae: Adeliini. Note: junior homonym of *Edalus* Broun, 1886 [Coleoptera: Zopheridae].

*Edrotes* J.L. LeConte, 1851: 140 [M]. Type species: *Edrotesventricosus* J.L. LeConte, 1851, by monotypy. Status: valid genus and subgenus in Pimeliinae: Edrotini.

*Edrotinus* Fairmaire, 1904b: 461 [M]. Type species: *Edrotinustucumanus* Fairmaire, 1904, by original designation. Status: junior synonym of *Trilobocara* Solier, 1851 in Pimeliinae: Trilobocarini. Synonymy: Gebien (1937a: 592).

*Edrotopus* Haag-Rutenberg, 1877: 129 [M]. Type species: *Edrotopusstrigicollis* Haag-Rutenberg, 1877, by monotypy. Status: junior synonym of *Platyholmus* Dejean, 1834 genus in Pimeliinae: Praociini. Synonymy: Fairmaire (1884a: 508).

*Edylius* Champion, 1894a: 353 [M]. Type species: *Edyliuscanescens* Champion, 1894, by monotypy. Status: valid genus in Tenebrioninae: Heleini: Heleina.

*Eichleria* Kamiński, 2015b: 132 [F]. Type species: *Eichleriaostrowskii* Kamiński, 2015, by original designation. Status: valid genus in Blaptinae: Opatrini: Stizopodina.

*Elaeodes* Gemminger in Gemminger and Harold, 1870: 1868 [F]. Type species [automatic]: *Eleodesdentipes* Eschscholtz, 1829, by subsequent designation (Hope 1841: 124). Status: junior synonym of *Eleodes* Eschscholtz, 1829 in Blaptinae: Amphidorini. Note: unjustified emendation of *Eleodes* Eschscholtz, 1829, not in prevailing usage.

*Elaeus* Gemminger in Gemminger and Harold, 1870: 1969 [M] Type species [automatic]: *Heleaperforata* Latreille, 1816, by subsequent monotypy (Latreille 1817: 261). Status: junior synonym of *Helea* Latreille, 1804 in Tenebrioninae: Heleini: Heleina. Note: unjustified emendation of *Helea* Latreille, 1804 (as “*Helaeus* Latreille”), not in prevailing usage.

*Elasmocera* Mäklin, 1867: 504 [F]. Type species: *Elasmoceradentipes* Mäklin, 1867, by monotypy. Status: senior synonym of *Elasmocerella* Strand, 1935 in Stenochiinae: Stenochiini. Note: junior homonym of *Elasmocera* Rondani, 1846 [Diptera].

*Elasmocerella* Strand, 1935b: 302 [F]. Type species [automatic]: *Elasmoceradentipes* Mäklin, 1867, by monotypy. Status: valid genus in Stenochiinae: Stenochiini. Note: replacement name for *Elasmocera* Mäklin, 1867.

*Eleates* Casey, 1886: 253 [M]. Type species: *Eleatesoccidentalis* Casey, 1886, by monotypy. Status: valid genus in Tenebrioninae: Bolitophagini.

*Eledona* Latreille, 1797: 19 [F]. Type species: *Opatrumagricola* Herbst, 1783, by subsequent monotypy (Latreille 1802: 162). Status: valid genus in Tenebrioninae: Bolitophagini. Note: originally proposed without included nominal species; Latreille (1802: 162)) , by including the species “*Bolitophagusagricola*” in association with this name, was the first author to subsequently and expressly include nominal species in *Eledona* (ICZN 1999, Article 67.2.2).

*Eledonoprius* Reitter, 1911: 329, 338 [M]. Type species: *Opatrumarmatum* Panzer, 1799, by subsequent designation (R. Lucas 1920: 258). Status: valid genus in Tenebrioninae: Bolitophagini.

*Elenophorus* Dejean, 1821: 64 [M]. Type species: *Tenebriocollaris* Linnaeus, 1767, by monotypy. Status: junior synonym of *Leptoderis* Billberg, 1820 in Pimeliinae: Elenophorini: Elenophorina. Synonymy: Silfverberg (1984: 59).

*Eleodes* Eschscholtz, 1829: 8 [F]. Type species: *Eleodesdentipes* Eschscholtz, 1829, by subsequent designation (Hope 1841: 124). Status: valid genus and subgenus in Blaptinae: Amphidorini.

*Eleodimorpha* Blaisdell, 1909: 477 [F]. Type species: *Eleodimorphabolcan* Blaisdell, 1909, by original designation. Status: valid genus in Blaptinae: Amphidorini.

*Eleodopsis* Blaisdell, 1939: 52 [F]. Type species: *Eleodopsissubvestita* Blaisdell, 1939, by original designation. Status: junior synonym of *Blapylis* Horn, 1870 in Blaptinae: Amphidorini. Synonymy: Spilman (1962a: 59).

*Eleoselinus* Kamiński, 2014: 101 [M]. Type species: *Ectateusvilliersi* Ardoin, 1965, by original designation. Status: valid genus in Blaptinae: Platynotini: Platynotina.

*Elixota* Pascoe, 1866a: 475 [F]. Type species: *Elixotacuprea* Pascoe, 1866 (= *Amarygmushopei* Bremer, 2001), by original designation. Status: junior synonym of *Amarygmus* Dalman, 1823 in Tenebrioninae: Amarygmini. Synonymy: Bremer (2001a: 65).

*Ellaemus* Pascoe, 1866c: 495 [M]. Type species: *Emcephalussubmaculatus* Brême, 1842, by **present designation**. Status: junior synonym of *Emcephalus* W. Kirby, 1828 in Tenebrioninae: Heleini: Heleina. Synonymy: Matthews and Bouchard (2008: 304).

*Ellidoneus* Wilke, 1922: 277 [M]. Type species: *Cardigeniusgranulatus* Fairmaire, 1873, by original designation. Status: valid subgenus of *Cardigenius* Solier, 1836 in Pimeliinae: Asidini.

*Ellipsodes* Wollaston, 1854: 485 [M]. Type species: *Sphaeridiumglabratum* Fabricius, 1781, by monotypy. Status: valid genus and subgenus in Diaperinae: Crypticini.

*Elomosda* Bates, 1870: 273 [F]. Type species: *Elomosdabeltii* Bates, 1870, by monotypy. Status: valid genus in Stenochiinae: Cnodalonini.

*Elongasida* Escalera, 1906: 306 [F]. Type species: *Asidagrandipalpis* Allard, 1869, by subsequent designation (Viñolas and Cartagena 2005: 164). Status: valid subgenus of *Alphasida* Escalera, 1905 in Pimeliinae: Asidini.

*Embaphion* Say, 1824: 254 [N]. Type species: *Akismuricata* Say, 1824, by monotypy. Status: valid genus in Blaptinae: Amphidorini.

*Emcephalus* W. Kirby, 1828: 524 [M]. Type species: *Emcephalusgibbosus* W. Kirby, 1828, by monotypy. Status: valid genus in Tenebrioninae: Heleini: Heleina.

*Emeacoides* Gebien, 1937a: 698 [M]. Type species [automatic]: *Enneacoidesvinculiger* Fairmaire, 1881 (= *Nyctoporiscarinata* J.L. LeConte, 1851), by monotypy. Status: junior synonym of *Nyctoporis* Eschscholtz, 1831 in Pimeliinae: Nyctoporini. Note: unjustified emendation of *Enneacoides* Fairmaire, 1881, not in prevailing usage.

*Emeax* Pascoe, 1866a: 450 [M]. Type species: *Emeaxsculpturatus* Pascoe, 1866 (= *Nyctoporiscristata* Eschscholtz, 1831), by monotypy. Status: junior synonym of *Nyctoporis* Eschscholtz, 1831 in Pimeliinae: Nyctoporini. Synonymy: J.L. LeConte (1873: 334). Note: as mentioned by Matthews and Bouchard (2008: 351) the type species was originally described from Australia in error; the genus *Nyctoporis* Eschscholtz, 1831 is endemic to the Nearctic realm.

*Emmallodera* Blanchard, 1842: pl. 13 [F]. Type species: *Emmalloderacrenaticostata* Blanchard, 1842, by monotypy. Status: valid genus in Tenebrioninae: Scotobiini. Note: unjustified emendation of the original spelling *Emalodera*, introduced by Agassiz (1846b: 137), in prevailing usage and treated as a justified emendation (ICZN 1999, Article 33.2.3.1).

*Emmallus* Agassiz, 1846b: 137 [M]. Type species [automatic]: *Emmaluspilosus* Erichson, 1843, by monotypy. Status: junior synonym of *Emmalus* Erichson, 1843 in Blaptinae: Opatrini: Ammobiina. Note: unjustified emendation of *Emmalus* Erichson, 1843, not in prevailing usage.

*Emmalus* Erichson, 1843: 251 [M]. Type species: *Emmaluspilosus* Erichson, 1843, by monotypy. Status: valid genus in Blaptinae: Opatrini: Ammobiina.

*Emmenastrichus* Horn, 1894: 413 [M]. Type species: *Emmenastrichuscribratus* Horn, 1894, by subsequent designation (Casey 1907: 289). Status: valid genus in Pimeliinae: Edrotini.

*Emmenastus* Motschulsky, 1845a: 75 [M]. Type species: *Emmenastusrugosus* Motschulsky, 1845, by subsequent designation (J.L. LeConte 1866b: 106). Status: junior synonym of *Oxycara* Solier, 1835 in Pimeliinae: Tentyriini. Synonymy: Aalbu et al. (1995: 484).

*Emmenides* Casey, 1907: 329 [M]. Type species: *Emmenastuspunctatus* J.L. LeConte, 1866, by original designation. Status: valid genus in Pimeliinae: Edrotini.

*Emydodes* Pascoe, 1860a: 56 [M]. Type species: *Emydodescollaris* Pascoe, 1860, by monotypy. Status: valid genus in Lagriinae: Lagriini: Lagriina.

*Emyon* Gerstaecker, 1854: 532 [M]. Type species: *Emyoncaelatus* Gerstaecker, 1854, by monotypy. Status: junior synonym of *Helopinus* Solier, 1848 in Blaptinae: Pedinini: Helopinina. Synonymy: Koch (1958: 149).

*Emypsara* Pascoe, 1866a: 460 [F]. Type species: *Emypsaraadamsii* Pascoe, 1866 (= *Diaperisriederii* Faldermann, 1833), by subsequent designation (Löbl et al. 2008a: 41). Status: valid genus in Diaperinae: Phaleriini.

*Enanea* Lewis, 1894: 467 [F]. Type species: *Enaneatestacea* Lewis, 1894, by monotypy. Status: valid genus in Diaperinae: Gnathidiini: Gnathidiina.

*Encara* Gemminger, 1870: 124 [N]. Type species [automatic]: *Emcephalusgibbosus* W. Kirby, 1828, by monotypy. Status: junior synonym of *Emcephalus* W. Kirby, 1828 in Tenebrioninae: Heleini: Heleina. Note: replacement name for *Emcephalus* W. Kirby, 1828 (as “*Encephalus* Brême”).

*Encephalus* Agassiz, 1846b: 137 [M]. Type species [automatic]: *Emcephalusgibbosus* W. Kirby, 1828, by monotypy. Status: junior synonym of *Emcephalus* W. Kirby, 1828 in Tenebrioninae: Heleini: Heleina. Note: unjustified emendation of *Emcephalus* W. Kirby, 1828, not in prevailing usage; junior homonym of *Encephalus* Stephens, 1832 [Coleoptera: Staphylinidae].

*Encyalesthus* Motschulsky, 1860d: 139 [M]. Type species: *Encyalesthussubviolaceus* Motschulsky, 1860, by monotypy. Status: junior synonym of *Derosphaerus* J. Thomson, 1858 in Stenochiinae: Cnodalonini. Synonymy: Kaszab (1987: 43).

*Endostomus* Gemminger in Gemminger and Harold, 1870: 1973 [M]. Type species [automatic]: *Cossyphussenegalensis* Laporte, 1833, by subsequent monotypy (Duponchel 1844b: 315). Status: junior synonym of *Endustomus* Brême, 1842 in Lagriinae: Cossyphini. Note: unjustified emendation of *Endustomus* Brême, 1842, not in prevailing usage.

*Endostostomus* Jakobson, 1914: 528 [M]. Type species [automatic]: *Cossyphussenegalensis* Laporte, 1833, by subsequent monotypy (Duponchel 1844b: 315). Status: junior synonym of *Endustomus* Brême, 1842 in Lagriinae: Cossyphini. Note: unjustified emendation of *Endustomus* Brême, 1842, not in prevailing usage.

*Endothina* Carter, 1924b: 536 [F]. Type species: *Endothinasquamosa* Carter, 1924 (= *Opatrumcanaliculatum* Fabricius, 1798), by monotypy. Status: junior synonym of *Leichenum* Dejean, 1834 in Blaptinae: Pedinini: Leichenina. Synonymy: Carter (1928: 284).

*Endroeditagalus* Schawaller & Bouchard, 2019: 192 [M]. Type species: *Endroeditagalusntsubanus* Schawaller & Bouchard, 2019, by original designation. Status: valid genus in Phrenapatinae: Penetini.

*Endustomus* Brême, 1842b: 17 [M]. Type species: *Cossyphussenegalensis* Laporte, 1833, by subsequent monotypy (Duponchel 1844b: 315). Status: valid genus in Lagriinae: Cossyphini. Note: originally proposed without included nominal species; Duponchel (1844b: 315), by including the species “*Cossyphussenegalensis*” in association with this name, was the first author to subsequently and expressly include nominal species in *Endustomus* (ICZN 1999, Article 67.2.2).

*Enganodia* Fairmaire, 1898d: 398 [F]. Type species: *Enganodiasanguinicrus* Fairmaire, 1898, by monotypy. Status: junior synonym of *Lophocnemis* Mäklin, 1867 in Stenochiinae: Stenochiini. Synonymy: Gebien (1948: 543).

*Enicmonota* Ardoin, 1959a: 61 [F]. Type species: *Enicmosomacrassicorne* Ardoin, 1959, by monotypy. Status: junior synonym of *Enicmosoma* Gebien, 1922 in Lagriinae: Lupropini. Synonymy: Schawaller (2013a: 139).

*Enicmosoma* Gebien, 1922b: 312 [N]. Type species: *Enicmosomapunctum* Gebien, 1922, by subsequent designation (Gebien 1941: 823). Status: valid genus in Lagriinae: Lupropini.

*Enigmatica* Ferrer, 2005: 202 [F]. Type species: *Enigmaticaendroedyi* Ferrer, 2005, by monotypy. Status: valid genus in Lagriinae: Laenini.

*Enneacoides* Fairmaire, 1881c: 277 [M]. Type species: *Enneacoidesvinculiger* Fairmaire, 1881 (= *Nyctoporiscarinata* J.L. LeConte, 1851), by monotypy. Status: junior synonym of *Nyctoporis* Eschscholtz, 1831 in Pimeliinae: Nyctoporini. Synonymy: Gebien (1908b: 287).

*Ennychiatus* Koch, 1963: 28 [M]. Type species: *Stizopuscaraboides* Fairmaire, 1897, by original designation. Status: junior synonym of *Parastizopus* Gebien, 1938 in Blaptinae: Opatrini: Stizopodina. Synonymy: Iwan and Schimrosczyk (2017: 384).

*Ennychius* Fåhraeus, 1870: 299 [M]. Type species: *Ennychiusmorio* Fåhraeus, 1870, by monotypy. Status: junior synonym of *Helibatus* Mulsant & Rey, 1859 in Blaptinae: Opatrini: Stizopodina. Synonymy: Gebien (1938a: 74).

*Enoplopus* Solier, 1848: 151, 158 [M]. Type species [automatic]: **fixed herein** (ICZN 1999, Article 70.3) as *Tenebriovelikensis* Piller & Mitterpacher, 1783, misidentified as *Tenebriocaraboides* Linnaeus, 1758 in the original designation by monotypy in Dejean (1821). Status: junior synonym of *Accanthopus* Dejean, 1821 in Tenebrioninae: Helopini: Enoplopodina. Note: unnecessary replacement name for *Accanthopus* Dejean, 1821.

*Entinopoda* Gebien, 1938b: 64 [F]. Type species: *Eustolopusoctoseriatus* Gebien, 1938, by monotypy. Status: junior synonym of *Eustolopus* Gebien, 1938 in Pimeliinae: Adesmiini. Synonymy: Penrith (1979: 40).

*Entomobalia* Flores & Triplehorn, 2002: 607 [F]. Type species: *Asidaplatynotos* Perty, 1830, by original designation. Status: valid genus in Pimeliinae: Nycteliini.

*Entomochilus* Gay & Solier in Solier, 1843: 48 [M]. Type species: *Entomochiluspilosus* Gay & Solier, 1843, by original designation. Status: valid genus in Pimeliinae: Physogasterini.

*Entomoderes* Solier, 1836: 308, 346 [M]. Type species: *Entomodereserebi* Solier, 1836, by monotypy. Status: valid genus in Pimeliinae: Nycteliini.

*Entomogonus* Solier, 1848: 151, 155 [M]. Type species: *Entomogonusbarthelemyi* Solier, 1848, by monotypy. Status: valid genus and subgenus in Tenebrioninae: Helopini: Helopina.

*Entypodera* Gerstaecker, 1871: 66 [F]. Type species: *Entypoderaanthicoides* Gerstaecker, 1871, by monotypy. Status: valid genus in Lagriinae: Lagriini: Statirina. Note: **new placement** [OM], previously included in Lagriinae: Lagriini: Lagriina.

†*Eoallognosis* Haupt, 1950: 115, 137 [F]. Type species: *Eoallognosisundulata* Haupt, 1950, by original designation. Status: valid genus in Tenebrionidae: incertae sedis. Note: described from Middle Eocene deposits (Germany).

†*Eocallidium* Haupt, 1950: 143 [N]. Type species: *Eocallidiumrugulosum* Haupt, 1950, by monotypy. Status: valid genus in Tenebrionidae: incertae sedis. Note: originally included in the family Cerambycidae, transferred to Tenebrionidae by Vitali (2008: 8); described from Middle Eocene deposits (Germany).

*Eocyphogenia* G.S. Medvedev, 1968b: 897, 898 [F]. Type species: *Akisrugipennis* Faldermann, 1835, by original designation. Status: junior synonym of *Cyphogenia* Solier, 1837 in Pimeliinae: Akidini. Synonymy: Ren and Yu (2000: 54).

*Eodirosis* Kwieton, 1980: 25 [F]. Type species: *Erodiusquadrilineatus* Kraatz, 1865, by original designation. Status: valid subgenus of *Erodius* Fabricius, 1775 in Pimeliinae: Erodiini. Note: the original combination of the accepted name of the type species, *Erodiusquadrilineatus* Kraatz, 1865, is a junior primary homonym of *Erodiusquadrilineatus* G.-A. Olivier, 1792.

†*Eodromus* Haupt, 1950: 113, 120 [M] Type species: †*Ancylochiraagilis* Meunier, 1915, by original designation. Status: valid genus in Stenochiinae: incertae sedis. Note: originally proposed in the family Carabidae by Pongrácz (1935: 538) without type species (ICZN 1999, Article 13.3); we act as First Revisers and reject the alternative original spelling *Edromus*, used by Haupt (1950: 120); described from Middle Eocene deposits (Germany).

†*Eohelaeus* Haupt, 1950: 115, 135 [M]. Type species: *Eohelaeussublaevis* Haupt, 1950, by original designation. Status: valid genus in Tenebrionidae: incertae sedis. Note: described from Middle Eocene deposits (Germany).

*Epairops* Fåhraeus, 1870: 282 [M]. Type species: *Epairopsfragilis* Fåhraeus, 1870, by monotypy. Status: junior synonym of *Ossiporis* Pascoe, 1866 in Pimeliinae: Sepidiini: Trachynotina. Synonymy: Gebien (1937b: 37).

*Epairopsis* Koch, 1955a: 47 [F]. Type species: *Trachynotusfrontalis* Haag-Rutenberg, 1873, by original designation. Status: valid genus in Pimeliinae: Sepidiini: Trachynotina.

*Epantius* J.L. LeConte, 1851: 144 [M]. Type species: *Epantiusobscurus* J.L. LeConte, 1851, by monotypy. Status: valid genus in Tenebrioninae: Eulabini.

*Epeurycaulus* Kolbe, 1902a: 579 [M]. Type species: *Epeurycaulusaldabricus* Kolbe, 1902, by **present designation**. Status: junior synonym of *Plesioderes* Mulsant, 1859 in Blaptinae: Opatrini: Ammobiina. Synonymy: Gebien (1922b: 268).

*Ephalus* J.L. LeConte, 1862: 228 [M]. Type species: *Heliopateslatimanus* J.L. LeConte, 1847, by monotypy. Status: valid genus in Blaptinae: Opatrini: Opatrina. Note: transferred from Ammobiina by Lumen et al. (2020: 344).

*Ephidonius* Pascoe, 1869: 151 [M]. Type species: *Ephidoniusacuticornis* Pascoe, 1869, by original designation. Status: junior synonym of *Brises* Pascoe, 1869 in Tenebrioninae: Heleini: Heleina. Synonymy: Carter (1914b: 46).

*Epicalla* Lacordaire, 1859a: 309 [F]. Type species: *Epicallavaripes* Champion, 1886, by subsequent designation (R. Lucas 1920: 268). Status: valid genus in Stenochiinae: Cnodalonini. Note: the authorship of this genus name has been credited to Champion (1886: 249) in the literature; however, Lacordaire (1859a: 309) mentioned that the genus *Epicalla* has the “mandibules entières au bout”, which fulfils the requirements of availability (ICZN 1999, Article 12.1); Champion (1886: 249–251), by describing three new species in association with the genus *Epicalla*, was the first author to subsequently and expressly include nominal species in this genus (ICZN 1999, Article 67.2.2).

*Epicydes* Champion, 1889: 60 [M]. Type species: *Epicydesoculatus* Champion, 1889, by subsequent designation (Borchmann 1936: 429). Status: valid genus and subgenus in Lagriinae: Lagriini: Statirina.

*Epilamprus* Gistel, 1848a: xiv [M]. Type species [automatic]: *Helopsindutus* Wiedemann, 1819, by subsequent designation (Duponchel 1844b: 359). Status: junior synonym of *Ceropria* Laporte & Brullé, 1831 in Diaperinae: Diaperini: Diaperina. Note: unjustified emendation of *Epilampus* Dejean, 1834, not in prevailing usage.

*Epilampus* Dejean, 1834: 198 [M]. Type species [automatic]: *Helopsindutus* Wiedemann, 1819, by subsequent designation (Duponchel 1844b: 359). Status: junior synonym of *Ceropria* Laporte & Brullé, 1831 in Diaperinae: Diaperini: Diaperina. Note: unnecessary replacement name for *Ceropria* Laporte & Brullé, 1831 (see Bousquet and Bouchard 2013a: 52).

*Epilasium* Erichson, 1842a: 237 [N]. Type species: *Trichotoncayennense* Hope, 1841, by monotypy. Status: junior synonym of *Trichoton* Hope, 1841 in Blaptinae: Opatrini: Blapstinina. Synonymy: Erichson (1842a: 237). Note: the name *Epilasium* was listed as synonym of *Trichoton* Hope, 1841 by Erichson (1842a: 237), it was treated before 1961 as an available name and adopted as the name of a taxon (e.g., Curtis 1844: 222), *Epilasium* was therefore made available from its first publication as a synonym (ICZN 1999, Article 11.6.1).

*Epipagus* Haag-Rutenberg, 1872: 274, 311 [M]. Type species: *Epipagusbenguelensis* Haag-Rutenberg, 1872, by monotypy. Status: valid genus in Pimeliinae: Cryptochilini: Cryptochilina.

*Epipedodema* Gebien, 1921b: 54 [F]. Type species: *Epipedodemadepressa* Gebien, 1921, by monotypy. Status: valid genus in Tenebrioninae: Alphitobiini.

*Epipedonota* Solier, 1836: 307, 342 [F]. Type species: *Epipedonotaebenina* Solier, 1836, by subsequent designation (Solier 1851: 159). Status: valid genus in Pimeliinae: Nycteliini. Note: the alternative original spelling *Epipedonata*, used by Solier (1836: 342), was rejected by Solier (1838a: 487) who acted as the First Reviser.

*Epiphaleria* Lewis, 1894: 382 [F]. Type species: *Epiphaleriaatriceps* Lewis, 1894, by original designation. Status: valid subgenus of *Phaleria* Latreille, 1802 in Diaperinae: Phaleriini.

*Epiphysa* Dejean, 1834: 178 [F]. Type species: *Pimeliaflavicollis* Fabricius, 1794, by monotypy. Status: valid genus in Pimeliinae: Adesmiini.

*Epiplecta* Mäklin, 1867: 498 [F]. Type species: *Epiplectamaculata* Mäklin, 1867, by monotypy. Status: valid genus in Stenochiinae: Stenochiini.

*Episopus* Bates, 1873e: 372 [M]. Type species: *Episopuspolitus* Bates, 1873, by subsequent designation (R. Lucas 1920: 270). Status: valid genus in Stenochiinae: Cnodalonini.

*Epitoxicum* Bates, 1873d: 46 [N]. Type species: *Epitoxicumhaplandroides* Bates, 1873, by monotypy. Status: valid genus in Tenebrioninae: Toxicini: Toxicina.

*Epitragella* Kulzer, 1958b: 184 [F]. Type species: *Epitragelladimorpha* Kulzer, 1958, by original designation. Status: valid genus in Pimeliinae: Epitragini.

*Epitragodes* Casey, 1890b: 365 [M]. Type species: *Epitragustomentosus* J.L. LeConte, 1866, by monotypy. Status: valid genus in Pimeliinae: Epitragini.

*Epitragoma* Casey, 1907: 386 [N]. Type species: *Epitragusvestitus* Casey, 1891, by monotypy. Status: junior synonym of *Pechalius* Casey, 1907 in Pimeliinae: Epitragini. Synonymy: Freude (1968: 61).

*Epitragopsis* Casey, 1907: 386 [F]. Type species: *Epitragusgodmani* Champion, 1884, by original designation. Status: valid genus in Pimeliinae: Epitragini.

*Epitragosoma* K.W. Brown & Triplehorn, 2002: 515 [N]. Type species: *Epitragosomaarenaria* K.W. Brown & Triplehorn, 2002, by original designation. Status: valid genus in Pimeliinae: Epitragini.

*Epitragus* Latreille, 1802: 165 [M]. Type species: *Epitragusfuscus* Latreille, 1804, by subsequent monotypy (Latreille 1804: 322). Status: valid genus and subgenus in Pimeliinae: Epitragini. Note: originally proposed without included nominal species since the species listed (*Helopsvariegatus* ? Fab.) is conditionally included; Latreille (1804: 322), by including the species *Epitragusfuscus* Latreille, 1804 in association with this name, was the first author to subsequently and expressly include a nominal species in *Epitragus* (ICZN 1999, Article 67.2.2).

*Epitrichia* Gebler, 1859: 475 [F]. Type species: *Helopstomentosus* Gebler, 1842, by monotypy. Status: valid genus in Pimeliinae: Tentyriini.

*Epityria* Koch, 1950b: 298 [F]. Type species: *Himatismusstriatopunctatus* Haag-Rutenberg, 1877, by original designation. Status: valid subgenus of *Derosphaerius* Westwood, 1881 in Pimeliinae: Tentyriini.

*Epomidus* Matthews, 1998: 705, 763 [M]. Type species: *Epomidusprionodes* Matthews, 1998, by original designation. Status: valid genus in Lagriinae: Adeliini.

*Erelus* Mulsant & Rey, 1853a: 185 [M]. Type species: *Erelussulcipennis* Mulsant & Rey, 1853, by monotypy. Status: valid genus in Tenebrionidae: incertae sedis. Note: included in a list of “Tenebrionidae*nomina dubia*” by Iwan et al. (2020: 475).

*Eremobates* Gebien, 1921b: 120 [M]. Type species: *Eremobatescrux* Gebien, 1921, by monotypy. Status: senior synonym of *Eremobatodes* Gebien, 1943 in Stenochiinae: Cnodalonini. Note: junior homonym of *Eremobates* Banks, 1900 [Arachnida].

*Eremobatodes* Gebien, 1943: 404 [M]. Type species [automatic]: *Eremobatescrux* Gebien, 1921, by monotypy. Status: valid genus in Stenochiinae: Cnodalonini. Note: replacement name for *Eremobates* Gebien, 1921.

*Eremocantor* Smith & Wirth, 2016: 582 [M]. Type species: *Eremocantormarioni* Smith & Wirth, 2016, by original designation. Status: valid genus in Pimeliinae: Edrotini.

*Eremoecus* Lacordaire, 1859a: 69 [M]. Type species: *Hyperopseschscholtzii* Solier, 1851, by monotypy. Status: valid genus in Pimeliinae: Trilobocarini.

*Eremonomus* Wollaston, 1861: 199 [M]. Type species: *Eremonomushuttoni* Wollaston, 1861 (= *Ammidiumciliatum* Erichson, 1843), by monotypy. Status: junior synonym of *Ammidium* Erichson, 1843 in Blaptinae: Opatrini: Ammobiina. Synonymy: Ardoin (1971: 358).

*Eremophaleria* Español, 1951: 32, 34 [F]. Type species: *Phaleriabedeli* Chobaut, 1900, by original designation. Status: valid subgenus of *Phaleria* Latreille, 1802 in Diaperinae: Phaleriini.

*Eremostibes* Koch, 1963: 60 [M]. Type species: *Eremostibesopacus* Koch, 1963, by original designation. Status: valid genus in Blaptinae: Opatrini: Stizopodina.

*Ergenna* Fairmaire, 1897f: 139 [F]. Type species: *Ergennacaerulescens* Fairmaire, 1897, by monotypy. Status: junior synonym of *Praeugena* Laporte, 1840 in Tenebrioninae: Praeugenini. Synonymy: De Moor (1970: 42).

*Erionura* Reitter, 1903: 18 [F]. Type species: *Helopsgiganteus* Kraatz, 1862, by monotypy. Status: valid genus in Tenebrioninae: Helopini: Helopina.

*Ernocharis* C.G. Thomson, 1859: 118 [F]. Type species: *Cistelabrevis* Illiger, 1794 (= *Cistelamaura* Fabricius, 1792), by original designation. Status: valid subgenus of *Mycetochara* Guérin-Méneville, 1827 in Alleculinae: Alleculini: Mycetocharina.

*Erodibius* Löbl, Bouchard, Merkl & Bousquet, 2020: 2 [M]. Type species: *Arthrodeiscyphonotus* Fairmaire, 1887, by original designation. Status: valid subgenus of *Arthrodibius* Lesne, 1915 in Pimeliinae: Erodiini. Note: name first proposed by Koch (1960: 354) without fixation of a type species in the original publication (ICZN 1999, Article 13.3); Löbl et al. (2008a: 40) designated *Arthrodeiscyphonotus* Fairmaire, 1887 as the type species of Koch’s name but did not explicitly indicate the genus-group name as intentionally new (ICZN 1999, Article 16.1).

*Erodinus* Reitter, 1900b: 299 [M]. Type species: none designated. Status: undetermined taxon in Pimeliinae: Erodiini. Note: this genus was included in a key, which fulfils the criteria of availability for new genus-group names proposed before 1931 (ICZN 1999, Article 12.1); however, we could not find any nominal species that were subsequently and expressly included in *Erodinus* and therefore no “originally included nominal species” could be used to fix the type species (ICZN 1999, Article 67.2.2).

*Erodiontes* Reitter, 1914a: 48, 79 [M]. Type species: *Erodiontesvermiculatus* Reitter, 1914, by monotypy. Status: valid genus in Pimeliinae: Erodiini.

*Erodius* Fabricius, 1775: 258 [M]. Type species: *Erodiusgibbus* Fabricius, 1775, by subsequent designation (Latreille 1810: 429). Status: valid genus and subgenus in Pimeliinae: Erodiini.

*Erulipothydemus* Pic, 1918b: 19 [M]. Type species: *Erulipothydemuslatior* Pic, 1918, by subsequent designation (Gebien 1943: 902). Status: valid genus in Tenebrioninae: Helopini: incertae sedis.

*Erulipus* Fairmaire, 1903a: 14 [M]. Type species: *Erulipusfruhstorferi* Fairmaire, 1903, by monotypy. Status: valid subgenus of *Ainu* Lewis, 1894 in Stenochiinae: Cnodalonini.

*Erxias* Champion, 1888: 460 [M]. Type species: *Erxiasbicolor* Champion, 1888, by subsequent designation (Bousquet et al. 2015: 138). Status: valid genus in Alleculinae: Alleculini: Xystropodina.

*Erycastus* Fairmaire, 1897f: 133 [M]. Type species: *Erycastusnavicularis* Fairmaire, 1897, by monotypy. Status: valid genus in Tenebrioninae: Amarygmini.

*Eryx* Stephens, 1832b: 27 [M]. Type species: *Pyrochroanigra* DeGeer, 1775 (= *Helopsater* Fabricius, 1775), by monotypy. Status: senior synonym of *Prionychus* Solier, 1835 in Alleculinae: Alleculini: Alleculina. Synonymy: Mulsant (1856a: 12, 61). Note: junior homonym of *Eryx* Daudin, 1803 [Reptilia].

*Erzika* Novák, 2020d: 53 [F]. Type species: *Erzikatamdaoica* Novák, 2020, by original designation. Status: valid genus in Alleculinae: Alleculini: Alleculina.

*Eschatomoxys* Blaisdell, 1935: 125 [M]. Type species: *Eschatomoxyswagneri* Blaisdell, 1935, by original designation. Status: valid genus in Pimeliinae: Edrotini.

*Eschatoporis* Blaisdell, 1906: 76 [F]. Type species: *Eschatoporisnunenmacheri* Blaisdell, 1906, by monotypy. Status: valid genus in Lagriinae: Eschatoporiini.

*Eschatostena* Keleinikova, 1977: 654 [F]. Type species: *Eschatostenakuznetzovi* Keleinikova, 1977, by monotypy. Status: valid genus in Pimeliinae: Tentyriini.

*Esemephe* Steiner, 1980: 385 [F]. Type species: *Esemephetumi* Steiner, 1980, by original designation. Status: valid genus in Pimeliinae: Cossyphodini: Esemephina.

*Espagnolina* Kaszab, 1965: 117 [F]. Type species: *Espagnolinaassamica* Kaszab, 1965, by original designation. Status: valid genus in Diaperinae: Diaperini: Diaperina.

*Espidium* Rafinesque, 1815: 113 [N]. Type species [automatic]: *Sepidium tricuspidatum* Fabricius, 1775, by subsequent designation (Latreille 1810: 429). Status: junior synonym of *Sepidium* Fabricius, 1775 in Pimeliinae: Sepidiini: Sepidiina. Note: unnecessary replacement name for *Sepidium* Fabricius, 1775.

*Espites* Pascoe, 1882: 32 [M]. Type species: *Espitesbasalis* Pascoe, 1882, by monotypy. Status: valid genus in Stenochiinae: Cnodalonini.

*Espitomorphus* Pic, 1921d: 24 [M]. Type species: *Espitomorphusmulticolor* Pic, 1921, by monotypy. Status: junior synonym of *Camarimena* Motschulsky, 1863 in Stenochiinae: Cnodalonini. Synonymy: Kaszab (1983a: 136).

*Etazeta* Fairmaire, 1889a: 358 [F]. Type species: *Etazetaaeneicolor* Fairmaire, 1889, by monotypy. Status: junior synonym of *Luprops* Hope, 1833 in Lagriinae: Lupropini. Synonymy: Gebien (1914a: 35).

*Ethas* Pascoe, 1862: 324 [M]. Type species: *Ethascarbonarius* Pascoe, 1862, by monotypy. Status: valid genus in Pimeliinae: Stenosini: Stenosina.

*Ethmomerus* Koch, 1953a: 243 [M]. Type species: *Ethmussubcylindricus* Koch, 1953, by original designation. Status: valid subgenus of *Ethmus* Haag-Rutenberg, 1873 in Pimeliinae: Sepidiini: Trachynotina.

*Ethmophobes* Koch, 1953a: 244 [M]. Type species: *Ethmuslatus* Haag-Rutenberg, 1873, by original designation. Status: valid subgenus of *Ethmus* Haag-Rutenberg, 1873 in Pimeliinae: Sepidiini: Trachynotina.

*Ethmus* Haag-Rutenberg, 1873: 44 [M]. Type species: *Ethmusmaculatus* Haag-Rutenberg, 1873, by subsequent designation (Gebien 1937a: 778). Status: valid genus and subgenus in Pimeliinae: Sepidiini: Trachynotina. Note: nomenclatural stability is threatened by the discovery of an older type species designation (*Ethmuslatus* Haag-Rutenberg, 1873, by subsequent designation by R. Lucas (1920: 276), which is the type species of the valid subgenusEthmophobes Koch, 1953); we recommend that an application be submitted to the International Commission on Zoological Nomenclature to maintain the type species designation proposed by Gebien (1937a: 778).

*Eubalia* Laporte, 1840: 257 [F]. Type species: *Statiraovalis* Laporte, 1840, by monotypy. Status: valid genus in Alleculinae: Alleculini: Gonoderina.

*Euboeus* Boieldieu, 1865: 10 [M]. Type species: *Euboeusmimonti* Boieldieu, 1865, by monotypy. Status: valid genus in Tenebrioninae: Helopini: Helopina.

*Eucaliga* Fairmaire & Germain, 1861: 5 [F]. Type species: *Eucaligasanguinicollis* Fairmaire & Germain, 1861, by monotypy. Status: valid genus in Alleculinae: Alleculini: Alleculina.

*Eucamaria* Gebien, 1919: 28, 149 [F]. Type species: *Camariaspectabilis* Pascoe, 1860, by original designation. Status: junior synonym of *Falsocamaria* Pic, 1917 in Stenochiinae: Cnodalonini. Synonymy: Masumoto (1993a: 142).

*Eucamptus* Germar, 1842: 444 [M]. Type species: *Eucamptusiridis* Germar, 1842 (= *Hegemonaresplendens* Laporte, 1840), by monotypy. Status: junior synonym of *Hegemona* Laporte, 1840 in Stenochiinae: Cnodalonini. Synonymy: Duponchel (1845b: 498).

*Euclarkia* Lea, 1919: 180 [F]. Type species: *Euclarkiacostata* Lea, 1919, by monotypy. Status: valid genus in Lagriinae: Belopini.

*Eucolus* Mulsant & Rey, 1853b: 39, 67 [M]. Type species: *Eucoluspolinierii* Mulsant & Rey, 1853, by monotypy. Status: valid genus in Blaptinae: Platynotini: Platynotina.

*Euconibius* Casey, 1895: 618 [M]. Type species: *Notibiusgagates* Horn, 1870, by monotypy. Status: junior synonym of *Conibius* J.L. LeConte, 1851 in Blaptinae: Opatrini: Blapstinina. Synonymy: Aalbu in Bousquet et al. (2018: 202).

*Eucosmus* Gistel, 1848a: x [M]. Type species [automatic]: *Spheniscuserotyloides* W. Kirby, 1819, by monotypy. Status: junior synonym of *Cuphotes* Champion, 1887 in Stenochiinae: Stenochiini. Note: replacement name for *Spheniscus* W. Kirby, 1819; junior homonym of *Eucosmus* Agassiz, 1846 [Echinoidea].

*Eucrossoscelis* Nakane, 1963: 29 [F]. Type species: *Eucrossoscelisbroscosomoides* Nakane, 1963, by monotypy. Status: valid genus in Stenochiinae: Stenochiini.

*Eucyrtus* Lacordaire, 1859b: 417 [M]. Type species: *Eucyrtuspretiosus* Lacordaire, 1859, by subsequent designation (Gebien 1921a: 335). Status: valid genus in Stenochiinae: Cnodalonini. Note: the name *Eucyrtus* was listed as synonym of *Scotaeus* Hope, 1834 by Lacordaire (1859b: 417); as it was treated before 1961 as an available name and adopted as the name of a taxon (e.g., Pascoe 1866a: 473), *Eucyrtus* was therefore made available from its first publication as a synonym (ICZN 1999, Article 11.6.1).

*Eudissonomus* G.S. Medvedev, 1968a: 218 [M]. Type species: *Heterophylussubstriatus* Reitter, 1898, by original designation. Status: valid subgenus of *Dissonomus* Jacquelin du Val, 1861 in Tenebrioninae: Dissonomini.

*Eudysantes* Bouchard, Lawrence, Davies & Newton, 2005: 508 [M]. Type species [automatic]: *Dicerodereselongatus* Redtenbacher, 1868, by original designation. Status: junior synonym of *Dysantes* Pascoe, 1871 in Tenebrioninae: Toxicini: Dysantina. Note: unnecessary replacement name for *Dysantes* Pascoe, 1871 (see Bouchard and Bousquet 2020a: 101 for additional comments).

*Euglyptonotus* Gestro, 1901: 744 [M]. Type species: *Euglyptonotusmagrettii* Gestro, 1901, by monotypy. Status: valid genus in Tenebrioninae: Amarygmini.

*Euhelaeus* Gebien, 1921a: 281 [M]. Type species: *Euhelaeusspeculiferus* Gebien, 1921, by monotypy. Status: junior synonym of *Emcephalus* W. Kirby, 1828 in Tenebrioninae: Heleini: Heleina. Synonymy: Matthews (1993: 1074).

*Euhemicera* Ando, 1996: 189, 197 [F]. Type species: *Epilampuspulcher* Hope, 1843, by original designation. Status: valid genus in Stenochiinae: Cnodalonini.

*Eulabis* Eschscholtz, 1829: 14 [F]. Type species: *Eulabisbicarinata* Eschscholtz, 1829, by subsequent designation (Blaisdell 1932: 44). Status: valid genus in Tenebrioninae: Eulabini.

*Eulea* Carter, 1937: 131 [F]. Type species: *Euleacaeca* Carter, 1937, by monotypy. Status: valid genus in Lagriinae: Belopini.

*Euleantus* Haag-Rutenberg, 1876: 85 [M]. Type species: *Euleantushumeralis* Haag-Rutenberg, 1876, by monotypy. Status: junior synonym of *Rhammatodes* Haag-Rutenberg, 1876 in Pimeliinae: Tentyriini. Synonymy: Koch (1952a: 133).

*Eulipus* Wollaston, 1864: 448 [M]. Type species: *Tentyriaelongata* Brullé, 1839 (= *Tentyriabrullaei* Wollaston, 1865), by monotypy. Status: valid genus in Pimeliinae: Tentyriini.

*Eulytus* C.O. Waterhouse, 1882a: 175 [M]. Type species: *Eulytusnodipennis* C.O. Waterhouse, 1882, by monotypy. Status: valid genus in Tenebrioninae: Amarygmini.

*Eumicrositus* Viñolas, 1990: 57, 62 [M]. Type species: *Pedinusulissiponensis* Germar, 1823, by original designation. Status: valid subgenus of *Phylan* Sturm, 1826 in Blaptinae: Dendarini: Dendarina. Note: this name was first proposed by Español (1947: 11, 15) without type species designation and is therefore unavailable from that date.

*Eumolpamarygmus* Pic, 1923b: 11 [M]. Type species: *Eumolpamarygmusbigibbosus* Pic, 1923, by monotypy. Status: valid genus in Tenebrioninae: Amarygmini.

*Eumolparamarygmus* Bremer, 2006: 8 [M]. Type species: *Eumolpamarygmusnitidus* Pic, 1935, by original designation. Status: valid genus in Tenebrioninae: Amarygmini. Note: the earlier usage of *Eumolparamarygmus* by Pic (1935: 24) is interpreted as an incorrect subsequent spelling of *Eumolpamarygmus* Pic, 1923 (see Bremer 2005: 210).

*Eumolpocyriogeton* Pic, 1923f: 305 [M]. Type species: *Eumolpocyriogetonconvexus* Pic, 1923 (= *Plesiophthalmusconcameratus* Bremer & Lillig, 2014), by monotypy. Status: valid subgenus of *Plesiophthalmus* Motschulsky, 1857 in Tenebrioninae: Amarygmini.

*Eumylada* Reitter, 1904: 170 [F]. Type species: *Myladinapunctifera* Reitter, 1889, by subsequent designation (Gebien 1939: 461). Status: valid genus in Blaptinae: Opatrini: Opatrina.

*Eunotiodes* Casey, 1907: 519 [M]. Type species: *Eunotiodesbrevicollis* Casey, 1907, by original designation. Status: valid genus in Pimeliinae: Epitragini.

*Euomma* Boheman, 1858: 101 [N]. Type species: *Euommalaterale* Boheman, 1858, by monotypy. Status: valid genus in Alleculinae: Alleculini: Alleculina.

*Euomophlus* Iablokoff-Khnzorian, 1983: 134 [M]. Type species: *Cistelarugosicollis* Brullé, 1832, by original designation. Status: valid subgenus of *Omophlus* Dejean, 1834 in Alleculinae: Cteniopodini.

†*Eupachypterus* Kirejtshuk, Nabozhenko & Nel, 2010: 192 [M]. Type species: *Eupachypteruseocenicus* Kirejtshuk, Nabozhensko & Nel, 2010, by original designation. Status: valid genus in Blaptinae: Opatrini: Neopachypterina. Note: described from Eocene deposits (France).

*Eupezoplonyx* Pic, 1922c: 12 [M]. Type species: *Eupezoplonyxater* Pic, 1922, by monotypy. Status: valid genus in Tenebrioninae: Amarygmini.

*Eupezus* Dejean, 1834: 211 [M]. Type species: *Helopslongipes* Fabricius, 1781, by monotypy. Status: valid genus in Tenebrioninae: Amarygmini.

*Euphloeus* Pascoe, 1887: 15 [M]. Type species: *Euphloeusverrucosus* Pascoe, 1887, by monotypy. Status: valid genus in Stenochiinae: Cnodalonini.

*Euphron* Dejean, 1834: 206 [M]. Type species: *Helopscoerulescens* Guérin-Méneville, 1831, by monotypy. Status: senior synonym of *Derosphaerus* J. Thomson, 1858 in Stenochiinae: Cnodalonini. Synonymy: Bousquet and Bouchard (2013a: 55). Note: nomen oblitum (see Bouchard and Bousquet 2020b: 6).

*Euphrynus* Fairmaire, 1897f: 114 [M]. Type species: *Euphrynusspinithorax* Fairmaire, 1897, by monotypy. Status: valid genus in Pimeliinae: Sepidiini: Molurina.

*Eupomeca* Solier, 1848: 289 [F]. Type species: *Blapscylindrica* Herbst, 1799 (= *Blaps obtusa* Fabricius, 1798), by subsequent designation (Nabozhenko 2008: 35). Status: junior synonym of *Prosodes* Eschscholtz, 1829 in Blaptinae: Blaptini: Prosodina. Synonymy: Gemminger in Gemminger and Harold (1870: 1866).

*Eupsophulus* Cockerell, 1906: 242 [M]. Type species [automatic]: *Eupsophuscastaneus* Horn, 1870, by monotypy. Status: valid genus in Pimeliinae: Vacronini. Note: replacement name for *Eupsophus* Horn, 1870.

*Eupsophus* Horn, 1870: 344, 347 [M]. Type species: *Eupsophuscastaneus* Horn, 1870, by monotypy. Status: senior synonym of *Eupsophulus* Cockerell, 1906 in Pimeliinae: Vacronini. Note: junior homonym of *Eupsophus* Fitzinger, 1843 [Amphibia].

*Eupterocoma* Skopin, 1974b: 152 [F]. Type species: *Pterocomaganglbaueri* Reitter, 1890, by original designation. Status: valid subgenus of *Pterocoma* Dejean, 1834 in Pimeliinae: Pimeliini.

*Eurepipleura* Bogdanov-Katjkov, 1915: 2 [F]. Type species: *Anatolicaminima* Bogdanov-Katjkov, 1915, by monotypy. Status: valid subgenus of *Anatolica* Eschscholtz, 1831 in Pimeliinae: Tentyriini.

*Eurhysodina* Wasmann, 1921: 18 [F]. Type species: *Rhyzodinamarshalli* Blair, 1913, by original designation. Status: valid subgenus of *Rhyzodina* Chevrolat, 1873 in Tenebrioninae: Rhyssopaussini.

*Euryasida* Reitter, 1917a: 40, 58 [F]. Type species: *Asidabarceloi* Pérez Arcas, 1868, by monotypy. Status: junior synonym of *Asida* Latreille, 1802 in Pimeliinae: Asidini. Synonymy: F. Soldati (2008: 34).

*Eurycaulinus* Koch, 1937: 468 [M]. Type species: *Eurycaulusquedenfeldti* Heyden, 1890, by monotypy. Status: valid subgenus of *Eurycaulus* Fairmaire, 1868 in Blaptinae: Opatrini: Sclerina.

*Eurycaulus* Fairmaire, 1868: 492 [M]. Type species: *Eurycaulusmarmottani* Fairmaire, 1868, by monotypy. Status: valid genus and subgenus in Blaptinae: Opatrini: Sclerina.

*Eurychora* Thunberg, 1789: 9 [F]. Type species: *Pimeliaciliata* Fabricius, 1781, by monotypy. Status: valid genus in Pimeliinae: Adelostomini. Note: *Eurychora* is an incorrect subsequent spelling of the original spelling *Evrychora*, first used by Thunberg (1791: 116), in prevailing usage; *Eurychora* is deemed to be the correct original spelling (ICZN 1999, Article 33.3.1).

*Eurychorula* Koch, 1952b: 7 [F]. Type species: *Eurychoraacuminata* Fairmaire, 1891, by original designation. Status: valid genus in Pimeliinae: Adelostomini.

*Eurygona* Laporte, 1840: 187 [F]. Type species: *Aulacuschilensis* Gray, 1832, by monotypy. Status: senior synonym of *Orthogonoderes* Solier, 1841 in Pimeliinae: Praociini. Note: replacement name for *Aulacus* Gray, 1832; junior homonym of *Eurygona* Boisduval, 1836 [Lepidoptera].

*Euryhelops* Reitter, 1902b: 209 [M]. Type species: *Helopschampioni* Reitter, 1891, by monotypy. Status: valid subgenus of *Bioramix* Bates, 1879 in Blaptinae: Platyscelidini.

*Euryhelops* Reitter, 1902b: 214 [M]. Type species: *Euryhelopstiro* Reitter, 1902, by subsequent designation (Gebien 1943: 424). Status: senior synonym of *Zophohelops* Reitter, 1902 in Tenebrioninae: Helopini: Cylindrinotina. Note: junior homonym of *Euryhelops* Reitter, 1902 [Tenebrionidae: Blaptinae: Platyscelidini].

*Eurymetopon* Eschscholtz, 1831: 5, 8 [N]. Type species: *Eurymetoponrufipes* Eschscholtz, 1831, by subsequent designation (Casey 1907: 288). Status: valid genus in Pimeliinae: Edrotini.

*Eurymetopum* Agassiz, 1846b: 151 [N]. Type species [automatic]: *Eurymetoponrufipes* Eschscholtz, 1831, by subsequent designation (Casey 1907: 288). Status: junior synonym of *Eurymetopon* Eschscholtz, 1831 in Pimeliinae: Edrotini. Note: unjustified emendation of *Eurymetopon* Eschscholtz, 1831, not in prevailing usage.

*Eurynotus* W. Kirby, 1819a: 418 [M]. Type species: *Eurynotusmuricatus* W. Kirby, 1819 (= *Helopscapensis* Fabricius, 1794), by monotypy. Status: valid genus and subgenus in Blaptinae: Platynotini: Eurynotina.

*Euryostola* Reitter, 1893: 202, 207 [F]. Type species: *Pachyscelisminor* Baudi di Selve, 1875, by monotypy. Status: valid genus in Pimeliinae: Pimeliini.

*Eurypera* Pascoe, 1870: 106 [F]. Type species: *Euryperacuprea* Pascoe, 1870 (= *Amarygmuspascoei* Gebien, 1911), by monotypy. Status: junior synonym of *Amarygmus* Dalman, 1823 in Tenebrioninae: Amarygmini. Synonymy: Blackburn (1893a: 92).

*Eurypimelia* Reitter, 1915: 9, 49 [F]. Type species: *Tenebriosubglobosus* Pallas, 1781, by subsequent designation (Chernei 2005: 104). Status: junior synonym of *Camphonota* Solier, 1836 in Pimeliinae: Pimeliini. Synonymy: Gebien (1937a: 837).

*Euryprosodes* Reitter, 1909a: 122 [M]. Type species: *Prosodesareolata* Reitter, 1893, by original designation. Status: valid subgenus of *Prosodes* Eschscholtz, 1829 in Blaptinae: Blaptini: Prosodina.

*Euryprosternum* Chatanay, 1914b: 3 [N]. Type species: *Andremiusparallelus* Chatanay, 1913, by monotypy. Status: valid genus in Pimeliinae: Asidini.

*Eusarca* Chevrolat, 1845: 526 [F]. Type species: *Eusarcairidipennis* Chevrolat, 1845 (= *Hegemonaresplendens* Laporte, 1840), by monotypy. Status: junior synonym of *Hegemona* Laporte, 1840 in Stenochiinae: Cnodalonini. Synonymy: Duponchel (1845a: 498). Note: junior homonym of *Eusarca* Hübner, 1813 [Lepidoptera].

*Eusattodes* Casey, 1908: 56, 64 [M]. Type species: *Eusattuslaevis* J.L. LeConte, 1866, by original designation. Status: junior synonym of *Eusattus* J.L. LeConte, 1851 in Pimeliinae: Coniontini. Synonymy: Triplehorn (1968: 379).

*Eusattus* J.L. LeConte, 1851: 131 [M]. Type species: *Eusattusdifficilis* J.L. LeConte, 1851, by subsequent designation (Casey 1908: 56). Status: valid genus in Pimeliinae: Coniontini.

*Euschatia* Solier, 1851: 227 [F]. Type species: *Euschatiaproxima* Solier, 1851, by **present designation**. Status: junior synonym of *Heliofugus* Guérin-Méneville, 1831 in Stenochiinae: Cnodalonini. Synonymy: Lacordaire (1859b: 443).

*Euschides* J.L. LeConte, 1851: 127 [F]. Type species [automatic]: *Stenomorphablapsoides* Solier, 1836, by subsequent designation (Desmarest 1860: 150). Status: junior synonym of *Stenomorpha* Solier, 1836 in Pimeliinae: Asidini. Note: unnecessary replacement name for *Stenomorpha* Solier, 1836.

*Euspinamarygmus* Masumoto, 1989b: 295 [M]. Type species: *Euspinamarygmuskaszabi* Masumoto, 1989, by original designation. Status: valid genus in Tenebrioninae: Amarygmini.

*Eustenia* Fairmaire, 1905: 303 [F]. Type species: *Eusteniatenuimembris* Fairmaire, 1905, by monotypy. Status: senior synonym of *Tucumana* Gebien, 1911 in Alleculinae: Alleculini: Xystropodina. Note: junior homonym of *Eustenia* Snellen, 1899 [Lepidoptera].

*Eustenomacidius* Nabozhenko, 2006: 807 [M]. Type species: *Helopsluridus* Ménétriés, 1849 (= *Stenomaxlaevicollis* Kraatz, 1882), by original designation. Status: valid genus and subgenus in Tenebrioninae: Helopini: Cylindrinotina.

*Eustolopus* Gebien, 1938b: 61 [M]. Type species: *Eustolopuscalcaratus* Gebien, 1938, by original designation. Status: valid genus in Pimeliinae: Adesmiini. Note: the First Reviser (*Eustolopus* Gebien, 1938 versus *Entinopoda* Gebien, 1938) is Penrith (1979: 40).

*Eustrongylium* Kolbe, 1895: 366 [N]. Type species: *Strongyliumepiscopale* Kolbe, 1895, by subsequent designation (Löbl et al. 2008a: 41). Status: junior synonym of *Strongylium* W. Kirby, 1819 in Stenochiinae: Stenochiini. Synonymy: Gebien (1911b: 590).

*Eusyntelia* C.O. Waterhouse, 1881: 473 [F]. Type species: *Eusynteliabalfouri* C.O. Waterhouse, 1881, by subsequent designation (R. Lucas 1920: 290). Status: valid genus in Pimeliinae: Tentyriini.

*Eutagenia* Reitter, 1886: 100, 125 [F]. Type species: *Stenosissmyrnensis* Solier, 1848, by subsequent designation (Iwan et al. 2020: 208). Status: valid genus in Pimeliinae: Stenosini: Stenosina. Note: Reitter (1916: 152) first noted that *Stenosissmyrnensis* Solier of Reitter (1886) was misidentified and proposed the new species *Eutageniacribricollis* for it; the earlier selection of the taxonomic species *Stenosissmyrnensis* Solier sensu Reitter, 1886 (= *Eutageniacribricollis* Reitter, 1916) by Gebien (1937a: 685) as type species is invalid since such an action would have required approval from the International Commission on Zoological Nomenclature at the time; Iwan et al. (2020: 208) fixed the nominal species *Stenosissmyrnensis* Solier, 1848 as the type species following Article 70.3.1 (ICZN 1999).

*Euteleocera* Agassiz, 1846b: 152 [F]. Type species [automatic]: *Euteloceraviatica* Solier, 1841, by original designation. Status: junior synonym of *Eutelocera* Solier, 1841 in Pimeliinae: Praociini. Note: unjustified emendation of *Eutelocera* Solier, 1841, not in prevailing usage.

*Eutelocera* Solier, 1841a: 209, 237 [F]. Type species: *Euteloceraviatica* Solier, 1841, by original designation. Status: valid genus in Pimeliinae: Praociini.

*Eutelogonus* Reitter, 1922a: 24 [M]. Type species: *Helopsdavidis* Fairmaire, 1884, by monotypy. Status: valid subgenus of *Entomogonus* Solier, 1848 in Tenebrioninae: Helopini: Helopina.

*Eutelonodolinus* Robiche, 2007: 450 [M]. Type species: *Eutelonodolinusjolyi* Robiche, 2007, by monotypy. Status: valid genus in Stenochiinae: Cnodalonini.

*Eutelonotus* Fairmaire in Alluaud, 1902: 456 [M]. Type species [automatic]: *Eutelusrequieni* Solier, 1843, by subsequent designation (R. Lucas 1920: 291). Status: valid genus in Stenochiinae: Cnodalonini. Note: replacement name for *Eutelus* Solier, 1843 (see Bousquet 2016a: 44).

*Eutelus* Solier, 1843: 56 [M]. Type species: *Eutelusrequieni* Solier, 1843, by subsequent designation (R. Lucas 1920: 291). Status: senior synonym of *Eutelonotus* Fairmaire in Alluaud, 1902 in Stenochiinae: Cnodalonini. Note: we act as First Revisers and reject the alternative original spelling *Lutelus*, used by Solier (1843: 4); junior homonym of *Eutelus* Walker, 1834 [Hymenoptera].

*Eutermicola* Lea, 1916: 273 [M]. Type species: *Eutermicolasculpticollis* Lea, 1916, by monotypy. Status: valid genus in Tenebrioninae: Palorini.

*Eutherama* Carter, 1914c: 405 [N]. Type species: *Eutheramacyaneum* Carter, 1914, by monotypy. Status: valid genus in Stenochiinae: Stenochiini.

*Euthriptera* Reitter, 1893: 203, 229 [F]. Type species: *Thripteragrisescens* Fairmaire, 1875, by monotypy. Status: valid genus in Pimeliinae: Pimeliini. Note: we act as First Revisers and reject the alternative original spelling *Enthriptera*, used by Reitter (1893: 203).

*Euthysternum* Chatanay, 1915a: 508 [N]. Type species: *Chemolanusepiscopalis* Fairmaire, 1886, by original designation. Status: valid genus in Stenochiinae: Cnodalonini.

*Eutichus* Haag-Rutenberg, 1875b: 4, 58 [M]. Type species: *Eutichuswahlbergi* Haag-Rutenberg, 1875, by monotypy. Status: valid genus in Pimeliinae: Adelostomini.

*Eutochia* J.L. LeConte, 1862: 238 [F]. Type species [automatic]: *Ulomapiceum* Melsheimer, 1846, by monotypy. Status: valid genus in Tenebrioninae: Ulomini. Note: replacement name for *Aniara* Melsheimer, 1853.

*Eutomus* Lacordaire, 1865: 369 [M]. Type species: *Eutomusmicrographus* Lacordaire, 1865, by subsequent designation (Barber 1914: 191). Status: junior synonym of *Rhipidandrus* J.L. LeConte, 1862 in Tenebrioninae: Bolitophagini. Synonymy: J.L. LeConte and Horn (1883: 232). Note: originally described as a member of Curculionoidea: Curculionidae: Scolytinae.

*Eutoreuma* Carter, 1914b: 78 [N]. Type species [automatic]: *Toreumacupreum* Carter, 1913, by monotypy. Status: senior synonym of *Atoreuma* Gebien, 1941 in Tenebrioninae: Heleini: Cyphaleina. Note: replacement name for *Toreuma* Carter, 1913; junior homonym of *Eutoreuma* Grote, 1872 [Lepidoptera].

*Eutrapela* Dejean, 1834: 215 [F]. Type species: *Crioceriselongata* Fabricius, 1781 (= *Chrysomelaunifasciata* DeGeer, 1778), by subsequent designation (Duponchel 1845a: 533). Status: senior synonym of *Neoeutrapela* Bousquet & Bouchard, 2013 in Lagriinae: Lagriini: Statirina. Synonymy: Bousquet and Bouchard (2013a: 61). Note: junior homonym of *Eutrapela* Hübner, 1809 [Lepidoptera].

*Eutrapelodes* Borchmann, 1929b: 132 [F]. Type species: *Cteniopusgracillimus* Fairmaire, 1888, by original designation. Status: valid genus in Alleculinae: incertae sedis.

*Eutriorophus* Casey, 1924: 296 [M]. Type species: *Eutriorophustuckeri* Casey, 1924, by original designation. Status: junior synonym of *Stibia* Horn, 1870 in Pimeliinae: Edrotini. Synonymy: Blaisdell (1933: 210).

*Euzadenos* Koch, 1956a: 286 [M]. Type species: *Eurynotusdelalandii* Mulsant & Rey, 1854, by original designation. Status: junior synonym of *Selenepistoma* Dejean, 1834 in Blaptinae: Dendarini: Melambiina. Synonymy: **new synonym** [PB]. Note: *Euzadenos* Koch, 1956 was recently treated as a valid subgenus of *Zadenos* Laporte, 1840 (Kamiński 2015: 549); however, the type species of the older available genus name *Selenepistoma* Dejean, 1834 is currently placed in *Euzadenos* and therefore *Selenepistoma* has priority.

*Evaniosomus* Guérin-Méneville, 1834: 14 [M]. Type species: *Evaniosomusorbignianus* Guérin-Méneville, 1834, by monotypy. Status: valid genus in Pimeliinae: Evaniosomini.

*Evaostetha* Novák, 2008a: 208 [F]. Type species: *Evaostethapetri* Novák, 2008, by original designation. Status: valid genus in Alleculinae: Alleculini: Alleculina.

*Evelina* J. Thomson, 1860c: 22 [F]. Type species: *Evelinalacordairei* J. Thomson, 1860, by monotypy. Status: valid genus in Pimeliinae: Evaniosomini.

*Eviropodus* Koch, 1956a: 84 [M]. Type species: *Trigonopusalternans* Fåhraeus, 1870, by original designation. Status: valid genus in Blaptinae: Platynotini: Platynotina.

*Evoplus* J.L. LeConte, 1866b: 128 [M]. Type species: *Evoplusferrugineus* J.L. LeConte, 1866, by monotypy. Status: junior synonym of *Neomida* Latreille, 1829 in Diaperinae: Diaperini: Diaperina. Synonymy: Champion (1886: 175, with *Arrhenoplita* W. Kirby, 1837, a junior synonym of *Neomida* Latreille, 1829).

*Exadelium* Watt, 1992: 29 [N]. Type species: *Adeliumrufilabrum* Broun, 1886, by original designation. Status: valid genus in Lagriinae: Adeliini.

*Exangeltus* Blackburn, 1897b: 93 [M]. Type species: *Exangeltusangustus* Blackburn, 1897, by monotypy. Status: valid genus in Pimeliinae: Vacronini.

*Exapinaeus* Pascoe, 1882: 34 [M]. Type species: *Exapinaeuspolitus* Pascoe, 1882, by monotypy. Status: valid genus in Diaperinae: Diaperini: Diaperina.

*Exechophthalmus* Ardoin, 1974a: 168 [M]. Type species: *Exechophthalmusguillaumeti* Ardoin, 1974, by monotypy. Status: valid genus in Phrenapatinae: Penetini.

*Exerestus* Bates, 1870: 268 [M]. Type species: *Exerestusjansonii* Bates, 1870 (= *Rhinandruselongatus* Horn, 1866), by monotypy. Status: junior synonym of *Rhinandrus* J.L. LeConte, 1866 in Tenebrioninae: Tenebrionini. Synonymy: Bates (1872a: 98).

*Exeniotis* Pascoe, 1871: 353 [F]. Type species: *Exeniotiscollaris* Pascoe, 1871, by monotypy. Status: valid genus in Lagriinae: Belopini.

*Exocolena* Gebien, 1914c: 43 [F]. Type species: *Exocolenalongipes* Gebien, 1914, by monotypy. Status: valid genus in Stenochiinae: Cnodalonini.

*Exostira* Borchmann, 1925: 353 [F]. Type species: *Exostirasellata* Borchmann, 1925, by subsequent designation (Borchmann 1930a: 512). Status: valid genus in Lagriinae: Lagriini: Statirina.

*Extetranosis* Koch, 1940b: 741 [F]. Type species: *Tetranosisethasicornis* Koch, 1940, by monotypy. Status: valid subgenus of *Microtelopsis* Koch, 1940 in Pimeliinae: Stenosini: Stenosina. Note: we act as First Revisers and select *Microtelopsis* Koch, 1940 as the valid name for this genus instead of *Extetranosis* Koch, 1940 and *Hypermicrotelopsis* Koch, 1940.

*Fahraeus* Ardoin, 1963b: 309, 349 [M]. Type species: *Oplocheiruspunctatissimus* Fåhraeus, 1870, by monotypy. Status: valid genus in Tenebrioninae: Amarygmini.

*Falacer* Laporte, 1840: 233 [M]. Type species: *Accanthopuscupreus* Laporte, 1840 (= *Helopscontractus* Palisot de Beauvois, 1811), by subsequent designation (Bousquet et al. 2018: 141). Status: junior synonym of *Meracantha* W. Kirby, 1837 in Tenebrioninae: Amarygmini. Synonymy: Lacordaire (1859b: 466).

*Falsammidium* Koch, 1960: 395, 405 [N]. Type species: *Clitobiuslaevipennis* Fairmaire, 1892, by original designation. Status: valid genus in Blaptinae: Opatrini: Ammobiina.

*Falsandrosus* Kaszab, 1980b: 307 [M]. Type species: *Falsandrosustetrops* Kaszab, 1980, by original designation. Status: valid genus in Stenochiinae: Cnodalonini. Note: *Faslandrosus* was introduced earlier by Kaszab (1979a: 104) but the name is unavailable from that date since it was published after 1930 without a description, a definition or a bibliographic reference to such a published statement (ICZN 1999, Article 13.1).

*Falsaspila* Koch, 1952b: 28 [F]. Type species: *Adelostomabatesi* Haag-Rutenberg, 1875, by original designation. Status: junior synonym of *Zarudnionymus* Semenov-Tjan-Shansky & Bogatchev, 1947 in Pimeliinae: Adelostomini. Synonymy: Ardoin (1976: 149).

*Falsastenochirus* Pic, 1938: 13 [M]. Type species: *Asthenochiruscontractus* Fairmaire, 1894, by monotypy. Status: valid genus in Tenebrioninae: Amarygmini.

*Falsasthenochirus* Ardoin, 1965a: 637 [M]. Type species [automatic]: *Asthenochiruscontractus* Fairmaire, 1894, by monotypy. Status: junior synonym of *Falsastenochirus* Pic, 1938 in Tenebrioninae: Amarygmini. Note: unjustified emendation of *Falsastenochirus* Pic, 1938, not in prevailing usage.

*Falsoarthroconus* Kaszab, 1978b: 52, 57 [M]. Type species: *Falsoarthroconusnocturnus* Kaszab, 1978, by original designation. Status: valid genus in Pimeliinae: Edrotini.

*Falsoaugolesthus* Masumoto, 1993c: 38 [M]. Type species: *Eucyrtuspulcher* Pic, 1927, by original designation. Status: junior synonym of *Tetragonomenes* Chevrolat, 1878 in Stenochiinae: Cnodalonini. Synonymy: Ando et al. (2016: 65).

*Falsobates* Kaszab, 1941a: 5, 27 [M]. Type species: *Falsobatesxantusi* Kaszab, 1941, by original designation. Status: valid genus in Stenochiinae: Cnodalonini.

*Falsobrachys* Kulzer, 1954a: 65 [M]. Type species: *Falsobrachyslongipes* Kulzer, 1954, by original designation. Status: valid genus in Stenochiinae: Cnodalonini.

*Falsocaedius* Español, 1943: 140, 142 [M]. Type species: *Clitobiusfossulatus* Escalera, 1914, by monotypy. Status: valid genus in Blaptinae: Opatrini: Ammobiina.

*Falsocalcar* Pic, 1925: 9 [N]. Type species: *Falsocalcarbicolor* Pic, 1925, by monotypy. Status: valid genus in Tenebrioninae: Tenebrionini.

*Falsocamaria* Pic, 1917g: 19 [F]. Type species: *Falsocamariaobscura* Pic, 1917, by monotypy. Status: valid genus in Stenochiinae: Cnodalonini.

*Falsocamariodes* Ardoin, 1956a: 159 [M]. Type species: *Falsocamariodesviettei* Ardoin, 1956, by original designation. Status: valid genus in Stenochiinae: Cnodalonini.

*Falsocasnonidea* Pic, 1934a: 31 [F]. Type species: *Nemostiradiversipennis* Pic, 1923, by monotypy. Status: junior synonym of *Casnonidea* Fairmaire, 1882 in Lagriinae: Lagriini: Statirina. Synonymy: Borchmann (1936: 393).

*Falsocatomulus* Pic, 1914c: 10 [M]. Type species: *Falsocatomuluseuphraticus* Pic, 1914, by monotypy. Status: valid genus in Pimeliinae: Tentyriini.

*Falsocosmonota* Kaszab, 1962b: 75 [F]. Type species: *Falsocosmonotacheni* Kaszab, 1962, by original designation. Status: valid genus in Diaperinae: Diaperini: Diaperina.

*Falsocossyphus* Pic, 1916b: 4 [M]. Type species: *Falsocossyphuspilosus* Pic, 1916, by monotypy. Status: valid genus in Tenebrioninae: Falsocossyphini.

*Falsocuphotes* Pic, 1918b: 22 [F]. Type species: *Falsocuphotescurticornis* Pic, 1918, by monotypy. Status: valid genus in Stenochiinae: Stenochiini.

*Falsodiopethes* Pic, 1924b: 12 [M]. Type species: *Falsodiopethesgounellei* Pic, 1924, by monotypy. Status: valid genus in Stenochiinae: Cnodalonini.

*Falsoencyalesthus* Pic, 1923c: 29 [M]. Type species: *Falsoencyalesthuslatipennis* Pic, 1923, by monotypy. Status: junior synonym of *Derosphaerus* J. Thomson, 1858 in Stenochiinae: Cnodalonini. Synonymy: Ardoin (1969b: 125, with *Encyalesthus* Motschulsky, 1860, a junior synonym of *Derosphaerus* J. Thomson, 1858).

*Falsogauromaia* Pic, 1921d: 22 [F]. Type species: *Falsogauromaiaannulipes* Pic, 1921, by monotypy. Status: valid subgenus of *Gauromaia* Pascoe, 1866 in Stenochiinae: Cnodalonini.

*Falsolagria* Pic, 1927a: 44 [F]. Type species: *Falsolagriamarmorata* Pic, 1927, by monotypy. Status: valid genus in Lagriinae: Lagriini: Lagriina.

*Falsolobodera* Kaszab, 1967: 24 [F]. Type species: *Falsoloboderaskopini* Kaszab, 1967, by original designation. Status: valid genus in Blaptinae: Opatrini: Opatrina.

*Falsolophocnemis* Pic, 1917d: 13 [F]. Type species: *Falsolophocnemissinuatipes* Pic, 1917, by subsequent designation (Gebien 1948: 542). Status: junior synonym of *Strongylium* W. Kirby, 1819 in Stenochiinae: Stenochiini. Synonymy: Kaszab (1977b: 29).

*Falsomicrodera* Kaszab, 1966: 291, 294 [F]. Type species: *Microderaturkestanica* Schuster, 1915, by original designation. Status: valid subgenus of *Microdera* Eschscholtz, 1831 in Pimeliinae: Tentyriini.

*Falsomophlus* Pic, 1925c: 11 [M]. Type species: *Falsomophlusniger* Pic, 1925, by monotypy. Status: valid genus in Alleculinae: Cteniopodini.

*Falsomycterus* Pic, 1907a: 127 [M]. Type species: *Falsomycterusdiversipes* Pic, 1907, by monotypy. Status: valid genus in Pimeliinae: Falsomycterini.

*Falsonannocerus* Pic, 1947: 150 [M]. Type species: *Falsonannocerusdentaticeps* Pic, 1947, by monotypy. Status: valid genus in Stenochiinae: Cnodalonini.

*Falsonemostira* Pic, 1917c: 14 [F]. Type species: *Nemostiraannulipes* Pic, 1912, by monotypy. Status: valid genus in Lagriinae: Lagriini: Statirina.

*Falsonotostrongylium* Kaszab, 1955a: 538, 552 [N]. Type species: *Falsonotostrongyliumbradymeroides* Kaszab, 1955, by original designation. Status: valid genus in Stenochiinae: Stenochiini.

*Falsoperichilus* Pic, 1920a: 16 [M]. Type species: *Falsoperichilussemirugosus* Pic, 1920, by monotypy. Status: valid genus in Stenochiinae: Cnodalonini.

*Falsophthora* Kaszab, 1977a: 306, 309 [F]. Type species: *Tagalusfordi* Kulzer, 1957, by monotypy. Status: valid subgenus of *Pseudophthora* Kaszab, 1970 in Phrenapatinae: Penetini.

*Falsoplonyx* Ardoin, 1963b: 308, 337 [M]. Type species: *Gonocnemisrubripes* Fairmaire, 1899, by original designation. Status: valid genus in Tenebrioninae: Amarygmini.

*Falsopraocis* Kulzer, 1958a: 5, 102 [M]. Type species: *Amphidoraricardae* (as “*richardae*”) Solier, 1851, by original designation. Status: valid genus in Pimeliinae: Praociini.

*Falsopsilonycha* Pic, 1931c: 303 [F]. Type species: *Psilonychausambarana* Pic, 1917, by original designation. Status: valid genus in Alleculinae: incertae sedis.

*Falsostrongylium* Pic, 1915: 11 [N]. Type species: *Falsostrongyliumsemirufum* Pic, 1915, by subsequent designation (Gebien 1948: 539). Status: valid genus in Stenochiinae: Stenochiini.

*Falsosynopticus* Pic, 1936b: 17 [M]. Type species: *Falsosynopticusater* Pic, 1936, by monotypy. Status: valid genus in Tenebrioninae: Amarygmini.

*Falsotagalus* Kaszab, 1977a: 301, 310 [M]. Type species: *Falsotagalussubcoecus* Kaszab, 1977, by original designation. Status: valid genus in Phrenapatinae: Penetini.

*Falsotithassa* Pic, 1934b: 18 [F]. Type species: *Falsotithassasumatrana* Pic, 1934, by monotypy. Status: valid genus in Lagriinae: Lupropini.

*Falsozialeus* Pic, 1951: 11 [M]. Type species: *Falsozialeusater* Pic, 1951 (= *Asyleptusfumosus* Péringuey, 1896), by monotypy. Status: junior synonym of *Asyleptus* Péringuey, 1896 in Tenebrioninae: Amarygmini. Synonymy: Ardoin (1962b: 957); Schawaller and Bremer (2013: 81).

*Falsozotypus* Kaszab, 1980b: 334 [M]. Type species: *Falsozotypusopacipennis* Kaszab, 1980, by original designation. Status: valid genus in Stenochiinae: Cnodalonini. Note: this name was first published by Kaszab (1979a: 108) without a description, a definition, or a bibliographic reference to such a published statement (ICZN 1999, Article (13.1) and is therefore unavailable from that date.

*Farsarthrosis* Kaszab, 1979a: 86 [F]. Type species: *Farsarthrosisbenardi* Kaszab, 1979, by original designation. Status: valid genus in Pimeliinae: Erodiini.

*Faustia* Kraatz, 1882: 92 [F]. Type species: *Faustiamodesta* Kraatz, 1882, by monotypy. Status: valid subgenus of *Bioramix* Bates, 1879 in Blaptinae: Platyscelidini.

*Ferganoprosodes* G.S. Medvedev, 1997: 595 [M]. Type species: *Prosodesangulicollis* Kraatz, 1883, by original designation. Status: valid subgenus of *Prosodes* Eschscholtz, 1829 in Blaptinae: Blaptini: Prosodina.

*Ferveoventer* Smith, 2013: 604 [M]. Type species: *Ferveoventerbrowni* Smith, 2013, by original designation. Status: valid genus in Pimeliinae: Asidini.

*Fifina* Novák, 2018d: 470 [F]. Type species: *Fifinaromani* Novák, 2018, by original designation. Status: valid genus in Alleculinae: Alleculini: Alleculina.

*Fifinoides* Novák, 2020h: 78 [M]. Type species: *Fifinoideschinensis* Novák, 2020, by original designation. Status: valid genus in Alleculinae: Alleculini: Alleculina.

*Filotarsus* Solier, 1841a: 209, 239 [M]. Type species: *Filotarsustenuicornis* Solier, 1841, by original designation. Status: valid subgenus of *Praocis* Eschscholtz, 1829 in Pimeliinae: Praociini.

*Fitzsimonsia* Koch, 1955b: 415 [F]. Type species: *Fitzsimonsiacymbium* Koch, 1955, by original designation. Status: senior synonym of *Fitzsimonsium* Koch, 1962 in Pimeliinae: Stenosini: Platamodina. Note: junior homonym of *Fitzsimonsia* Witte, 1943 [Reptilia].

*Fitzsimonsium* Koch, 1962b: 152 [N]. Type species [automatic]: *Fitzsimonsiacymbium* Koch, 1955, by original designation. Status: valid genus in Pimeliinae: Stenosini: Platamodina. Note: replacement name for *Fitzsimonsia* Koch, 1955.

*Flabellalogista* Pic, 1954: 256 [F]. Type species: *Flabellalogistaminuta* Pic, 1954, by monotypy. Status: valid genus in Alleculinae: incertae sedis.

*Flabellolagria* Pic, 1927c: 27 [F]. Type species: *Flabellolagrialuteovittata* Pic, 1927, by monotypy. Status: valid genus in Lagriinae: Lagriini: Lagriina.

*Flabellostrongylium* Pic, 1938: 18 [N]. Type species: *Flabellostrongyliumatronitidum* Pic, 1938, by monotypy. Status: valid genus in Stenochiinae: Stenochiini.

*Flavipoda* Campbell, 1966: 21 [F]. Type species: *Helopsflavipes* Fabricius, 1792 [as “*Alleculaflavipes* Jacquelin du Val, 1857”], by original designation. Status: valid subgenus of *Lobopoda* Solier, 1835 in Alleculinae: Alleculini: Alleculina.

*Foleya* Peyerimhoff, 1916: 71 [F]. Type species: *Foleyabrevicornis* Peyerimhoff, 1916, by monotypy. Status: valid genus in Pimeliinae: Erodiini.

*Foochounus* Pic, 1921d: 22 [M]. Type species: *Foochounusconvexipennis* Pic, 1921, by monotypy. Status: valid genus in Stenochiinae: Cnodalonini.

*Foranotum* Nabozhenko & Sadeghi, 2017: 165 [N]. Type species: *Foranotumperforatum* Nabozhenko & Sadeghi, 2017, by original designation. Status: valid genus in Kuhitangiinae: Foranotini.

*Fossilochile* Koch, 1952c: 63 [F]. Type species: *Fossilochilerufa* Koch, 1952, by original designation. Status: junior synonym of *Pachynotelus* Solier, 1841 in Pimeliinae: Cryptochilini: Cryptochilina. Synonymy: Penrith and Endrödy-Younga (1994: 54).

*Fourtaus* Pic in Alfieri, 1921: 47 [M]. Type species: *Fourtausbrevicornis* Pic, 1921 (= *Hegeterocaraarabicum* Reitter, 1900), by monotypy. Status: junior synonym of *Hegeterocara* Reitter, 1900 in Pimeliinae: Tentyriini. Synonymy: Koch (1941: 294).

*Foveostatira* Pic, 1918b: 24 [F]. Type species: *Statirafoveicollis* Pic, 1918, by subsequent designation (Borchmann 1936: 244). Status: valid subgenus of *Statira* Lepeletier & Audinet-Serville, 1828 in Lagriinae: Lagriini: Statirina.

*Freudeia* Kaszab, 1961b: 216 [F]. Type species: *Freudeianepalica* Kaszab, 1961, by original designation. Status: valid genus in Pimeliinae: Tentyriini.

*Freudella* Ardoin, 1961b: 30 [F]. Type species: *Freudellaauripunctata* Ardoin, 1961, by original designation. Status: valid genus in Stenochiinae: Stenochiini.

*Freyitia* Koch, 1943a: 522, 525 [F]. Type species: *Freyitiaornatipes* Koch, 1943, by monotypy. Status: valid genus in Pimeliinae: Tentyriini.

*Freyula* Koch, 1959: 591, 593 [F]. Type species: *Freyulapsammarina* Koch, 1959, by original designation. Status: valid genus in Blaptinae: Opatrini: Ammobiina.

*Fundulus* Gistel, 1848a: 125 [M]. Type species [automatic]: *Opatrumtibiale* Fabricius, 1781, by monotypy. Status: junior synonym of *Melanimon* Steven, 1828 in Tenebrioninae: Melanimonini. Note: unnecessary replacement name for *Microzoum* Dejean, 1834; junior homonym of *Fundulus* Lacepède, 1803 [Pisces].

*Gabonia* Fairmaire, 1894e: 326 [F]. Type species: *Gaboniadenticulata* Fairmaire, 1894, by monotypy. Status: senior synonym of *Gabonisca* Fairmaire, 1894 in Lagriinae: Pycnocerini. Note: junior homonym of *Gabonia* Jacoby, 1893 [Coleoptera: Chrysomelidae].

*Gabonisca* Fairmaire, 1894f: 395 [F]. Type species [automatic]: *Gaboniadenticulata* Fairmaire, 1894, by monotypy. Status: valid genus in Lagriinae: Pycnocerini. Note: replacement name for *Gabonia* Fairmaire, 1894.

*Gahanosis* Penrith, 1983: 371, 379 [F]. Type species: *Zophosisundulata* Gahan, 1900, by original designation. Status: valid subgenus of *Zophosis* Latreille, 1802 in Pimeliinae: Zophosini.

*Gamaxus* Bates, 1868: 315 [M]. Type species: *Gamaxushauxwellii* Bates, 1868, by monotypy. Status: valid genus in Lagriinae: Goniaderini. Note: combined description of a new genus and single new species (ICZN 1999, Article 12.2.6).

*Garambanus* Ardoin, 1964a: 847 [M]. Type species: *Garambanusverschureni* Ardoin, 1964, by monotypy. Status: valid genus in Tenebrioninae: Amarygmini.

*Gargilius* Fairmaire, 1891b: 251 [M]. Type species: *Gargiliustrispinosus* Fairmaire, 1891, by subsequent designation (Gebien 1940: 421). Status: valid genus in Diaperinae: Diaperini: Diaperina.

*Garridoa* Marcuzzi, 1985: 180 [F]. Type species: *Garridoakaszabi* Marcuzzi, 1985, by monotypy. Status: valid genus in Pimeliinae: Edrotini.

*Gastrhaema* Jacquelin du Val, 1863: 353 [N]. Type species: *Cistelarufiventris* Waltl, 1835, by subsequent designation (Novák and Pettersson 2008: 331). Status: valid genus in Alleculinae: Cteniopodini.

*Gaurobates* Gebien, 1928: 170, 184 [M]. Type species: *Gaurobatespictus* Gebien, 1928, by monotypy. Status: valid genus in Stenochiinae: Cnodalonini.

*Gauromaia* Pascoe, 1866a: 473 [F]. Type species: *Gauromaiadives* Pascoe, 1866, by monotypy. Status: valid genus and subgenus in Stenochiinae: Cnodalonini.

*Gebienella* Kaszab, 1941a: 4, 21 [F]. Type species: *Gebienellainterrumpens* Kaszab, 1941, by original designation. Status: valid genus in Stenochiinae: Cnodalonini.

*Gebienia* Borchmann, 1921: 219, 352 [F]. Type species: *Gebieniarimulosa* Borchmann, 1921, by original designation. Status: valid genus in Lagriinae: Lagriini: Statirina.

*Gebieniella* Koch, 1940b: 736 [F]. Type species: *Ethasstenosides* Pascoe, 1862, by original designation. Status: valid genus in Pimeliinae: Stenosini: Stenosina.

*Gebienocamaria* Masumoto, 1993b: 223, 232 [F]. Type species: *Camariaangulicollis* Fairmaire, 1896, by original designation. Status: valid genus in Stenochiinae: Cnodalonini.

*Gebleria* Motschulsky, 1846: 410 [F]. Type species: *Dilaphilacoides* Fischer von Waldheim, 1844, by monotypy. Status: valid subgenus of *Prosodes* Eschscholtz, 1829 in Blaptinae: Blaptini: Prosodina.

*Gedeon* Reiche & Saulcy, 1857: 219 [M]. Type species: *Gedeonhierichonticus* Reiche & Saulcy, 1857, by monotypy. Status: valid genus in Pimeliinae: Pimeliini.

*Gedrosia* Bogatchev, 1961: 111 [F]. Type species: *Gedrosiamonstrosa* Bogatchev, 1961 (= *Pseudopodhomalagabrieli* Schuster, 1938), by original designation. Status: junior synonym of *Pseudopodhomala* Schuster, 1938 in Pimeliinae: Pimeliini. Synonymy: Löbl et al. (2008b: 166). Note: junior homonym of *Gedrosia* Stål, 1862 [Hemiptera].

*Genateropa* Bouchard & Bousquet, **new replacement name** [F]. Type species [automatic]: *Apterogenacanonnei* Ardoin, 1962, by original designation. Status: valid genus in Stenochiinae: Stenochiini. Note: replacement name for *Apterogena* Ardoin, 1962.

*Genoblaps* Bauer, 1921: 230 [F]. Type species: *Blaps tentyroides* Seidlitz, 1893 (= *Blapssocia* Seidlitz, 1893), by monotypy. Status: valid subgenus of *Blaps* Fabricius, 1775 in Blaptinae: Blaptini: Blaptina.

*Gentinadis* Laporte, 1840: 240 [M]. Type species: *Stenochiacaerulea* Laporte, 1840 (= *Strongyliumazureum* Germar, 1823), by monotypy. Status: junior synonym of *Strongylium* W. Kirby, 1819 in Stenochiinae: Stenochiini. Synonymy: Lacordaire (1859b: 484).

*Geoborus* Blanchard, 1842: pl. 13 [M]. Type species: *Geoboruscostatus* Blanchard, 1842 (= *Gyriosomuslineatus* Guérin-Méneville, 1834), by subsequent designation (Pizarro-Araya and Flores 2006: 95). Status: valid genus in Pimeliinae: Epitragini.

*Geophanus* Haag-Rutenberg, 1875b: 4, 46 [M]. Type species: *Psaryphisconfusa* Fåhraeus, 1870, by subsequent designation (Gebien 1937a: 675). Status: valid genus in Pimeliinae: Adelostomini.

*Gerandryus* Rottenberg, 1873: 217 [M]. Type species [automatic]: *Parablopsaetnensis* Rottenberg, 1871, by monotypy. Status: valid genus in Alleculinae: Alleculini: Gonoderina. Note: replacement name for *Parablops* Rottenberg, 1871.

*Gerardia* Pic, 1954: 258 [F]. Type species: *Gerardiasublineata* Pic, 1954, by monotypy. Status: senior synonym of *Piccula* Bousquet & Bouchard, 2015 in Alleculinae: Alleculini: Gonoderina. Note: junior homonym of *Gerardia* Lacaze-Duthiers, 1864 [Cnidaria].

*Gerdacula* Novák, 2015b: 145 [F]. Type species: *Gerdaculafujianica* Novák, 2015, by original designation. Status: valid genus in Alleculinae: Alleculini: Alleculina.

*Ghaleca* Péringuey, 1899: 316 [F]. Type species: *Ghalecalaeta* Péringuey, 1899 (= *Alymonprolatus* Pascoe, 1866), by monotypy. Status: junior synonym of *Alymon* Pascoe, 1866 in Tenebrioninae: Amarygmini. Synonymy: Péringuey (1904: 297, through synonymy of its type species with *Alymonprolatus* Pascoe, 1866).

*Gibbostrongylium* Pic, 1917d: 18 [N]. Type species: *Strongyliummedanense* Pic, 1917, by subsequent designation (Löbl et al. 2008a: 41). Status: junior synonym of *Strongylium* W. Kirby, 1819 in Stenochiinae: Stenochiini. Synonymy: Gebien (1948: 519).

*Gigantopigeus* Kaszab, 1984: 355, 374 [M]. Type species: *Gigantopigeusmirabilis* Kaszab, 1984, by original designation. Status: valid genus in Stenochiinae: Cnodalonini.

*Girardius* L. Soldati, 2009: 73 [M]. Type species: *Micipsapersica* Baudi, 1874, by original designation. Status: valid genus in Pimeliinae: Tentyriini.

*Girardocamaria* Masumoto, 1993b: 227, 232 [F]. Type species: *Girardocamariaardoini* Masumoto, 1993, by original designation. Status: valid genus in Stenochiinae: Cnodalonini.

*Glabrasida* Escalera, 1910: 408 [F]. Type species: *Glabrasidaconspuata* Escalera, 1910, by subsequent designation (F. Soldati 2008: 32). Status: valid subgenus of *Alphasida* Escalera, 1905 in Pimeliinae: Asidini.

*Glabrilobopoda* Campbell, 1966: 46 [F]. Type species: *Lobopodaglabrata* Champion, 1888, by original designation. Status: valid subgenus of *Lobopoda* Solier, 1835 in Alleculinae: Alleculini: Alleculina.

*Globasida* Escalera, 1905b: 430 [F]. Type species: *Asidaoblonga* Rambur, 1838, by subsequent designation (Viñolas and Cartagena 2005: 209). Status: valid subgenus of *Asida* Latreille, 1802 in Pimeliinae: Asidini.

*Globularthrodosis* Kaszab, 1979a: 95 [F]. Type species: *Diaphanidussemenowi* Reitter, 1900, by original designation. Status: junior synonym of *Diaphanidus* Reitter, 1900 in Pimeliinae: Erodiini. Synonymy: G.S. Medvedev and Nepesova (1985: 52).

*Glyptariobius* Koch, 1948: 423 [M]. Type species: *Hoplariobiusexacavatus* Koch, 1948, by original designation. Status: valid subgenus of *Hoplarion* Mulsant & Rey, 1854 in Blaptinae: Dendarini: Melambiina.

*Glyptasida* Casey, 1912: 75, 95 [F]. Type species: *Ophryastessordidus* J.L. LeConte, 1853, by original designation. Status: valid subgenus of *Philolithus* Lacordaire, 1858 in Pimeliinae: Asidini.

*Glyptophrynus* Fairmaire, 1899e: 532 [M]. Type species: *Glyptophrynustenuesculptus* Fairmaire, 1899, by monotypy. Status: valid genus in Pimeliinae: Sepidiini: Molurina.

*Glyptopteryx* Gebien, 1910c: 376 [F]. Type species: *Glyptopteryxforticostis* Gebien, 1910 (= *Selinusquadricollis* Fairmaire, 1887), by monotypy. Status: valid genus in Blaptinae: Platynotini: Platynotina.

*Glyptothorax* Borchmann, 1937: 223 [M]. Type species: *Glyptothoraxpilosus* Borchmann, 1937, by original designation. Status: senior synonym of *Borchmannius* Bousquet & Bouchard, 2015 in Alleculinae: incertae sedis. Note: junior homonym of *Glyptothorax* Blyth, 1860 [Pisces]; taxon also described as new by Borchmann (1938: 122).

*Glyptotus* J.L. LeConte, 1858a: 75 [M]. Type species: *Glyptotuscribratus* J.L. LeConte, 1858, by monotypy. Status: valid genus in Stenochiinae: Cnodalonini.

*Gnaptor* Brullé, 1831: 254 [M]. Type species: *Tenebriospinimanus* Pallas, 1781, by monotypy. Status: valid genus and subgenus in Blaptinae: Blaptini: Gnaptorina. Note: this name was previously attributed to Brullé (1832: 202 as *Petrobius*, corrected to *Gnaptor* in the “Errata” of the same work) in the literature.

*Gnaptorina* Reitter, 1887a: 364 [F]. Type species: *Gnaptorinafelicitana* Reitter, 1887, by monotypy. Status: valid genus and subgenus in Blaptinae: Blaptini: Gnaptorinina.

*Gnathelops* Gebien, 1922b: 320 [M]. Type species: *Gnathelopschatanayi* Gebien, 1922, by monotypy. Status: valid genus in Tenebrionidae: incertae sedis. Note: removed from the tribe Helopini and placed as Tenebrionidae incertae sedis by Nabozhenko (2018: 183).

*Gnathidium* Gebien, 1921b: 41 [N]. Type species: *Gnathidiumcephalotes* Gebien, 1921, by monotypy. Status: valid genus in Diaperinae: Gnathidiini: Gnathidiina.

*Gnathocerus* Agassiz, 1846b: 164 [M]. Type species [automatic]: *Gnatocerusruber* Thunberg, 1814 (= *Trogossitacornuta* Fabricius, 1798), by monotypy (see ICZN 1975, Opinion 1039). Status: junior synonym of *Gnatocerus* Thunberg, 1814 in Diaperinae: Diaperini: Adelinina. Note: unjustified emendation of *Gnatocerus* Thunberg, 1814, not in prevailing usage.

*Gnathosia* Fischer, 1821: 13 [F]. Type species: *Gnathosiaglabra* Fischer, 1821, by monotypy. Status: valid genus in Pimeliinae: Tentyriini.

*Gnatocerus* Thunberg, 1814: 47 [M]. Type species: *Gnatocerusruber* Thunberg, 1814 (= *Trogossitacornuta* Fabricius, 1798), by monotypy (see ICZN 1975, Opinion 1039). Status: valid genus and subgenus in Diaperinae: Diaperini: Adelinina. Note: placed on the Official List of Generic Names in Zoology by the ICZN (1975, Opinion 1039).

*Gnesis* Pascoe, 1866a: 477 [M]. Type species: *Gnesishelopioides* Pascoe, 1866, by monotypy. Status: valid genus in Stenochiinae: Cnodalonini.

*Gnophota* Erichson, 1843: 237 [F]. Type species: *Gnophotaantracina* Erichson, 1843, by subsequent designation (R. Lucas 1920: 306). Status: valid genus in Pimeliinae: Tentyriini.

*Goajiria* Ivie & Hart, 2016: 474 [F]. Type species: *Diastolinuscurtus* Mulsant & Rey, 1859, by original designation. Status: valid subgenus of *Diastolinus* Mulsant & Rey, 1859 in Blaptinae: Opatrini: Blapstinina. Note: name first introduced by Marcuzzi (1986: 180) without a type species designation.

*Gobretus* Freude, 1967: 148, 152 [M]. Type species: *Epitraguscephalotes* Freude, 1967, by monotypy. Status: valid subgenus of *Epitragus* Latreille, 1802 in Pimeliinae: Epitragini.

*Gonasida* Casey, 1912: 75, 117 [F]. Type species: *Pelecyphoruselatus* J.L. LeConte, 1853, by original designation. Status: valid subgenus of *Philolithus* Lacordaire, 1858 in Pimeliinae: Asidini.

*Gondvanadelium* Kaszab, 1981a: 81 [N]. Type species: *Gondvanadeliumseirotranoides* Kaszab, 1981, by original designation. Status: valid genus in Lagriinae: Adeliini.

*Gondwanocrypticus* Español, 1955: 10 [M]. Type species: *Crypticusplatensis* Fairmaire, 1884, by original designation. Status: valid genus in Diaperinae: Crypticini. Note: this name was introduced earlier by Koch (1950c: 64) without a description, a definition, or a bibliographic reference to such a published statement (ICZN 1999, Article 13.1) and is therefore unavailable from that date.

*Gondwanodilamus* Kaszab, 1969b: 320 [M]. Type species: *Conibiusfranzi* Kaszab, 1969, by original designation. Status: junior synonym of *Conibius* J.L. LeConte, 1851 in Blaptinae: Opatrini: Blapstinina. Synonymy: **new synonym** [RLA]. Note: the new synonymy is based on taxonomic studies of all known species in the genus *Conibius* J.L. LeConte, 1851 and follows the previous synonymization of the two other subgenera previously treated as valid, *Ooconibius* Casey, 1895 and *Euconibius* Casey, 1895, by Aalbu in Bousquet et al. (2018: 202).

*Gonespites* Gebien, 1921a: 325, 396 [M]. Type species: *Gonespitessubcrenatus* Gebien, 1921, by monotypy. Status: valid genus in Stenochiinae: Cnodalonini.

*Goniadera* Perty, 1832: 62 [F]. Type species: *Goniaderacrenata* Perty, 1832, by monotypy. Status: valid genus and subgenus in Lagriinae: Goniaderini. Note: the alternative original spelling *Gonyodera*, used by Perty (1832: 63), was rejected by Perty (1833: 14) who acted as the First Reviser.

†*Gonialaena* Nabozhenko, Bukejs & Telnov, 2019: 254 [F]. Type species: *Gonialaenagroehni* Nabozhenko, Bukejs & Telnov, 2019, by original designation. Status: valid genus in Lagriinae: Gonialaenini. Note: described from Eocene Baltic amber.

*Goniodera* Agassiz, 1846b: 165 [F]. Type species [automatic]: *Goniaderacrenata* Perty, 1832, by monotypy. Status: junior synonym of *Goniadera* Perty, 1832 in Lagriinae: Goniaderini. Note: unjustified emendation of *Goniadera* Perty, 1832, not in prevailing usage.

*Gonocephalum* Solier, 1834: 498 [N]. Type species: *Opatrumfuscum* Herbst, 1793 (= *Opatrumrusticum* G.-A. Olivier, 1812), by subsequent designation (Gebien 1939: 443). Status: valid genus and subgenus in Blaptinae: Opatrini: Opatrina.

*Gonocnemis* J. Thomson, 1858: 101 [F]. Type species: *Gonocnemisstrigipennis* J. Thomson, 1858, by monotypy. Status: valid genus in Tenebrioninae: Amarygmini.

*Gonocnemocistela* Pic, 1935a: 22 [F]. Type species: *Gonocnemocistelalutea* Pic, 1935, by monotypy. Status: valid genus in Tenebrioninae: Amarygmini.

*Gonodera* Mulsant, 1856a: 41 [F]. Type species: *Cistelafulvipes* Fabricius, 1792 (= *Cistelaluperus* Herbst, 1783), by monotypy. Status: valid genus in Alleculinae: Alleculini: Gonoderina.

*Gonogenius* Solier, 1838b: 8, 48 [M]. Type species: *Scotobiusvulgaris* Guérin-Méneville, 1834, by original designation. Status: junior synonym of *Scotobius* Germar, 1823 in Tenebrioninae: Scotobiini. Synonymy: Lacordaire (1859a: 129).

*Gonopterus* Solier, 1843: 101 [M]. Type species: *Sepidiumrugosum* Fabricius, 1781, by monotypy. Status: junior synonym of *Somaticus* Hope, 1841 in Pimeliinae: Sepidiini: Trachynotina. Synonymy: Gemminger in Gemminger and Harold (1870: 1900).

*Gonopus* Latreille, 1828: 580 [M]. Type species: *Blapstibialis* Fabricius, 1798, by monotypy. Status: valid genus and subgenus in Blaptinae: Platynotini: Platynotina.

*Gonospa* Champion, 1886: 216 [F]. Type species: *Gonospaphaedonoides* Champion, 1886, by subsequent designation (Gebien 1940: 426). Status: valid genus in Stenochiinae: Cnodalonini.

*Grabulax* Kanda, 2016: 558 [M]. Type species: *Grabulaxdarlingtoni* Kanda, 2016, by original designation. Status: valid genus in Lagriinae: Laenini.

*Gracilasida* Escalera, 1905b: 445 [F]. Type species: *Asidaariasi* Escalera, 1909, by subsequent designation (F. Soldati 2008: 32). Status: valid subgenus of *Asida* Latreille, 1802 in Pimeliinae: Asidini. Note: this genus was established without included nominal species; *Asidaariasi* Escalera, 1909 and *A.pusillima* Kraatz, 1874 were first subsequently and expressly included in *Gracilasida* by Escalera (1909: 135–136).

*Graecopachys* Skopin, 1968a: 99 [M]. Type species: *Tentyriaquadricollis* Brullé, 1832, by original designation. Status: junior synonym of *Phymatiotris* Solier, 1836 in Pimeliinae: Pimeliini. Synonymy: Löbl et al. (2008a: 43).

*Grammicus* G.R. Waterhouse, 1845b: 323 [M]. Type species: *Grammicuschilensis* G.R. Waterhouse, 1845, by monotypy. Status: valid genus in Pimeliinae: Stenosini: Stenosina.

*Granasida* Reitter, 1917a: 9, 14 [F]. Type species: *Asidagranulifera* Chevrolat, 1840, by monotypy. Status: valid subgenus of *Alphasida* Escalera, 1905 in Pimeliinae: Asidini.

*Grandelagria* Pic, 1940: 3 [F]. Type species: *Grandelagriabicoloripes* Pic, 1940, by monotypy. Status: valid subgenus of *Lagria* Fabricius, 1775 in Lagriinae: Lagriini: Lagriina.

*Grandicyrtomius* Freude, 1967: 225 [M]. Type species: *Epitragusgrandis* Champion, 1884, by original designation. Status: valid subgenus of *Cyrtomius* Casey, 1907 in Pimeliinae: Epitragini.

*Granulasida* Escalera, 1922a: 427 [F]. Type species: *Asidasetipennis* Allard, 1869, by subsequent designation (Viñolas and Cartagena 2005: 192). Status: junior synonym of *Gracilasida* Escalera, 1905 in Pimeliinae: Asidini. Synonymy: F. Soldati (2008: 34, with *Planasida* Escalera, 1907, a junior synonym of *Gracilasida* Escalera, 1905).

*Granulophanes* Nabozhenko, 2013: 2 [M]. Type species: *Hedyphaneslutosus* Allard, 1877, by original designation. Status: valid subgenus of *Hedyphanes* Fischer, 1820 in Tenebrioninae: Helopini: Helopina.

*Graptopezus* Gebien, 1921a: 296 [M]. Type species: *Seteniscostipennis* Blair, 1915 (= *Nyctozoiluscrenaticollis* W.J. MacLeay, 1886), by original designation. Status: valid genus in Stenochiinae: Cnodalonini.

*Gressittiola* Kaszab, 1955a: 462, 464 [F]. Type species: *Gressittiolaplatydemoides* Kaszab, 1955, by original designation. Status: valid genus in Diaperinae: Diaperini: Diaperina.

*Gridellia* Kammerer, 2006: 270 [F]. Type species [automatic]: *Tenebrioclypealis* Gebien, 1920, by original designation. Status: valid genus in Tenebrioninae: Tenebrionini. Note: replacement name for *Villiersia* Gridelli, 1951.

*Gridelliopus* Koch, 1956a: 358 [M]. Type species: *Gridelliopussubsquamosus* Koch, 1956, by monotypy. Status: valid genus in Blaptinae: Dendarini: Melambiina.

*Gronophora* Borchmann, 1916a: 48, 103 [F]. Type species: *Gronophoragravida* Borchmann, 1916, by monotypy. Status: valid genus in Lagriinae: Lagriini: Lagriina.

*Guanobius* Grimm, 2008: 375 [M]. Type species: *Guanobiusborneensis* Grimm, 2008, by original designation. Status: valid genus in Tenebrioninae: Alphitobiini.

*Guildia* Antoine, 1957: 346, 359 [F]. Type species: *Microsituspunctistriatus* Escalera, 1925, by monotypy. Status: valid genus in Blaptinae: Dendarini: Melambiina.

*Gunarellus* Reitter, 1922a: 22 [M]. Type species: *Helopsgratus* Frivaldszky, 1894, by subsequent designation (Nabozhenko 2008: 38). Status: junior synonym of *Stenohelops* Reitter, 1922 in Tenebrioninae: Helopini: Helopina. Synonymy: Nabozhenko et al. (2020b: 297). Note: *Gunarellus* was first described by Reitter (1922a: 22, in key; issued 30 March 1922) without originally included species; Reitter (1922b: 114–115; issued 25 October 1922) was the first author to subsequently and expressly include nominal species in *Gunarellus* (ICZN 1999, Article 67.2.2).

*Gunarus* Gozis, 1886: 25 [M]. Type species: *Helopshirtulus* Reiche, 1862, by monotypy. Status: valid genus in Tenebrioninae: Helopini: Cylindrinotina.

*Gymnetasida* Reitter, 1917a: 10, 22 [F]. Type species: *Asidatricostata* Allard, 1869, by subsequent designation (F. Soldati 2008: 33). Status: junior synonym of *Glabrasida* Escalera, 1910 in Pimeliinae: Asidini. Synonymy: Viñolas and Cartagena (2005: 282).

*Gymnognathus* Solier, 1851: 136 [M]. Type species: *Gymnognathusfuscus* Solier, 1851, by monotypy. Status: junior synonym of *Arthroconus* Solier, 1851 in Pimeliinae: Edrotini. Synonymy: Lacordaire (1859a: 67). Note: junior homonym of *Gymnognathus* Schönherr, 1823 [Coleoptera: Anthribidae].

*Gynandrocera* Gebien, 1920: 46 [F]. Type species: *Gynandroceracephalotes* Gebien, 1920 (= *Afrinusminor* Péringuey, 1908), by subsequent designation (Koch 1950b: 328). Status: valid subgenus of *Afrinus* Fairmaire, 1888 in Pimeliinae: Tentyriini.

*Gyrasida* Koch, 1962a: 127, 143 [F]. Type species: *Afrasidapropensa* Wilke, 1922, by original designation. Status: valid genus in Pimeliinae: Praociini. Note: elevated to the rank of genus and transferred from Pimeliinae: Asidini by Flores and Vidal (2009: 52); as mentioned by Flores and Vidal (2009: 48, 56) the type species was originally described from South Africa in error: the genus *Gyrasida* Koch, 1962 is endemic to Chile.

*Gyrinodes* Fauvel, 1897: 61 [M]. Type species: *Helopsgagatinus* Küster, 1850, by monotypy. Status: junior synonym of *Nesotes* Allard, 1876 in Tenebrioninae: Helopini: Helopina. Synonymy: **new synonym** [YB]. Note: this genus-group taxon has been forgotten in the literature; its type species is currently placed in the nominotypical subgenus of *Nesotes* Allard, 1876 and for that reason Fauvel’s name is regarded as a new junior synonym of *Nesotes*.

*Gyriosomus* Guérin-Méneville, 1834: 6 [M]. Type species: *Nyctelialuczotii* Guérin-Méneville, 1831, by subsequent designation (Duponchel 1845b: 449). Status: valid genus in Pimeliinae: Nycteliini.

*Gyrosis* Gebien, 1920: 33, 37 [F]. Type species: *Zophosisorbicularis* Deyrolle, 1867, by original designation. Status: valid subgenus of *Zophosis* Latreille, 1802 in Pimeliinae: Zophosini.

*Habrobates* Semenov, 1903a: 11 [M]. Type species: *Habrobatesvernalis* Semenov, 1903, by monotypy. Status: valid genus in Pimeliinae: Pimeliini.

*Habrochiton* Semenov-Tjan-Shansky, 1907a: 177, 179 [M]. Type species: *Habrochitonvernus* Semenov-Tjan-Shansky, 1907, by original designation. Status: valid genus in Pimeliinae: Pimeliini.

*Hades* J. Thomson, 1860a: 13 [M]. Type species: *Hadestenebrosus* J. Thomson, 1860 (= *Platydemahemisphaerica* Laporte & Brullé, 1831), by monotypy. Status: senior synonym of *Pimplema* Pascoe, 1887 in Diaperinae: Leiochrinini. Synonymy: Gebien (1940: 755). Note: junior homonym of *Hades* Westwood, 1851 [Lepidoptera].

*Hadroderus* Koch, 1956a: 347 [M]. Type species: *Hadroderustuberculiferus* Koch, 1956, by monotypy. Status: valid genus in Blaptinae: Dendarini: Melambiina.

*Hadrodes* Wollaston, 1877: 226 [M]. Type species: *Hadrodeshelenensis* Wollaston, 1877, by monotypy. Status: valid genus in Blaptinae: Opatrini: AMMOBIINA.

*Hadromelambius* Koch, 1948: 432 [M]. Type species: *Melambiustelueticus* Escalera, 1914, by original designation. Status: valid subgenus of *Melambius* Mulsant & Rey, 1854 in Blaptinae: Dendarini: Melambiina.

*Hadrophasis* Ferrer, 1992: 87 [F]. Type species: *Hadrophasisangolensis* Ferrer, 1992, by original designation. Status: valid genus in Blaptinae: Opatrini: Opatrina.

*Hadrus* Wollaston, 1854: 502 [M]. Type species: *Hadrusalpinus* Wollaston, 1854, by subsequent designation (R. Lucas 1920: 313). Status: senior synonym of *Wolladrus* Iwan & Kamiński, 2016 in Blaptinae: Opatrini: Opatrina. Note: junior homonym of *Hadrus* Perty, 1833 [Diptera].

*Haemerophygus* Baudi di Selve, 1876b: 266 [M]. Type species: *Helopsmucoreus* Waltl, 1838, by monotypy. Status: junior synonym of *Ceratanisus* Gemminger, 1870 in Pimeliinae: Ceratanisini. Synonymy: Nabozhenko et al. (2016b: 608).

*Haemodus* Gebien, 1943: 904 [M]. Type species [automatic]: *Haemuscarinatipennis* Péringuey, 1904, by monotypy. Status: valid genus in Blaptinae: Dendarini: Melambiina. Note: replacement name for *Haemus* Péringuey, 1904.

*Haemus* Péringuey, 1904: 228 [M]. Type species: *Haemuscarinatipennis* Péringuey, 1904, by monotypy. Status: senior synonym of *Haemodus* Gebien, 1943 in Blaptinae: Dendarini: Melambiina. Note: junior homonym of *Haemus* Stål, 1862 [Hemiptera].

*Halammobia* Semenov, 1901: 92 [F]. Type species: *Tenebriopellucidus* Herbst, 1799, by monotypy. Status: valid genus in Diaperinae: Phaleriini.

*Halonomus* Wollaston, 1861: 201 [M]. Type species: *Halonomusgrayii* Wollaston, 1861 (= *Opatrumovatum* Erichson, 1843), by subsequent designation (Iwan and Löbl 2007: 734). Status: junior synonym of *Clitobius* Mulsant & Rey, 1859 in Blaptinae: Opatrini: Ammobiina. Synonymy: Fairmaire (1886b: ccv). Note: the original combination of the accepted name of the type species, *Opatrumovatum* Erichson, 1843, is a junior primary homonym of *Opatrumovatum* Fabricius, 1801.

*Halophalerus* Crotch, 1874: 107 [M]. Type species: *Phaleriarotundata* J.L. LeConte, 1851, by subsequent designation (Bousquet et al. 2018: 289). Status: junior synonym of *Phaleria* Latreille, 1802 in Diaperinae: Phaleriini. Synonymy: Austin (1880: 38).

*Hangaya* Matthews & Merkl, 2015: 479 [F]. Type species: *Hangayaenigmatica* Matthews & Merkl, 2015, by original designation. Status: valid genus in Tenebrioninae: incertae sedis. Note: according to Matthews and Lawrence (2019: 628) the closest relative of this genus within the subfamily Tenebrioninae remains to be determined.

*Hanstroemium* Koch, 1953c: 19 [N]. Type species: *Hanstroemiumadelostomoide* Koch, 1953, by original designation. Status: valid genus in Blaptinae: Dendarini: Melambiina.

*Haplandrus* J.L. LeConte, 1862: 230 [M]. Type species: *Helopsfemoratus* Fabricius, 1798 (= *Upisfulvipes* Herbst, 1797), by monotypy. Status: valid genus in Stenochiinae: Cnodalonini.

*Haporema* Fairmaire, 1892a: 109 [F]. Type species: *Haporemadecipiens* Fairmaire, 1892, by monotypy. Status: valid genus in Stenochiinae: Cnodalonini.

*Hapsida* Gemminger in Gemminger and Harold, 1870: 1955 [F]. Type species [automatic]: *Apsidachrysomelina* Lacordaire, 1859, by original designation. Status: junior synonym of *Apsida* Lacordaire, 1859 in Stenochiinae: Cnodalonini. Note: unjustified emendation of *Apsida* Lacordaire, 1859, not in prevailing usage.

*Harvengia* Ferrer, 2004b: 367 [F]. Type species: *Harvengiavietnamita* Ferrer, 2004, by original designation. Status: valid genus in Pimeliinae: Stenosini: Harvengiina.

*Hasticollinum* Kaszab, 1939a: 96 [N]. Type species: *Hasticollinumpodagrarium* Kaszab, 1939, by original designation. Status: junior synonym of *Gonocephalum* Solier, 1834 in Blaptinae: Opatrini: Opatrina. Synonymy: Ferrer (1995a: 72).

*Havanalia* Novák, 2020c: 489 [F]. Type species: *Havanaliaqazvinica* Novák, 2020, by original designation. Status: valid genus in Alleculinae: Alleculini: Alleculina.

*Hectus* Pascoe, 1869: 288, 289 [M]. Type species: *Hectusanthracinus* Pascoe, 1869, by monotypy. Status: junior synonym of *Olisthaena* Erichson, 1842 in Tenebrioninae: Heleini: Cyphaleina. Synonymy: Carter (1913a: 62).

*Hedrotes* Gemminger in Gemminger and Harold, 1870: 1816 [M]. Type species [automatic]: *Edrotesventricosus* J.L. LeConte, 1851, by monotypy. Status: junior synonym of *Edrotes* J.L. LeConte, 1851 in Pimeliinae: Edrotini. Note: unjustified emendation of *Edrotes* J.L. LeConte, 1851, not in prevailing usage.

*Hedyphanes* Fischer, 1820: pl.15 [M]. Type species: *Hedyphanescoerulescens* Fischer, 1820, by monotypy. Status: valid genus and subgenus in Tenebrioninae: Helopini: Helopina.

*Hegemona* Laporte, 1840: 230 [F]. Type species: *Hegemonaresplendens* Laporte, 1840, by monotypy. Status: valid genus in Stenochiinae: Cnodalonini.

*Hegeter* Latreille, 1802: 172 [M]. Type species: *Hegeterstriatus* Latreille, 1804 (= *Blapstristis* Fabricius, 1792), by subsequent monotypy (Latreille 1804: 276). Status: valid genus and subgenus in Pimeliinae: Tentyriini. Note: originally proposed without included nominal species; Latreille (1804: 276), by including the species *Hegeterstriatus* Latreille, 1804 in association with this name, was the first author to subsequently and expressly include nominal species in *Hegeter* (ICZN 1999, Article 67.2.2); the previously accepted type species for this genus was *Blapselongata* G.-A. Olivier, 1795 (= *Blapstristis* Fabricius, 1792), by subsequent designation (Latreille 1810: 429); Latreille (1804: 276) mentioned “Le type de ce genre paroît être le blaps alongé d’Olivier” however he did not use a Latin name for the species.

*Hegeterocara* Reitter, 1900c: 94, 190 [N]. Type species: *Hegeterocaraarabicum* Reitter, 1900, by monotypy. Status: valid genus in Pimeliinae: Tentyriini.

*Hegeteromorpha* Escalera, 1913: 38 [F]. Type species: *Pachychilaexternecostata* Haag-Rutenberg, 1875, by subsequent monotypy (Escalera 1914: 281). Status: valid subgenus of *Pachychila* Eschscholtz, 1831 in Pimeliinae: Tentyriini. Note: originally proposed without included nominal species; Escalera (1914: 281), by including the species *Pachychilaexternecostata* Haag-Rutenberg, 1875 in association with this name, was the first author to subsequently and expressly include nominal species in *Hegeteromorpha* (ICZN 1999, Article 67.2.2).

*Heinrichesia* Carl, 2000: 258 [F]. Type species: *Heinrichesiaschaeferi* Carl, 2000, by monotypy. Status: junior synonym of *Waterhousia* Skopin, 1973 in Pimeliinae: Pimeliini. Synonymy: G.S. Medvedev (2005a: 309). Note: combined description of new genus-group taxon and new species (ICZN 1999, Article 13.4).

*Helea* Latreille, 1804: 326 [F]. Type species: *Heleaperforata* Latreille, 1817, by subsequent monotypy (Latreille 1817: 261). Status: valid genus in Tenebrioninae: Heleini: Heleina. Note: the name *Helea* Meigen, 1800 (Diptera) was suppressed for the purposes of zoological nomenclature by the ICZN (1963, Opinion 678); originally proposed without included nominal species; Latreille (1817: 261), by including the new species *Heleaperforata* Latreille, 1817 [as *Heleusperforatus*] in association with this name, was the first author to subsequently and expressly include nominal species in *Helea* (ICZN 1999, Article 67.2.2).

*Heledona* Agassiz, 1846b: 135, 174 [F]. Type species [automatic]: *Opatrumagricola* Herbst, 1783, by subsequent monotypy (Latreille 1802: 162). Status: junior synonym of *Eledona* Latreille, 1797 in Tenebrioninae: Bolitophagini. Note: unjustified emendation of *Eledona* Latreille, 1797, not in prevailing usage.

*Helenomelas* Ardoin, 1972: 190 [M]. Type species: *Helenomelasbasilewskyi* Ardoin, 1972, by monotypy. Status: valid genus in Blaptinae: Opatrini: Ammobiina.

*Helenophorus* Gemminger in Gemminger and Harold, 1870: 1851 [M]. Type species [automatic]: *Tenebriocollaris* Linnaeus, 1767, by monotypy. Status: junior synonym of *Leptoderis* Billberg, 1820 in Pimeliinae: Elenophorini: Elenophorina. Note: unjustified emendation of *Elenophorus* Dejean, 1821, not in prevailing usage.

*Helibatus* Mulsant & Rey, 1859c: 94, 100 [M]. Type species: *Helibatusmorio* Mulsant & Rey, 1859, by monotypy. Status: valid genus in Blaptinae: Opatrini: Stizopodina.

*Helioarthrodibius* Koch, 1960: 353 [M]. Type species: *Arthrodeisruguliventris* Fairmaire, 1884, by monotypy. Status: valid subgenus of *Arthrodibius* Lesne, 1915 in Pimeliinae: Erodiini.

*Heliocaes* Bedel, 1906a: 92 [M]. Type species: *Blaps emarginata* Fabricius, 1792, by **present designation**. Status: junior synonym of *Heliopates* Dejean, 1834 in Blaptinae: Dendarini: Dendarina. Synonymy: Gebien (1938a: 304). Note: this genus was proposed as a replacement name for “*Heliopathes*” Dejean sensu Mulsant, 1854, an incorrect subsequent spelling of *Heliopates* Dejean, 1834, which is unavailable (ICZN 1999, Article 33.3); *Heliocaes* Bedel, 1906 was made available by indication (ICZN 1999, Article 12.2.1); Escalera (1914: 326) was the first author to subsequently and expressly include nominal species in *Heliocaes* (ICZN 1999, Article 67.2.2).

*Heliocrates* Reitter, 1904: 98 [M]. Type species: *Heliophilushumerangulus* Reitter, 1904, by subsequent designation (Iwan and Löbl 2007: 734). Status: valid subgenus of *Heliopates* Dejean, 1834 in Blaptinae: Dendarini: Dendarina.

*Heliodromus* Brullé, 1832: 196 [M]. Type species: *Heliodromusrotundatus* Brullé, 1832, by subsequent designation (Löbl et al. 2008a: 41). Status: junior synonym of *Tentyria* Latreille, 1802 in Pimeliinae: Tentyriini. Synonymy: Solier (1835b: 314).

*Heliofugus* Guérin-Méneville, 1831a: pl. 4 [M]. Type species: *Heliofugusarenosus* Guérin-Méneville, 1831, by monotypy. Status: valid genus and subgenus in Stenochiinae: Cnodalonini.

*Heliomelasma* Koch, 1948: 408 [N]. Type species: *Melasmanaappenhageni* Koch, 1948, by original designation. Status: valid subgenus of *Melasmana* Strand, 1935 in Blaptinae: Dendarini: Melambiina.

*Heliomophlus* Reitter, 1906b: 147 [M]. Type species: *Heliotaurusscabriusculus* Fairmaire, 1866, by subsequent designation (R. Lucas 1920: 319). Status: valid genus in Alleculinae: Cteniopodini.

*Heliopates* Dejean, 1834: 191 [M]. Type species: *Tenebriolusitanicus* Herbst, 1797, by subsequent designation (Gebien 1938a: 304). Status: valid genus and subgenus in Blaptinae: Dendarini: Dendarina. Note: this genus was originally proposed as a replacement name for the junior homonym *Heliophilus* Dejean, 1821 (see Bousquet and Bouchard 2013a: 46) and should have the same type species as *Heliophilus* Dejean, 1821 (i.e., *Pedinushybridus* Latreille, 1804); however, recent authors (e.g., Silfverberg 1984: 59, Iwan and Löbl 2008: 279, Iwan and Löbl 2020: 302) have followed the type species designation proposed by Gebien (1938a: 304) and treated *Heliopates* as a separate genus with *Tenebriolusitanicus* Herbst, 1797 as its type species; in order to maintain nomenclatural stability we recommend that an application be submitted to the International Commission on Zoological Nomenclature to request that the type species designation proposed by Gebien (1938a: 304) be used for *Heliopates* Dejean, 1834.

*Heliopathes* Gebien, 1938a: 304 [M]. Type species: *Tenebriolusitanicus* Herbst, 1797, by subsequent designation (Gebien 1938a: 304). Status: junior synonym of *Heliopates* Dejean, 1834 in Blaptinae: Dendarini: Dendarina. Note: unjustified emendation of *Heliopates* Dejean, 1834, not in prevailing usage; see comments regarding the type species of *Heliopates* Dejean, 1834 under that name.

*Heliophilus* Dejean, 1821: 65 [M]. Type species: *Pedinushybridus* Latreille, 1804, by subsequent designation (Duponchel 1845b: 517). Status: senior synonym of *Phylan* Sturm, 1826 in Blaptinae: Dendarini: Dendarina. Synonymy: Iwan and Löbl (2008: 282). Note: junior homonym of *Heliophilus* Meigen, 1803 [Diptera]; the currently accepted valid name of the type species, “*Phylan abbreviatus* (G.-A. Olivier, 1795)”, should not be considered as an available name since G.-A. Olivier (1795: [57] 17) clearly refers to *Tenebrioabbreviatus* Fabricius, 1775.

*Heliophosis* Koch, 1952a: 95 [F]. Type species: *Heliophosiskalaharica* Koch, 1952, by original designation. Status: valid subgenus of *Zophosis* Latreille, 1802 in Pimeliinae: Zophosini.

*Heliophygus* Agassiz, 1846b: 175 [M]. Type species [automatic]: *Heliofugusarenosus* Guérin-Méneville, 1831, by monotypy. Status: junior synonym of *Heliofugus* Guérin-Méneville, 1831 in Stenochiinae: Cnodalonini. Note: unjustified emendation of *Heliofugus* Guérin-Méneville, 1831, not in prevailing usage.

*Heliosteres* Hope, 1841: 124 [M]. Type species [automatic]: *Heliofugusarenosus* Guérin-Méneville, 1831, by monotypy. Status: junior synonym of *Heliofugus* Guérin-Méneville, 1831 in Stenochiinae: Cnodalonini. Note: unnecessary replacement name for *Heliofugus* Guérin-Méneville, 1831.

*Heliostrhaema* Reitter, 1890a: 34 [N]. Type species: *Heliotaurusrolphii* Fairmaire, 1867, by subsequent designation (R. Lucas 1920: 319). Status: valid genus in Alleculinae: Cteniopodini. Note: *Heliostrhaema* is an unjustified emendation of the original spelling *Heliosthraema*, introduced by Seidlitz (1896: 223), in prevailing usage and attributed to the original author and date; it is considered here to be a justified emendation (ICZN 1999, Article 33.2.3.1).

*Heliotaurus* Mulsant, 1856a: 73 [M]. Type species: *Cisteladistincta* Laporte, 1840, by subsequent designation (Novák and Pettersson 2008: 332). Status: valid genus and subgenus in Alleculinae: Cteniopodini.

*Helogria* Borchmann, 1916a: 48, 110 [F]. Type species: *Lagriapruinosa* Chevrolat, 1841, by subsequent designation (Borchmann 1936: 143). Status: valid genus in Lagriinae: Lagriini: Lagriina.

*Helopelius* Reitter, 1922b: 123, 152 [M]. Type species: *Stenomaxaeneipennis* Allard, 1876, by subsequent designation (Nabozhenko 2008: 38). Status: valid subgenus of *Stenohelops* Reitter, 1922 in Tenebrioninae: Helopini: Helopina. Note: downgraded to a subgenus of *Stenohelops* Reitter, 1922 by Nabozhenko et al. (2020b: 293).

*Helopidesthes* Fairmaire, 1895b: 446 [F]. Type species: *Helopidesthescoriaria* Fairmaire, 1895, by monotypy. Status: valid genus in Tenebrioninae: Helopini: incertae sedis.

*Helopidoxus* Reitter, 1922a: 32, 44 [M]. Type species: *Helopssuperbus* Mulsant & Godart, 1855, by monotypy. Status: valid subgenus of *Euboeus* Boieldieu, 1865 in Tenebrioninae: Helopini: Helopina.

*Helopimorphus* Desbrochers des Loges, 1881: 140 [M]. Type species: *Helopimorphusangulipennis* Desbrochers des Loges, 1881, by monotypy. Status: junior synonym of *Heterotarsus* Latreille, 1829 in Blaptinae: Opatrini: Heterotarsina. Synonymy: Seidlitz (1896: 24).

*Helopinus* Solier, 1848: 152, 197 [M]. Type species: *Helopinuscostatus* Solier, 1848, by monotypy. Status: valid subgenus of *Drosochrus* Erichson, 1843 in Blaptinae: Pedinini: Helopinina. Note: the First Reviser (*Helopinus* Solier, 1848 versus *Pteraulus* Solier, 1848) is Koch (1958: 149)

*Helopocerodes* Reitter, 1922b: 122, 144 [M]. Type species: *Helopsfaldermanni* Faldermann, 1837, by subsequent designation (Nabozhenko 2001a: 633). Status: junior synonym of *Nalassus* Mulsant, 1854 in Tenebrioninae: Helopini: Cylindrinotina. Synonymy: Keskin et al. (2017: 726).

*Helopogonus* Reitter, 1922b: 122, 150 [M]. Type species: *Helopsviridicollis* Schaufuss, 1869, by monotypy. Status: valid subgenus of *Nesotes* Allard, 1876 in Tenebrioninae: Helopini: Helopina.

*Helopondrus* Reitter, 1922b: 123, 153 [M]. Type species: *Stenomaxsareptanus* Allard, 1876, by subsequent designation (Nabozhenko 2001a: 652). Status: junior synonym of *Horistelops* Gozis, 1910 in Tenebrioninae: Helopini: Cylindrinotina. Synonymy: **new synonym** [YB]. Note: *Horistelops* Gozis, 1910 has been forgotten in the literature; its type species is currently included in the subgenusHelopondrus Reitter, 1922 and for that reason Reitter’s name is considered a new junior synonym of *Horistelops*.

*Helopostygnus* Antoine, 1949: 133, 153 [M]. Type species: *Helopsatlantis* Antoine, 1926, by original designation. Status: valid subgenus of *Euboeus* Boieldieu, 1865 in Tenebrioninae: Helopini: Helopina.

*Helopotrichus* Reitter, 1922a: 32, 44 [M]. Type species: *Helopsvillosipennis* P.H. Lucas, 1846, by subsequent designation (Viñolas and Cartagena 2005: 38). Status: valid subgenus of *Euboeus* Boieldieu, 1865 in Tenebrioninae: Helopini: Helopina.

*Helops* Fabricius, 1775: 257 [M]. Type species: *Tenebriocaeruleus* Linnaeus, 1758, by subsequent designation (Hope 1841: 133; see ICZN 2009, Opinion 2237). Status: valid genus in Tenebrioninae: Helopini: Helopina. Note: as mentioned in Bousquet et al. (2018: 183) and Vela et al. (2020: 63) the type of the nominal species *Tenebriocaeruleus* Linnaeus, 1758 is actually a leaf beetle that belongs to the genus *Timarcha* Samouelle, 1819 (Coleoptera: Chrysomelidae) based on examination of a syntype; since *Tenebriocaeruleus* Linnaeus, 1758 was placed on the Official List of Specific Names in Zoology and fixed as the type species of *Helops* Fabricius, 1775 in Opinion 2237 (ICZN 2009), an application to the International Commission on Zoological Nomenclature is necessary to request that the type species of *Helops* be changed to *Tenebriocaeruleus* Linnaeus sensu Fabricius, 1775 (= *Helopschalibaeus* Rossi, 1790).

*Helopsallecula* Pic, 1936a: 33 [F]. Type species: *Helopsalleculaminutissima* Pic, 1936, by monotypy. Status: valid genus in Alleculinae: incertae sedis.

*Helopsisomira* Pic, 1952a: 63 [F]. Type species: *Helopsisomirakochi* Pic, 1952, by monotypy. Status: valid genus in Alleculinae: Alleculini: Gonoderina.

*Hemasodes* Casey, 1907: 378 [M]. Type species: *Schoenicusvestitus* Champion, 1884, by original designation. Status: valid genus in Pimeliinae: Epitragini.

*Hemeralopius* Gistel, 1848a: viii, 125 [M]. Type species [automatic]: *Tenebrioelongatus* Herbst, 1797, by monotypy. Status: senior synonym of *Belopus* Gebien, 1911 in Lagriinae: Belopini. Note: replacement name for *Calcar* Dejean, 1821; nomen oblitum (see Bouchard and Bousquet 2020b: 6).

*Hemerobates* Kolbe, 1884: 189 [M]. Type species: *Nyctobatesmechowi* Kolbe, 1884, by monotypy. Status: junior synonym of *Amenophis* J. Thomson, 1858 in Stenochiinae: Cnodalonini. Synonymy: Gebien (1911a: 441).

*Hemicera* Laporte & Brullé, 1831: 332, 393 [F]. Type species: *Cnodalonsplendens* Wiedemann, 1823, by subsequent designation (Gebien 1921a: 335). Status: valid genus and subgenus in Stenochiinae: Cnodalonini. Note: nomenclatural stability in this genus is threatened by the discovery of an older type species designation (*Hemiceraarcuata* Laporte & Brullé, 1831 [misspelled “*Hemiceraarmata*”], by subsequent designation by Duponchel (1845b: 528), which is currently the type species of the valid genus *Hypocalis* Dejean, 1834); we recommend that an application be submitted to the International Commission on Zoological Nomenclature to maintain the type species designation proposed by Gebien (1921a: 335).

*Hemicistela* Blackburn, 1891: 331 [F]. Type species: *Hemicisteladiscoidalis* Blackburn, 1891, by monotypy. Status: valid genus in Alleculinae: Alleculini: Alleculina.

*Hemicyclus* Westwood, 1841a: 44 [M]. Type species: *Hemicyclusgrandis* Westwood, 1841 (= *Tetraphyllusreaumuri* Laporte, 1840), by subsequent designation (Gebien 1941: 1133). Status: valid genus in Tenebrioninae: Heleini: Cyphaleina.

*Hemimmedia* Gebien, 1928: 220, 229 [F]. Type species: *Hemimmediacorpulenta* Gebien, 1928, by monotypy. Status: valid genus in Stenochiinae: Cnodalonini.

*Hemipraocis* Flores & Pizarro-Araya, 2014: 66 [M]. Type species: *Praocissellatus* Berg, 1889, by original designation. Status: valid subgenus of *Praocis* Eschscholtz, 1829 in Pimeliinae: Praociini. Note: this name was first proposed by Kulzer (1958a: 13, 60) without type species designation; the fact that *Praocissellatus* Berg, 1889 was listed as the “type” of *Hemipraocis* in the Zoological Record for the year 1958 (Anonymous in Commonwealth Institute of Entomology 1960) does not represent a valid type species designation since the nomenclatural act is anonymous (ICZN 1999, Article 14).

*Hemipristis* Kolbe, 1903: 165, 177 [F]. Type species: *Hemipristisukamia* Kolbe, 1903, by **present designation**. Status: senior synonym of *Hemipristula* Bouchard & Bousquet, **nom. nov.** in Lagriinae: Pycnocerini. Note: junior homonym of *Hemipristis* Agassiz, 1833 [Pisces].

*Hemipristula* Bouchard & Bousquet, **new replacement name** [F]. Type species [automatic]: *Hemipristisukamia* Kolbe, 1903, by **present designation**. Status: valid genus in in Lagriinae: Pycnocerini. Note: replacement name *Hemipristis* Kolbe, 1903; *Hemipristula* was proposed earlier by Strand (1935a: 291); however, this name is unavailable because Strand did not designate a type species for the nominal taxon, a mandatory requirement for replacement name without valid typification proposed after 1930 (ICZN 1999; Article 13.3.1).

*Hemipterocoma* Skopin, 1974b: 159 [F]. Type species: *Pterocomanikolskii* Semenov-Tjan-Shansky, 1910, by original designation. Status: valid subgenus of *Pterocoma* Dejean, 1834 in Pimeliinae: Pimeliini.

*Hemitrichestes* Reitter, 1904: 169 [M]. Type species: *Penthicushirsutus* Reitter, 1896, by monotypy. Status: junior synonym of *Melanesthes* Dejean, 1834 in Blaptinae: Opatrini: Opatrina. Synonymy: Löbl et al. (2008b: 267).

*Heptaphylla* Friedenreich, 1883: 375 [F]. Type species: *Heptaphyllafungicola* Friedenreich, 1883, by monotypy. Status: junior synonym of *Rhipidandrus* J.L. LeConte, 1862 in Tenebrioninae: Bolitophagini. Synonymy: Arrow (1904: 31).

*Herbertfranzia* Kaszab, 1973a: 26, 28 [F]. Type species: *Herbertfranzianepalica* Kaszab, 1973, by original designation. Status: valid genus in Pimeliinae: Stenosini: Dichillina.

*Herbertfranziella* Kaszab, 1973a: 26, 28 [F]. Type species: *Herbertfranziaeutagenoides* Kaszab, 1973, by monotypy. Status: valid genus in Pimeliinae: Stenosini: Dichillina.

*Herlesa* Reitter, 1897a: 298, 301 [F]. Type species: *Herlesaglobicollis* Reitter, 1897 (= *Micipsacavifrons* Fairmaire, 1863), by monotypy. Status: valid genus in Pimeliinae: Tentyriini.

*Herodius* Agassiz, 1846b: 143,179 [M]. Type species [automatic]: *Erodiusgibbus* Fabricius, 1775, by subsequent designation (Latreille 1810: 429). Status: junior synonym of *Erodius* Fabricius, 1775 in Pimeliinae: Erodiini. Note: unjustified emendation of *Erodius* Fabricius, 1775, not in prevailing usage.

*Herpiscius* Solier, 1838b: 160, 188 [M]. Type species: *Herpisciusspinolae* Solier, 1838, by subsequent designation (Hope 1841: 115). Status: valid genus in Tenebrioninae: Scaurini.

*Herpsis* Haag-Rutenberg, 1875b: 4, 66 [F]. Type species: *Adelostomarugosum* Guérin-Méneville, 1831, by monotypy. Status: valid genus in Pimeliinae: Adelostomini.

*Herthasida* Wilke, 1922: 269 [F]. Type species: *Asidaingens* Champion, 1892, by monotypy. Status: valid subgenus of *Philolithus* Lacordaire, 1858 in Pimeliinae: Asidini.

†*Hesiodobates* Kaszab & Schawaller, 1984: 1 [M]. Type species: *Hesiodobatesantiquus* Kaszab & Schawaller, 1984, by original designation. Status: junior synonym of *Nesocyrtosoma* Marcuzzi, 1976 in Stenochiinae: Cnodalonini. Synonymy: Doyen and Poinar (1994: 45). Note: described from Early Miocene amber (Dominican Republic).

*Hesiodus* Champion, 1885: 115 [M]. Type species: *Hesioduslongitarsis* Champion, 1885, by subsequent designation (R. Lucas 1920: 323). Status: valid genus in Stenochiinae: Cnodalonini.

*Hesperoptorina* G.S. Medvedev, 2009: 417 [F]. Type species: *Gnaptorinabrucei* Blair, 1923, by original designation. Status: valid subgenus of *Gnaptorina* Reitter, 1887 in Blaptinae: Blaptini: Gnaptorinina.

*Hesseodes* Ardoin, 1963a: 92 [M]. Type species: *Hoplonyxkalaharica* Hesse, 1935, by original designation. Status: valid genus in Tenebrioninae: Amarygmini. Note: the name *Hesseodes* used earlier by Ardoin (1962b: 969) is unavailable since it was published after 1930 without a type species designation.

*Hesseosis* Koch, 1958: 76 [F]. Type species: *Hesseosispurpurascens* Koch, 1958, by original designation. Status: valid subgenus of *Zophosis* Latreille, 1802 in Pimeliinae: Zophosini.

*Heterarthron* Gistel, 1848a: 190 [N]. Type species [automatic]: *Eulabisbicarinata* Eschscholtz, 1829, by subsequent designation (Blaisdell 1932: 44). Status: junior synonym of *Eulabis* Eschscholtz, 1829 in Tenebrioninae: Eulabini. Note: junior homonym of *Heterarthron* Dejean, 1836 [Coleoptera: Bostrichidae]; unnecessary replacement name for *Eulabis* Eschscholtz, 1829 (as “*Eulabes*”).

*Heterasida* Casey, 1912: 76, 165 [F]. Type species: *Pelecyphorusbifurcus* J.L. LeConte, 1861, by original designation. Status: valid genus in Pimeliinae: Asidini.

*Heterocheira* Dejean, 1836: 220 [F]. Type species: *Ulomaaustrale* Boisduval, 1835, by monotypy. Status: valid genus in Blaptinae: Opatrini: Heterotarsina.

*Heterochira* Agassiz, 1846b: 180 [F]. Type species [automatic]: *Ulomaaustrale* Boisduval, 1835, by monotypy. Status: junior synonym of *Heterocheira* Dejean, 1836 in Blaptinae: Opatrini: Heterotarsina. Note: unjustified emendation of *Heterocheira* Dejean, 1836, not in prevailing usage.

*Heterogena* Froussart, 1961: 60, 105 [F]. Type species: *Nesogenagoudotii* Fairmaire, 1868, by original designation. Status: valid subgenus of *Nesogena* Mäklin, 1863 in Tenebrioninae: Praeugenini.

*Heterogria* Fairmaire, 1896a: 42 [F]. Type species: *Heterogriapunctatissima* Fairmaire, 1896, by monotypy. Status: junior synonym of *Xanthalia* Fairmaire, 1894 in Lagriinae: Lagriini: Statirina. Synonymy: Merkl (2004: 285).

*Heteromerotylus* Pic, 1921b: 11 [M]. Type species: *Heteromerotylusbicoloripes* Pic, 1921, by monotypy. Status: junior synonym of *Tearchus* Kraatz, 1880 in Stenochiinae: Cnodalonini. Synonymy: Kulzer (1954a: 71).

*Heteromira* Hölzel, 1958: 19 [F]. Type species: *Isomiramoroi* Hölzel, 1958, by monotypy. Status: valid subgenus of *Isomira* Mulsant, 1856 in Alleculinae: Alleculini: Gonoderina.

*Heteronicandra* Koch, 1958: 151 [F]. Type species: *Nicandrazumpti* Kulzer, 1951, by original designation. Status: valid subgenus of *Nicandra* Fairmaire, 1888 in Blaptinae: Pedinini: Helopinina.

*Heterophaga* Dejean, 1834: 199 [F]. Type species: **fixed herein** (ICZN 1999, Article 70.3) as *Opatrumlaevigatum* Fabricius, 1781, misidentified as *Tenebriomauritanicus* Linnaeus, 1764 in the subsequent designation of Duponchel (1845b: 601). Status: junior synonym of *Alphitobius* Stephens, 1829 in Tenebrioninae: Alphitobiini. Synonymy: Wollaston (1854: 498). Note: see Spilman (1972: 32) for the history of the misidentification.

*Heterophyllus* Gemminger in Gemminger and Harold, 1870: 1955 [M]. Type species [automatic]: *Heterophyluschrysomelinus* Klug, 1833, by monotypy. Status: junior synonym of *Heterophylus* Klug, 1833 in Diaperinae: Diaperini: Diaperina. Note: unjustified emendation of *Heterophylus* Klug, 1833, not in prevailing usage.

*Heterophylus* Klug, 1833: 91 [M]. Type species: *Heterophyluschrysomelinus* Klug, 1833, by monotypy. Status: valid genus in Diaperinae: Diaperini: Diaperina.

*Heterophylus* Mulsant & Rey, 1859c: 7 [M]. Type species: *Heliopatespicipes* Faldermann, 1837, by subsequent designation (Gebien 1938a: 397). Status: senior synonym of *Dissonomus* Jacquelin du Val, 1861 in Tenebrioninae: Dissonomini. Note: junior homonym of *Heterophylus* Klug, 1833 [Tenebrionidae: Diaperinae: Diaperini: Diaperina].

*Heteropromus* Blaisdell, 1909: 179 [M]. Type species: *Eleodesvetorator* Horn, 1874, by monotypy. Status: valid subgenus of *Eleodes* Eschscholtz, 1829 in Blaptinae: Amphidorini.

*Heteropsectropus* Kaszab, 1941c: 33, 34 [M]. Type species: *Heteropsectropusaenescens* Kaszab, 1941, by original designation. Status: valid genus in Blaptinae: Platynotini: Eurynotina.

*Heteropus* Laporte, 1840: 221 [M]. Type species: *Heteropusholosericeus* Laporte, 1840, by monotypy. Status: junior synonym of *Blapstinus* Dejean, 1821 in Blaptinae: Opatrini: Blapstinina. Synonymy: Lacordaire (1859a: 250). Note: junior homonym of *Heteropus* Palisot de Beauvois, 1820 [Mammalia].

*Heteroscelis* Agassiz, 1846b: 181, 266 [F]. Type species [automatic]: *Adesmiapulcherrima* Solier, 1835 (= *Adesmiaaudouini* Solier, 1835), by subsequent designation (Hope 1841: 118). Status: junior synonym of *Oteroscelis* Solier, 1835 in Pimeliinae: Adesmiini. Note: unjustified emendation of *Oteroscelis* Solier, 1835, not in prevailing usage; junior homonym of *Heteroscelis* Latreille, 1828 [Coleoptera: Tenebrionidae: Blaptinae: Platynotini: Platynotina].

*Heteroscelis* Latreille, 1828: 574 [F]. Type species: *Blapsdentipes* Fabricius, 1794, by subsequent designation (Blanchard 1844: pl. 48). Status: senior synonym of *Anomalipus* Guérin-Méneville, 1831 in Blaptinae: Platynotini: Platynotina. Synonymy: Lacordaire (1859a: 257). Note: nomen oblitum (see Bouchard and Bousquet 2020a: 101); this genus used to be credited to Latreille (1829a: 18) and treated as a junior homonym of *Heteroscelis* Latreille, 1829 [Hemiptera] until bibliographic research determined that the tenebrionid name was made available earlier by Latreille (1828: 574) and is in fact the senior homonym (see Bouchard and Bousquet 2020a: 101).

*Heterostrongylium* Kaszab, 1977b: 10, 27 [N]. Type species: *Strongyliumweiskei* Gebien, 1921, by original designation. Status: valid genus in Stenochiinae: Stenochiini.

*Heterotarsus* Latreille, 1829a: 26 [M]. Type species: *Heterotarsustenebrioides* Guérin-Méneville, 1831, by subsequent monotypy (Guérin-Méneville 1831b: pl. 30). Status: valid genus in Blaptinae: Opatrini: Heterotarsina. Note: originally proposed without included nominal species; Guérin-Méneville (1831b: pl. 30), by including the new species *Heterotarsustenebrioides* Guérin-Méneville, 1831 in association with this name, was the first author to subsequently and expressly include nominal species in *Heterotarsus* (ICZN 1999, Article 67.2.2).

*Hexagonochilus* Solier, 1851: 168 [M]. Type species: *Hexagonochilusdilaticollis* Solier, 1851, by original designation. Status: valid genus in Pimeliinae: Stenosini: Dichillina. Note: *Hexagonochilus* is an incorrect subsequent spelling of the original spelling *Hexagonocheilus*, first used by Imhoff (1856: 234), and is in prevailing usage; *Hexagonochilus* is deemed to be the correct original spelling (ICZN 1999, Article 33.3.1).

*Hexarhopalus* Fairmaire, 1891c: xix [M]. Type species: *Hexarhopalussculpticollis* Fairmaire, 1891, by monotypy. Status: valid genus and subgenus in Stenochiinae: Cnodalonini.

*Hexarhoptrum* Fairmaire, 1894a: 38 [N]. Type species [automatic]: *Hexarhopalussculpticollis* Fairmaire, 1891, by monotypy. Status: junior synonym of *Hexarhopalus* Fairmaire, 1891 in Stenochiinae: Cnodalonini. Note: the alternative original spelling *Hexaroptrum*, used by Fairmaire (1894a: 38), was rejected by Bečvář and Purchart (2008: 39) who acted as the First Revisers; unnecessary replacement name for *Hexarhopalus* Fairmaire, 1891.

*Hicetaon* Champion, 1885: 111 [M]. Type species: *Hicetaonfrontalis* Champion, 1885, by monotypy. Status: valid genus in Stenochiinae: Cnodalonini.

*Hidrosella* Koch, 1952b: 32 [F]. Type species: *Hidrosisincostata* Haag-Rutenberg, 1875, by original designation. Status: valid subgenus of *Machlopsis* Pomel, 1871 in Pimeliinae: Adelostomini.

*Hidrosis* Haag-Rutenberg, 1875a: 120 [F]. Type species: *Steiracrenatocostata* Redtenbacher, 1868, by subsequent designation (Löbl et al. 2008b: 121). Status: junior synonym of *Machlopsis* Pomel, 1871 in Pimeliinae: Adelostomini. Synonymy: Bedel (1887: 199).

*Himastethe* Koch, 1950b: 286 [F]. Type species: *Cyphostethegigantea* Koch, 1950, by original designation. Status: valid subgenus of *Cyphostethe* Marseul, 1866 in Pimeliinae: Tentyriini.

*Himatismus* Erichson, 1843: 253 [M]. Type species: *Himatismusmandibularis* Erichson, 1843, by subsequent designation (Gebien 1937a: 574). Status: valid subgenus of *Imatismus* Dejean, 1834 in Pimeliinae: Tentyriini.

*Hionthis* Miller, 1861: 174 [F]. Type species: *Hionthistentyrioides* Miller, 1861, by monotypy. Status: valid genus in Pimeliinae: Tentyriini.

*Hionthisoma* Gebien, 1937a: 609 [N]. Type species [automatic]: *Hionthisoccidentalis* Fairmaire, 1897, by monotypy. Status: junior synonym of *Hyonthosoma* Reitter, 1900 in Pimeliinae: Tentyriini. Note: unjustified emendation of *Hyonthosoma* Reitter, 1900, not in prevailing usage.

*Hipalmus* Bates, 1870: 269 [M]. Type species: *Tenebriocostatus* Guérin-Méneville, 1831, by original designation. Status: valid genus in Tenebrioninae: Tenebrionini. Note: the original combination of the name of the type species, *Tenebriocostatus* Guérin-Méneville, 1831, is a junior primary homonym of *Tenebriocostatus* Pallas, 1781.

*Hipomelus* Dejean, 1834: 181 [M]. Type species: *Sepidium vittatum* Fabricius, 1781, by subsequent designation (Hope 1841: 116). Status: junior synonym of *Trachynotus* Latreille, 1828 in Pimeliinae: Sepidiini: Trachynotina. Synonymy: Bousquet and Bouchard (2013a: 47).

*Hipponome* Laporte, 1840: 235 [F]. Type species: *Helopsazureus* Brullé, 1832, by monotypy. Status: senior synonym of *Raiboscelis* Allard, 1876 in Tenebrioninae: Helopini: Helopina. Synonymy: Seidlitz (1895: 754). Note: nomen oblitum (see Nabozhenko and Löbl 2009: 194).Note: the original combination of the name of the type species, *Helopsazureus* Brullé, 1832, is a junior primary homonym of *Helopsazureus* Germar, 1823.

*Hirsutosora* Pic, 1934a: 32 [F]. Type species: *Nemostirafortithorax* Pic, 1922, by monotypy. Status: valid subgenus of *Sora* Walker, 1859 in Lagriinae: Lagriini: Statirina.

*Hirtograbies* Koch, 1954a: 23 [M]. Type species: *Hirtograbiesoograbiesensis* Koch, 1954, by original designation. Status: valid genus in Blaptinae: Platynotini: Eurynotina.

*Hispanomelia* Mas-Peinado, Buckley, Ruiz & García-París, 2018: 541 [F]. Type species: *Pimeliamanchega* Lauffer, 1905, by original designation. Status: valid subgenus of *Pimelia* Fabricius, 1775 in Pimeliinae: Pimeliini.

*Histeromimus* Gahan, 1895: 288 [M]. Type species: *Histeromimusarabicus* Gahan, 1895, by monotypy. Status: valid genus in Pimeliinae: Erodiini.

*Histeromorphus* Kraatz, 1865: 6, 11 [M]. Type species: *Histeromorphusplicatus* Kraatz, 1865, by monotypy. Status: valid genus in Pimeliinae: Erodiini.

*Histeropsis* Chevrolat, 1878a: 221 [F]. Type species: *Platydemaamericana* Laporte & Brullé, 1831, by subsequent designation (Löbl et al. 2008a: 42). Status: junior synonym of *Platydema* Laporte & Brullé, 1831 in Diaperinae: Diaperini: Diaperina. Synonymy: Champion (1886: 181).

*Histiaea* Fairmaire, 1892a: 107 [F]. Type species: *Histiaeabidentula* Fairmaire, 1892, by monotypy. Status: valid subgenus of *Cheirodes* Gené, 1839 in Tenebrioninae: Melanimonini.

*Histrionotus* Koch, 1955a: 44 [M]. Type species: *Trachynotuslightfooti* Péringuey, 1892, by original designation. Status: valid genus in Pimeliinae: Sepidiini: Trachynotina.

*Holaniara* Fairmaire, 1871b: 43 [F]. Type species [automatic]: *Ulomapiceum* Melsheimer, 1846, by monotypy. Status: junior synonym of *Eutochia* J.L. LeConte, 1862 in Tenebrioninae: Ulomini. Note: replacement name for *Aniara* Melsheimer, 1853.

*Holdhausia* Reitter, 1906b: 125 [F]. Type species: *Cteniopuscrassus* Fairmaire, 1892, by monotypy. Status: valid genus in Alleculinae: Cteniopodini.

*Holeleodes* Blaisdell, 1937: 132 [M]. Type species: *Eleodesbeameri* Blaisdell, 1937 (= *Eleodeshepburni* Champion, 1884), by original designation. Status: junior synonym of *Steneleodes* Blaisdell, 1909 in Blaptinae: Amphidorini. Synonymy: Johnston (2015: 12).

*Holoblaps* Bauer, 1921: 233 [F]. Type species: none designated. Status: undetermined taxon in Blaptinae: Blaptini: Blaptina. Note: this genus was described before 1931 (ICZN 1999, Article 12.1); however, we could not find any nominal species that were subsequently and expressly included in *Holoblaps* and therefore no “originally included nominal species” could be used to fix the type species (ICZN 1999, Article 67.2.2).

*Holobrachium* Gebien, 1905: 256 [N]. Type species [automatic]: *Hoplobrachiumasperipenne* Fairmaire, 1886 (= *Helopsdentipes* Fabricius, 1781), by monotypy. Status: junior synonym of *Hoplobrachium* Fairmaire, 1886 in Tenebrioninae: Amarygmini. Note: unjustified emendation of *Hoplobrachium* Fairmaire, 1886, not in prevailing usage.

*Holobrachys* Fairmaire, 1869b: 233 [M]. Type species: *Holobrachysheterocerus* Fairmaire, 1869, by monotypy. Status: valid genus in Stenochiinae: Cnodalonini.

*Hologenosis* Deyrolle, 1867: 81, 82 [F]. Type species: *Hologenosislacerata* Deyrolle, 1867, by monotypy. Status: valid subgenus of *Zophosis* Latreille, 1802 in Pimeliinae: Zophosini.

*Holostrongylium* Kaszab, 1977b: 10, 20 [N]. Type species: *Strongyliumgravidum* Mäklin, 1867, by original designation. Status: valid genus in Stenochiinae: Stenochiini.

*Homala* Eschscholtz, 1831: 5, 6 [F]. Type species: *Homalapolita* Eschscholtz, 1831, by monotypy. Status: valid genus in Pimeliinae: Tentyriini.

*Homalapipleurus* Español, 1957a: 166, 167 [M]. Type species: *Hegetergonzalezi* Español, 1957, by original designation. Status: valid subgenus of *Hegeter* Latreille, 1802 in Pimeliinae: Tentyriini.

*Homaleis* Rye, 1879: 62 [M]. Type species [automatic]: *Helopscongener* Reiche, 1861, by subsequent designation (Nabozhenko 2008: 38). Status: junior synonym of *Odocnemis* Allard, 1876 in Tenebrioninae: Helopini: Cylindrinotina. Note: unjustified emendation of *Omaleis* Allard, 1877, not in prevailing usage.

*Homalinota* Koch, 1950a: 66 [F]. Type species [automatic]: *Homalaagona* Fairmaire, 1884, by subsequent designation (Gebien 1937a: 610). Status: valid genus in Pimeliinae: Tentyriini. Note: replacement name for *Homalopsis* Lesne, 1922.

*Homalopsis* Lesne, 1922: 675 [F]. Type species: *Homalaagona* Fairmaire, 1884, by subsequent designation (Gebien 1937a: 610). Status: senior synonym of *Homalinota* Koch, 1950 in Pimeliinae: Tentyriini. Note: junior homonym of *Homalopsis* Kuhl & Hasselt, 1822 [Reptilia].

*Homalus* Rye, 1878: 69 [M]. Type species [automatic]: *Helopscongener* Reiche, 1861, by subsequent designation (Nabozhenko 2008: 38). Status: junior synonym of *Odocnemis* Allard, 1876 in Tenebrioninae: Helopini: Cylindrinotina. Note: unjustified emendation of *Omaleis* Allard, 1876 (as “*Omalus*”); junior homonym of *Homalus* Agassiz, 1846 [Hymenoptera].

*Homebius* Endrödy-Younga, 1989: 124 [M]. Type species: *Homebiuskaszabi* Endrödy-Younga, 1989, by original designation. Status: valid genus in Pimeliinae: Cryptochilini: Homebiina.

*Homocyrtus* Dejean, 1834: 211 [M]. Type species [automatic]: *Cyphonotusdromedarius* Guérin-Méneville, 1831, by monotypy. Status: valid genus in Tenebrionidae: incertae sedis. Note: replacement name for *Cyphonotus* Guérin-Méneville, 1831; based on a recent assessment of adult and larval morphological characters, Matthews and Lawrence (2015: 290) determined that this genus could not be placed in any known tribe and that the status of *Homocyrtus* should be regarded as Tenebrionidae incertae sedis.

*Homoeocamaria* Blair, 1919b: 75 [F]. Type species [automatic]: *Homoeogenuslaticornis* C.O. Waterhouse, 1882, by monotypy. Status: junior synonym of *Borneocamaria* Pic, 1917 in Stenochiinae: Cnodalonini. Note: replacement name for *Homoeogenus* C.O. Waterhouse, 1882.

*Homoeogenus* C.O. Waterhouse, 1882a: 174 [M]. Type species: *Homoeogenuslaticornis* C.O. Waterhouse, 1882, by monotypy. Status: senior synonym of *Borneocamaria* Pic, 1917 in Stenochiinae: Cnodalonini. Synonymy: Gebien (1942a: 323). Note: junior homonym of *Homoeogenus* C.O. Waterhouse, 1880 [Coleoptera: Psephenidae].

*Homoeonota* Fairmaire in Fairmaire et al., 1882: 63 [F]. Type species: *Homoeonotasubopaca* Fairmaire, 1882, by original designation. Status: valid genus in Pimeliinae: Tentyriini.

*Homopsis* Semenov, 1893: 258, 263 [F]. Type species: *Homopsisgrumi* Semenov, 1893, by monotypy. Status: valid genus in Pimeliinae: Pimeliini.

*Homoropsis* Fairmaire, 1886a: 450 [F]. Type species: *Homoropsisustulata* Fairmaire, 1886, by monotypy. Status: valid genus in Alleculinae: incertae sedis.

*Homotrysis* Pascoe, 1866a: 489 [F]. Type species: *Alleculatristis* Germar, 1848 (= *Alleculacarbonaria* Germar, 1848), by original designation. Status: valid genus in Alleculinae: Alleculini: Alleculina. Note: the First Reviser (*Homotrysis* Pascoe, 1866 versus *Hybrenia* Pascoe, 1866) is Champion (1897: 158).

*Hopatrinus* Agassiz, 1846b: 185, 260 [M]. Type species [automatic]: *Opatrumclathratum* Fabricius, 1787, by monotypy. Status: junior synonym of *Opatrinus* Dejean, 1821 in Blaptinae: Platynotini: Platynotina. Note: unjustified emendation of *Opatrinus* Dejean, 1821, not in prevailing usage.

*Hopatroides* Agassiz, 1846b: 185, 260 [M]. Type species [automatic]: *Opatroidespunctulatus* Brullé, 1832, by monotypy. Status: junior synonym of *Opatroides* Brullé, 1832 in Blaptinae: Opatrini: Opatrina. Note: unjustified emendation of *Opatroides* Brullé, 1832, not in prevailing usage.

*Hopatromorpha* Blackburn, 1907: 286 [F]. Type species [automatic]: *Opatrummurinum* Baudi di Selve, 1876 (= *Opatrinussetosus* Mulsant & Rey, 1853), by subsequent designation (Gebien 1938a: 399). Status: junior synonym of *Mesomorphus* Miedel, 1880 in Blaptinae: Opatrini: Opatrina. Note: unnecessary replacement name for *Mesomorphus* Miedel, 1880 (incorrectly attributed to “Reitter, 1904”).

*Hopatropteron* Reitter, 1889a: 701 [N]. Type species: *Hopatropteronsubcostatum* Reitter, 1889 (= *Heterotarsuscarinula* Marseul, 1876), by monotypy. Status: junior synonym of *Heterotarsus* Latreille, 1829 in Blaptinae: Opatrini: Heterotarsina. Synonymy: Seidlitz (1894: 413), Gebien (1911a: 473).

*Hopatrum* Agassiz, 1846b: 185, 260 [N]. Type species [automatic]: *Silphasabulosa* Linnaeus, 1758, by subsequent designation (Latreille 1810: 429). Status: junior synonym of *Opatrum* Fabricius, 1775 in Blaptinae: Opatrini: Opatrina. Note: unjustified emendation of *Opatrum* Fabricius, 1775, not in prevailing usage.

*Hoplambius* Reitter, 1904: 114 [M]. Type species: *Hoplarionmelambioides* (as “*melambioide*”) Fairmaire, 1893, by monotypy. Status: valid subgenus of *Melambius* Mulsant & Rey, 1854 in Blaptinae: Dendarini: Melambiina.

*Hoplariobius* Reitter, 1904: 115 [M]. Type species: *Micrositusdecurtatus* Fairmaire, 1884, by subsequent designation (Antoine 1942: 25). Status: valid subgenus of *Hoplarion* Mulsant & Rey, 1854 in Blaptinae: Dendarini: Melambiina.

*Hoplarion* Mulsant & Rey, 1854: 150 [N]. Type species: *Micrositustumidus* Mulsant & Rey, 1854, by monotypy. Status: valid genus and subgenus in Blaptinae: Dendarini: Melambiina.

*Hoplaspis* Motschulsky, 1858a: 113 [F]. Type species: *Hoplaspislamellicornis* Motschulsky, 1858, by subsequent designation (Crotch 1876: 418). Status: valid genus in Diaperinae: Diaperini: Diaperina. Note: the alternative original spelling *Hoplapsis*, used by Motschulsky (1858: 113), was rejected by Motschulsky (1868: 69) who acted as the First Reviser (ICZN 1999, Article 24.2.4); transferred from Cucujoidea: Erotylidae by Arrow (1909: 196).

*Hoplitoblaps* Fairmaire, 1889b: 26 [F]. Type species: *Hoplitoblapsfallaciosa* Fairmaire, 1889, by monotypy. Status: valid genus in Blaptinae: Blaptini: Blaptina.

*Hoplobrachium* Fairmaire, 1886c: 74 [N]. Type species: *Hoplobrachiumasperipenne* Fairmaire, 1886 (= *Helopsdentipes* Fabricius, 1781), by monotypy. Status: valid genus in Tenebrioninae: Amarygmini.

*Hoplocephala* Agassiz, 1846b: 185, 262 [F]. Type species [automatic]: *Ips haemorrhoidalis* Fabricius, 1787, by subsequent designation (Motschulsky 1845a: 80). Status: junior synonym of *Neomida* Latreille, 1829 in Diaperinae: Diaperini: Diaperina. Note: unjustified emendation of *Oplocephala* Laporte & Brullé, 1831, not in prevailing usage.

*Hoplochirus* Scudder, 1882: 153 [M]. Type species [automatic]: *Hoplonyxalleculoides* J. Thomson, 1858, by subsequent designation (Gebien 1943: 919). Status: junior synonym of *Hoplonyx* J. Thomson, 1858 in Tenebrioninae: Amarygmini. Note: unjustified emendation of *Oplocheirus* Klug, 1835, not in prevailing usage.

*Hoploedipinus* Kaszab, 1984: 355, 356 [M]. Type species: *Hoploedipusheterodoxus* Fairmaire, 1898, by original designation. Status: valid genus in Stenochiinae: Cnodalonini.

*Hoploedipus* Fairmaire, 1898d: 395 [M]. Type species: *Camarimenaarmipes* Fairmaire, 1882, by original designation. Status: valid genus in Stenochiinae: Cnodalonini.

*Hoplonyx* J. Thomson, 1858: 98 [M]. Type species: *Hoplonyxalleculoides* J. Thomson, 1858, by subsequent designation (Gebien 1943: 919). Status: valid genus and subgenus in Tenebrioninae: Amarygmini.

*Hoplopeltis* Fairmaire, 1894a: 22 [F]. Type species: *Hoplopeltistricornis* Fairmaire, 1894, by monotypy. Status: valid genus in Tenebrioninae: Alphitobiini.

*Hoploptera* Gemminger in Gemminger and Harold, 1870: 2037 [F]. Type species [automatic]: *Strongyliumserraticorne* Guérin-Méneville, 1834, by monotypy. Status: junior synonym of *Oploptera* Chevrolat, 1844 in Stenochiinae: Stenochiini. Note: unjustified emendation of *Oploptera* Chevrolat, 1844, not in prevailing usage.

*Hoplostira* Borchmann, 1921: 217, 225 [F]. Type species: *Hoplostirafemoralis* Borchmann, 1921, by original designation. Status: junior synonym of *Spinostatira* Pic, 1918 in Lagriinae: Lagriini: Statirina. Synonymy: Borchmann (1936: 247).

*Hoplostrongylium* Ardoin, 1965c: 1326 [N]. Type species: *Hoplostrongyliumghesquierei* Ardoin, 1965, by monotypy. Status: valid genus in Stenochiinae: Stenochiini.

*Horatoma* Solier, 1841a: 210, 264 [364] [F]. Type species: *Horatomaparvula* Solier, 1841, by original designation. Status: valid genus in Pimeliinae: Cryptochilini: Cryptochilina.

*Horatomella* Penrith & Endrödy-Younga, 1994: 6, 7 [F]. Type species: *Parapachynotelajohni* Koch, 1957, by original designation. Status: valid genus in Pimeliinae: Cryptochilini: Cryptochilina.

*Horatomodes* Haag-Rutenberg, 1872: 274, 305 [M]. Type species: *Horatomodesbatesi* Haag-Rutenberg, 1872, by monotypy. Status: junior synonym of *Horatoma* Solier, 1841 in Pimeliinae: Cryptochilini: Cryptochilina. Synonymy: Penrith and Endrödy-Younga (1994: 10).

*Horistelops* Gozis, 1910: 112 [M]. Type species: *Helopsassimilis* Küster, 1850, by original designation. Status: valid subgenus of *Nalassus* Mulsant, 1854 in Tenebrioninae: Helopini: Cylindrinotina.

*Hosohamudama* Masumoto, 1988b: 43 [F]. Type species: *Hosohamudamasasajii* Masumoto, 1988, by original designation. Status: valid genus in Lagriinae: Lagriini: Statirina.

*Houaphanica* Novák, 2020b: 470 [F]. Type species: *Houaphanicafera* Novák, 2020, by original designation. Status: valid genus in Alleculinae: Alleculini: Alleculina.

*Hovacula* Fairmaire, 1898a: 236 [F]. Type species: *Hovaculalineolata* Fairmaire, 1898, by monotypy. Status: valid genus in Alleculinae: incertae sedis. Note: placed in Alleculinae by Chatanay (1915a: 526).

*Hovadelium* Ardoin, 1961c: 34 [N]. Type species: *Hovadeliumdiscoidale* Ardoin, 1961, by original designation. Status: valid genus in Lagriinae: Laenini.

*Hovademulus* Iwan, 1996: 385, 390 [M]. Type species: *Selinuspunctipennis* Fairmaire, 1902, by original designation. Status: junior synonym of *Clastopus* Fairmaire, 1898 in Blaptinae: Platynotini: Platynotina. Synonymy: Iwan (2001: 500).

*Hovademus* Iwan, 1996: 385, 395 [M]. Type species: *Hovademusandringitrensis* Ardoin, 1974, by original designation. Status: valid genus in Blaptinae: Platynotini: Platynotina. Note: the name *Hovademus* was proposed earlier by Ardoin (1974a: 165) without a type species designation; the fact that *Hovademusandringitrensis* Ardoin, 1974 was listed as the “type” of *Hovademus* in the Zoological Record for the year 1974 (Anonymous in Staff of the Zoological Society of London 1979) does not represent a valid type species designation since the nomenclatural act is anonymous (ICZN 1999, Article 14).

*Hovarygmus* Fairmaire, 1898a: 234 [M]. Type species: *Hovarygmusinsularis* Fairmaire, 1898, by monotypy. Status: valid genus in Blaptinae: Opatrini: incertae sedis. Note: placed in Opatrini incertae sedis by Kamiński et al. (2021b: 151).

*Huilamus* Koch, 1953d: 79 [M]. Type species: *Huilamuswelwitschi* Koch, 1953, by original designation. Status: valid genus in Pimeliinae: Sepidiini: Molurina.

*Hummelinckia* Marcuzzi, 1954: 19 [F]. Type species: *Hummelinckiacaraibica* Marcuzzi, 1954, by monotypy. Status: valid genus in Blaptinae: Opatrini: Blapstinina.

*Hyalarthrodosis* Kaszab, 1979a: 74 [F]. Type species: *Arthrodosismonodi* Pierre, 1974, by original designation. Status: valid genus in Pimeliinae: Erodiini.

*Hyalerodius* Kaszab, 1979a: 80 [M]. Type species: *Hyalerodiusjirofti* Kaszab, 1979, by original designation. Status: valid genus in Pimeliinae: Erodiini.

*Hybocaulus* Fairmaire, 1895a: 27 [M]. Type species: *Hybocauluslaticornis* Fairmaire, 1895 (= *Porphyrhybaviolaceicolor* Fairmaire, 1877), by monotypy. Status: junior synonym of *Porphyrhyba* Fairmaire, 1877 in Stenochiinae: Cnodalonini. Synonymy: Ardoin (1956b: 89).

*Hybonotus* Dejean, 1834: 211 [M]. Type species: *Tetraphyllusformosus* Laporte & Brullé, 1831, by monotypy. Status: senior synonym of *Damatris* Laporte, 1840 in Stenochiinae: Cnodalonini. Synonymy: **new synonym** [PB]. Note: the type species of *Hybonotus* Dejean, 1834 is the same as the type species of *Damatris* Laporte, 1840 and therefore the two genera are objective synonyms; junior homonym of *Hybonotus* Klug, 1803 [Hymenoptera].

*Hyboproctus* Kolbe, 1897a: 241 [M]. Type species: *Hyboproctusnodifer* Kolbe, 1897, by subsequent designation (Gebien 1940: 1089). Status: valid genus in Stenochiinae: Cnodalonini.

*Hybrenia* Pascoe, 1866a: 489 [F]. Type species: *Hybreniavittata* Pascoe, 1866, by subsequent designation (Matthews and Bouchard 2008: 328). Status: junior synonym of *Homotrysis* Pascoe, 1866 in Alleculinae: Alleculini: Alleculina. Synonymy: Champion (1897: 158).

*Hydissus* Pascoe, 1869: 148 [M]. Type species: *Nyctobatesferonioides* Pascoe, 1866, by monotypy. Status: valid genus in Stenochiinae: Cnodalonini.

*Hydisus* Scudder, 1882: 167 [M]. Type species [automatic]: *Nyctobatesferonioides* Pascoe, 1866, by monotypy. Status: junior synonym of *Hydissus* Pascoe, 1869 in Stenochiinae: Cnodalonini. Note: unjustified emendation of *Hydissus* Pascoe, 1869, not in prevailing usage.

*Hylithus* Guérin-Méneville, 1834: 12 [M]. Type species: *Hylithustentyrioides* Guérin-Méneville, 1834, by monotypy. Status: valid genus in Pimeliinae: Edrotini.

*Hylocrinus* Casey, 1907: 289, 331 [M]. Type species: *Eurymetoponlungulum* J.L. LeConte, 1851, by original designation. Status: valid genus and subgenus in Pimeliinae: Edrotini.

*Hylonoma* Macquart, 1850: 183 [F]. Type species: *Alegoriadilatata* Laporte, 1840, by monotypy. Status: junior synonym of *Alegoria* Laporte, 1840 in Tenebrioninae: Ulomini. Synonymy: Lacordaire (1859a: 325).

*Hyloplonyx* Ardoin, 1963c: 716 [M]. Type species: *Hoplonyxmonophthalmus* J. Thomson, 1858, by original designation. Status: valid subgenus of *Hoplonyx* J. Thomson, 1858 in Tenebrioninae: Amarygmini.

*Hymenalia* Mulsant, 1856a: 48 [F]. Type species: *Cistelafusca* Illiger, 1794 (= *Cistelarufipes* Fabricius, 1792), by monotypy. Status: valid genus in Alleculinae: Alleculini: Alleculina.

*Hymenochara* Campbell, 1978: 435 [F]. Type species: *Mycetophilarufipes* J.E. LeConte, 1824, by original designation. Status: valid genus in Alleculinae: Alleculini: Mycetocharina.

*Hymenorus* Mulsant, 1852: 68 [M]. Type species: *Hymenorusdoublieri* Mulsant, 1852, by monotypy. Status: valid genus in Alleculinae: Alleculini: Alleculina. Note: the alternative original spelling *Hymenophorus* was corrected to *Hymenorus* in the “Emendanda” of the same work (p. 188), *Hymenorus* is considered to be the correct original spelling (ICZN 1999, Article 32.5.1.1); see Bousquet et al. (2015: 133).

*Hyocis* Pascoe, 1866a: 457 [M]. Type species: *Hyocisbakewellii* Pascoe, 1866, by original designation. Status: valid genus and subgenus in Diaperinae: Hyociini: Hyociina.

*Hyonthosoma* Reitter, 1900c: 89, 142 [N]. Type species: *Hionthisoccidentalis* Fairmaire, 1897, by monotypy. Status: valid genus in Pimeliinae: Tentyriini.

*Hypamarygmus* Gebien, 1904b: 27 [M]. Type species: *Hypamarygmuscoccinelloides* Gebien, 1904, by monotypy. Status: valid genus in Tenebrioninae: Amarygmini.

*Hypaulax* Bates, 1868: 259 [F]. Type species: *Hypaulaxmarginata* Bates, 1868, by subsequent designation (Gebien 1941: 333). Status: valid genus in Stenochiinae: Cnodalonini. Note: the First Reviser (*Hypaulax* Bates, 1868 versus *Chileone* Bates, 1868) is Carter (1914b: 46).

*Hyperamarygmus* Kaszab, 1964a: 291 [M]. Type species: *Hyperamarygmusantennalis* Kaszab, 1964, by original designation. Status: valid subgenus of *Amarygmus* Dalman, 1823 in Tenebrioninae: Amarygmini.

*Hyperchalca* Fairmaire, 1869b: 238 [F]. Type species: *Hyperchalcaaenescens* Fairmaire, 1869, by monotypy. Status: valid genus and subgenus in Stenochiinae: Stenochiini.

*Hypercossyphodes* Andreae, 1961: 205, 215 [M]. Type species: *Cossyphodesvandami* Andreae, 1961, by original designation. Status: junior synonym of *Cossyphodes* Westwood, 1851 in Pimeliinae: Cossyphodini: Cossyphodina. Synonymy: Schawaller (2013c: 362, implied by inclusion of *Cossyphodesvandami* Andreae, 1961 in Cossyphodes Westwood, 1851 without use of a subgenus rank).

*Hypermicrotelopsis* Koch, 1940b: 743 [F]. Type species: *Microtelopsisthibetana* Koch, 1940, by monotypy. Status: valid subgenus of *Microtelopsis* Koch, 1940 in Pimeliinae: Stenosini: Stenosina. Note: combined description of new genus-group taxon and a single new species (ICZN 1999, Article 13.4); we act as First Revisers and select *Microtelopsis* Koch, 1940 as the valid name for this genus instead of *Extetranosis* Koch, 1940 and *Hypermicrotelopsis* Koch, 1940.

*Hyperops* Eschscholtz, 1831: 5, 9 [M]. Type species: *Hyperopstagenioides* Eschscholtz, 1831, by monotypy. Status: valid genus and subgenus in Pimeliinae: Tentyriini.

*Hyperopsis* Bouchard & Bousquet, **new subgenus** [F]. Type species: *Pachyceracolasi* Koch, 1943, by **present designation**. Status: valid subgenus of *Hyperops* Eschscholtz, 1831 in Pimeliinae: Tentyriini. Note: Koch (1943a: 524, 536) introduced the new subgenus name *Hyperopsis* for five nominal species, but unfortunately did not designate a type species; the subgenusHyperopsis, which has been treated as valid since 1943, is therefore unavailable (ICZN 1999, Article 13.3); we hereby make the name available by selecting *Pachyceracolasi* Koch, 1943 as type species and referring to Koch (1943a: 524) for the character states that characterise and differentiate *Hyperopsis*.

*Hypoblaps* Kolbe, 1928: 200 [F]. Type species: *Blapsrotundata* Solier, 1848, by original designation. Status: junior synonym of *Blaps* Fabricius, 1775 in Blaptinae: Blaptini: Blaptina. Synonymy: Nabozhenko and Chigray (2020: 10).

*Hypocalis* Dejean, 1834: 206 [F]. Type species: *Hemiceraarcuata* Laporte & Brullé, 1831, by monotypy. Status: valid genus in Stenochiinae: Cnodalonini.

*Hypocilibe* Bates, 1872b: 275 [F]. Type species: *Hypocilibemacleayi* Bates, 1872, by monotypy. Status: junior synonym of *Nyctozoilus* Guérin-Méneville, 1831 in Tenebrioninae: Heleini: Cyphaleina. Synonymy: Carter (1911b: 139, with *Onosterrhus* Pascoe, 1866, a junior synonym of *Nyctozoilus* Guérin-Méneville, 1831).

*Hypocistela* Bates, 1879b: 482 [F]. Type species: *Hypocistelatenuipes* Bates, 1879, by monotypy. Status: valid genus in Alleculinae: Cteniopodini.

*Hypogena* Dejean, 1834: 199 [F]. Type species: *Tenebriobiimpressus* Latreille, 1833 (= *Peltisbrasilica* Perty, 1830), by monotypy. Status: valid genus in Tenebrioninae: Triboliini.

*Hypolaenopsis* Masumoto, 2001: 45 [F]. Type species: *Hypolaenopsisuenoi* Masumoto, 2001 (= *Laenananpingica* Schawaller, 2001), by original designation. Status: valid genus in Lagriinae: Laenini.

*Hypomelus* Solier, 1843: 4, 93, 126 [M]. Type species: *Hypomelusbicolor* Solier, 1843 (= *Helopsperonatus* Germar, 1823), by original designation. Status: valid genus in Pimeliinae: Sepidiini: Hypomelina.

*Hypophlaeus* Fabricius, 1790: 222 [M]. Type species: *Hypophlaeuscastaneus* Fabricius, 1790 (= *Corticeusunicolor* Piller & Mitterpacher, 1783), by subsequent designation (Curtis 1832: pl. 430). Status: junior synonym of *Corticeus* Piller & Mitterpacher, 1783 in Diaperinae: Hypophlaeini. Synonymy: Crotch (1870a: 47).

*Hypoprosodes* Reitter, 1909a: 122 [M]. Type species: *Prosodesminuta* Kraatz, 1881, by original designation. Status: valid subgenus of *Prosodes* Eschscholtz, 1829 in Blaptinae: Blaptini: Prosodina.

*Hypostatira* Fairmaire, 1889c: xlix [F]. Type species: *Hypostatiravariicolor* Fairmaire, 1889, by monotypy. Status: valid genus in Lagriinae: Lagriini: Statirina.

*Hypovinsonia* Ardoin, 1961a: 207 [F]. Type species: *Hypovinsoniaalbopilosa* Ardoin, 1961, by monotypy. Status: valid genus in Stenochiinae: Cnodalonini.

*Hypselops* Solier, 1851: 135 [M]. Type species: *Hypselopsoblongus* Solier, 1851, by subsequent designation (R. Lucas 1920: 347). Status: valid genus in Pimeliinae: Epitragini.

*Hypsosoma* Ménétriés, 1854: 30 [N]. Type species: *Hypsosomamongolicum* Ménétriés, 1854, by monotypy. Status: valid genus in Pimeliinae: Tentyriini.

*Hypulus* Rafinesque, 1815: 114 [M]. Type species [automatic]: *Tenebriocaeruleus* Linnaeus, 1758, by subsequent designation (Hope 1841: 133; see ICZN 2009, Opinion 2237). Status: junior synonym of *Helops* Fabricius, 1775 in Tenebrioninae: Helopini: Helopina. Note: unnecessary replacement name for *Helops* Fabricius, 1775; junior homonym of *Hypulus* Paykull, 1798 [Coleoptera: Melandryidae]; see entry for *Helops* Fabricius, 1775 for information regarding the identity of the type species.

*Hysterarthron* J. Thomson, 1864: 224 [N]. Type species: *Hysterarthroncollare* J. Thomson, 1864, by original designation. Status: valid genus in Lagriinae: Lagriini: Statirina.

*Iberomelia* Mas-Peinado, Buckley, Ruiz & García-París, 2018: 539 [F]. Type species: *Pimeliacastellana* Pérez Arcas, 1865, by original designation. Status: valid subgenus of *Pimelia* Fabricius, 1775 in Pimeliinae: Pimeliini.

*Ibnsaudia* Koch, 1941: 290 [F]. Type species: *Ibnsaudiapropheta* Koch, 1941, by monotypy. Status: junior synonym of *Thraustocolus* Kraatz, 1866 in Pimeliinae: Tentyriini. Synonymy: Kaszab (1981b: 341).

*Iccius* Champion, 1886: 147 [M]. Type species: *Icciuscephalotes* Champion, 1886, by subsequent designation (Gebien 1940: 760). Status: valid genus in Diaperinae: Diaperini: Adelinina.

*Idahelops* Keskin & Nabozhenko, 2012: 63 [M]. Type species: *Idahelopsalpagutae* Keskin & Nabozhenko, 2012, by original designation. Status: valid genus in Tenebrioninae: Helopini: Cylindrinotina.

*Idastrandiella* Strand, 1929: 23 [F]. Type species [automatic]: *Helopsmucoreus* Waltl, 1838, by monotypy. Status: junior synonym of *Ceratanisus* Gemminger, 1870 in Pimeliinae: Ceratanisini. Note: replacement name for *Apolites* Jacquelin du Val, 1861.

*Idatius* Fairmaire, 1906: 276 [M]. Type species: *Idatiusophtalmicus* Fairmaire, 1906, by monotypy. Status: valid genus in Alleculinae: incertae sedis. Note: placed in Alleculinae by Chatanay (1915a: 526).

*Idiesa* Reitter, 1893: 203, 245 [F]. Type species: *Diesiafischeri* Ménétriés, 1849, by subsequent designation (Gebien 1937a: 807). Status: valid genus in Pimeliinae: Pimeliini.

*Idiobates* Casey, 1891: 62 [M]. Type species: *Tenebriocastaneus* Knoch, 1801, by monotypy. Status: valid genus in Tenebrioninae: Tenebrionini.

*Idiopsis* Kaszab, 1981a: 78 [F]. Type species: *Idiopsisopaca* Kaszab, 1981, by original designation. Status: senior synonym of *Kocakia* Kaszab, 1985 in Pimeliinae: Edrotini. Note: junior homonym of *Idiopsis* Brauer & Bergenstamm, 1890 [Diptera].

*Idisia* Pascoe, 1866a: 452 [F]. Type species: *Idisiaornata* Pascoe, 1866, by monotypy. Status: valid genus in Pimeliinae: Idisiini.

*Idricus* Fairmaire, 1888c: 199 [M]. Type species: *Idricusdiabolicus* Fairmaire, 1888, by monotypy. Status: junior synonym of *Ametrocera* Fåhraeus, 1870 in Blaptinae: Pedinini: Helopinina. Synonymy: Péringuey (1904: 296).

*Iliodera* Skopin, 1961a: 396 [F]. Type species: *Microderadesertoides* Skopin, 1961, by original designation. Status: valid subgenus of *Microdera* Eschscholtz, 1831 in Pimeliinae: Tentyriini.

*Ilus* Champion, 1885: 117 [M]. Type species: *Ilusapicicornis* Champion, 1885, by monotypy. Status: valid genus in Stenochiinae: Cnodalonini.

*Ilyxerus* Pascoe, 1866a: 458 [M]. Type species: *Ilyxerusasper* Pascoe, 1866, by monotypy. Status: valid genus in Tenebrioninae: Toxicini: Dysantina.

*Imatismus* Dejean, 1834: 202 [M]. Type species: *Helopsfasciculatus* Fabricius, 1798, by monotypy. Status: valid genus and subgenus in Pimeliinae: Tentyriini.

*Immedia* Pascoe, 1882: 33 [F]. Type species: *Immediaocculta* Pascoe, 1882, by monotypy. Status: valid genus in Stenochiinae: Cnodalonini.

*Impressallecula* Pic, 1951: 12 [F]. Type species: *Impressalleculapurpureipes* Pic, 1951, by original designation. Status: valid genus in Alleculinae: Alleculini: Alleculina.

*Impressosora* Pic, 1952b: 254 [F]. Type species: *Impressosoranotaticollis* Pic, 1952, by monotypy. Status: valid genus and subgenus in Lagriinae: Lagriini: Statirina. Note: **new placement** [OM], previously included in Lagriinae: Lagriini: Lagriina.

*Indenicmosoma* Ardoin, 1964b: 688, 689 [N]. Type species: *Enicmosomaindochinense* Kaszab, 1940, by original designation. Status: valid genus in Lagriinae: Lupropini.

*Indeucolus* Kaszab, 1975b: 280, 282 [M]. Type species: *Indeucoluscostatus* Kaszab, 1975, by original designation. Status: junior synonym of *Eucolus* Mulsant & Rey, 1853 in Blaptinae: Platynotini: Platynotina. Synonymy: Iwan (1997: 258).

*Indianosis* Koch, 1941: 297 [F]. Type species: *Stenosiscapitata* Koch, 1941, by monotypy. Status: valid subgenus of *Stenosis* Herbst, 1799 in Pimeliinae: Stenosini: Stenosina.

*Indochillus* Koch, 1941: 300 [M]. Type species: *Indochilluscristatus* Koch, 1941, by monotypy. Status: valid genus in Pimeliinae: Stenosini: Dichillina.

*Indoprosodes* G.S. Medvedev, 2003: 690 [M]. Type species: *Prosodesboorpi* Kaszab, 1956, by original designation. Status: valid subgenus of *Prosodes* Eschscholtz, 1829 in Blaptinae: Blaptini: Prosodina.

*Indostola* G.S. Medvedev, 1991: 557 [F]. Type species: *Indostolapulchella* G.S. Medvedev, 1991, by original designation. Status: valid genus in Pimeliinae: Stenosini: Stenosina.

*Indricula* Novák, 2016c: 47 [F]. Type species: *Indriculaargynnis* Novák, 2016, by original designation. Status: valid genus in Alleculinae: Alleculini: Alleculina.

*Inscutoheliofugus* Freude, 1960a: 126, 130 [M]. Type species: *Heliofuguskuscheli* Freude, 1960, by original designation. Status: valid subgenus of *Heliofugus* Guérin-Méneville, 1831 in Stenochiinae: Cnodalonini.

*Insolitoplonyx* Bremer, 2014b: 178, 181 [M]. Type species: *Insolitoplonyxseorsus* Bremer, 2014, by original designation. Status: valid genus in Tenebrioninae: Amarygmini.

*Inspinogeton* Pic, 1937a: 174 [M]. Type species: *Cyriogetonimpressipennis* Pic, 1937, by monotypy. Status: valid subgenus of *Plesiophthalmus* Motschulsky, 1857 in Tenebrioninae: Amarygmini.

*Insulasida* Escalera, 1922b: 64 [F]. Type species: *Asidamoraguezi* Schaufuss, 1879, by subsequent designation (F. Soldati 2008: 33). Status: junior synonym of *Asida* Latreille, 1802 in Pimeliinae: Asidini. Synonymy: Viñolas and Cartagena (2005: 291).

*Iphicorynus* Jacquelin du Val, 1861: 299 [M]. Type species: *Pentaphyllusmelanophthalmus* Mulsant, 1854 (= *Nitidulachrysomeloides* Rossi, 1792), by monotypy. Status: junior synonym of *Pentaphyllus* Dejean, 1821 in Diaperinae: Diaperini: Diaperina. Synonymy: Gemminger in Gemminger and Harold (1870: 1956).

*Iphius* Dejean, 1834: 203 [M]. Type species: *Tenebrioserratus* Fabricius, 1775, by monotypy. Status: senior synonym of *Prioscelis* Hope, 1841 in Lagriinae: Pycnocerini. Synonymy: Imhoff (1856: 238). Note: junior homonym of *Iphius* Schönherr, 1823 [Coleoptera: Curculionidae].

*Iphthimera* Reitter, 1916a: 4 [F]. Type species: *Stenocararuficorne* Solier, 1835, by **present designation**. Status: junior synonym of *Metriopus* Solier, 1835 in Pimeliinae: Adesmiini. Synonymy: **new synonym** [PB]. Note: the type species of *Iphthimera* Reitter, 1916 is currently included in the genus *Metriopus* Solier, 1835 and for that reason *Iphthimera* is considered a junior synonym of Solier’s name.

*Iphthiminus* Spilman, 1973: 42 [M]. Type species: *Iphthinusitalicus* Truqui, 1857, by original designation. Status: valid genus in Stenochiinae: Cnodalonini.

*Iphthimulus* Reitter, 1920a: 16, 17 [M]. Type species: *Iphthinustruquii* Marseul, 1869, by monotypy. Status: valid genus in Stenochiinae: Cnodalonini.

*Iphthimus* Gemminger in Gemminger and Harold, 1870: 1977 [M]. Type species [automatic]: *Tenebriogigas* Linnaeus, 1763, by subsequent designation (Spilman 1973: 42). Status: junior synonym of *Mylaris* Pallas, 1781 in Stenochiinae: Cnodalonini. Note: unjustified emendation of *Iphthinus* Dejean, 1834, not in prevailing usage.

*Iphthinus* Dejean, 1834: 203 [M]. Type species: *Tenebriogigas* Linnaeus, 1763, by subsequent designation (Spilman 1973: 42). Status: junior synonym of *Mylaris* Pallas, 1781 in Stenochiinae: Cnodalonini. Synonymy: Chevrolat (1845b: 106, with *Nyctobates* Guérin-Méneville, 1834, a junior synonym of *Mylaris* Pallas, 1781), Spilman (1973: 42).

*Iranarthrodosis* Kaszab, 1959a: 334 [F]. Type species: *Arthrodosispfaundleri* Schuster, 1935, by original designation. Status: junior synonym of *Erodiontes* Reitter, 1914 in Pimeliinae: Erodiini. Synonymy: Kaszab (1979a: 88).

*Iranerodius* Kaszab, 1959a: 334 [M]. Type species: *Arthrodosisrichteri* Kaszab, 1957, by original designation. Status: valid genus in Pimeliinae: Erodiini.

*Iranolasiostola* Pierre, 1968: 1020 [F]. Type species: *Iranolasiostoladavatchii* Pierre, 1968, by original designation. Status: valid genus in Pimeliinae: Pimeliini.

*Iranopachyscelis* Pierre, 1968: 1027 [F]. Type species: *Iranopachysceliseghbali* Pierre, 1968 (= *Thripterapersica* Redtenbacher, 1850), by original designation. Status: valid genus in Pimeliinae: Pimeliini.

*Iranosodes* G.S. Medvedev, 1996: 605 [M]. Type species: *Prosodeskaszabi* G.S. Medvedev & Kabakov, 1996, by original designation. Status: valid subgenus of *Prosodes* Eschscholtz, 1829 in Blaptinae: Blaptini: Prosodina.

*Irianobates* Kaszab, 1986: 291 [M]. Type species: *Irianobateskrikkeni* Kaszab, 1986, by original designation. Status: valid genus in Stenochiinae: Cnodalonini.

*Isaminas* Champion, 1887: 266 [M]. Type species: *Isaminasgibbipennis* Champion, 1887, by subsequent designation (Gebien 1943: 401). Status: valid genus in Stenochiinae: Cnodalonini.

*Isarida* Pascoe, 1866a: 456 [F]. Type species: *Isaridatestacea* Pascoe, 1866 (= *Caediusfulvus* Mulsant & Rey, 1859), by monotypy. Status: junior synonym of *Caedius* Blanchard, 1845 in Blaptinae: Opatrini: Ammobiina. Synonymy: Gebien (1939: 466).

*Iscanus* Fauvel, 1904: 176 [M]. Type species: *Iscanuskuniensis* Fauvel, 1904, by monotypy. Status: valid genus in Lagriinae: Lupropini.

*Ischnarthron* Gebien, 1921b: 47 [N]. Type species: *Ischnarthronlongipes* Gebien, 1921, by monotypy. Status: valid genus in Diaperinae: Hypophlaeini.

*Ischnodactylus* Chevrolat, 1877: 173 [M]. Type species: *Ischnodactylusquadrioculatus* Chevrolat, 1877, by monotypy. Status: junior synonym of *Basides* Motschulsky, 1873 in Diaperinae: Diaperini: Diaperina. Synonymy: Schawaller (2004: 47, implied through placement of the type species of *Basides* Motschulsky, 1873 in *Ischnodactylus* Chevrolat, 1877). Note: the original spelling of the type species, *Ischnodactylusquadridentatus*, was corrected to *Ischnodactylusquadrioculatus* in the “Errata & Notes” of the same work (p. 178), *Ischnodactylusquadrioculatus* is considered to be the correct original spelling (ICZN 1999, Article 32.5.1.1).

*Isicerdes* Champion, 1885: 113 [M]. Type species: *Isicerdesoccultus* Champion, 1885, by subsequent designation (R. Lucas 1920: 353). Status: valid genus in Stenochiinae: Cnodalonini.

*Ismarus* Haag-Rutenberg, 1878: 104 [M]. Type species: *Ismarusgodeffroyi* Haag-Rutenberg, 1878, by monotypy. Status: senior synonym of *Simarus* Borchmann, 1909 in Alleculinae: Alleculini: Alleculina. Note: junior homonym of *Ismarus* Haliday, 1835 [Hymenoptera].

*Isocera* Borchmann, 1909a: 713 [F]. Type species [automatic]: *Isotomaemarginaticollis* Blanchard, 1842, by monotypy. Status: valid genus in Lagriinae: Lagriini: Statirina. Note: replacement name for *Isotoma* Blanchard, 1842.

*Isocerus* Dejean, 1821: 66 [M]. Type species: *Tenebriopurpurascens* Herbst, 1799 (= *Helopsferrugineus* Fabricius, 1798), by monotypy. Status: senior synonym of *Neoisocerus* Bouchard, Lawrence, Davies & Newton, 2005 in Blaptinae: Dendarini: Dendarina. Note: junior homonym of *Isocerus* Illiger, 1802 [Coleoptera: Cerambycidae].

*Isomira* Mulsant, 1856a: 52 [F]. Type species: *Chrysomelamurina* Linnaeus, 1758, by subsequent designation (C.G. Thomson 1859: 119). Status: valid genus and subgenus in Alleculinae: Alleculini: Gonoderina.

*Isomiropsis* Borchmann, 1942: 48 [F]. Type species: *Isomiropsiswittei* Borchmann, 1942, by original designation. Status: valid genus in Alleculinae: incertae sedis.

*Isoncophallus* Koch, 1954a: 55 [M]. Type species: *Isoncophalluszabroides* Koch, 1954, by original designation. Status: valid genus in Blaptinae: Platynotini: Eurynotina.

*Isonota* Fairmaire, 1887a: 171 [F]. Type species: *Isonotaopaca* Fairmaire, 1887, by monotypy. Status: junior synonym of *Homoeonota* Fairmaire, 1882 in Pimeliinae: Tentyriini. Synonymy: Koch (1962c: 254).

*Isopedus* Stein in Stein and Weise, 1877: 121 [M]. Type species: *Helopstenebrioides* Germar, 1813, **by present designation**. Status: junior synonym of *Odocnemis* Allard, 1876 in Tenebrioninae: Helopini: Cylindrinotina. Synonymy: Heyden et al. (1883: 136, with *Omaleis* Allard, 1876, a synonym of *Odocnemis* Allard, 1876). Note: the original combination of the name of the type species, *Helopstenebrioides* Germar, 1813, is a junior primary homonym of *Helopstenebrioides* Palisot de Beauvois, 1812.

*Isopteron* Hope, 1841: 112 [N]. Type species: *Isopteronaustrale* Hope, 1841, by original designation. Status: valid genus in Lagriinae: Adeliini. Note: combined description of a new genus-group taxon and a single new species (ICZN 1999, Article 12.2.6).

*Isopteroplonyx* Bremer, 2006: 7 [M]. Type species: *Amarygmustermitophilus* Lea, 1910, by monotypy. Status: valid genus in Tenebrioninae: Amarygmini.

*Isopterum* Agassiz, 1846b: 197 [N]. Type species [automatic]: *Isopteronaustrale* Hope, 1841, by original designation. Status: junior synonym of *Isopteron* Hope, 1841 in Lagriinae: Adeliini. Note: unjustified emendation of *Isopteron* Hope, 1841, not in prevailing usage.

*Isopus* Montrouzier, 1860: 299 [M]. Type species: *Isopusblanchardi* Montrouzier, 1860, by monotypy. Status: valid genus in Stenochiinae: Cnodalonini.

*Isostira* Pascoe, 1870: 97 [F]. Type species: *Isostiracrenata* Pascoe, 1870, by monotypy. Status: junior synonym of *Bradymerus* Perroud & Montrouzier, 1865 in Stenochiinae: Cnodalonini. Synonymy: Gebien (1921a: 253).

*Isotoma* Blanchard, 1842: pl. 15 [F]. Type species: *Isotomaemarginaticollis* Blanchard, 1842, by monotypy. Status: senior synonym of *Isocera* Borchmann, 1909 in Lagriinae: Lagriini: Statirina. Note: junior homonym of *Isotoma* Bourlet, 1839 [Collembola].

*Itagonia* Reitter, 1887a: 362 [F]. Type species: *Itagoniagnaptorinoides* Reitter, 1887, by monotypy. Status: valid genus in Blaptinae: Blaptini: Gnaptorinina.

*Italohelops* Español, 1961b: 295 [M]. Type species: *Parablopssubchalybaeus* Reitter, 1907, by monotypy. Status: valid genus in Tenebrioninae: Helopini: Helopina.

*Italomelia* Mas-Peinado, Buckley, Ruiz & García-París, 2018: 539 [F]. Type species: *Pimeliarugulosa* Germar, 1823, by original designation. Status: valid subgenus of *Pimelia* Fabricius, 1775 in Pimeliinae: Pimeliini.

*Itampolis* Koch, 1962a: 31, 143 [F]. Type species: *Itampolisoceanica* Koch, 1962, by original designation. Status: valid genus in Pimeliinae: Stenosini: Stenosina.

*Iugidorsum* Louw, 1979: 99, 102 [N]. Type species: *Iugidorsumcumstriis* Louw, 1979, by original designation. Status: valid genus in Pimeliinae: Sepidiini: Hypomelina.

*Ixalus* Gistel, 1848a: x [M]. Type species [automatic]: *Blapsexcavata* Fabricius, 1775, by subsequent designation (Hope 1841: 110). Status: junior synonym of *Platynotus* Fabricius, 1801 in Blaptinae: Platynotini: Platynotina. Note: unnecessary replacement name for *Platynotus* Fabricius, 1801.

*Jaklia* Novák, 2010: 180 [F]. Type species: *Jakliaserraticornis* Novák, 2010, by original designation. Status: valid genus in Alleculinae: Alleculini: Alleculina.

*Japetus* Reitter, 1904: 160 [M]. Type species: *Japetusmelanarius* Reitter, 1904 (= *Opatrummelanarium* Erichson, 1843), by monotypy. Status: junior synonym of *Trichosternum* Wollaston, 1861 in Blaptinae: Opatrini: Opatrina. Synonymy: Gebien (1905: 253, with *Trichopodus* [as *Trichopodum*] Mulsant & Rey, 1859, a senior synonym of *Trichosternum* Wollaston, 1861). Note: junior homonym of *Japetus* Stål, 1863 [Hemiptera].

*Javamarygmus* Pic, 1928a: 22 [M]. Type species: *Javamarygmustristis* Pic, 1928, by monotypy. Status: valid genus in Tenebrioninae: Amarygmini.

*Jintaium* Ren in Ren and Yu, 1999: 228 [N]. Type species: *Jintaiumsulcatum* Ren, 1999, by original designation. Status: valid genus in Blaptinae: Opatrini: Opatrina.

*Jophon* Champion, 1895a: 224 [M]. Type species: *Jophonmyrmecophilus* Champion, 1895, by monotypy. Status: valid genus in Alleculinae: Alleculini: Alleculina. Note: *Jophon* is an incorrect subsequent spelling of the original spelling *Iophon*, first used by Kolbe (1898: 601), in prevailing usage; *Jophon* is deemed to be the correct original spelling (ICZN 1999, Article 33.3.1), see Matthews and Bouchard (2008: 330).

*Julogenius* Reitter, 1906b: 138 [M]. Type species: *Heliotaurusreichii* Mulsant, 1856, by subsequent designation (Novák and Pettersson 2008: 333). Status: valid subgenus of *Heliotaurus* Mulsant, 1856 in Alleculinae: Cteniopodini.

†*Jurallecula* L.N. Medvedev, 1969: 124 [F]. Type species: *Juralleculagrossa* L.N. Medvedev, 1969, by original designation. Status: valid genus in Alleculinae: Alleculini: Alleculina. Note: described from Upper Jurassic deposits (Kazakhstan).

*Kabakoviella* Kaszab, 1980c: 205 [F]. Type species: *Kabakoviellamenephiloides* Kaszab, 1980, by original designation. Status: valid genus in Stenochiinae: Cnodalonini.

*Kaindilagria* Merkl, 1988a: 138 [F]. Type species: *Kaindilagriaforcipata* Merkl, 1988, by original designation. Status: valid genus in Lagriinae: Lagriini: Lagriina.

*Karroocara* Koch, 1952a: 176 [N]. Type species: *Stenocaragibbipenne* Haag-Rutenberg, 1875, by original designation. Status: junior synonym of *Stenodesia* Reitter, 1916 in Pimeliinae: Adesmiini. Synonymy: Penrith (1979: 70, 85).

*Kaszaba* Matthews & Doyen, 1989: 40 [F]. Type species: *Tenebriocorvinus* Erichson, 1842, by original designation. Status: valid genus in Stenochiinae: Cnodalonini.

*Kaszabadelium* Watt, 1992: 29 [N]. Type species: *Adeliumaucklandicum* Broun, 1880, by original designation. Status: valid genus in Lagriinae: Adeliini.

*Kaszabiella* Koch, 1943b: 774, 885 [F]. Type species: *Thalpophilasubhemisphaerica* Koch, 1943, by monotypy. Status: valid subgenus of *Thalpophilodes* Strand, 1942, in Pimeliinae: Tentyriini.

*Kaszabochillus* Fouquè, 2015: 228, 240 [M]. Type species: *Indochillusandamanus* Kaszab, 1981, by original designation. Status: valid subgenus of *Pseudochillus* Fouquè, 2015 in Pimeliinae: Stenosini: Dichillina.

*Kaszaboscelis* Löbl & Merkl, 2003: 246 [F]. Type species: *Tenebriohypolithus* Pallas, 1781, by original designation. Status: junior synonym of *Platyscelis* Latreille, 1818 in Blaptinae: Platyscelidini. Synonymy: Egorov (2004: 605).

*Kaszabus* Freude, 1967: 194 [M]. Type species: *Kaszabusaurulentiformis* Freude, 1967, by original designation. Status: valid genus in Pimeliinae: Epitragini.

*Kawiria* Schuster, 1935: 26 [F]. Type species: *Kawiriagabrieli* Schuster, 1935, by monotypy. Status: valid genus in Pimeliinae: Pimeliini.

*Kershawia* Lea, 1905: 379 [F]. Type species: *Kershawiarugiceps* Lea, 1905, by monotypy. Status: valid genus in Lagriinae: Belopini.

*Kirgisomira* Weise, 1974: 71 [F]. Type species: *Isomiraophthalmica* Seidlitz, 1896, by original designation. Status: junior synonym of *Asiomira* Dubrovina, 1973 in Alleculinae: Alleculini: Gonoderina. Synonymy: Muche (1981: 157).

*Klapperichia* Kaszab, 1954: 249 [F]. Type species: *Klapperichiamirabilis* Kaszab, 1954 (= *Tenebriocephalonthoracicum* Pic, 1925), by original designation. Status: junior synonym of *Tenebriocephalon* Pic, 1925 in Pimeliinae: Ceratanisini. Synonymy: Kaszab (1983a: 130).

*Klewaria* Reitter, 1910: 20 [F]. Type species: *Klewariacolydiiformis* Reitter, 1910, by monotypy. Status: valid genus in Pimeliinae: Klewariini.

*Knausia* Fall, 1931: 15 [F]. Type species: *Knausiacrassicornis* Fall, 1931, by monotypy. Status: valid genus in Alleculinae: Alleculini: Alleculina.

*Kocakia* Kaszab, 1985: 54 [F]. Type species [automatic]: *Idiopsisopaca* Kaszab, 1981, by original designation. Status: valid genus in Pimeliinae: Edrotini. Note: replacement name for *Idiopsis* Kaszab, 1981.

*Kocheria* Antoine, 1946: 25, 30 [F]. Type species: *Arthrodeisrungsi* Español, 1943, by monotypy. Status: valid subgenus of *Arthrodeis* Solier, 1834 in Pimeliinae: Erodiini.

*Kochogaster* Kamiński & Raś, 2011: 654 [F]. Type species [automatic]: *Anchophthalmusimpressicollis* Fairmaire, 1897, by original designation. Status: valid subgenus of *Anchophthalmus* Gerstaecker, 1854 in Blaptinae: Platynotini: Platynotina. Note: replacement name for *Cosmogaster* Koch, 1956.

*Kochotella* Bouchard & Bousquet, **new replacement name** [F]. Type species [automatic]: *Millotellamicrocornis* Koch, 1962, by original designation. Status: valid genus in Pimeliinae: Asidini. Note: replacement name for *Millotella* Koch, 1962.

*Kokeniella* Reitter, 1906a: 41 [F]. Type species: *Kokeniellamesostenoides* Reitter, 1906, by subsequent designation (Gebien 1937a: 626). Status: valid genus in Pimeliinae: Tentyriini.

*Kombacula* Novák, 2012: 271 [F]. Type species: *Kombaculakantneri* Novák, 2012, by original designation. Status: valid genus in Alleculinae: Alleculini: Alleculina.

*Koneus* Giraldo-Mendoza & Flores, 2019: 94 [M]. Type species: *Prohylithusperuanus* Kaszab, 1981, by original designation. Status: valid genus in Pimeliinae: Edrotini.

*Kralia* Novák, 2013: 500 [F]. Type species: *Kraliaminshanica* Novák, 2013, by original designation. Status: valid genus in Alleculinae: Alleculini: Gonoderina.

*Krollus* R. Lucas, 1920: 357 [M]. Type species [automatic]: *Homoeogenuslaticornis* C.O. Waterhouse, 1882, by monotypy. Status: junior synonym of *Borneocamaria* Pic, 1917 in Stenochiinae: Cnodalonini. Note: replacement name for *Homoeogenus* C.O. Waterhouse, 1882.

*Ksukolcula* Novák, 2017a: 168 [F]. Type species: *Ksukolculahesperia* Novák, 2017, by original designation. Status: valid genus in Alleculinae: Alleculini: Alleculina.

*Kuhitangia* G.S. Medvedev, 1962: 1184 [F]. Type species: *Kuhitangiakryzhanovskii* G.S. Medvedev, 1962, by original designation. Status: valid genus in Kuhitangiinae: Kuhitangiini.

*Kuschelus* Kaszab, 1982b: 112 [M]. Type species: *Kuscheluslathridioides* Kaszab, 1982, by original designation. Status: valid genus in Lagriinae: Lupropini.

*Labetis* C.O. Waterhouse, 1879a: 267 [F]. Type species: *Labetistibialis* C.O. Waterhouse, 1879, by monotypy. Status: valid genus in Alleculinae: Alleculini: Mycetocharina.

*Lachna* Billberg, 1820: 35 [F]. Type species: *Chrysomelahirta* Linnaeus, 1758, by subsequent designation (Merkl 2006: 223). Status: junior synonym of *Lagria* Fabricius, 1775 in Lagriinae: Lagriini: Lagriina. Synonymy: Lawrence and Newton (1995: 893).

*Lachnodactylus* Seidlitz, 1898a: 838 [M]. Type species [automatic]: *Lachnopusdigitalis* Seidlitz, 1894, by monotypy. Status: valid genus in Pimeliinae: Lachnogyini: Lachnodactylina. Note: replacement name for *Lachnopus* Seidlitz, 1894.

*Lachnoderes* Mulsant & Rey, 1859a: 70, 102 [M]. Type species: *Pedonoecespubescens* G.R. Waterhouse, 1845, by monotypy. Status: junior synonym of *Blapstinus* Dejean, 1821 in Blaptinae: Opatrini: Blapstinina. Synonymy: Gemminger in Gemminger and Harold (1870: 1923, with *Pedonoeces* G.R. Waterhouse, 1845, a junior synonym of *Blapstinus* Dejean, 1821); Aalbu and Triplehorn (1991: 170).

*Lachnogya* Ménétriés, 1849: 228 [F]. Type species: *Lachnogyasquamosa* Ménétriés, 1849, by monotypy. Status: valid genus in Pimeliinae: Lachnogyini: Lachnogyina.

*Lachnopus* Seidlitz, 1894: 476 [M]. Type species: *Lachnopusdigitalis* Seidlitz, 1894, by monotypy. Status: senior synonym of *Lachnodactylus* Seidlitz, 1898 in Pimeliinae: Lachnogyini: Lachnodactylina. Note: junior homonym of *Lachnopus* Schönherr, 1840 [Coleoptera: Curculionidae].

*Laena* Dejean, 1821: 64 [F]. Type species: **fixed herein** (ICZN 1999, Article 70.3) as *Scaurusviennensis* Sturm, 1807, misidentified as *Helopspimelia* Fabricius, 1787 in the original designation by monotypy in Dejean (1821). Status: valid genus in Lagriinae: Laenini. Note: the type species “*Helopspimelia* Fabricius” was first established by monotypy (ICZN 1999, Article 68.3); Bouchard and Bousquet (2020b: 7) noted that the only valid species originally included in *Laena*, *Helopspimelia* Fabricius, 1787, is currently considered a valid species in the genus *Penthe* Newman, 1838 [Coleoptera: Tetratomidae] and therefore the taxonomic species involved, *Helopspimelia* Fabricius sensu Dejean 1821 (= *Scaurusviennensis* Sturm, 1807) is fixed here as the type species of *Laena* Dejean, 1821 according to the requirements of Article 70.3.2 (ICZN 1999).

*Lagria* Fabricius, 1775: 124 [F]. Type species: *Chrysomelahirta* Linnaeus, 1758, by subsequent designation (Latreille 1810: 429). Status: valid genus and subgenus in Lagriinae: Lagriini: Lagriina.

*Lagriallecula* Pic, 1920b: 19 [F]. Type species: *Lagrialleculaaeneipennis* Pic, 1920, by monotypy. Status: valid genus in Alleculinae: incertae sedis.

*Lagriella* Borchmann, 1916a: 61 [F]. Type species: *Lagriamima* Borchmann, 1916, by original designation. Status: valid subgenus of *Lagria* Fabricius, 1775 in Lagriinae: Lagriini: Lagriina.

*Lagrimina* Fairmaire, 1894g: 675 [F]. Type species: *Lagriminastrigipennis* Fairmaire, 1894 (= *Porrolagrianuda* Kolbe, 1883), by monotypy. Status: junior synonym of *Porrolagria* Kolbe, 1883 in Lagriinae: Lagriini: Lagriina. Synonymy: Kolbe (1897a: 250).

*Lagriocera* Fairmaire, 1896a: 41 [F]. Type species: *Lagrioceracavicornis* Fairmaire, 1896, by monotypy. Status: junior synonym of *Xanthalia* Fairmaire, 1894 in Lagriinae: Lagriini: Statirina. Synonymy: Merkl (2004: 285).

*Lagriodema* Borchmann, 1930a: 442, 524 [F]. Type species: *Nemostiragestroi* Borchmann, 1910, by subsequent designation (Borchmann 1936: 458). Status: valid genus in Lagriinae: Lagriini: Statirina.

*Lagriodes* Borchmann, 1930a: 405, 432 [M]. Type species: *Heterogriaarmigera* Borchmann, 1930, by monotypy. Status: junior synonym of *Xanthalia* Fairmaire, 1894 in Lagriinae: Lagriini: Statirina. Synonymy: Merkl (2004: 285).

*Lagriogonia* Fairmaire, 1891e: ccxvii [F]. Type species: *Lagriogoniahumerosa* Fairmaire, 1891, by monotypy. Status: valid genus in Lagriinae: Lagriini: Statirina.

*Lagriola* Kirsch, 1874: 409 [F]. Type species: *Lagriolaoperosa* Kirsch, 1874, by subsequent designation (Bousquet et al. 2018: 33). Status: junior synonym of *Paratenetus* Spinola, 1845 in Lagriinae: Goniaderini. Synonymy: Gebien (1922b: 268), Matthews and Lawrence (2015: 311).

*Lagriomima* Pic, 1934a: 31 [F]. Type species: *Nemostiramaxima* Pic, 1912, by monotypy. Status: valid genus in Lagriinae: Lagriini: Statirina.

*Lagriomima* Pic, 1950: 11 [F]. Type species: *Lagriomimaalbolineata* Pic, 1950, by monotypy. Status: junior synonym of *Neogria* Borchmann, 1911 in Lagriinae: Lagriini: Lagriina. Synonymy: **new synonym** [OM]. Note: based on the description, the type species belongs to *Neogria* Borchmann, 1911 and is probably synonymous with the valid species *Neogriacyanipennis* Borchmann, 1911, therefore, *Lagriomima* Pic, 1950 is proposed as a new synonym of *Neogria*; junior homonym of *Lagriomima* Pic, 1934 [Coleoptera: Tenebrionidae: Lagriinae: Lagriini: Statirina].

*Lagriopsis* Borchmann, 1916a: 49, 138 [F]. Type species: *Lagriopsisinsularis* Borchmann, 1916, by subsequent designation (Borchmann 1936: 146). Status: valid genus in Lagriinae: Lagriini: Lagriina.

*Lagriostira* Fairmaire, 1883b: 103 [F]. Type species: *Statirarufonitens* Fairmaire, 1883, by monotypy. Status: valid genus in Lagriinae: Lagriini: Statirina.

*Lagriostira* Kolbe, 1902b: 550 [F]. Type species: *Lagriostirahispida* Kolbe, 1902 (= *Lagriasubseriata* Reitter, 1880), by subsequent designation (Borchmann 1936: 224). Status: senior synonym of *Alagria* Borchmann, 1916 in Lagriinae: Lagriini: Lagriina. Note: junior homonym of *Lagriostira* Fairmaire, 1883 [Coleoptera: Tenebrionidae: Lagriinae: Lagriini: Statirina].

*Lamperos* Allard, 1876a: 4 [M]. Type species: *Helopsmicans* Fabricius, 1798, by subsequent designation (Nabozhenko and Löbl 2008: 256). Status: junior synonym of *Tarpela* Bates, 1870 in Tenebrioninae: Helopini: Helopina. Synonymy: Champion (1887: 288).

*Lamprobothris* Fairmaire, 1887b: 302 [F]. Type species: *Lamprobothrisattenuata* Fairmaire, 1887 (= *Praogenafossulata* Müller, 1887), by monotypy. Status: junior synonym of *Praeugena* Laporte, 1840 in Tenebrioninae: Praeugenini. Synonymy: De Moor (1970: 42).

*Lamprocrypticus* Español, 1950: 127 [M]. Type species: *Crypticusalpinus* Comolli, 1837, by original designation. Status: valid genus in Diaperinae: Crypticini.

*Lanhsia* Shibata, 1980: 63, 64 [F]. Type species: *Lanhsiabucca* Shibata, 1980, by original designation. Status: valid genus in Tenebrioninae: Bolitophagini.

*Laonicus* Haag-Rutenberg, 1878: 100 [M]. Type species: *Laonicuspilosus* Haag-Rutenberg, 1878, by subsequent designation (R. Lucas 1920: 361). Status: junior synonym of *Platyphanes* Westwood, 1849 in Tenebrioninae: Heleini: Cyphaleina. Synonymy: Matthews (1992: 454).

*Laoscapha* Schawaller, 2016: 440 [F]. Type species: *Laoscaphaphoupanensis* Schawaller, 2016, by original designation. Status: valid genus in Diaperinae: Scaphidemini.

*Laosocryptobates* Pic, 1928a: 25 [M]. Type species: *Laosocryptobatestuberculatus* Pic, 1928, by monotypy. Status: junior synonym of *Hexarhopalus* Fairmaire, 1891 in Stenochiinae: Cnodalonini. Synonymy: Bečvář and Purchart (2008: 39).

*Laraliprosodes* Bogatchev, 1947: 513 [M]. Type species: *Prosodeslar* Bogatchev, 1947, by original designation. Status: junior synonym of *Dineria* Motschulsky, 1860 in Blaptinae: Blaptini: Blaptina. Synonymy: G.S. Medvedev and Iwan (2006: 617).

*Lariversius* Blaisdell, 1947: 59 [M]. Type species: *Lariversiustibialis* Blaisdell, 1947, by original designation. Status: valid genus in Blaptinae: Amphidorini.

*Lasiocnema* G.S. Medvedev, 1993: 110 [F]. Type species: *Lasiostolaheterogena* Fischer von Waldheim, 1844, by original designation. Status: valid subgenus of *Lasiostola* Dejean, 1834 in Pimeliinae: Pimeliini.

*Lasioderus* Mulsant & Rey, 1854: 13, 42 [M]. Type species: *Lasioderussulcipennis* Mulsant & Rey, 1854, by monotypy. Status: valid genus in Blaptinae: Dendarini: Melambiina.

*Lasiograna* G.S. Medvedev, 1993: 111 [F]. Type species: *Lasiostolainterrupta* Reitter, 1901, by original designation. Status: valid subgenus of *Lasiostola* Dejean, 1834 in Pimeliinae: Pimeliini.

*Lasiostola* Dejean, 1834: 179 [F]. Type species: *Tenebriopubescens* Pallas, 1781, by subsequent designation (Hope 1841: 118). Status: valid genus and subgenus in Pimeliinae: Pimeliini.

*Latacula* Campbell, 1971: 103 [F]. Type species: *Lataculabeckeri* Campbell, 1971, by original designation. Status: valid genus in Alleculinae: Alleculini: Alleculina.

*Latetribolium* Lepesme, 1943: 46 [N]. Type species: *Triboliumrisbeci* Lepesme, 1943 (= *Opatrumlaevigatum* Fabricius, 1781), by monotypy. Status: junior synonym of *Alphitobius* Stephens, 1829 in Tenebrioninae: Alphitobiini. Synonymy: Hinton (1948: 13, through synonymy of its type species with *Opatrumlaevigatum* Fabricius, 1781).

*Latheticus* C.O. Waterhouse, 1880: 147 [M]. Type species: *Latheticusoryzae* C.O. Waterhouse, 1880, by monotypy. Status: valid genus in Tenebrioninae: Triboliini.

*Latipleurosis* Penrith, 1977: 19, 204 [F]. Type species: *Zophosisbenguelensis* Deyrolle, 1867, by original designation. Status: valid subgenus of *Zophosis* Latreille, 1802 in Pimeliinae: Zophosini.

*Latorhascius* Pic, 1925b: 8 [M]. Type species: *Rhaciusbaeri* Pic, 1925, by monotypy. Status: valid subgenus of *Adelonia* Laporte, 1840 in Lagriinae: Belopini.

*Lawrenceus* Iwan, 1998b: 307 [M]. Type species: *Lawrenceuscapensis* Iwan, 1998, by original designation. Status: junior synonym of *Schelodontes* Koch, 1956 in Blaptinae: Platynotini: Platynotina. Synonymy: Iwan and Kamiński (2014: 171).

*Leanum* Uyttenboogaart, 1934: 29, 31 [N]. Type species: *Triboliummyrmecophilum* Lea, 1904, by monotypy. Status: junior synonym of *Tribolium* W.S. MacLeay, 1825 in Tenebrioninae: Triboliini. Synonymy: Hinton (1948: 21).

*Leaus* Matthews & Lawrence, 1992: 312 [M]. Type species: *Leaustasmanicus* Matthews & Lawrence, 1992, by original designation. Status: valid genus in Tenebrioninae: Trachelostenini. Note: transferred from Titaenini by Matthews and Lawrence (2015: 293).

*Lechinius* Blair, 1922: 561 [M]. Type species: *Lechiniuscatenulatus* Blair, 1922 (= *Cistelomorphafossulata* Pic, 1913), by monotypy. Status: valid subgenus of *Cteniopinus* Seidlitz, 1896 in Alleculinae: Cteniopodini.

*Lechinius* Borchmann, 1930b: 151 [M]. Type species: *Cistelomorphafossulata* Pic, 1913, by subsequent designation (Novák and Pettersson 2008: 330). Status: junior synonym of *Lechinius* Blair, 1922 in Alleculinae: Cteniopodini. Note: junior homonym of *Lechinius* Blair, 1922 [Coleoptera: Alleculinae: Cteniopodini].

*Lechius* Iwan, 1995c: 404 [M]. Type species: *Selinusabacoides* Fairmaire, 1902, by original designation. Status: valid genus in Blaptinae: Platynotini: Platynotina.

*Lechriomus* Morawitz, 1865: 21 [M]. Type species: *Akislucifuga* Adams, 1817, by subsequent designation (Chernei 2005: 102). Status: valid subgenus of *Cyphogenia* Solier, 1837 in Pimeliinae: Akidini.

*Leichenum* Dejean, 1834: 194 [N]. Type species: *Opatrumpictum* Fabricius, 1801, by monotypy. Status: valid genus in Blaptinae: Pedinini: Leichenina.

*Leichrodomorphus* Pic, 1921c: 6 [M]. Type species: *Leichrodomorphusbrevicornis* Pic, 1921, by subsequent designation (Pic 1922e: 135). Status: junior synonym of *Stethotrypes* Gebien, 1914 in Diaperinae: Leiochrinini. Synonymy: Gebien (1940: 431).

*Leiochrinus* Westwood, 1883: 68 [M]. Type species: *Leiochrinusfulvicollis* Westwood, 1883, by subsequent designation (W.F. Kirby 1885a: 89). Status: valid genus in Diaperinae: Leiochrinini.

*Leiochrodes* Westwood, 1883: 69 [M]. Type species: *Leiochrodesdiscoidalis* Westwood, 1883, by subsequent designation (W.F. Kirby 1885a: 89). Status: junior synonym of *Ades* Guérin-Méneville, 1857 in Diaperinae: Leiochrinini. Synonymy: Gebien (1911a: 388).

*Leiochrodinus* Kaszab, 1961a: 358, 365 [M]. Type species: *Leiochrodinustetraphyllus* Kaszab, 1961, by original designation. Status: valid genus in Diaperinae: Leiochrinini. Note: the alternative original spelling *Leichrodinus*, used by Kaszab (1961a: 365), was rejected by Papp and Seeno (1981: 53, 71) who acted as the First Revisers.

*Leiochrodontes* Kaszab, 1946a: 30, 200 [M]. Type species: *Leiochrodesmadurensis* Pic, 1918, by original designation. Status: valid genus in Diaperinae: Leiochrinini. Note: the earlier usage of *Leiochrodontes* by Gebien (1940: 755) was not accompanied by a description, a definition or a bibliographic reference to such a published statement and therefore is not available from that date.

*Leiochromimus* Ardoin, 1966: 187, 199 [M]. Type species: *Coccimarygmuspunctatus* Ardoin, 1966, by monotypy. Status: valid subgenus of *Coccimarygmus* Ardoin, 1966 in Tenebrioninae: Amarygmini.

*Leiochrota* Westwood, 1883: 70 [F]. Type species: *Leiochrinusuniformis* Westwood, 1883, by subsequent designation (W.F. Kirby 1885a: 90). Status: valid genus in Diaperinae: Leiochrinini.

*Leiochrotina* Westwood, 1883: 70, 76 [F]. Type species: *Leiochrinusindicus* Westwood, 1883, by monotypy. Status: junior synonym of *Crypsis* C.O. Waterhouse, 1877 in Diaperinae: Leiochrinini. Synonymy: Gebien (1940: 436).

*Leiopeplus* Broun, 1893b: 1160 [M]. Type species: *Helopsexpolitus* Broun, 1880, by original designation. Status: senior synonym of *Chrysopeplus* Gebien, 1942 in Stenochiinae: Cnodalonini. Note: junior homonym of *Leiopeplus* Murray, 1864 [Coleoptera: Nitidulidae].

*Leipopleura* Seidlitz, 1893: 342, 345 [F]. Type species: *Faustiaintegra* Reitter, 1887, by subsequent designation (Kaszab 1940a: 167). Status: valid subgenus of *Bioramix* Bates, 1879 in Blaptinae: Platyscelidini.

*Lelegeis* Champion, 1886: 209 [M]. Type species: *Lelegeisaeneipennis* Champion, 1886, by monotypy. Status: valid genus in Diaperinae: Diaperini: Diaperina.

*Leleupium* Kaszab, 1956b: 106 [N]. Type species: *Leleupiumsubcoecum* Kaszab, 1956, by original designation. Status: valid genus in Phrenapatinae: Penetini.

*Lemoultia* Chatanay, 1913b: 313 [F]. Type species: *Lemoultiascabripennis* Chatanay, 1913, by original designation. Status: valid genus in Tenebrioninae: Amarygmini.

*Lenkous* Kaszab, 1973b: 315 [M]. Type species: *Lenkousmyrmecophilus* Kaszab, 1973, by original designation. Status: valid genus in Stenochiinae: Cnodalonini.

*Lepidocaulinus* Schawaller, Masumoto & Merkl, 2013: 378 [M]. Type species: *Lepidocaulinusmirabilis* Schawaller, Masumoto & Merkl, 2013, by original designation. Status: valid genus in Stenochiinae: Cnodalonini.

*Lepidochora* Koch, 1952b: 36 [F]. Type species: *Lepidochoraeberlanzi* Gebien, 1938, by original designation. Status: valid genus in Pimeliinae: Adelostomini. Note: the earlier usage of *Lepidochora* by Gebien (1938b: 71) is unavailable since it was published after 1930 without fixation of a type species in the original publication (ICZN 1999, Article 13.3).

*Lepidocnemeplatia* Bousquet & Bouchard in Bousquet et al., 2018: 20 [F]. Type species: *Cnemeplatiasericea* Horn, 1870, by original designation. Status: valid genus in Pimeliinae: Cnemeplatiini: Cnemeplatiina. Note: *Lepidocnemeplatia* was previously described by Kaszab (1938: 80) without original type species fixation and is therefore unavailable from that date (ICZN 1999, Article 13.3); Löbl and Merkl (2003: 245) designated *Cnemeplatiasericea* Horn, 1870 as the type species of Koch’s name but did not explicitly indicate the genus-group name as intentionally new (ICZN 1999, Article 16.1).

*Lepidospilus* Agassiz, 1846b: 205 [M]. Type species [automatic]: *Pachycoeliasulcicollis* Boisduval, 1835, by monotypy. Status: junior synonym of *Pachycoelia* Boisduval, 1835 in Tenebrioninae: Heleini: Cyphaleina. Note: unjustified emendation of *Lepispilus* Westwood, 1841, not in prevailing usage.

*Lepispilus* Westwood, 1841a: 44 [M]. Type species: *Pachycoeliasulcicollis* Boisduval, 1835, by monotypy. Status: junior synonym of *Pachycoelia* Boisduval, 1835 in Tenebrioninae: Heleini: Cyphaleina. Synonymy: Lacordaire (1859b: 413).

*Leprocaulinus* Kaszab, 1982c: 75 [M]. Type species: *Leprocaulinuskrikkeni* Kaszab, 1982, by original designation. Status: valid genus in Stenochiinae: Cnodalonini. Note: this genus-group name has been considered a junior homonym of *Leprocaulinus* Uvarov, 1940 [Orthoptera] in literature on Tenebrionidae; however, no type species has yet been designated for orthopteran name *Leprocaulus* Redtenbacher, 1908 nor its replacement name *Leprocaulinus* Uvarov, 1940 (see Herwaarden 1998: 65) and therefore *Leprocaulinus* Uvarov, 1940 is unavailable since it has been proposed as a replacement name after 1930, without a type species designation, for a generic taxon without valid typification (ICZN 1999: Article 13.3.1).

*Leprocaulus* Fairmaire, 1896c: 95 [M]. Type species: *Leprocaulusclavipes* Fairmaire, 1896, by monotypy. Status: valid subgenus of *Hexarhopalus* Fairmaire, 1891 in Stenochiinae: Cnodalonini.

*Leptasida* Chatanay, 1914b: 3 [F]. Type species: *Leptasidatenuipes* Chatanay, 1914, by original designation. Status: valid genus in Pimeliinae: Asidini.

*Leptasida* Reitter, 1917a: 40, 60 [F]. Type species: *Asidadiecki* Allard, 1869, by subsequent designation (F. Soldati 2008: 33). Status: junior synonym of *Asida* Latreille, 1802 in Pimeliinae: Asidini. Synonymy: Viñolas and Cartagena (2005: 291). Note: junior homonym of *Leptasida* Chatanay, 1914 [Coleoptera: Tenebrionidae: Pimeliinae: Asidini].

*Leptinostethus* Borchmann, 1936: 238, 466 [M]. Type species: *Leptinostethusmethneri* Borchmann, 1936, by original designation. Status: valid genus in Lagriinae: Lagriini: Statirina.

*Leptocolena* Allard, 1880: 74 [F]. Type species: *Blapsmucronata* Latreille, 1804, by subsequent designation (Harold 1882: 194). Status: junior synonym of *Blaps* Fabricius, 1775 in Blaptinae: Blaptini: Blaptina. Synonymy: Gebien (1910b: 226).

*Leptoderis* Billberg, 1820: 31 [F]. Type species: *Tenebriocollaris* Linnaeus, 1767, by monotypy. Status: valid genus in Pimeliinae: Elenophorini: Elenophorina.

*Leptoderops* Löbl, Bouchard, Merkl & Bousquet, 2020: 5 [M]. Type species: *Thraustocoluspriesneri* Koch, 1934, by original designation. Status: valid subgenus of *Thraustocolus* Kraatz, 1866 in Pimeliinae: Tentyriini. Note: name first proposed by Koch (1934: 96) without fixation of a type species in the original publication (ICZN 1999, Article 13.3); Löbl et al. (2008a: 40) designated *Thraustocoluspriesneri* Koch, 1934 as the type species of Koch’s name but did not explicitly indicate the genus-group name as intentionally new (ICZN 1999, Article 16.1).

*Leptodes* Dejean, 1834: 181 [M]. Type species: *Sepidium boisduvalii* Zubkov, 1833, by monotypy. Status: valid genus and subgenus in Pimeliinae: Leptodini.

*Leptodinopsis* Kaszab, 1959b: 353, 359 [F]. Type species: *Sepidium boisduvalii* Zubkov, 1833, by original designation. Status: junior synonym of *Leptodes* Dejean, 1834 in Pimeliinae: Leptodini. Synonymy: G.S. Medvedev and Iljina (2007: 882).

*Leptodopsis* Haag-Rutenberg, 1879a: 408 [F]. Type species: *Leptodopsisinsignis* Haag-Rutenberg, 1879, by monotypy. Status: valid subgenus of *Leptodes* Dejean, 1834 in Pimeliinae: Leptodini.

*Leptogastrus* W.J. MacLeay, 1872: 293 [M]. Type species: *Leptogastrusmastersii* W.J. MacLeay, 1872, by monotypy. Status: valid genus in Lagriinae: Adeliini.

*Leptomorpha* Faldermann, 1835: 406 [F]. Type species: *Leptomorphachinensis* Faldermann, 1835, by monotypy. Status: junior synonym of *Blaps* Fabricius, 1775 in Blaptinae: Blaptini: Blaptina. Synonymy: Champion (1895: 48).

*Leptonychoides* Schawaller, 1990: 51 [M]. Type species: *Leptonychoidesjuengeri* Schawaller, 1990, by original designation. Status: valid genus in Pimeliinae: Erodiini.

*Leptonychus* Chevrolat, 1833a: 26, pl. 1 [M]. Type species: *Leptonychuserodioides* Chevrolat, 1833, by monotypy. Status: valid genus in Pimeliinae: Erodiini.

*Leptoscapha* Fairmaire, 1886c: 73 [F]. Type species [automatic]: *Stenoscaphaspissicornis* Fairmaire, 1885, by monotypy. Status: senior synonym of *Brachypophlaeus* Fairmaire, 1897 in Tenebrioninae: Ulomini. Synonymy: Alluaud (1899: 342, as “*Brachypophloeus*”). Note: replacement name for *Stenoscapha* Fairmaire, 1885; junior homonym of *Leptoscapha* Fischer, 1883 [Mollusca].

*Leptosora* Borchmann, 1936: 238, 471 [F]. Type species: *Leptosorahamata* Borchmann, 1936, by original designation. Status: valid genus in Lagriinae: Lagriini: Statirina.

*Leptosphena* Semenov, 1891: 356, 358 [F]. Type species: *Sphenariatomentosa* Semenov, 1889 (= *Sphenariarubripes* Reitter, 1889), by monotypy. Status: valid genus in Pimeliinae: Tentyriini.

*Lepturidea* Fauvel, 1862: 150 [F]. Type species: *Lepturideadeplanchei* Fauvel, 1862, by monotypy. Status: valid genus in Alleculinae: Alleculini: Alleculina.

*Leptynoderes* Solier, 1838b: 8, 44 [M]. Type species: *Scotobiusvaricosus* Germar, 1823, by original designation. Status: valid genus in Tenebrioninae: Scotobiini.

*Lesbidana* Reitter, 1904: 173 [F]. Type species: *Melanesthessimplex* Reitter, 1897, by subsequent designation (G.S. Medvedev 1990: 203). Status: valid subgenus of *Melanesthes* Dejean, 1834 in Blaptinae: Opatrini: Opatrina.

*Leucolaephus* P.H. Lucas, 1859: xxii [M]. Type species: *Leucolaephusperrisii* P.H. Lucas, 1859 (= *Pimelialiliputana* P.H. Lucas, 1857), by subsequent designation (Gebien 1937a: 803). Status: valid genus in Pimeliinae: Pimeliini. Note: *Leucolaephus* is an incorrect subsequent spelling of the original spelling *Leucoloephus*, first used by Kraatz (1865: 314), and is in prevailing usage; *Leucolaephus* is deemed to be the correct original spelling (ICZN 1999, Article 33.3.1), see Bouchard et al. (2005: 506).

*Lichenum* Agassiz, 1846b: 203, 209 [N]. Type species [automatic]: *Opatrumpictum* Fabricius, 1801, by monotypy. Status: junior synonym of *Leichenum* Dejean, 1834 in Blaptinae: Pedinini: Leichenina. Note: unjustified emendation of *Leichenum* Dejean, 1834, not in prevailing usage.

*Licinoma* Pascoe, 1869: 140 [F]. Type species: *Licinomanitida* Pascoe, 1869, by monotypy. Status: valid genus in Lagriinae: Adeliini.

*Licymnius* Bates, 1868: 271 [M]. Type species: *Licymniusfoveicollis* Bates, 1868, by monotypy. Status: junior synonym of *Lepturidea* Fauvel, 1862 in Alleculinae: Alleculini: Alleculina. Synonymy: Carter (1915a: 60, with *Chromomaea* Pascoe, 1866, a junior synonym of *Lepturidea* Fauvel, 1862).

*Lindia* Blackburn, 1888: 275 [F]. Type species: *Lindiaangusta* Blackburn, 1888, by monotypy. Status: junior synonym of *Lyphia* Mulsant & Rey, 1859 in Tenebrioninae: Triboliini. Synonymy: Champion (1894a: 351, 370). Note: junior homonym of *Lindia* Dujardin, 1841 [Rotifera].

*Lineocrypticus* Koch, 1950c: 52 [M]. Type species: *Lineocrypticushessei* Koch, 1950, by original designation. Status: valid genus in Diaperinae: Crypticini.

*Linio* Bouchard & Bousquet, **new subgenus** [M]. Type species: *Niliolanatus* Germar, 1823, by **present designation**. Status: valid subgenus of *Nilio* Latreille, 1802 in Nilioninae. Note: Mader (1936: 94) introduced the new subgenus name *Linio* for several nominal species, but unfortunately did not designate a type species; the subgenusLinio, which has been treated as valid since 1936, is therefore unavailable (ICZN 1999, Article 13.3); we hereby make the name available by selecting *Niliolanatus* Germar, 1823 as type species and referring to Mader (1936: 94) for the character states that characterise and differentiate *Linio*.

*Liodema* Horn, 1870: 378, 385 [F]. Type species: *Platydemalaevis* Haldeman, 1848, by monotypy. Status: valid genus in Diaperinae: Diaperini: Diaperina.

*Liodocistela* Pic, 1930a: 28 [F]. Type species: *Cistelopsisrufomarginata* Pic, 1930, by monotypy. Status: valid genus in Alleculinae: Alleculini: Alleculina.

*Lioprosodes* Reitter, 1909a: 121 [M]. Type species: *Prosodesdilaticollis* Motschulsky, 1859, by original designation. Status: junior synonym of *Prosodes* Eschscholtz, 1829 in Blaptinae: Blaptini: Prosodina. Synonymy: Skopin (1960a: 46).

*Lisa* Haag-Rutenberg, 1879c: 134 [F]. Type species: *Lisasingularis* Haag-Rutenberg, 1879 (= *Alleculaomophloides* Hope, 1843), by monotypy. Status: junior synonym of *Metistete* Pascoe, 1866 in Alleculinae: Alleculini: Alleculina. Synonymy: Carter (1915a: 78).

*Litasida* Casey, 1912: 77, 184 [F]. Type species: *Litasidatownsendi* Casey, 1912, by original designation. Status: valid genus in Pimeliinae: Asidini.

*Litheleodes* Blaisdell, 1909: 114 [M]. Type species: *Blaps extricata* Say, 1824, by subsequent designation (Triplehorn and Thomas 2015: 11). Status: valid subgenus of *Eleodes* Eschscholtz, 1829 in Blaptinae: Amphidorini.

*Lithoblaps* Motschulsky, 1860c: 532 [F]. Type species: *Tenebriogigas* Linnaeus, 1767, by subsequent designation (Skopin, 1960a: 44). Status: junior synonym of *Blaps* Fabricius, 1775 in Blaptinae: Blaptini: Blaptina. Synonymy: Gemminger in Gemminger and Harold (1870: 1860). Note: the original combination of the name of the type species, *Tenebriogigas* Linnaeus, 1767, is a junior primary homonym of *Tenebriogigas* Linnaeus, 1763.

*Litoboriolus* Español, 1945: 310, 313 [M]. Type species: *Olocratescollaris* Mulsant & Rey, 1854, by original designation. Status: valid genus in Blaptinae: Dendarini: Dendarina.

*Litoboromimus* Koch, 1948: 413 [M]. Type species: *Litoborusparallelus* Schuster, 1919, by original designation. Status: valid subgenus of *Allophylax* Bedel, 1906 in Blaptinae: Dendarini: Melambiina.

*Litoborus* Mulsant & Rey, 1854: 124, 126 [M]. Type species: *Phylaxmoreletii* P.H. Lucas, 1846, by subsequent designation (Antoine 1931: 181). Status: valid genus and subgenus in Blaptinae: Dendarini: Melambiina.

*Litopous* Matthews, 2012: 1 [M]. Type species: *Litopousbaehri* Matthews, 2012, by original designation. Status: valid genus in Alleculinae: Alleculini: Alleculina. Note: placed in the subtribe Alleculina by Matthews and Lawrence (2019: 645).

*Litororus* Reitter, 1904: 96 [M]. Type species: *Micrositussemicostatus* Mulsant & Rey, 1854, by monotypy. Status: valid genus in Blaptinae: Dendarini: Dendarina.

*Lixionica* Blackburn, 1896: 280 [F]. Type species: *Lixionicacostatipennis* Blackburn, 1896, by monotypy. Status: valid genus in Pimeliinae: Vacronini.

*Lobatopezus* Pic, 1952c: 2 [M]. Type species: *Lobatopezusdiversecostatus* Pic, 1952, by monotypy. Status: valid genus in Tenebrioninae: Amarygmini. Note: Bremer (2005: 211) transferred this genus to Chrysomeloidea: Chrysomelidae, but later retained this genus in Tenebrioninae: Amarygmini after further study (Bremer 2013b: 127–128).

*Lobetas* Motschulsky, 1872: 26 [M]. Type species: *Tenebriocostatus* Guérin-Méneville, 1831, by original designation. Status: junior synonym of *Hipalmus* Bates, 1870 in Tenebrioninae: Tenebrionini. Synonymy: C.O. Waterhouse (1876: 288). Note: the original combination of the name of the type species, *Tenebriocostatus* Guérin-Méneville, 1831, is a junior primary homonym of *Tenebriocostatus* Pallas, 1781.

*Lobodera* Mulsant & Rey, 1859c: 12, 18 [F]. Type species: *Loboderarufescens* Mulsant & Rey, 1859, by monotypy. Status: junior synonym of *Penthicus* Faldermann, 1836 in Blaptinae: Opatrini: Opatrina. Synonymy: Gebien (1910b: 333).

*Lobometopon* Casey, 1907: 379, 385 [N]. Type species: *Epitragusfusiformis* Casey, 1890, by original designation. Status: valid genus in Pimeliinae: Epitragini.

*Lobophilomorphus* Pic, 1911a: 183 [M]. Type species: *Lobophilomorphusrubicollis* Pic, 1911, by monotypy. Status: valid genus in Lagriinae: Lagriini: Statirina.

*Lobopoda* Solier, 1835a: 233 [F]. Type species: *Lobopodastriata* Solier, 1835, by subsequent designation (Bousquet et al. 2015: 134). Status: valid genus and subgenus in Alleculinae: Alleculini: Alleculina.

*Lobothorax* Gemminger, 1870: 124 [M]. Type species [automatic]: *Loboderarufescens* Mulsant & Rey, 1859, by monotypy. Status: junior synonym of *Penthicus* Faldermann, 1836 in Blaptinae: Opatrini: Opatrina. Note: unnecessary replacement name for *Lobodera* [as “*Loboderus*”] Mulsant & Rey, 1859.

*Locrodes* Casey, 1907: 332 [M]. Type species: *Emmenastuspiceus* Casey, 1890, by subsequent designation (Bousquet et al. 2018: 104). Status: valid subgenus of *Hylocrinus* Casey, 1907 in Pimeliinae: Edrotini.

*Lodinus* Mulsant & Rey, 1859a: 113, 131 [M]. Type species: *Lodinusnigroaeneus* Mulsant & Rey, 1859 (= *Blapstinuspunctulatus*Solier 1851), by monotypy. Status: junior synonym of *Blapstinus* Dejean, 1821 in Blaptinae: Opatrini: Blapstinina. Synonymy: Gemminger in Gemminger and Harold (1870: 1923).

*Loensus* R. Lucas, 1920: 380, 489 [M]. Type species [automatic]: *Pedinopsispilipes* Gebien, 1910, by monotypy. Status: valid genus and subgenus in Blaptinae: Pedinini: Pedinina. Note: replacement name for *Pedinopsis* Gebien, 1910.

*Lomocnemis* Gebien, 1921a: 287 [F]. Type species: *Lomocnemispolita* Gebien, 1921, by monotypy. Status: valid genus in Stenochiinae: Cnodalonini.

*Longuloodescelis* Kaszab, 1940b: 940, 957 [F]. Type species: *Platyscelishirta* Seidlitz, 1893, by original designation. Status: valid subgenus of *Oodescelis* Motschulsky, 1845 in Blaptinae: Platyscelidini. Note: the First Reviser (*Longuloodescelis* Kaszab, 1940 versus *Trichoodescelis* Kaszab, 1940) is Egorov (2004: 595).

*Lophocnemis* Mäklin, 1867: 505 [F]. Type species: *Lophocnemisamabilis* Mäklin, 1867, by monotypy. Status: valid genus in Stenochiinae: Stenochiini.

*Lopholagria* Borchmann, 1916a: 48, 97 [F]. Type species: *Lagriaamoena* Fåhraeus, 1870, by monotypy. Status: valid genus and subgenus in Lagriinae: Lagriini: Lagriina.

*Lophoma* Solier, 1835b: 253, 285 [N]. Type species: *Pimeliapunctata* Fabricius, 1798, by monotypy. Status: junior synonym of *Pachychila* Eschscholtz, 1831 in Pimeliinae: Tentyriini. Synonymy: Lacordaire (1859a: 46). Note: the original combination of the name of type species, *Pimeliapunctata* Fabricius, 1798, is a junior primary homonym of *Pimeliapunctata* Thunberg, 1787.

*Lophophyllus* Fairmaire, 1887c: 71 [M]. Type species: *Lophophylluscostipennis* Fairmaire, 1887, by monotypy. Status: valid genus in Lagriinae: Lagriini: Statirina.

*Lordodera* Gebien, 1921b: 64 [F]. Type species: *Tenebrioquadrihamatus* Fairmaire, 1875, by monotypy. Status: valid genus in Stenochiinae: Cnodalonini.

*Lorelopsis* Champion, 1896: 15 [F]. Type species: *Lorelopsispilosa* Champion, 1896, by monotypy. Status: junior synonym of *Lorelus* Sharp, 1876 in Lagriinae: Lupropini. Synonymy: Doyen (1993: 295).

*Lorelus* Sharp, 1876: 76 [M]. Type species: *Loreluspriscus* Sharp, 1876, by monotypy. Status: valid genus in Lagriinae: Lupropini.

*Loricula* Novák, 2016d: 45 [F]. Type species: *Alleculasubaeneipennis* Pic, 1922, by original designation. Status: senior synonym of *Loriculoides* Novák, 2020 in Alleculinae: Alleculini: Alleculina. Note: junior homonym of *Loricula* Curtis, 1833 [Hemiptera].

*Loriculoides* Novák, 2020i: 9 [M]. Type species [automatic]: *Alleculasubaeneipennis* Pic, 1922, by original designation. Status: valid genus in Alleculinae: Alleculini: Alleculina. Note: replacement name for *Loricula* Novák, 2016.

*Lornamus* Koch, 1952a: 191 [M]. Type species: *Lornamusdividiopsis* Koch, 1952, by original designation. Status: junior synonym of *Cryptocarpes* Koch, 1952 in Pimeliinae: Caenocrypticini. Synonymy: Endrödy-Younga (1996: 21).

*Lorona* Borchmann, 1936: 16, 62 [F]. Type species: *Lagriabakeri* Borchmann, 1930, by original designation. Status: valid genus in Lagriinae: Lagriini: Lagriina.

*Loubacantus* Bonadona, 1959: 1033 [M]. Type species: *Loubacantusgiganteus* Bonadona, 1959, by original designation. Status: junior synonym of *Entypodera* Gerstaecker, 1871 in Lagriinae: Lagriini: Statirina. Synonymy: Bonadona (1984: 469).

*Louwerensia* Kaszab, 1964b: 104 [F]. Type species: *Louwerensiapapuana* Kaszab, 1964, by original designation. Status: valid genus in Diaperinae: Diaperini: Diaperina.

*Loxostethus* Triplehorn, 1962: 504 [M]. Type species: *Loxostethusfasciatus* Triplehorn, 1962, by original designation. Status: valid genus in Diaperinae: Diaperini: Diaperina. Note: **new placement** [RLA], previously included in Diaperinae: Diaperini: Adelinina.

*Lucidolaena* Endrödy-Younga & Schawaller, 2002: 9, 20 [F]. Type species: *Lucidolaenaamatolensis* Endrödy-Younga & Schawaller, 2002, by original designation. Status: valid genus in Lagriinae: Laenini.

*Luebbertia* Koch, 1963: 20, 44 [F]. Type species: *Luebbertiaplana* Koch, 1963, by monotypy. Status: valid genus in Blaptinae: Opatrini: Stizopodina.

*Luprops* Hope, 1833: 63 [M]. Type species: *Lupropschrysophthalmus* Hope, 1833 (= *Tageniaindica* Wiedemann, 1823), by monotypy. Status: valid genus in Lagriinae: Lupropini.

*Luzonoplonyx* Bremer, 2009: 331 [M]. Type species: *Luzonoplonyxgrandis* Bremer, 2009, by original designation. Status: valid genus in Tenebrioninae: Amarygmini.

*Lycanthropa* J. Thomson, 1860b: 20 [F]. Type species: *Eurychoracimicoides* Quensel, 1806, by monotypy. Status: valid genus in Pimeliinae: Adelostomini.

*Lycidioides* Ando, 2003a: 107 [M]. Type species: *Lycidioideskaniei* Ando, 2003, by original designation. Status: valid genus in Stenochiinae: Cnodalonini.

*Lycogonocnemis* Pic, 1915a: 13 [F]. Type species: *Lycogonocnemisrufa* Pic, 1915, by monotypy. Status: valid subgenus of *Paragonocnemis* Kraatz, 1899 in Tenebrioninae: Amarygmini.

*Lycoscelis* Blair, 1929a: 242 [F]. Type species: *Lycoscelisfulva* Blair, 1929 (= *Plinthochrousgounellei* Fairmaire, 1891), by original designation. Status: junior synonym of *Plinthochrous* Fairmaire, 1891 in Tenebrioninae: Amarygmini. Synonymy: Ardoin (1962b: 957).

*Lycula* Campbell, 1976: 30 [F]. Type species: *Lyculachilensis* Campbell, 1976, by original designation. Status: valid genus in Alleculinae: Alleculini: Alleculina.

*Lygestira* Pascoe, 1866a: 470 [F]. Type species: *Prophanessimplex* Westwood, 1849, by subsequent designation (R. Lucas 1920: 384). Status: junior synonym of *Prophanes* Westwood, 1849 in Tenebrioninae: Heleini: Cyphaleina. Synonymy: Matthews (1992: 488).

*Lygophilus* Rafinesque, 1815: 114 [M]. Type species [automatic]: *Epitragusfuscus* Latreille, 1804, by subsequent monotypy (Latreille 1804: 322). Status: junior synonym of *Epitragus* Latreille, 1802 in Pimeliinae: Epitragini. Note: unnecessary replacement name for *Epitragus* Latreille, 1802.

*Lyphia* Mulsant & Rey, 1859b: 166 [F]. Type species: *Lyphiaficicola* Mulsant & Rey, 1859 (= *Biustetraphyllus* Fairmaire, 1857), by monotypy. Status: valid genus in Tenebrioninae: Triboliini.

*Lyprochelyda* Fairmaire, 1899d: 214 [F]. Type species: *Lyprochelydapurpurina* Fairmaire, 1899, by monotypy. Status: valid genus in Lagriinae: Goniaderini.

*Lyprosodes* Reitter, 1909a: 116 [M]. Type species: *Prosodesquadricostata* Reitter, 1893, by original designation. Status: valid subgenus of *Prosodes* Eschscholtz, 1829 in Blaptinae: Blaptini: Prosodina.

*Lystronychus* Latreille, 1829a: 41 [M]. Type species: *Helopsequestris* Fabricius, 1775, by subsequent designation (Saunders 1837: 154). Status: valid genus and subgenus in Alleculinae: Alleculini: Xystropodina. Note: *Lystronychus* is an incorrect subsequent spelling of the original spelling *Lystronichus*, first used by Perty (1832: 63), in prevailing usage; *Lystronychus* is deemed to be the correct original spelling (ICZN 1999, Article 33.3.1).

*Macellocerus* Solier, 1848: 154, 263 [M]. Type species [automatic]: *Dolichoderusacuminatus* Klug, 1833, by monotypy. Status: valid genus in Tenebrioninae: Toxicini: Nycteropina. Note: replacement name for *Dolichoderus* Klug, 1833; discovery of the older unused name *Dillacerus* Solier, 1835 threatens the stability of *Macellocerus* Solier, 1848; we recommend that an application be submitted to the International Commission on Zoological Nomenclature to conserve usage of *Macellocerus* Solier, 1848.

*Machla* Herbst, 1799: 152 [F]. Type species: *Opatrumvillosum* G.-A. Olivier, 1795, by subsequent designation (R. Lucas 1920: 386). Status: valid genus in Pimeliinae: Asidini. Note: the older name *Machla* Lichtenstein, 1796 was published in a work that was suppressed for nomenclatural purposes (ICZN 1995, Opinion 1820).

*Machlasida* Escalera, 1907: 336 [F]. Type species: *Asidamuleyhafidi* Escalera, 1907, by subsequent designation (Escalera 1928: 137). Status: valid subgenus of *Alphasida* Escalera, 1905 in Pimeliinae: Asidini.

*Machleida* Fåhraeus, 1870: 256 [F]. Type species: *Machleidanodulosa* Fåhraeus, 1870, by monotypy. Status: valid genus in Pimeliinae: Asidini.

*Machloida* Rye, 1873: 286 [F]. Type species [automatic]: *Machleidanodulosa* Fåhraeus, 1870, by monotypy. Status: junior synonym of *Machleida* Fåhraeus, 1870 in Pimeliinae: Asidini. Note: unjustified emendation of *Machleida* Fåhraeus, 1870, not in prevailing usage.

*Machlomorpha* Péringuey, 1899: 257 [F]. Type species: *Machlomorphaaltitudinis* Péringuey, 1899, by subsequent designation (Gebien 1937a: 739). Status: valid genus and subgenus in Pimeliinae: Asidini.

*Machlophila* Wilke, 1924: 521 [F]. Type species: *Machlophilavogti* Wilke, 1924, by monotypy. Status: junior synonym of *Machla* Herbst, 1799 in Pimeliinae: Asidini. Synonymy: Koch (1962a: 121, 144).

*Machloplasta* Wilke, 1922: 263 [F]. Type species: *Opatrumvillosum* G.-A. Olivier, 1795, by original designation. Status: junior synonym of *Machla* Herbst, 1799 in Pimeliinae: Asidini. Synonymy: Koch (1962a: 119).

*Machlopsis* Pomel, 1871: 236 [F]. Type species: *Eurychoralevaillantii* P.H. Lucas, 1850, by original designation. Status: valid genus and subgenus in Pimeliinae: Adelostomini. Note: *Machlopsis* is an incorrect subsequent spelling of the original spelling *Maclopsis*, first used by Bedel (1887: 199), in prevailing usage; *Machlopsis* is deemed to be the correct original spelling (ICZN 1999, Article 33.3.1).

*Macradesmia* Kaszab, 1959a: 402 [F]. Type species: *Adesmiaservillei* Solier, 1835, by monotypy. Status: valid subgenus of *Adesmia* Fischer, 1822 in Pimeliinae: Adesmiini. Note: name first proposed by Koch (1944b: 146) without fixation of a type species in the original publication (ICZN 1999, Article 13.3).

*Macradesmia* Löbl & Merkl in Löbl et al., 2020: 1 [F]. Type species: *Pimeliacancellata* Klug, 1830, by original designation. Status: junior synonym of *Macradesmia* Kaszab, 1959 in Pimeliinae: Adesmiini. Note: name first proposed by Koch (1944b: 146) without fixation of a type species in the original publication (ICZN 1999, Article 13.3); not knowing about the availability of *Macradesmia* Kaszab, 1959, Löbl & Merkl (2003: 244) designated *Pimeliacancellata* Klug, 1830 as the type species of Koch’s name but did not explicitly indicate the genus-group name as intentionally new (ICZN 1999, Article 16.1) and subsequently *Macradesmia* was proposed as new by Löbl and Merkl in Löbl et al. (2020: 1); junior homonym of *Macradesmia* Kaszab, 1959 [Coleoptera: Tenebrionidae: Pimeliinae: Adesmiini].

*Macrarthra* Agassiz, 1846b: 35, 219 [F]. Type species [automatic]: *Arthromacradonacioides* W. Kirby, 1837 (= *Lagriaaenea* Say, 1824), by monotypy. Status: junior synonym of *Arthromacra* W. Kirby, 1837 in Lagriinae: Lagriini: Statirina. Note: unjustified emendation of *Arthromacra* W. Kirby, 1837, not in prevailing usage; the original combination of the accepted name of the type species, *Lagriaaenea* Say, 1824, is a junior primary homonym of *Lagriaaenea* Fabricius, 1775.

*Macroartactes* Pic, 1924a: 24 [M]. Type species: *Macroartactescostulatus* Pic, 1924, by subsequent designation (Gebien 1935: 64). Status: junior synonym of *Artactes* Pascoe, 1868 in Stenochiinae: Cnodalonini. Synonymy: Gebien (1935: 64).

*Macrocasnonidea* Pic, 1934a: 31 [F]. Type species: *Nemostiraquadrimaculata* Pic, 1912, by monotypy. Status: valid genus in Lagriinae: Lagriini: Statirina.

*Macrocistela* Pic, 1941: 13 [F]. Type species: *Macrocistelastriata* Pic, 1941, by monotypy. Status: valid genus in Alleculinae: incertae sedis.

*Macrocistelopsis* Pic, 1956: 89 [F]. Type species: *Macrocistelopsistestaceicolor* Pic, 1956, by monotypy. Status: valid genus in Alleculinae: Alleculini: Alleculina.

*Macrohyperchalca* Pic, 1935b: 98 [F]. Type species: *Hyperchalcastriata* Pic, 1935, by monotypy. Status: valid subgenus of *Hyperchalca* Fairmaire, 1869 in Stenochiinae: Stenochiini.

*Macrolagria* Lewis, 1895: 422 [F]. Type species: *Statirarufobrunnea* Marseul, 1876, by original designation. Status: valid genus in Lagriinae: Lagriini: Statirina.

*Macropachylesthus* Pic, 1923c: 28 [M]. Type species: *Macropachylesthusgigas* Pic, 1923, by subsequent designation (Gebien 1941: 350). Status: valid genus in Stenochiinae: Cnodalonini.

*Macroperas* Carter, 1914c: 393 [N]. Type species: *Macroperasantennale* Carter, 1914, by monotypy. Status: junior synonym of *Daedrosis* Bates, 1868 in Lagriinae: Adeliini. Synonymy: Matthews (1998: 756).

*Macrophanes* Iablokoff-Khnzorian, 1957: 165 [M]. Type species: *Hedyphanescorax* Iablokoff-Khnzorian, 1957 (= *Helopsamandanus* Reitter, 1902), by original designation. Status: junior synonym of *Delonurops* Reitter, 1922 in Tenebrioninae: Helopini: Helopina. Synonymy: Nabozhenko (2002b: 689).

*Macrophtalmus* Montrouzier, 1855: 33 [M]. Type species: *Macrophtalmuscoeruleus* Montrouzier, 1855, by monotypy. Status: senior synonym of *Macrophthalmata* Strand, 1935 in Tenebrionidae: incertae sedis. Note: this genus has been treated as a junior homonym of *Macrophtalmus* Laporte, 1832 [Hemiptera] in the literature; however, the hemipteran name was originally proposed with two spellings, *Macrophthalmus* (Laporte, 1832: 6, 11, 87) and *Macrophtalmus* (Laporte, 1832: 78); the alternative original spelling *Macrophtalmus* of the hemipteran name, used by Laporte (1832: 78), was rejected by Neave (1940: 17) who acted as First Reviser; therefore *Macrophtalmus* Montrouzier, 1855 is the valid name for the tenebrionid genus since it is not a junior homonym.

*Macrophthalmata* Strand, 1935b: 302 [F]. Type species [automatic]: *Macrophtalmuscoeruleus* Montrouzier, 1855, by monotypy. Status: junior synonym of *Macrophtalmus* Montrouzier, 1855 in Tenebrionidae: incertae sedis. Note: replacement name for *Macrophtalmus* Montrouzier, 1855.

*Macropoda* Solier, 1835b: 512, 515 [F]. Type species: *Pimeliavariolaris* G.-A. Olivier, 1795, by subsequent designation (Hope 1841: 118). Status: valid subgenus of *Adesmia* Fischer, 1822 in Pimeliinae: Adesmiini.

*Macropodesmia* Löbl & Merkl in Löbl et al., 2020: 1 [F]. Type species: *Pimeliareticulata* Klug, 1830, by original designation. Status: valid subgenus of *Adesmia* Fischer, 1822 in Pimeliinae: Adesmiini. Note: name first proposed by Koch (1944b: 147) without fixation of a type species in the original publication (ICZN 1999, Article 13.3); Löbl and Merkl (2003: 244) designated *Pimeliareticulata* Klug, 1830 as the type species of Koch’s name but did not explicitly indicate the genus-group name as intentionally new (ICZN 1999, Article 16.1).

*Macrostethus* Wollaston, 1854: 504 [M]. Type species: *Macrostethustuberculatus* Wollaston, 1854, by monotypy. Status: valid genus in Stenochiinae: Cnodalonini.

*Macrosynopticus* Pic, 1922d: 25 [M]. Type species: *Macrosynopticuscostatus* Pic, 1922, by monotypy. Status: valid genus in Tenebrioninae: Amarygmini.

*Macrotrachyscelis* Pic, 1925b: 12 [F]. Type species: *Macrotrachyscelisrufa* Pic, 1925, by monotypy. Status: valid genus in Diaperinae: Trachyscelini.

*Macrozophobas* Pic, 1913b: 6 [M]. Type species: *Macrozophobasgracilicornis* Pic, 1913, by monotypy. Status: valid subgenus of *Zophobas* Dejean, 1834 in Tenebrioninae: Tenebrionini.

*Macruloma* Pic, 1921d: 20 [N]. Type species: *Macrulomagigas* Pic, 1921, by monotypy. Status: valid genus in Tenebrioninae: Ulomini.

*Madagassa* Koch, 1950a: 67 [F]. Type species [automatic]: *Pycnaaphodina* Fairmaire, 1894, by monotypy. Status: valid genus in Phrenapatinae: Penetini. Note: replacement name for *Pycna* Fairmaire, 1894.

*Madobalus* Fairmaire, 1901a: 73 [M]. Type species: *Madobalusrotundicollis* Fairmaire, 1901, by monotypy. Status: valid genus in Blaptinae: Platynotini: Platynotina.

*Madreallecula* Kanda, 2013: 587 [F]. Type species: *Madrealleculamcclevei* Kanda, 2013, by original designation. Status: valid genus in Alleculinae: Alleculini: Alleculina.

*Maerodes* C.O. Waterhouse, 1877: 72 [M]. Type species: *Prophaneswestwoodi* W.J. MacLeay, 1872 (= *Prophanesaculeatus* Westwood, 1849), by monotypy. Status: junior synonym of *Prophanes* Westwood, 1849 in Tenebrioninae: Heleini: Cyphaleina. Synonymy: Carter (1918: 717).

*Magdanalia* Novák, 2020c: 496 [F]. Type species: *Prionychusdenticulatus* Muche, 1982, by original designation. Status: valid genus in Alleculinae: Alleculini: Alleculina.

*Magela* G.S. Medvedev & Lawrence, 1986: 578 [F]. Type species: *Magelauptoni* G.S. Medvedev & Lawrence, 1986, by original designation. Status: valid genus in Diaperinae: Hyociini: Brittonina.

*Magrebmelia* Mas-Peinado, Buckley, Ruiz & García-París, 2018: 540 [F]. Type species: *Pimeliaxauenensis* Escalera, 1923, by original designation. Status: valid subgenus of *Pimelia* Fabricius, 1775 in Pimeliinae: Pimeliini.

*Mahena* Gebien, 1922b: 315 [F]. Type species: *Mahenacuprea* Gebien, 1922, by monotypy. Status: valid genus in Stenochiinae: Cnodalonini.

*Makicula* Novák, 2012: 275 [F]. Type species: *Makiculaphoupaneica* Novák, 2012, by original designation. Status: valid genus in Alleculinae: Alleculini: Alleculina.

*Malacova* Fairmaire, 1898c: 479 [F]. Type species: *Malacovabicolor* Fairmaire, 1898, by **present designation**. Status: junior synonym of *Damatris* Laporte, 1840 in Stenochiinae: Cnodalonini. Synonymy: Gebien (1942a: 316).

*Malaiseum* Borchmann, 1941a: 13 [N]. Type species: *Malaiseumsingulare* Borchmann, 1941, by monotypy. Status: valid genus in Lagriinae: Lagriini: Statirina.

*Malayaplamius* Masumoto, 1986a: 17 [M]. Type species: *Malayaplamiussakaii* Masumoto, 1986, by original designation. Status: valid genus in Stenochiinae: Cnodalonini.

*Malaymira* Novák, 2020g: 56 [F]. Type species: *Malaymirajenisi* Novák, 2020, by original designation. Status: valid genus in Alleculinae: Alleculini: Gonoderina.

*Malayoscelis* Schawaller, 2003: 198 [F]. Type species: *Malayoscelisgebieni* Schawaller, 2003, by original designation. Status: valid genus in Lagriinae: Pycnocerini.

*Malaysphena* Bečvář & Purchart, 2008: 38 [F]. Type species: *Laosocryptobatesrotundipennis* Kaszab 1960, by original designation. Status: valid genus in Stenochiinae: Cnodalonini.

*Mallogria* Borchmann, 1936: 20, 165 [F]. Type species: *Lagrialongipilis* Fairmaire, 1875, by original designation. Status: valid genus in Lagriinae: Lagriini: Lagriina.

*Mamorina* Antoine, 1951: 98 [F]. Type species: *Mamorinasulcaticeps* Antoine, 1951, by monotypy. Status: valid genus in Tenebrioninae: Helopini: Helopina.

*Mantichorula* Reitter, 1889a: 695 [F]. Type species: *Mantichorulasemenowi* Reitter, 1889, by monotypy. Status: valid genus in Pimeliinae: Pimeliini.

*Maracia* Gebien, 1919: 27, 34 [F]. Type species: *Camariafemoralis* Kirsch, 1866, by subsequent designation (Gebien 1942a: 319). Status: valid genus in Stenochiinae: Cnodalonini.

*Margus* Dejean, 1834: 200 [M]. Type species: **fixed herein** (ICZN 1999, Article 70.3) as *Colydiumcastaneum* Herbst, 1797, misidentified as *Tenebrioferrugineus* Fabricius, 1781 in the original designation by monotypy in Dejean (1834). Status: junior synonym of *Tribolium* W.S. MacLeay, 1825 in Tenebrioninae: Triboliini. Synonymy: Guérin-Méneville (1846: cxvii). Note: the type species “*Tenebrioferrugineus* Fabricius” was first established by monotypy (ICZN 1999, Article 68.3); as noted by C.O. Waterhouse (1896: 230) and Blair (1913: 223) the *Tenebrioferrugineus* Fabricius of authors, including Stephens (1829: 19, as “*ferruginea*, Oliv.”), was misidentified; Blair (1913: 223) noted that the species to which the authors referred is in fact *Colydiumcastaneum* Herbst, 1797; we follow currently accepted concepts (e.g., Bousquet et al. 2018: 224) and fix the type species according to the requirements of Article 70.3.2 (ICZN 1999); the nominal species *Tenebrioferrugineus* Fabricius, 1781 is a valid species in the genus *Tribolioides* Blair, 1913 [Coleoptera: Cucujidae].

*Mariepskopia* Schawaller, 2012c: 218 [F]. Type species: *Mariepskopiaalbomaculata* Schawaller, 2012, by original designation. Status: valid genus in Stenochiinae: Cnodalonini.

*Martianus* Fairmaire, 1893d: 540 [M]. Type species: *Martianuscastaneus* Fairmaire, 1893 (= *Palembusocularis* Casey, 1891), by original designation. Status: junior synonym of *Ulomoides* Blackburn, 1888 in Diaperinae: Diaperini: Diaperina. Synonymy: Halstead (1975: 241, with *Palembus* Casey, 1891, a junior synonym of *Ulomoides* Blackburn, 1888).

*Massadraamelia* Mas-Peinado, Buckley, Ruiz & García-París, 2018: 541 [F]. Type species: *Pimeliagranulithorax* Escalera, 1914, by original designation. Status: valid subgenus of *Pimelia* Fabricius, 1775 in Pimeliinae: Pimeliini.

*Mateuina* Español, 1944: 22, 30 [F]. Type species: *Mateuinakaszabi* Español, 1944, by original designation. Status: valid genus in Blaptinae: Opatrini: Ammobiina.

*Matthewsotys* Bouchard & Bousquet, **new genus** [M]. Type species: *Otysarmatus* Champion, 1895, by **present designation**. Status: valid genus in Alleculinae: Alleculini: Alleculina. Note: Matthews and Bouchard (2008: 333) selected *Otysarmatus* Champion, 1895 as the type species of *Otys* Champion, 1895 and treated it as a valid genus; however, an earlier type species designation by R. Lucas (1920: 468) confirms *Otys* Champion, 1895 as a junior synonym of *Scaletomerus* Blackburn, 1891; we hereby establish *Matthewsotys* as a new genus and refer to Matthews and Bouchard (2008: 205) for the character states that characterise and differentiate this taxon.

*Mauritianopidium* Dajoz, 1977: 244 [N]. Type species: *Mauritianopidiumoculatum* Dajoz, 1977, by original designation. Status: valid genus in Diaperinae: Gnathidiini: Anopidiina.

*Mayidicistela* Pic, 1954: 259 [F]. Type species: *Mayidicistelapaulostriata* Pic, 1954, by monotypy. Status: valid genus in Alleculinae: incertae sedis.

*Mechanetes* C.O. Waterhouse, 1887: 448 [M]. Type species: *Mechanetescornutus* C.O. Waterhouse, 1887, by monotypy. Status: valid genus in Stenochiinae: Cnodalonini.

*Mecocerus* Solier, 1835a: 241 [M]. Type species: *Xystropusdejeanii* Solier, 1835, by monotypy. Status: junior synonym of *Prostenus* Klug, 1829 in Alleculinae: Alleculini: Xystropodina. Synonymy: Lacordaire (1859b: 513). Note: junior homonym of *Mecocerus* Schönherr, 1833 [Coleoptera: Anthribidae].

*Mecopisthopus* Karsch, 1881: 46 [M]. Type species: *Mecopisthopusrohlfsi* Karsch, 1881, by original designation. Status: junior synonym of *Leucolaephus* P.H. Lucas, 1859 in Pimeliinae: Pimeliini. Synonymy: Semenov (1893: 261).

*Mecysmus* Horn, 1870: 349 [M]. Type species: *Blapstinusangustus* J.L. LeConte, 1851, by monotypy. Status: junior synonym of *Blapstinus* Dejean, 1821 in Blaptinae: Opatrini: Blapstinina. Synonymy: Lumen et al. (2020: 342).

*Mederis* Motschulsky, 1872: 24 [M]. Type species: *Upisangulata* Erichson, 1842, by original designation. Status: junior synonym of *Promethis* Pascoe, 1869 in Stenochiinae: Cnodalonini. Synonymy: C.O. Waterhouse (1876: 288).

*Medvedevia* Chigray, 2019: 915 [F]. Type species: *Medvedeviaglebi* Chigray, 2019, by original designation. Status: valid genus in Blaptinae: Blaptini: Blaptina.

*Medvedevoblaps* Bouchard & Bousquet, **new replacement name** [F]. Type species [automatic]: *Protoblapskashkarovi* G.S. Medvedev, 1998, by original designation. Status: valid genus in Blaptinae: Blaptini: Blaptina. Note: replacement name for *Protoblaps* G.S. Medvedev, 1998.

*Megacantha* Westwood, 1843: 121 [F]. Type species: *Megacanthatenebrosa* Westwood, 1843 (= *Helopsdentatus* Fabricius, 1801), by monotypy. Status: valid genus in Tenebrioninae: Amarygmini. Note: genus also described as new by Westwood (1844: 228).

*Megadasus* Reitter, 1904: 146 [M]. Type species: *Opatrumlefranci* Fairmaire, 1863, by original designation. Status: junior synonym of *Gonocephalum* Solier, 1834 in Blaptinae: Opatrini: Opatrina. Synonymy: Gebien (1939: 443).

*Megagenius* Solier, 1835b: 512, 513 [M]. Type species: *Megageniusfrioli* Solier, 1835, by monotypy. Status: valid genus in Pimeliinae: Tentyriini.

*Megalophrys* G.R. Waterhouse, 1845b: 321, 322 [F]. Type species: *Megalophryspatagonica* G.R. Waterhouse, 1845, by monotypy. Status: senior synonym of *Peltolobus* Lacordaire, 1859 in Pimeliinae: Trilobocarini. Note: although the older name *Megalophrys* Wagler, 1830 [Amphibia] was placed on the Official Index of Rejected and Invalid Generic Names in Zoology by the ICZN (1994b, Opinion 1763), it was not suppressed for the Principle of Homonymy and therefore remains available and the senior homonym.

*Megaprosodes* Reitter, 1909a: 118 [M]. Type species: *Prosodesstriata* Reitter, 1893, by original designation. Status: valid subgenus of *Prosodes* Eschscholtz, 1829 in Blaptinae: Blaptini: Prosodina.

*Megasattus* Casey, 1908: 56, 62 [M]. Type species: *Eusattuserosus* Horn, 1870, by original designation. Status: junior synonym of *Eusattus* J.L. LeConte, 1851 in Pimeliinae: Coniontini. Synonymy: Triplehorn (1968: 379).

*Megascythis* Keleinikova, 1963: 622 [F]. Type species: *Megascythispanfilovi* Keleinikova, 1963, by monotypy. Status: junior synonym of *Scythis* Kraatz, 1865 in Pimeliinae: Tentyriini. Synonymy: Skopin (1979: 171).

*Megasida* Casey, 1912: 77, 202 [F]. Type species: *Asidaobliterata* Champion, 1892, by original designation. Status: valid subgenus of *Stenomorpha* Solier, 1836 in Pimeliinae: Asidini.

*Megatenebrio* Gridelli, 1951: 221 [M]. Type species: *Tenebriogiganteus* Fairmaire, 1897, by monotypy. Status: valid subgenus of *Tenebrio* Linnaeus, 1758 in Tenebrioninae: Tenebrionini.

*Megatlasion* Koch, 1948: 427 [N]. Type species: *Micrositusatlantis* Escalera, 1914, by original designation. Status: junior synonym of *Atlasion* Koch, 1948 in Blaptinae: Dendarini: Melambiina. Synonymy: Antoine (1957: 351).

*Megeleates* Casey, 1895: 623 [M]. Type species: *Megeleatessequoiarum* Casey, 1895, by monotypy. Status: valid genus in Tenebrioninae: Bolitophagini.

*Megelenophorus* Gebien, 1910a: 121 [M]. Type species [automatic]: *Elenophorusamericanus* Lacordaire, 1830, by monotypy. Status: valid genus in Pimeliinae: Elenophorini: Megelenophorina. Note: replacement name for *Cacicus* Dejean, 1834.

*Megischia* Solier, 1835a: 245, 247 [F]. Type species: *Cistelacurvipes* Brullé, 1832, by monotypy. Status: valid genus in Alleculinae: Cteniopodini.

*Megischina* Reitter, 1906b: 118, 171 [F]. Type species: *Cistelaarmillata* Brullé, 1832, by subsequent designation (R. Lucas 1920: 399). Status: valid genus in Alleculinae: Cteniopodini.

*Meglyphus* Motschulsky, 1872: 38 [M]. Type species: *Meglyphuslaenoides* Motschulsky, 1872, by original designation. Status: valid genus in Blaptinae: Dendarini: Dendarina. Note: we act as First Revisers and reject the alternative original spelling *Megliphus*, used by Motschulsky (1872: 41).

*Meladeras* Mulsant & Rey, 1854: 191, 219 [N]. Type species: *Meladerasquadratulum* Mulsant & Rey, 1854, by subsequent designation (Viñolas 1990: 61). Status: valid subgenus of *Phylan* Sturm, 1826 in Blaptinae: Dendarini: Dendarina.

*Meladiesia* Reitter, 1909b: 309 [F]. Type species: *Meladiesiamiritarsis* Reitter, 1909, by monotypy. Status: valid genus in Pimeliinae: Pimeliini.

*Meladocrates* Reitter, 1904: 96 [M]. Type species: *Olocratesplaniusculus* Mulsant & Rey, 1854, by subsequent designation (Viñolas 1990: 60). Status: valid subgenus of *Phylan* Sturm, 1826 in Blaptinae: Dendarini: Dendarina.

*Melambasida* Reitter, 1917a: 10, 21 [F]. Type species: *Alphasidainterjecta* Reitter, 1917, by monotypy. Status: junior synonym of *Glabrasida* Escalera, 1910 in Pimeliinae: Asidini. Synonymy: Viñolas and Cartagena (2005: 282).

*Melambatlasus* Koch, 1948: 433 [M]. Type species: *Micrositushebes* Antoine, 1933, by original designation. Status: valid subgenus of *Melambius* Mulsant & Rey, 1854 in Blaptinae: Dendarini: Melambiina.

*Melambiophylax* Schuster, 1922: 48, 49 [M]. Type species: *Phylaxsardous* Baudi di Selve, 1876, by monotypy. Status: junior synonym of *Allophylax* Bedel, 1906 in Blaptinae: Dendarini: Melambiina. Synonymy: Leo (1994: 134).

*Melambius* Mulsant & Rey, 1854: 124 [M]. Type species: *Opatrumbarbarum* Erichson, 1841, by monotypy. Status: valid genus and subgenus in Blaptinae: Dendarini: Melambiina.

*Melanastus* Casey, 1907: 289, 353 [M]. Type species: *Eurymetoponatrum* J.L. LeConte, 1851, by original designation. Status: valid genus in Pimeliinae: Edrotini.

*Melancrus* Reiche & Saulcy, 1857: 190 [N]. Type species: *Melancruslaevigatum* Reiche & Saulcy, 1857, by subsequent designation (Löbl et al. 2008a: 42). Status: junior synonym of *Oxycara* Solier, 1835 in Pimeliinae: Tentyriini. Synonymy: Lacordaire (1859a: 57). Note: authors have assigned a masculine gender for this name in the literature; however, the correct gender is neuter; *Melancrus* has two roots: *melan*- and -*crus*, the first root is clearly Greek: *melas*, *melaina*, *melan* (an irregular adjective meaning dark or black) while the second is Latin: *crus* (the name for a leg, shank, or shin); the second word is a neuter noun and therefore the gender of *Melancrus* is neuter (ICZN 1999, Article 30.1.1).

*Melaneleodes* Blaisdell, 1909: 36 [M]. Type species: *Blapscarbonaria* Say, 1824, by subsequent designation (Triplehorn and Thomas 2012: 254). Status: valid subgenus of *Eleodes* Eschscholtz, 1829 in Blaptinae: Amphidorini.

*Melanesthes* Dejean, 1834: 191 [F]. Type species: *Opatrumsibiricum* Faldermann, 1833, by subsequent designation (Gebien 1939: 462). Status: valid genus and subgenus in Blaptinae: Opatrini: Opatrina.

*Melanimon* Motschulsky, 1845a: 78 [N]. Type species: *Microzoumcollare* Motschulsky, 1839, by monotypy. Status: senior synonym of *Platynosum* Mulsant & Rey, 1859 in Blaptinae: Opatrini: Sclerina. Synonymy: Seidlitz (1894: 413, 454). Note: junior homonym of *Melanimon* Steven, 1828 [Coleoptera: Tenebrionidae: Tenebrioninae: Melanimonini].

*Melanimon* Steven, 1828: 98 [N]. Type species: *Opatrumtibiale* Fabricius, 1781, by monotypy. Status: valid genus in Tenebrioninae: Melanimonini.

*Melanochrus* Wollaston, 1864: 467 [M]. Type species: *Melanochruslacordairii* Wollaston, 1864, by monotypy. Status: valid genus in Pimeliinae: Tentyriini.

*Melanocoma* Wollaston, 1868: 181 [F]. Type species: *Melanocomavestita* Wollaston, 1868, by monotypy. Status: valid genus in Blaptinae: Opatrini: Opatrina.

*Melanocratus* Fairmaire, 1895a: 21 [M]. Type species: *Melanocratusvalidipes* Fairmaire, 1895, by monotypy. Status: valid genus in Blaptinae: Platynotini: Platynotina.

*Melanolophus* Fairmaire in Fairmaire et al., 1882: 69 [M]. Type species: *Melanolophusseptemcostatus* Fairmaire, 1882, by monotypy. Status: valid genus in Pimeliinae: Sepidiini: Molurina.

*Melanophorus* Lacordaire, 1859a: 74 [M]. Type species [automatic]: *Melaphorusreichii* Guérin-Méneville, 1834, by monotypy. Status: junior synonym of *Melaphorus* Guérin-Méneville, 1834 in Pimeliinae: Evaniosomini. Note: unjustified emendation of *Melaphorus* Guérin-Méneville, 1834, not in prevailing usage.

*Melanopterus* Mulsant & Rey, 1854: 13, 14 [M]. Type species: *Melanopterusporcatus* Mulsant & Rey, 1854, by subsequent designation (Gebien 1938a: 292). Status: valid genus in Blaptinae: Platynotini: Platynotina.

*Melanostola* Solier, 1836: 123 [F]. Type species: *Pimeliasimplex* Solier, 1836, by monotypy. Status: valid subgenus of *Pimelia* Fabricius, 1775 in Pimeliinae: Pimeliini. Note: the name *Melanostola* was listed as synonym of *Pimelia* Fabricius, 1775 by Solier (1836; published by 16 May), being treated before 1961 as an available name and adopted as the name of a taxon (e.g., Dejean 1836 (published by 30 July), Hope 1841: 118); *Melanostola* was therefore made available from its first publication as a synonym (ICZN 1999, Article 11.6.1).

*Melansis* Wollaston, 1864: 491 [F]. Type species: *Phylaxcostatus* Brullé, 1839, by subsequent designation (Dallas 1865: 401). Status: valid genus in Blaptinae: Dendarini: Melambiina.

*Melaphorus* Guérin-Méneville, 1834: 13 [M]. Type species: *Melaphorusreichii* Guérin-Méneville, 1834, by monotypy. Status: valid genus in Pimeliinae: Evaniosomini.

*Melaps* Carter, 1908b: 409 [M]. Type species: *Melapscisteloides* Carter, 1908, by monotypy. Status: junior synonym of *Oocistela* Borchmann, 1908 in Alleculinae: Alleculini: Alleculina. Synonymy: Carter (1915a: 78). Note: although this name has been used as valid, with *Oocistela* Borchmann, 1908 as a synonym, in recent literature (e.g., Matthews and Lawrence 2019: 645), data on the date of publication of both names reveals that *Oocistela* was published first and has priority over *Melaps*.

*Melarachnica* Kraatz, 1865: 80, 174 [F]. Type species: *Melarachnicawestermanni* Kraatz, 1865 (= *Microderacoromandelensis* Solier, 1835), by monotypy. Status: junior synonym of *Nemapus* Solier, 1835 in Pimeliinae: Tentyriini. Synonymy: Koch (1943b: 790).

*Melasia* Perroud & Mulsant, 1856: 160 [F]. Type species: *Melasiagagatina* Perroud & Mulsant, 1856 (= *Tenebrioculinaris* Linnaeus, 1758), by subsequent designation (G.S. Medvedev 1990: 227). Status: junior synonym of *Uloma* Dejean, 1821 in Tenebrioninae: Ulomini. Synonymy: Lacordaire (1859a: 332).

*Melasma* Wollaston, 1864: 484 [N]. Type species: *Phylaxlineatus* Brullé, 1838, by monotypy. Status: senior synonym of *Melasmana* Strand, 1935 in Blaptinae: Dendarini: Melambiina. Note: junior homonym of *Melasma* Adams, 1854 [Mollusca].

*Melasmana* Strand, 1935a: 297 [F]. Type species [automatic]: *Phylaxlineatus* Brullé, 1838, by monotypy. Status: valid genus and subgenus in Blaptinae: Dendarini: Melambiina. Note: replacement name for *Melasma* Wollaston, 1864.

*Melasmocara* Reitter, 1900c: 95 [N]. Type species: *Melanochruslacordairii* Wollaston, 1864, by subsequent monotypy (Löbl et al. 2008a: 42). Status: junior synonym of *Melanochrus* Wollaston, 1864 in Pimeliinae: Tentyriini. Synonymy: Jakobson (1924: 243), Löbl et al. (2008a: 42). Note: originally proposed without included nominal species; Löbl et al. (2008a: 42), by including the species *Melanochruslacordairii* Wollaston, 1864 in association with this name, were the first authors to subsequently and expressly include nominal species in *Melasmocara* (ICZN 1999, Article 67.2.2).

*Melaxumia* Reitter, 1895: 280 [F]. Type species: *Melaxumiaacutangula* Reitter, 1895 (= *Tentyriaangulosa* Gebler, 1832), by monotypy. Status: valid genus in Pimeliinae: Tentyriini.

*Melobates* Kaszab, 1941a: 4, 23 [M]. Type species: *Melobatesbiroi* Kaszab, 1941, by original designation. Status: junior synonym of *Rehumius* Fairmaire, 1893 in Stenochiinae: Cnodalonini. Synonymy: Kaszab (1983d: 84).

*Melobrachys* Kaszab, 1960b: 273 [M]. Type species: *Melobrachyssarawakensis* Kaszab, 1960, by original designation. Status: valid genus in Stenochiinae: Cnodalonini.

*Menandris* Haag-Rutenberg, 1878: 103 [F]. Type species: *Menandrisaenea* Haag-Rutenberg, 1878, by monotypy. Status: valid genus in Stenochiinae: Cnodalonini.

*Mencheres* Champion, 1884: 5 [M]. Type species: *Mencheresnicaraguensis* Champion, 1884, by subsequent designation (R. Lucas 1920: 403). Status: valid genus in Pimeliinae: Edrotini.

*Menearchus* Carter, 1920a: 229 [M]. Type species: *Menearchusimpressosulcatus* Carter, 1920 (= *Helopsnigritus* Fabricius, 1777), by monotypy. Status: valid genus in Blaptinae: Platynotini: Platynotina. Note: as mentioned by Matthews and Bouchard (2008: 351) the type species was originally described from Australia in error, as the genus *Menearchus* Carter, 1920 is endemic to India and Sri Lanka.

*Menechides* Motschulsky, 1872: 26 [M]. Type species: *Helopscalcaratus* Fabricius, 1798, by original designation. Status: valid subgenus of *Centronopus* Solier, 1848 in Tenebrioninae: Centronopini.

*Menederes* Solier, 1848: 152, 203 [M]. Type species: *Menederesrufilabris* Solier, 1848, by original designation. Status: valid genus and subgenus in Blaptinae: Platynotini: Eurynotina.

*Menederopsis* Koch, 1954a: 16 [F]. Type species: *Menederopsisconstricta* Koch, 1954, by original designation. Status: valid genus in Blaptinae: Platynotini: Eurynotina.

*Menedrio* Motschulsky, 1872: 27 [M]. Type species: *Tenebrioobscurus* Fabricius, 1792, by original designation. Status: junior synonym of *Tenebrio* Linnaeus, 1758 in Tenebrioninae: Tenebrionini. Synonymy: Heyden in Heyden et al. (1883: 134).

*Menephilus* Mulsant, 1854: 291 [M]. Type species: *Tenebriocurvipes* Fabricius, 1792 (= *Tenebriocylindricus* Herbst, 1784), by monotypy. Status: valid genus in Stenochiinae: Cnodalonini.

*Meneristes* Pascoe, 1869: 150 [M]. Type species: *Meneristeslaticollis* Pascoe, 1869 (= *Tenebrioaustralis* Boisduval, 1835), by original designation. Status: valid genus in Tenebrioninae: Heleini: Asphalina.

*Menes* Champion, 1888: 442 [M]. Type species: *Menesmeridanus* Champion, 1888, by subsequent designation (Bousquet et al. 2015: 134). Status: valid genus in Alleculinae: Alleculini: Alleculina.

*Menimoides* Kaszab, 1946b: 19 [M]. Type species: *Menimoidestarandus* Kaszab, 1946, by original designation. Status: junior synonym of *Micropeneta* Pic, 1921 in Diaperinae: Gnathidiini: Gnathidiina. Synonymy: Kaszab (1978a: 172).

*Menimopsis* Champion, 1896: 16 [F]. Type species: *Menimopsisexcaecus* Champion, 1896, by monotypy. Status: valid genus in Diaperinae: Gnathidiini: Anopidiina.

*Menimus* Sharp, 1876: 73 [M]. Type species: *Menimusbatesi* Sharp, 1876, by subsequent designation (Gebien 1940: 429). Status: valid genus and subgenus in Diaperinae: Gnathidiini: Gnathidiina.

*Meniscophorus* Champion, 1889: 64 [M]. Type species: *Meniscophorusamazonicus* Champion, 1889, by subsequent designation (R. Lucas 1920: 404). Status: valid genus in Lagriinae: Lagriini: Statirina.

*Menoeceus* Champion, 1888: 443 [M]. Type species: *Menoeceuscrassicornis* Champion, 1888, by subsequent designation (Casey 1891: 122). Status: valid genus in Alleculinae: Alleculini: Alleculina.

*Menoncotus* Koch, 1954a: 34 [M]. Type species: *Oncotuschaetotaxicus* Koch, 1954, by original designation. Status: valid subgenus of *Oncotus* Blanchard, 1845 in Blaptinae: Platynotini: Eurynotina.

*Mentariobius* Koch, 1948: 425 [M]. Type species: *Micrositusdistinguendus* Mulsant & Rey, 1854, by original designation. Status: valid subgenus of *Hoplarion* Mulsant & Rey, 1854 in Blaptinae: Dendarini: Melambiina.

*Mentes* Champion, 1893a: 559 [M]. Type species: *Mentesruficollis* Champion, 1893, by subsequent designation (R. Lucas 1920: 404). Status: valid genus in Stenochiinae: Stenochiini.

*Meracantha* W. Kirby, 1837: 237 [F]. Type species: *Meracanthacanadensis* W. Kirby, 1837 (= *Helopscontractus* Palisot de Beauvois, 1811), by monotypy. Status: valid genus in Tenebrioninae: Amarygmini.

*Meracanthoides* Linell, 1896: 698 [M]. Type species: *Meracanthoidescupreolineatus* Linell, 1896, by monotypy. Status: valid subgenus of *Paramarygmus* Quedenfeldt, 1885 in Tenebrioninae: Amarygmini.

*Merinus* J.L. LeConte, 1862: 230 [M]. Type species: *Tenebriolaevis* G.-A. Olivier, 1795, by original designation. Status: valid genus in Stenochiinae: Cnodalonini. Note: the original combination of the name of the type species, *Tenebriolaevis* G.-A. Olivier, 1795, is a junior primary homonym of *Tenebriolaevis* Forskål, 1775.

*Merklia* Chen, 1997: 307 [F]. Type species: *Merkliabimaculata* Chen, 1997, by original designation. Status: valid genus in Lagriinae: Lagriini: Statirina.

*Meropersina* Reitter, 1909a: 117 [F]. Type species: *Prosodescordicollis* Allard, 1884, by original designation. Status: valid subgenus of *Prosodes* Eschscholtz, 1829 in Blaptinae: Blaptini: Prosodina.

*Meropria* Borchmann, 1921: 217, 228 [F]. Type species: *Statiraglabrata* Mäklin, 1863, by original designation. Status: valid genus in Lagriinae: Lagriini: Statirina.

*Merotemnus* Horn, 1870: 364, 367 [M]. Type species: *Merotemnuselongatus* Horn, 1870 (= *Ulomafiliforme* Laporte, 1840), by monotypy. Status: junior synonym of *Adelonia* Laporte, 1840 in Lagriinae: Belopini. Synonymy: Spilman (1961: 49).

*Meroxys* Ardoin, 1963a: 132 [M]. Type species: *Meroxyshaafi* Ardoin, 1963, by original designation. Status: valid genus in Tenebrioninae: Amarygmini. Note: name first proposed by Ardoin (1962b: 970) without fixation of a type species in the original publication (ICZN 1999, Article 13.3).

*Mesabates* Champion, 1884: 3 [M]. Type species: *Mesabateslatifrons* Champion, 1884, by monotypy. Status: valid genus in Pimeliinae: Edrotini.

*Mesabatodes* Casey, 1907: 517 [M]. Type species: *Mesabatesinaequalis* Champion, 1892, by original designation. Status: valid genus in Pimeliinae: Edrotini.

*Mesoblaps* Bauer, 1921: 231 [F]. Type species: *Blaps rugulipennis* Fairmaire, 1891, by monotypy. Status: junior synonym of *Blaps* Fabricius, 1775 in Blaptinae: Blaptini: Blaptina. Synonymy: Nabozhenko and Chigray (2020: 10).

*Mesohelops* Reitter, 1922a: 31 [M]. Type species: *Helopscyanipes* Allard, 1877, by subsequent designation (Nabozhenko and Löbl 2008: 251). Status: junior synonym of *Helops* Fabricius, 1775 in Tenebrioninae: Helopini: Helopina. Synonymy: Nabozhenko and Keskin (2017: 41). Note: the original combination of the name of the type species, *Helopscyanipes* Allard, 1877, is a junior primary homonym of *Helopscyanipes* Fabricius, 1801.

*Mesoleptodes* G.S. Medvedev & Iljina, 2007: 881 [M]. Type species: *Leptodessemenowi* Reitter, 1892, by original designation. Status: valid subgenus of *Leptodes* Dejean, 1834 in Pimeliinae: Leptodini.

*Mesolobopoda* Campbell, 1966: 34 [F]. Type species: *Alleculasocia* J.L. LeConte, 1854, by original designation. Status: valid subgenus of *Lobopoda* Solier, 1835 in Alleculinae: Alleculini: Alleculina.

*Mesomorphus* Miedel, 1880: 140 [M]. Type species: *Opatrummurinum* Baudi di Selve, 1876 (= *Opatrinussetosus* Mulsant & Rey, 1853), by subsequent designation (Gebien 1938a: 399). Status: valid genus in Blaptinae: Opatrini: Opatrina.

*Mesopatrum* Broun, 1893b: 1355 [N]. Type species: *Mesopatrumgranulosum* Broun, 1893, by monotypy. Status: valid genus in Lagriinae: Adeliini.

*Mesopraocis* Flores & Pizarro-Araya, 2014: 60 [M]. Type species: *Praociscalderanus* Kulzer, 1958, by original designation. Status: valid subgenus of *Praocis* Eschscholtz, 1829 in Pimeliinae: Praociini. Note: this name was first proposed by Kulzer (1958a: 12, 31) without type species designation.

*Mesoprosodes* G.S. Medvedev, 1995b: 827 [M]. Type species: *Prosodesnuratensis* Semenov, 1894, by original designation. Status: valid subgenus of *Prosodes* Eschscholtz, 1829 in Blaptinae: Blaptini: Prosodina.

*Mesopterocoma* Skopin, 1974b: 147 [F]. Type species: *Pterocomasemicarinata* Bates, 1879, by original designation. Status: valid subgenus of *Pterocoma* Dejean, 1834 in Pimeliinae: Pimeliini.

*Mesostena* Eschscholtz, 1831: 5, 9 [F]. Type species: *Mesostenapunctata* Eschscholtz, 1831 (= *Pimeliaangustata* Fabricius, 1775), by monotypy. Status: valid genus and subgenus in Pimeliinae: Tentyriini.

*Mesostenopa* Kraatz, 1865: 80, 179 [F]. Type species: *Mesostenopapicea* Kraatz, 1865, by subsequent designation (Gebien 1937a: 608). Status: valid subgenus of *Mesostena* Eschscholtz, 1831 in Pimeliinae: Tentyriini.

*Mesosternoplax* Skopin, 1973: 110, 142 [F]. Type species: *Trigonoscelislaeviuscula* Kraatz, 1882, by original designation. Status: valid subgenus of *Sternoplax* Frivaldszky, 1890 in Pimeliinae: Pimeliini.

*Mesotretis* Bates, 1872c: 151 [F]. Type species: *Mesotretisferruginea* Bates, 1872, by monotypy. Status: valid genus in Lagriinae: Lupropini.

*Messalia* Pascoe, 1883: 442 [F]. Type species: *Messaliavarians* Pascoe, 1883, by monotypy. Status: junior synonym of *Strongylium* W. Kirby, 1819 in Stenochiinae: Stenochiini. Synonymy: Gebien (1948: 519).

*Messoricolum* Koch, 1960: 384 [N]. Type species: *Messoricolumscotti* Koch, 1960, by original designation. Status: valid genus in Blaptinae: Opatrini: Ammobiina.

*Metablapylis* Blaisdell, 1909: 391 [F]. Type species: *Eleodesnigrina* J.L. LeConte, 1858, by subsequent designation (Bousquet et al. 2018: 159). Status: valid subgenus of *Eleodes* Eschscholtz, 1829 in Blaptinae: Amphidorini.

*Metabolocerus* Bates, 1873c: 259 [M]. Type species: *Metaboloceruspilosus* Bates, 1873, by subsequent designation (R. Lucas 1920: 407). Status: valid genus in Tenebrioninae: Ulomini.

*Metaclisa* Jacquelin du Val, 1861: 296 [F]. Type species: *Platydemaparallela* Fairmaire, 1855 (= *Diaperisazurea* Waltl, 1838), by original designation. Status: valid genus in Tenebrioninae: Metaclisini. Note: nomen protectum (see Bouchard et al. 2007: 393).

*Metacorticeus* Bremer & Lillig, 2017a: 71 [M]. Type species: *Corticeusornatus* Bremer, 1993, by original designation. Status: valid subgenus of *Corticeus* Piller & Mitterpacher, 1783 in Diaperinae: Hypophlaeini.

*Metacossyphodes* Andreae, 1961: 205, 215 [M]. Type species: *Cossyphodeskundelunguensis* Basilewsky, 1950, by original designation. Status: junior synonym of *Cossyphodes* Westwood, 1851 in Pimeliinae: Cossyphodini: Cossyphodina. Synonymy: Schawaller (2013c: 362, implied by inclusion of *Cossyphodeskundelunguensis* Basilewsky, 1950 in Cossyphodes Westwood, 1851 without use of a subgenus rank).

*Metallonotus* Gray in Griffith and Pidgeon, 1832: 790 [M]. Type species: *Metallonotusdenticollis* Gray, 1832, by monotypy. Status: valid genus in Lagriinae: Pycnocerini.

*Methistamena* Gebien, 1919: 28, 151 [F]. Type species: *Methistamenaclavipes* Gebien, 1919, by original designation. Status: junior synonym of *Camariomorpha* Pic, 1915 in Stenochiinae: Cnodalonini. Synonymy: Kulzer (1954a: 71).

*Metisopus* Bates, 1873e: 371 [M]. Type species: *Metisopuspurpureipennis* Bates, 1873, by monotypy. Status: valid genus in Stenochiinae: Cnodalonini.

*Metistete* Pascoe, 1866a: 489 [F]. Type species: *Tanychilusgibbicollis* Newman, 1838, by original designation. Status: valid genus in Alleculinae: Alleculini: Alleculina.

*Metonites* Gistel, 1848a: viii, 126 [M]. Type species [automatic]: *Blapidaokeni* Perty, 1830, by monotypy. Status: junior synonym of *Blapida* Perty, 1830 in Stenochiinae: Cnodalonini. Note: unnecessary replacement name for *Blapida* Perty, 1830.

*Metopoloba* Casey, 1907: 379 [F]. Type species: *Epitraguspruinosus* Horn, 1870, by original designation. Status: valid genus in Pimeliinae: Epitragini.

*Metoponiopsis* Casey, 1907: 290 [F]. Type species: *Eurymetoponbicolor* Horn, 1870, by monotypy. Status: valid subgenus of *Metoponium* Casey, 1907 in Pimeliinae: Edrotini.

*Metoponium* Casey, 1907: 288 [N]. Type species: *Eurymetoponabnorme* J.L. LeConte, 1851, by original designation. Status: valid genus and subgenus in Pimeliinae: Edrotini.

*Metriolagria* Merkl, 1987: 124, 138 [F]. Type species: *Lagriaaffinis* Boisduval, 1835, by original designation. Status: valid genus in Lagriinae: Lagriini: Lagriina.

*Metriopus* Solier, 1835b: 512, 570 [M]. Type species: *Metriopushoffmanseggii* Solier, 1835, by monotypy. Status: valid genus and subgenus in Pimeliinae: Adesmiini.

*Metulosonia* Bates, 1873c: 261 [F]. Type species: *Metulosoniahorni* Bates, 1873, by subsequent designation (Gebien 1940: 1061). Status: valid genus in Tenebrioninae: Triboliini.

*Micipsa* P.H. Lucas, 1855: xxxiv [M]. Type species: *Micipsarufitarsis* P.H. Lucas, 1855 (= *Pimeliamulsanti* Levrat, 1853), by monotypy. Status: valid genus and subgenus in Pimeliinae: Tentyriini.

*Micipsina* Reitter, 1900c: 94, 188 [F]. Type species: *Micipsinarolphi* Reitter, 1900, by monotypy. Status: junior synonym of *Thalpobia* Fairmaire, 1871 in Pimeliinae: Tentyriini. Synonymy: Escalera (1914: 279).

*Micrantereus* Solier, 1848: 151, 175 [M]. Type species: *Acanthomerusanomalus* Guérin-Méneville, 1834, by original designation. Status: valid genus in Blaptinae: Pedinini: Helopinina.

*Micrarmalia* Casey, 1907: 516 [F]. Type species: *Emmenastusconstrictus* Champion, 1892, by monotypy. Status: valid genus in Pimeliinae: Edrotini.

*Micrasida* Smith, 2013: 608 [F]. Type species: *Micrasidaobrienorum* Smith, 2013, by original designation. Status: valid genus in Pimeliinae: Asidini.

*Micrectyche* Bates, 1873e: 362 [F]. Type species: *Micrectycheintermedia* Bates, 1873, by subsequent designation (R. Lucas 1920: 412). Status: valid genus in Diaperinae: Ectychini.

*Micreuphlaeus* Fairmaire, 1897e: 223 [M]. Type species: *Micreuphlaeusasperipellis* Fairmaire, 1897, by monotypy. Status: valid genus in Stenochiinae: Cnodalonini.

*Micrisomira* Pic, 1930a: 30 [F]. Type species: *Micrisomiraruficollis* Pic, 1930, by monotypy. Status: valid genus in Alleculinae: Alleculini: Gonoderina.

*Microamarygmus* Pic, 1915d: 8 [M]. Type species: *Microamarygmusmadurensis* Pic, 1915, by monotypy. Status: valid genus in Alleculinae: incertae sedis.

*Microanaedus* Pic, 1923b: 16 [M]. Type species: *Microanaedusnotatus* Pic, 1923, by monotypy. Status: valid genus in Lagriinae: Goniaderini.

*Microatasthalus* Ando, 2010: 153 [M]. Type species: *Microatasthalushadrocerus* Ando, 2010, by original designation. Status: valid genus in Tenebrioninae: Bolitophagini.

*Microbasanus* Pic, 1921a: 1 [M]. Type species: *Microbasanusjureceki* Pic, 1921, by monotypy. Status: junior synonym of *Scaphidema* Redtenbacher, 1848 in Diaperinae: Scaphidemini. Synonymy: Löbl et al. (2008b: 318), see also Schawaller (2008: 384).

*Microblattellus* Ferrer, 2006a: 78 [M]. Type species: *Microblattelluslecongmani* Ferrer, 2006, by original designation. Status: valid genus in Tenebrioninae: Falsocossyphini.

*Microblemma* Semenov, 1889: 213 [N]. Type species: *Microblemmasimplex* Semenov, 1889, by monotypy. Status: valid genus in Pimeliinae: Stenosini: Platamodina.

*Microbolitonaeus* Grimm, 2014: 194 [M]. Type species: *Microbolitonaeusarmatus* Grimm, 2014, by original designation. Status: valid genus in Tenebrioninae: Bolitophagini.

*Microbradymerus* Schawaller, 1999a: 144 [M]. Type species: *Microbradymerusmerkli* Schawaller, 1999, by original designation. Status: valid genus in Stenochiinae: Cnodalonini.

*Microcalcar* Pic, 1925b: 9 [N]. Type species: *Belopusinstriatus* Pic, 1925, by monotypy. Status: valid genus in Lagriinae: Lupropini.

*Microcalydonis* Pic, 1923d: 17 [F]. Type species: *Microcalydonismetallica* Pic, 1923, by monotypy. Status: valid subgenus of *Osternus* Fairmaire, 1895 in Stenochiinae: Cnodalonini.

*Microcameria* Ren, 1998: 108, 113 [F]. Type species: *Microcameriapygmaea* Ren, 1998, by original designation. Status: junior synonym of *Foochounus* Pic, 1921 in Stenochiinae: Cnodalonini. Synonymy: Ando (2008: 39).

*Microcatomus* Pic, 1925b: 7 [M]. Type species: *Microcatomuslongipilis* Pic, 1925, by monotypy. Status: valid genus in Tenebrioninae: Helopini: incertae sedis. Note: Nabozhenko (2018: 183) mentioned that the position of this genus within the tribe Helopini is unclear.

*Microcenoscelis* Schawaller, 2015: 438 [F]. Type species: *Microcenosceliscaeca* Schawaller, 2015, by original designation. Status: valid genus in Tenebrioninae: Ulomini.

*Microcilibe* Carter, 1919: 147 [F]. Type species: *Microcilibecastanea* Carter, 1919, by monotypy. Status: junior synonym of *Menimus* Sharp, 1876 in Diaperinae: Gnathidiini: Gnathidiina. Synonymy: Kaszab (1978a: 173).

*Microcistela* Pic, 1904: 26 [F]. Type species: *Microcistelarosinae* Pic, 1904, by monotypy. Status: valid genus in Alleculinae: Alleculini: Gonoderina.

*Microcistela* Pic, 1919b: 6 [F]. Type species: *Microcistelaobscura* Pic, 1919, by monotypy. Status: senior synonym of *Microcistelopsis* Pic, 1922 in Alleculinae: Alleculini: Alleculina. Note: junior homonym of *Microcistela* Pic, 1904 [Coleoptera: Tenebrionidae: Alleculinae: Alleculini: Gonoderina].

*Microcistelopsis* Pic, 1922d: 20 [F]. Type species [automatic]: *Microcistelaobscura* Pic, 1919, by monotypy. Status: valid genus in Alleculinae: Alleculini: Alleculina. Note: replacement name for *Microcistela* Pic, 1919.

*Microcrypticus* Gebien, 1921b: 7 [M]. Type species: *Diaperisvariegata* Klug, 1833, by original designation. Status: valid genus and subgenus in Diaperinae: Crypticini.

*Microdendarus* Escalera, 1944: 84 [M]. Type species: *Dendarusschusteri* Español, 1937, by original designation. Status: junior synonym of *Dendarus* Dejean, 1821 in Blaptinae: Dendarini: Dendarina. Synonymy: Español (1961a: 44).

*Microdera* Eschscholtz, 1831: 5, 6 [F]. Type species: *Tentyriadeserta* Tauscher, 1812, by subsequent designation (Gebien 1937a: 619). Status: valid genus and subgenus in Pimeliinae: Tentyriini.

*Microderopsis* Haag-Rutenberg, 1876: 86 [F]. Type species: *Microderopsisbenguelensis* Haag-Rutenberg, 1876, by monotypy. Status: valid genus in Pimeliinae: Tentyriini.

*Microdisema* Pic, 1917b: 2, 3 [F]. Type species: *Disemagounellei* Pic, 1912, by monotypy. Status: valid subgenus of *Barsenis* Pascoe, 1887 in Lagriinae: Lagriini: Statirina. Note: this genus was established without included nominal species; *Disemagounellei* Pic, 1912 was first and expressly included in *Microdisema* by Pic (1937c: 36).

*Microdocnemis* Nabozhenko & Keskin, 2010: 841 [F]. Type species: *Microdocnemisxerophilica* Nabozhenko & Keskin, 2010, by original designation. Status: valid genus in Tenebrioninae: Helopini: Cylindrinotina.

*Microeucyrtus* Pic, 1926a: 47 [M]. Type species: *Microeucyrtuscoomani* Pic, 1926, by monotypy. Status: junior synonym of *Eucyrtus* Lacordaire, 1859 in Stenochiinae: Cnodalonini. Synonymy: Gebien (1941: 1139).

*Microgauromaia* Pic, 1921d: 23 [F]. Type species: *Microgauromaiaminuta* Pic, 1921, by monotypy. Status: junior synonym of *Phaedis* Pascoe, 1866 in Stenochiinae: Cnodalonini. Synonymy: Ando (2007: 164).

*Microgoniadera* Pic, 1917d: 9 [F]. Type species: *Microgoniaderahirsuta* Pic, 1917, by monotypy. Status: valid genus in Lagriinae: Goniaderini.

*Microgonocnemis* Pic, 1936b: 16 [F]. Type species: *Microgonocnemiscarinata* Pic, 1936, by monotypy. Status: valid subgenus of *Paragonocnemis* Kraatz, 1899 in Tenebrioninae: Amarygmini.

*Microhedyphanes* Nabozhenko & Lillig, 2013: 189 [M]. Type species: *Hedyphaneschikatunovi* Nabozhenko & Lillig, 2013, by original designation. Status: valid subgenus of *Hedyphanes* Fischer, 1820 in Tenebrioninae: Helopini: Helopina.

*Microhemicera* Pic, 1921d: 30 [F]. Type species: *Hemicerahumeralis* Pic, 1921, by monotypy. Status: junior synonym of *Simalura* Gebien, 1914 in Stenochiinae: Cnodalonini. Synonymy: Löbl et al. (2008b: 347).

*Microhionthis* Blair, 1923b: 120 [F]. Type species: *Microhionthispatriciae* Blair, 1923 (= *Falsocatomuluseuphraticus* Pic, 1914), by monotypy. Status: junior synonym of *Falsocatomulus* Pic, 1914 in Pimeliinae: Tentyriini. Synonymy: Koch (1941: 267).

*Microlagria* Seidlitz, 1898b: 336, 339 [F]. Type species: *Lagriapoupillieri* Reiche, 1864, by monotypy. Status: junior synonym of *Adynata* Fåhraeus, 1870 in Lagriinae: Lagriini: Lagriina. Synonymy: Borchmann (1936: 23).

*Microleichenum* G.S. Medvedev, 1973: 648 [N]. Type species: *Microleichenumchoresmense* G.S. Medvedev, 1973, by original designation. Status: junior synonym of *Apsheronellus* Bogatchev, 1967 in Blaptinae: Pedinini: Leichenina. Synonymy: G.S. Medvedev and Iwan (2006: 614).

*Microlyprops* Kaszab, 1939a: 108 [M]. Type species: *Microlypropsceylonicus* Kaszab, 1939, by original designation. Status: valid genus in Lagriinae: Goniaderini.

*Micromenandris* Kaszab, 1955a: 511, 513 [F]. Type species: *Micromenandrismirabilis* Kaszab, 1955, by original designation. Status: valid genus in Stenochiinae: Cnodalonini.

*Micromes* Casey, 1907: 432, 441 [M]. Type species: *Stibiaovipennis* Horn, 1874, by original designation. Status: valid genus in Pimeliinae: Edrotini.

*Micromophlus* Znojko in Ogloblin and Znojko, 1950: 124 [M]. Type species: *Omophlussubtilis* Solsky, 1881, by monotypy. Status: valid subgenus of *Omophlus* Dejean, 1834 in Alleculinae: Cteniopodini.

*Micronilio* Pic, 1936c: 198 [M]. Type species: *Niliopunctatus* Pic, 1918 (= *Niliogounellei* Ihering, 1914), by monotypy. Status: valid subgenus of *Nilio* Latreille, 1802 in Nilioninae.

*Microomopheres* Freude, 1993: 214 [M]. Type species: *Omopheresbrendelli* Freude, 1993, by monotypy. Status: valid subgenus of *Omopheres* Casey, 1907 in Pimeliinae: Epitragini.

*Micropedinus* Lewis, 1894: 379 [M]. Type species: *Micropedinusalgae* Lewis, 1894 (= *Heterophagapullulus* Boheman, 1858), by subsequent designation (R. Lucas 1920: 415). Status: valid genus in Lagriinae: Lupropini.

*Micropeltoides* Pic, 1916d: 16 [M]. Type species: *Micropeltoidescrypticoides* Pic, 1916, by monotypy. Status: valid subgenus of *Peltoides* Laporte, 1833 in Tenebrioninae: Alphitobiini.

*Micropeneta* Pic, 1921d: 19 [F]. Type species: *Micropenetatestacea* Pic, 1921, by subsequent designation (Riley 1923: 128). Status: valid genus in Diaperinae: Gnathidiini: Gnathidiina.

*Microphenus* Gebien, 1921a: 324, 338 [M]. Type species: *Microphenuscordicollis* Gebien, 1921 (= *Espitesobscurus* Blair, 1915), by monotypy. Status: valid genus in Stenochiinae: Cnodalonini.

*Microphligra* Koch, 1955a: 47 [F]. Type species: *Phligraminuta* Péringuey, 1904, by original designation. Status: valid genus in Pimeliinae: Sepidiini: Trachynotina.

*Microphyes* W.J. MacLeay, 1872: 286 [M]. Type species: *Microphyesrufipes* W.J. MacLeay, 1872 (= *Opatrumlaevigatum* Fabricius, 1781), by monotypy. Status: junior synonym of *Alphitobius* Stephens, 1829 in Tenebrioninae: Alphitobiini. Synonymy: Blair (1914: 486).

*Microphylacinus* Iwan, Kamiński & Aalbu, 2011: 2 [M]. Type species: *Microphylacinusverendus* Iwan, Kamiński & Aalbu, 2011, by original designation. Status: valid genus in Blaptinae: Dendarini: Dendarina.

*Microplatyscelis* Kaszab, 1940a: 142, 144 [F]. Type species: *Faustiaseriepunctata* Reitter, 1890, by original designation. Status: valid genus in Blaptinae: Platyscelidini.

*Microprostenus* Pic, 1921d: 14 [M]. Type species: *Microprostenuslongicornis* Pic, 1921, by monotypy. Status: valid genus in Alleculinae: Alleculini: Xystropodina.

*Micropseudochillus* Fouquè, 2015: 230, 240 [M]. Type species: *Pseudochilluspalawanus* Fouquè, 2015, by original designation. Status: valid subgenus of *Pseudochillus* Fouquè, 2015 in Pimeliinae: Stenosini: Dichillina.

*Microschatia* Solier, 1836: 406, 474 [F]. Type species: *Microschatiapunctata* Solier, 1836, by monotypy. Status: valid genus in Pimeliinae: Asidini.

*Microselinus* Koch, 1956a: 214 [M]. Type species: *Microselinusmuelleri* Koch, 1956, by original designation. Status: junior synonym of *Glyptopteryx* Gebien, 1910 in Blaptinae: Platynotini: Platynotina. Synonymy: Kamiński (2015a: 91).

*Microsis* Koch, 1958: 76, 82 [F]. Type species: *Microsisvilhenai* Koch, 1958, by original designation. Status: valid subgenus of *Zophosis* Latreille, 1802 in Pimeliinae: Zophosini.

*Micrositus* Mulsant & Rey, 1854: 131, 148 [M]. Type species: *Micrositusorbicularis* Mulsant & Rey, 1854, by subsequent designation (Gebien 1938a: 415). Status: valid genus in Blaptinae: Dendarini: Dendarina.

*Microsphaerotus* Pic, 1928b: 10 [M]. Type species: *Microsphaerotusruficornis* Pic, 1928, by monotypy. Status: valid genus in Stenochiinae: Cnodalonini.

*Microstenogena* Pic, 1924a: 30 [F]. Type species: *Microstenogenaruficornis* Pic, 1924, by monotypy. Status: valid genus in Alleculinae: Alleculini: Alleculina.

*Microsthes* Novák, 2011: 322 [M]. Type species: *Microsthesbarborae* Novák, 2011, by original designation. Status: valid genus in Alleculinae: Alleculini: Alleculina.

*Microstizopus* Koch, 1963: 36 [M]. Type species: *Microstizopusciliatus* Koch, 1963, by original designation. Status: valid genus in Blaptinae: Opatrini: Stizopodina.

*Microstrongylium* Pic, 1917d: 13 [N]. Type species: *Microstrongyliumcyanicolle* Pic, 1917, by monotypy. Status: junior synonym of *Strongylium* W. Kirby, 1819 in Stenochiinae: Stenochiini. Synonymy: Kaszab (1977b: 30).

*Microtelopsis* Koch, 1940b: 742 [F]. Type species: *Tetranillussimplicifrons* Gridelli, 1934, by monotypy. Status: valid genus and subgenus in Pimeliinae: Stenosini: Stenosina. Note: we act as First Revisers and select *Microtelopsis* Koch, 1940 as the valid name for this genus instead of *Extetranosis* Koch, 1940 and *Hypermicrotelopsis* Koch, 1940.

*Microtelus* Solier, 1838b: 7, 9 [M]. Type species: *Microtelusasiaticus* Solier, 1838, by original designation. Status: valid genus in Pimeliinae: Stenosini: Dichillina.

*Microthelecta* Pic, 1952a: 65 [F]. Type species: *Cylindrothorusbraunsi* Pic, 1952, by monotypy. Status: valid subgenus of *Cylindrothorus* Solier, 1843 in Alleculinae: Alleculini: Mycetocharina.

*Microtocerus* Pic, 1918a: 11 [M]. Type species: *Microtocerusgrandicornis* Pic, 1918, by monotypy. Status: valid genus in Stenochiinae: Stenochiini.

*Microzoon* Hope, 1841: 112 [N]. Type species [automatic]: *Opatrumtibiale* Fabricius, 1781, by monotypy. Status: junior synonym of *Melanimon* Steven, 1828 in Tenebrioninae: Melanimonini. Note: unjustified emendation of *Microzoum* Dejean, 1834, not in prevailing usage.

*Microzophobas* Pic, 1944: 7 [M]. Type species: *Microzophobasluteomaculatus* Pic, 1944, by monotypy. Status: valid genus in Tenebrioninae: Tenebrionini.

*Microzoum* Dejean, 1834: 193 [N]. Type species: *Opatrumtibiale* Fabricius, 1781, by monotypy. Status: junior synonym of *Melanimon* Steven, 1828 in Tenebrioninae: Melanimonini. Synonymy: Seidlitz (1894: 452).

*Mictopsis* Fairmaire, 1899e: 538 [F]. Type species: *Mictopsislaticollis* Fairmaire, 1899, by monotypy. Status: valid genus in Stenochiinae: Stenochiini.

*Miglica* Reitter, 1904: 171 [F]. Type species: *Pedinuslaticollis* Gebler, 1830, by subsequent designation (Iwan and Löbl 2008: 267). Status: junior synonym of *Melanesthes* Dejean, 1834 in Blaptinae: Opatrini: Opatrina. Synonymy: Reichardt (1936: 175).

*Millotella* Koch, 1962a: 116, 130 [F]. Type species: *Millotellamicrocornis* Koch, 1962, by original designation. Status: senior synonym of *Kochotella* Bouchard & Bousquet, **nom. nov.** in Pimeliinae: Asidini. Note: junior homonym of *Millotella* Poisson, 1949 [Hemiptera].

*Millstreamia* G.S. Medvedev & Lawrence, 1984: 579 [F]. Type species: *Csiroparodoxa* G.S. Medvedev & Lawrence, 1984, by original designation. Status: valid subgenus of *Csiro* G.S. Medvedev & Lawrence, 1984 in Diaperinae: Hyociini: Hyociina.

*Miltoprepes* Gerstaecker, 1871: 65 [M]. Type species: *Miltoprepeslaetus* Gerstaecker, 1871, by monotypy. Status: valid genus in Tenebrioninae: Praeugenini.

*Mimelasida* Reitter, 1917a: 10, 21 [F]. Type species: *Asidapuncticollis* Solier, 1836, by subsequent designation (F. Soldati 2008: 33). Status: junior synonym of *Glabrasida* Escalera, 1910 in Pimeliinae: Asidini. Synonymy: Viñolas and Cartagena (2005: 282).

*Mimoborchmania* Pic, 1934a: 31 [F]. Type species: *Nemostiracoloripes* Pic, 1922, by monotypy. Status: valid genus in Lagriinae: Lagriini: Statirina.

*Mimocellus* Wasmann, 1904: 11 [M]. Type species: *Mimocellustrechoides* Wasmann, 1904, by subsequent designation (R. Lucas 1920: 419). Status: valid genus in Lagriinae: Lupropini.

*Mimocistela* Borchmann, 1938: 124 [F]. Type species: *Mimocistelazumpti* Borchmann, 1938, by original designation. Status: valid genus in Alleculinae: incertae sedis.

*Mimocossyphus* Pic, 1923a: 5 [M]. Type species: *Mimocossyphusminor* Pic, 1923, by monotypy. Status: valid genus in Pimeliinae: Cossyphodini: incertae sedis. Note: Schawaller (2013c: 362) mentioned that the position of this taxon within Cossyphodini is doubtful.

*Mimogoueum* Pic, 1952d: 10 [N]. Type species: *Mimogoueumhutteli* Pic, 1952, by monotypy. Status: junior synonym of *Strongylium* W. Kirby, 1819 in Stenochiinae: Stenochiini. Synonymy: Ardoin (1962a: 64).

†*Mimohelops* Haupt, 1950: 114, 130 [M]. Type species: *Mimohelopsvenosus* Haupt, 1950, by original designation. Status: valid genus in Stenochiinae: incertae sedis. Note: described from Middle Eocene deposits (Germany).

*Mimolaena* Ardoin, 1961c: 36 [F]. Type species: *Mimolaenapauliani* Ardoin, 1961, by monotypy. Status: valid genus in Lagriinae: Laenini.

*Mimolagria* Pic, 1927a: 44 [F]. Type species: *Mimolagriabruchi* Pic, 1927, by monotypy. Status: valid genus in Lagriinae: Lagriini: Lagriina.

*Mimopeus* Pascoe, 1866a: 477 [M]. Type species: *Mimopeusamaroides* Pascoe, 1866 (= *Celibeelongata* Brême, 1842), by monotypy. Status: valid genus in Tenebrioninae: Heleini: Heleina. Note: as mentioned by Matthews and Bouchard (2008: 350) the type species was originally described from Australia in error, the genus *Mimopeus* Pascoe, 1866 is endemic to New Zealand; Matthews and Lawrence (2019: 629) mentioned that this genus seems indistinguishable from the Australian genus *Celibe* Boisduval, 1835.

*Mimopraogena* Pic, 1952d: 10 [F]. Type species: *Alleculametallicipennis* Pic, 1942, by monotypy. Status: valid genus in Alleculinae: Alleculini: Alleculina.

*Mimosynopticus* Pic, 1922d: 25 [M]. Type species: *Mimosynopticusparvulus* Pic, 1922, by monotypy. Status: valid genus in Tenebrioninae: Amarygmini.

*Mimothydemus* Pic, 1923c: 20 [M]. Type species: *Mimothydemusangustatus* Pic, 1923 (= *Lophocnemiscyaneus* Kraatz, 1880), by monotypy. Status: junior synonym of *Lophocnemis* Mäklin, 1867 in Stenochiinae: Stenochiini. Synonymy: Kulzer (1966: 352).

*Mimoxenotermes* Pic, 1931a: 106 [M]. Type species: *Mimoxenotermesduporti* Pic, 1931, by monotypy. Status: valid genus in Tenebrioninae: Rhysopaussini.

*Mimuroplatopsis* Borchmann, 1936: 485 [F]. Type species: *Mimuroplatopsisatricolor* Pic, 1931, by original designation. Status: valid genus in Lagriinae: Lagriini: Statirina. Note: this genus was proposed earlier by Pic (1931b: 32) without fixation of a type species in the original publication (ICZN 1999, Article 13.3).

*Minasius* Pic, 1932: 18 [M]. Type species: *Minasiusopacipennis* Pic, 1932, by monotypy. Status: valid genus in Lagriinae: Lagriini: Lagriina.

*Minorus* Mulsant & Rey, 1854: 41 [M]. Type species: *Eurynotusrugicollis* Mulsant & Rey, 1854, by monotypy. Status: valid genus in Blaptinae: Dendarini: Melambiina.

†*Miostenosis* Wickham,1913: 297 [F]. Type species: *Miostenosislacordairei* Wickham, 1913, by original designation. Status: valid genus in Pimeliinae: Stenosini: incertae sedis. Note: described from Upper Eocene deposits (USA).

*Miotodera* Fairmaire, 1901b: 190 [F]. Type species: *Miotoderafuneraria* Fairmaire, 1901, by monotypy. Status: valid genus in Stenochiinae: Stenochiini.

*Mireanopidium* Dajoz, 1977: 240 [N]. Type species: *Mireanopidiumcamerunense* Dajoz, 1977, by original designation. Status: valid genus in Diaperinae: Gnathidiini: incertae sedis. Note: placed in “Gnathidiini incertae sedis” by Schawaller and Purchart (2012: 312).

*Miripronotum* Louw, 1979: 117, 118 [N]. Type species: *Miripronotumprominoculatum* Louw, 1979, by original designation. Status: valid genus in Pimeliinae: Sepidiini: Oxurina.

*Misolampidius* Solsky, 1876: 292 [M]. Type species: *Misolampidiustentyrioides* Solsky, 1876, by monotypy. Status: valid genus in Stenochiinae: Cnodalonini.

*Misolampomorphus* Kaszab, 1941a: 2, 6 [M]. Type species: *Misolampomorphuskochi* Kaszab, 1941 (= *Leprocaulusreitteri* Pic, 1934), by original designation. Status: valid genus in Stenochiinae: Cnodalonini.

*Misolampus* Latreille, 1806: 160 [M]. Type species: *Misolampushoffmannseggii* Latreille, 1806 (= *Pimeliagibbula* Herbst, 1799), by monotypy. Status: valid genus in Stenochiinae: Cnodalonini.

*Mithippia* Pascoe, 1869: 288, 293 [F]. Type species: *Mithippiaaurita* Pascoe, 1869, by monotypy. Status: valid genus in Tenebrioninae: Heleini: Cyphaleina.

*Mitotagenia* Reitter, 1916d: 138, 153 [F]. Type species: *Stenosisarabs* Baudi di Selve, 1881, by monotypy. Status: valid genus in Pimeliinae: Stenosini: Stenosina.

*Mitragardhus* Koch, 1956a: 379 [M]. Type species: *Tragardhusnodosus* Koch, 1956, by monotypy. Status: valid subgenus of *Tragardhus* Koch, 1956 in Blaptinae: Dendarini: Melambiina. Note: combined description of a new genus-group taxon and a single new species (ICZN 1999, Article 13.4).

*Mitragenius* Solier, 1836: 307, 328 [M]. Type species: *Mitrageniusdejeanii* Solier, 1836, by monotypy. Status: valid genus in Pimeliinae: Nycteliini. Note: the alternative original spelling *Mitrogenius*, used by Solier (1836: 330), was rejected by Solier (1838a: 487) who acted as First Reviser (ICZN 1999, Article 24.2.4).

*Mitrephorus* Carter, 1913a: 83 [M]. Type species: *Mitrephorusconvexicollis* Carter, 1913, by monotypy. Status: senior synonym of *Mitrothorax* Carter, 1914 in Tenebrioninae: Heleini: Cyphaleina. Synonymy: Carter (1914b: 78, with *Ctimene* Bates, 1873, a senior synonym of *Mitrothorax* Carter, 1914). Note: junior homonym of *Mitrephorus* Schönherr, 1837 [Coleoptera: Curculionidae].

*Mitrothorax* Carter, 1914b: 78 [M]. Type species [automatic]: *Mitrephorusconvexicollis* Carter, 1913, by monotypy. Status: valid genus in Tenebrioninae: Heleini: Cyphaleina. Note: replacement name for *Mitrephorus* Carter, 1913. Note: the First Reviser (*Mitrothorax* Carter, 1914 versus *Timeneca* Carter, 1914) is Matthews (1992: 456)

*Mitua* Hope, 1848: 56 [F]. Type species: *Mituabidwelli* Hope, 1848 (= *Opatrumtuberculicostatum* White, 1846), by original designation. Status: senior synonym of *Pseudopatrum* Sharp, 1886 in Lagriinae: Adeliini. Synonymy: Blair (1919a: 531). Note: *Mitua* Hope, 1848 has been used as valid in recent literature despite the fact that it is a junior homonym of *Mitua* Strickland, 1841, an unjustified emendation for *Mitu* Lesson, 1831 [Aves]; Strickland's name has been used as valid since 1899 (e.g., Heinroth 1931: 278) and therefore reversal of precedence cannot be used to conserve the younger name as valid in Tenebrionidae.

*Mitys* Champion, 1885: 97 [M]. Type species: *Mitysinflatus* Champion, 1885, by subsequent designation (Gebien 1943: 402). Status: valid genus in Stenochiinae: Cnodalonini.

*Modicodisema* Pic, 1917b: 3 [F]. Type species: *Disemasubopaca* Pic, 1912, by **present designation**. Status: valid subgenus of *Barsenis* Pascoe, 1887 in Lagriinae: Lagriini: Statirina. Note: originally proposed without included nominal species; based on personal communications with Maurice Pic, Borchmann (1936: 510) was the first author to subsequently and expressly include nominal species in *Modicodisema* (ICZN 1999, Article 67.2.2), by including the species *Disemaserraticornis* Mäklin, 1875 and *Disemasubopaca* Pic, 1912, in association with this name.

*Moeon* Champion, 1886: 251 [N]. Type species: *Moeonisthmicum* Champion, 1886, by subsequent designation (Gebien 1942a: 330). Status: valid genus in Stenochiinae: Cnodalonini. Note: as pointed out by M.A. Alonso-Zarazaga (in Bousquet et al. 2018: 307) the name *Moeon* is the Latinization of the Greek noun *moion* (privy parts) and is neuter.

*Moerodes* Rye, 1879: 62 [M]. Type species [automatic]: *Prophaneswestwoodi* W.J. MacLeay, 1872 (= *Prophanesaculeatus* Westwood, 1849), by monotypy. Status: junior synonym of *Prophanes* Westwood, 1849 in Tenebrioninae: Heleini: Cyphaleina. Note: unjustified emendation of *Maerodes* C.O. Waterhouse, 1877, not in prevailing usage.

*Mogadoria* Escalera, 1905c: 467 [F]. Type species: *Tentyriasubelegans* Fairmaire, 1871, by monotypy. Status: junior synonym of *Eulipus* Wollaston, 1864 in Pimeliinae: Tentyriini. Synonymy: Gebien (1937a: 634).

*Mokalagria* Pic, 1953: 163 [F]. Type species: *Lopholagriaangustata* Pic, 1953, by original designation. Status: valid subgenus of *Lopholagria* Borchmann, 1916 in Lagriinae: Lagriini: Lagriina.

*Molion* Champion, 1886: 142 [M]. Type species: *Penetataurus* Lacordaire, 1859, by subsequent designation (Gebien 1940: 758). Status: valid genus in Phrenapatinae: Penetini.

*Moluris* Latreille, 1802: 169 [F]. Type species: *Pimeliagibba* Fabricius, 1787 (= *Tenebriogibbus* Pallas, 1781), by monotypy. Status: valid genus in Pimeliinae: Sepidiini: Molurina. Note: the original combination of the accepted name of the type species, *Tenebriogibbus* Pallas, 1781, is a junior primary homonym of both *Tenebriogibbus* Linnaeus, 1767 and *Tenebriogibbus* DeGeer, 1778.

*Monatrum* Reichardt, 1936: 81, 208 [N]. Type species: *Opatrumcarinatum* Gebler, 1830, by original designation. Status: junior synonym of *Scleropatrum* Reitter, 1887 in Blaptinae: Opatrini: Opatrina. Synonymy: Löbl and Merkl (2003: 250).

*Mongolesthes* Reitter, 1904: 174 [F]. Type species: *Melanesthesheydeni* Csiki, 1901, by monotypy. Status: valid subgenus of *Melanesthes* Dejean, 1834 in Blaptinae: Opatrini: Opatrina.

*Mongolopterocoma* Skopin, 1974b: 146 [F]. Type species: *Pterocomareitteri* Frivaldszky, 1890, by original designation. Status: valid subgenus of *Pterocoma* Dejean, 1834 in Pimeliinae: Pimeliini.

*Monodius* Koch, 1956a: 180 [M]. Type species: *Selinusconvexipennis* Gebien, 1904, by original designation. Status: valid genus in Blaptinae: Platynotini: Platynotina.

*Monoloba* Solier, 1835a: 235 [F]. Type species: *Lobopodadircaeoides* Solier, 1835, by monotypy. Status: valid subgenus of *Lobopoda* Solier, 1835 in Alleculinae: Alleculini: Alleculina.

*Montagona* G.S. Medvedev, 1998a: 176 [F]. Type species: *Tagonoidesasperula* Fairmaire, 1896, by original designation. Status: valid genus in Blaptinae: Blaptini: Gnaptorinina.

*Montaguea* Kaszab, 1982b: 227 [F]. Type species: *Montagueacaledonica* Kaszab, 1982, by original designation. Status: valid genus in Lagriinae: Adeliini.

*Montanocatomus* Nabozhenko, 2006: 842 [M]. Type species: *Catomusgrandis* G.S. Medvedev, 1978, by original designation. Status: valid subgenus of *Catomus* Allard, 1876 in Tenebrioninae: Helopini: Helopina.

*Montanoodescelis* Egorov, 2004: 592 [F]. Type species: *Platyscelissahlbergi* Reitter, 1900, by original designation. Status: valid subgenus of *Oodescelis* Motschulsky, 1845 in Blaptinae: Platyscelidini.

*Monteithium* Matthews, 1998: 704, 803 [N]. Type species: *Monteithiumascetum* Matthews, 1998, by original designation. Status: valid genus in Lagriinae: Adeliini.

*Montiprosodes* G.S. Medvedev, 2001: 83 [M]. Type species: *Prosodesalaiensis* Kraatz, 1885, by original designation. Status: valid subgenus of *Prosodes* Eschscholtz, 1829 in Blaptinae: Blaptini: Prosodina.

*Mophis* Champion, 1886: 168 [M]. Type species: *Mophismarginicollis* Champion, 1886, by subsequent designation (Gebien 1940: 1061). Status: valid genus in Diaperinae: Diaperini: Adelinina.

*Mophon* Champion, 1886: 247 [M]. Type species: *Mophontinctipennis* Champion, 1886, by monotypy. Status: valid genus in Stenochiinae: Cnodalonini.

*Moragacinella* Español, 1954a: 162 [F]. Type species [automatic]: *Moralesialongepilosa* Kaszab, 1944, by original designation. Status: valid genus in Blaptinae: Opatrini: Ammobiina. Note: replacement name for *Moralesia* Kaszab, 1944.

*Moralesia* Kaszab in Español, 1944: 18, 29 [F]. Type species: *Moralesialongepilosa* Kaszab, 1944, by original designation. Status: senior synonym of *Moragacinella* Español, 1954 in Blaptinae: Opatrini: Ammobiina. Note: junior homonym of *Moralesia* Fowler, 1943 [Pisces].

*Morica* Dejean, 1834: 182 [F]. Type species: *Akisplanata* Fabricius, 1801, by subsequent designation (Hope 1841: 122). Status: valid genus in Pimeliinae: Akidini.

*Morocaulus* Fairmaire, 1899d: 215 [M]. Type species: *Morocaulusremipes* Fairmaire, 1899 (= *Bratynaapicalis* Westwood, 1875), by monotypy. Status: junior synonym of *Bratyna* Westwood, 1875 in Alleculinae: incertae sedis. Synonymy: Borchamann (1938: 120, through synonymy of the type species with the type species of *Bratyna* Westwood, 1875).

*Moromelas* Fairmaire, 1898c: 481 [M]. Type species: *Moromelasfoveipennis* Fairmaire, 1898, by monotypy. Status: valid genus in Stenochiinae: Cnodalonini.

*Morphostenophanes* Pic, 1925b: 7 [M]. Type species: *Morphostenophanesaenescens* Pic, 1925, by monotypy. Status: valid genus in Stenochiinae: Cnodalonini.

*Mrazius* Pic, 1925d: 86 [M]. Type species: *Mraziusnodulosus* Pic, 1925, by monotypy. Status: valid genus in Stenochiinae: Cnodalonini.

*Mucheimira* Novák, 2016a: 188 [F]. Type species: *Isomirastoetzneri* Muche, 1981, by original designation. Status: valid subgenus of *Isomira* Mulsant, 1856 in Alleculinae: Alleculini: Gonoderina.

*Mutiloxicum* Nabozhenko & Ivanov, 2018: 546 [N]. Type species: *Toxicumelvirae* Nabozhenko & Ivanov, 2018, by original designation. Status: valid subgenus of *Toxicum* Latreille, 1802 in Tenebrioninae: Toxicini: Toxicina.

*Myatis* Bates, 1879b: 480 [F]. Type species: *Myatishumeralis* Bates, 1879, by subsequent designation (R. Lucas 1920: 425). Status: valid genus in Blaptinae: Platyscelidini.

*Mycetochara* Guérin-Méneville, 1827a: 346 [F]. Type species [automatic]: *Cistelaflavipes* Fabricius, 1792, by subsequent designation (C.G. Thomson 1859: 118). Status: valid genus and subgenus in Alleculinae: Alleculini: Mycetocharina. Note: replacement name for *Mycetophila* Gyllenhal, 1810; *Mycetochara* was also proposed later in the same year by Berthold (1827: 371), see Bouchard and Bousquet (2020a: 101); nomenclatural stability in this genus is threatened by the discovery that 1) the accepted type species, *Cistelascapularis* Illiger, 1805 (= *Cistelahumeralis* Fabricius, 1787) by subsequent designation by Westwood (1838: 32), is currently placed in the valid subgenusErnocharis C.G. Thomson, 1859 (e.g., Novák 2020i: 436) and 2) there are two other species currently placed in subgenusErnocharis C.G. Thomson, 1859 that were selected as type species of *Mycetochara* before the currently accepted designation in Westwood (1838: 32): *Cistelahumeralis* Fabricius, 1787 was chosen as the type species by Guérin-Méneville (1827: 346) when he proposed the replacement name *Mycetochara*, and *Cistelalinearis* Illiger, 1794 (= *Cistelamaura* Fabricius, 1792) was selected as the type species for *Mycetocharis* Gyllenhal, 1827, another replacement name for *Mycetophila* Gyllenhal, 1810, by Stephens (1832a: 375); of the nominal species originally included in *Mycetophila* Gyllenhal, 1810 that are currently placed in the subgenusMycetochara Guérin-Méneville, 1827, C.G. Thomson (1859: 118) was the first to select *Cistelaflavipes* Fabricius, 1792 as the type species; we recommend that an application be submitted to the International Commission on Zoological Nomenclature to set aside previous type species designations for *Mycetochara* Guérin-Méneville, 1827 and select the type species designation proposed by C.G. Thomson (1859: 118).

*Mycetochares* Latreille, 1829a: 42 [M]. Type species [automatic]: *Cistelaflavipes* Fabricius, 1792, by subsequent designation (C.G. Thomson 1859: 118). Status: junior synonym of *Mycetochara* Guérin-Méneville, 1827 in Alleculinae: Alleculini: Mycetocharina. Note: replacement name for *Mycetophila* Gyllenhal, 1810.

*Mycetocharina* Seidlitz, 1890: 136 [F]. Type species: *Alleculaorientalis* Faust, 1877, by monotypy. Status: valid genus and subgenus in Alleculinae: Alleculini: Alleculina.

*Mycetocharis* Gyllenhal, 1827: 510 [F]. Type species [automatic]: *Cistelaflavipes* Fabricius, 1792, by subsequent designation (C.G. Thomson 1859: 118). Status: junior synonym of *Mycetochara* Guérin-Méneville, 1827 in Alleculinae: Alleculini: Mycetocharina. Note: replacement name for *Mycetophila* Gyllenhal, 1810.

†*Mycetocharoides* Schaufuss, 1889: 269 [M]. Type species: *Mycetocharoidesbaumeisteri* Schaufuss, 1889, by monotypy. Status: valid genus in Alleculinae: Alleculini: Mycetocharina. Note: described from Eocene Baltic amber.

*Mycetocula* Novák, 2015c: 78 [F]. Type species: *Mycetocharinasubcruciata* Pic, 1922, by original designation. Status: valid genus in Alleculinae: Alleculini: Alleculina.

*Mycetophila* Gyllenhal, 1810: 541 [F]. Type species: *Cistelaflavipes* Fabricius, 1792, by subsequent designation (C.G. Thomson 1859: 118). Status: senior synonym of *Mycetochara* Guérin-Méneville, 1827 in Alleculinae: Alleculini: Mycetocharina. Note: junior homonym of *Mycetophila* Meigen, 1803 [Diptera].

*Mychestes* Pascoe, 1870: 96 [M]. Type species: *Mychesteslignarius* Pascoe, 1870, by monotypy. Status: valid genus in Tenebrioninae: Toxicini: Dysantina.

*Mycotrogus* Horn, 1870: 364, 367 [M]. Type species: *Mycotroguspiceus* Horn, 1870, by subsequent designation (R. Lucas 1920: 427). Status: valid genus in Tenebrioninae: Triboliini.

*Myladanesthes* Skopin, 1961b: 202 [F]. Type species: *Myladinafortidens* Reitter, 1915, by original designation. Status: valid subgenus of *Gonocephalum* Solier, 1834 in Blaptinae: Opatrini: Opatrina. Note: the alternative original spelling *Myladansthes*, used by Skopin (1961b: 202), was rejected by Skopin (1967: 205) who acted as the First Reviser (ICZN 1999, Article 24.2.4).

*Myladina* Reitter, 1889a: 706 [F]. Type species: *Myladinaunguiculina* Reitter, 1889, by original designation. Status: valid genus in Blaptinae: Opatrini: Opatrina.

*Myladion* Reitter, 1887a: 386 [N]. Type species: *Myladionacuticolle* Reitter, 1887, by monotypy. Status: valid subgenus of *Penthicus* Faldermann, 1836 in Blaptinae: Opatrini: Opatrina. Note: the alternative original spelling *Miladion*, used by Reitter (1887a: 385), was rejected by Reitter (1896b: 166) who acted as the First Reviser (ICZN 1999, Article 24.2.4).

*Mylaris* Pallas, 1781: 37 [F]. Type species: *Tenebriogigas* Linnaeus, 1763, by subsequent designation (Guérin-Méneville 1844: 120). Status: valid genus in Stenochiinae: Cnodalonini. Note: see Bousquet et al. (2018: 307, footnote 82) regarding a possible nomenclatural problem with this genus name.

*Myonophloeus* Bremer & Lillig, 2017a: 68 [M]. Type species: *Corticeustuberculatus* Triplehorn, 1979, by original designation. Status: valid genus in Diaperinae: Hypophlaeini.

*Myrmechixenus* Chevrolat, 1835: 267 [M]. Type species: *Myrmechixenussubterraneus* Chevrolat, 1835, by monotypy. Status: valid genus in Diaperinae: Myrmechixenini.

*Myrmechoxenus* Gaubil, 1849: 288 [M]. Type species [automatic]: *Myrmechixenussubterraneus* Chevrolat, 1835, by monotypy. Status: junior synonym of *Myrmechixenus* Chevrolat, 1835 in Diaperinae: Myrmechixenini. Note: unjustified emendation of *Myrmechixenus* Chevrolat, 1835, not in prevailing usage; while both *Myrmechixenus* (p. 71) and *Myrmechoxenus* (p. 266) were used by Gaubil (1849), the fact that *Myrmechixenus* was written in italics in the Index (p. 282) indicates that the original spelling was treated as a synonym.

*Myrmecocatops* Wasmann, 1897: 268 [M]. Type species: *Myrmecocatopslatus* Wasmann, 1897, by monotypy. Status: valid genus in Diaperinae: Crypticini.

*Myrmecodema* Gebien, 1943: 402 [F]. Type species [automatic]: *Myrmecosomanycterinoides* Germain, 1855, by monotypy. Status: valid genus in Tenebrioninae: Trachelostenini. Note: replacement name for *Myrmecosoma* Germain, 1855. Note: transferred from Stenochiinae: Cnodalonini by Matthews and Lawrence (2015: 293).

*Myrmecodichillus* Kaszab, 1960a: 6, 7, 14 [M]. Type species: *Dichillusreichenspergeri* Kaszab, 1960, by original designation. Status: valid subgenus of *Dichillus* Jacquelin du Val, 1860 in Pimeliinae: Stenosini: Dichillina.

*Myrmecopeltoides* Kaszab, 1973b: 318 [M]. Type species: *Myrmecopeltoidescamponoti* Kaszab, 1973, by original designation. Status: valid genus in Lagriinae: Goniaderini.

*Myrmecophosis* Koch, 1958: 72 [F]. Type species: *Zophosispedinoides* Gebien, 1920, by original designation. Status: valid subgenus of *Zophosis* Latreille, 1802 in Pimeliinae: Zophosini.

*Myrmecosoma* Germain, 1855: 403 [N]. Type species: *Myrmecosomanycterinoides* Germain, 1855, by monotypy. Status: senior synonym of *Myrmecodema* Gebien, 1943 in Tenebrioninae: Trachelostenini. Note: junior homonym of *Myrmecosoma* Mannerheim, 1846 [Coleoptera: Anthicidae].

*Myrmecoxenus* Märkel, 1844: 253 [M]. Type species [automatic]: *Myrmechixenussubterraneus* Chevrolat, 1835, by monotypy. Status: junior synonym of *Myrmechixenus* Chevrolat, 1835 in Diaperinae: Myrmechixenini. Note: unjustified emendation of *Myrmechixenus* Chevrolat, 1835, not in prevailing usage.

*Nalassus* Mulsant, 1854: 323 [M]. Type species: *Helopsdryadophilus* Mulsant, 1854, by subsequent designation (Nabozhenko 2001a: 630). Status: valid genus and subgenus in Tenebrioninae: Helopini: Cylindrinotina.

*Nalepa* Reitter, 1887a: 366 [F]. Type species: *Blapscylindracea* Reitter, 1887, by monotypy. Status: valid genus in Blaptinae: Blaptini: Blaptina.

*Namaphosis* Penrith, 1981c: 127, 143 [F]. Type species: *Zophosissolivaga* Koch, 1958, by original designation. Status: valid subgenus of *Zophosis* Latreille, 1802 in Pimeliinae: Zophosini.

*Namaquaeon* Koch, 1950b: 305, 341 [N]. Type species: *Phaeotribonaustralis* Péringuey, 1908, by original designation. Status: valid genus in Pimeliinae: Tentyriini.

*Namazopus* Koch, 1963: 34 [M]. Type species: *Namazopusarachnipes* Koch, 1963, by original designation. Status: valid genus in Blaptinae: Opatrini: Stizopodina.

*Namibismus* Koch, 1952a: 107 [M]. Type species: *Namibismuscastaneus* Koch, 1952, by original designation. Status: valid genus in Pimeliinae: Tentyriini.

*Namibomodes* Koch, 1952d: 223 [M]. Type species: *Psammodesserrimargo* Gebien, 1938, by original designation. Status: valid genus in Pimeliinae: Sepidiini: Oxurina.

*Nannalcyon* Koch, 1950a: 68 [F]. Type species [automatic]: *Nannoceruscylindrus* Fairmaire, 1887, by monotypy. Status: valid genus in Stenochiinae: Cnodalonini. Note: replacement name for *Nannocerus* Fairmaire, 1887.

*Nannocerus* Fairmaire, 1887b: 292 [M]. Type species: *Nannoceruscylindrus* Fairmaire, 1887, by monotypy. Status: senior synonym of *Nannalcyon* Koch, 1950 in Stenochiinae: Cnodalonini. Note: junior homonym of *Nannocerus* Mayr, 1885 [Hymenoptera].

*Nannohyocis* G.S. Medvedev & Lawrence, 1983: 569 [M]. Type species: *Hyocisinquilinus* Carter, 1921, by original designation. Status: valid subgenus of *Hyocis* Pascoe, 1866 in Diaperinae: Hyociini: Hyociina.

*Nanoblaps* Semenov-Tjan-Shansky & Bogatchev, 1936: 565 [F]. Type species: *Blaps jakovlevi* Semenov-Tjan-Shansky & Bogatchev, 1936, by monotypy. Status: junior synonym of *Blaps* Fabricius, 1775 in Blaptinae: Blaptini: Blaptina. Synonymy: Löbl et al. (2008c: 219).

*Nanocaecus* Schawaller & Purchart, 2012: 305, 310 [M]. Type species: *Nanocaecushlavaci* Schawaller & Purchart, 2012, by original designation. Status: valid genus in Diaperinae: Gnathidiini: Anopidiina.

*Nanocalcar* Skopin, 1974a: 67, 102 [N]. Type species: *Nanocalcarnanum* Skopin, 1974, by monotypy. Status: valid subgenus of *Centorus* Mulsant, 1854 in Lagriinae: Belopini.

*Nanohemicera* Pic, 1923c: 25 [F]. Type species: *Hemicerarufomaculata* Pic, 1923, by monotypy. Status: valid subgenus of *Hemicera* Laporte & Brullé, 1831 in Stenochiinae: Cnodalonini.

*Nanotagalus* Gebien, 1942b: 118, 120 [M]. Type species: *Afrotagalususambaricus* Gebien, 1942, by monotypy. Status: valid genus in Phrenapatinae: Penetini.

*Narses* Champion, 1888: 423 [M]. Type species: *Narsessubalatus* Champion, 1888, by monotypy. Status: junior synonym of *Charisius* Champion, 1888 in Alleculinae: Alleculini: Alleculina. Synonymy: Campbell (2014: 271).

*Narsodes* Campbell, 1976: 32 [M]. Type species: *Narsodesbrachypterus* Campbell, 1976, by original designation. Status: valid genus in Alleculinae: Alleculini: Alleculina.

*Nataloplonyx* Ardoin, 1963c: 716 [M]. Type species: *Hoplonyxmicans* Schaufuss, 1870, by original designation. Status: valid subgenus of *Hoplonyx* J. Thomson, 1858 in Tenebrioninae: Amarygmini.

*Natalostira* Pic, 1913c: 143 [F]. Type species: *Natalostirabrevithorax* Pic, 1913, by monotypy. Status: valid genus in Lagriinae: Lagriini: Statirina.

*Nautes* Pascoe, 1866a: 475 [M]. Type species: *Nautesfervidus* Pascoe, 1866, by monotypy. Status: valid genus in Tenebrioninae: Helopini: Helopina.

*Neacisba* Peyerimhoff, 1927: 53 [F]. Type species [automatic]: *Pachychiladissecta* Kraatz, 1865, by monotypy. Status: junior synonym of *Neocisba* Reitter, 1900 in Pimeliinae: Tentyriini. Note: unjustified emendation of *Neocisba* Reitter, 1900, not in prevailing usage.

*Neandrosus* Pic, 1921b: 12 [M]. Type species: *Neandrosussingularipes* Pic, 1921, by monotypy. Status: junior synonym of *Derosphaerus* J. Thomson, 1858 in Stenochiinae: Cnodalonini. Synonymy: Kaszab (1987: 44). Note: we act as First Revisers and reject the alternative original spelling *Neoandrosus*, used by Pic (1921b: 12).

*Neanopidium* Dajoz, 1975a: 93 [N]. Type species: *Neanopidiummexicanum* Dajoz, 1975, by original designation. Status: valid genus in Diaperinae: Gnathidiini: Anopidiina.

*Neatus* J.L. LeConte, 1862: 233 [M]. Type species: *Helopstenebrioides* Palisot de Beauvois, 1812, by monotypy. Status: valid genus in Tenebrioninae: Tenebrionini.

*Necrobioides* Fairmaire, 1882a: 234 [M]. Type species: *Necrobioidescoeruleatus* Fairmaire, 1882, by monotypy. Status: valid genus in Stenochiinae: Cnodalonini.

*Neglectophloeus* Bremer & Lillig, 2017a: 71 [M]. Type species: *Hypophlaeusluteomaculatus* Pic, 1914, by original designation. Status: valid subgenus of *Corticeus* Piller & Mitterpacher, 1783 in Diaperinae: Hypophlaeini.

*Nelites* J.L. LeConte, 1850: 232 [M]. Type species: *Nelitesaeneolus* J.L. LeConte, 1850, by monotypy. Status: junior synonym of *Scaphidema* Redtenbacher, 1848 in Diaperinae: Scaphidemini. Synonymy: J.L. LeConte (1862: 237).

*Nemanes* Fairmaire, 1888a: 195 [M]. Type species: *Nemanesexpansicollis* Fairmaire, 1888, by monotypy. Status: valid genus in Blaptinae: Opatrini: Stizopodina.

*Nemapus* Solier, 1835b: 313 [M]. Type species: *Microderacoromandelensis* Solier, 1835, by original designation. Status: valid subgenus of *Rhytinota* Eschscholtz, 1831 in Pimeliinae: Tentyriini. Note: the name *Nemapus* was listed as synonym of *Microdera* Eschscholtz, 1831 by Solier (1835b: 313); it was treated before 1961 as an available name and adopted as the name of a taxon (e.g., Koch 1943b: 860); therefore, *Nemapus* was made available from its first publication as a synonym (ICZN 1999, Article 11.6.1).

*Nemoplonyx* Ardoin, 1963c: 715, 734 [M]. Type species: *Hoplonyxgallanus* Gridelli, 1939, by original designation. Status: valid subgenus of *Hoplonyx* J. Thomson, 1858 in Tenebrioninae: Amarygmini.

*Nemostira* Fairmaire, 1869a: 815 [F]. Type species: *Nemostiracoquerelii* Fairmaire, 1869, by subsequent designation (R. Lucas 1920: 434). Status: junior synonym of *Sora* Walker, 1859 in Lagriinae: Lagriini: Statirina. Synonymy: Borchmann (1929a: 5).

*Nemostiromorpha* Pic, 1917b: 2,3 [F]. Type species: *Disemalongicornis* Mäklin, 1875, by subsequent monotypy (Borchmann 1936: 510). Status: valid subgenus of *Barsenis* Pascoe, 1887 in Lagriinae: Lagriini: Statirina. Note: originally proposed without included nominal species; based on personal communications with Maurice Pic, Borchmann (1936: 510) was the first author to subsequently and expressly include nominal species in *Nemostiromorpha* (ICZN 1999, Article 67.2.2), by including the species *Disemalongicornis* Mäklin, 1875, in association with this name.

*Nemostiropsis* Borchmann, 1936: 341 [F]. Type species: *Sorapurpureipennis* Borchmann, 1930, by original designation. Status: valid subgenus of *Sora* Walker, 1859 in Lagriinae: Lagriini: Statirina.

*Neoabantis* Gebien, 1910b: 341 [F]. Type species [automatic]: *Abantisaenescens* Fairmaire, 1892, by monotypy. Status: junior synonym of *Diphyrrhynchus* Fairmaire, 1849 in Blaptinae: Opatrini: Heterotarsina. Synonymy: Gebien (1938a: 407). Note: replacement name for *Abantiades* Fairmaire, 1894.

*Neoadelium* Carter, 1908a: 259 [N]. Type species: *Adeliumfairmairei* Bates, 1873, by subsequent designation (Gebien 1942a: 742). Status: valid genus in Lagriinae: Adeliini.

*Neoatractus* Borchmann, 1909a: 713 [M]. Type species [automatic]: *Atractusviridis* Boisduval, 1835, by subsequent designation (Duponchel and Chevrolat 1841: 312). Status: junior synonym of *Lepturidea* Fauvel, 1862 in Alleculinae: Alleculini: Alleculina. Synonymy: Matthews and Bouchard (2008: 324). Note: unnecessary replacement name for *Atractus* Boisduval, 1835.

*Neobaphion* Blaisdell, 1925: 390 [N]. Type species: *Eleodesplanipennis* J.L. LeConte, 1866, by monotypy. Status: valid genus in Blaptinae: Amphidorini.

*Neoblaps* Ren & Li, 2001: 310 [F]. Type species: *Neoblapshuizensis* Ren & Li, 2001, by monotypy. Status: junior synonym of *Coelocnemodes* Bates, 1879 in Blaptinae: Blaptini: Blaptina. Synonymy: Ren et al. (2016: 432).

*Neocabirutus* Kulzer, 1964: 221 [M]. Type species: *Neocabirutusindicus* Kulzer, 1964, by monotypy. Status: valid subgenus of *Cabirutus* Strand, 1929 in Blaptinae: Pedinini: Pedinina.

*Neocaedius* Pierre, 1972: 960, 967 [M]. Type species: *Caediushalli* Kaszab, 1949, by original designation. Status: junior synonym of *Cyptus* Gerstaecker, 1871 in Blaptinae: Opatrini: Ammobiina. Synonymy: Ferrer (1996: 254, 2003: 297).

*Neocamaria* Kulzer, 1954a: 52 [F]. Type species: *Neocamariatibialis* Kulzer, 1954, by original designation. Status: junior synonym of *Robustocamaria* Pic, 1922 in Stenochiinae: Cnodalonini. Synonymy: Masumoto (1993b: 222).

*Neocisba* Reitter, 1900c: 154 [F]. Type species: *Pachychiladissecta* Kraatz, 1865, by monotypy. Status: valid subgenus of *Pachychila* Eschscholtz, 1831 in Pimeliinae: Tentyriini.

*Neocistela* Borchmann, 1909a: 713 [F]. Type species [automatic]: *Pseudocistelaovalis* Blackburn, 1891, by monotypy. Status: valid genus in Alleculinae: Alleculini: Alleculina. Note: replacement name for *Pseudocistela* Blackburn, 1891.

*Neodissonomus* G.S. Medvedev, 1968a: 235 [M]. Type species: *Heterophylusangustitarsis* Reitter, 1896, by original designation. Status: valid subgenus of *Dissonomus* Jacquelin du Val, 1861 in Tenebrioninae: Dissonomini.

*Neoeutrapela* Bousquet & Bouchard, 2013a: 61 [F]. Type species [automatic]: *Crioceriselongata* Fabricius, 1781 (= *Chrysomelaunifasciata* DeGeer, 1778), by subsequent designation (Duponchel 1844b: 533). Status: valid subgenus (**stat. nov.** [OM]) of *Impressosora* Pic, 1952 in Lagriinae: Lagriini: Statirina. Note: replacement name for *Eutrapela* Dejean, 1834; **new placement** [OM], previously included in Lagriinae: Lagriini: Lagriina.

*Neognathosia* Kaszab, 1959a: 383 [F]. Type species: *Gnathosiapseudanemia* Reitter, 1915, by original designation. Status: valid genus in Pimeliinae: Tentyriini.

*Neogria* Borchmann, 1911: 222 [F]. Type species: *Neogriasulcipennis* Borchmann, 1911, by subsequent designation (R. Lucas 1920: 436). Status: valid genus in Lagriinae: Lagriini: Lagriina.

*Neohelops* Dajoz, 2001: 356 [M]. Type species: *Neohelopstexanus* Dajoz, 2001, by original designation. Status: valid genus in Tenebrioninae: Helopini: Helopina. Note: placed in the subtribe Helopina by Nabozhenko and Purchart (2019: 156).

*Neohyocis* G.S. Medvedev & Lawrence, 1983: 571 [M]. Type species: *Hyociswaterhousei* G.S. Medvedev & Lawrence, 1983, by original designation. Status: valid subgenus of *Hyocis* Pascoe, 1866 in Diaperinae: Hyociini: Hyociina.

*Neoisocerus* Bouchard, Lawrence, Davies & Newton, 2005: 510 [M]. Type species [automatic]: *Tenebriopurpurascens* Herbst, 1799 (= *Helopsferrugineus* Fabricius, 1798), by monotypy. Status: valid genus in Blaptinae: Dendarini: Dendarina. Note: replacement name for *Isocerus* Dejean, 1821.

*Neomenimus* Kaszab, 1939b: 190 [M]. Type species: *Neomenimusclavatus* Kaszab, 1939, by original designation. Status: junior synonym of *Menimus* Sharp, 1876 in Diaperinae: Gnathidiini: Gnathidiina. Synonymy: G.S. Medvedev (2007b: 665).

*Neomida* Latreille, 1829a: 29 [F]. Type species: *Ips haemorrhoidalis* Fabricius, 1787, by monotypy. Status: valid genus in Diaperinae: Diaperini: Diaperina. Note: *Neomida* was used earlier by Dahl (1823: 44) but Dahl’s work was suppressed for the purposes of zoological nomenclature by the ICZN (1964, Opinion 710).

*Neooligocara* Guerrero, Vidal & Moore, 2007: 407 [N]. Type species: *Oligocarabucki* Kulzer, 1962, by original designation. Status: valid genus in Tenebrioninae: Ulomini.

*Neopachypterus* Bouchard, Löbl & Merkl, 2007: 386 [M]. Type species [automatic]: *Pachypterusmauritanicus* P.H. Lucas, 1847, by monotypy. Status: valid genus in Blaptinae: Opatrini: Neopachypterina. Note: replacement name for *Pachypterus* P.H. Lucas, 1847.

*Neophaleria* Español, 1963a: 74, 76 [F]. Type species: *Phaleriaardoini* Español, 1963, by monotypy. Status: valid subgenus of *Phaleria* Latreille, 1802 in Diaperinae: Phaleriini.

*Neophylax* Bedel, 1906a: 92 [M]. Type species [automatic]: *Phylaxlittoralis* Mulsant, 1854 (= *Opatrumpicipes* G.-A. Olivier, 1812), by subsequent designation (Gebien 1938a: 412). Status: senior synonym of *Allophylax* Bedel, 1906 in Blaptinae: Dendarini: Melambiina. Note: junior homonym of *Neophylax* McLachlan, 1871 [Trichoptera].

*Neoplamius* Löbl, Bouchard, Merkl & Bousquet, 2020: 4 [M]. Type species: *Neoplamiuszoltani* Masumoto, 1981, by original designation. Status: valid genus in Stenochiinae: Cnodalonini. Note: name first proposed by Masumoto (1981: 15) without fixation of a type species in the original publication (ICZN 1999, Article 13.3).

*Neoplateia* Marcuzzi, 1986: 177 [F]. Type species: *Neoplateiakaszabi* Marcuzzi, 1986, by monotypy. Status: valid genus in Diaperinae: Diaperini: Adelinina.

*Neoplonyx* Ardoin, 1963b: 309, 352 [M]. Type species: *Gonocnemissulcicollis* Fairmaire, 1899, by original designation. Status: valid genus in Tenebrioninae: Amarygmini.

*Neoporphyrhyba* Ardoin, 1956b: 89 [F]. Type species: *Porphyrhybacyaneocuprea* Fairmaire, 1894, by original designation. Status: valid genus in Stenochiinae: Cnodalonini.

*Neopraocis* Kulzer, 1958a: 3, 6 [M]. Type species: *Praocisreflexicollis* Solier, 1851, by monotypy. Status: valid genus in Pimeliinae: Praociini.

*Neopsectropus* Kaszab, 1941c: 30 [M]. Type species: *Neopsectropusgebieni* Kaszab, 1941, by original designation. Status: valid genus in Tenebrioninae: Ulomini.

*Neopterocoma* Skopin, 1974b: 158 [F]. Type species: *Pterocomabalchashensis* Skopin, 1974, by original designation. Status: valid subgenus of *Pterocoma* Dejean, 1834 in Pimeliinae: Pimeliini.

*Neosolenopistoma* Bouchard & Bousquet, **new subgenus**. [N]. Type species: *Eurynotusdenticosta* Mulsant & Rey, 1854, by **present designation**. Status: valid subgenus of *Eurynotus* W. Kirby, 1819 in Blaptinae: Platynotini: Eurynotina. Note: taxon first proposed by Mulsant and Rey (1854: 29; as “*Solenopistoma* Solier”); however, this is treated as an incorrect subsequent spelling of *Selenepistoma* Dejean, 1834 (see Bousquet and Bouchard 2013: 50); the subgenusSolenopistoma Mulsant & Rey, 1854, which is currently used as valid, is therefore unavailable (ICZN 1999, Articles 33.3); we hereby make the name *Neosolenopistoma* available by selecting *Eurynotusdenticosta* Mulsant & Rey, 1854 as type species and referring to Mulsant and Rey (1854: 29) for the character states that characterise and differentiate *Neosolenopistoma*.

*Neotagalus* Kaszab, 1955a: 471, 477 [M]. Type species: *Neotagalustuberculiger* Kaszab, 1955, by original designation. Status: valid genus in Phrenapatinae: Penetini.

*Neotheca* Carter, 1930: 540 [F]. Type species: *Neothecafusca* Carter, 1930, by original designation. Status: valid genus in Stenochiinae: Cnodalonini.

*Neozophobas* Ferrer, 2006b: 235 [M]. Type species: *Zophobaslaticollis* Kraatz, 1880, by original designation. Status: valid genus in Tenebrioninae: Tenebrionini.

*Nepalindia* G.S. Medvedev, 1998a: 187 [F]. Type species: *Tagonoidesalpina* Kaszab, 1965, by original designation. Status: valid genus in Blaptinae: Blaptini: Gnaptorinina.

*Nepalofranziella* Fouquè, 2013: 194, 196 [F]. Type species: *Nepalofranziellakaszabi* Fouquè, 2013, by original designation. Status: valid genus in Pimeliinae: Stenosini: Dichillina.

*Nepalolaena* Schawaller, 2001a: 277 [F]. Type species: *Nepalolaenakira* Schawaller, 2001, by original designation. Status: valid genus in Lagriinae: Laenini.

*Nepaloplonyx* Bremer, 2014b: 175, 181 [M]. Type species: *Nepaloplonyxcaelebs* Bremer, 2014, by original designation. Status: valid genus in Tenebrioninae: Amarygmini.

*Nephodes* Blanchard, 1845: 34 [M]. Type species: *Nephodesvilliger* Rosenhauer, 1856, by subsequent monotypy (Rosenhauer 1856: 218). Status: senior synonym of *Nephodinus* Gebien, 1943 in Tenebrioninae: Helopini: Helopina. Note: originally proposed without included nominal species; Rosenhauer (1856: 218), by including the new species *Nephodesvilliger* Rosenhauer, 1856 in association with this name, was the first author to subsequently and expressly include nominal species in *Nephodes* (ICZN 1999, Article 67.2.2); the older name *Nephodes* Schönherr, 1840 [Coleoptera: Curculionidae] is available as it was originally proposed as a synonym and subsequently treated as a senior homonym by Gebien (1943: 900; see ICZN 1999, Article 11.6.1), therefore *Nephodes* Blanchard, 1845 is a junior homonym of *Nephodes* Schönherr, 1840.

*Nephodinus* Gebien, 1943: 900 [M]. Type species [automatic]: *Nephodesvilliger* Rosenhauer, 1856, by subsequent monotypy (Rosenhauer 1856: 218). Status: valid genus and subgenus in Tenebrioninae: Helopini: Helopina. Note: replacement name for *Nephodes* Blanchard, 1845.

*Nerina* Lacordaire, 1859a: 70 [F]. Type species: *Nerinadispar* Lacordaire, 1859, by monotypy. Status: senior synonym of *Afrinus* Fairmaire, 1888 in Pimeliinae: Tentyriini. Synonymy: Blair (1935a: 103). Note: junior homonym of *Nerina* Robineau-Desvoidy, 1830 [Diptera].

*Nerinodon* Koch, 1952a: 138 [M]. Type species: *Nerinodoncaviceps* Koch, 1952, by monotypy. Status: valid genus in Pimeliinae: Tentyriini.

*Nesioticus* Westwood, 1843: 120 [M]. Type species: *Nesioticusflavopictus* Westwood, 1843, by monotypy. Status: valid genus in Tenebrioninae: Amarygmini. Note: redescribed as new by Westwood (1844: 227).

*Nesocaedius* Kolbe, 1915: 262 [M]. Type species: *Nesocaediusschultzei* Kolbe, 1915, by monotypy. Status: valid genus in Blaptinae: Opatrini: Ammobiina.

*Nesocyrtosoma* Marcuzzi, 1976: 137 [N]. Type species: *Cyrtosomainflatum* Marcuzzi, 1976, by plenary powers (ICZN 2017, Opinion 2398). Status: valid genus in Stenochiinae: Cnodalonini. Note: following an application by Hopp et al. (2014) to conserve the generic name *Nesocyrtosoma* Marcuzzi, 1976, the ICZN (2017, Opinion 2398) determined that this name is nomenclaturally available despite not having been accompanied by a type species fixation in the original publication, placed it on the Official List of Generic Names in Zoology and, designated *Cyrtosomainflatum* Marcuzzi, 1976 as its type species.

*Nesogena* Mäklin, 1863b: 557 [F]. Type species: *Nesogenahybrida* Mäklin, 1863, by subsequent designation (Gebien 1948: 549). Status: valid genus and subgenus in Tenebrioninae: Praeugenini.

*Nesogenomorpha* Pic, 1917c: 18 [F]. Type species: *Nesogenomorphasemiviolacea* Pic, 1917, by monotypy. Status: valid genus in Alleculinae: Alleculini: Alleculina.

*Nesopatrum* Gebien, 1921b: 20 [N]. Type species: *Opatrinusjosephi* Karsch, 1881, by original designation. Status: valid genus in Blaptinae: Platynotini: Platynotina.

*Nesophaerotus* Ardoin, 1962a: 66, 67 [M]. Type species [automatic]: *Nesosphaerotusaeneus* Gebien, 1921, by subsequent designation (Gebien 1943: 403). Status: junior synonym of *Nesosphaerotus* Gebien, 1921 in Stenochiinae: Cnodalonini. Note: unjustified emendation of *Nesosphaerotus* Gebien, 1921, not in prevailing usage.

*Nesosphaerotus* Gebien, 1921b: 115 [M]. Type species: *Nesosphaerotusaeneus* Gebien, 1921, by subsequent designation (Gebien 1943: 403). Status: valid genus in Stenochiinae: Cnodalonini.

*Nesostes* Casey, 1908: 56, 58 [M]. Type species: *Eusattusrobustus* J.L. LeConte, 1866, by original designation. Status: junior synonym of *Eusattus* J.L. LeConte, 1851 in Pimeliinae: Coniontini. Synonymy: Triplehorn (1968: 379).

*Nesotaurus* Fairmaire, 1896b: 354 [M]. Type species: *Nesotaurussericans* Fairmaire, 1896, by monotypy. Status: valid genus in Alleculinae: Cteniopodini.

*Nesotes* Allard, 1876a: 5 [M]. Type species: *Helopsasper* Küster, 1850, by subsequent designation (Nabozhenko 2008: 38). Status: valid genus and subgenus in Tenebrioninae: Helopini: Helopina. Note: the First Reviser (*Nesotes* Allard, 1876 versus *Diastixus* Allard, 1876) is Antoine (1949: 134).

*Netopha* Fairmaire, 1893c: 299 [F]. Type species: *Netophapallidipes* Fairmaire, 1893, by monotypy. Status: valid genus in Alleculinae: Alleculini: Alleculina.

*Netuschilia* Reitter, 1904: 34, 35 [F]. Type species: *Lachnopushauseri* Reitter, 1897, by monotypy. Status: valid genus in Pimeliinae: Lachnogyini: Netuschiliina.

*Nevermanniella* Borchmann, 1936: 235, 332 [F]. Type species: *Statiraalbolineata* Champion, 1889, by original designation. Status: valid genus in Lagriinae: Lagriini: Statirina. Note: the alternative original spelling *Nevermannia* (pp. 11, 235) was corrected to *Nevermanniella* in the “Corrigenda” of the same work (p. 541), *Nevermanniella* is considered to be the correct original spelling (ICZN 1999, Article 32.5.1.1)

*Nevisia* Marcuzzi, 1986: 179 [F]. Type species: *Diastolinusbardudensis* Marcuzzi, 1962, by monotypy. Status: valid genus in Blaptinae: Opatrini: Blapstinina.

*Nicandra* Fairmaire, 1888a: 191 [F]. Type species: *Nicandracostulipennis* Fairmaire, 1888, by monotypy. Status: valid genus and subgenus in Blaptinae: Pedinini: Helopinina.

*Nikomenalia* Dubrovina, 1975: 166 [F]. Type species: *Hymenaliakaszabi* Muche, 1972, by original designation. Status: valid genus in Alleculinae: Alleculini: Alleculina. Note: elevated from subgenus of *Hymenalia* Mulsant, 1856 by Novák (2020c: 507).

*Nilio* Latreille, 1802: 179 [M]. Type species: *Coccinellavillosa* Fabricius, 1787, by monotypy. Status: valid genus and subgenus in Nilioninae. Note: *Nilio* is an incorrect subsequent spelling of the original spelling *Nilion*, first used by Latreille (1804: 333), in prevailing usage; *Nilio* is deemed to be the correct original spelling (ICZN 1999, Article 33.3.1); the original combination of the name of the type species, *Coccinellavillosa* Fabricius, 1787, is a junior primary homonym of *Coccinellavillosa* Fourcroy, 1785.

*Nipponalassus* Nabozhenko & Ando, 2018: 306 [M]. Type species: *Tarpelaandoi* Masumoto, 1993, by original designation. Status: valid subgenus of *Nalassus* Mulsant, 1854 in Tenebrioninae: Helopini: Cylindrinotina.

*Nipponohelops* Masumoto, Ando & Akita, 2006: 33 [M]. Type species: *Nipponohelopsishikawai* Masumoto, Ando & Akita, 2006, by original designation. Status: valid genus in Tenebrioninae: Helopini: Helopina.

*Nocar* Blackburn, 1891: 328 [N]. Type species: *Cisteladepressiuscula* W.J. MacLeay, 1872, by subsequent designation (R. Lucas 1920: 441). Status: valid genus in Alleculinae: Alleculini: Alleculina.

*Nochelius* Gistel, 1848a: xi [M]. Type species [automatic]: *Evaniosomusorbignianus* Guérin-Méneville, 1834, by monotypy. Status: junior synonym of *Evaniosomus* Guérin-Méneville, 1834 in Pimeliinae: Evaniosomini. Note: unnecessary replacement name for *Evaniosomus* Guérin-Méneville, 1834, not in prevailing usage.

*Nocibiotes* Casey, 1895: 617 [M]. Type species: *Notibiusgranulatus* J.L. LeConte, 1851, by subsequent designation (Gebien 1938a: 407). Status: valid genus in Blaptinae: Opatrini: Blapstinina.

*Nodosogylium* Pic, 1951: 12 [N]. Type species: *Nodosogyliuminaequale* Pic, 1951, by monotypy. Status: valid genus in Stenochiinae: Stenochiini. Note: we act as First Revisers and reject the alternative original spelling *Nodosogilium*, used by Pic (1951: 12).

*Nodotelus* Koch, 1950a: 67 [M]. Type species [automatic]: *Eutelusrequieni* Solier, 1843, by subsequent designation (R. Lucas 1920: 291). Status: junior synonym of *Eutelonotus* Fairmaire in Alluaud, 1902 in Stenochiinae: Cnodalonini. Note: unnecessary replacement name for *Eutelus* Solier, 1843.

*Nolicima* Matthews, 1998: 708, 714 [F]. Type species: *Cardiothoraxangusticollis* Carter, 1906, by original designation. Status: valid genus in Lagriinae: Adeliini.

*Notacula* Campbell, 1971: 107 [F]. Type species: *Notaculahowdenae* Campbell, 1971, by original designation. Status: valid genus in Alleculinae: Alleculini: Alleculina.

*Nothogria* Borchmann, 1916a: 49, 142 [F]. Type species: *Nothogrianodipennis* Borchmann, 1916, by monotypy. Status: valid genus in Lagriinae: Lagriini: Lagriina.

*Nothrocerus* Fairmaire, 1887a: 174 [M]. Type species: *Nothroceruscylindricornis* Fairmaire, 1887, by monotypy. Status: valid genus in Pimeliinae: Tentyriini.

*Notiasida* Casey, 1912: 76, 124 [F]. Type species: *Notiasidaabstrusa* Casey, 1912, by original designation. Status: valid subgenus of *Stenomorpha* Solier, 1836 in Pimeliinae: Asidini.

*Notibius* J.L. LeConte, 1851: 144 [M]. Type species: *Notibiuspuberulus* J.L. LeConte, 1851, by subsequent designation (Gebien 1938a: 406). Status: valid genus in Blaptinae: Opatrini: Blapstinina.

*Notiolesthus* Motschulsky, 1872: 25 [M]. Type species: *Notiolesthusnatalensis* Motschulsky, 1872, by original designation. Status: junior synonym of *Derosphaerus* J. Thomson, 1858 in Stenochiinae: Cnodalonini. Synonymy: Gebien (1921b: 69). Note: we act as First Revisers and reject the alternative original spelling *Notiolosthus*, used by Motschulsky (1872: 34).

*Notioscythis* Fairmaire, 1883a: 31 [F]. Type species: *Notioscythispunctoseriata* Fairmaire, 1883, by monotypy. Status: junior synonym of *Stenosida* Solier, 1835 in Pimeliinae: Tentyriini. Synonymy: Chatanay (1917: 231).

*Notoblaps* Bauer, 1921: 232 [F]. Type species: *Blaps juliae* Allard, 1881, by monotypy. Status: junior synonym of *Blaps* Fabricius, 1775 in Blaptinae: Blaptini: Blaptina. Synonymy: Nabozhenko and Chigray (2020: 10).

*Notocistela* Carter, 1915a: 78, 102 [F]. Type species: *Notocistelatibialis* Carter, 1915, by subsequent designation (Matthews and Bouchard 2008: 333). Status: valid genus in Alleculinae: Alleculini: Alleculina.

*Notocorax* Dejean, 1834: 191 [M]. Type species: *Opatrumjavanum* Wiedemann, 1819, by monotypy. Status: valid genus in Blaptinae: Platynotini: Platynotina.

*Notoprataeus* Carter, 1924a: 37 [M]. Type species: *Notoprataeuslitoralis* Carter, 1924 (= *Mesotertisinconstans* Lea, 1917), by monotypy. Status: junior synonym of *Micropedinus* Lewis, 1894 in Lagriinae: Lupropini. Synonymy: G.S. Medvedev (1992: 656), Matthews and Lawrence (2005: 534).

*Notostrongylium* Carter, 1915b: 523 [N]. Type species: *Notostrongyliumrugosicolle* Carter, 1915, by subsequent designation (Kulzer 1966: 387). Status: junior synonym of *Strongylium* W. Kirby, 1819 in Stenochiinae: Stenochiini. Synonymy: Kaszab (1977b: 3, 29).

*Nototrintus* Carter, 1924a: 40 [M]. Type species: *Otrintusjacksoni* Carter, 1905, by original designation. Status: valid genus in Lagriinae: Adeliini.

*Nudoplatyscelis* Kaszab, 1940a: 149, 222 [F]. Type species: *Platynoscelisturanica* Reitter, 1896, by original designation. Status: valid subgenus of *Bioramix* Bates, 1879 in Blaptinae: Platyscelidini.

*Nuptis* Motschulsky, 1872: 25 [M]. Type species: *Nuptistenuis* Motschulsky, 1872, by original designation. Status: valid genus in Stenochiinae: Cnodalonini.

*Nyctalops* Gistel, 1848a: 125 [M]. Type species [automatic]: *Pseudoblapssubstriata* Guérin-Méneville, 1834, by subsequent designation (Hope 1841: 124). Status: junior synonym of *Pseudoblaps* Guérin-Méneville, 1834 in Blaptinae: Platynotini: Platynotina. Note: unnecessary replacement name for *Pseudoblaps* Guérin-Méneville, 1834; junior homonym of *Nyctalops* Wagler, 1832 [Aves].

*Nyctelia* Berthold, 1827: 367 [F]. Type species: *Zophosisnodosa* Germar, 1823 (= *Zophosispicipes* Billberg, 1815), by monotypy. Status: valid genus in Pimeliinae: Nycteliini. Note: nomen protectum (see Silvestro and Flores 2016: 658).

*Nyctelioma* Casey, 1908: 163 [F]. Type species: *Nycteliomaexplanata* Casey, 1908, by original designation. Status: junior synonym of *Nyctelia* Berthold, 1827 in Pimeliinae: Nycteliini. Synonymy: Gebien (1910a: 141).

*Nyctelius* Guérin-Méneville, 1827b: 21 [M]. Type species: *Zophosisnodosa* Germar, 1823 (= *Zophosispicipes* Billberg, 1815), by monotypy. Status: senior synonym of *Nyctelia* Berthold, 1827 in Pimeliinae: Nycteliini. Synonymy: Silvestro and Flores (2016: 658). Note: nomen oblitum (see Silvestro and Flores 2016: 658).

*Nycterinus* Eschscholtz, 1829: 9.13 [M]. Type species: *Nycterinusthoracicus* Eschscholtz, 1829, by subsequent designation (Hope 1841: 124). Status: valid genus in Blaptinae: Amphidorini.

*Nycteropus* Klug, 1833: 89 [M]. Type species: *Nycteropusebeninus* Klug, 1833, by subsequent designation (Hope 1841: 124). Status: valid genus in Tenebrioninae: Toxicini: Nycteropina.

*Nyctipates* Gebler, 1841: 373 [M]. Type species: *Nyctipatesrugulosus* Gebler, 1841, by monotypy. Status: junior synonym of *Prosodes* Eschscholtz, 1829 in Blaptinae: Blaptini: Prosodina. Synonymy: Erichson (1845a: 116).

*Nyctipates* Solier, 1848: 154, 285 [M]. Type species: *Nyctipatescoriaceus* Solier, 1848 (= *Blapsangustata* Zubkov, 1833), by subsequent designation (Löbl et al. 2008c: 236). Status: senior synonym of *Prosodopria* Reitter, 1909 in Blaptinae: Blaptini: Prosodina. Synonymy: Löbl et al. (2008a: 43). Note: junior homonym of *Nyctipates* Gebler, 1841 [Coleoptera: Tenebrionidae: Blaptinae: Blaptini: Prosodina].

*Nyctobates* Guérin-Méneville, 1834: 33 [M]. Type species: *Tenebriogigas* Linnaeus, 1763, by original designation. Status: junior synonym of *Mylaris* Pallas, 1781 in Stenochiinae: Cnodalonini. Synonymy: Spilman (1973: 42), Ferrer and Siliansky (2008: 186).

*Nyctopetus* Guérin-Méneville, 1831a: pl. 4 [M]. Type species: *Nyctopetustenebrioides* Guérin-Méneville, 1831, by monotypy. Status: valid genus in Pimeliinae: Epitragini.

*Nyctoporis* Eschscholtz, 1831: 10, 11 [F]. Type species: *Nyctoporiscristata* Eschscholtz, 1831, by subsequent designation (Hope 1841: 124). Status: valid genus in Pimeliinae: Nyctoporini.

*Nyctozoilus* Guérin-Méneville, 1831a: pl. 4 [M]. Type species: *Nyctozoilusobesus* Guérin-Méneville, 1831, by monotypy. Status: valid genus in Tenebrioninae: Heleini: Cyphaleina.

*Nypsius* Champion, 1895a: 219 [M]. Type species: *Nypsiusaeneopiceus* Champion, 1895, by subsequent designation (R. Lucas 1920: 447). Status: valid genus in Alleculinae: Alleculini: Alleculina.

*Oatesius* Westwood, 1889: 376 [M]. Type species [automatic]: *Derosphaeriusanthracinus* Westwood, 1881, by monotypy. Status: junior synonym of *Derosphaerius* Westwood, 1881 in Pimeliinae: Tentyriini. Note: unnecessary replacement name for *Derosphaerius* Westwood, 1881.

*Obenbergeria* Strand, 1929: 24 [F]. Type species [automatic]: *Diaperisriederii* Faldermann, 1833, by subsequent designation (Jakobson 1924: 243). Status: junior synonym of *Emypsara* Pascoe, 1866 in Diaperinae: Phaleriini. Synonymy: Gebien (1939: 743). Note: replacement name for *Callicomus* Motschulsky, 1860.

*Obesacula* Campbell, 1971: 109 [F]. Type species: *Obesaculaaptera* Campbell, 1971, by original designation. Status: valid genus in Alleculinae: Alleculini: Alleculina.

*Oblongoodescelis* Kaszab, 1940b: 941, 958 [F]. Type species: *Platyscelisoblonga* Ballion, 1878, by original designation. Status: junior synonym of *Clavatoodescelis* Kaszab, 1940 in Blaptinae: Platyscelidini. Synonymy: Egorov (2020: 380).

*Oblongoplatyscelis* Kaszab, 1940b: 910, 916 [F]. Type species: *Platyscelisganglbaueri* Seidlitz, 1893, by original designation. Status: valid subgenus of *Platyscelis* Latreille, 1818 in Blaptinae: Platyscelidini.

*Obriomaia* Gebien, 1927: 45 [F]. Type species: *Eucyrtussubcostatus* Fairmaire, 1893, by original designation. Status: junior synonym of *Tetragonomenes* Chevrolat, 1878 in Stenochiinae: Cnodalonini. Synonymy: Kaszab (1983a: 133).

*Occidentophosis* Penrith, 1977: 18, 157 [F]. Type species: *Zophosisdamarina* Péringuey, 1908, by original designation. Status: valid subgenus of *Zophosis* Latreille, 1802 in Pimeliinae: Zophosini.

*Ochrolamus* Reitter, 1904: 73 [M]. Type species: *Dilamuspictus* Baudi di Selve, 1881, by monotypy. Status: valid subgenus of *Dilamus* Jacquelin du Val, 1861 in Blaptinae: Opatrini: Ammobiina.

*Ocnera* Fischer, 1822: 169 [F]. Type species: *Pimeliaimbricata* Fischer, 1820, by subsequent designation (Wilke 1922: 260). Status: valid genus in Pimeliinae: Pimeliini. Note: as mentioned by Bouchard and Bousquet (2020b: 7) the nomenclatural stability of this name is threatened by the discovery of an older type species designation (*Tenebriocephalotes* Pallas, 1781, by subsequent designation by Crotch (1870b: 241), currently the type species of the valid subgenusChaetotoma Motschulsky, 1860 in *Pimelia* Fabricius, 1775); we recommend that an application be submitted to the International Commission on Zoological Nomenclature to maintain the type species designation proposed by Gebien (1937a: 814).

*Ocnodes* Fåhraeus, 1870: 270 [F]. Type species: *Ocnodesscrobicollis* Fåhraeus, 1870, by subsequent designation (Kamiński et al. 2019b: 55). Status: valid genus and subgenus in Pimeliinae: Sepidiini: Molurina.

*Ocularisora* Pic, 1934a: 32 [F]. Type species: *Nemostirabenitensis* Pic, 1912, by monotypy. Status: valid genus in Lagriinae: Lagriini: Statirina.

*Oculochara* Novák, 2020e: 80 [F]. Type species: *Mycetocharaocularis* Reitter, 1884, by original designation. Status: valid subgenus of *Mycetochara* Guérin-Méneville, 1827 in Alleculinae: Alleculini: Mycetocharina.

*Oculosis* Penrith, 1977: 18, 126 [F]. Type species: *Zophosisboei* Solier, 1834, by original designation. Status: valid subgenus of *Zophosis* Latreille, 1802 in Pimeliinae: Zophosini.

*Odocnemis* Allard, 1876a: 4 [F]. Type species: *Odocnemiscaudata* Allard, 1876 (= *Helopspraelongus* Baudi di Selve, 1876), by subsequent designation (Nabozhenko 2001a: 662). Status: valid genus and subgenus in Tenebrioninae: Helopini: Cylindrinotina. Note: the First Reviser (*Odocnemis* Allard, 1876 versus *Omaleis* Allard, 1876) is Nabozhenko (2001a: 628).

*Odontocera* Chen & Yuan, 1996: 183 [F]. Type species: *Odontoceraqinlingensis* Chen & Yuan, 1996, by original designation. Status: senior synonym of *Odontocerostira* Merkl, 2007 in Lagriinae: Lagriini: Statirina. Note: junior homonym of *Odontocera* Audinet-Serville, 1834 [Coleoptera: Cerambycidae].

*Odontocerostira* Merkl, 2007: 269 [F]. Type species [automatic]: *Odontoceraqinlingensis* Chen & Yuan, 1996, by original designation. Status: valid genus in Lagriinae: Lagriini: Statirina. Note: replacement name for *Odontocera* Chen & Yuan, 1996.

*Odontocnemis* Rye, 1878: 69 [F]. Type species [automatic]: *Odocnemiscaudata* Allard, 1876 (= *Helopspraelongus* Baudi di Selve, 1876), by subsequent designation (Nabozhenko 2001a: 662). Status: junior synonym of *Odocnemis* Allard, 1876 in Tenebrioninae: Helopini: Cylindrinotina. Note: unjustified emendation of *Odocnemis* Allard, 1876, not in prevailing usage.

*Odontogria* Borchmann, 1936: 16, 62 [F]. Type species: *Lagriablairi* Borchmann, 1925, by original designation. Status: valid genus in Lagriinae: Lagriini: Lagriina.

*Odontomophlus* Seidlitz, 1896: 240 [M]. Type species: *Omophlusochraceipennis* Faldermann, 1837, by subsequent designation (Bousquet et al. 2015: 141). Status: valid subgenus of *Omophlus* Dejean, 1834 in Alleculinae: Cteniopodini. Note: as pointed out by Bousquet et al. (2015: 141) the type species of this genus was first designated by Novák and Pettersson (2008: 335) who selected *Cistelaarmillata* Brullé, 1832, a species originally included in *Odontomophlus* Seidlitz, 1896 but also the type species of the valid genus *Megischina* Reitter, 1906; Löbl and Smetana (2011: 34) “retracted” the act proposed earlier by Novák and Pettersson (2008: 335); in order to conserve nomenclatural stability Bousquet et al. (2015: 141) recommended usage of *Omophlusochraceipennis* Faldermann, 1837 as the type species of this genus until an application be submitted to the Commission to rule on the Case.

*Odontopezus* Alluaud, 1889: xlv [M]. Type species [automatic]: *Odontopuscostatus* Silbermann, 1833, by subsequent designation (Hope 1841: 126). Status: junior synonym of *Pezodontus* Dejean, 1834 in Lagriinae: Pycnocerini. Note: unnecessary replacement name for *Odontopus* Silbermann, 1833.

*Odontopus* Silbermann, 1833: no 3 [M]. Type species: *Odontopuscostatus* Silbermann, 1833, by subsequent designation (Hope 1841: 126). Status: senior synonym of *Pezodontus* Dejean, 1834 in Lagriinae: Pycnocerini. Note: junior homonym of *Odontopus* Say, 1831 [Coleoptera: Curculionidae].

*Odrotes* La Rivers, 1947: 320 [M]. Type species: *Edrotesarens* La Rivers, 1947, by monotypy. Status: valid subgenus of *Edrotes* J.L. LeConte, 1851 in Pimeliinae: Edrotini.

*Oeatus* Champion, 1885: 111 [M]. Type species: *Oeatuschevrolati* Champion, 1885, by subsequent designation (Gebien 1941: 342). Status: valid genus in Stenochiinae: Cnodalonini.

*Oectosis* Pascoe, 1869: 149 [F]. Type species: *Upiscylindrica* Germar, 1848, by monotypy. Status: valid genus in Stenochiinae: Cnodalonini.

*Oedemutes* Pascoe, 1860a: 51 [M]. Type species: *Oedemutestumidus* Pascoe, 1860, by monotypy. Status: valid genus and subgenus in Stenochiinae: Cnodalonini.

*Oedenocera* Reiche, 1862: 372 [F]. Type species [automatic]: **fixed herein** (ICZN 1999, Article 70.3) as *Tenebriobuprestoides* Fabricius, 1781, misidentified as *Akislaevigata* Fabricius, 1801 in the original designation by monotypy in Eschscholtz (1831). Status: junior synonym of *Hyperops* Eschscholtz, 1831 in Pimeliinae: Tentyriini. Synonymy: Löbl et al. (2008b: 192). Note: replacement name for *Pachycera* Eschscholtz, 1831. Note: see additional information in the entry for *Pachycera* Eschscholtz, 1831.

*Oenomia* Pascoe, 1883: 441 [F]. Type species: *Oenomiafemorata* Pascoe, 1883, by monotypy. Status: valid genus in Stenochiinae: Stenochiini.

*Oenopion* Champion, 1885: 98 [M]. Type species: *Oenopiongibbosus* Champion, 1885, by monotypy. Status: valid genus in Stenochiinae: Cnodalonini.

*Ogoueum* Pic, 1923c: 27 [N]. Type species: *Ogoueumsemirufum* Pic, 1923, by monotypy. Status: junior synonym of *Eccoptostoma* Gebien, 1913 in Stenochiinae: Cnodalonini. Synonymy: Ardoin (1962a: 64).

*Ograbies* Péringuey, 1899: 293 [M]. Type species: *Ograbiessingularis* Péringuey, 1899 (= *Oncotustestaceus* Solier, 1848), by monotypy. Status: valid genus in Blaptinae: Platynotini: Eurynotina.

*Ohyonthis* Reitter, 1898: 347 [F]. Type species: *Ohyonthismicroderoides* Reitter, 1898, by monotypy. Status: junior synonym of *Stegastopsis* Kraatz, 1865 in Pimeliinae: Tentyriini. Synonymy: Reitter (1900c: 89, 139).

*Oligocara* Solier, 1848: 153, 224 [N]. Type species: *Oligocaranitidum* Solier, 1848, by original designation. Status: valid genus in Tenebrioninae: Ulomini.

*Oligorus* Dejean, 1834: 206 [M]. Type species: *Tageniaindica* Wiedemann, 1823, by monotypy. Status: junior synonym of *Luprops* Hope, 1833 in Lagriinae: Lupropini. Synonymy: Lacordaire (1859a: 397, as “*Lyprops*”).

*Oliprosodes* Reitter, 1909a: 118 [M]. Type species: *Prosodestrisulcata* Bates, 1879, by subsequent designation (G.S. Medvedev 1999a: 851). Status: valid subgenus of *Prosodes* Eschscholtz, 1829 in Blaptinae: Blaptini: Prosodina.

*Olisthaena* Erichson, 1842a: 177 [F]. Type species: *Olisthaenanitida* Erichson, 1842, by monotypy. Status: valid genus in Tenebrioninae: Heleini: Cyphaleina.

*Olocrates* Mulsant, 1854: 150, 383 [M]. Type species: *Opatrumgibbum* Fabricius, 1775, by subsequent designation (C.G. Thomson 1859: 115, as “*Omocrates*”). Status: junior synonym of *Phylan* Sturm, 1826 in Blaptinae: Dendarini: Dendarina. Synonymy: Seidlitz (1898a: 828). Note: the original spelling *Omocrates* (p. 150) was corrected to *Olocrates* in the “Errata” of the same work (p. 383), *Olocrates* is considered to be the correct original spelling (ICZN 1999, Article 32.5.1.1); see Bouchard and Bousquet (2020a: 100).

*Ologlyptus* Lacordaire [in LeConte], 1858: 19 [M]. Type species [automatic]: *Stenosidesgraciliformis* Solier, 1836, by monotypy. Status: junior synonym of *Stenosides* Solier, 1836 in Pimeliinae: Asidini. Note: unnecessary replacement name for *Stenosides* Solier, 1836 (as *Stenorides*).

*Omala* Agassiz, 1846b: 184 [F]. Type species [automatic]: *Homalapolita* Eschscholtz, 1831, by monotypy. Status: junior synonym of *Homala* Eschscholtz, 1831 in Pimeliinae: Tentyriini. Note: unjustified emendation of *Homala* Eschscholtz, 1831, not in prevailing usage.

*Omaleis* Allard, 1876a: 4 [M]. Type species: *Helopscongener* Reiche, 1861, by subsequent designation (Nabozhenko 2008: 38). Status: junior synonym of *Odocnemis* Allard, 1876 in Tenebrioninae: Helopini: Cylindrinotina. Synonymy: Nabozhenko (2001a: 628). Note: Allard’s original spelling *Omalus*, which is a junior homonym of *Omalus* Panzer, 1801 [Hymenoptera], was corrected to “*Omaloïs*” [= *Omalois*] in the errata for volume 14 of *L’Abeille*, *Journal d’Entomologie* (at the end of page 36 of the section “Table alphabétique”) and therefore *Omalois* should be considered the correct original spelling (ICZN 1999, Article 32.5.1.1); however, the incorrect subsequent spelling *Omaleis*, which was introduced by Allard (1877: 36), is in prevailing usage and attributed to the original author, it is deemed to be the correct original spelling (ICZN 1999, Article 33.3.1).

*Omandelostoma* Purchart, 2017: 282 [N]. Type species: *Adelostomamuqalensis* Purchart, 2017, by original designation. Status: valid subgenus of *Adelostoma* Duponchel, 1827 in Pimeliinae: Adelostomini.

*Omedes* Broun, 1893b: 1169 [M]. Type species: *Omedesnitidus* Broun, 1893, by subsequent designation (R. Lucas 1920: 456). Status: valid genus in Alleculinae: Alleculini: incertae sedis.

*Omegeleodes* Triplehorn & Thomas, 2012: 253 [M]. Type species: *Eleodesdebilis* J.L. LeConte, 1858, by original designation. Status: valid subgenus of *Eleodes* Eschscholtz, 1829 in Blaptinae: Amphidorini.

*Ommatochara* Borchmann, 1932a: 347 [F]. Type species: *Ommatocharatibialis* Borchmann, 1932, by original designation. Status: valid genus in Alleculinae: Alleculini: Alleculina.

*Ommatophorus* W.J. MacLeay, 1872: 304 [M]. Type species: *Ommatophorusmastersii* W.J. MacLeay, 1872, by monotypy. Status: valid genus in Alleculinae: Alleculini: Alleculina.

*Omocula* Borchmann, 1937: 222 [F]. Type species: *Alleculacollaris* Borchmann, 1930, by original designation. Status: valid genus in Alleculinae: Alleculini: Alleculina.

*Omolepta* Fåhraeus, 1870: 320 [F]. Type species: *Omoleptaelegans* Fåhraeus, 1870, by monotypy. Status: valid genus in Alleculinae: incertae sedis.

*Omolipus* Pascoe, 1860b: 127 [M]. Type species: *Omolipuscorvus* Pascoe, 1860, by monotypy. Status: valid genus in Stenochiinae: Cnodalonini.

*Omopheres* Casey, 1907: 519 [M]. Type species: *Omopheresfarctus* Casey, 1907, by original designation. Status: valid genus and subgenus in Pimeliinae: Epitragini.

*Omophlina* Reitter, 1890a: 34 [F]. Type species: *Omophluspodontoides* Reitter, 1890, by subsequent designation (Novák and Pettersson 2008: 334). Status: valid genus in Alleculinae: Cteniopodini.

*Omophlus* Dejean, 1834: 213 [M]. Type species: *Cistelalepturoides* Fabricius, 1787, by subsequent designation (Solier 1835a: 246). Status: valid genus and subgenus in Alleculinae: Cteniopodini. Note: the earlier usage of the name *Omophlus* by Dahl (1823: 46) was suppressed for the purposes of zoological nomenclature by the ICZN (1964, Opinion 710).

*Oncopterus* Fairmaire, 1887a: 178 [M]. Type species: *Oncopterusacantholophus* Fairmaire, 1887, by monotypy. Status: senior synonym of *Oncopteryx* Gebien, 1943 in Blaptinae: Pedinini: Helopinina. Note: junior homonym of *Oncopterus* Steindachner, 1875 [Pisces].

*Oncopteryx* Gebien, 1943: 905 [F]. Type species [automatic]: *Oncopterusacantholophus* Fairmaire, 1887, by monotypy. Status: valid genus in Blaptinae: Pedinini: Helopinina. Note: replacement name for *Oncopterus* Fairmaire, 1887.

*Oncosoma* Westwood, 1843: 121 [N]. Type species: *Oncosomagranulare* Westwood, 1843 (= *Pimeliagemmata* Fabricius, 1801), by monotypy. Status: junior synonym of *Amatodes* Dejean, 1834 in Blaptinae: Pedinini: Helopinina. Synonymy: Erichson (1844: 282). Note: unjustified emendation of the original spelling *Ogcosoma*, introduced by Agassiz (1846b: 257, 259), in prevailing usage and treated as a justified emendation (ICZN 1999, Article 33.2.3.1), see Bouchard et al. (2005: 509); redescribed by Westwood (1844: 227) under the name *Ogcoosoma*; the original combination of the accepted name of the type species, *Pimeliagemmata* Fabricius, 1801, is a junior primary homonym of *Pimeliagemmata* Herbst, 1799.

*Oncoosoma* Gebien, 1911a: 563 [N]. Type species [automatic]: *Oncosomagranulare* Westwood, 1843 (= *Pimeliagemmata* Fabricius, 1801), by monotypy. Status: junior synonym of *Amatodes* Dejean, 1834 in Blaptinae: Pedinini: Helopinina. Note: unjustified emendation of *Oncosoma* Westwood, 1843 (as “*Ogcosoma*”), not in prevailing usage.

*Oncotiphallops* Koch, 1956a: 162 [M]. Type species: *Oncotiphallopsbarbosai* Koch, 1956, by original designation. Status: junior synonym of *Anchophthalmus* Gerstaecker, 1854 in Blaptinae: Platynotini: Platynotina. Synonymy: Iwan (2002a: 53).

*Oncotopsis* Koch, 1958: 152 [F]. Type species: *Nicandrabicolor* Kulzer, 1951, by original designation. Status: valid subgenus of *Nicandra* Fairmaire, 1888 in Blaptinae: Pedinini: Helopinina.

*Oncotus* Blanchard, 1845: 13, 24 [M]. Type species: *Oncotusfarctus* Solier, 1848, by subsequent designation (Gebien 1938a: 72). Status: valid genus and subgenus in Blaptinae: Platynotini: Eurynotina. Note: originally proposed without included nominal species; Solier (1848: 216–222), by including five new species in association with the genus “*Oncotus* Dejean”, was the first author to subsequently and expressly include nominal species in *Oncotus* (ICZN 1999, Article 67.2.2).

*Onocera* Borchmann, 1936: 139 [F]. Type species: *Ecnolagriasecurigera* Borchmann, 1916, by original designation. Status: valid subgenus of *Ecnolagria* Borchmann, 1916 in Lagriinae: Lagriini: Lagriina.

*Onoglypta* Carter, 1926: 144 [F]. Type species [automatic]: *Aglyptaoctocostata* Gebien, 1908, by monotypy. Status: junior synonym of *Aglypta* Gebien, 1908 in Tenebrioninae: Heleini: Cyphaleina. Note: unnecessary replacement name for *Aglypta* Gebien, 1908.

*Ononyctus* Carter, 1914c: 382 [M]. Type species: *Ononyctussulcatus* Carter, 1914, by monotypy. Status: junior synonym of *Nyctozoilus* Guérin-Méneville, 1831 in Tenebrioninae: Heleini: Cyphaleina. Synonymy: Matthews (1992: 473).

*Onosterrhus* Pascoe, 1866a: 451 [M]. Type species: *Onosterrhuslaevis* Pascoe, 1866, by monotypy. Status: junior synonym of *Nyctozoilus* Guérin-Méneville, 1831 in Tenebrioninae: Heleini: Cyphaleina. Synonymy: Matthews (1992: 473).

*Onotrichus* Carter, 1911b: 138, 164 [M]. Type species: *Onotrichuslateralis* Carter, 1911, by monotypy. Status: valid genus in Tenebrioninae: Heleini: Cyphaleina.

*Onychomira* Campbell, 1984: 289 [F]. Type species: *Onychomirafloridensis* Campbell, 1984, by original designation. Status: valid genus in Alleculinae: Alleculini: Gonoderina.

*Onychosis* Deyrolle, 1867: 226 [F]. Type species: *Onychosisgracilipes* Deyrolle, 1867, by monotypy. Status: valid subgenus of *Zophosis* Latreille, 1802 in Pimeliinae: Zophosini. Note: the name *Anisosis*, also described in the same publication, was used in error for *Onychosis* in the “Tableau des genres” on p. 81 (see Dallas 1868: 265–266).

*Onymacris* Allard, 1885: 157 [F]. Type species: *Adesmiacandidipennis* Brême, 1840, by subsequent designation (Gebien 1937a: 655). Status: valid genus in Pimeliinae: Adesmiini.

*Oochila* J.L. LeConte, 1862: 220 [F]. Type species: *Asbolusinfaustus* J.L. LeConte, 1854, by original designation. Status: junior synonym of *Cryptoglossa* Solier, 1837 in Pimeliinae: Cryptoglossini. Synonymy: Horn (1870: 278, with *Centrioptera* Mannerheim, 1843, a junior synonym of *Cryptoglossa* Solier, 1837).

*Oochrotus* P.H. Lucas, 1852: xxix [M]. Type species: *Oochrotusunicolor* P.H. Lucas, 1852, by monotypy. Status: valid genus in Diaperinae: Crypticini.

*Oocistela* Borchmann, 1908: 356 [F]. Type species: *Oocistelaconvexa* Borchmann, 1908, by monotypy. Status: valid genus in Alleculinae: Alleculini: Alleculina.

*Ooconibius* Casey, 1895: 618 [M]. Type species: *Notibiusopacus* J.L. LeConte, 1866, by monotypy. Status: junior synonym of *Conibius* J.L. LeConte, 1851 in Blaptinae: Opatrini: Blapstinina. Synonymy: Aalbu in Bousquet et al. (2018: 202).

*Oodeoscelis* Agassiz, 1846b: 260 [F]. Type species [automatic]: *Blapspolita* Sturm, 1807, by subsequent designation (Kaszab 1940b: 937; see ICZN 1993b, Opinion 1729). Status: junior synonym of *Oodescelis* Motschulsky, 1845 in Blaptinae: Platyscelidini. Note: unjustified emendation of *Oodescelis* Motschulsky, 1845, not in prevailing usage.

*Oodescelis* Motschulsky, 1845a: 76 [F]. Type species: *Blapspolita* Sturm, 1807, by subsequent designation (Kaszab 1940b: 937; see ICZN 1993b, Opinion 1729). Status: valid genus and subgenus in Blaptinae: Platyscelidini. Note: placed on the Official List of Generic Names in Zoology (ICZN 1993b, Opinion 1729).

*Oogaster* Faldermann, 1837: 30 [F]. Type species: *Oogastermenetriesii* Faldermann, 1837 (= *Tageniapicea* Ménétriés, 1832), by monotypy. Status: valid genus in Pimeliinae: Stenosini: Dichillina.

*Oogeton* Kaszab, 1941b: 69 [M]. Type species: *Oogetonnigrocoeruleus* Kaszab, 1941 (= *Amarygmusmakii* Miwa, 1939), by original designation. Status: valid subgenus of *Amarygmus* Dalman, 1823 in Tenebrioninae: Amarygmini. Note: name first used by Miwa (1939: 412) without type species fixation (see Masumoto 1989a: 116; Löbl et al. 2008a: 44).

*Oopiestus* Chevrolat, 1833b: 30, pl. 2 [M]. Type species: *Oopiestusovalis* Chevrolat, 1833 (= *Peltoidessenegalensis* Laporte, 1833), by monotypy. Status: senior synonym of *Peltoides* Laporte, 1833 in Tenebrioninae: Alphitobiini. Synonymy: Audoin and Milne-Edward (1835: 188). Note: the alternative original spelling *Opiestus*, used by Chevrolat (1833b: 30), was rejected by Chevrolat (1847a: 118) who acted as the First Reviser (ICZN 1999, Article 24.2.4); bibliographic evidence indicates that *Oopiestus* Chevrolat, 1833 was published by 16 March 1833 before *Peltoides* Laporte, 1833, issued by 1 April 1833, and should be treated as the valid name instead; an application to the ICZN is necessary to conserve usage of *Peltoides* Laporte, 1833 as the valid name.

*Opacoplonyx* Bremer, 2014a: 35 [M]. Type species: *Plesiophthalmusdavidis* Fairmaire, 1878, by original designation. Status: valid subgenus of *Plesiophthalmus* Motschulsky, 1857 in Tenebrioninae: Amarygmini.

*Opatrasida* Escalera, 1922b: 69 [F]. Type species: *Asidajurinei* Solier, 1836, by subsequent designation (F. Soldati 2008: 33). Status: junior synonym of *Polasida* Reitter, 1917 in Pimeliinae: Asidini. Synonymy: Gebien (1937a: 727).

*Opatresthes* Gebien, 1928: 192 [F]. Type species: *Opatresthesbinodosa* Gebien, 1928, by subsequent designation (Gebien 1941: 817). Status: valid subgenus of *Goniadera* Perty, 1832 in Lagriinae: Goniaderini.

*Opatrinus* Dejean, 1821: 66 [M]. Type species: *Opatrumclathratum* Fabricius, 1787, by monotypy. Status: valid genus and subgenus in Blaptinae: Platynotini: Platynotina.

*Opatroides* Brullé, 1832: 219 [M]. Type species: *Opatroidespunctulatus* Brullé, 1832, by monotypy. Status: valid genus in Blaptinae: Opatrini: Opatrina.

*Opatronesthes* Reitter, 1904: 174 [F]. Type species: *Melanesthespunctipennis* Reitter, 1889, by subsequent designation (Iwan and Löbl 2007: 734). Status: valid subgenus of *Melanesthes* Dejean, 1834 in Blaptinae: Opatrini: Opatrina.

*Opatropis* Reitter, 1904: 134, 159 [F]. Type species: *Opatrumhispidum* Brullé, 1839 (= *Opatrumaffine* Billberg, 1815), by monotypy. Status: valid subgenus of *Gonocephalum* Solier, 1834 in Blaptinae: Opatrini: Opatrina.

*Opatrum* Fabricius, 1775: 76 [N]. Type species: *Silphasabulosa* Linnaeus, 1758, by subsequent designation (Latreille 1810: 429). Status: valid genus and subgenus in Blaptinae: Opatrini: Opatrina.

*Ophthalmosis* Deyrolle, 1867: 81, 229 [F]. Type species: *Ophthalmosislongipes* Deyrolle, 1867, by monotypy. Status: valid subgenus of *Zophosis* Latreille, 1802 in Pimeliinae: Zophosini. Note: the original spelling *Ophtalmosis* (pp. 77, 229, 231) was corrected to *Ophthalmosis* in a footnote in the “Table des matières” of the same work (p. clxiv), *Ophthalmosis* is considered to be the correct original spelling (ICZN 1999, Article 32.5.1.1).

*Opigenia* Pascoe, 1869: 288 [F]. Type species: *Opigeniairidescens* Pascoe, 1869 (= *Platyphanesvittatus* Westwood, 1849), by monotypy. Status: junior synonym of *Platyphanes* Westwood, 1849 in Tenebrioninae: Heleini: Cyphaleina. Synonymy: Matthews (1992: 454).

*Opisthoblaps* Kolbe, 1928: 201 [F]. Type species: *Blaps sulcifera* Seidlitz, 1893, by subsequent designation (Nabozhenko and Chigray 2020: 10). Status: junior synonym of *Blaps* Fabricius, 1775 in Blaptinae: Blaptini: Blaptina. Synonymy: Nabozhenko and Chigray (2020: 10).

*Oplocephala* Laporte & Brullé, 1831: 332, 338 [F]. Type species: *Ips haemorrhoidalis* Fabricius, 1787, by subsequent designation (Motschulsky 1845a: 80). Status: junior synonym of *Neomida* Latreille, 1829 in Diaperinae: Diaperini: Diaperina. Synonymy: Dejean (1834: 197).

*Oplocheirus* Klug, 1835: 40 [M]. Type species: *Helopscarbonarius* Klug, 1835 (= *Acanthomerusstriatus* Guérin-Méneville, 1834), by monotypy. Status: valid genus in Tenebrioninae: Amarygmini.

*Oploptera* Chevrolat in Guérin-Méneville, 1844: 126 [F]. Type species: *Strongyliumserraticorne* Guérin-Méneville, 1834, by monotypy. Status: valid genus and subgenus in Stenochiinae: Stenochiini.

*Opostirus* Kirsch, 1865: 45 [M]. Type species: *Opostirusexsectus* Kirsch, 1865, by monotypy. Status: valid genus in Tenebrioninae: Toxicini: Dysantina. Note: transferred from Zopheridae: Colydiinae by Ivie et al. (2016: 759).

*Oppenheimeria* Koch, 1952a: 110 [F]. Type species: *Oppenheimeriabombophthalma* Koch, 1952, by original designation. Status: valid genus in Pimeliinae: Evaniosomini.

*Oracula* Novák, 2019f: 54 [F]. Type species: *Oraculabicolor* Novák, 2019, by original designation. Status: valid genus and subgenus in Alleculinae: Alleculini: Alleculina.

*Orarabion* Leo & Liberto, 2011: 157 [N]. Type species: *Orarabiondominici* Leo & Liberto, 2011, by original designation. Status: valid genus in Blaptinae: Dendarini: Melambiina.

*Orchesiolobopoda* Pic, 1919: 6 [F]. Type species: *Orchesiolobopodaminutissima* Pic, 1919, by monotypy. Status: valid genus in Alleculinae: Alleculini: Alleculina.

*Orcopagia* Pascoe, 1868: xii [F]. Type species: *Orcopagiamonstrosa* Pascoe, 1868, by monotypy. Status: valid genus in Tenebrioninae: Toxicini: Dysantina.

*Oremasis* Pascoe, 1866a: 470 [M]. Type species: *Adeliumcupreum* Gray, 1831, by original designation. Status: junior synonym of *Cyphaleus* Westwood, 1841 in Tenebrioninae: Heleini: Cyphaleina. Synonymy: Matthews (1992: 490).

*Oreogria* Merkl, 1988b: 248 [F]. Type species: *Oreogriakaszabi* Merkl, 1988, by original designation. Status: valid genus in Lagriinae: Lagriini: Lagriina.

*Oreomelasma* Español, 1975: 238, 240 [N]. Type species: *Oreomelasmaoromii* Español, 1975, by original designation. Status: valid genus in Blaptinae: Dendarini: Melambiina.

*Orghidania* Ardoin, 1977: 383 [F]. Type species: *Orghidaniatorrei* Ardoin, 1977, by monotypy. Status: senior synonym of *Spelaebiosis* Bousquet & Bouchard, 2018 in Tenebrioninae: Triboliini. Note: junior homonym of *Orghidania* Capuse, 1971 [Lepidoptera].

*Orientocara* Koch, 1952a: 176 [N]. Type species: *Stenocaraarachnoides* Gerstaecker, 1854, by original designation. Status: valid genus in Pimeliinae: Adesmiini.

*Orientochile* Penrith & Endrödy-Younga, 1994: 82 [F]. Type species: *Cryptochileelegans* Gerstaecker, 1854, by original designation. Status: valid genus in Pimeliinae: Cryptochilini: Cryptochilina.

*Orobychus* Pascoe, 1868: xii [M]. Type species: *Orobychuslacordairii* Pascoe, 1868 (= *Taphrosomadohrnii* Kirsch, 1866), by monotypy. Status: junior synonym of *Taphrosoma* Kirsch, 1866 in Stenochiinae: Cnodalonini. Synonymy: Champion (1885: 108).

*Orocina* Reitter, 1897a: 303 [F]. Type species: *Orocinacapnisiceps* Reitter, 1897, by subsequent designation (R. Lucas 1920: 463). Status: junior synonym of *Syachis* Bates, 1879 in Pimeliinae: Tentyriini. Synonymy: Bogdanov-Katjkov (1916: 68).

*Orophylaxus* Koch, 1948: 417 [M]. Type species: *Phylaxincertus* Mulsant & Godart, 1866, by original designation. Status: valid subgenus of *Otinia* Antoine, 1942 in Blaptinae: Dendarini: Melambiina.

*Oroptera* Borchmann, 1916a: 48, 104 [F]. Type species: *Oropteraphysoptera* Borchmann, 1916, by monotypy. Status: valid genus in Lagriinae: Lagriini: Lagriina.

*Orostegastopsis* Koch, 1962c: 255 [F]. Type species: *Stegastopsisscorteccii* Koch, 1962, by monotypy. Status: valid genus in Pimeliinae: Tentyriini.

*Orphelops* Gozis, 1910: 102 [M]. Type species: *Helopsimpressicollis* Faldermann, 1837 (= *Helopsfaldermanni* Faldermann, 1837), by original designation. Status: junior synonym of *Nalassus* Mulsant, 1854 in Tenebrioninae: Helopini: Cylindrinotina. Synonymy: **new synonym** [YB]. Note: this genus-group taxon has been forgotten in the literature; its type species is currently placed in the nominotypical subgenus of *Nalassus* Mulsant, 1854 and for that reason Gozis’s name is regarded as a new junior synonym of *Nalassus*.

*Ortheolus* Casey, 1907: 380 [M]. Type species: *Schoenicusoculatus* Champion, 1884, by original designation. Status: valid genus in Pimeliinae: Epitragini.

*Orthogonoderes* Solier, 1841a: 233 [M]. Type species: *Praocissubreticulatus* Solier, 1841, by subsequent designation (Flores and Pizarro-Araya 2014: 68). Status: valid subgenus of *Praocis* Eschscholtz, 1829 in Pimeliinae: Praociini.

*Orthonychius* Gebien, 1926: 83 [M]. Type species: *Orthonychiusdigitatus* Gebien, 1926, by monotypy. Status: junior synonym of *Trilobocara* Solier, 1851 in Pimeliinae: Trilobocarini. Synonymy: Doyen (1994: 501).

*Orthostibia* Blaisdell, 1923: 235 [F]. Type species: *Orthostibiafrontalis* Blaisdell, 1923, by original designation. Status: valid genus in Pimeliinae: Edrotini.

*Osdara* Walker, 1858: 284 [F]. Type species: *Osdarapicipes* Walker, 1858, by monotypy. Status: valid genus and subgenus in Stenochiinae: Cnodalonini.

*Osdaroides* Kaszab, 1980b: 324 [M]. Type species: *Osdaroidesmetallicus* Kaszab, 1980, by original designation. Status: valid genus in Stenochiinae: Cnodalonini. Note: *Osdaroides* was used earlier by Kaszab (1979a: 108) without a description, a definition or a bibliographic reference to such a published statement (ICZN 1999, Article 13.1) and is therefore unavailable from that date.

*Ospidus* Pascoe, 1866a: 467 [M]. Type species: *Ospiduschrysomeloides* Pascoe, 1866, by monotypy. Status: valid genus in Tenebrioninae: Heleini: Heleina.

*Ossiporis* Pascoe, 1866a: 451 [F]. Type species: *Ossiporisterrena* Pascoe, 1866, by monotypy. Status: valid genus in Pimeliinae: Sepidiini: Trachynotina.

*Osternus* Fairmaire, 1895b: 446 [M]. Type species: *Osternusopacicollis* Fairmaire, 1895, by monotypy. Status: valid genus and subgenus in Stenochiinae: Cnodalonini.

*Ostorius* Fairmaire, 1889c: xxxiii [M]. Type species: *Ostoriusmulticostatus* Fairmaire, 1889, by monotypy. Status: junior synonym of *Adelonia* Laporte, 1840 in Blaptinae: Pedinini: Leichenina. Synonymy: Kamiński et al. (2019a: 92).

*Oterophloeus* Desbrochers des Loges, 1881: 52 [M]. Type species: *Oterophloeuspicipes* Desbrochers des Loges, 1881 (= *Pachychilahumerosus* Fairmaire, 1875), by monotypy. Status: valid genus in Pimeliinae: Tentyriini.

*Oteroscelis* Solier, 1835b: 546 [F]. Type species: *Adesmiapulcherrima* Solier, 1835 (= *Adesmiaaudouini* Solier, 1835), by subsequent designation (Hope 1841: 118). Status: valid subgenus of *Adesmia* Fischer, 1822 in Pimeliinae: Adesmiini.

*Oteroscelopsis* Löbl & Merkl in Löbl et al., 2020: 1 [F]. Type species: *Pimeliadilatata* Klug, 1830, by original designation. Status: valid subgenus of *Adesmia* Fischer, 1822 in Pimeliinae: Adesmiini. Note: name first proposed by Koch (1944b: 147) without fixation of a type species in the original publication (ICZN 1999, Article 13.3); Löbl and Merkl (2003: 244) designated *Pimeliadilatata* Klug, 1830 as the type species of Koch’s name but did not explicitly indicate the genus-group name as intentionally new (ICZN 1999, Article 16.1).

*Othelecta* Pascoe, 1866a: 488 [F]. Type species: *Othelectatorrida* Pascoe, 1866, by monotypy. Status: junior synonym of *Cylindrothorus* Solier, 1843 in Alleculinae: Alleculini: Mycetocharina. Synonymy: Haag-Rutenberg (1879c: 412).

*Othryades* Champion, 1889: 72 [M]. Type species: *Othryadesfragilicornis* Champion, 1889, by monotypy. Status: valid genus in Lagriinae: Lagriini: Statirina.

*Othryoneus* Champion, 1886: 245 [M]. Type species: *Othryoneuserotyloides* Champion, 1886, by subsequent designation (Gebien 1942a: 315). Status: valid genus in Stenochiinae: Cnodalonini.

*Otinia* Antoine, 1942: 22, 44 [F]. Type species: *Otiniaiblanensis* Antoine, 1942, by monotypy. Status: valid genus and subgenus in Blaptinae: Dendarini: Melambiina.

*Otoceromorphus* Pic, 1915d: 11 [M]. Type species: *Otoceromorphusgounellei* Pic, 1915, by monotypy. Status: valid genus in Stenochiinae: Stenochiini.

*Otocerus* Mäklin, 1867: 484 [M]. Type species [automatic]: *Strongyliumserraticorne* Guérin-Méneville, 1834, by monotypy. Status: junior synonym of *Oploptera* Chevrolat, 1844 in Stenochiinae: Stenochiini. Note: unnecessary replacement name for *Oploptera* Chevrolat, 1844 (as “*Hoploptera*”).

*Otrintus* Pascoe, 1866a: 483 [M]. Type species: *Prosodesbehrii* Germar, 1848, by original designation. Status: junior synonym of *Cardiothorax* Motschulsky, 1860 in Lagriinae: Adeliini. Synonymy: Bates (1879c: 30).

*Otys* Champion, 1895a: 221 [M]. Type species: *Otysharpalinus* Champion, 1895 (= *Scaletomerusproximus* Blackburn, 1891), by subsequent designation (R. Lucas 1920: 468). Status: junior synonym of *Scaletomerus* Blackburn, 1891 in Alleculinae: Alleculini: Alleculina. Synonymy: Carter (1915a: 78). Note: Matthews and Bouchard (2008: 333) selected *Otysarmatus* Champion, 1895 as the type species of this genus (and treated *Otys* Champion, 1895 as valid) not knowing of the earlier valid designation by R. Lucas (1920: 468).

*Oubanghinum* Pic, 1933: 4 [N]. Type species: *Oubanghinumatrum* Pic, 1933, by monotypy. Status: junior synonym of *Heterotarsus* Latreille, 1829 in Blaptinae: Opatrini: Heterotarsina. Synonymy: Ardoin (1969b: 126).

*Ovalobioramix* Egorov, 2004: 603 [F]. Type species: *Platyscelismolesta* Bogatchev, 1947, by original designation. Status: valid subgenus of *Bioramix* Bates, 1879 in Blaptinae: Platyscelidini.

*Ovaloodescelis* Kaszab, 1940b: 940, 947 [F]. Type species: *Platyscelisaffinis* Seidlitz, 1893, by original designation. Status: valid subgenus of *Oodescelis* Motschulsky, 1845 in Blaptinae: Platyscelidini.

*Overlaetia* Pic, 1937b: 304 [F]. Type species: *Overlaetiagracilitarsis* Pic, 1937, by monotypy. Status: valid genus in Tenebrioninae: Amarygmini. Note: as pointed out by Bremer and Lillig (2014: 118) the older name *Overlaetia* Schouteden, 1932 [Hemiptera] is not nomenclaturally available and therefore *Overlaetia* Pic, 1937 is not a junior homonym.

*Oxidates* Champion, 1886: 263 [M]. Type species: *Oxidatesplanicollis* Champion, 1886, by subsequent designation (Gebien 1943: 402). Status: valid genus in Stenochiinae: Cnodalonini.

*Oxinthas* Champion, 1884: 72 [M]. Type species: *Oxinthaspraocioides* Champion, 1884, by monotypy. Status: valid genus in Pimeliinae: Branchini.

*Oxura* W. Kirby, 1819a: 413 [F]. Type species: *Oxurasetosa* W. Kirby, 1819, by monotypy. Status: valid genus in Pimeliinae: Sepidiini: Oxurina.

*Oxycara* Solier, 1835b: 253, 254 [N]. Type species: *Oxycarablapsoides* Solier, 1835, by monotypy. Status: valid genus and subgenus in Pimeliinae: Tentyriini.

*Oxycarops* Reitter, 1900c: 94 [M]. Type species: *Hegeterfuscipes* Brullé, 1839, by subsequent designation (Gebien 1937a: 640). Status: valid genus in Pimeliinae: Tentyriini.

*Oxycerus* Koch, 1955a: 46 [M]. Type species: *Trachynotusresolutus* Péringuey, 1904, by original designation. Status: valid genus in Pimeliinae: Sepidiini: Trachynotina.

*Oxyge* Chatanay, 1914b: 7 [F]. Type species: *Oxygerugosa* Chatanay, 1914, by original designation. Status: valid genus in Pimeliinae: Asidini.

*Oxygonodera* Casey, 1907: 433, 444 [F]. Type species: *Oxygonoderavillosa* Casey, 1907, by original designation. Status: valid genus in Pimeliinae: Edrotini.

*Oxypistoma* Löbl, Bouchard, Merkl & Bousquet, 2020: 5 [N]. Type species: *Prochomabucculentum* Koch, 1940, by original designation. Status: valid subgenus of *Prochoma* Solier, 1835 in Pimeliinae: Tentyriini. Note: name first proposed with two alternative spellings, as *Oxypistoma* (Koch 1940a: 258, 259, 260, 262) and *Oxipistoma* (Koch 1940a: 259), without fixation of a type species in the original publication (ICZN 1999, Article 13.3); Löbl et al. (2008a: 40) designated *Prochomabucculenta* Koch, 1940 as the type species of Koch’s name but did not explicitly indicate the genus-group name as intentionally new (ICZN 1999, Article 16.1).

*Oxythorax* Fåhraeus, 1870: 288 [M]. Type species: *Oxythoraxclathratus* Fåhraeus, 1870, by monotypy. Status: junior synonym of *Anchophthalmus* Gerstaecker, 1854 in Blaptinae: Platynotini: Platynotina. Synonymy: Péringuey (1904: 238).

*Oxyura* Agassiz, 1846b: 267, 268 [F]. Type species [automatic]: *Oxurasetosa* W. Kirby, 1819, by monotypy. Status: junior synonym of *Oxura* W. Kirby, 1819 in Pimeliinae: Sepidiini: Oxurina. Note: unjustified emendation of *Oxura* W. Kirby, 1819, not in prevailing usage; junior homonym of *Oxyura* Bonaparte, 1831 [Aves].

*Oyanus* Pic, 1921: 23 [M]. Type species: *Oyanuscurticornis* Pic, 1921, by monotypy. Status: junior synonym of *Cleomis* Fairmaire, 1892 in Stenochiinae: Cnodalonini. Synonymy: Löbl et al. (2008b: 340).

*Ozaenimorphus* Fairmaire, 1882b: 127 [M]. Type species: *Ozaenimorphuscostulipennis* Fairmaire, 1882, by monotypy. Status: valid genus in Stenochiinae: Cnodalonini.

*Ozolais* Pascoe, 1866a: 457 [F]. Type species: *Ozolaisscruposa* Pascoe, 1866, by monotypy. Status: valid genus in Tenebrioninae: Toxicini: Dysantina.

*Ozotypoides* Kaszab, 1982b: 222 [M]. Type species: *Ozotypoidesgranulatus* Kaszab, 1982, by original designation. Status: valid genus in Lagriinae: Adeliini.

*Ozotypus* Pascoe, 1862: 328 [M]. Type species: *Ozotypussetosus* Pascoe, 1862, by monotypy. Status: valid genus in Stenochiinae: Cnodalonini.

*Pachacamacius* Flores & Giraldo-Mendoza in Giraldo-Mendoza and Flores, 2019: 84 [M]. Type species: *Pachacamaciusaguilari* Giraldo-Mendoza & Flores, 2019, by original designation. Status: valid genus in Pimeliinae: Edrotini.

*Pachycera* Eschscholtz, 1831: 5, 7 [F]. Type species: **fixed herein** (ICZN 1999, Article 70.3) as *Tenebriobuprestoides* Fabricius, 1781, misidentified as *Akislaevigata* Fabricius, 1801 in the original designation by monotypy in Eschscholtz (1831). Status: junior synonym of *Hyperops* Eschscholtz, 1831 in Pimeliinae: Tentyriini. Synonymy: Löbl et al. (2008b: 192). Note: junior homonym of *Pachycera* Billberg, 1820 [Hemiptera]; the type species “*Akislaevigata* Fabricius” was first established by monotypy; Koch (1943: 530) noted that *Akislaevigata* Fabricius of Eschscholtz (1831) was misidentified and corresponded to the species *Tenebriobuprestoides* Fabricius, 1781; we follow currently accepted concepts (e.g., Löbl et al. 2008b: 192) and fix the type species according to the requirements of Article 70.3.2 (ICZN 1999); the nominal species *Akislaevigata* Fabricius, 1801 also belongs to the genus *Hyperops* Eschscholtz, 1831; the original combination of the accepted name of the type species, *Tenebriobuprestoides* Fabricius, 1781, is a junior primary homonym of *Tenebriobuprestoides* Scopoli, 1763 and *Tenebriobuprestoides* Pallas, 1773.

*Pachycerops* Koch, 1943a: 524, 533 [M]. Type species: *Pachycerainsidiosa* Fairmaire, 1896, by monotypy. Status: valid subgenus of *Hyperops* Eschscholtz, 1831 in Pimeliinae: Tentyriini.

*Pachycerus* Montrouzier, 1860: 291 [M]. Type species: *Pachycerusdomesticus* Montrouzier, 1860, by monotypy. Status: senior synonym of *Sciophagus* Sharp, 1885 in Diaperinae: Diaperini: Diaperina. Synonymy: Fauvel (1904: 185). Note: junior homonym of *Pachycerus* Schönherr, 1823 [Coleoptera: Curculionidae].

*Pachychila* Eschscholtz, 1831: 5 [F]. Type species: *Pimeliapunctata* Fabricius, 1798, by monotypy. Status: valid genus and subgenus in Pimeliinae: Tentyriini. Note: the original combination of the name of the type species, *Pimeliapunctata* Fabricius, 1798, is a junior primary homonym of *Pimeliapunctata* Thunberg, 1787.

*Pachychile* Lacordaire, 1859a: 46 [F]. Type species [automatic]: *Pimeliapunctata* Fabricius, 1798, by monotypy. Status: junior synonym of *Pachychila* Eschscholtz, 1831 in Pimeliinae: Tentyriini. Note: unjustified emendation of *Pachychila* Eschscholtz, 1831, not in prevailing usage. Note: the original combination of the name of the type species, *Pimeliapunctata* Fabricius, 1798, is a junior primary homonym of *Pimeliapunctata* Thunberg, 1787.

*Pachychilina* Reitter, 1900c: 91, 145 [F]. Type species: *Tentyriadejeani* Besser, 1832, by subsequent designation (R. Lucas 1920: 473). Status: valid subgenus of *Pachychila* Eschscholtz, 1831 in Pimeliinae: Tentyriini.

*Pachycoelia* Boisduval, 1835: 248 [F]. Type species: *Pachycoeliasulcicollis* Boisduval, 1835, by monotypy. Status: valid genus in Tenebrioninae: Heleini: Cyphaleina. Note: name incorrectly listed as preoccupied in the literature (e.g., Matthews and Bouchard 2008: 296) but used as valid recently (Matthews and Lawrence 2019: 632).

*Pachycossyphodes* Andreae, 1961: 203, 214 [M]. Type species: *Cossyphodesmachadoi* Basilewsky, 1952, by original designation. Status: junior synonym of *Cossyphodes* Westwood, 1851 in Pimeliinae: Cossyphodini: Cossyphodina. Synonymy: Schawaller (2013c: 362, implied by inclusion of *Cossyphodesmachadoi* Basilewsky, 1952 in Cossyphodes Westwood, 1851 without use of a subgenus rank).

*Pachycyrtosoma* Marcuzzi, 1999: 81 [N]. Type species: *Cyrtosomamerkli* Marcuzzi, 1999, by original designation. Status: junior synonym of *Nesocyrtosoma* Marcuzzi, 1976 in Stenochiinae: Cnodalonini. Synonymy: Hopp and Ivie (2009: 13).

*Pachylagria* Borchmann, 1912a: 17 [F]. Type species: *Pachylagriaovata* Borchmann, 1912, by subsequent designation (Merkl 2008: 115). Status: junior synonym of *Xanthalia* Fairmaire, 1894 in Lagriinae: Lagriini: Statirina. Synonymy: Borchmann (1916a: 163, with *Heterogria* Fairmaire, 1896, a junior synonym of *Xanthalia* Fairmaire, 1894).

*Pachylesthus* Fairmaire, 1897e: 219 [M]. Type species: *Pachylesthusvalidus* Fairmaire, 1897, by monotypy. Status: valid genus in Stenochiinae: Cnodalonini.

*Pachylocerus* Hope, 1841: 186 [M]. Type species: *Pachyloceruswestermanni* Hope, 1841, by monotypy. Status: senior synonym of *Pycnocerus* Westwood, 1841 in Lagriinae: Pycnocerini. Note: junior homonym of *Pachylocerus* Hope, 1834 [Coleoptera: Cerambycidae].

*Pachylodera* Quedenfeldt, 1890: 399 [F]. Type species: *Pachyloderabrevicornis* Quedenfeldt, 1890, by monotypy. Status: valid genus in Pimeliinae: Pimeliini.

*Pachymastus* Fairmaire, 1896b: 350 [M]. Type species: *Pachymastusasperulus* Fairmaire, 1896, by monotypy. Status: valid genus in Blaptinae: Opatrini: incertae sedis. Note: placed in Opatrini incertae sedis by Kamiński et al. (2021b: 151).

*Pachynotelus* Solier, 1841a: 210, 267 [367] [M]. Type species: *Pachynotelusalbiventris* Solier, 1841, by original designation. Status: valid genus in Pimeliinae: Cryptochilini: Cryptochilina.

*Pachyphaleria* Gebien, 1920: 136 [F]. Type species: *Phaleriacapensis* Laporte, 1840, by monotypy. Status: valid genus in Diaperinae: Phaleriini.

*Pachypterocoma* Skopin, 1974b: 157 [F]. Type species: *Pterocomapallasi* Semenov-Tjan-Shansky, 1910, by original designation. Status: valid subgenus of *Pterocoma* Dejean, 1834 in Pimeliinae: Pimeliini.

*Pachypterus* P.H. Lucas, 1847: pl. 29 [M]. Type species: *Pachypterusmauritanicus* P.H. Lucas, 1847, by monotypy. Status: senior synonym of *Neopachypterus* Bouchard, Löbl & Merkl, 2007 in Blaptinae: Opatrini: Neopachypterina. Note: junior homonym of *Pachypterus* Swainson, 1839 [Pisces].

*Pachyscelina* Kwieton, 1978: 29, 30 [F]. Type species: *Pachyscelismicros* Kaszab, 1970, by original designation. Status: valid genus in Pimeliinae: Pimeliini.

*Pachyscelis* Solier, 1836: 9, 54 [F]. Type species [automatic]: *Pimeliamusiva* Ménétriés, 1832, by subsequent designation (Bouchard et al. 2007: 388). Status: valid genus and subgenus in Pimeliinae: Pimeliini. Note: unnecessary replacement name for *Brachyscelis* Dejean, 1834. Note: nomen protectum (see Bouchard et al. 2007: 388).

*Pachyscelodes* Sénac, 1887: 189 [M]. Type species: *Pachyscelishenoni* Sénac, 1887, by subsequent designation (Gebien 1937a: 823). Status: junior synonym of *Scelace* Marseul, 1887 in Pimeliinae: Pimeliini. Synonymy: Reitter (1915: 2).

*Pachysternoplax* Skopin, 1973: 110, 154 [F]. Type species: *Trigonoscelisarmeniaca* Faldermann, 1837, by original designation. Status: valid subgenus of *Sternoplax* Frivaldszky, 1890 in Pimeliinae: Pimeliini.

*Pachystira* Chen, 1997: 308 [F]. Type species: *Pachystiraimpressipennis* Chen, 1997, by original designation. Status: valid genus in Lagriinae: Lagriini: Statirina.

*Pachyurgus* J.L. LeConte, 1862: 230 [M]. Type species: *Iphthinusaereus* Melsheimer, 1846, by original designation. Status: junior synonym of *Derosphaerus* J. Thomson, 1858 in Stenochiinae: Cnodalonini. Synonymy: J.L. LeConte (1873: 335, with *Encyalesthus* Motschulsky, 1860, a junior synonym of *Derosphaerus* J. Thomson, 1858). Note: the type locality for *Iphthinusaereus* Melsheimer, 1846 was incorrectly given as “Pennsylvania” originally (see Bousquet et al. 2018: 384).

*Pactostoma* J.L. LeConte, 1858b: 19 [N]. Type species: *Asidaanastomosis* Say, 1824, by original designation. Status: junior synonym of *Stenosides* Solier, 1836 in Pimeliinae: Asidini. Synonymy: J.L. LeConte (1862: 222, with *Ologlyptus* Lacordaire, 1858, a junior synonym of *Stenosides* Solier, 1836).

*Paita* Fauvel, 1904: 173 [F]. Type species: *Paitasetosella* Fauvel, 1904, by monotypy. Status: junior synonym of *Menimus* Sharp, 1876 in Diaperinae: Gnathidiini: Gnathidiina. Synonymy: Kaszab (1982b: 56).

*Paivaea* Wollaston, 1864: 449 [F]. Type species: *Tentyriahispida* Brullé, 1839, by monotypy. Status: valid genus in Pimeliinae: Tentyriini.

*Paivea* Scudder, 1882: 213 [F]. Type species [automatic]: *Tentyriahispida* Brullé, 1839, by monotypy. Status: junior synonym of *Paivaea* Wollaston, 1864 in Pimeliinae: Tentyriini. Note: unjustified emendation of *Paivaea* Wollaston, 1864, not in prevailing usage.

†*Palaeobasanus* Nabozhenko & Kirejtshuk, 2020: 25 [M]. Type species: *Palaeobasanusneli* Nabozhenko & Kirejtshuk, 2020, by original designation. Status: valid genus in Diaperinae: Scaphidemini. Note: described from Middle-Upper Paleocene deposits (France).

†*Palaeosclerum* Nabozhenko & Kirejtshuk, 2017: 308 [N]. Type species: *Palaeosclerumpohli* Nabozhenko & Kirejtshuk, 2017, by original designation. Status: valid genus in Blaptinae: Opatrini: Sclerina. Note: described from Middle-Upper Paleocene deposits (France).

*Palembomimus* Matthews & Lawrence, 2005: 539 [M]. Type species: *Platydemadeplanata* Champion, 1894, by original designation. Status: valid genus in Diaperinae: Diaperini: Adelinina.

*Palembus* Casey, 1891: 65 [M]. Type species: *Palembusocularis* Casey, 1891, by monotypy. Status: junior synonym of *Ulomoides* Blackburn, 1888 in Diaperinae: Diaperini: Diaperina. Synonymy: Doyen et al. (1990: 237).

*Palorinus* Blair, 1930: 135 [M]. Type species: *Palorushumeralis* Gebien, 1914, by original designation. Status: valid genus in Tenebrioninae: Palorini.

*Paloropsis* Masumoto & Grimm, 2004: 127 [F]. Type species: *Paloropsisirei* Masumota & Grimm, 2004, by original designation. Status: valid genus in Tenebrioninae: Palorini.

*Palorus* Mulsant, 1854: 250 [M]. Type species: *Hypophlaeusdepressus* Fabricius, 1790, by monotypy. Status: valid genus in Tenebrioninae: Palorini.

*Palpafrina* Koch, 1950b: 329 [F]. Type species: *Afrinuswatsoni* Koch, 1950, by original designation. Status: valid subgenus of *Afrinus* Fairmaire, 1888 in Pimeliinae: Tentyriini.

*Palpichara* Borchmann, 1932a: 355 [F]. Type species: *Palpicharaserricornis* Borchmann, 1932, by original designation. Status: valid genus in Alleculinae: Alleculini: Alleculina.

*Palpicula* Novák, 2018a: 168 [F]. Type species: *Alleculafiliola* Borchmann, 1925, by original designation. Status: valid genus in Alleculinae: Alleculini: Alleculina.

*Palpomodes* Koch, 1952d: 223 [M]. Type species: *Psammodesphysopterus* Gebien, 1920, by monotypy. Status: valid genus and subgenus in Pimeliinae: Sepidiini: Oxurina.

*Pandarinus* Mulsant & Rey, 1854: 50, 103 [M]. Type species: *Pandarinustenellus* Mulsant & Rey, 1854, by subsequent designation (Chatzimanolis and Löbl 2003: 260). Status: valid subgenus of *Dendarus* Dejean, 1821 in Blaptinae: Dendarini: Dendarina.

*Pandarus* Dejean, 1834: 191 [M]. Type species [automatic]: Type species: *Helopstristis* Rossi, 1790, by subsequent designation Blanchard (1844: pl. 48). Status: junior synonym of *Dendarus* Dejean, 1821 in Blaptinae: Dendarini: Dendarina. Note: unjustified emendation of *Dendarus* Dejean, 1821, not in prevailing usage (see Bousquet and Bouchard 2013a: 48); junior homonym of *Pandarus* Leach, 1816 [Crustacea].

*Paniasis* Champion, 1886: 208 [M]. Type species: *Paniasisdilatipes* Champion, 1886, by monotypy. Status: valid genus in Diaperinae: Diaperini: Diaperina.

*Paoligena* Pic, 1928c: 42 [F]. Type species: *Praogenainhumeralis* Pic, 1928, by monotypy. Status: valid genus in Tenebrioninae: Paoligenini. Note: originally described as a subgenus of *Praeugena* Laporte, 1840, elevated to the rank of genus by De Moor (1970: 15).

*Papuamisolampus* Kaszab, 1986: 288 [M]. Type species: *Papuamisolampustoxopeusi* Kaszab, 1986, by original designation. Status: valid genus in Stenochiinae: Cnodalonini.

*Parabantodemus* Iwan, 2000: 345 [M]. Type species: *Trigonopusspinipes* Mulsant & Rey, 1853, by original designation. Status: valid genus in Blaptinae: Platynotini: Platynotina.

*Parabigopsis* Español, 1946: 111, 114 [F]. Type species: *Parabigopsispeyerimhoffi* Español, 1946, by original designation. Status: valid genus in Pimeliinae: Tentyriini.

*Parablops* Rottenberg, 1871: 254 [M]. Type species: *Parablopsaetnensis* Rottenberg, 1871, by monotypy. Status: senior synonym of *Gerandryus* Rottenberg, 1873 in Alleculinae: Alleculini: Gonoderina. Note: junior homonym of *Parablops* Schönherr, 1839 [Coleoptera: Anthribidae].

*Parabolitophagus* Miyatake, 1964: 67, 70 [M]. Type species: *Bolitophagusfelix* Lewis, 1894, by original designation. Status: valid genus in Tenebrioninae: Bolitophagini.

*Paracirta* Schuster, 1930: 66 [F]. Type species: *Paracirtaschatzmayri* Schuster, 1930, by monotypy. Status: valid genus in Pimeliinae: Tentyriini.

*Paracistela* Borchmann, 1941a: 30 [F]. Type species: *Paracistelavariabilis* Borchmann, 1941, by monotypy. Status: valid genus in Alleculinae: Alleculini: Gonoderina.

*Paracossyphus* Viñolas & Cartagena, 2005: 29, 55 [M]. Type species [automatic]: *Cossyphusminutissimus* Laporte, 1840, by original designation. Status: junior synonym of *Acontodactylus* Desbrochers des Loges, 1894 in Lagriinae: Cossyphini. Note: unnecessary replacement name for *Acontodactylus* Desbrochers des Loges, 1894 (as “*Acanthodactylus*”).

*Paradissonomus* G.S. Medvedev, 1968a: 229 [M]. Type species: *Dissonomuslongulus* Bogatchev & Kryzhanovsky, 1960, by original designation. Status: valid subgenus of *Dissonomus* Jacquelin du Val, 1861 in Tenebrioninae: Dissonomini.

*Paradrus* Jakobson, 1924: 242 [M]. Type species [automatic]: *Pseudhadrusseriatus* Kolbe, 1910, by subsequent designation (Gebien 1941: 332). Status: junior synonym of *Pseudhadrus* Kolbe, 1910 in Stenochiinae: Cnodalonini. Note: unnecessary replacement name for *Pseudhadrus* Kolbe, 1910.

*Paragena* Bouchard & Bousquet, **new subgenus** [F]. Type species: *Nesogenaviridicuprea* Fairmaire, 1868, by **present designation**. Status: valid subgenus of *Nesogena* Mäklin, 1863 in Tenebrioninae: Praeugenini. Note: first proposed by Froussart (1961: 60) without type species designation; the subgenusParagena, which is currently used as valid, is therefore unavailable (ICZN 1999, Articles 13.3, 16.1); we hereby make the name available by selecting *Nesogenaviridicuprea* Fairmaire, 1868 as type species and referring to Froussart (1961: 60) for the character states that characterise and differentiate *Paragena*.

*Paragonocnemis* Kraatz, 1899: 118 [F]. Type species: *Paragonocnemissculpticollis* Kraatz, 1899 (= *Gonocnemisfoveicollis* Fairmaire, 1891), by subsequent designation (Gebien 1943: 918). Status: valid genus and subgenus in Tenebrioninae: Amarygmini.

*Paraguania* Marcuzzi, 1953: 31 [F]. Type species: *Paraguaniarelicta* Marcuzzi, 1953, by monotypy. Status: valid genus in Pimeliinae: Edrotini.

*Parahymenorus* Campbell, 1971: 100 [M]. Type species: *Parahymenorusmetallicus* Campbell, 1971, by original designation. Status: valid genus in Alleculinae: Alleculini: Alleculina.

*Parahyocis* Kaszab, 1955b: 650 [M]. Type species: *Hyocischampioni* Fauvel, 1904, by original designation. Status: valid genus in Diaperinae: Hyociini: Hyociina.

*Paraisomira* Dubrovina, 1982: 138 [F]. Type species: *Cistelaoculata* Marseul, 1876, by original designation. Status: valid subgenus of *Isomira* Mulsant, 1856 in Alleculinae: Alleculini: Gonoderina.

†*Parakeleusticus* Haupt, 1950: 114, 126 [M]. Type species: *Parakeleusticuspostumus* Haupt, 1950, by original designation. Status: valid genus in Stenochiinae: incertae sedis. Note: described from Middle Eocene deposits (Germany).

*Paraleptodes* G.S. Medvedev, 1967: 354 [M]. Type species: *Leptodeslindbergi* Kaszab, 1959, by original designation. Status: valid subgenus of *Leptodes* Dejean, 1834 in Pimeliinae: Leptodini.

*Paralitoborus* Antoine, 1931: 190 [M]. Type species: *Litoborussternalis* Fairmaire, 1884, by original designation. Status: valid subgenus of *Litoborus* Mulsant & Rey, 1854 in Blaptinae: Dendarini: Melambiina.

*Paralorelopsis* Marcuzzi, 1994: 117 [F]. Type species: *Paralorelopsisbordoni* Marcuzzi, 1994, by monotypy. Status: valid genus in Lagriinae: Lupropini.

*Paralyreus* Grouvelle, 1918: 24 [M]. Type species: *Paralyreusscotti* Grouvelle, 1918, by original designation. Status: valid genus in Diaperinae: Gnathidiini: Anopidiina.

*Paramarygmus* Quedenfeldt, 1885: 25 [M]. Type species: *Paramarygmusnigroaeneus* Quedenfeldt, 1885 (= *Hybonotusfemoralis* Imhoff, 1843), by monotypy. Status: valid genus and subgenus in Tenebrioninae: Amarygmini.

*Paramellon* C.O. Waterhouse, 1882b: iv [N]. Type species: *Paramellonsociale* C.O. Waterhouse, 1882, by monotypy. Status: valid genus in Pimeliinae: Cossyphodini: Paramellonina.

*Paramellops* Andreae, 1961: 201 [M]. Type species: *Cossyphodesbewicki* Wollaston, 1861, by original designation. Status: valid genus in Pimeliinae: Cossyphodini: Cossyphodina.

*Paramisolampidius* Merkl & Masumoto in Löbl et al., 2020: 4 [M]. Type species: *Paramisolampidiuskagoshimensis* Nakane, 1968, by original designation. Status: valid genus in Stenochiinae: Cnodalonini. Note: name first proposed by Nakane (1968: 82) without fixation of a type species in the original publication (ICZN 1999, Article 13.3); Merkl and Masumoto (2007: 1) designated *Paramisolampidiuskagoshimensis* Nakane, 1968 as the type species of Nakane’s name but did not explicitly indicate the genus-group name as intentionally new (ICZN 1999, Article 16.1).

*Paranemia* Heyden, 1892: 103 [F]. Type species: *Paranemiaschroederi* Heyden, 1892, by monotypy. Status: valid genus in Diaperinae: Phaleriini.

*Paranephodes* Antoine, 1955: 208 [M]. Type species: *Nephodescamusi* Antoine, 1955, by monotypy. Status: valid subgenus of *Nephodinus* Gebien, 1943 in Tenebrioninae: Helopini: Helopina.

*Paranopidium* Dajoz, 1974: 429, 434 [N]. Type species: *Paranopidiumafricanum* Dajoz, 1974, by original designation. Status: valid genus in Diaperinae: Gnathidiini: Anopidiina.

*Parapachynotela* Koch, 1952c: 54 [F]. Type species: *Parapachynotelabushmanica* Koch, 1952, by original designation. Status: junior synonym of *Horatoma* Solier, 1841 in Pimeliinae: Cryptochilini: Cryptochilina. Synonymy: Penrith and Endrödy-Younga (1994: 10).

*Parapachyscelis* Kwieton, 1978: 29 [F]. Type species: *Pimeliavillosa* Drapiez, 1820, by original designation. Status: valid subgenus of *Pachyscelis* Solier, 1836 in Pimeliinae: Pimeliini.

*Paraphanes* W.J. MacLeay, 1887: 308 [M]. Type species: *Paraphanesnitidus* W.J. MacLeay, 1887, by monotypy. Status: valid genus in Tenebrioninae: Heleini: Cyphaleina.

*Paraphloeus* Seidlitz, 1894: 553, 557 [M]. Type species: *Hypophlaeuslongulus* Gyllenhal, 1827, by subsequent designation (G.S. Medvedev 1990: 231). Status: junior synonym of *Corticeus* Piller & Mitterpacher, 1783 in Diaperinae: Hypophlaeini. Synonymy: Bremer (1995: 3).

*Paraplatyope* Löbl, Bouchard, Merkl & Bousquet, 2020: 4 [F]. Type species: *Leucolaephusarabicus* Blair, 1931, by original designation. Status: valid genus in Pimeliinae: Pimeliini. Note: name first proposed by Gridelli (1953: 45) without fixation of a type species in the original publication (ICZN 1999, Article 13.3).

*Paraplatyscelis* Kaszab, 1940b: 912, 936 [F]. Type species: *Platyscelissinuata* Seidlitz, 1893, by original designation. Status: valid subgenus of *Platyscelis* Latreille, 1818 in Blaptinae: Platyscelidini.

*Parapraocis* Flores & Giraldo, 2020: 35 [M]. Type species: *Praocisvagecostatus* Fairmaire, 1902, by original designation. Status: valid genus in Pimeliinae: Praociini. Note: name originally proposed by Kulzer (1958a: 13, 58) as subgenus of *Praocis* Eschscholtz, 1829 without type species designation; Flores and Pizarro-Araya (2012: 4) designated *Praocisvagecostatus* Fairmaire, 1902 as type species, but did not explicitely propose the taxon as new (ICZN 1999, Article 16.1).

*Paraprosodes* Reitter, 1909a: 119 [M]. Type species: *Prosodestriangulipes* Reitter, 1909, by original designation. Status: junior synonym of *Prosodella* Reitter, 1909 in Blaptinae: Blaptini: Prosodina. Synonymy: G.S. Medvedev (1997: 597).

*Parapterocoma* Skopin, 1974b: 149 [F]. Type species: *Pterocomavittata* Frivaldszky, 1890, by original designation. Status: valid subgenus of *Pterocoma* Dejean, 1834 in Pimeliinae: Pimeliini.

*Paraselinus* Kamiński, 2013: 705, 711 [M]. Type species: *Paraselinusiwani* Kamiński, 2013, by original designation. Status: valid genus in Blaptinae: Platynotini: Platynotina.

*Parasida* Casey, 1912: 76, 126 [F]. Type species: *Parasidalaciniata* Casey, 1912, by original designation. Status: senior synonym of *Pleisiasida* Smith, 2013 in Pimeliinae: Asidini. Note: junior homonym of *Parasida* Daday, 1904 [Crustacea].

*Parasternoplax* Skopin, 1973: 110, 148 [F]. Type species: *Pimeliadeplanata* Krynicki, 1832, by original designation. Status: valid subgenus of *Sternoplax* Frivaldszky, 1890 in Pimeliinae: Pimeliini.

*Parastizopus* Gebien, 1938b: 93 [M]. Type species: *Parastizopusdiehli* Gebien, 1938, by original designation. Status: valid genus in Blaptinae: Opatrini: Stizopodina.

*Parastrongylium* Kaszab, 1977b: 10, 24 [N]. Type species: *Strongyliumlorentzi* Gebien, 1921, by original designation. Status: valid genus in Stenochiinae: Stenochiini.

*Paratenetus* Spinola, 1845: 116 [M]. Type species: *Paratenetuspunctatus* Spinola, 1845, by subsequent designation (R. Lucas 1920: 483). Status: valid genus in Lagriinae: Goniaderini.

*Paratoxicum* Champion, 1894a: 380 [N]. Type species: *Paratoxicumiridescens* Champion, 1894, by monotypy. Status: valid genus in Tenebrioninae: Tenebrionini.

*Paravius* Casey, 1907: 332 [M]. Type species: *Emmenastusmarginatus* Casey, 1890, by monotypy. Status: valid subgenus of *Hylocrinus* Casey, 1907 in Pimeliinae: Edrotini.

*Parecatus* Fairmaire, 1900c: 245 [M]. Type species: *Parecatusplicatulus* Fairmaire, 1900, by subsequent designation (Gebien 1937a: 741). Status: junior synonym of *Scotinesthes* Fairmaire, 1895 in Pimeliinae: Asidini. Synonymy: Koch (1962a: 67).

*Parepitragus* Casey, 1907: 518 [M]. Type species: *Parepitragussolieri* Casey, 1907, by original designation. Status: valid genus in Pimeliinae: Epitragini.

*Pareupezus* Kolbe, 1889: 129 [M]. Type species: *Pareupezusglaber* Kolbe, 1889, by monotypy. Status: junior synonym of *Paramarygmus* Quedenfeldt, 1885 in Tenebrioninae: Amarygmini. Synonymy: Gebien (1921b: 148).

*Parimmedia* Gebien, 1928: 220, 226 [F]. Type species: *Parimmediaepipleuralis* Gebien, 1928, by subsequent designation (Gebien 1943: 404). Status: valid genus in Stenochiinae: Cnodalonini.

*Parmularia* Koch, 1955a: 35 [F]. Type species: *Psammodescaffer* Fåhraeus, 1870, by monotypy. Status: junior synonym of *Psammodes* W. Kirby, 1819 in Pimeliinae: Sepidiini: Molurina. Synonymy: Kamiński et al. (2019b: 31). Note: junior homonym of *Parmularia* MacGillivray, 1887 [Bryozoa].

*Paroderus* Mulsant & Rey, 1854: 111 [M]. Type species: *Pandarinuselongatus* Mulsant & Rey, 1854, by subsequent designation (Chatzimanolis and Löbl 2003: 260). Status: valid subgenus of *Dendarus* Dejean, 1821 in Blaptinae: Dendarini: Dendarina.

*Paroeatus* Gebien, 1928: 169, 178 [M]. Type species: *Paroeatusopacus* Gebien, 1928, by original designation. Status: valid genus in Stenochiinae: Cnodalonini.

*Parogria* Borchmann, 1936: 119 [F]. Type species: *Lagriaatrata* Borchmann, 1912, by original designation. Status: valid subgenus of *Cerogria* Borchmann, 1911 in Lagriinae: Lagriini: Lagriina.

*Paromophlus* Iablokoff-Khnzorian, 1983: 134, 139 [M]. Type species: *Cistelapicipes* Fabricius, 1792, by original designation. Status: junior synonym of *Phibalus* Gistel, 1856 in Alleculinae: Cteniopodini. Synonymy: Bousquet et al. (2015: 142). Note: the name *Paromophlus* was first introduced by Znojko in Ogloblin and Znojko (1950: 125) without designation of a type species and is therefore unavailable from that date.

†*Paropiophorus* Haupt, 1950: 114, 132 [M]. Type species: *Paropiophorusnitidus* Haupt, 1950, by original designation. Status: valid genus in Tenebrionidae: incertae sedis. Note: described from Middle Eocene deposits (Germany); this genus was previously considered to be “definitely not a tenebrionid” by Watt (1975: 389) but is included here following Nabozhenko (2019: 8).

*Partystona* Watt, 1992: 47 [F]. Type species: *Partystonametallica* Watt, 1992, by original designation. Status: valid genus in Tenebrioninae: Titaenini.

*Passalocharis* Koch, 1954b: 13 [F]. Type species: *Chirocharisintermedius* Gebien, 1911, by original designation. Status: valid genus in Lagriinae: Pycnocerini.

*Patagonogenius* Flores, 2000a: 371, 390 [M]. Type species: *Mitrageniusquadricollis* Fairmaire, 1876, by original designation. Status: valid genus in Pimeliinae: Nycteliini.

*Patagonopraocis* Flores & Chani-Posse, 2005: 576 [M]. Type species: *Patagonopraocismagellanicus* Flores & Chani-Posse, 2005, by original designation. Status: valid genus in Pimeliinae: Praociini.

*Paulianaria* Bouchard & Bousquet, **new genus** [F]. Type species: *Paulianariastrongylioides* Ardoin, 1961, by **present designation**. Status: valid genus in Stenochiinae: Cnodalonini. Note: Ardoin (1961d: 97) introduced the new genus name *Paulianaria* for five nominal species, but unfortunately did not designate a type species; the genus *Paulianaria*, which has been treated as valid since 1961, is therefore unavailable (ICZN 1999, Article 13.3); we hereby make the name available by selecting *Paulianariastrongylioides* Ardoin, 1961 as type species and referring to Ardoin (1971: 362) for the character states that characterise and differentiate *Paulianaria*.

*Paulianesthes* Koch, 1962a: 39, 57 [F]. Type species: *Paulianesthesamplipennis* Koch, 1962, by monotypy. Status: valid genus in Pimeliinae: Tentyriini.

*Paurodontomophlus* Muche, 1979: 171 [M]. Type species: *Omophluswittmeri* Muche, 1979, by original designation. Status: valid subgenus of *Omophlus* Dejean, 1834 in Alleculinae: Cteniopodini.

*Pechalius* Casey, 1907: 379, 420 [M]. Type species: *Pechaliussubvittatus* Casey, 1907, by original designation. Status: valid genus in Pimeliinae: Epitragini. Note: the First Reviser (*Pechalius* Casey, 1907 versus *Epitragoma* Casey, 1907) is Freude (1968: 61).

*Pectinepitragus* Pic, 1927a: 44 [M]. Type species: *Pectinepitraguspubescens* Pic, 1927, by monotypy. Status: valid genus in Pimeliinae: Epitragini.

*Pectphegoneus* Freude, 1968: 90, 98 [M]. Type species: *Schoenicuspectoralis* Champion, 1884, by monotypy. Status: valid subgenus of *Phegoneus* Casey, 1907 in Pimeliinae: Epitragini.

*Pedarasida* Reitter, 1917a: 11, 28 [F]. Type species: *Asidacariosicollis* Solier, 1836, by subsequent designation (F. Soldati 2008: 33). Status: junior synonym of *Glabrasida* Escalera, 1910 in Pimeliinae: Asidini. Synonymy: Viñolas and Cartagena (2005: 282).

*Pedinopsis* Gebien, 1910d: 157 [F]. Type species: *Pedinopsispilipes* Gebien, 1910, by monotypy. Status: senior synonym of *Loensus* R. Lucas, 1920 in Blaptinae: Pedinini: Pedinina. Note: junior homonym of *Pedinopsis* Cotteau, 1863 [Echinoidea].

*Pedinulus* Seidlitz, 1893: 364, 373 [M]. Type species: *Pedinusragusae* Baudi di Selve, 1875, by subsequent designation (Iwan and Löbl 2008: 288). Status: junior synonym of *Pedinus* Latreille, 1797 in Blaptinae: Pedinini: Pedinina. Synonymy: Kamiński and Iwan (2017: 599).

*Pedinus* Latreille, 1797: 20 [M]. Type species: *Tenebriofemoralis* Linnaeus, 1767, by subsequent designation (Stephens 1835: 159). Status: valid genus and subgenus in Blaptinae: Pedinini: Pedinina. Note: originally proposed without included nominal species; Latreille (1802: 175), by listing *Tenebriofemoralis* Linnaeus, 1767 and *Tenebriodermestoides* Fabricius, 1787 in association with this genus name, was the first author to subsequently and expressly include nominal species in *Pedinus* (ICZN 1999, Article 67.2.2).

*Pedionomus* Haag-Rutenberg, 1875c: 42 [M]. Type species: *Metriopusfavosus* Erichson, 1843, by subsequent designation (Gebien 1937a: 661). Status: senior synonym of *Alogenius* Gebien, 1910 in Pimeliinae: Adesmiini. Note: junior homonym of *Pedionomus* Gould, 1841 [Aves].

*Pediris* Motschulsky, 1872: 24 [F]. Type species: *Pedirislongipes* Motschulsky, 1872, by original designation. Status: junior synonym of *Promethis* Pascoe, 1869 in Stenochiinae: Cnodalonini. Synonymy: Gebien (1921a: 289, with *Setenis* Motschulsky, 1872, a junior synonym of *Promethis* Pascoe, 1869).

*Pedoeces* Agassiz, 1846b: 276 [M]. Type species [automatic]: *Pedonoecesgalapagoensis* G.R. Waterhouse, 1845, by subsequent designation (Aalbu and Triplehorn 1991: 170). Status: junior synonym of *Blapstinus* Dejean, 1821 in Blaptinae: Opatrini: Blapstinina. Note: unjustified emendation of *Pedonoeces* G.R. Waterhouse, 1845, not in prevailing usage.

*Pedonoeces* G.R. Waterhouse, 1845a: 32, 34 [M]. Type species: *Pedonoecesgalapagoensis* G.R. Waterhouse, 1845, by subsequent designation (Aalbu and Triplehorn 1991: 170). Status: junior synonym of *Blapstinus* Dejean, 1821 in Blaptinae: Opatrini: Blapstinina. Synonymy: Aalbu and Triplehorn (1991: 170).

*Pedostrongylium* Pic, 1916e: 11 [N]. Type species: *Strongyliumluteonotatum* Pic, 1916 (= *Stenochiaxanthozona* J. Thomson, 1858), by original designation. Status: junior synonym of *Strongylium* W. Kirby, 1819 in Stenochiinae: Stenochiini. Synonymy: Gebien (1948: 519).

*Pelecypalpus* Hinton, 1947: 91 [M]. Type species: *Pelecypalpusmedon* Hinton, 1947, by original designation. Status: junior synonym of *Scotoderus* Perroud & Montrouzier, 1865 in Stenochiinae: Cnodalonini. Synonymy: Kaszab (1973c: 258).

*Pelecyphorus* Solier, 1836: 406, 467 [M]. Type species: *Pelecyphorusmexicanus* Solier, 1836, by subsequent designation (Hope 1841: 110). Status: valid genus and subgenus in Pimeliinae: Asidini.

*Pelleas* Bates, 1872a: 98 [M]. Type species: *Tenebriocrotchii* Wollaston, 1865, by original designation. Status: valid genus in Diaperinae: Diaperini: incertae sedis.

*Pelops* Gistel, 1834: 22 [M]. Type species: *Helopsater* Fabricius, 1775, by monotypy. Status: senior synonym of *Prionychus* Solier, 1835 in Alleculinae: Alleculini: Alleculina. Note: nomen oblitum (see Bousquet and Bouchard 2017: 132).

*Pelorinus* Vauloger de Beaupré, 1900: 675, 678 [M]. Type species: *Helopsanthracinus* Germar, 1813, by subsequent designation (Cherney 2005: 382). Status: valid subgenus of *Euboeus* Boieldieu, 1865 in Tenebrioninae: Helopini: Helopina.

*Pelorocnemis* Solsky, 1876: 283 [F]. Type species: *Pimeliapunctigera* Ménétriés, 1849, by original designation. Status: valid genus in Pimeliinae: Pimeliini.

*Peltadesmia* Kuntzen, 1916: 149 [F]. Type species: *Metriopusplatynotus* Gerstaecker, 1854, by **present designation**. Status: junior synonym of *Coeladesmia* Reitter, 1916 in Pimeliinae: Adesmiini. Synonymy: Gebien (1937a: 656).

*Peltarium* Fischer von Waldheim, 1844: 106 [N]. Type species: *Peltariummarginatum* Fischer von Waldheim, 1844 (= *Blaps baerii* Fischer von Waldheim, 1842), by subsequent designation (G.S. Medvedev 1995b: 831). Status: valid subgenus of *Prosodes* Eschscholtz, 1829 in Blaptinae: Blaptini: Prosodina.

*Peltasida* Reitter, 1917a: 39, 41 [F]. Type species: *Asidafavieri* Fairmaire, 1880, by monotypy. Status: valid subgenus of *Asida* Latreille, 1802 in Pimeliinae: Asidini.

*Peltoides* Laporte, 1833a: 401 [M]. Type species: *Peltoidessenegalensis* Laporte, 1833, by subsequent designation (Lacordaire 1859a: 337). Status: valid genus and subgenus in Tenebrioninae: Alphitobiini. Note: bibliographic evidence indicates that *Oopiestus* Chevrolat, 1833 was published before *Peltoides* Laporte, 1833 and should be treated as the valid name for this genus and subgenus instead; an application to the ICZN is necessary to conserve usage of *Peltoides* Laporte, 1833.

*Peltolobus* Lacordaire, 1859a: 71 [M]. Type species [automatic]: *Megalophryspatagonica* G.R. Waterhouse, 1845, by monotypy. Status: valid genus in Pimeliinae: Trilobocarini. Note: replacement name for *Megalophrys* G.R. Waterhouse, 1845.

*Pemanoa* Buck, 1955: 269 [F]. Type species: *Pemanoamixta* Buck, 1955, by original designation. Status: valid genus in Alleculinae: Alleculini: Alleculina.

*Penadelium* Matthews, 1998: 710, 754 [N]. Type species: *Penadeliumaraucanum* Matthews, 1998, by original designation. Status: valid genus in Lagriinae: Adeliini.

*Penaus* Freude, 1968: 130 [M]. Type species: *Penauspenai* Freude, 1968, by original designation. Status: valid genus in Pimeliinae: Epitragini.

*Peneta* Lacordaire, 1859a: 319 [F]. Type species: *Penetalebasii* Lacordaire, 1859, by subsequent designation (R. Lucas 1920: 492). Status: valid genus in Phrenapatinae: Penetini.

*Pengalenganus* Pic, 1917d: 10 [M]. Type species: *Pengalenganusinaequalis* Pic, 1917, by monotypy. Status: valid genus in Lagriinae: Goniaderini.

*Penichrus* Champion, 1885: 134 [M]. Type species: *Penichrusblapstinoides* Champion, 1885, by monotypy. Status: valid genus in Tenebrioninae: incertae sedis. Note: placed in Tenebrioninae incertae sedis by Johnston et al. (2020: 771).

*Pentaphyllus* Dejean, 1821: 68 [M]. Type species: *Mycetophagustestaceus* Hellwig, 1792, by monotypy. Status: valid genus in Diaperinae: Diaperini: Diaperina.

*Penthicinus* Reitter, 1896b: 161 [M]. Type species: *Penthicinuskoltzei* Reitter, 1896, by subsequent designation (Gebien 1939: 458). Status: valid genus in Blaptinae: Opatrini: Opatrina. Note: Reitter (1896b) used two different spellings for this genus, including *Penticinus* (Reitter 1896b: 171); subsequently, Reitter (1904: 135, 170) used *Penthicinus* as the valid name of the genus and so acted as First Reviser (ICZN 1999: Article 24.2.4).

*Penthicoides* Fairmaire, 1896a: 20 [M]. Type species: *Penthicoidesseriatoporus* Fairmaire, 1896, by monotypy. Status: valid genus in Blaptinae: Platynotini: Platynotina.

*Penthicomelanesthes* Bogatchev, 1972: 631 [F]. Type species: *Heliophilusgibbulus* Faldermann, 1835, by original designation. Status: junior synonym of *Myladion* Reitter, 1887 in Blaptinae: Opatrini: Opatrina. Synonymy: G.S. Medvedev (1990: 186).

*Penthicus* Faldermann, 1836: 384 [M]. Type species: *Penthicuspinguis* Faldermann, 1836, by subsequent designation (Hope 1841: 126). Status: valid genus and subgenus in Blaptinae: Opatrini: Opatrina.

*Pentholasius* Reitter, 1904: 178 [M]. Type species: *Halonomusvariolatus* Allard, 1884, by monotypy. Status: junior synonym of *Mesomorphus* Miedel, 1880 in Blaptinae: Opatrini: Opatrina. Synonymy: Ferrer (2002: 376).

*Penthomegus* Reitter, 1904: 161 [M]. Type species: *Penthicuscorpulentus* Reitter, 1896, by subsequent designation (Iwan and Löbl 2007: 734). Status: junior synonym of *Penthicus* Faldermann, 1836 in Blaptinae: Opatrini: Opatrina. Synonymy: Gebien (1939: 459, with *Lobodera* Mulsant & Rey, 1859, a junior synonym of *Penthicus* Faldermann, 1836).

*Perdicus* Fairmaire, 1899c: 386 [M]. Type species: *Perdicusantrophilus* Fairmaire, 1899, by monotypy. Status: valid genus in Pimeliinae: Stenosini: Stenosina.

*Perdistretus* Koch, 1953d: 65 [M]. Type species: *Distretusvilhenai* Koch, 1953, by original designation. Status: valid subgenus of *Distretus* Haag-Rutenberg, 1871 in Pimeliinae: Sepidiini: Molurina.

*Periatrum* Sharp, 1886: 407 [N]. Type species: *Periatrumhelmsi* Sharp, 1886, by monotypy. Status: valid genus in Lagriinae: Adeliini.

*Periblaps* Bauer, 1921: 32 [F]. Type species: none designated. Status: undetermined taxon in Blaptinae: Blaptini: Blaptina. Note: this genus was described before 1931 (ICZN 1999, Article 12.1); however, we could not find any nominal species that were subsequently and expressly included in *Periblaps* and therefore no “originally included nominal species” could be used to fix the type species (ICZN 1999, Article 67.2.2).

*Perichilus* Quedenfeldt, 1885: 18 [M]. Type species: *Perichilusbrevicornis* Quedenfeldt, 1885, by monotypy. Status: valid genus in Stenochiinae: Cnodalonini.

*Periloma* Gebien, 1938b: 100 [N]. Type species: *Perilomaalfkeni* Gebien, 1938, by monotypy. Status: valid genus in Blaptinae: Opatrini: Stizopodina.

*Peringueyia* Koch, 1958: 44 [F]. Type species: *Echinotusdispar* Péringuey, 1899, by monotypy. Status: valid genus in Pimeliinae: Sepidiini: Sepidiina.

*Periphanes* Fairmaire, 1882a: 245 [M]. Type species: *Periphanesorichalceus* Fairmaire, 1882, by monotypy. Status: senior synonym of *Periphanodes* Gebien, 1943 in Stenochiinae: Cnodalonini. Note: junior homonym of *Periphanes* Hübner, 1821 [Lepidoptera].

*Periphanodes* Gebien, 1943: 902 [M]. Type species [automatic]: *Periphanesorichalceus* Fairmaire, 1882, by monotypy. Status: valid genus in Stenochiinae: Cnodalonini. Note: replacement name for *Periphanes* Fairmaire, 1882; **new placement** [OM], previously included in Tenebrioninae: Helopini.

*Peristeptus* Haag-Rutenberg, 1875b: 3, 24 [M]. Type species: *Pogonobasislaevigata* Gerstaecker, 1854, by subsequent designation (R. Lucas 1920: 495). Status: junior synonym of *Pogonobasis* Solier, 1837 in Pimeliinae: Adelostomini. Synonymy: Koch (1952b: 17).

*Perithrix* Fairmaire, 1879a: 193 [F]. Type species: *Perithrixgranidorsis* Fairmaire, 1879, by monotypy. Status: valid genus in Blaptinae: Opatrini: Ammobiina.

*Pescennius* Champion, 1884: 3 [M]. Type species: *Pescenniusvillosus* Champion, 1884, by monotypy. Status: valid genus in Pimeliinae: Edrotini.

*Petria* Semenov, 1894: 611 [F]. Type species: *Petriatachyptera* Semenov, 1894, by subsequent designation (R. Lucas 1920: 496). Status: valid genus in Alleculinae: Cteniopodini.

*Petrostetha* Novák, 2008a: 212 [F]. Type species: *Petrostethatibialis* Novák, 2008, by original designation. Status: valid genus in Alleculinae: Alleculini: Alleculina.

*Peyerimhoffius* Koch, 1948: 420 [M]. Type species: *Phylaxplicatus* P.H. Lucas, 1846, by original designation. Status: valid genus in Blaptinae: Dendarini: Melambiina.

*Peyrierasia* Dajoz, 1975b: 113, 114 [F]. Type species: *Peyrierasiasechellensis* Dajoz, 1975, by original designation. Status: valid genus in Diaperinae: Gnathidiini: Anopidiina.

*Pezodontus* Dejean, 1834: 203 [M]. Type species [automatic]: *Odontopuscostatus* Silbermann, 1833, by subsequent designation (Hope 1841: 126). Status: valid genus in Lagriinae: Pycnocerini. Note: replacement name for *Odontopus* Silbermann, 1833 (see Bousquet and Bouchard 2013a: 57).

*Pezohelaeus* Gebien, 1921a: 282 [M]. Type species: *Pterohelaeushirtus* W.J. MacLeay, 1888, by monotypy. Status: junior synonym of *Pterohelaeus* Brême, 1842 in Tenebrioninae: Heleini: Heleina. Synonymy: Carter (1927: 230).

*Pezomaia* Kulzer, 1952: 752 [F]. Type species: *Pezomaiafemoralis* Kulzer, 1952, by original designation. Status: valid genus in Stenochiinae: Cnodalonini.

*Pezophenus* Gebien, 1921a: 325, 339 [M]. Type species: *Pezophenusrutilans* Gebien, 1921 (= *Prophanessubmetallicus* W.J. MacLeay, 1887), by monotypy. Status: valid genus in Stenochiinae: Cnodalonini.

*Phaedeucyrtus* Pic, 1916e: 14 [M]. Type species: *Phaedeucyrtusobscuripes* Pic, 1916, by monotypy. Status: junior synonym of *Phaedis* Pascoe, 1866 in Stenochiinae: Cnodalonini. Synonymy: Ando (2016: 70).

*Phaedis* Pascoe, 1866a: 474 [M]. Type species: *Phaediselysius* Pascoe, 1866, by monotypy. Status: valid genus in Stenochiinae: Cnodalonini.

*Phaedogria* Borchmann, 1936: 16, 57 [F]. Type species: *Lagriaionoptera* Erichson, 1834, by original designation. Status: valid genus in Lagriinae: Lagriini: Lagriina.

*Phaenogeton* Bremer, 2016: 220 [M]. Type species: *Amarygmusvaricolor* Gebien, 1921, by original designation. Status: valid subgenus of *Amarygmus* Dalman, 1823 in Tenebrioninae: Amarygmini.

*Phaeostolus* Fairmaire, 1884c: cxlvi [M]. Type species: *Phaeostolusgrandicornis* Fairmaire, 1884, by monotypy. Status: valid genus in Tenebrioninae: Praeugenini. Note: redescribed as new by Fairmaire (1887b: 293).

*Phaeotribon* Kraatz, 1865: 81, 242 [M]. Type species: *Phaeotribonpulchellus* Kraatz, 1865, by monotypy. Status: valid genus in Pimeliinae: Tentyriini.

*Phaleria* Latreille, 1802: 162 [F]. Type species: *Tenebriocadaverinus* Fabricius, 1792, by plenary powers (ICZN 1975, Opinion 1039). Status: valid genus and subgenus in Diaperinae: Phaleriini. Note: placed on the Official List of Generic Names in Zoology (ICZN 1975, Opinion 1039).

*Phaleriderma* Koch, 1954a: 25 [N]. Type species: *Oncotusobscuricollis* Solier, 1848, by original designation. Status: valid genus in Blaptinae: Platynotini: Eurynotina.

*Phalerisida* Kulzer, 1959: 563 [F]. Type species: *Phalerisidamaculata* Kulzer, 1959, by original designation. Status: junior synonym of *Phaleria* Latreille, 1802 in Diaperinae: Phaleriini. Synonymy: Triplehorn (1991: 258).

*Phaleromela* Reitter, 1916b: 4 [F]. Type species: *Phaleriasubhumeralis* Marseul, 1876, by monotypy. Status: valid genus in Diaperinae: Phaleriini.

*Phallocentrion* Koch, 1956a: 166 [N]. Type species: *Selinusedentatus* Gebien, 1914, by original designation. Status: valid genus in Blaptinae: Platynotini: Platynotina.

*Phanechloros* Matthews & Bouchard, 2008: 300 [M]. Type species [automatic]: *Platyphanespunctipennis* Carter, 1911, by original designation. Status: valid genus in Tenebrioninae: Heleini: Cyphaleina. Note: replacement name for *Chlorophanes* Matthews, 1992.

*Phanerops* Solier, 1851: 233 [M]. Type species: *Phaneropselongatus* Solier, 1851, by monotypy. Status: valid genus in Tenebrioninae: Tenebrionini.

*Phanerotoma* Solier, 1843: 82, 126 [F]. Type species: *Phanerotomaelongata* Solier, 1843 (= *Pimelialaevigata* G.-A. Olivier, 1795), by original designation. Status: senior synonym of *Ocnodes* Fåhraeus, 1870 in Pimeliinae: Sepidiini: Molurina. Synonymy: Gebien (1937a: 759), Kamiński et al. (2019b: 55). Note: junior homonym of *Phanerotoma* Wesmael, 1838 [Hymenoptera].

*Phanerotomea* Koch, 1958: 58 [F]. Type species [automatic]: *Phanerotomaelongata* Solier, 1843 (= *Pimelialaevigata* G.-A. Olivier, 1795), by original designation. Status: junior synonym of *Ocnodes* Fåhraeus, 1870 in Pimeliinae: Sepidiini: Molurina. Note: replacement name for *Phanerotoma* Solier, 1843.

*Phayllidius* Gebien, 1922a: 451 [M]. Type species: *Phayllidiusdispar* Gebien, 1922, by monotypy. Status: junior synonym of *Ulomoides* Blackburn, 1888 in Diaperinae: Diaperini: Diaperina. Synonymy: Grimm (2018: 103).

*Phayllus* Champion, 1886: 167 [M]. Type species: *Phayllusminutus* Champion, 1886, by monotypy. Status: valid genus in Diaperinae: Diaperini: Diaperina.

*Phediodes* Campbell, 1976: 26 [M]. Type species: *Phediodesapterus* Campbell, 1976, by original designation. Status: valid genus in Alleculinae: Alleculini: Alleculina.

*Phedius* Champion, 1888: 447 [M]. Type species: *Phediuschevrolati* Champion, 1888, by subsequent designation (R. Lucas 1920: 500). Status: valid genus in Alleculinae: Alleculini: Alleculina.

*Phegoneus* Casey, 1907: 380, 426 [M]. Type species: *Epitragodesjulichi* Casey, 1891, by original designation. Status: valid genus and subgenus in Pimeliinae: Epitragini.

*Phellidius* J.L. LeConte, 1862: 236 [M]. Type species: *Bolitophaguscornutus* Fabricius, 1801, by original designation. Status: junior synonym of *Bolitotherus* Candèze, 1861 in Tenebrioninae: Bolitophagini. Synonymy: J.L. LeConte (1866a: 62). Note: the younger species name *Bolitophaguscornutus* Fabricius, 1801 was given priority over the older synonym *Opatrumbifurcum* Fabricius, 1798 by the ICZN (2019, Opinion 2438).

*Pheloneis* Pascoe, 1866a: 483 [M]. Type species: *Adeliumharpaloides* White, 1846 (= *Adeliumamaroides* Bates, 1874), by original designation. Status: valid genus in Lagriinae: Adeliini.

*Phelopatrum* Marseul, 1876: 100 [N]. Type species: *Hadrusscaphoides* Marseul, 1876, by monotypy. Status: valid genus in Blaptinae: Opatrini: Opatrina.

*Phenus* Gebien, 1921a: 324, 325 [M]. Type species: *Phenuslatitarsis* Gebien, 1921, by original designation. Status: valid genus in Stenochiinae: Cnodalonini.

*Pheres* Champion, 1886: 150 [M]. Type species: *Pheresbatesi* Champion, 1886, by monotypy. Status: valid genus in Tenebrioninae: Ulomini.

*Pheugonius* Fairmaire, 1899b: 313 [M]. Type species: *Pheugoniusborneensis* Fairmaire, 1899, by monotypy. Status: valid genus in Lagriinae: Pycnocerini.

*Phibalus* Gistel, 1856: 384 [M]. Type species: *Chrysomelapubescens* Linnaeus, 1767, by monotypy. Status: valid subgenus of *Omophlus* Dejean, 1834 in Alleculinae: Cteniopodini.

*Philhamellus* Kaszab, 1962b: 83, 84 [M]. Type species: *Philhammusmyrmecophilus* Kaszab, 1960, by original designation. Status: valid subgenus of *Philhammus* Fairmaire, 1871 in Pimeliinae: Cnemeplatiini: Cnemeplatiina.

*Philhammus* Fairmaire, 1871a: 393 [M]. Type species: *Philhammussericans* Fairmaire, 1871, by monotypy. Status: valid genus and subgenus in Pimeliinae: Cnemeplatiini: Cnemeplatiina.

*Philolithus* Lacordaire in J.L. LeConte, 1858b: 18 [M]. Type species: *Pelecyphoruscarinatus* J.L. LeConte, 1851, by subsequent designation (Casey 1912: 79). Status: valid genus and subgenus in Pimeliinae: Asidini.

*Philorea* Erichson, 1834: 242 [F]. Type species: *Philoreapicipes* Erichson, 1834, by monotypy. Status: valid genus in Pimeliinae: Physogasterini.

*Phligra* Laporte, 1840: 197 [F]. Type species: *Sepidium degeeri* Laporte, 1840 (= *Tenebriocristatus* DeGeer, 1778), by monotypy. Status: junior synonym of *Cyrtoderes* Dejean, 1834 in Pimeliinae: Sepidiini: Trachynotina. Synonymy: Lacordaire (1859a: 202).

*Phobelius* Blanchard, 1842: pl. 14 [M]. Type species: *Phobeliuscrenatus* Blanchard, 1842, by monotypy. Status: valid genus in Lagriinae: Goniaderini. Note: see Bouchard and Bousquet (2020a: 99) for comments about the date of publication of this genus.

*Phrenapates* Gray in Griffith and Pidgeon, 1831: pl. 50 [M]. Type species: *Phrenapatesbennettii* Gray, 1831, by monotypy. Status: valid genus in Phrenapatinae: Phrenapatini.

*Phrynocarenum* Gebien, 1928: 106 [N]. Type species: *Phrynocarenumbruchianum* Gebien, 1928 (= *Emmalloderastrangulata* Fairmaire, 1905), by monotypy. Status: valid genus in Pimeliinae: Phrynocarenini.

*Phrynocolopsis* Koch, 1951: 93 [F]. Type species: *Phrynocolusfrondosus* Gerstaecker, 1871, by original designation. Status: valid subgenus of *Phrynocolus* Lacordaire, 1859 in Pimeliinae: Sepidiini: Molurina.

*Phrynocolus* Lacordaire, 1859a: 201 [M]. Type species [automatic]: *Cryptogeniusdentatus* Solier, 1843, by original designation. Status: valid genus and subgenus in Pimeliinae: Sepidiini: Molurina. Note: replacement name for *Cryptogenius* Solier, 1843.

*Phrynophanes* Koch, 1951: 92 [M]. Type species: *Molurisgredleri* Haag-Rutenberg, 1877, by original designation. Status: valid genus in Pimeliinae: Sepidiini: Molurina.

*Phthora* Champion, 1893a: 531 [F]. Type species [automatic]: *Phtoracrenata* Mulsant, 1854 (= *Clamorisinsurgens* Gozis, 1886), by monotypy. Status: junior synonym of *Clamoris* Gozis, 1886 in Phrenapatinae: Penetini. Note: unjustified emendation of *Phtora* Mulsant, 1854, not in prevailing usage; junior homonym of *Phthora* Gemminger, 1870 [Coleoptera: Tenebrionidae: Diaperinae: Phaleriini].

*Phthora* Gemminger in Gemminger and Harold, 1870: 1959 [F]. Type species [automatic]: *Phtoracrenata* Germar, 1836, by monotypy. Status: junior synonym of *Phtora* Germar, 1836 in Diaperinae: Phaleriini. Note: unjustified emendation of *Phtora* Germar, 1836, not in prevailing usage.

*Phtora* Germar, 1836: pl. 11 [F]. Type species: *Phtoracrenata* Germar, 1836, by monotypy. Status: valid genus and subgenus in Diaperinae: Phaleriini. Note: the alternative original spelling *Phthora*, used by Germar (1836: explanation for pl. 11), was rejected by Neave (1940: 736) who acted as First Reviser.

*Phtora* Mulsant, 1854: 228 [F]. Type species: *Phtoracrenata* Mulsant, 1854 (= *Clamorisinsurgens* Gozis, 1886), by monotypy. Status: senior synonym of *Clamoris* Gozis, 1886 in Phrenapatinae: Penetini. Note: junior homonym of *Phtora* Germar, 1836 [Coleoptera: Tenebrionidae: Diaperinae: Phaleriini].

*Phygoscotus* Schulz, 1902: 134 [M]. Type species [automatic]: *Spheniscuserotyloides* W. Kirby, 1819, by monotypy. Status: junior synonym of *Cuphotes* Champion, 1887 in Stenochiinae: Stenochiini. Note: replacement name for *Spheniscus* W. Kirby, 1819.

*Phylacastus* Fairmaire, 1897f: 116 [M]. Type species: *Phylacastusstriolatus* Fairmaire, 1897, by monotypy. Status: valid genus in Blaptinae: Platynotini: Eurynotina.

*Phylacinus* Fairmaire, 1896b: 349 [M]. Type species: *Phylacinusasperipennis* Fairmaire, 1896, by monotypy. Status: valid genus in Blaptinae: Dendarini: Dendarina.

*Phylan* Sturm, 1826: 23 [M]. Type species: *Opatrumgibbum* Fabricius, 1775, by subsequent monotypy (Steven 1828: 99). Status: valid genus and subgenus in Blaptinae: Dendarini: Dendarina. Note: originally proposed without included nominal species; Steven (1828: 99), by including the species *Opatrumgibbum* Fabricius, 1775 in association with this name, was the first author to subsequently and expressly include nominal species in *Phylan* (ICZN 1999, Article 67.2.2).

*Phylanmania* Ferrer, 2013: 38 [F]. Type species: *Phylan ilerdensis* Español & Viñolas, 1981, by original designation. Status: valid genus in Blaptinae: Dendarini: Dendarina.

*Phylax* Brullé, 1832: 209 [M]. Type species [automatic]: *Opatrumgibbum* Fabricius, 1775, by subsequent monotypy (Steven 1828: 99). Status: junior synonym of *Phylan* Sturm, 1826 in Blaptinae: Dendarini: Dendarina. Note: unnecessary replacement name of *Phylan* Sturm, 1826 (see Bouchard and Bousquet 2020a: 100).

*Phylaximon* Koch, 1948: 414 [M]. Type species: *Opatrumvariolosum* G.-A. Olivier, 1812, by original designation. Status: valid subgenus of *Allophylax* Bedel, 1906 in Blaptinae: Dendarini: Melambiina.

*Phyletes* Redtenbacher, 1845: 128 [M]. Type species: *Phyletespopuli* Chevrolat, 1847 (= *Diaperisbifasciata* Say, 1824), by subsequent monotypy (Chevrolat 1847b: 57). Status: junior synonym of *Alphitophagus* Stephens, 1832 in Diaperinae: Diaperini: Adelinina. Synonymy: Heyden et al. (1883: 133). Note: genus originally proposed without included nominal species; Chevrolat (1847b: 57) included the species “*Phylethuspopuli* Még.” as a synonym of *Alphitophagusquadripustulatus* Stephens, 1832 in the genus “*Phylethus*” and Redtenbacher (1848: 589) used “*Phylethuspopuli*” as valid subsequently; therefore, the author of the type species dates back to its first publication as a synonym (ICZN 1999, Article 11.6.1).

*Phyllechus* Bouchard & Bousquet, **new genus** [M]. Type species: *Phyllechusboettcheri* Kulzer, 1966, by **present designation**. Status: valid genus in Stenochiinae: Stenochiini. Note: Kulzer (1966: 374) introduced the new genus name *Phyllechus* for two new species, but unfortunately did not designate a type species; the genus *Phyllechus*, which has been treated as valid since 1966, is therefore unavailable (ICZN 1999, Article 13.3); we hereby make the name available by selecting *Phyllechusboettcheri* Kulzer, 1966 as type species and referring to Kulzer (1966: 374) for the character states that characterise and differentiate *Phyllechus*.

*Phyloradix* Endrödy-Younga, 1996: 15, 16 [M]. Type species: *Caenocrypticussoror* Endrödy-Younga, 1996, by original designation. Status: valid subgenus of *Caenocrypticus* Gebien, 1920 in Pimeliinae: Caenocrypticini.

*Phymaeus* Pascoe, 1883: 439 [M]. Type species: *Phymaeuspustulosus* Pascoe, 1883, by monotypy. Status: valid genus in Stenochiinae: Cnodalonini.

*Phymatestes* Pascoe, 1866b: 142 [M]. Type species [automatic]: *Lagriatuberculata* Fabricius, 1787, by monotypy (see ICZN 1989, Opinion 1525). Status: valid genus in Lagriinae: Goniaderini. Note: replacement name for *Phymatodes* Dejean, 1834; placed on the Official List of Generic Names in Zoology by the ICZN (1989, Opinion 1525).

*Phymatiotris* Solier, 1836: 63 [F]. Type species: *Tentyriaquadricollis* Brullé, 1832, by subsequent designation (Hope 1841: 118). Status: valid genus in Pimeliinae: Pimeliini.

*Phymatium* Billberg, 1820: 31 [N]. Type species: *Pimeliamaculata* Fabricius, 1781, by **present designation**. Status: senior synonym of *Cryptochile* Latreille, 1828 in Pimeliinae: Cryptochilini: Calognathina. Synonymy: **new synonym** [PB]. Note: discovery of this forgotten name threatens the stability of the junior objective synonym *Cryptochile* Latreille, 1828; although *Phymatium* Billberg, 1820 has not been used as valid in the literature after 1899, we could not find usage of *Cryptochile* Latreille, 1828 in at least 25 works, published by at least ten authors in the immediately preceding 50 years and therefore reversal of precedence cannot be used to treat *Phymatium* Billberg, 1820 as a nomen oblitum; we recommend that an application be submitted to the International Commission on Zoological Nomenclature to conserve usage of *Cryptochile* Latreille, 1828, the type genus of the valid tribe Cryptochilini and the subtribe Cryptochilina.

*Phymatodes* Dejean, 1834: 203 [M]. Type species: *Lagriatuberculata* Fabricius, 1787, by monotypy. Status: senior synonym of *Phymatestes* Pascoe, 1866 in Lagriinae: Goniaderini. Note: name suppressed for the purposes of the Principle of Homonymy and the Principle of Priority by the ICZN (1989, Opinion 1525).

*Phymatoplata* Koch, 1956a: 269 [F]. Type species: *Selinusasperulus* Fairmaire, 1897, by monotypy. Status: valid genus in Blaptinae: Platynotini: Platynotina.

*Phymatosoma* Laporte & Brullé, 1831: 332, 408 [N]. Type species: *Phymatosomatuberculatum* Laporte & Brullé, 1831, by monotypy. Status: valid genus in Stenochiinae: Stenochiini. Note: unjustified emendation of the original spelling *Phymatisoma*, introduced by Agassiz (1846b: 290), in prevailing usage and treated as a justified emendation (ICZN 1999, Article 33.2.3.1).

*Physadesmia* Penrith, 1979: 7, 35 [F]. Type species: *Adesmiaglobosa* Haag-Rutenberg, 1875, by original designation. Status: valid genus in Pimeliinae: Adesmiini.

*Physciolagria* Pic, 1930c: 7 [F]. Type species: *Physciolagrialiturata* Pic, 1930, by monotypy. Status: valid genus in Tenebrionidae: incertae sedis. Note: we could not assign this genus to any particular group in Tenebrionidae based on the description.

*Physignathus* Gistel, 1834: 22 [M]. Type species: *Helopsundatus* Fabricius, 1792 (= *Erotylusnebulosus* Fabricius, 1781), by monotypy. Status: junior synonym of *Cymatothes* Dejean, 1834 Tenebrioninae: Amarygmini. Synonymy: Bousquet and Bouchard (2017: 132). Note: junior homonym of *Physignathus* Cuvier, 1829 [Reptilia].

*Physocoelus* Haldeman, 1850: 347 [M]. Type species: *Psorodesinflata* Solier, 1848 (= *Helopscontractus* Palisot de Beauvois, 1812), by monotypy. Status: junior synonym of *Meracantha* W. Kirby, 1837 in Tenebrioninae: Amarygmini. Synonymy: Schaum (1850: 181, through synonymy of the type species).

*Physodera* Solier, 1843: 78, 125 [F]. Type species: *Pimeliagibba* Fabricius, 1787, by original designation. Status: junior synonym of *Moluris* Latreille, 1802 in Pimeliinae: Sepidiini: Molurina. Synonymy: Lacordaire (1859a: 194). Note: junior homonym of *Physodera* Eschscholtz, 1829 [Coleoptera: Carabidae].

*Physogaster* Lacordaire, 1830a: 276 [F]. Type species: *Physogastermendocina* Lacordaire, 1830, by monotypy. Status: valid genus in Pimeliinae: Physogasterini.

*Physogasterinus* Kaszab, 1981a: 79 [M]. Type species: *Physogasterinuslanuginosus* Kaszab, 1981, by original designation. Status: valid genus in Pimeliinae: Physogasterini.

*Physogria* Borchmann, 1916a: 48, 108 [F]. Type species: *Lagriagibbosa* Kolbe, 1901, by monotypy. Status: valid genus in Lagriinae: Lagriini: Lagriina.

*Physohelops* Schuster, 1937: 50 [M]. Type species: *Physohelopsfreyi* Schuster, 1937, by monotypy. Status: valid genus in Tenebrioninae: Helopini: Helopina.

*Physolagria* Fairmaire, 1891g: 114 [F]. Type species: *Physolagriamolleri* Fairmaire, 1891, by monotypy. Status: valid genus in Lagriinae: Lagriini: Lagriina.

*Physophrynus* Fairmaire, 1882c: l [M]. Type species: *Physophrynusburdoi* Fairmaire, 1882, by monotypy. Status: valid genus in Pimeliinae: Sepidiini: Molurina.

*Physosterna* Dejean, 1834: 179 [F]. Type species: *Pimeliaovata* G.-A. Olivier, 1795 (= *Tenebriotorulosus* Pallas, 1781), by monotypy. Status: valid subgenus of *Adesmia* Fischer, 1822 in Pimeliinae: Adesmiini.

*Phytolostoma* Koch, 1952b: 34 [N]. Type species: *Phytolostomalimpopoana* Koch, 1952, by original designation. Status: valid genus in Pimeliinae: Adelostomini.

*Phitophilus* Guérin-Méneville, 1831a: pl. 4 [M]. Type species: *Phitophilushelopioides* Guérin-Méneville, 1831, by monotypy. Status: valid genus in Pimeliinae: Epitragini.

*Piccula* Bousquet & Bouchard in Bousquet et al., 2015: 137 [F]. Type species [automatic]: *Gerardiasublineata* Pic, 1954, by monotypy. Status: valid genus in Alleculinae: Alleculini: Gonoderina. Note: replacement name for *Gerardia* Pic, 1954.

*Piciella* Borchmann, 1936: 237, 435 [F]. Type species: *Piciellahelopioides* Borchmann, 1936, by original designation. Status: valid genus in Lagriinae: Lagriini: Statirina.

*Picnotagalus* Kaszab, 1939a: 102 [M]. Type species: *Picnotagalushorni* Kaszab, 1939, by original designation. Status: junior synonym of *Scolytocaulus* Fairmaire, 1896 in Phrenapatinae: Penetini. Synonymy: Schawaller (1999b: 422).

*Picocamaria* Masumoto, 1993b: 226, 232 [F]. Type species: *Camariageniculata* Pic, 1915, by original designation. Status: valid genus in Stenochiinae: Cnodalonini.

*Piesomera* Solier, 1843: 77 [F]. Type species: *Pimeliascabra* Fabricius, 1775, by monotypy. Status: junior synonym of *Psammodes* W. Kirby, 1819 in Pimeliinae: Sepidiini: Molurina. Synonymy: Gebien (1937a: 759).

*Piesterotarsa* Sénac, 1884: 8 [F]. Type species [automatic]: *Pimeliagigantea* Fischer, 1820, by subsequent designation (Gebien 1937a: 840). Status: junior synonym of *Pisterotarsa* Motschulsky, 1860 in Pimeliinae: Pimeliini. Note: unjustified emendation of *Pisterotarsa* Motschulsky, 1860, not in prevailing usage.

*Piestognathoides* Kaszab, 1981b: 305 [M]. Type species: *Piestognathoidesbahrainicus* Kaszab, 1981, by original designation. Status: valid genus in Pimeliinae: Erodiini.

*Piestognathus* P.H. Lucas, 1858: clxxxviii [M]. Type species: *Piestognathusdouei* P.H. Lucas, 1858, by monotypy. Status: valid genus in Pimeliinae: Erodiini.

*Pigeocaulinus* Kaszab, 1984: 355, 388 [M]. Type species: *Pigeocaulinussumatranus* Kaszab, 1984 (= *Leprocaulinuskrikkeni* Kaszab, 1982), by original designation. Status: junior synonym of *Leprocaulinus* Kaszab, 1982 in Stenochiinae: Cnodalonini. Synonymy: Masumoto (2003: 60); see notes in the entry for *Leprocaulinus* Kaszab, 1982 regarding the validity of this older name.

*Pigeostrongylium* Kaszab, 1984: 355, 385 [N]. Type species: *Pigeostrongyliumkedahense* Kaszab, 1984, by original designation. Status: valid genus in Stenochiinae: Cnodalonini.

*Pigeus* Gebien, 1919: 28, 153 [M]. Type species: *Camarimenanitidipes* Fairmaire, 1893, by original designation. Status: valid genus in Stenochiinae: Cnodalonini.

*Pilioloba* Erichson in Agassiz, 1846a: 144 [F]. Type species: *Salaxlacordairii* Guérin-Méneville, 1834, by monotypy. Status: junior synonym of *Salax* Guérin-Méneville, 1834 in Pimeliinae: Trilobocarini. Synonymy: Erichson in Agassiz (1846a: 144). Note: the name *Pilioloba* was listed as synonym of *Salax* Guérin-Méneville, 1834 by Erichson in Agassiz (1846a: 144), it was treated before 1961 as an available name and adopted as the name of a taxon (e.g., Cazurro Ruiz 1894b: 451), *Pilioloba* was therefore made available from its first publication as a synonym (ICZN 1999, Article 11.6.1).

*Pilobalia* Burmeister, 1875: 487 [F]. Type species: *Nycteliadecorata* Erichson, 1834, by subsequent designation (Rye 1877: 338). Status: valid genus in Pimeliinae: Nycteliini.

*Pilobaloderes* Kulzer, 1958b: 192 [M]. Type species: *Pilobaloderesgebieni* Kulzer, 1958, by original designation. Status: valid genus in Pimeliinae: Praociini.

*Pilosocasnonidea* Pic, 1934a: 31 [F]. Type species: *Nemostirabipartita* Pic, 1911, by monotypy. Status: valid subgenus of *Casnonidea* Fairmaire, 1882 in Lagriinae: Lagriini: Statirina.

*Pilosoplonyx* Bremer, 2014a: 37 [M]. Type species: *Plesiophthalmusbremeri* Masumoto, 1999, by original designation. Status: valid genus in Tenebrioninae: Amarygmini.

*Piloxys* Fairmaire, 1895a: 23 [M]. Type species: *Piloxysfoveatus* Fairmaire, 1895, by monotypy. Status: valid genus in Stenochiinae: Cnodalonini.

*Pimalius* Casey, 1907: 367 [M]. Type species: *Trimytispulverea* Horn, 1870, by original designation. Status: junior synonym of *Trimytis* J.L. LeConte, 1851 in Pimeliinae: Edrotini. Synonymy: MacLachlan and Olson (1990: 79).

*Pimelia* Fabricius, 1775: 251 [F]. Type species: *Pimeliaangulata* Fabricius, 1775, by subsequent designation (Hope 1841: 118). Status: valid genus and subgenus in Pimeliinae: Pimeliini.

*Pimeliocnera* Reitter, 1909b: 310 [F]. Type species: *Pimeliocneragebieni* Reitter, 1909, by monotypy. Status: valid genus in Pimeliinae: Pimeliini.

*Pimelionotus* Ardoin, 1963a: 86 [M]. Type species: *Psorodeslugens* Fåhraeus, 1870, by monotypy. Status: valid genus in Tenebrioninae: Amarygmini.

*Pimeliopsis* Champion, 1892: 477 [F]. Type species: *Pimeliopsisgranulata* Champion, 1892, by monotypy. Status: valid genus in Pimeliinae: Edrotini.

*Pimelipachys* Skopin, 1962: 232 [M]. Type species: *Pachyscelislaevicollis* Reitter, 1893, by original designation. Status: valid genus in Pimeliinae: Pimeliini.

*Pimelosomus* Burmeister, 1875: 488, 489 [M]. Type species: *Pimelosomussphaericus* Burmeister, 1875, by monotypy. Status: valid genus in Pimeliinae: Physogasterini.

*Pimidia* Rafinesque, 1815: 113 [F]. Type species [automatic]: *Pimeliaangulata* Fabricius, 1775, by subsequent designation (Hope 1841: 118). Status: junior synonym of *Pimelia* Fabricius, 1775 in Pimeliinae: Pimeliini. Note: unjustified emendation of *Pimelia* Fabricius, 1775, not in prevailing usage.

*Pimplema* Pascoe, 1887: 16 [F]. Type species: *Pimplemaampliata* Pascoe, 1887 (= *Platydemahemisphaerica* Laporte & Brullé, 1831), by monotypy. Status: valid genus in Diaperinae: Leiochrinini.

*Piscicula* Robiche, 2004a: 736 [F]. Type species: *Pisciculasprecherae* Robiche, 2004, by monotypy. Status: valid genus in Blaptinae: Pedinini: Helopinina.

*Pisterotarsa* Motschulsky, 1860c: 532 [F]. Type species: *Pimeliagigantea* Fischer, 1820, by subsequent designation (Gebien 1937a: 840). Status: valid genus in Pimeliinae: Pimeliini.

*Pitholaus* Champion, 1888: 446 [M]. Type species: *Pitholaushelopioides* Champion, 1888, by monotypy. Status: valid genus in Alleculinae: Alleculini: Alleculina.

*Pizura* Novák, 2016b: 436 [F]. Type species: *Pizurabarbucha* Novák, 2016, by original designation. Status: valid genus in Alleculinae: Alleculini: Alleculina.

*Plamius* Fairmaire, 1896a: 30 [M]. Type species: *Plamiustenuestriatus* Fairmaire, 1896, by monotypy. Status: valid genus in Stenochiinae: Cnodalonini.

*Planasida* Escalera, 1907: 337 [F]. Type species: *Asidapaulinoi* Pérez Arcas, 1868, by subsequent designation (Viñolas and Cartagena 2005: 223). Status: junior synonym of *Gracilasida* Escalera, 1905 in Pimeliinae: Asidini. Synonymy: F. Soldati (2008: 137). Note: *Planasida* Escalera, 1907 was used as valid in the literature recently (e.g., F. Soldati 2008: 137); however, the name *Gracilasida* Escalera, 1905 is older and should be used as valid (e.g., F. Soldati 2020: 152–153).

*Planibates* Kaszab, 1939b: 221 [M]. Type species: *Planibatespapuanus* Kaszab, 1939, by monotypy. Status: junior synonym of *Bradymerus* Perroud & Montrouzier, 1865 in Stenochiinae: Cnodalonini. Synonymy: Schawaller (2013b: 163).

*Planirostrosis* Penrith, 1977: 18, 172 [F]. Type species: *Zophosishimba* Koch, 1958, by original designation. Status: valid subgenus of *Zophosis* Latreille, 1802 in Pimeliinae: Zophosini.

*Planodes* Mulsant & Rey, 1859c: 94, 117 [M]. Type species: *Planodesbyrroides* Mulsant & Rey, 1859, by subsequent designation (R. Lucas 1920: 517). Status: senior synonym of *Planostibes* Gemminger, 1870 in Blaptinae: Opatrini: Stizopodina. Note: junior homonym of *Planodes* Newman, 1842 [Coleoptera: Cerambycidae].

*Planoodescelis* Egorov, 2004: 591 [F]. Type species: *Oodesceliskansouensis* Kaszab, 1940, by original designation. Status: valid subgenus of *Oodescelis* Motschulsky, 1845 in Blaptinae: Platyscelidini.

*Planoplatyscelis* Kaszab, 1940a: 157, 206 [F]. Type species: *Platyscelismargelanica* Kraatz, 1882 (= *Bioramixpamirensis* Bates, 1879), by plenary powers (ICZN 1993a, Opinion 1728). Status: valid subgenus of *Bioramix* Bates, 1879 in Blaptinae: Platyscelidini. Note: placed on the Official List of Generic Names in Zoology (ICZN 1993a, Opinion 1728); the First Reviser (*Planoplatyscelis* Kaszab, 1940 versus *Pleioplatyscelis* Kaszab, 1940) is Egorov (2004: 601).

*Planoprosodes* G.S. Medvedev, 2005b: 90 [M]. Type species: *Prosodesreitteri* Reitter, 1893, by original designation. Status: valid subgenus of *Prosodes* Eschscholtz, 1829 in Blaptinae: Blaptini: Prosodina. Note: originally described as a section within a subgenus.

*Planostibes* Gemminger in Gemminger and Harold, 1870: 1926 [M]. Type species [automatic]: *Planodesbyrroides* Mulsant & Rey, 1859, by subsequent designation (R. Lucas 1920: 517). Status: valid genus in Blaptinae: Opatrini: Stizopodina. Note: replacement name for *Planodes* Mulsant & Rey, 1859.

*Plastica* C.O. Waterhouse, 1903: 563 [F]. Type species: *Plasticapolita* C.O. Waterhouse, 1903, by monotypy. Status: valid genus in Tenebrioninae: Apocryphini.

*Platamodes* Ménétriés, 1849: 233 [M]. Type species: *Platamodesdentipes* Ménétriés, 1849, by monotypy. Status: valid genus in Pimeliinae: Stenosini: Platamodina.

*Platasida* Casey, 1912: 77, 182 [F]. Type species: *Asidaembaphionides* Horn, 1894, by original designation. Status: valid subgenus of *Stenomorpha* Solier, 1836 in Pimeliinae: Asidini.

*Plateia* Laporte, 1840: 215 [F]. Type species: *Plateiaorientalis* Laporte, 1840, by monotypy. Status: junior synonym of *Catapiestus* Perty, 1831 in Stenochiinae: Cnodalonini. Synonymy: Blanchard (1845: 16). Note: junior homonym of *Plateia* Hübner, 1820 [Lepidoptera].

*Platesthes* G.R Waterhouse, 1845b: 317 [F]. Type species: *Platesthessilphoides* G.R. Waterhouse, 1845, by monotypy. Status: valid genus in Pimeliinae: Praociini.

*Platolenes* Gebien, 1914b: 420 [M]. Type species: *Platolenesrufipes* Gebien, 1914, by monotypy. Status: junior synonym of *Amarygmus* Dalman, 1823 in Tenebrioninae: Amarygmini. Synonymy: Bremer (2001a: 69).

*Platyallecula* Blair, 1935b: 266 [F]. Type species: *Cistelabrunnea* C.O. Waterhouse, 1876, by original designation. Status: valid genus in Alleculinae: Alleculini: Alleculina.

*Platyblaps* Motschulsky, 1860c: 531 [F]. Type species: *Blaps holconota* Fischer von Waldheim, 1844, by subsequent designation (Nabozhenko 2008: 36). Status: junior synonym of *Blaps* Fabricius, 1775 in Blaptinae: Blaptini: Blaptina. Synonymy: Gemminger in Gemminger and Harold (1870: 1860)

*Platybolium* Blair, 1938: 222 [N]. Type species: *Platyboliumalvearium* Blair, 1938, by original designation. Status: valid genus in Tenebrioninae: Triboliini.

*Platyburak* Iwan, 1990: 124 [M]. Type species: *Notocoraxnervosus* Mulsant & Rey, 1853, by original designation. Status: valid genus in Blaptinae: Platynotini: Platynotina.

*Platyburmanicus* Iwan, 2003b: 715 [M]. Type species: *Platyburmanicusignotus* Iwan, 2003, by original designation. Status: valid genus in Blaptinae: Platynotini: Platynotina.

*Platycharlesus* Iwan, 1998b: 307 [M]. Type species: *Trigonopusmorosus* Mulsant & Rey, 1853, by original designation. Status: junior synonym of *Schelodontes* Koch, 1956 in Blaptinae: Platynotini: Platynotina. Synonymy: Iwan and Kamiński (2014: 171).

*Platycilibe* Carter, 1911a: 205 [F]. Type species: *Platycilibebrevis* Carter, 1911, by monotypy. Status: junior synonym of *Scolytocaulus* Fairmaire, 1896 in Phrenapatinae: Penetini. Synonymy: Kaszab (1978a: 168).

*Platycolpotus* Iwan, 1997: 255, 266 [M]. Type species: *Platydendarusdendaroides* Kaszab, 1975, by original designation. Status: valid genus in Blaptinae: Platynotini: Platynotina.

*Platycotylus* Olliff, 1883: 182 [M]. Type species: *Platycotylusinusitatus* Olliff, 1883 (= *Ipsaphesnitidulus* W.J. MacLaeay, 1873), by monotypy. Status: valid genus in Tenebrioninae: Palorini. Note: originally described in the family Cucujidae, transferred to Tenebrionidae by Crowson (1955: 103).

*Platycrepis* Lacordaire, 1859b: 418 [F]. Type species: *Platycrepisviolacea* Lacordaire, 1859, by monotypy. Status: valid genus in Stenochiinae: Cnodalonini.

*Platycrypticus* Español, 1955: 9, 17 [M]. Type species: *Crypticusviaticus* Fairmaire, 1851, by original designation. Status: valid subgenus of *Crypticus* Latreille, 1816 in Diaperinae: Crypticini. Note: replacement name for *Ulomoides* Escalera, 1927. Note: Español (1952a: 117) introduced the replacement name *Platycrypticus* earlier; however, the name is unavailable from that date since the author did not fix a type species (ICZN 1999, Article 13.3.1).

†*Platycteniopus* Chang, Nabozhenko, Pu, Xu, Jia & Li, 2016: 291 [M]. Type species: *Platycteniopusdiversoculatus* Chang, Nabozhenko, Pu, Xu, Jia & Li, 2016, by original designation. Status: valid genus in Alleculinae: Cteniopodini. Note: described from Upper Cretaceous deposits (China).

*Platydema* Laporte & Brullé, 1831: 332, 350 [F]. Type species: *Diaperisviolacea* Fabricius, 1790, by subsequent designation (Westwood 1838: 32). Status: valid genus in Diaperinae: Diaperini: Diaperina. Note: see Löbl and Smetana (2010: 34) for comments on the gender of this name.

*Platydemoides* Kaszab, 1980a: 161 [M]. Type species: *Platydemoidesbrincki* Kaszab, 1980, by original designation. Status: valid genus in Diaperinae: Diaperini: Diaperina. Note: *Platydemoides* was used earlier by Kaszab (1979a: 77) without a description, a definition or a bibliographic reference to such a published statement (ICZN 1999, Article 13.1) and is therefore unavailable from that date.

*Platydendarus* Kaszab, 1975b: 281, 312 [M]. Type species: *Opatrumjavanum* Wiedemann, 1819, by original designation. Status: junior synonym of *Notocorax* Dejean, 1834 in Blaptinae: Platynotini: Platynotina. Synonymy: Iwan (1990: 123).

*Platyesia* Skopin, 1971: 335 [F]. Type species: *Diesiakarelini* Fischer von Waldheim, 1844, by original designation. Status: valid genus in Pimeliinae: Pimeliini.

*Platyesthus* Mäklin, 1878: 92 [M]. Type species: *Platyesthuspallidipennis* Mäklin, 1878, by monotypy. Status: valid genus in Stenochiinae: Stenochiini.

*Platyholmus* Dejean, 1834: 180 [M]. Type species: *Praocisdilaticollis* Lacordaire, 1830, by subsequent designation (Solier 1841a: 270 [370]). Status: valid genus in Pimeliinae: Praociini.

*Platykochius* Iwan, 2002a: 48, 88 [M]. Type species: *Selinusplatessa* Fairmaire, 1887, by original designation. Status: junior synonym of *Anchophthalmops* Koch, 1956 in Blaptinae: Platynotini: Platynotina. Synonymy: Kamiński (2015a: 89).

*Platylus* Mulsant & Rey, 1859a: 70 [M]. Type species: *Blapsdilatata* Fabricius, 1798, by monotypy. Status: valid genus in Blaptinae: Opatrini: Blapstinina.

*Platymedvedevia* Iwan & Banaszkiewicz, 2007: 725 [F]. Type species: *Platymedvedeviademeyeri* Iwan & Banaszkiewicz, 2007, by monotypy. Status: junior synonym of *Angolositus* Koch, 1955 in Blaptinae: Platynotini: Platynotina. Synonymy: Kamiński (2015a: 90).

*Platynoscelis* Kraatz, 1882: 91 [F]. Type species: *Platynoscelishelopioides* Kraatz, 1882, by original designation. Status: valid subgenus of *Bioramix* Bates, 1879 in Blaptinae: Platyscelidini.

*Platynosum* Mulsant & Rey, 1859c: 73, 81, 150 [N]. Type species: *Platynosumpaulinae* Mulsant & Rey, 1859, by monotypy. Status: valid genus in Blaptinae: Opatrini: Sclerina. Note: the spelling *Platysum* (pp. 73, 81) was corrected to *Platynosum* in the “Errata” of the same work (p. 150), *Platynosum* is considered to be the correct original spelling (ICZN 1999, Article 32.5.1.1).

*Platynotoides* Kaszab, 1975b: 281, 296 [M]. Type species: *Platynotusbellii* Fairmaire, 1896, by original designation. Status: valid genus in Blaptinae: Platynotini: Platynotina.

*Platynotus* Fabricius, 1801a: 138 [M]. Type species: *Blapsexcavata* Fabricius, 1775, by subsequent designation (Hope 1841: 110). Status: valid genus in Blaptinae: Platynotini: Platynotina.

*Platyolmus* Burmeister, 1875: 492 [M]. Type species [automatic]: *Praocisdilaticollis* Lacordaire, 1830, by subsequent designation (Solier 1841a: 270 [370]). Status: junior synonym of *Platyholmus* Dejean, 1834 in Pimeliinae: Praociini. Note: unjustified emendation of *Platyholmus* Dejean, 1834, not in prevailing usage.

*Platyolus* Mulsant & Rey, 1854: 153 [M]. Type species: *Microsituslongulus* Mulsant & Rey, 1854, by subsequent designation (Viñolas 1990: 65). Status: valid subgenus of *Phylan* Sturm, 1826 in Blaptinae: Dendarini: Dendarina.

*Platyope* Fischer, 1820: pl. 15 [F]. Type species: *Tenebrioleucographus* Pallas, 1781, by subsequent designation (Jacquelin du Val 1860: 261). Status: valid genus in Pimeliinae: Pimeliini. Note: Pallas (1773) used two different spellings for this type species, *Tenebrioleucographus* (p. 474) and *Tenebrioleucogrammus* (p. 719); subsequently, Pallas (1781: 54) used *T.leucographus* as the valid name of the species and so acted as First Reviser (ICZN 1999: Article 24.2.4).

*Platyotus* Gerstaecker, 1871: 62 [M]. Type species: *Platyotusglabratus* Gerstaecker, 1871, by monotypy. Status: junior synonym of *Palorus* Mulsant, 1854 in Tenebrioninae: Palorini. Synonymy: Halstead (1967a: 72). Note: name also described as new in Gerstaecker (1873: 185).

*Platyphanes* Westwood, 1849: 206 [M]. Type species: *Platyphanesgibbosus* Westwood, 1849, by subsequent designation (Lacordaire 1859b: 410). Status: valid genus in Tenebrioninae: Heleini: Cyphaleina.

*Platyphanus* Koch, 1952b: 26 [M]. Type species: *Acestussimilis* Haag-Rutenberg, 1878, by original designation. Status: valid genus in Pimeliinae: Adelostomini.

*Platyprocnemis* Español & Lindberg, 1963: 19, 21 [F]. Type species: *Trichosternumgranulosum* Wollaston, 1868, by original designation. Status: valid genus in Blaptinae: Opatrini: Ammobiina.

*Platyprosodes* Reitter, 1909a: 121 [M]. Type species: *Nyctipatesrugulosus* Gebler, 1841, by original designation. Status: junior synonym of *Prosodes* Eschscholtz, 1829 in Blaptinae: Blaptini: Prosodina. Synonymy: Skopin (1960a: 46).

*Platypsorodes* Ardoin, 1963b: 307, 310 [M]. Type species: *Acanthomerushelopioides* Guérin-Méneville, 1834, by original designation. Status: valid genus in Tenebrioninae: Amarygmini.

*Platyscelis* Latreille, 1818: 23 [F]. Type species: *Tenebriohypolithus* Pallas, 1781, by plenary powers (ICZN 1993b, Opinion 1729). Status: valid genus and subgenus in Blaptinae: Platyscelidini. Note: placed on the Official List of Generic Names in Zoology (ICZN 1993b, Opinion 1729).

*Platysemodes* Strand, 1935b: 302 [M]. Type species [automatic]: *Platysemusbenguelensis* Haag-Rutenberg, 1875, by monotypy. Status: valid genus in Pimeliinae: Adelostomini. Note: replacement name for *Platysemus* Haag-Rutenberg, 1875.

*Platysemus* Haag-Rutenberg, 1875b: 4, 55 [M]. Type species: *Platysemusbenguelensis* Haag-Rutenberg, 1875, by monotypy. Status: senior synonym of *Platysemodes* Strand, 1935 in Pimeliinae: Adelostomini. Note: junior homonym of *Platysemus* Middendorff, 1848 [Mollusca].

*Platystena* Koch, 1940c: 95 [F]. Type species: *Mesostenablairi* Koch, 1940, by monotypy. Status: valid subgenus of *Mesostena* Eschscholtz, 1831 in Pimeliinae: Tentyriini. Note: combined description of a new genus-group taxon and a single new species (ICZN 1999, Article 13.4).

*Plegacerus* Gebien, 1921b: 142 [M]. Type species: *Plegacerussensitivus* Gebien, 1921, by monotypy. Status: valid genus in Tenebrioninae: Amarygmini.

*Pleioplatyscelis* Kaszab, 1940a: 153, 220 [F]. Type species: *Platynoscelislederi* Seidlitz, 1893, by original designation. Status: junior synonym of *Planoplatyscelis* Kaszab, 1940 in Blaptinae: Platyscelidini. Synonymy: Egorov (2004: 601).

*Pleiopleura* Seidlitz, 1893: 342, 343 [F]. Type species: *Platyscelisstriatus* Motschulsky, 1859, by monotypy. Status: valid subgenus of *Platyscelis* Latreille, 1818 in Blaptinae: Platyscelidini.

*Pleisiasida* Smith, 2013: 610 [F]. Type species [automatic]: *Parasidalaciniata* Casey, 1912, by original designation. Status: valid subgenus of *Pelecyphorus* Solier, 1836 in Pimeliinae: Asidini. Note: replacement name for *Parasida* Casey, 1912.

*Plesia* Klug, 1833: 25 [F]. Type species: *Plesiamelanura* Klug, 1833, by subsequent designation (Bousquet et al. 2015: 137). Status: senior synonym of *Eubalia* Laporte, 1840 in Alleculinae: Alleculini: Gonoderina. Synonymy: Borchmann (1909a: 713). Note: junior homonym of *Plesia* Jurine, 1807 [Hymenoptera].

*Plesiamarygmus* Masumoto, 1989b: 314 [M]. Type species: *Dietysusovoideus* Fairmaire, 1882, by original designation. Status: junior synonym of *Amarygmus* Dalman, 1823 in Tenebrioninae: Amarygmini. Synonymy: Bremer (2005: 205).

*Plesioderes* Mulsant & Rey, 1859c: 126 [M]. Type species: *Caediuscoriaceus* Mulsant & Rey, 1859, by subsequent designation (Gebien 1939: 466). Status: valid genus in Blaptinae: Opatrini: Ammobiina.

*Plesiognaptor* Chigray, Nabozhenko & Keskin, 2015: 1277 [M]. Type species: *Gnaptorprolixus* Fairmaire, 1866, by original designation. Status: valid subgenus of *Gnaptor* Brullé, 1831 in Blaptinae: Blaptini: Gnaptorina.

*Plesiophthalmus* Motschulsky, 1857: 34 [M]. Type species: *Plesiophthalmusnigrocyaneus* Motschulsky, 1857, by monotypy. Status: valid genus and subgenus in Tenebrioninae: Amarygmini.

*Pleuromophlus* Reitter, 1906b: 138, 146 [M]. Type species: *Omophlusbaudueri* Baudi, 1877, by subsequent designation (Bousquet et al. 2015: 141). Status: junior synonym of *Odontomophlus* Seidlitz, 1896 in Alleculinae: Cteniopodini. Synonymy: Bousquet et al. (2015: 141).

*Pleurophorus* Solier, 1851: 162 [M]. Type species: *Pleurophorusquadricollis* Solier, 1851, by monotypy. Status: senior synonym of *Discopleurus* Lacordaire, 1859 in Pimeliinae: Stenosini: Dichillina. Note: junior homonym of *Pleurophorus* Mulsant, 1842 [Coleoptera: Scarabaeidae].

*Pleurostira* Borchmann, 1921: 217, 230 [F]. Type species: *Pleurostiraepipleuralis* Borchmann, 1921, by original designation. Status: valid subgenus of *Statira* Lepeletier & Audinet-Serville, 1828 in Lagriinae: Lagriini: Statirina.

*Pleuroxycara* Koch, 1959: 582 [N]. Type species: *Oxycaraproblematicum* Koch, 1959, by original designation. Status: valid subgenus of *Oxycara* Solier, 1835 in Pimeliinae: Tentyriini.

*Plicatocerus* Pic, 1918a: 11 [M]. Type species: *Otocerusimpressipennis* Champion, 1888, by monotypy. Status: valid subgenus of *Oploptera* Chevrolat, 1844 in Stenochiinae: Stenochiini.

*Plinthochrous* Fairmaire, 1891b: 263 [M]. Type species: *Plinthochrousgounellei* Fairmaire, 1891, by monotypy. Status: valid genus in Tenebrioninae: Amarygmini.

*Pocadiopsis* Fairmaire, 1896a: 17 [F]. Type species: *Pocadiopsissimulator* Fairmaire, 1896, by subsequent designation (Gebien 1939: 457). Status: valid genus in Blaptinae: Opatrini: incertae sedis. Note: placed in Opatrini incertae sedis by Kamiński et al. (2021b: 151).

*Podacamptus* Ardoin, 1964a: 849 [M]. Type species: *Podacamptusruficolor* Ardoin, 1964, by monotypy. Status: valid genus in Tenebrioninae: Amarygmini.

*Podamarygmus* Carter, 1928: 287 [M]. Type species: *Podamarygmusalternatus* Carter, 1928 (= *Amarygmusviridipes* Gebien, 1927), by monotypy. Status: valid subgenus of *Amarygmus* Dalman, 1823 in Tenebrioninae: Amarygmini.

*Podhomala* Solier, 1836: 9, 72 [F]. Type species: *Podhomalasuturalis* Solier, 1836, by monotypy. Status: valid genus and subgenus in Pimeliinae: Pimeliini.

*Podoces* Péringuey, 1886: 122 [F]. Type species: *Podocesgranosula* Péringuey, 1886, by **present designation**. Status: senior synonym of *Carchares* Pascoe, 1887 in Tenebrioninae: Scaurini. Synonymy: Péringuey (1892b: [135]). Note: junior homonym of *Podoces* Fischer, 1821 [Aves].

*Pododonta* Agassiz, 1846b: 300 [F]. Type species [automatic]: *Cistelanigrita* Fabricius, 1794, by subsequent designation (R. Lucas 1920: 527). Status: junior synonym of *Podonta* Solier, 1835 in Alleculinae: Cteniopodini. Note: unjustified emendation of *Podonta* Solier, 1835, not in prevailing usage.

*Podomala* Agassiz, 1846b: 300 [F]. Type species [automatic]: *Podhomalasuturalis* Solier, 1836, by monotypy. Status: junior synonym of *Podhomala* Solier, 1836 in Pimeliinae: Pimeliini. Note: unjustified emendation of *Podhomala* Solier, 1836, not in prevailing usage.

*Podonta* Solier, 1835a: 247 [F]. Type species: *Cistelanigrita* Fabricius, 1794, by subsequent designation (Cazurro Ruiz 1894b: 883). Status: valid genus in Alleculinae: Cteniopodini.

*Podontinus* Seidlitz, 1896: 197 [M]. Type species: *Cteniopuspunctatissimus* Kiesenwetter, 1861, by subsequent designation (R. Lucas 1920: 527). Status: valid genus in Alleculinae: Cteniopodini.

*Poecilesthostrongylium* Pic, 1918a: 12 [N]. Type species: *Strongyliumamazonicum* Pic, 1918, by monotypy. Status: junior synonym of *Strongylium* W. Kirby, 1819 in Stenochiinae: Stenochiini. Synonymy: Gebien (1948: 519).

*Poecilesthus* Dejean, 1834: 207 [M]. Type species: *Erotylusfasciatus* Fabricius, 1781, by subsequent designation (Hope 1841: 133). Status: valid genus in Stenochiinae: Stenochiini. Note: *Poecilesthus* is an incorrect subsequent spelling of the original spelling *Paecilesthus*, first used by Dejean (1836: 229), and in prevailing usage; *Poecilesthus* is deemed to be the correct original spelling (ICZN 1999, Article 33.3.1), see Bousquet and Bouchard (2013a: 60).

*Poecilocrypticus* Gebien, 1928: 121 [M]. Type species: *Poecilocrypticusformicophilus* Gebien, 1928, by monotypy. Status: valid genus in Diaperinae: Crypticini.

*Poeciltoides* Fairmaire, 1896b: 352 [M]. Type species: *Poeciltoidesalternans* Fairmaire, 1896, by monotypy. Status: valid genus in Stenochiinae: Cnodalonini.

*Pogonobasis* Solier, 1837b: 153, 161 [F]. Type species: *Pogonobasisopatroides* Solier, 1837 (= *Eurychorarugosula* Guérin-Méneville, 1831), by subsequent designation (Hope 1841: 121). Status: valid genus in Pimeliinae: Adelostomini.

*Pogonocanta* Koch, 1952b: 22 [F]. Type species: *Pogonobasislongipilis* Fairmaire, 1894, by original designation. Status: valid genus in Pimeliinae: Adelostomini. Note: the alternative original spelling *Pogonacanta*, used by Koch (1952b: 123), was rejected by Koch (1953c: 3) who acted as the First Reviser (ICZN 1999, Article 24.2.4).

*Pogonophloeus* Bremer, 1998: 9 [M]. Type species: *Hypophlaeusthoracicus* Melsheimer, 1846, by original designation. Status: valid subgenus of *Corticeus* Piller & Mitterpacher, 1783 in Diaperinae: Hypophlaeini.

*Pogonoxenus* Wasmann, 1899b: 172 [M]. Type species: *Pogonoxenuskraatzi* Wasmann, 1899, by monotypy. Status: valid genus in Diaperinae: Hypophlaeini.

*Pokryszkiella* Iwan, 1996: 385, 414 [F]. Type species: *Pokryszkiellacornuta* Iwan, 1996, by original designation. Status: valid genus in Blaptinae: Platynotini: Platynotina.

*Polasida* Reitter, 1917a: 39, 41 [F]. Type species: *Opatrumsericeum* G.-A. Olivier, 1795, by subsequent designation (Viñolas and Cartagena 2005: 188). Status: valid subgenus of *Asida* Latreille, 1802 in Pimeliinae: Asidini.

*Polemiotus* Casey, 1907: 379, 381 [M]. Type species: *Epitragussubmetallicus* J.L. LeConte, 1854, by original designation. Status: valid genus in Pimeliinae: Epitragini.

*Poliorcetes* Champion, 1884: 70 [M]. Type species: *Poliorcetesplatesthoides* Champion, 1884, by monotypy. Status: valid subgenus of *Pelecyphorus* Solier, 1836 in Pimeliinae: Asidini.

*Polopinus* Casey, 1924: 326 [M]. Type species: *Polypleurusnitidus* J.L. LeConte, 1866, by original designation. Status: valid genus in Stenochiinae: Cnodalonini.

*Polpocara* Solier, 1843: 46, 123 [N]. Type species: *Polpocarapicipes* Solier, 1843, by original designation. Status: junior synonym of *Philorea* Erichson, 1834 in Pimeliinae: Physogasterini. Synonymy: Erichson (1847a: 116).

*Polpogenia* Solier, 1836: 9, 70 [F]. Type species: *Polpogeniaasidioides* Solier, 1836, by monotypy. Status: valid genus in Pimeliinae: Pimeliini.

*Polposipus* Solier, 1848: 154, 260 [M]. Type species: *Polposipusherculeanus* Solier, 1848, by original designation. Status: valid genus in Stenochiinae: Cnodalonini.

*Polycoelogastridion* Reichardt, 1936: 85, 208 [N]. Type species: *Sclerumsexcostatum* Motschulsky, 1858, by original designation. Status: valid genus in Blaptinae: Opatrini: Opatrina.

*Polyidus* Champion, 1888: 441 [M]. Type species: *Polyidusmeridionalis* Champion, 1888, by monotypy. Status: valid genus in Alleculinae: Alleculini: Alleculina.

*Polypleurus* Eschscholtz, 1831: 10, 11 [M]. Type species: *Polypleurusgeminatus* Eschscholtz, 1831, by monotypy. Status: valid genus in Stenochiinae: Cnodalonini.

*Polyscopus* Waltl, 1835: 73 [M]. Type species: *Polyscopuscostatus* Waltl, 1835 (= *Adelostomasulcatum* Duponchel, 1827), by monotypy. Status: junior synonym of *Adelostoma* Duponchel, 1827 in Pimeliinae: Adelostomini. Synonymy: Erichson in Agassiz (1846a: 133).

*Polytropus* Kirsch, 1866: 201 [M]. Type species: *Polytropuslaenoides* Kirsch, 1866, by monotypy. Status: junior synonym of *Chaetyllus* Pascoe, 1860 in Lagriinae: Laenini. Synonymy: Kaszab (1983a: 138).

*Ponapeida* Kulzer, 1957: 242, 248 [F]. Type species: *Ponapeidarufitarsis* Kulzer, 1957, by original designation. Status: valid genus in Stenochiinae: Cnodalonini.

*Pontianacus* Fairmaire, 1898d: 397 [M]. Type species: *Pontianacusrubricrus* Fairmaire, 1898, by monotypy. Status: valid genus in Tenebrioninae: Amarygmini.

*Poopterocoma* Skopin, 1974b: 145 [F]. Type species: *Pterocomazaidamica* Skopin, 1974, by original designation. Status: valid subgenus of *Pterocoma* Dejean, 1834 in Pimeliinae: Pimeliini.

*Porphyrhyba* Fairmaire, 1877a: 137 [F]. Type species: *Porphyrhybaviolaceicolor* Fairmaire, 1877, by monotypy. Status: valid genus in Stenochiinae: Cnodalonini.

*Porphyrohyba* Rye, 1879: 62 [F]. Type species [automatic]: *Porphyrhybaviolaceicolor* Fairmaire, 1877, by monotypy. Status: junior synonym of *Porphyrhyba* Fairmaire, 1877 in Stenochiinae: Cnodalonini. Note: unjustified emendation of *Porphyrhyba* Fairmaire, 1877, not in prevailing usage.

*Porrolagria* Kolbe, 1883: 26 [F]. Type species: *Porrolagrianuda* Kolbe, 1883, by monotypy. Status: valid genus in Lagriinae: Lagriini: Lagriina.

*Posides* Champion, 1884: 6 [M]. Type species: *Posidesdissidens* Champion, 1884, by monotypy. Status: valid genus in Pimeliinae: Edrotini.

*Postandrosus* Kulzer, 1951b: 490 [M]. Type species: *Postandrosusmaculipennis* Kulzer, 1951, by original designation. Status: valid genus in Stenochiinae: Cnodalonini.

*Postpraocis* Flores & Pizarro-Araya, 2014: 60 [M]. Type species: *Praocispentachorda* Burmeister, 1875, by original designation. Status: valid subgenus of *Praocis* Eschscholtz, 1829 in Pimeliinae: Praociini. Note: this name was first proposed by Kulzer (1958a: 12, 33) without type species designation.

*Potocula* Novák, 2012: 290 [F]. Type species: *Potoculakubani* Novák, 2012, by original designation. Status: valid genus in Alleculinae: Alleculini: Alleculina.

*Praeugena* Laporte, 1840: 241 [F]. Type species: *Helopsmarginatus* Fabricius, 1792, by subsequent designation (Hope 1841: 133). Status: valid genus in Tenebrioninae: Praeugenini. Note: nomen protectum (see Bouchard and Bousquet 2020b: 6).

†*Praezolodinus* Bao in Bao and Antunes-Carvalho, 2020: 2 [M]. Type species: *Praezolodinuspilosus* Bao, 2020, by original designation. Status: valid genus in Zolodininae. Note: described from Cretaceous Burmese amber (Myanmar).

*Praocida* Flores & Pizarro-Araya, 2014: 70 [F]. Type species: *Praociszischkai* Kulzer, 1958, by original designation. Status: valid subgenus of *Praocis* Eschscholtz, 1829 in Pimeliinae: Praociini. Note: this name was first proposed by Kulzer (1958a: 13, 88) without type species designation.

*Praocidia* Fairmaire, 1904b: 463 [F]. Type species: *Praocisnervosus* Fairmaire, 1902, by original designation. Status: valid genus in Pimeliinae: Praociini.

*Praocis* Eschscholtz, 1829: 6 [M]. Type species: *Praocisrufipes* Eschscholtz, 1829, by subsequent designation (Guérin-Méneville 1834: 8–9). Status: valid genus and subgenus in Pimeliinae: Praociini. Note: although Guérin-Méneville (1834: 8–9) did not specifically mention the scientific name of the species he considered the type of this genus, his comment “J’ai donné une nouvelle figure de l’espèce qui a servi de type à Eschscholtz, dans la pl. 4, fig. 1, du Voyage de la Coquille” [translated to “I gave a new figure of the species that served as the type by Eschscholtz, in pl. 4, fig. 1, of the Voyage de la Coquille”] leaves no ambiguity regarding the identity of the species, i.e., *Praocisrufipes* Eschscholtz, 1829, to which he was referring.

*Praogena* Agassiz, 1846b: 306 [F]. Type species [automatic]: *Helopsmarginatus* Fabricius, 1792, by subsequent designation (Hope 1841: 133). Status: junior synonym of *Praeugena* Laporte, 1840 in Tenebrioninae: Praeugenini. Note: unjustified emendation of *Praeugena* Laporte, 1840, not in prevailing usage.

*Praonoda* Flores & Pizarro-Araya, 2014: 68 [F]. Type species: *Praocisbicarinatus* Burmeister, 1875, by original designation. Status: valid subgenus of *Praocis* Eschscholtz, 1829 in Pimeliinae: Praociini. Note: this name was first proposed by Kulzer (1958a: 13, 66) without type species designation.

*Praostetha* Fairmaire, 1897f: 138 [F]. Type species: *Praostethaimpressifrons* Fairmaire, 1897, by monotypy. Status: junior synonym of *Amenophis* J. Thomson, 1858 in Stenochiinae: Cnodalonini. Synonymy: Gebien (1921b: 64).

*Prateus* J.L. LeConte, 1862: 238 [M]. Type species: *Prateusfusculus* J.L. LeConte, 1862, by original designation. Status: valid genus in Lagriinae: Goniaderini.

*Predactylosis* Penrith, 1977: 19, 243 [F]. Type species: *Predactylosisholmi* Penrith, 1977, by original designation. Status: junior synonym of *Zophosis* Latreille, 1802 in Pimeliinae: Zophosini. Synonymy: Penrith (1981a: 22).

*Priocamaria* Gebien, 1919: 28, 143 [F]. Type species: *Priocamariamacilenta* Gebien, 1919, by subsequent designation (Gebien 1942a: 323). Status: valid genus in Stenochiinae: Cnodalonini.

*Prionalia* Novák, 2020c: 511 [F]. Type species: *Gonoderaatronitens* Fairmaire, 1892, by original designation. Status: valid genus in Alleculinae: Alleculini: Alleculina.

*Prionotheca* Dejean, 1834: 179 [F]. Type species: *Pimeliacoronata* G.-A. Olivier, 1795, by monotypy. Status: valid genus in Pimeliinae: Pimeliini.

*Prionotus* Mulsant & Rey, 1859c: 88 [M]. Type species: *Opatrumdenticolle* Blanchard, 1846, by monotypy. Status: junior synonym of *Isopteron* Hope, 1841 in Lagriinae: Adeliini. Synonymy: Gemminger in Gemminger and Harold (1870: 1929, with *Achora* Pascoe, 1869, a junior synonym of *Isopteron* Hope, 1841). Note: junior homonym of *Prionotus* Lacepède, 1802 [Pisces].

*Prionychus* Solier, 1835a: 231, 237 [M]. Type species: *Helopsater* Fabricius, 1775, by subsequent designation (R. Lucas 1920: 536). Status: valid genus in Alleculinae: Alleculini: Alleculina. Note: nomen protectum (see Bousquet and Bouchard 2017: 132); see Löbl and Smetana (2011: 33) for comments on the gender of this genus-group name.

*Prioproctus* Kolbe, 1903: 165, 174 [M]. Type species: *Prioproctusoertzeni* Kolbe, 1903, by original designation. Status: valid genus in Lagriinae: Pycnocerini.

*Prioscelida* White, 1846: 11 [F]. Type species: *Prioscelidatenebrionoides* White, 1846, by monotypy. Status: junior synonym of *Uloma* Dejean, 1821 in Tenebrioninae: Ulomini. Synonymy: Broun (1880: 365).

*Prioscelides* Kolbe, 1889: 128 [M]. Type species: *Prioscelidesrugosus* Kolbe, 1889, by monotypy. Status: valid genus in Lagriinae: Pycnocerini.

*Prioscelis* Hope, 1841: 128 [F]. Type species: *Prioscelisfabricii* Hope, 1841, by original designation. Status: valid genus in Lagriinae: Pycnocerini. Note: the alternative original spelling *Priopus*, used by Hope (1841: 73), was rejected by Westwood (1844: 211) who acted as First Reviser.

*Priothorax* Gebien, 1910b: 318 [M]. Type species [automatic]: *Opatrumdenticolle* Blanchard, 1846, by monotypy. Status: junior synonym of *Isopteron* Hope, 1841 in Lagriinae: Adeliini. Synonymy: Gebien (1910b: 318, as a valid synonym of *Achora* Pascoe, 1869), Blair (1919a: 529, as a synonym of *Achora* Pascoe, 1869, a junior synonym of *Isopteron* Hope, 1841). Note: replacement name for *Prionotus* Mulsant & Rey, 1859.

*Pristophilus* Kolbe, 1903: 165, 174 [M]. Type species: *Chiroscelispassaloides* Westwood, 1842, by original designation. Status: valid genus in Lagriinae: Pycnocerini.

*Probaticus* Seidlitz, 1895: 697, 704, 764 [M]. Type species: *Helopsmori* Brullé, 1832, by subsequent designation (Gebien 1943: 419). Status: junior synonym of *Euboeus* Boieldieu, 1865 in Tenebrioninae: Helopini: Helopina. Synonymy: Nabozhenko et al. (2017: 496). Note: the alternative original spelling *Probatius* (pp. 697, 704, 765) was corrected to *Probaticus* in the “Amendments and corrections” of the same work (p. 849), *Probaticus* is considered to be the correct original spelling (ICZN 1999, Article 32.5.1.1).

*Prochoma* Solier, 1835b: 253, 393 [N]. Type species: *Prochomaaudouini* Solier, 1835, by monotypy. Status: valid genus and subgenus in Pimeliinae: Tentyriini.

*Proctenius* Reitter, 1890b: 256 [M]. Type species: *Cistelagranatensis* Rosenhauer, 1856, by monotypy. Status: valid genus in Alleculinae: Cteniopodini.

*Proderops* Fairmaire, 1873: 393 [M]. Type species: *Proderopsforaminosus* Fairmaire, 1873 (= *Rhinandruselongatus* Horn, 1867), by monotypy. Status: junior synonym of *Rhinandrus* J.L. LeConte, 1866 in Tenebrioninae: Tenebrionini. Synonymy: Kraatz (1880a: 132, with *Exerestus* Bates, 1870, a junior synonym of *Rhinandrus* J.L. LeConte, 1866).

*Prodilamus* Ardoin, 1969e: 258 [M]. Type species: *Dilamusbrevicollis* Fairmaire, 1894, by original designation. Status: valid genus in Blaptinae: Opatrini: Ammobiina.

*Prohylithus* Kaszab, 1964c: 382 [M]. Type species: *Prohylithuskulzeri* Kaszab, 1964, by original designation. Status: valid genus in Pimeliinae: Edrotini.

*Prolabrus* Fairmaire, 1897a: 111 [M]. Type species: *Prolabrusparallelus* Fairmaire, 1897, by monotypy. Status: valid genus in Tenebrioninae: Palorini.

*Prolaena* Kaszab, 1980b: 322 [F]. Type species: *Laenaceylonica* Motschulsky, 1858, by original designation. Status: valid genus in Lagriinae: Laenini. Note: *Prolaena* was used earlier by Kaszab (1979a: 107) without a description, a definition, or a bibliographic reference to such a published statement (ICZN 1999, Article 13.1) and is therefore not available from that date.

*Proleptodes* G.S. Medvedev, 1967: 354 [M]. Type species: *Leptodessulcicollis* Reitter, 1889, by original designation. Status: valid subgenus of *Leptodes* Dejean, 1834 in Pimeliinae: Leptodini.

*Promethis* Pascoe, 1869: 148 [M]. Type species: *Upisangulata* Erichson, 1842, by original designation. Status: valid genus in Stenochiinae: Cnodalonini.

*Prometopion* Casey, 1907: 366, 370 [N]. Type species: *Prometopionamplipenne* Casey, 1907 (= *Chilometoponhelopioides* Horn, 1874), by original designation. Status: junior synonym of *Chilometopon* Horn, 1874 in Pimeliinae: Edrotini. Synonymy: MacLachlan and Olson (1990: 72).

*Promorphostenophanes* Kaszab, 1960b: 277 [M]. Type species: *Promorphostenophanesatavus* Kaszab, 1960, by original designation. Status: junior synonym of *Morphostenophanes* Pic, 1925 in Stenochiinae: Cnodalonini. Synonymy: Masumoto and Bečvář (2008: 206).

*Promus* J.L. LeConte, 1862: 226 [M]. Type species: *Blapsopaca* Say, 1824, by original designation. Status: valid subgenus of *Eleodes* Eschscholtz, 1829 in Blaptinae: Amphidorini.

*Propemicrosis* Penrith, 1981c: 127, 152 [F]. Type species: *Microsistransbechuana* Koch, 1958, by original designation. Status: valid subgenus of *Zophosis* Latreille, 1802 in Pimeliinae: Zophosini.

*Prophanes* Westwood, 1849: 203 [M]. Type species: *Prophanesaculeatus* Westwood, 1849, by subsequent designation (Lacordaire 1859b: 411). Status: valid genus in Tenebrioninae: Heleini: Cyphaleina.

*Propterocoma* Skopin, 1974b: 144 [F]. Type species: *Pterocomatibialis* Bates, 1879, by original designation. Status: valid subgenus of *Pterocoma* Dejean, 1834 in Pimeliinae: Pimeliini.

*Prorhytinota* Bouchard & Bousquet, **new subgenus** [F]. Type species: *Rhytinotaoxyoma* Fairmaire, 1884, by **present designation**. Status: valid subgenus of *Rhytinota* Eschscholtz, 1831 in Pimeliinae: Tentyriini. Note: Koch (1943b: 761, 795) introduced the new subgenus name *Prorhytinota* for several nominal species, but unfortunately did not designate a type species; the subgenusProrhytinota, which has been treated as valid since 1943, is therefore unavailable (ICZN 1999, Article 13.3); we hereby make the name available by selecting *Rhytinotaoxyoma* Fairmaire, 1884 as type species and referring to Koch (1943b: 761, 795) for the character states that characterise and differentiate *Prorhytinota*.

*Proscheimus* Desbrochers des Loges, 1881: 127 [M]. Type species: *Proscheimusarabicus* Desbrochers des Loges, 1881, by monotypy. Status: valid genus in Blaptinae: Opatrini: Ammobiina.

*Proscorus* Fairmaire, 1901b: 188 [M]. Type species: *Proscoruscyaneostriatus* Fairmaire, 1901, by monotypy. Status: valid genus in Stenochiinae: Cnodalonini.

*Proselytus* Fåhraeus, 1870: 302 [M]. Type species: *Proselytuscaffer* Fåhraeus, 1870 (= *Alphitobiusdiaperinus* Panzer, 1796), by monotypy. Status: junior synonym of *Alphitobius* Stephens, 1829 in Tenebrioninae: Alphitobiini. Synonymy: Ferrer (1999: 266; through synonymy of the type species).

*Prosoblapsia* Skopin & Kaszab, 1978: 208 [F]. Type species: *Leptocolenaallardiana* Reitter, 1889, by original designation. Status: junior synonym of *Genoblaps* Bauer, 1921 in Blaptinae: Blaptini: Blaptina. Synonymy: **new synonym** [YB]. Note: *Genoblaps* Bauer, 1921 has been forgotten in the literature; its type species is currently included in the subgenusProsoblapsia Skopin & Kaszab, 1978 and for that reason Skopin & Kaszab’s name is considered a junior synonym of *Genoblaps*.

*Prosodella* Reitter, 1909a: 120 [F]. Type species: *Prosodesbactriana* Semenov, 1894, by original designation. Status: valid subgenus of *Prosodes* Eschscholtz, 1829 in Blaptinae: Blaptini: Prosodina. Note: the First Reviser (*Prosodella* Reitter, 1909 versus *Paraprosodes* Reitter, 1909) is G.S. Medvedev (1997: 597).

*Prosodes* Eschscholtz, 1829: 9 [F]. Type species: *Blapsattenuata* Fischer, 1820 (= *Blaps obtusa* Fabricius, 1798), by subsequent designation (Gebien 1937a: 846). Status: valid genus and subgenus in Blaptinae: Blaptini: Prosodina. Note: the gender of *Prosodes* is feminine by indication of the author himself, who states: “von προσώδης, foetida”, the latter word being Latin and explaining the Greek meaning (stinking); the word foetida is feminine and therefore *Prosodes* is feminine also; note, however, that all other genus-group names in this list with the ending –*prosodes* are masculine.

*Prosodestes* Reitter, 1909a: 114 [M]. Type species: *Prosodesgrandicollis* Kraatz, 1883, by original designation. Status: valid subgenus of *Prosodes* Eschscholtz, 1829 in Blaptinae: Blaptini: Prosodina.

*Prosodidius* Fairmaire, 1903d: 69 [M]. Type species: *Prosodidiusperrieri* Fairmaire, 1903, by monotypy. Status: valid genus in Pimeliinae: Asidini.

*Prosodila* Reitter, 1909a: 121 [F]. Type species: *Prosodesstrigiventris* Reitter, 1893, by original designation. Status: junior synonym of *Prosodes* Eschscholtz, 1829 in Blaptinae: Blaptini: Prosodina. Synonymy: Löbl et al. (2008c: 219).

*Prosodinia* Reitter, 1909a: 115 [F]. Type species: *Prosodescalcarata* Reitter, 1893, by original designation. Status: valid subgenus of *Prosodes* Eschscholtz, 1829 in Blaptinae: Blaptini: Prosodina.

*Prosodopria* Reitter, 1909a: 116 [F]. Type species: *Blapsangustata* Zubkov, 1833, by monotypy. Status: valid subgenus of *Prosodes* Eschscholtz, 1829 in Blaptinae: Blaptini: Prosodina.

*Prosodoscelis* Reitter, 1909a: 117 [F]. Type species: *Prosodessolskyi* Faust, 1875, by original designation. Status: valid subgenus of *Prosodes* Eschscholtz, 1829 in Blaptinae: Blaptini: Prosodina.

*Prosodura* Reitter, 1909a: 118 [F]. Type species: *Prosodessemenowi* Reitter, 1893, by original designation. Status: valid subgenus of *Prosodes* Eschscholtz, 1829 in Blaptinae: Blaptini: Prosodina.

*Prosomenes* Blanchard, 1845: 10 [M]. Type species: *Diceroderesmexicanus* Solier, 1841, by subsequent monotypy (Chevrolat 1847b: 562). Status: junior synonym of *Diceroderes* Solier, 1841 in Tenebrioninae: Toxicini: Dysantina. Synonym: Lacordaire (1859a: 356, as “*Prosomenes* Dejean”), Bousquet et al. (2018: 219). Note: the name *Prosomenes* was originally introduced as a synonym of “Dicérodères” by Blanchard (1845: 10); however, because the name was treated before 1961 as an available name and adopted as the name of a taxon (e.g., Chevrolat 1847b: 562) we treat it as available and dating from its first publication as a synonym (ICZN 1999, Article 11.6.1); originally proposed without included nominal species; Chevrolat (1847b: 562), by including the species “*Pros.mexicanus*” in association with this name, was the first author to subsequently and expressly include nominal species in *Prosomenes* (ICZN 1999, Article 67.2.2).

*Prostenus* Klug, 1829: 5 [M]. Type species: *Prostenusperiscelis* Perty, 1830, by subsequent designation (Bousquet et al. 2015: 139, but see Note). Status: valid genus in Alleculinae: Alleculini: Xystropodina. Note: as pointed out by Bousquet et al. (2015: 139) this genus name had been attributed to other authors in the literature (e.g., Latreille, 1825: 377, Berthold, 1827: 369) but it was first made available by Klug (1829: 5) who described two new species in it, *Prostenusfemoratus* and *Prostenuspilosus*, neither species being included in *Prostenus* in its currently accepted sense; Bousquet et al. (2015: 139) suggested using *Prostenusperiscelis* Perty, 1830 as the type species of *Prostenus* to promote nomenclatural stability; however, an application to the Commission is needed to confirm the type species of *Prostenus*.

†*Proteleates* Wickham,1914a: 267 [M]. Type species: *Proteleatescentralis* Wickham, 1914, by original designation. Status: valid genus in Tenebrioninae: Bolitophagini. Note: described from Upper Eocene deposits (USA).

*Prothraustocola* Kaszab, 1957: 293 [F]. Type species: *Ibnsaudiabelutschistanica* Kaszab, 1957, by original designation. Status: valid subgenus of *Thraustocolus* Kraatz, 1866 in Pimeliinae: Tentyriini.

*Protoblaps* Bauer, 1921: 230, 231 [F]. Type species: none designated. Status: undetermined taxon in Blaptinae: Blaptini: Blaptina. Note: this genus was described before 1931 (ICZN 1999, Article 12.1); however, we could not find any nominal species that were subsequently and expressly included in *Protoblaps* and therefore no “originally included nominal species” could be used to fix the type species (ICZN 1999, Article 67.2.2).

*Protoblaps* G.S. Medvedev, 1998a: 200 [F]. Type species: *Protoblapskashkarovi* G.S. Medvedev, 1998, by original designation. Status: senior synonym of *Medvedevoblaps* Bouchard & Bousquet, **nom. nov.** in Blaptinae: Blaptini: Blaptina. Note: junior homonym of *Protoblaps* Bauer, 1921 [Coleoptera: Tenebrionidae: Blaptinae: Blaptini: Blaptina].

*Protocalosis* Penrith, 1977: 21, 116 [F]. Type species: *Zophosisbalti* Penrith, 1977, by original designation. Status: valid subgenus of *Zophosis* Latreille, 1802 in Pimeliinae: Zophosini.

*Protodactylus* Koch, 1952a: 83 [M]. Type species: *Protodactylusopticus* Koch, 1952, by original designation. Status: valid subgenus of *Zophosis* Latreille, 1802 in Pimeliinae: Zophosini.

*Protomachlasida* Escalera, 1928: 137 [F]. Type species: *Machlasidaliouvillei* Escalera, 1925, by original designation. Status: junior synonym of *Machlasida* Escalera, 1907 in Pimeliinae: Asidini. Synonymy: Gebien (1937a: 717).

†*Protoplatycera* Wickham, 1914b: 484 [F]. Type species: *Protoplatyceralaticornis* Wickham, 1914, by original designation. Status: valid genus in Tenebrionidae: incertae sedis. Note: described from Upper Eocene deposits (USA).

*Prototyrtaeus* Spiessberger & Ivie, 2020: 669 [M]. Type species: *Prototyrtaeusdarlingtoni* Spiessberger & Ivie, 2020, by original designation. Status: valid genus in Diaperinae: Gnathidiini: Anopidiina.

*Prunaspila* Koch, 1950a: 67 [F]. Type species [automatic]: *Aspilabicostata* Fåhraeus, 1870, by monotypy. Status: valid genus in Pimeliinae: Adelostomini. Note: replacement name for *Aspila* Fåhraeus, 1870.

*Przewalskia* Semenov, 1893: 262 [F]. Type species: *Platyopedilatata* Reitter, 1887, by original designation. Status: valid genus in Pimeliinae: Pimeliini.

*Psammestus* Reichardt, 1936: 194, 216 [M]. Type species: *Ammobiusdilatatus* Reitter, 1893, by original designation. Status: valid genus in Blaptinae: Opatrini: Ammobiina.

*Psammetichus* Latreille, 1828: 578 [M]. Type species: *Psammetichuscostatus* Guérin-Méneville, 1831, by subsequent monotypy (Guérin-Méneville 1831b: pl. 28bis). Status: valid genus in Pimeliinae: Elenophorini: Megelenophorina. Note: originally proposed without included nominal species; Guérin-Méneville (1831b: pl. 28bis), by including the new species “*Psammeticuscostatus* Guérin-Méneville, 1831” in association with this name, was the first author to subsequently and expressly include nominal species in *Psammetichus* (ICZN 1999, Article 67.2.2).

*Psammoardoinellus* Leo, 1981: 34 [M]. Type species: *Isocerussardiniensis* Ardoin, 1972, by original designation. Status: valid genus in Blaptinae: Dendarini: Melambiina.

*Psammocryptus* Kraatz, 1865: 81, 239 [M]. Type species: *Tentyriaminuta* Tauscher, 1812, by monotypy. Status: valid genus in Pimeliinae: Tentyriini.

*Psammodes* W. Kirby, 1819a: 412 [M]. Type species: *Psammodeslongicornis* W. Kirby, 1819, by monotypy. Status: valid genus in Pimeliinae: Sepidiini: Molurina.

*Psammodophysis* Péringuey, 1899: 296 [F]. Type species: *Psammodophysisprobes* Péringuey, 1899, by subsequent designation (Kamiński et al. 2019b: 31). Status: junior synonym of *Psammodes* W. Kirby, 1819 in Pimeliinae: Sepidiini: Molurina. Synonymy: Gebien (1910a: 154).

*Psammoeca* Agassiz, 1846b: 311 [F]. Type species [automatic]: *Microderalucida* Solier, 1835, by subsequent designation (Gebien 1937a: 611). Status: junior synonym of *Psammoica* Solier, 1835 in Pimeliinae: Tentyriini. Note: unjustified emendation of *Psammoica* Solier, 1835, not in prevailing usage.

*Psammogaster* Koch, 1953e: 237 [F]. Type species: *Psammogastermalani* Koch, 1953, by original designation. Status: valid genus in Blaptinae: Opatrini: Stizopodina.

*Psammoica* Solier, 1835b: 307 [F]. Type species: *Microderalucida* Solier, 1835, by subsequent designation (Gebien 1937a: 611). Status: valid genus in Pimeliinae: Tentyriini.

*Psammolophus* Koch, 1953f: 154 [M]. Type species: *Psammodesacuticosta* Fairmaire, 1884, by original designation. Status: valid subgenus of *Psammophanes* Lesne, 1922 in Pimeliinae: Sepidiini: Molurina.

*Psammophanes* Lesne, 1922: 690 [M]. Type species: *Moluriscatenata* Reiche, 1850, by original designation. Status: valid genus and subgenus in Pimeliinae: Sepidiini: Molurina.

*Psammophrynopsis* Koch, 1953f: 157 [F]. Type species: *Phrynocolusfrommi* Wilke, 1921, by original designation. Status: valid subgenus of *Psammophanes* Lesne, 1922 in Pimeliinae: Sepidiini: Molurina.

*Psammophrynus* Koch, 1953f: 146 [M]. Type species: *Psammophanesjokli* Koch, 1953, by original designation. Status: valid subgenus of *Psammophanes* Lesne, 1922 in Pimeliinae: Sepidiini: Molurina.

*Psammoryssus* Kolbe, 1886: 289 [M]. Type species: *Psammoryssustitanus* Kolbe, 1886, by monotypy. Status: valid genus in Pimeliinae: Sepidiini: Molurina.

*Psammostretus* Koch, 1953f: 145 [M]. Type species: *Psammodesbisbicostatus* Gebien, 1910, by original designation. Status: valid subgenus of *Psammophanes* Lesne, 1922 in Pimeliinae: Sepidiini: Molurina.

*Psammotopulus* Endrödy-Younga, 1996: 15, 29 [M]. Type species: *Caenocrypticuspenrithae* Endrödy-Younga, 1996, by original designation. Status: valid subgenus of *Caenocrypticus* Gebien, 1920 in Pimeliinae: Caenocrypticini.

*Psammotyria* Koch, 1953f: 137 [F]. Type species: *Psammodesertli* Kolbe, 1904, by original designation. Status: valid genus in Pimeliinae: Sepidiini: Molurina.

*Psammotyriopsis* Koch, 1953f: 144 [F]. Type species: *Psammophanesbredoi* Koch, 1953, by original designation. Status: valid subgenus of *Psammophanes* Lesne, 1922 in Pimeliinae: Sepidiini: Molurina.

*Psaryphis* Erichson, 1843: 241 [F]. Type species: *Psaryphisnana* Erichson, 1843, by monotypy. Status: valid genus in Pimeliinae: Adelostomini.

*Psaryphulum* Koch, 1952b: 32 [N]. Type species: *Adelostomaabbreviatum* Haag-Rutenberg, 1875, by original designation. Status: valid subgenus of *Adelostoma* Duponchel, 1827 in Pimeliinae: Adelostomini.

*Psectes* Hesse, 1935: 572 [M]. Type species: *Psectesbechuanus* Hesse, 1935, by original designation. Status: valid genus in Blaptinae: Pedinini: Helopinina.

*Psectrapus* Solier, 1848: 153, 213 [M]. Type species: *Psectrapusbipartitus* Solier, 1848, by original designation. Status: valid genus in Blaptinae: Platynotini: Eurynotina.

*Psectrascelis* Solier, 1836: 307, 311 [F]. Type species: *Nycteliapilipes* Guérin-Méneville, 1834, by subsequent designation (Gebien 1937a: 750). Status: valid genus in Pimeliinae: Nycteliini. Note: the First Reviser (*Psectrascelis* Solier, 1836 versus *Cerostena* Solier, 1836) is Fairmaire (1876: 356).

*Psectropus* Gemminger in Gemminger and Harold, 1870: 1911 [M]. Type species [automatic]: *Psectrapusbibartitus* Solier, 1848, by original designation. Status: junior synonym of *Psectrapus* Solier, 1848 in Blaptinae: Platynotini: Eurynotina. Note: unjustified emendation of *Psectrapus* Solier, 1848, not in prevailing usage.

*Pselaphidion* Gebien, 1921b: 29, 229 [N]. Type species: *Platydemamacularia* Gemminger, 1870, by subsequent designation (Gebien 1940: 405). Status: junior synonym of *Stomylus* Fåhraeus, 1870 in Diaperinae: Diaperini: Diaperina. Synonymy: Koch (1953g: 23). Note: the alternative original spelling *Pselaphidium*, used by Gebien (1921b: 28, 29), was rejected by Gebien (1940: 405) who acted as the First Reviser (ICZN 1999, Article 24.2.4).

*Pseudabax* Kraatz, 1880b: 107 [M]. Type species: *Pseudabaxformosus* Kraatz, 1880, by subsequent designation (Gebien 1942a: 308). Status: valid genus in Stenochiinae: Cnodalonini.

*Pseudadelium* Kaszab, 1982b: 234 [N]. Type species: *Adeliumpustulosum* Fauvel, 1904, by original designation. Status: junior synonym of *Neoadelium* Carter, 1908 in Lagriinae: Adeliini. Synonymy: Matthews (1998: 781).

*Pseudadrus* Fairmaire, 1897g: 39 [M]. Type species: *Hadrusscaphoides* Marseul, 1876, by monotypy. Status: junior synonym of *Phelopatrum* Marseul, 1876 in Blaptinae: Opatrini: Opatrina. Synonymy: Reichardt (1936: 90).

*Pseudalymon* Ardoin, 1969c: 543, 544 [M]. Type species: *Asthenochirusfoveolatus* Péringuey, 1899, by monotypy. Status: valid genus in Tenebrioninae: Amarygmini.

*Pseudamarsenes* Ardoin, 1955: 142 [M]. Type species: *Amarsenesviridistriatus* Fairmaire, 1894, by original designation. Status: valid genus in Stenochiinae: Cnodalonini.

*Pseudamarygmus* Pic, 1915d: 9 [M]. Type species: *Pseudamarygmustestaceipes* Pic, 1915, by subsequent designation (Gebien 1948: 509). Status: junior synonym of *Amarygmus* Dalman, 1823 in Tenebrioninae: Amarygmini. Synonymy: Bremer (2001a: 67).

*Pseudamblyptera* Pierre, 1985: 295 [F]. Type species: *Pimeliafrigida* Escalera, 1925, by original designation. Status: valid subgenus of *Pimelia* Fabricius, 1775 in Pimeliinae: Pimeliini.

*Pseudamenophis* Pic, 1916: 13 [M]. Type species: *Pseudamenophisaeneus* Pic, 1916 (= *Amenophisepipleuralis* Gebien, 1904), by monotypy. Status: junior synonym of *Amenophis* J. Thomson, 1858 in Stenochiinae: Cnodalonini. Synonymy: Ardoin (1962a: 64).

*Pseudanaedus* Gebien, 1921b: 107, 111 [M]. Type species: *Pseudanaedusbiangulatus* Gebien, 1921, by subsequent designation (Gebien 1941: 820). Status: valid genus in Lagriinae: Goniaderini.

*Pseudandrosus* Kulzer, 1951b: 486 [M]. Type species: *Chariothecaneomidina* Fairmaire, 1881, by original designation. Status: valid genus in Stenochiinae: Cnodalonini.

*Pseudanemia* Wollaston, 1864: 492 [F]. Type species: *Pseudanemiabrevicollis* Wollaston, 1864, by monotypy. Status: valid subgenus of *Cheirodes* Gené, 1839 in Tenebrioninae: Melanimonini.

*Pseudanopidium* Dajoz, 1974: 431, 434 [N]. Type species: *Pseudanopidiumpunctatum* Dajoz, 1974, by original designation. Status: valid genus in Diaperinae: Gnathidiini: Anopidiina.

*Pseudapocrypha* Champion, 1886: 260 [F]. Type species: *Pseudapocryphalacordairii* Champion, 1886, by monotypy. Status: valid genus in Tenebrioninae: Apocryphini.

*Pseudapsida* Kulzer, 1961: 219 [F]. Type species: *Pseudapsidabrasiliensis* Kulzer, 1961, by original designation. Status: junior synonym of *Paniasis* Champion, 1886 in Diaperinae: Diaperini: Diaperina. Synonymy: Ferrer and Ødegaard (2005: 637).

*Pseudasida* Fairmaire, 1895b: 444 [F]. Type species: *Pseudasidaobesa* Fairmaire, 1895, by monotypy. Status: valid genus in Pimeliinae: Asidini.

*Pseudeba* Blackburn, 1903: 119 [F]. Type species: *Pseudebanovica* Blackburn, 1903, by monotypy. Status: valid genus in Tenebrioninae: Palorini. Note: transferred from “Tenebrionoidea: Colydiidae” by Carter and Zeck (1937: 194).

*Pseudeleodes* Blaisdell, 1909: 146 [M]. Type species: *Eleodesgranosa* J.L. LeConte, 1866, by monotypy. Status: valid subgenus of *Eleodes* Eschscholtz, 1829 in Blaptinae: Amphidorini.

*Pseudemmallus* Koch, 1956a: 355 [M]. Type species: *Pseudemmallusaspericollis* Koch, 1956, by monotypy. Status: valid genus in Blaptinae: Dendarini: Melambiina.

*Pseudephalus* Casey, 1924: 333 [M]. Type species: *Pseudephalusbrevicornis* Casey, 1924, by original designation. Status: junior synonym of *Ephalus* J.L. LeConte, 1862 in Blaptinae: Opatrini: Opatrina. Synonymy: Lumen et al. (2020: 344).

*Pseudesarcus* Champion, 1913: 115 [M]. Type species: *Pseudesarcusvillosus* Champion, 1913, by original designation. Status: valid genus in Lagriinae: incertae sedis. Note: originally described in Mycetophagidae, transferred to Tenebrionidae: Lagriinae by Lawrence and Newton (1995: 886).

*Pseudethas* Fairmaire, 1896a: 57 [M]. Type species: *Pseudethasquadraticeps* Fairmaire, 1896, by monotypy. Status: valid genus and subgenus in Pimeliinae: Stenosini: Dichillina.

*Pseudethas* Fairmaire, 1898c: 477 [M]. Type species: *Pseudethaslongiceps* Fairmaire, 1898, by monotypy. Status: senior synonym of *Anethas* Jakobson, 1924 in Pimeliinae: Stenosini: Stenosina. Note: junior homonym of *Pseudethas* Fairmaire, 1896 [Coleoptera: Tenebrionidae: Pimeliinae: Stenosini: Dichillina].

*Pseudeucyrtus* Pic, 1916e: 14 [M]. Type species: *Pseudeucyrtusniasensis* Pic, 1916 (= *Cleomisviolaceipes* Fairmaire, 1892), by subsequent designation (Gebien 1942a: 308). Status: junior synonym of *Cleomis* Fairmaire, 1892 in Stenochiinae: Cnodalonini. Synonymy: Kaszab (1983b: 382). Note: we act as First Revisers and reject the alternative original spelling *Pseudocyrtus*, used by Pic (1916e: 14), in order to avoid homonymy with *Pseudocyrtus* Salmon, 1956 [Collembola].

*Pseudeumolpus* Kraatz, 1880b: 111 [M]. Type species: *Pseudeumolpusbicolor* Kraatz, 1880, by subsequent designation (Löbl et al. 2008b: 345). Status: junior synonym of *Phaedis* Pascoe, 1866 in Stenochiinae: Cnodalonini. Synonymy: Gebien (1914d: 71).

*Pseudeuthriptera* Bogatchev & Kryzhanovsky, 1955: 240 [F]. Type species: *Trigonoscelisuzboica* Bogatchev & Kryzhanovsky, 1955, by monotypy. Status: junior synonym of *Sternoplax* Frivaldszky, 1890 in Pimeliinae: Pimeliini. Synonymy: Skopin (1964: 395).

*Pseudeutrapela* Pic, 1952e: 110 [F]. Type species: *Pseudeutrapelanigra* Pic, 1952, by monotypy. Status: valid genus in Lagriinae: Lagriini: Statirina.

*Pseudhadrus* Kolbe, 1910: 31 [M]. Type species: *Pseudhadrusseriatus* Kolbe, 1910, by subsequent designation (Gebien 1941: 332). Status: valid genus in Stenochiinae: Cnodalonini.

*Pseudhelops* Guérin-Méneville, 1841: 124 [M]. Type species: *Pseudhelopstuberculatus* Guérin-Méneville, 1841, by monotypy. Status: valid genus in Tenebrioninae: Heleini: incertae sedis. Note: transferred from Titaenini by Matthews and Lawrence (2019: 628).

*Pseudimmedia* Kulzer, 1958b: 213 [F]. Type species: *Pseudimmediafreyi* Kulzer, 1958, by original designation. Status: valid genus in Stenochiinae: Cnodalonini.

*Pseudisopus* Kulzer, 1957: 254 [M]. Type species: *Pseudisopusgressitti* Kulzer, 1957, by original designation. Status: valid genus in Stenochiinae: Cnodalonini.

*Pseudobasides* Pic, 1916c: 2 [M]. Type species: *Pseudobasidescornutus* Pic, 1916, by subsequent designation (Gebien 1940: 419). Status: valid genus in Diaperinae: Diaperini: Diaperina.

*Pseudobates* Fairmaire, 1882a: 231 [M]. Type species: *Nyctobatescoracinus* Fairmaire, 1882 (= *Nyctobatessubrobustus* Motschulsky, 1872), by monotypy. Status: junior synonym of *Promethis* Pascoe, 1869 in Stenochiinae: Cnodalonini. Synonymy: Kolbe (1900: 74, with *Setenis* Motschulsky, 1872, a junior synonym of *Promethis* Pascoe, 1869).

*Pseudoblapida* Pic, 1917: 18 [F]. Type species: *Blapidaboliviensis* Pic, 1912, by monotypy. Status: valid genus and subgenus in Stenochiinae: Cnodalonini.

*Pseudoblaps* Guérin-Méneville, 1834: 28 [F]. Type species: *Pseudoblapssubstriata* Guérin-Méneville, 1834, by subsequent designation (Hope 1841: 124). Status: valid genus in Blaptinae: Platynotini: Platynotina.

*Pseudobolbophanes* Kulzer, 1954a: 29 [M]. Type species: *Pseudobolbophanesmalaisei* Kulzer, 1954, by original designation. Status: junior synonym of *Bolbophanes* Carter, 1913 in Tenebrioninae: Heleini: Cyphaleina. Synonymy: Matthews (1992: 490).

*Pseudobradymerus* Pic, 1926b: 16 [M]. Type species: *Bradymerussimplicithorax* Pic, 1926, by monotypy. Status: junior synonym of *Bradymerus* Perroud & Montrouzier, 1865 in Stenochiinae: Cnodalonini. Synonymy: Gebien (1939: 751).

*Pseudobyrsax* Kaszab, 1982b: 214 [M]. Type species: *Pseudobyrsaxcurtus* Kaszab, 1982, by original designation. Status: valid genus in Lagriinae: Adeliini.

*Pseudocaedius* Blackburn, 1890a: 91 [M]. Type species: *Pseudocaediussquamosus* Blackburn, 1890 (= *Trigonotarsusaustralis* Hope, 1843), by monotypy. Status: junior synonym of *Sobas* Pascoe, 1863 in Blaptinae: Opatrini: Opatrina. Synonymy: Carter (1921: 307).

*Pseudocaedius* G.S. Medvedev, 1966: 98 [M]. Type species: *Pseudocaediuskiseritzkii* G.S. Medvedev, 1966, by original designation. Status: senior synonym of *Asiocaedius* G.S. Medvedev & Nepesova, 1985 in Blaptinae: Opatrini: Ammobiina. Note: junior homonym of *Pseudocaedius* Blackburn, 1890 [Coleoptera: Tenebrionidae: Blaptinae: Opatrini: Ammobiina].

*Pseudocaelophus* Pic, 1922d: 28 [M]. Type species: *Strongyliumdifforme* Pic, 1922, by monotypy. Status: junior synonym of *Leprocaulus* Fairmaire, 1896 in Stenochiinae: Cnodalonini. Synonymy: Bečvář and Purchart (2008: 39).

*Pseudocamaria* Bates, 1879a: 287 [F]. Type species: *Camariaalternata* Fairmaire, 1875, by original designation. Status: valid genus in Stenochiinae: Cnodalonini.

*Pseudocamarimena* Pic, 1923e: 21 [F]. Type species: *Pseudocamarimenastriata* Pic, 1923, by monotypy. Status: junior synonym of *Pigeus* Gebien, 1919 in Stenochiinae: Cnodalonini. Synonymy: Kaszab (1984: 363).

*Pseudocasnonidea* Borchmann, 1936: 240, 489 [F]. Type species: *Pseudocasnonideaceylanica* Borchmann, 1936, by original designation. Status: valid genus in Lagriinae: Lagriini: Statirina.

*Pseudochariotheca* Pic, 1934c: 32 [F]. Type species: *Pseudochariothecaminutissima* Pic, 1934, by original designation. Status: junior synonym of *Steneucyrtus* Fairmaire, 1896 in Stenochiinae: Cnodalonini. Synonymy: Kaszab (1983a: 136).

*Pseudochillus* Fouquè, 2015: 226, 240 [M]. Type species: *Indochillusbangaloreanus* Kaszab, 1981, by original designation. Status: valid genus and subgenus in Pimeliinae: Stenosini: Dichillina.

*Pseudochrysomela* Pic, 1925a: 7 [F]. Type species: *Pseudeumolpusseriatoporus* Fairmaire, 1888, by monotypy. Status: valid genus in Stenochiinae: Cnodalonini. Note: the older name *Pseudochrysomela* Voet, 1806 [Coleoptera: Erotylidae] was published in a work that did not include consistent application of binominal nomenclature and is therefore unavailable (ICZN 1999, Article 11.4).

*Pseudocilibe* Kaszab, 1982b: 231 [F]. Type species: *Celibeasidaeformis* Fauvel, 1904, by original designation. Status: valid genus in Lagriinae: Adeliini. Note: we act as First Revisers and reject the alternative original spelling *Pseudocylibe*, used by Kaszab (1982b: 291).

*Pseudocistela* Blackburn, 1891: 316 [F]. Type species: *Pseudocistelaovalis* Blackburn, 1891, by monotypy. Status: senior synonym of *Neocistela* Borchmann, 1909 in Alleculinae: Alleculini: Alleculina. Note: junior homonym of *Pseudocistela* Crotch, 1874 [Coleoptera: Tenebrionidae: Alleculinae: Alleculini: Gonoderina].

*Pseudocistela* Crotch, 1874: 108 [F]. Type species: *Cistelabrevis* Say, 1824, by subsequent designation (Novák and Pettersson 2008: 327). Status: valid genus in Alleculinae: Alleculini: Gonoderina.

*Pseudocistelopsis* Novák, 2018b: 176 [M]. Type species: *Pseudocistelopsisjakli* Novák, 2018, by original designation. Status: valid genus in Alleculinae: Alleculini: Alleculina.

*Pseudocoelus* Casey, 1908: 152 [M]. Type species: *Coeluspacificus* Fall, 1897, by subsequent designation (Doyen 1976: 608). Status: junior synonym of *Coelus* Eschscholtz, 1829 in Pimeliinae: Coniontini. Synonymy: Blaisdell (1919: 322).

*Pseudocolparthrum* Borchmann, 1916b: 230, 236 [N]. Type species: *Colparthrumcalcaratum* Champion, 1889, by subsequent designation (Borchmann 1936: 452). Status: valid subgenus of *Colparthrum* Kirsch, 1866 in Lagriinae: Lagriini: Statirina.

*Pseudoderiles* Gebien, 1928: 169, 181 [M]. Type species: *Pseudoderilesdentipennis* Gebien, 1928, by monotypy. Status: valid genus in Stenochiinae: Cnodalonini.

*Pseudoderosphaerus* Pic, 1922d: 24 [M]. Type species: *Leprocaulusrotundicollis* Pic, 1922, by monotypy. Status: junior synonym of *Leprocaulus* Fairmaire, 1896 in Stenochiinae: Cnodalonini. Synonymy: Kaszab (1983c: 177).

*Pseudodiaphanidus* Bogatchev, 1950: 234 [M]. Type species: *Diaphanidusrobustus* Bogatchev, 1950, by monotypy. Status: valid subgenus of *Diaphanidus* Reitter, 1900 in Pimeliinae: Erodiini.

*Pseudoelongasida* Escalera, 1922c: 173 [F]. Type species: *Asidasilvestrei* Escalera, 1922, by subsequent designation (F. Soldati 2008: 33). Status: junior synonym of *Elongasida* Escalera, 1906 in Pimeliinae: Asidini. Synonymy: Pérez-Vera et al. (2017: 3).

*Pseudoenanea* Pic, 1924a: 25 [F]. Type species: *Pseudoenanearobusta* Pic, 1924, by monotypy. Status: valid genus in Diaperinae: Gnathidiini: Gnathidiina.

*Pseudogena* Fairmaire, 1899e: 539 [F]. Type species: *Pseudogenapolyzona* Fairmaire, 1899, by monotypy. Status: valid genus in Stenochiinae: Stenochiini.

*Pseudognaptorina* Kaszab, 1977c: 250 [F]. Type species: *Pseudognaptorinanepalica* Kaszab, 1977, by original designation. Status: valid genus in Blaptinae: Blaptini: Gnaptorinina.

†*Pseudohelops* Haupt, 1950: 130 [M]. Type species: *Pseudohelopsgroenlandicus* Haupt, 1950, by monotypy. Status: valid genus in Stenochiinae: incertae sedis. Note: combined description of new genus-group taxon and new species (ICZN 1999, Article 13.4); described from Middle Paleocene deposits (Greenland).

*Pseudohymenalia* Novák, 2008b: 213 [F]. Type species: *Pseudohymenaliayunnanica* Novák, 2008, by original designation. Status: valid genus in Alleculinae: Alleculini: Gonoderina.

*Pseudolagria* Champion, 1917: 218 [F]. Type species: *Pseudolagriamutabilis* Champion, 1917, by original designation. Status: valid genus in Lagriinae: Lagriini: Lagriina.

*Pseudolamus* Fairmaire, 1874: 388 [M]. Type species: *Pseudolamusseriatoporus* Fairmaire, 1874, by monotypy. Status: valid genus in Blaptinae: Opatrini: Neopachypterina.

*Pseudoleichenum* Ardoin, 1972: 205 [N]. Type species: *Pseudoleichenumbenoiti* Ardoin, 1972, by monotypy. Status: valid genus in Blaptinae: Opatrini: Ammobiina.

*Pseudolyprops* Fairmaire, 1882a: 236 [M]. Type species: *Pseudolypropsdilaticollis* Fairmaire, 1882, by monotypy. Status: valid genus in Lagriinae: Goniaderini.

*Pseudomachla* Wilke, 1922: 260 [F]. Type species [automatic]: *Opatrumvillosum* G.-A. Olivier, 1795, by subsequent designation (R. Lucas 1920: 386). Status: junior synonym of *Machla* Herbst, 1799 in Pimeliinae: Asidini. Note: unnecessary replacement name for *Machla* Herbst, 1799.

*Pseudomorocaulus* Pic, 1915c: 6 [M]. Type species: *Pseudomorocaulusrufonotatus* Pic, 1915, by monotypy. Status: valid genus in Alleculinae: incertae sedis.

*Pseudonautes* Fairmaire, 1892c: 52 [M]. Type species: *Pseudonautesvagevittatus* Fairmaire, 1892, by subsequent designation (R. Lucas 1920: 551). Status: valid genus in Stenochiinae: Cnodalonini.

*Pseudonomus* Fairmaire, 1884a: 510 [M]. Type species: *Pseudonomusdermestiformis* Fairmaire, 1884, by monotypy. Status: junior synonym of *Ammodonus* Mulsant, 1859 in Blaptinae: Opatrini: Ammobiina. Synonymy: Gebien (1939: 470).

*Pseudonotocorax* Iwan, 1997: 255, 269 [M]. Type species: *Pseudonotocoraxmroczkowskii* Iwan, 1997, by original designation. Status: valid genus in Blaptinae: Platynotini: Platynotina.

*Pseudoogeton* Masumoto, 1989b: 304 [M]. Type species: *Plesiophthalmusamplipennis* Fairmaire, 1897, by original designation. Status: valid genus in Tenebrioninae: Amarygmini.

*Pseudopachyscelis* Skopin, 1968a: 99 [F]. Type species: *Trigonoscelispygmaea* Ménétriés, 1849, by original designation. Status: valid genus in Pimeliinae: Pimeliini. Note: first proposed by Skopin (1962: 225) without a type species originally designated.

*Pseudoparablops* Heyden, 1908: 132 [M]. Type species: *Parablopssardiniensis* Allard, 1877, by monotypy. Status: junior synonym of *Allardius* Ragusa, 1898 in Tenebrioninae: Helopini: Helopina. Synonymy: Reitter (1922b: 167).

*Pseudopatrum* Sharp, 1886: 406 [N]. Type species: *Pseudopatrumsordidum* Sharp, 1886, by subsequent designation (Watt 1992: 30). Status: valid genus in Lagriinae: Adeliini.

*Pseudopedinus* Ardoin, 1969d: 178 [M]. Type species: *Pseudopedinuslaosensis* Ardoin, 1969, by original designation. Status: valid subgenus of *Loensus* R. Lucas, 1920 in Blaptinae: Pedinini: Pedinina.

*Pseudopenthicinus* Bogatchev, 1972: 628 [M]. Type species: *Penthicusmedvedevi* Bogatchev, 1972, by original designation. Status: valid subgenus of *Penthicus* Faldermann, 1836 in Blaptinae: Opatrini: Opatrina.

*Pseudoperichilus* Pic, 1921d: 21 [M]. Type species: *Pseudoperichilusolivaceus* Pic, 1921, by monotypy. Status: valid genus in Stenochiinae: Cnodalonini.

*Pseudophthora* Kaszab, 1970a: 267 [F]. Type species: *Pseudophthoralaeana* Kaszab, 1970, by original designation. Status: valid genus and subgenus in Phrenapatinae: Penetini.

*Pseudopigeus* Kaszab, 1984: 355, 359 [M]. Type species: *Pseudopigeusunidentatus* Kaszab, 1984, by original designation. Status: valid genus in Stenochiinae: Cnodalonini.

*Pseudopimelia* Gebler, 1859: 473 [F]. Type species: *Lasiostolavariolaris* Gebler, 1841, by monotypy. Status: junior synonym of *Lasiostola* Dejean, 1834 in Pimeliinae: Pimeliini. Synonymy: **new synonym** [YB]. Note: *Pseudopimelia* Gebler, 1859 has been forgotten in the literature; its type species is currently included in the nominotypical subgenus of *Lasiostola* Dejean, 1834 and for that reason Gebler’s name is considered a new junior synonym of *Lasiostola*.

*Pseudopimelia* Motschulsky, 1860c: 536 [F]. Type species: *Pterocomatuberculata* Motschulsky, 1845, by subsequent designation (Skopin 1974b: 159). Status: senior synonym of *Subpterocoma* Bouchard & Bousquet, **nom. nov.** in Pimeliinae: Pimeliini. Note: junior homonym of *Pseudopimelia* Gebler, 1859 [Coleoptera: Tenebrionidae: Pimeliinae: Pimeliini].

*Pseudoplanasida* Escalera, 1921: 360 [F]. Type species: *Asidapygmaea* Rosenhauer, 1856, by subsequent designation (F. Soldati 2008: 33). Status: junior synonym of *Gracilasida* Escalera, 1905 in Pimeliinae: Asidini. Synonymy: Viñolas and Cartagena (2005: 192, with *Planasida* Escalera, 1907, a junior synonym of *Gracilasida* Escalera, 1905).

*Pseudoplatyope* Pierre, 1964: 867, 873 [F]. Type species: *Storthocnemisantoinei* Español, 1951, by original designation. Status: valid genus in Pimeliinae: Pimeliini.

*Pseudopodhomala* Schuster, 1938: 88 [F]. Type species: *Pseudopodhomalagabrieli* Schuster, 1938, by monotypy. Status: valid genus in Pimeliinae: Pimeliini.

*Pseudopodhomalina* Kaszab, 1960a: 22 [F]. Type species: *Diesiacostifera* C.O. Waterhouse, 1889, by original designation. Status: junior synonym of *Pseudopodhomala* Schuster, 1938 in Pimeliinae: Pimeliini. Synonymy: Kwieton (1982: 35).

*Pseudopraeugena* De Moor, 1970: 8,40 [F]. Type species: *Pseudopraeugenarufa* De Moor, 1970, by original designation. Status: valid genus in Tenebrioninae: Praeugenini.

*Pseudoprobaticus* Nabozhenko, 2001b: 513 [M]. Type species: *Helopsgranipennis* Allard, 1876, by original designation. Status: valid genus in Tenebrioninae: Helopini: Cylindrinotina.

*Pseudoprosodes* Reitter, 1909a: 120 [M]. Type species: *Prosodestransfuga* Reitter, 1893, by original designation. Status: junior synonym of *Prosodes* Eschscholtz, 1829 in Blaptinae: Blaptini: Prosodina. Synonymy: Skopin (1960a: 46).

*Pseudopterocoma* Skopin, 1974b: 145 [F]. Type species: *Pterocomatrapezicollis* Skopin, 1974, by original designation. Status: valid subgenus of *Pterocoma* Dejean, 1834 in Pimeliinae: Pimeliini.

*Pseudorozonia* Bouchard & Bousquet, **new subgenus** [F]. Type species: *Rozoniaconophthalma* Koch, 1944, by **present designation**. Status: valid subgenus of *Rozonia* Fairmaire, 1888 in Pimeliinae: Tentyriini. Koch (1944a: 162) introduced the new subgenus name *Pseudorozonia* for three nominal species, but unfortunately did not designate a type species; the subgenusPseudorozonia, which has been treated as valid since 1944, is therefore unavailable (ICZN 1999, Article 13.3); we hereby make the name available by selecting *Rozoniaconophthalma* Koch, 1944 as type species and referring to Koch (1944a: 162) for the character states that characterise and differentiate *Anemiadena*.

*Pseudortheolus* Freude, 1968: 110 [M]. Type species: *Epitragusminutissimus* Fairmaire, 1892, by original designation. Status: valid genus in Pimeliinae: Epitragini.

*Pseudoscaphidema* Pic, 1926c: 2 [F]. Type species: *Pseudoscaphidemarufonotata* Pic, 1926, by monotypy. Status: valid genus in Diaperinae: Scaphidemini.

*Pseudoscotobius* Kulzer, 1955a: 384, 393 [M]. Type species: *Emmalloderastrangulata* Fairmaire, 1905, by original designation. Status: junior synonym of *Phrynocarenum* Gebien, 1928 in Tenebrioninae: Phrynocarenini. Synonymy: Marcuzzi (1976: 117).

*Pseudoselinus* Iwan, 2002a: 48, 94 [M]. Type species: *Eurynotuspunctatostriatus* Gerstaecker, 1854, by original designation. Status: valid subgenus of *Upembarus* Koch, 1956 in Blaptinae: Platynotini: Platynotina.

*Pseudoseriscius* Español, 1950: 125 [M]. Type species: *Pedinuspruinosus* Dufour, 1820, by original designation. Status: valid genus and subgenus in Diaperinae: Crypticini.

*Pseudostene* Wollaston, 1861: 247 [F]. Type species: *Pseudosteneangusta* Wollaston, 1861, by subsequent designation (Löbl et al. 2008b: 317). Status: junior synonym of *Phtora* Germar, 1836 in Diaperinae: Phaleriini. Synonymy: Escalera (1914: 347).

*Pseudosternoplax* Skopin, 1973: 110, 141 [F]. Type species: *Trigonoscelislacerta* Bates, 1879, by original designation. Status: valid subgenus of *Sternoplax* Frivaldszky, 1890 in Pimeliinae: Pimeliini.

*Pseudostira* Fairmaire, 1903c: 213 [F]. Type species: *Pseudostiralaevipennis* Fairmaire, 1903, by monotypy. Status: valid genus in Lagriinae: Lagriini: Statirina.

*Pseudostorthocnemis* Gridelli, 1952: 83 [F]. Type species: *Storthocnemispatrizii* Gridelli, 1933, by original designation. Status: valid genus in Pimeliinae: Pimeliini.

*Pseudostrongylium* Kraatz, 1880b: 114 [N]. Type species: *Pseudostrongyliumsemperi* Kraatz, 1880, by subsequent designation (Kaszab 1977b: 11). Status: junior synonym of *Lophocnemis* Mäklin, 1867 in Stenochiinae: Stenochiini. Synonymy: Gebien (1948: 543).

*Pseudotalpophila* Reitter, 1900c: 95 [F]. Type species: *Thalpophilaplicifrons* Wollaston, 1864, by subsequent designation (Gebien 1937a: 641). Status: valid subgenus of *Hegeter* Latreille, 1802 in Pimeliinae: Tentyriini.

*Pseudothinobatis* Bouchard & Bousquet, **new genus** [F]. Type species: *Thinobatisohausi* Kulzer, 1956, by **present designation**. Status: valid genus in Pimeliinae: Epitragini. Note: Freude (1960b: 32) introduced the new genus name *Pseudothinobatis* for two nominal species, but unfortunately did not designate a type species; the genus *Pseudothinobatis*, which has been treated as valid since 1960, is therefore unavailable (ICZN 1999, Article 13.3); we hereby make the name available by selecting *Thinobatisohausi* Kulzer, 1956 as type species and referring to Freude (1960b: 32) for the character states that characterise and differentiate *Pseudothinobatis*.

*Pseudothryoneus* Pic, 1921b: 12 [M]. Type species: *Pseudothryoneusbicoloripes* Pic, 1921, by monotypy. Status: valid genus in Stenochiinae: Cnodalonini.

*Pseudotocerus* Champion, 1888: 383 [M]. Type species: *Stenochialongipes* P.H. Lucas, 1859, by subsequent designation (Gebien 1948: 542). Status: valid genus in Stenochiinae: Stenochiini.

*Pseudotrichoplatyscelis* Kaszab, 1960a: 83, 111 [F]. Type species: *Platynoscelisbadakschanica* Kaszab, 1960 (= *Bioramixlapidicola* Kaszab, 1940), by original designation. Status: junior synonym of *Trichoplatyscelis* Reinig, 1931 in Blaptinae: Platyscelidini. Synonymy: Egorov (1990: 402). Note: we act as First Revisers and reject the alternative original spellings *Pseudotrichoplatynoscelis* and *Pseudotrichoplatycelis*, used by Kaszab (1960a: 82, 83).

*Pseuduloma* Fairmaire, 1893b: 27 [N]. Type species: *Pseudulomacribricolle* Fairmaire, 1893 (= *Ulomimusindicus* Bates, 1873), by monotypy. Status: junior synonym of *Ulomimus* Bates, 1873 in Tenebrioninae: Ulomini. Synonymy: Gebien (1940: 770).

*Pseuduroplatopsis* Pic, 1913a: 16 [F]. Type species: *Borchmanniajavana* Pic, 1913, by **present designation**. Status: valid subgenus of *Borchmannia* Pic, 1912 in Lagriinae: Lagriini: Statirina.

*Psilachnopus* Reitter, 1901: 161 [M]. Type species: *Psilachnopuscribratellus* Reitter, 1901, by monotypy. Status: junior synonym of *Philhammus* Fairmaire, 1871 in Pimeliinae: Cnemeplatiini: Cnemeplatiina. Synonymy: Gebien (1938a: 420), Löbl and Smetana (2010: 30).

*Psilocastus* Ardoin, 1963a: 149 [M]. Type species: *Psilocastusletestui* Ardoin, 1963, by original designation. Status: valid genus in Tenebrioninae: Amarygmini. Note: *Psilocastus* was used earlier by Ardoin (1962b: 970) without designation of a type species and is therefore unavailable from that date.

*Psilolaena* Heller, 1923: 70 [F]. Type species: *Psilolaenaschusteri* Heller, 1923, by monotypy. Status: junior synonym of *Laena* Dejean, 1821 in Lagriinae: Laenini. Synonymy: Schawaller (2001b: 34).

*Psilomera* Motschulsky, 1870: 400 [F]. Type species: *Pelecyphorusangulatus* J.L. LeConte, 1851, by monotypy. Status: junior synonym of *Stenomorpha* Solier, 1836 in Pimeliinae: Asidini. Synonymy: Bousquet in Bousquet et al. (2018: 74).

*Psilonesogena* Bates, 1879a: 305 [F]. Type species: *Psilonesogenahybrida* Bates, 1879, by monotypy. Status: valid genus in Stenochiinae: Stenochiini.

*Psis* Novák, 2019d: 71 [M]. Type species: *Psisnanensis* Novák, 2019, by original designation. Status: valid genus in Alleculinae: Alleculini: Alleculina.

*Psoroderes* Ardoin, 1962b: 969, 1017 [M]. Type species: *Psorodeshottentottus* Péringuey, 1899, by monotypy. Status: valid genus in Tenebrioninae: Amarygmini.

*Psorodes* Dejean, 1834: 189 [M]. Type species [automatic]: *Pimeliadentipes* Fabricius, 1787, by monotypy. Status: valid genus in Tenebrioninae: Amarygmini. Note: replacement name for *Acanthomera* Latreille, 1828.

*Psorophodes* Ardoin, 1963a: 83 [F]. Type species: *Pimeliaarmata* Herbst, 1799, by monotypy. Status: valid genus in Tenebrioninae: Amarygmini. Note: *Psorophodes* was used earlier by Ardoin (1962b: 969) without designation of a type species and is therefore unavailable from that date.

*Psydocamaria* Pic, 1923d: 17 [F]. Type species: *Psydocamariarobusta* Pic, 1923, by monotypy. Status: valid genus in Stenochiinae: Cnodalonini.

*Psydomorphus* Pic, 1921d: 24 [M]. Type species: *Psydomorphusdiversipes* Pic, 1921, by monotypy. Status: valid genus in Stenochiinae: Cnodalonini.

*Psydus* Pascoe, 1868: xii [M]. Type species: *Psydusplantaris* Pascoe, 1868, by monotypy. Status: valid genus in Stenochiinae: Cnodalonini.

*Pteraulus* Solier, 1848: 152, 200 [M]. Type species: *Pteraulussulcatipennis* Solier, 1848, by **present designation**. Status: junior synonym of *Helopinus* Solier, 1848 in Blaptinae: Pedinini: Helopinina. Synonymy: Koch (1958: 149).

*Pterelaeus* Gemminger in Gemminger and Harold, 1870: 1968 [M]. Type species [automatic]: *Pterohelaeuswalkerii* Brême, 1842, by subsequent designation (Gebien 1940: 1069). Status: junior synonym of *Pterohelaeus* Brême, 1842 in Tenebrioninae: Heleini: Heleina. Note: unjustified emendation of *Pterohelaeus* Brême, 1842, not in prevailing usage.

*Pterna* Reitter, 1884: 249 [F]. Type species: *Ernocharisauricoma* Reitter, 1884, by monotypy. Status: valid subgenus of *Mycetochara* Guérin-Méneville, 1827 in Alleculinae: Alleculini: Mycetocharina. Note: as mentioned by Bousquet et al. (2015: 138) this name was originally proposed as a synonym of *Mycetochares* Latreille, 1829 but was used as valid subsequently and adopted as the name of a taxon (e.g., Seidlitz 1896: 132, 137) before 1961; therefore, it is available and dates from its publication as a synonym (ICZN 1999, Article 11.6.1).

*Pterocoma* Dejean, 1834: 178 [F]. Type species: *Pimeliapiligera* Gebler, 1830, by monotypy. Status: valid genus and subgenus in Pimeliinae: Pimeliini.

*Pterocomodes* Reitter, 1901: 159 [M]. Type species: *Pterocomodesacutus* Reitter, 1901, by monotypy. Status: junior synonym of *Podhomala* Solier, 1836 in Pimeliinae: Pimeliini. Synonymy: Skopin (1962: 247).

*Pteroctenus* Kirsch, 1866: 193 [M]. Type species: *Pteroctenuspexus* Kirsch, 1866, by monotypy. Status: valid genus in Pimeliinae: Falsomycterini.

*Pterodes* Ardoin, 1963a: 97 [M]. Type species: *Hoplonyxasper* Péringuey, 1899, by original designation. Status: valid genus in Tenebrioninae: Amarygmini. Note: *Pterodes* was used earlier by Ardoin (1962: 970) without a type species designation and is therefore unavailable from that date.

*Pteroglymmius* Gebien, 1928: 219, 223 [M]. Type species: *Pteroglymmiuserotyloides* Gebien, 1928, by monotypy. Status: junior synonym of *Isaminas* Champion, 1887 in Stenochiinae: Cnodalonini. Synonymy: Doyen (1988: 301).

*Pterohelaeus* Brême, 1842b: 17, 27 [M]. Type species: *Pterohelaeuswalkerii* Brême, 1842, by subsequent designation (Gebien 1940: 1069). Status: valid genus in Tenebrioninae: Heleini: Heleina.

*Pterolasia* Solier, 1836: 9, 66 [F]. Type species: *Pterolasiasqualida* Solier, 1836, by subsequent designation (Hope 1841: 118). Status: valid genus in Pimeliinae: Pimeliini.

*Pteroselinus* Kamiński, 2015a: 92, 94 [M]. Type species: *Opatrinusinsularis* Mulsant & Rey, 1853, by original designation. Status: valid genus in Blaptinae: Platynotini: Platynotina.

*Pterostichula* Koch, 1952d: 224 [F]. Type species: *Pterostichulacalathoides* Koch, 1952, by original designation. Status: valid genus and subgenus in Pimeliinae: Sepidiini: Oxurina.

*Ptilonix* Allard, 1877: 62 [M]. Type species: *Helopsclavicrus* Marseul, 1876, by subsequent designation (Löbl et al. 2008a: 42). Status: junior synonym of *Misolampidius* Solsky, 1876 in Stenochiinae: Cnodalonini. Synonymy: Lewis (1894: 476).

*Pubamarygmus* Pic, 1915d: 8 [M]. Type species: *Pubamarygmusviridipennis* Pic, 1915, by subsequent designation (Gebien 1948: 510). Status: valid genus in Tenebrioninae: Amarygmini.

*Pubeirosoma* Pic, 1954: 257 [N]. Type species: *Isomiradiscoglabrata* Pic, 1954, by monotypy. Status: valid subgenus of *Isomira* Mulsant, 1856 in Alleculinae: Alleculini: Gonoderina.

*Pulposipes* Gemminger in Gemminger and Harold, 1870: 1974 [M]. Type species [automatic]: *Polposipusherculeanus* Solier, 1848, by original designation. Status: junior synonym of *Polposipus* Solier, 1848 in Stenochiinae: Cnodalonini. Note: unjustified emendation of *Polposipus* Solier, 1848, not in prevailing usage.

*Pumiliofossorum* Silvestro & Giraldo-Mendoza in Silvestro et al., 2015: 462, 470 [N]. Type species: *Pumiliofossorummoche* Silvestro & Flores, 2015, by original designation. Status: valid genus in Tenebrioninae: Scotobiini.

*Punctacula* Campbell, 1971: 112 [F]. Type species: *Punctaculahowdeni* Campbell, 1971, by original designation. Status: valid genus in Alleculinae: Alleculini: Alleculina.

*Pushtunillus* G.S. Medvedev, 1995a: 855, 865 [M]. Type species: *Dichillusskopini* G.S. Medvedev & Kabakov, 1995, by original designation. Status: valid subgenus of *Dichillus* Jacquelin du Val, 1860 in Pimeliinae: Stenosini: Dichillina.

*Pyanirygmus* Pic, 1915d: 9 [M]. Type species: *Pyanirygmuscorinthius* Pic, 1915, by monotypy. Status: valid subgenus of *Amarygmus* Dalman, 1823 in Tenebrioninae: Amarygmini.

*Pyanisia* Laporte, 1840: 235 [F]. Type species: *Helopsundatus* Fabricius, 1792 (= *Erotylusnebulosus* Fabricius, 1781), by subsequent designation (Lacordaire 1859b: 476). Status: junior synonym of *Cymatothes* Dejean, 1834 in Tenebrioninae: Amarygmini. Synonymy: Chevrolat (1847b: 643).

*Pycna* Fairmaire, 1894d: 68 [F]. Type species: *Pycnaaphodina* Fairmaire, 1894, by monotypy. Status: senior synonym of *Madagassa* Koch, 1950 in Phrenapatinae: Penetini. Note: redescribed as new by Fairmaire (1894b: 141); junior homonym of *Pycna* Amyot & Audinet-Serville, 1843 [Hemiptera].

*Pycnocerus* Westwood, 1841b: 67 [M]. Type species [automatic]: *Pachyloceruswestermanni* Hope, 1841, by monotypy. Status: valid genus and subgenus in Lagriinae: Pycnocerini. Note: replacement name for *Pachylocerus* Hope, 1841.

*Pycnochilus* C.O. Waterhouse, 1879b: 263 [M]. Type species: *Pycnochilusadvenus* C.O. Waterhouse, 1879, by monotypy. Status: valid genus in Phrenapatinae: Penetini.

*Pycnomorpha* Motschulsky, 1870: 398 [F]. Type species: *Pycnomorphacalifornica* Motschulsky, 1870, by monotypy. Status: valid subgenus of *Stenomorpha* Solier, 1836 in Pimeliinae: Asidini.

*Pycnonotida* Casey, 1912: 75, 89 [F]. Type species: *Microschatiainaequalis* J.L. LeConte, 1851, by original designation. Status: junior synonym of *Microschatia* Solier, 1836 in Pimeliinae: Asidini. Synonymy: K.W. Brown and Doyen (1992: 546).

*Pycnuloma* Fairmaire, 1896c: 99 [N]. Type species: *Pycnulomaraffrayi* Fairmaire, 1896, by monotypy. Status: valid genus in Tenebrioninae: Ulomini.

*Pygidiphorus* Mulsant, 1856b: [1, supplement] [M]. Type species: *Pygidiphoruscaroli* Mulsant, 1856, by monotypy. Status: valid genus in Tenebrionidae: incertae sedis. Note: included in a list of “Tenebrionidae*nomina dubia*” by Iwan et al. (2020: 475).

*Pygmaeodes* Koch, 1952d: 223 [M]. Type species: *Namibomodesrudebecki* Koch, 1952, by monotypy. Status: valid subgenus of *Palpomodes* Koch, 1952 in Pimeliinae: Sepidiini: Oxurina.

*Pyres* Champion, 1885: 100 [M]. Type species: *Pyresmetallicus* Champion, 1885 (= *Centronopusspeciosus* Pascoe, 1883), by subsequent designation (Gebien 1941: 336). Status: junior synonym of *Menechides* Motschulsky, 1872 in Tenebrioninae: Centronopini. Synonymy: Spilman (1962b: 3).

†*Pyrochalcaspis* Haupt, 1950: 113, 115 [F]. Type species: *Pyrochalcaspisgiseltalensis* Haupt, 1950, by original designation. Status: valid genus in Stenochiinae: incertae sedis. Note: described from Middle Eocene deposits (Germany).

*Pystelops* Gozis, 1910: 103 [M]. Type species: *Helopsmeridianus* Mulsant, 1854, by original designation. Status: valid subgenus of *Stenomax* Allard, 1876 in Tenebrioninae: Helopini: Cylindrinotina.

*Pythiopus* Koch, 1953e: 245 [M]. Type species: *Pythiopuscornutipectus* Koch, 1953, by original designation. Status: valid genus in Blaptinae: Dendarini: Dendarina.

*Pythonissus* Gistel, 1834: 21 [M]. Type species: *Helopsmorio* Fabricius, 1777 (= *Tenebrioatratus* Fabricius, 1775), by subsequent designation (Bousquet and Bouchard 2017: 132). Status: junior synonym of *Zophobas* Dejean, 1834 in Tenebrioninae: Tenebrionini. Synonymy: Bousquet and Bouchard (2017: 132).

*Quadrideres* Koch, 1956a: 189 [M]. Type species: *Anchophthalmusscutatus* Gerstaecker, 1871, by original designation. Status: junior synonym of *Glyptopteryx* Gebien, 1910 in Blaptinae: Platynotini: Platynotina. Synonymy: Kamiński (2015a: 91).

*Quadroncotus* Koch, 1954a: 40 [M]. Type species: *Oncotusirrepertus* Koch, 1954, by original designation. Status: valid subgenus of *Oncotus* Blanchard, 1845 in Blaptinae: Platynotini: Eurynotina.

*Raiboscelis* Allard, 1876a: 5 [F]. Type species: *Helopscorvinus* Küster, 1850, by subsequent designation (Gebien 1943: 415). Status: valid genus in Tenebrioninae: Helopini: Helopina. Note: nomen protectum (see Nabozhenko and Löbl 2009: 194); *Raiboscelis* is an incorrect subsequent spelling of the original spelling *Raibosceles* and is in prevailing usage; *Raiboscelis* is deemed to be the correct original spelling (ICZN 1999, Article 33.3.1), see Nabozhenko and Löbl (2009: 189).

*Raptor* Gistel, 1848a: ix, xi [M]. Type species [automatic]: *Melaphorusreichii* Guérin-Méneville, 1834, by monotypy. Status: junior synonym of *Melaphorus* Guérin-Méneville, 1834 in Pimeliinae: Evaniosomini. Note: unnecessary replacement name for *Melaphorus* Guérin-Méneville, 1834.

*Rasphytus* Kulzer, 1956a: 639 [M]. Type species: *Rasphytusfreyi* Kulzer, 1956, by original designation. Status: valid subgenus of *Apentanodes* Reitter, 1914 in Pimeliinae: Erodiini.

*Raynalius* Chatanay, 1912a: 297 [M]. Type species: *Raynaliushispidus* Chatanay, 1912, by monotypy. Status: valid genus in Blaptinae: Opatrini: Ammobiina.

*Rehumius* Fairmaire, 1893b: 32 [M]. Type species: *Rehumiusamplithorax* Fairmaire, 1893, by subsequent designation (Gebien 1942a: 312). Status: valid genus in Stenochiinae: Cnodalonini.

*Reichardtiella* Kaszab, 1942: 18 [F]. Type species: *Reichardtiellaarmata* Kaszab, 1942, by original designation. Status: senior synonym of *Reichardtiellina* Kaszab, 1982 in Blaptinae: Opatrini: Opatrina. Note: junior homonym of *Reichardtiella* Filipjev, 1928 [Lepidoptera].

*Reichardtiellina* Kaszab, 1982c: 79 [F]. Type species [automatic]: *Reichardtiellaarmata* Kaszab, 1942, by original designation. Status: valid genus in Blaptinae: Opatrini: Opatrina. Note: replacement name for *Reichardtiella* Kaszab, 1942 (as “*Reichardtia* Kaszab, 1940”; see Iwan and Löbl 2007: 735).

*Reichenspergeria* Wasmann, 1921: 18, 19 [F]. Type species: *Reichenspergeriaaurocincta* Wasmann, 1921, by monotypy. Status: valid genus in Tenebrioninae: Amarygmini.

*Reitterella* Semenov, 1891: 362 [F]. Type species: *Reitterellafusiformis* Semenov, 1891, by monotypy. Status: valid genus in Pimeliinae: Stenosini: Dichillina.

*Reitterellus* Escalera, 1944: 91 [M]. Type species: *Dendarusdepressus* Reitter, 1915, by monotypy. Status: junior synonym of *Dendarus* Dejean, 1821 in Blaptinae: Dendarini: Dendarina. Synonymy: Español (1961a: 44).

*Reitterohelops* Skopin, 1960b: 308, 309 [M]. Type species: *Zophohelopslazarus* Reitter, 1922, by original designation. Status: valid genus in Tenebrioninae: Helopini: Cylindrinotina.

*Reminius* Casey, 1924: 321 [M]. Type species: *Reminiusocularis* Casey, 1924 (= *Tenebrioterminatus* Say, 1824), by original designation. Status: junior synonym of *Strongylium* W. Kirby, 1819 in Stenochiinae: Stenochiini. Synonymy: Spilman (1959: 63).

*Remipedella* Semenov-Tjan-Shansky, 1907b: 257 [F]. Type species: *Remipedelladeserti* Semenov-Tjan-Shansky, 1907, by monotypy. Status: valid genus in Blaptinae: Blaptini: Remipedellina.

*Renatiella* Koch, 1944b: 155 [F]. Type species: *Macropodareticulata* Gerstaecker, 1854, by monotypy. Status: valid genus in Pimeliinae: Adesmiini. Note: the First Reviser (*Renatiella* Koch, 1944 versus *Spongesmima* Koch, 1944) is Penrith (1979: 27).

*Renefouqueosis* Aalbu, Smith, Kanda & Bouchard, 2017: 314 [F]. Type species: *Renefouqueosisperuviensis* Aalbu, Smith, Kanda & Bouchard, 2017, by original designation. Status: valid genus in Pimeliinae: Stenosini: Stenosina.

*Rhacius* Champion, 1885: 120 [M]. Type species: *Rhaciussulcatulus* Champion, 1885, by subsequent designation (Gebien 1941: 805). Status: junior synonym of *Adelonia* Laporte, 1840 in Lagriinae: Belopini. Synonymy: Spilman (1961: 50).

*Rhacolaena* Kaszab, 1979b: 299 [F]. Type species: *Rhacolaenatarsalis* Kaszab, 1979, by original designation. Status: valid genus in Lagriinae: Laenini.

*Rhaebosceles* Rye, 1878: 69 [F]. Type species [automatic]: *Helopscorvinus* Küster, 1850, by subsequent designation (Gebien 1943: 415). Status: junior synonym of *Raiboscelis* Allard, 1876 in Tenebrioninae: Helopini: Helopina. Note: unjustified emendation of *Raiboscelis* Allard, 1876, not in prevailing usage.

*Rhagostira* Borchmann, 1936: 238, 468 [F]. Type species: *Rhagostiracollarti* Borchmann, 1936, by original designation. Status: valid genus in Lagriinae: Lagriini: Statirina.

*Rhaibodera* Borchmann, 1921: 217, 219 [F]. Type species: *Rhaiboderapachycera* Borchmann, 1921 (= *Statiraeurydera* Champion, 1917), by original designation. Status: valid genus in Lagriinae: Lagriini: Statirina.

*Rhaibogria* Borchmann, 1936: 17, 144 [F]. Type species: *Lagriaampla* Fairmaire, 1887, by original designation. Status: valid genus in Lagriinae: Lagriini: Lagriina.

*Rhammatodes* Haag-Rutenberg, 1876: 83 [M]. Type species: *Rhammatodeslongicornis* Haag-Rutenberg, 1876, by monotypy. Status: valid genus and subgenus in Pimeliinae: Tentyriini. Note: the First Reviser (*Rhammatodes* Haag-Rutenberg, 1876 versus *Euleantus* Haag-Rutenberg, 1876) is Koch (1952a: 133).

*Rhicnodus* Fairmaire, 1892f: 87 [M]. Type species: *Rhicnodusasper* Fairmaire, 1892, by subsequent designation (R. Lucas 1920: 568). Status: junior synonym of *Chaetyllus* Pascoe, 1860 in Lagriinae: Laenini. Synonymy: Ardoin (1969b: 126), Kaszab (1983a: 132).

*Rhinandrus* J.L. LeConte, 1866b: 119 [M]. Type species: *Rhinandrusgracilis* J.L. LeConte, 1866, by monotypy. Status: valid genus in Tenebrioninae: Tenebrionini.

*Rhinobarus* Reitter, 1906b: 131 [M]. Type species: *Cistelasulphuripes* Germar, 1823, by subsequent designation (Iablokoff-Khnzorian 1983: 133). Status: valid subgenus of *Cteniopus* Solier, 1835 in Alleculinae: Cteniopodini.

†*Rhinohelaeites* Haupt, 1950: 115, 140 [M]. Type species: *Rhinohelaeiteslongipes* Haupt, 1950, by original designation. Status: valid genus in Tenebrionidae: incertae sedis. Note: described from Middle Eocene deposits (Germany); this genus was previously considered to be “definitely not a tenebrionid” by Watt (1975: 389) but is included here following Nabozhenko (2019: 8).

*Rhipidandrus* J.L. LeConte, 1862: 236 [M]. Type species: *Xylotinusflabellicornis* Sturm, 1826 (= *Melolonthaparadoxa* Palisot de Beauvois, 1818), by monotypy. Status: valid genus in Tenebrioninae: Bolitophagini.

*Rhipidonyx* Reitter, 1876b: 304 [M]. Type species: *Rhipidonyxadustus* Reitter, 1876, by monotypy. Status: valid genus in Alleculinae: incertae sedis. Note: taxon originally described in Mycetophagidae, transferred to Tenebrionidae by Lawrence et al. (2014: 209).

*Rhizalemus* Reitter, 1904: 79 [M]. Type species: *Dendarusreitteri* Seidlitz, 1893, by subsequent designation (Chatzimanolis and Löbl 2003: 260). Status: valid subgenus of *Dendarus* Dejean, 1821 in Blaptinae: Dendarini: Dendarina.

*Rhizalus* Gebien, 1938a: 303 [M]. Type species [automatic]: *Opatrumpiceum* G.-A. Olivier, 1812, by monotypy. Status: junior synonym of *Rizalus* Mulsant & Rey, 1854 in Blaptinae: Dendarini: Dendarina. Note: unjustified emendation of *Rizalus* Mulsant & Rey, 1854, not in prevailing usage.

*Rhizoblaps* Motschulsky, 1860c: 532 [F]. Type species: *Blapspruinosa* Faldermann, 1833, by subsequent designation (Nabozhenko 2008: 36). Status: junior synonym of *Blaps* Fabricius, 1775 in Blaptinae: Blaptini: Blaptina. Synonymy: Gemminger in Gemminger and Harold (1870: 1860)

*Rhomaleus* Chatanay, 1915b: 64 [M]. Type species: *Rhomaleusscauroides* Chatanay, 1915, by monotypy. Status: valid genus in Pimeliinae: Tentyriini.

*Rhopalobates* Fairmaire, 1897c: 230 [M]. Type species: *Rhopalobatesvillardi* Fairmaire, 1897, by monotypy. Status: valid genus in Stenochiinae: Cnodalonini.

*Rhophobas* Motschulsky, 1872: 36 [M]. Type species: *Rhophobasasperatus* Motschulsky, 1872, by original designation. Status: valid genus in Stenochiinae: Cnodalonini. Note: we act as First Revisers and reject the alternative original spelling *Rophobas*, used by Motschulsky (1872: 26).

*Rhosaces* Champion, 1889: 73 [M]. Type species: *Rhosacesclavipes* Champion, 1889, by monotypy. Status: valid genus in Lagriinae: Lagriini: Statirina.

*Rhostax* Fischer von Waldheim, 1844: 67 [M]. Type species: *Rhostaxmenetriesii* Fischer von Waldheim, 1844 (= *Microderagracilis* Eschscholtz, 1831), by subsequent designation (G.S. Medvedev 1990: 109). Status: junior synonym of *Microdera* Eschscholtz, 1831 in Pimeliinae: Tentyriini. Synonymy: Kraatz (1865: 184).

*Rhydimorpha* Koch, 1943b: 768, 863 [F]. Type species: *Tentyriasubfossulata* Solier, 1835, by monotypy. Status: valid subgenus of *Rhytinota* Eschscholtz, 1831 in Pimeliinae: Tentyriini. Note: we act as First Revisers and reject the alternative original spelling *Rhytimorpha*, used by Koch (1943b: 888), since *Rhytimorpha* Szépligeti, 1901 is available in Hymenoptera.

*Rhypasma* Pascoe, 1862: 325 [N]. Type species: *Rhypasmapusillum* Pascoe, 1862, by monotypy. Status: valid genus in Lagriinae: Belopini.

*Rhysodina* Wasmann, 1921: 16 [F]. Type species [automatic]: *Rhyzodinamniszechii* Chevrolat, 1873, by monotypy. Status: junior synonym of *Rhyzodina* Chevrolat, 1873 in Tenebrioninae: Rhysopaussini. Note: unjustified emendation of *Rhyzodina* Chevrolat, 1873, not in prevailing usage.

*Rhysopaussus* Wasmann, 1896: 616 [M]. Type species: *Rhysopaussusdohertyi* Wasmann, 1896, by monotypy. Status: valid genus in Tenebrioninae: Rhysopaussini.

*Rhytidonota* Agassiz, 1846b: 327 [F]. Type species [automatic]: *Rhytinotascabriuscula* Eschscholtz, 1831, by subsequent designation (Chevrolat 1848: 279, as “*Rytinota*”). Status: junior synonym of *Rhytinota* Eschscholtz, 1831 in Pimeliinae: Tentyriini. Note: unjustified emendation of *Rhytinota* Eschscholtz, 1831 (as “*Rytinota*”), not in prevailing usage.

*Rhytinopsis* Bouchard & Bousquet, **new subgenus** [F]. Type species: *Rhytinotafossulata* Kraatz, 1880, by **present designation**. Status: valid subgenus of *Thalpophilodes* Strand, 1942, in Pimeliinae: Tentyriini. Note: Koch (1943b: 774, 870) introduced the new subgenus name *Rhytinopsis* for several nominal species, but unfortunately did not designate a type species; the subgenusRhytinopsis, which has been treated as valid since 1943, is therefore unavailable (ICZN 1999, Article 13.3); we hereby make the name available by selecting *Rhytinotafossulata* Kraatz, 1880 as type species and referring to Koch (1943b: 774, 870) for the character states that characterise and differentiate *Rhytinopsis*.

*Rhytinota* Eschscholtz, 1831: 5, 7 [F]. Type species: *Rhytinotascabriuscula* Eschscholtz, 1831, by subsequent designation (Chevrolat 1848: 279). Status: valid genus and subgenus in Pimeliinae: Tentyriini. Note: this name is an unjustified emendation of the original spelling *Rytinota*, introduced by Chevrolat (1848: 279), in prevailing usage and treated as a justified emendation (ICZN 1999, Article 33.2.3.1).

*Rhytistena* Bouchard & Bousquet, **new subgenus** [F]. Type species: *Rhytistenagridelli* Koch, 1943, by **present designation**. Status: valid subgenus of *Rhytinota* Eschscholtz, 1831 in Pimeliinae: Tentyriini. Note: Koch (1943b: 769) introduced the new subgenus name *Rhytistena* (also spelled *Rhytisten* on page 888 of the same work) for several nominal species, but unfortunately did not designate a type species; the subgenusRhytistena, which has been treated as valid since 1943, is therefore unavailable (ICZN 1999, Article 13.3); we hereby make the name available by selecting *Rhytistenagridelli* Koch, 1943 as type species and referring to Koch (1943b: 769) for the character states that characterise and differentiate *Rhytistena*.

*Rhyzodina* Chevrolat, 1873: 208 [F]. Type species: *Rhyzodinamniszechii* Chevrolat, 1873, by monotypy. Status: valid genus and subgenus in Tenebrioninae: Rhysopaussini.

*Ripicolodes* Koch, 1952d: 225, 230 [M]. Type species: *Pterostichulamisanthropa* Koch, 1952, by original designation. Status: valid subgenus of *Pterostichula* Koch, 1952 in Pimeliinae: Sepidiini: Oxurina.

*Rizalus* Mulsant & Rey, 1854: 104 [M]. Type species: *Opatrumpiceum* G.-A. Olivier, 1812, by monotypy. Status: valid subgenus of *Dendarus* Dejean, 1821 in Blaptinae: Dendarini: Dendarina.

*Robustocamaria* Pic, 1922b: 25 [F]. Type species: *Camariafortipes* Pic, 1922, by original designation. Status: valid genus in Stenochiinae: Cnodalonini.

*Robustosora* Pic, 1954: 232 [F]. Type species: *Robustosorafreynei* Pic, 1954, by original designation. Status: valid genus in Lagriinae: Lagriini: Statirina.

*Rondoniella* Kaszab, 1970c: 112 [F]. Type species: *Rondoniellacostata* Kaszab, 1970, by original designation. Status: valid genus in Pimeliinae: Cnemeplatiini: Rondoniellina.

*Rouyerus* Pic, 1911b: 3 [M]. Type species: *Rouyerusbimaculatus* Pic, 1911, by monotypy. Status: valid genus and subgenus in Lagriinae: Lagriini: Statirina.

*Rozonia* Fairmaire, 1888a: 184 [F]. Type species: *Rozoniastrigicollis* Fairmaire, 1888, by monotypy. Status: valid genus and subgenus in Pimeliinae: Tentyriini.

*Ruandania* Pic, 1955: 182 [F]. Type species: *Ruandaniarufescens* Pic, 1955, by original designation. Status: valid genus in Lagriinae: Lagriini: Lagriina.

*Rues* Casey, 1891: 66 [M]. Type species: *Helopsovipennis* Casey, 1890, by monotypy. Status: junior synonym of *Adelium* W. Kirby, 1819 in Lagriinae: Adeliini. Synonymy: Spilman (1959: 63). Note: as pointed out by Spilman (1959: 63) the type species, which was erroneously described from the United States of America, is in fact Australiana.

*Rugasida* Escalera, 1922b: 65 [F]. Type species: *Asidareticulata* Solier, 1836, by subsequent designation (F. Soldati 2008: 33). Status: junior synonym of *Asida* Latreille, 1802 in Pimeliinae: Asidini. Synonymy: Viñolas and Cartagena (2005: 291).

*Rugoplatynotus* Kaszab, 1975b: 281, 288 [M]. Type species: *Pseudoblapsandrewesii* Fairmaire, 1896, by original designation. Status: valid genus in Blaptinae: Platynotini: Platynotina.

*Rugosiheliofugus* Freude, 1960a: 125, 128 [M]. Type species: *Heliophygussulcatulus* Gemminger, 1870, by original designation. Status: valid subgenus of *Heliofugus* Guérin-Méneville, 1831 in Stenochiinae: Cnodalonini.

*Rhyssochiton* Gray in Griffith and Pidgeon, 1831: pl. 50 [M]. Type species: *Rhyssochitonpolitus* Gray, 1831, by monotypy. Status: junior synonym of *Blapida* Perty, 1830 in Stenochiinae: Cnodalonini. Synonymy: Lacordaire (1859b: 425). Note: two spellings originally appeared in plates of the same work, *Ryssocheton* (Gray in Griffith and Pidgeon, 1831: pl. 50) and *Rhyssochiton* (Gray in Griffith and Pidgeon, 1831: pl. 69); the third spelling *Ryssochiton* was also used in the “Index for plates” of Volume 15 of the same work (Gray in Griffith and Pidgeon, 1832: 793); we act as First Revisers and select the alternative original spelling *Rhyssochiton*.

*Sabularius* Escalera, 1914: 353 [M]. Type species: *Helopsfossor* Escalera, 1914, by monotypy. Status: valid genus in Tenebrioninae: Helopini: Helopina.

*Sabulophosis* Penrith, 1977: 19, 227 [F]. Type species: *Zophosisgaerdesi* Koch, 1958, by original designation. Status: valid subgenus of *Zophosis* Latreille, 1802 in Pimeliinae: Zophosini.

*Saccophorella* Strand, 1935b: 303 [F]. Type species [automatic]: *Saccophoruscrenulatus* Haag-Rutenberg, 1872, by monotypy. Status: junior synonym of *Horatoma* Solier, 1841 in Pimeliinae: Cryptochilini: Cryptochilina. Note: replacement name for *Saccophorus* Haag-Rutenberg, 1872.

*Saccophorus* Haag-Rutenberg, 1872: 274, 303 [M]. Type species: *Saccophoruscrenulatus* Haag-Rutenberg, 1872, by monotypy. Status: junior synonym of *Horatoma* Solier, 1841 in Pimeliinae: Cryptochilini: Cryptochilina. Synonymy: Penrith and Endrödy-Younga (1994: 10). Note: junior homonym of *Saccophorus* Kuhl, 1820 [Mammalia].

*Sadanaria* Ando & Ichiyanagi, 2009: 80 [F]. Type species: *Sadanariasakaii* Ando & Ichiyanagi, 2009, by original designation. Status: valid genus in Stenochiinae: Cnodalonini.

*Saeculum* Kamiński, Kanda & Smith, 2021: 196 [N]. Type species: *Saeculumzoologicum* Kamiński, Kanda & Smith, 2021, by original designation. Status: valid genus in Pimeliinae: Asidini.

*Saerangodes* Sturm, 1843: 163 [M]. Type species: *Helopsinterpunctatus* Germar, 1823, by monotypy. Status: junior synonym of *Strongylium* W. Kirby, 1819 in Stenochiinae: Stenochiini. Synonymy: Blanchard (1845: 33, with *Stenochia* W. Kirby, 1819, a synonym of *Styrongylium* W. Kirby, 1819).

*Saharoplarion* Koch, 1948: 431 [N]. Type species: *Microsituscompactus* Fairmaire, 1880, by original designation. Status: valid subgenus of *Hoplarion* Mulsant & Rey, 1854 in Blaptinae: Dendarini: Melambiina.

*Saitostrongylium* Masumoto, 1996a: 34 [N]. Type species: *Saitostrongyliumacco* Masumoto, 1996, by original designation. Status: valid genus in Stenochiinae: Stenochiini.

*Sakaiomenimus* Ando, 2003b: 135 [M]. Type species: *Sakaiomenimustodai* Ando, 2003, by original designation. Status: valid genus in Diaperinae: Gnathidiini: Gnathidiina.

*Salax* Guérin-Méneville, 1834: 11 [M]. Type species: *Salaxlacordairii* Guérin-Méneville, 1834, by monotypy. Status: valid genus in Pimeliinae: Trilobocarini.

*Saptine* Champion, 1886: 180 [F]. Type species: *Saptineovata* Champion, 1886, by monotypy. Status: valid genus in Diaperinae: Diaperini: Diaperina.

*Sarachus* Gistel, 1848a: viii [M]. Type species [automatic]: *Adesmialongipes* Fischer, 1822 (= *Pimeliaanomala* Fischer, 1820), by monotypy. Status: junior synonym of *Adesmia* Fischer, 1822 in Pimeliinae: Adesmiini. Note: unnecessary replacement name for *Adesmia* Fischer, 1822.

*Saragella* Carter, 1937: 136 [F]. Type species: *Saragellapalpalis* Carter, 1937, by monotypy. Status: junior synonym of *Dysarchus* Pascoe, 1866 in Tenebrioninae: Heleini: Heleina. Synonymy: Matthews (1993: 1059).

*Saragodinus* Bates, 1872b: 269 [M]. Type species: *Saragodinusduboulayi* Bates, 1872, by subsequent designation (R. Lucas 1920: 579). Status: junior synonym of *Dysarchus* Pascoe, 1866 in Tenebrioninae: Heleini: Heleina. Synonymy: Carter (1921: 307).

*Saragus* Erichson, 1842a: 171 [M]. Type species: **fixed herein** (ICZN 1999, Article 70.3) as *Celibecostata* Solier, 1848, misidentified as *Silphalaevicollis* Fabricius, 1775 in the original designation by monotypy in Erichson (1842a). Status: valid genus in Tenebrioninae: Heleini: Heleina. Note: the type species “*Silphalaevicollis* Fabricius” was first established by monotypy; Schaum (1850: 183) first noted that *Silphalaevicollis* Fabricius of Erichson (1842a) was misidentified and corresponded to *Celibecostata* Solier, 1848 (as “*Cilibecostatus*”); we follow currently accepted concepts (e.g., Matthews and Bouchard 2008: 310) and fix the type species according to the requirements of Article 70.3.2 (ICZN 1999); the nominal species *Silphalaevicollis* Fabricius, 1775 is a valid species in the genus *Boreosaragus* Matthews, 1993 [Coleoptera: Tenebrionidae].

*Sarandonyx* Gozis, 1881: 151 [M]. Type species [automatic]: *Chrysomelasulphurea* Linnaeus, 1758, by subsequent designation (Westwood 1838: 32). Status: junior synonym of *Cteniopus* Solier, 1835 in Alleculinae: Cteniopodini. Note: unnecessary replacement name for *Cteniopus* Solier, 1835.

*Sarothropus* Kraatz, 1865: 268 [M]. Type species: *Akisdepressa* Zoubkoff, 1837, by monotypy. Status: valid genus in Pimeliinae: Akidini.

*Satanocalcar* Pic, 1925b: 9 [N]. Type species: *Satanocalcarcornutum* Pic, 1925, by monotypy. Status: valid genus in Tenebrioninae: Tenebrionini.

*Saxistena* Löbl & Merkl in Löbl et al., 2020: 3 [F]. Type species: *Mesostenalongicornis* Kraatz, 1865, by original designation. Status: valid subgenus of *Mesostena* Eschscholtz, 1831 in Pimeliinae: Tentyriini. Note: name first proposed by Koch (1940c: 65) without fixation of a type species in the original publication (ICZN 1999, Article 13.3); Löbl and Merkl (2003: 252) designated *Mesostenalongicornis* Kraatz, 1865 as the type species of Koch’s name but did not explicitly indicate the genus-group name as intentionally new (ICZN 1999, Article 16.1).

*Saziches* Champion, 1886: 261 [M]. Type species: *Sazichessubcaudatus* Champion, 1886, by monotypy. Status: valid genus in Stenochiinae: Cnodalonini.

*Scaletomerus* Blackburn, 1891: 330 [M]. Type species: *Scaletomerusharpaloides* Blackburn, 1891 (= *Cistelapolitus* W.J. MacLeay, 1872), by subsequent designation (R. Lucas 1920: 580). Status: valid genus in Alleculinae: Alleculini: Alleculina.

*Scaphidema* Redtenbacher, 1848: 591 [F]. Type species: *Scaphidiumbicolor* Fabricius, 1798, by monotypy. Status: valid genus in Diaperinae: Scaphidemini. Note: see Kerzhner (2003: 322) and Löbl and Smetana (2010: 34) for comments on the gender of this name.

*Scaphinion* Matthews, 2012: 2 [M]. Type species: *Scaphinionclavatus* Matthews, 2012, by original designation. Status: valid genus in Alleculinae: Alleculini: Alleculina.

*Scaptes* Champion, 1886: 222 [M]. Type species: *Scaptessquamulatus* Champion, 1886 (= *Asidatropica* Kirsch, 1866), by subsequent designation (Bousquet et al. 2018: 194). Status: junior synonym of *Ammodonus* Mulsant, 1859 in Blaptinae: Opatrini: Ammobiina. Synonymy: Fall (1912: 48).

*Scauris* Rafinesque, 1815: 113 [F]. Type species [automatic]: *Scaurusatratus* Fabricius, 1775, by monotypy. Status: junior synonym of *Scaurus* Fabricius, 1775 in Tenebrioninae: Scaurini. Note: unjustified emendation of *Scaurus* Fabricius, 1775, not in prevailing usage.

*Scaurus* Fabricius, 1775: 253 [M]. Type species: *Scaurusatratus* Fabricius, 1775, by monotypy. Status: valid genus in Tenebrioninae: Scaurini.

*Scelace* Marseul, 1887: 325 [F]. Type species: *Pimeliatuberculifera* P.H. Lucas, 1858, by subsequent designation (Bouchard and Bousquet 2020b: 7). Status: valid genus in Pimeliinae: Pimeliini. Note: *Scelace* was published by Marseul on June 9, 1887 while its junior synonym *Pachyscelodes* was published by Sénac (1887: 189) on August 15 of the same year.

*Sceleodis* Gemminger in Gemminger and Harold, 1870: 1836 [M]. Type species [automatic]: *Cratopuscastaneus* Eschscholtz, 1831, by monotypy. Status: junior synonym of *Scelosodis* Solier, 1835 in Pimeliinae: Tentyriini. Note: unjustified emendation of *Scelosodis* Solier, 1835, not in prevailing usage.

*Sceleoides* Agassiz, 1846b: 333 [M]. Type species [automatic]: *Cratopuscastaneus* Eschscholtz, 1831, by monotypy. Status: junior synonym of *Scelosodis* Solier, 1835 in Pimeliinae: Tentyriini. Note: unjustified emendation of *Scelosodis* Solier, 1835, not in prevailing usage.

*Scelidospecta* Kulzer, 1954b: 204 [F]. Type species: *Entomodereslobatus* Burmeister, 1875, by original designation. Status: valid genus in Pimeliinae: Nycteliini. Note: *Scelidospecta* is an incorrect subsequent spelling of the original name *Scelidopsecta* in prevailing usage and attributed to the publication of the original spelling; *Scelidospecta* is treated as the correct original spelling (ICZN 1999, Article 33.3.1).

*Scelocolpis* Reitter, 1900c: 105 [M]. Type species: *Colposcelisdamone* Reitter, 1900, by subsequent designation (G.S. Medvedev 1990: 64). Status: valid subgenus of *Colposcelis* Dejean, 1834 in Pimeliinae: Tentyriini.

*Scelosodis* Solier, 1835b: 253, 283 [M]. Type species [automatic]: *Cratopuscastaneus* Eschscholtz, 1831, by monotypy. Status: valid genus in Pimeliinae: Tentyriini. Note: replacement name for *Cratopus* Eschscholtz, 1831.

*Schedarosus* Reitter, 1876a: 42 [M]. Type species: *Schedarosuscucujiformis* Reitter, 1876 (= *Pythopallidus* Say, 1823), by subsequent designation (Löbl et al. 2008a: 42). Status: junior synonym of *Adelina* Dejean, 1835 in Diaperinae: Diaperini: Adelinina. Synonymy: Champion (1886: 157, with *Doliema* Pascoe, 1860, a junior synonym of *Adelina* Dejean, 1835).

*Schelodontes* Koch, 1956a: 81 [M]. Type species: *Trigonopusimmundus* Mulsant & Rey, 1853, by original designation. Status: valid genus in Blaptinae: Platynotini: Platynotina.

*Schevodera* Borchmann, 1936: 16, 59 [F]. Type species: *Lagriahirticollis* Borchmann, 1911, by original designation. Status: valid genus in Lagriinae: Lagriini: Lagriina.

*Schevogria* Borchmann, 1936: 20, 164 [F]. Type species: *Schevogriamethneri* Borchmann, 1936, by original designation. Status: valid genus in Lagriinae: Lagriini: Lagriina.

*Schizaraeus* Kulzer, 1955b: 479 [M]. Type species: *Schizaraeusacuticosta* Kulzer, 1955, by original designation. Status: valid genus in Pimeliinae: Stenosini: Stenosina.

*Schizillus* Horn, 1874: 33 [M]. Type species: *Schizilluslaticeps* Horn, 1874, by monotypy. Status: valid genus in Pimeliinae: Cryptoglossini.

*Schizillus* Wasmann, 1899a: 166 [M]. Type species: *Schizillusrogersi* Wasmann, 1899, by monotypy. Status: junior synonym of *Pseudethas* Fairmaire, 1896 in Pimeliinae: Stenosini: Dichillina. Synonymy: Gebien (1937a: 679). Note: junior homonym of *Schizillus* Horn, 1874 [Coleoptera: Tenebrionidae: Pimeliinae: Cryptoglossini].

*Schizomma* Gebien, 1921a: 325, 392 [N]. Type species: *Schizommacucumericola* Gebien, 1921, by original designation. Status: senior synonym of *Diachoriops* Ando, 2020 in Stenochiinae: Cnodalonini. Note: junior homonym of *Schizomma* Ehrenberg, 1861 [Protista].

*Schizophthalmotribolium* Kaszab, 1940c: 173 [N]. Type species: *Schizophthalmotriboliumaustraliae* Kaszab, 1940, by original designation. Status: junior synonym of *Paratoxicum* Champion, 1894 in Tenebrioninae: Tenebrionini. Synonymy: Matthews (2004: 261).

*Schlinkus* R. Lucas, 1920: 584 [M]. Type species [automatic]: *Cyphonotusdromedarius* Guérin-Méneville, 1831, by monotypy. Status: junior synonym of *Homocyrtus* Dejean, 1834 in Tenebrionidae: incertae sedis. Synonymy: Gebien (1948: 514). Note: replacement name for *Cyphonotus* Guérin-Méneville, 1831.

*Schoenicus* J.L. LeConte, 1866b: 109 [M]. Type species: *Schoenicuspuberulus* J.L. LeConte, 1866, by monotypy. Status: valid genus in Pimeliinae: Epitragini.

*Schoeniphegoneus* Freude, 1968: 99 [M]. Type species: *Epitragussemicastaneus* Curtis, 1845, by original designation. Status: valid subgenus of *Phegoneus* Casey, 1907 in Pimeliinae: Epitragini.

*Schusteriella* Koch, 1940b: 745, 746 [F]. Type species: *Stenosisruficornis* Reitter, 1886, by monotypy. Status: valid genus in Pimeliinae: Stenosini: Stenosina.

*Schweinfurthia* Andres in Kneucker, 1922: 26 [F]. Type species: *Schweinfurthiasinaitica* Andres, 1922, by monotypy. Status: valid genus in Pimeliinae: Tentyriini.

*Schyzoschelus* Koch, 1954a: 72 [M]. Type species: *Schyzoscheluskaszabi* Koch, 1954, by original designation. Status: valid genus in Blaptinae: Platynotini: Eurynotina.

*Sciaca* Solier, 1835b: 408 [F]. Type species: *Hylithusdisctinctus* Solier, 1835, by **present designation**. Status: junior synonym of *Hylithus* Guérin-Méneville, 1834 in Pimeliinae: Edrotini. Synonymy: Chevrolat (1848: 423). Note: the name *Sciaca* was originally listed as synonym of *Hylithus* Guérin-Méneville, 1834; it was treated before 1961 as an available name and adopted as the name of a taxon (e.g., Dejean 1836: 204), *Sciaca* was therefore made available from its first publication as a synonym (ICZN 1999, Article 11.6.1).

*Sciophagus* Sharp in Blackburn and Sharp, 1885: 167 [M]. Type species: *Ulomapandanicola* Boisduval, 1835, by monotypy. Status: valid genus in Diaperinae: Diaperini: Diaperina.

*Scleroides* Fairmaire, 1883a: 32 [M]. Type species: *Scleroidespluricostatus* Fairmaire, 1883, by monotypy. Status: valid genus in Blaptinae: Opatrini: incertae sedis. Note: placed in Opatrini incertae sedis by Kamiński et al. (2021b: 151).

*Scleron* Hope, 1841: 111 [N]. Type species: *Opatrumorientale* Fabricius, 1775, by original designation. Status: junior synonym of *Sclerum* Dejean, 1834 in Blaptinae: Opatrini: Sclerina. Synonymy: Erichson (1842b: 237); Bouchard et al. (2005: 511).

*Scleronimon* Reitter, 1904: 127 [M]. Type species: *Eurycaulusgranulatus* Reitter, 1904, by subsequent designation (Löbl and Merkl 2003: 249). Status: junior synonym of *Eurycaulus* Fairmaire, 1868 in Blaptinae: Opatrini: Sclerina. Synonymy: Grimm (2005: 9).

*Scleronopsis* Koch, 1935: 86, 87 [F]. Type species: *Scleronhirsutus* Miller, 1861, by monotypy. Status: junior synonym of *Eurycaulus* Fairmaire, 1868 in Blaptinae: Opatrini: Sclerina. Synonymy: Grimm (2005: 9).

*Scleropatroides* Löbl & Merkl, 2003: 250 [M]. Type species: *Opatrumhirtulum* Baudi di Selve, 1876, by original designation. Status: valid genus in Blaptinae: Opatrini: Opatrina.

*Scleropatrum* Reitter, 1887a: 388 [N]. Type species: *Scleropatrumtuberculatum* Reitter, 1887, by monotypy. Status: valid genus in Blaptinae: Opatrini: Opatrina.

*Sclerum* Dejean, 1834: 193 [N]. Type species: *Opatrumorientale* Fabricius, 1775, by subsequent designation (Hope 1841: 110). Status: valid genus in Blaptinae: Opatrini: Sclerina.

*Scolytocaulus* Fairmaire, 1896c: 98 [M]. Type species: *Scolytocaulusbouchardi* Fairmaire, 1896, by monotypy. Status: valid genus in Phrenapatinae: Penetini.

*Scopulophosis* Penrith, 1977: 19, 215 [F]. Type species: *Zophosiscacozela* Koch, 1958, by original designation. Status: valid subgenus of *Zophosis* Latreille, 1802 in Pimeliinae: Zophosini.

*Scotaeus* Hope, 1834: 14 [M]. Type species: *Scotaeuscorallipes* Hope, 1834, by monotypy. Status: valid genus in Stenochiinae: Cnodalonini.

*Scotera* Motschulsky, 1845b: 365 [F]. Type species: *Scoteragibbosa* Motschulsky, 1845, by monotypy. Status: junior synonym of *Cibdelis* Mannerheim, 1843 in Stenochiinae: Cnodalonini. Synonymy: Motschulsky (1845b: 365). Note: as pointed out by Bousquet et al. (2018: 296) the generic name *Scotera* and the specific name *gibbosa*, proposed as synonyms of *Cibdelisblaschkii* by Motschulsky (1845b: 365, 366), are available from Motschulsky’s work (ICZN 1999, Article 11.6.1) because they were treated as valid names before 1961 (e.g., Chevrolat 1848: 454).

*Scotinesthes* Fairmaire, 1895a: 19 [F]. Type species: *Scotinesthesacuticosta* Fairmaire, 1895, by subsequent designation (Gebien 1937a: 741). Status: valid genus in Pimeliinae: Asidini.

*Scotinus* W. Kirby, 1819a: 415 [M]. Type species: *Scotinuscrenicollis* W. Kirby, 1819, by monotypy. Status: valid genus in Pimeliinae: Asidini.

*Scotobaenus* J.L. LeConte, 1859: 87 [M]. Type species: *Scotobaenusparallelus* J.L. LeConte, 1859, by monotypy. Status: valid genus in Tenebrioninae: Centronopini.

*Scotobates* Rye, 1877: 341 [M]. Type species: *Helopscalcaratus* Fabricius, 1798, by subsequent designation (R. Lucas 1920: 587). Status: junior synonym of *Menechides* Motschulsky, 1872 in Tenebrioninae: Centronopini. Synonymy: R. Lucas (1920: 587). Note: see Spilman (1962b: 2–3) regarding availability of this genus name; *Scotobates* was used earlier by Horn (1876a: 151) without a description, a definition, or an indication (ICZN 1999, Article 12.1) and is therefore unavailable from that date.

*Scotobiopsis* Brèthes, 1910: 207 [F]. Type species: *Scotobiopsisbreyeri* Brèthes, 1910, by monotypy. Status: valid genus in Alleculinae: Alleculini: Xystropodina.

*Scotobius* Germar, 1823: 135 [M]. Type species: *Scotobiuspilularius* Germar, 1823, by subsequent designation (Lacordaire 1830a: 282). Status: valid genus in Tenebrioninae: Scotobiini.

*Scotochares* Boheman, 1858: 95 [M]. Type species: *Scotocharesinsularis* Boheman, 1858, by monotypy. Status: valid genus in Tenebrioninae: Ulomini.

*Scotoderus* Perroud & Montrouzier, 1865: 114 [M]. Type species: *Scotoderuscancellatus* Perroud & Montrouzier, 1865 (= *Tenebriocancellatus* Montrouzier, 1860), by monotypy. Status: valid genus in Stenochiinae: Cnodalonini.

*Scutopiloxys* Pic, 1924b: 13 [M]. Type species: *Scutopiloxysperroti* Pic, 1924, by monotypy. Status: valid genus in Stenochiinae: Cnodalonini.

*Scymena* Pascoe, 1866a: 455 [F]. Type species: *Scymenavariabilis* Pascoe, 1866, by original designation. Status: valid genus in Blaptinae: Opatrini: Heterotarsina.

*Scythis* Schaum in Kraatz, 1865: 80, 102 [F]. Type species: *Tentyriamacrocephala* Tauscher, 1812, by subsequent designation (Gebien 1937a: 622). Status: valid genus in Pimeliinae: Tentyriini.

*Scythodonta* Reitter, 1897a: 300 [F]. Type species: *Scythishumeridens* Reitter, 1887, by monotypy. Status: junior synonym of *Scytosoma* Reitter, 1895 in Pimeliinae: Tentyriini. Synonymy: Skopin (1979: 171).

*Scytosoma* Reitter, 1895: 281 [N]. Type species: *Scytosomaarcibasis* Reitter, 1895 (= *Tentyriapygmaea* Gebler, 1832), by subsequent designation (Gebien 1937a: 622). Status: valid genus in Pimeliinae: Tentyriini. Note: redescribed as new by Reitter (1897a: 299).

*Sebastianus* Iwan, 1996: 385, 416 [M]. Type species: *Melanocratusmajor* Fairmaire, 1899, by original designation. Status: valid genus in Blaptinae: Platynotini: Platynotina.

*Sechuranus* Flores & Giraldo-Mendoza in Giraldo-Mendoza and Flores, 2019: 93 [M]. Type species: *Prohylithusbarbatus* Kaszab, 1964, by original designation. Status: valid genus in Pimeliinae: Edrotini.

*Seidlitzellus* Reitter, 1920b: 221 [M]. Type species [automatic]: *Anisocerustristis* Faldermann, 1837, by monotypy. Status: junior synonym of *Ceratanisus* Gemminger, 1870 in Pimeliinae: Ceratanisini. Note: unnecessary replacement name for *Anisocerus* Faldermann, 1837, a senior synonym of *Ceratanisus* Gemminger, 1870.

*Seirotrana* Pascoe, 1866a: 483 [F]. Type species: *Adeliumcatenulatum* Boisduval, 1835, by original designation. Status: valid genus in Lagriinae: Adeliini.

*Selenomma* Dejean, 1836: 203 [F]. Type species [automatic]: *Ammophorusperuvianus* Guérin-Méneville, 1831, by monotypy. Status: junior synonym of *Ammophorus* Guérin-Méneville, 1831 in Tenebrioninae: Scotobiini. Note: unnecessary replacement name for *Ammophorus* Guérin-Méneville, 1831.

*Selenepistoma* Dejean, 1834: 190 [N]. Type species: *Opatrumacutum* Wiedemann, 1823, by subsequent designation (Bousquet and Bouchard 2013a: 50). Status: valid genus in Blaptinae: Dendarini: Melambiina.

*Selinopodus* Koch, 1956a: 79 [M]. Type species: *Selinopodusgiganteus* Koch, 1956, by original designation. Status: valid genus in Blaptinae: Platynotini: Platynotina.

*Selinus* Mulsant & Rey, 1853b: 70, 97 [M]. Type species: *Opatrumplanum* Fabricius, 1792, by subsequent designation (Cazurro Ruiz 1896: 963). Status: valid genus in Blaptinae: Platynotini: Platynotina.

*Sellio* Mulsant & Rey, 1859a: 70, 105 [M]. Type species: *Blaps tibidens* Quensel, 1806, by subsequent designation (Gebien 1938a: 407). Status: junior synonym of *Diastolinus* Mulsant & Rey, 1859 in Blaptinae: Opatrini: Blapstinina. Synonymy: Ivie and Hart (2016: 468).

*Semenovonymus* Bogatchev, 1946: 391 [M]. Type species: *Semenovonymustenuis* Bogatchev, 1946, by monotypy. Status: junior synonym of *Scythis* Kraatz, 1865 in Pimeliinae: Tentyriini. Synonymy: G.S. Medvedev (1990: 101).

*Semieutochia* Kaszab, 1980a: 187 [F]. Type species: *Semieutochiaooidea* Kaszab, 1980, by original designation. Status: valid genus in Tenebrioninae: Ulomini. Note: *Semieutochia* was used earlier by Kaszab (1979a: 91) without a description, a definition, or a bibliographic reference to such a published statement (ICZN 1999, Article 13.1) and is therefore unavailable from that date.

*Seorsophloeus* Bremer, 1998: 10, 12 [M]. Type species: *Corticeusbirmanicus* Blair, 1921, by original designation. Status: valid subgenus of *Corticeus* Piller & Mitterpacher, 1783 in Diaperinae: Hypophlaeini.

*Seorsoplonyx* Bremer, 2010: 144, 156 [M]. Type species: *Seorsoplonyxantennatus* Bremer, 2010, by original designation. Status: valid genus in Tenebrioninae: Amarygmini.

*Sepedonastes* Gistel, 1856: 382 [M]. Type species: *Tenebriobimaculatus* Herbst, 1799 (= *Dytiscusbimaculatus* Linnaeus, 1767), by subsequent designation (Bouchard et al. 2005: 501). Status: junior synonym of *Phaleria* Latreille, 1802 in Diaperinae: Phaleriini. Synonymy: Bouchard et al. (2005: 513).

*Sepidiacis* Fairmaire, 1884c: cxlvi [F]. Type species: *Sepidiaciscompressa* Fairmaire, 1884, by subsequent designation (W.F. Kirby 1885b: 83). Status: junior synonym of *Sepidiostenus* Fairmaire, 1884 in Pimeliinae: Sepidiini: Sepidiina. Synonymy: Gestro (1892: 775). Note: redescribed as new by Fairmaire (1887a: 185).

*Sepidiopsis* Gestro, 1892: 771 [F]. Type species: *Sepidiopsiscornigera* Gestro, 1892, by original designation. Status: valid genus in Pimeliinae: Sepidiini: Sepidiina.

*Sepidiostenus* Fairmaire, 1884b: lxxv [M]. Type species: *Sepidiostenuserinaceus* Fairmaire, 1884, by monotypy. Status: valid genus in Pimeliinae: Sepidiini: Sepidiina. Note: redescribed as new, under the spelling *Sepidostenus*, by Fairmaire (1887a: 184).

*Sepidium* Fabricius, 1775: 250 [N]. Type species: *Sepidium tricuspidatum* Fabricius, 1775, by subsequent designation (Latreille 1810: 429). Status: valid genus in Pimeliinae: Sepidiini: Sepidiina.

*Sepilokus* Iwan & Raś, 2020: 776 [M]. Type species: *Sepilokustenenbaumi* Iwan & Raś, 2020, by original designation. Status: valid genus in Phrenapatinae: Archaeoglenini.

*Septentriophosis* Penrith, 1982: 167 [F]. Type species: *Erodiusplanus* Fabricius, 1775, by original designation. Status: valid subgenus of *Zophosis* Latreille, 1802 in Pimeliinae: Zophosini.

*Seriscius* Motschulsky, 1845a: 77 [M]. Type species: *Serisciuspubescens* Motschulsky, 1845 (= *Crypticusrufipes* Gebler, 1830), by monotypy. Status: valid subgenus of *Crypticus* Latreille, 1816 in Diaperinae: Crypticini.

*Serrania* Garrido, 2003: 50 [F]. Type species: *Diaperisviridula* Zayas, 1988 (= *Platydemavirens* Laporte & Brullé, 1831), by original designation. Status: junior synonym of *Nesocyrtosoma* Marcuzzi, 1976 in Stenochiinae: Cnodalonini. Synonymy: Hopp and Ivie (2009: 13).

*Serrichora* Koch, 1952b: 15 [F]. Type species: *Eurychoracrenata* Solier, 1837, by original designation. Status: valid genus in Pimeliinae: Adelostomini.

*Serridenos* Koch, 1956a: 325 [M]. Type species: *Zadenossolenopistoma* Koch, 1956, by original designation. Status: valid subgenus of *Selenepistoma* Dejean, 1834 in Blaptinae: Dendarini: Melambiina.

*Setenis* Motschulsky, 1872: 24 [M]. Type species: *Tenebriovalgus* Wiedemann, 1823, by original designation. Status: junior synonym of *Promethis* Pascoe, 1869 in Stenochiinae: Cnodalonini. Synonymy: Kaszab (1988: 70).

*Seydelicistela* Pic, 1954: 260 [F]. Type species: *Seydelicistelarubrithorax* Pic, 1954, by monotypy. Status: valid genus in Alleculinae: incertae sedis.

*Sicharbas* Champion, 1884: 67 [M]. Type species: *Sicharbaslobatus* Champion, 1884, by monotypy. Status: valid subgenus of *Pelecyphorus* Solier, 1836 in Pimeliinae: Asidini.

*Sicinus* Champion, 1886: 146 [M]. Type species: *Sicinusguatemalensis* Champion, 1886, by subsequent designation (Löbl et al. 2008a: 43). Status: junior synonym of *Gnatocerus* Thunberg, 1814 in Diaperinae: Diaperini: Adelinina. Synonymy: Leng (1920: 233).

*Silvestriellum* Koch, 1956a: 362 [N]. Type species: *Silvestriellumalatum* Koch, 1956, by original designation. Status: valid genus in Blaptinae: Dendarini: Melambiina.

*Simalura* Gebien, 1914d: 71 [F]. Type species: *Simalurajacobsoni* Gebien, 1914, by monotypy. Status: valid genus in Stenochiinae: Cnodalonini.

*Simarus* Borchmann, 1909a: 713 [M]. Type species [automatic]: *Ismarusgodeffroyi* Haag-Rutenberg, 1878, by monotypy. Status: valid genus in Alleculinae: Alleculini: Alleculina. Note: replacement name for *Ismarus* Haag-Rutenberg, 1878.

*Similepitragus* Freude, 1967: 148, 167 [M]. Type species: *Epitragussimilis* Steinheil, 1872, by original designation. Status: valid subgenus of *Epitragus* Latreille, 1802 in Pimeliinae: Epitragini.

*Singapura* Gebien, 1925f: 325 [F]. Type species: *Singapuraquadrihamata* Gebien, 1925, by monotypy. Status: valid genus in Tenebrioninae: Amarygmini.

*Sinocatomus* Nabozhenko, 2006: 852 [M]. Type species: *Catomussolitarius* Nabozhenko, 2006, by original designation. Status: valid subgenus of *Catomus* Allard, 1876 in Tenebrioninae: Helopini: Helopina.

†*Sinocistela* Zhang, 1989: 145 [F]. Type species: *Sinocistelasiphla* Zhang, 1989, by original designation. Status: valid genus in Alleculinae: Cteniopodini. Note: described from Lower Miocene deposits (China).

*Sinoecia* Chatanay, 1914a: 220 [F]. Type species: *Sinoeciapuncticollis* Chatanay, 1914, by original designation. Status: valid genus in Pimeliinae: Tentyriini.

*Sinomenimus* G.S. Medvedev, 2007b: 675 [M]. Type species: *Menimuskabaki* G.S. Medvedev, 2007, by original designation. Status: valid subgenus of *Menimus* Sharp, 1876 in Diaperinae: Gnathidiini: Gnathidiina.

*Sinopium* Pascoe, 1866a: 487 [N]. Type species: *Strongyliumvariabile* Walker, 1858, by original designation. Status: junior synonym of *Camarimena* Motschulsky, 1863 in Stenochiinae: Cnodalonini. Synonymy: Gebien (1919: 155).

*Sinorus* Mulsant & Revelière, 1861: 153 [M]. Type species: *Sinorusciliaris* Mulsant & Revelière, 1861 (= *Opatrumcolliardi* Fairmaire, 1860), by monotypy. Status: valid genus in Blaptinae: Opatrini: Opatrina.

*Sintagona* G.S. Medvedev, 1998b: 585 [F]. Type species: *Sintagonamiranda* G.S. Medvedev, 1998, by original designation. Status: valid genus in Blaptinae: Blaptini: Gnaptorinina.

*Sipirocus* Fairmaire, 1896c: 103 [M]. Type species: *Sipirocusritsemae* Fairmaire, 1896, by monotypy. Status: junior synonym of *Aediatorix* Bates, 1868 in Lagriinae: Pycnocerini. Synonymy: Gebien (1905: 258).

*Sipolisia* Fairmaire, 1889c: xlix [F]. Type species: *Sipolisiaserricornis* Fairmaire, 1889, by monotypy. Status: valid genus in Lagriinae: Lagriini: Statirina.

*Sitophagus* Mulsant, 1854: 264 [M]. Type species: *Sitophagussolieri* Mulsant, 1854 (= *Ulomahololeptoides* Laporte, 1840), by monotypy. Status: valid genus in Diaperinae: Diaperini: Adelinina.

*Sloanea* Carter, 1916: 209 [F]. Type species: *Sloaneacostata* Carter, 1916, by monotypy. Status: valid genus in Tenebrioninae: Heleini: Asphalina.

*Smiliophanus* Koch, 1950a: 66 [M]. Type species [automatic]: *Smiliotussteiroides* Haag-Rutenberg, 1875, by monotypy. Status: valid genus in Pimeliinae: Adelostomini. Note: replacement name for *Smiliotus* Haag-Rutenberg, 1875.

*Smiliotus* Haag-Rutenberg, 1875b: 4, 52 [M]. Type species: *Smiliotussteiroides* Haag-Rutenberg, 1875, by monotypy. Status: senior synonym of *Smiliophanus* Koch, 1950 in Pimeliinae: Adelostomini. Note: junior homonym of *Smiliotus* Loew, 1857 [Diptera].

*Sobas* Pascoe, 1863a: 45 [F]. Type species [automatic]: *Trigonotarsusaustralis* Hope, 1843, by original designation. Status: valid genus in Blaptinae: Opatrini: Opatrina. Note: replacement name for *Trigonotarsus* Hope, 1843. Note: moved from the subtribe Ammobiina to Opatrina by Kamiński et al. (2021b: 151).

*Socotralia* Novák, 2007: 321 [F]. Type species: *Socotraliamajor* Novák, 2007, by original designation. Status: valid genus in Alleculinae: Alleculini: Alleculina.

*Socotraphanes* Nabozhenko in Nabozhenko and Purchart, 2019: 150 [M]. Type species: *Socotraphaneskrali* Nabozhenko, 2019, by original designation. Status: valid genus in Tenebrioninae: Helopini: Helopina.

*Socotropatrum* Koch, 1970: 109 [N]. Type species: *Opatrumcostiferum* C.O. Waterhouse, 1881, by original designation. Status: valid genus in Blaptinae: Opatrini: Opatrina.

*Soemias* Champion, 1884: 4 [F]. Type species: *Soemiasminuta* Champion, 1884, by monotypy. Status: valid genus in Pimeliinae: Edrotini.

*Solenomerus* Fåhraeus, 1870: 306 [M]. Type species: *Solenomeruslongipes* Fåhraeus, 1870, by monotypy. Status: junior synonym of *Micrantereus* Solier, 1848 in Blaptinae: Pedinini: Helopinina. Synonymy: Fairmaire (1897f: 131).

*Solskyia* Solsky, 1881: 48 [F]. Type species: *Solskyiaperegrina* Solsky, 1881, by monotypy. Status: valid genus in Pimeliinae: Akidini.

*Somaladesmia* Koch, 1944b: 147 [F]. Type species: *Adesmiaconsimilis* Gahan, 1896, by monotypy. Status: valid subgenus of *Adesmia* Fischer, 1822 in Pimeliinae: Adesmiini.

*Somalammodes* Koch, 1943a: 500, 510 [M]. Type species: *Somalammodesdelaruei* Koch, 1943, by monotypy. Status: valid genus in Pimeliinae: Erodiini.

*Somalarabes* Koch, 1953f: 155 [M]. Type species: *Psammodesgracilentus* Fairmaire, 1882, by original designation. Status: valid subgenus of *Psammophanes* Lesne, 1922 in Pimeliinae: Sepidiini: Molurina.

*Somaticus* Hope, 1841: 117 [M]. Type species: *Sepidiumrugosum* Fabricius, 1781, by original designation. Status: valid genus and subgenus in Pimeliinae: Sepidiini: Trachynotina. Note: *Somaticus*, introduced by Lacordaire (1859a: 197), is an incorrect subsequent spelling of the original name *Somaticum* in prevailing usage and attributed to the publication of the original spelling; we follow Kamiński et al. (2019b: 86) and treat *Somaticus* as the correct original spelling (ICZN 1999, Article 33.3.1); the First Reviser (*Somaticus* Hope, 1841 versus *Tracheloeum* Hope, 1841) is Koch (1955a: 53).

*Somocoelia* Heyden & Kraatz, 1882: 331 [F]. Type species: *Somocoeliapinguis* Heyden & Kraatz, 1882, by monotypy. Status: valid genus in Blaptinae: Platyscelidini.

*Somocoeloplatys* Skopin, 1968a: 82 [M]. Type species: *Platynoscelisboroldaica* Skopin, 1965, by original designation. Status: valid genus in Blaptinae: Platyscelidini.

*Sophrobates* Fairmaire, 1889c: xxxvi [M]. Type species: *Sophrobatesarcadii* Fairmaire, 1889, by monotypy. Status: valid genus in Stenochiinae: Cnodalonini.

*Sora* Walker, 1859: 259 [F]. Type species: *Soramarginata* Walker, 1859, by monotypy. Status: valid genus and subgenus in Lagriinae: Lagriini: Statirina.

*Soradeus* Rafinesque, 1815: 114 [M]. Type species [automatic]: *Heleaperforata* Latreille, 1816, by subsequent monotypy (Latreille 1816b: 261). Status: junior synonym of *Helea* Latreille, 1804 in Tenebrioninae: Heleini: Heleina. Note: unnecessary replacement name for *Helea* Latreille, 1804 (as “*Heleus* Latr.”).

*Spathulipezus* Gebien, 1921a: 398, 458 [M]. Type species: *Spathulipezusmiritaris* Gebien, 1921, by monotypy. Status: valid genus in Tenebrioninae: Amarygmini.

*Spectrocnera* Kwieton, 1981: 402 [F]. Type species: *Spectrocneraanguliceps* Kwieton, 1981, by original designation. Status: valid genus in Pimeliinae: Pimeliini.

*Spelaebiosis* Bousquet & Bouchard in Bousquet et al., 2018: 223 [F]. Type species [automatic]: *Orghidaniatorrei* Ardoin, 1977, by monotypy. Status: valid genus in Tenebrioninae: Triboliini. Note: replacement name for *Ardoinia* Özdikmen, 2005.

*Sphaeriontis* Casey, 1908: 56, 75 [F]. Type species: *Eusattusmuricatus* J.L. LeConte, 1851, by original designation. Status: junior synonym of *Eusattus* J.L. LeConte, 1851 in Pimeliinae: Coniontini. Synonymy: La Rivers (1949: 180).

*Sphaerocaulus* Fairmaire, 1869b: 235 [M]. Type species: *Sphaerocaulusgraniger* Fairmaire, 1869, by monotypy. Status: valid genus in Stenochiinae: Cnodalonini.

*Sphaerognathium* Dajoz, 1975a: 112 [N]. Type species: *Sphaerognathiumglobosum* Dajoz, 1975, by original designation. Status: valid genus in Diaperinae: Gnathidiini: Anopidiina.

*Sphaeromatris* Fairmaire, 1899e: 535 [F]. Type species: *Sphaeromatrisaurovittata* Fairmaire, 1899, by monotypy. Status: valid genus in Stenochiinae: Cnodalonini.

*Sphaerostibes* Koch, 1963: 64 [M]. Type species: *Sphaerostibessabulicola* Koch, 1963, by monotypy. Status: valid genus in Blaptinae: Opatrini: Stizopodina. Note: combined description of a new genus and a single new species (ICZN 1999, Article 13.4).

*Sphaerotidius* Kaszab, 1941a: 3, 38 [M]. Type species: *Sphaerotidiusduplicatus* Kaszab, 1941, by original designation. Status: valid genus in Stenochiinae: Cnodalonini. Note: we act as First Revisers and reject the alternative original spelling *Spaerotidius*, used by Kaszab (1941a: 39, 40).

*Sphaerotus* W. Kirby, 1819a: 416 [M]. Type species: *Sphaerotuscurvipes* W. Kirby, 1819, by monotypy. Status: valid genus in Stenochiinae: Cnodalonini.

*Sphargeris* Pascoe, 1860b: 122 [M]. Type species: *Sphargerisphysodes* Pascoe, 1860, by monotypy. Status: valid genus in Lagriinae: Chaerodini.

*Sphenaria* Ménétriés, 1849: 240 [F]. Type species: *Sphenariaelongata* Ménétriés, 1849, by subsequent designation (Gebien 1937a: 576). Status: valid genus in Pimeliinae: Tentyriini.

*Sphenariopsis* Kraatz, 1865: 80, 175 [F]. Type species: *Sphenariopsistristis* Kraatz, 1865, by monotypy. Status: valid subgenus of *Rhytinota* Eschscholtz, 1831 in Pimeliinae: Tentyriini.

*Spheneuphloeus* Kaszab, 1941a: 5, 35 [M]. Type species: *Osdarametallica* Pic, 1931, by original designation. Status: valid genus in Stenochiinae: Cnodalonini.

*Spheniscus* W. Kirby, 1819a: 421 [M]. Type species: *Spheniscuserotyloides* W. Kirby, 1819, by monotypy. Status: senior synonym of *Cuphotes* Champion, 1887 in Stenochiinae: Stenochiini. Note: junior homonym of *Spheniscus* Moehring, 1758 [Aves].

*Sphenogenius* Solier, 1848: 154, 351 [M]. Type species: *Sphenogeniusclathratus* Solier, 1848 (= *Nyctozoilusobesus* Guérin-Méneville, 1831), by original designation. Status: junior synonym of *Nyctozoilus* Guérin-Méneville, 1831 in Tenebrioninae: Heleini: Cyphaleina. Synonymy: Lacordaire (1859a: 350).

*Sphenolampidius* Kaszab, 1941a: 4, 40 [M]. Type species: *Sphenolampidiushemisphaericus* Kaszab, 1941, by original designation. Status: valid genus in Stenochiinae: Cnodalonini.

*Sphenosdara* Kaszab, 1941a: 2, 28 [F]. Type species: *Sphenosdarasachtlebeni* Kaszab, 1941, by original designation. Status: valid genus in Stenochiinae: Cnodalonini.

*Sphenosoma* Dejean, 1834: 212 [N]. Type species [automatic]: *Acropteronrufipes* Perty, 1832, by subsequent designation (Hope 1841: 133). Status: junior synonym of *Acropteryx* Gistel, 1831 in Tenebrioninae: Acropteronini. Note: unnecessary replacement name for *Acropteron* Perty, 1832.

*Sphenothorax* Gebien, 1906: 232 [M]. Type species: *Tenebrionitidulus* Fabricius, 1801, by monotypy. Status: junior synonym of *Zophophilus* Fairmaire, 1881 in Stenochiinae: Cnodalonini. Synonymy: Carter (1930: 547).

*Sphinctoderus* Fairmaire, 1903e: 301 [M]. Type species: *Sphinctoderusstrangulatus* Fairmaire, 1903, by monotypy. Status: valid genus in Lagriinae: Lagriini: Lagriina.

*Sphingocorse* Gebien, 1921b: 110, 111 [F]. Type species: *Sphingocorseangulicollis* Gebien, 1921, by monotypy. Status: valid genus in Lagriinae: Lupropini.

*Sphragidophorus* Champion, 1889: 61 [M]. Type species: *Statiracyanipennis* Mäklin, 1863, by subsequent designation (R. Lucas 1920: 603). Status: valid genus in Lagriinae: Lagriini: Statirina.

*Spiloscapha* Bates, 1873a: 202 [F]. Type species: *Spiloscaphacrassicornis* Bates, 1873 (= *Platydemathallioides* Pascoe, 1869), by monotypy. Status: valid genus in Diaperinae: Scaphidemini.

*Spinadaenus* Pic, 1921d: 18 [M]. Type species: *Spinadaenussingularis* Pic, 1921, by monotypy. Status: valid genus in Lagriinae: Goniaderini.

*Spinamarygmus* Pic, 1915d: 7 [M]. Type species: *Spinamarygmusindicus* Pic, 1915, by monotypy. Status: valid subgenus of *Plesiophthalmus* Motschulsky, 1857 in Tenebrioninae: Amarygmini.

*Spinanemia* Löbl, Bouchard, Merkl & Bousquet, 2020: 2 [F]. Type species: *Anemiacornuta* Pic, 1898, by original designation. Status: valid subgenus of *Cheirodes* Gené, 1839 in Tenebrioninae: Melanimonini. Note: subgenus first proposed by Ardoin (1971: 362, 378) without type species originally designated.

*Spinecula* Novák, 2019c: 437 [F]. Type species: *Spineculahouaphanica* Novák, 2019, by original designation. Status: valid genus in Alleculinae: Alleculini: Alleculina.

*Spinepicalla* Pic, 1921d: 21 [F]. Type species: *Spinepicallaarmata* Pic, 1921, by monotypy. Status: valid genus in Stenochiinae: Cnodalonini.

*Spinoderosphaerus* Pic, 1922d: 26 [M]. Type species: *Spinoderosphaerusbrevicornis* Pic, 1922, by monotypy. Status: valid genus in Stenochiinae: Cnodalonini. Note: the First Reviser (*Spinoderosphaerus* Pic, 1922 versus *Spinogauromaia* Pic, 1922) is Kaszab (1983a: 132).

*Spinodietysus* Pic, 1927b: 21 [M]. Type species: *Cyriogetonconvexipennis* Pic, 1927, by monotypy. Status: valid genus in Tenebrioninae: Amarygmini.

*Spinogauromaia* Pic, 1922a: 23 [F]. Type species: *Spinogauromaiarufescens* Pic, 1922 (= *Spinoderosphaerusbrevicornis* Pic, 1922), by monotypy. Status: junior synonym of *Spinoderosphaerus* Pic, 1922 in Stenochiinae: Cnodalonini. Synonymy: Kaszab (1983a: 132).

*Spinolagriella* Pic, 1955: 183 [F]. Type species: *Spinolagriellaminutissima* Pic, 1955, by original designation. Status: valid genus in Lagriinae: Lupropini.

*Spinolyprops* Pic, 1917d: 12 [M]. Type species: *Spinolypropsrufithorax* Pic, 1917, by monotypy. Status: valid genus in Lagriinae: Lupropini.

*Spinoodescelis* Kaszab, 1940b: 938, 966 [F]. Type species: *Platyscelissomocoeloides* Seidlitz, 1893, by original designation. Status: valid subgenus of *Oodescelis* Motschulsky, 1845 in Blaptinae: Platyscelidini.

*Spinophrynus* Koch, 1951: 90 [M]. Type species: *Phrynocolusspinipennis* Gebien, 1910, by original designation. Status: valid subgenus of *Phrynocolus* Lacordaire, 1859 in Pimeliinae: Sepidiini: Molurina.

*Spinorhacus* Kaszab, 1969a: 262 [M]. Type species: *Spinorhacusbaloghi* Kaszab, 1969, by original designation. Status: junior synonym of *Spinolagriella* Pic, 1955 in Lagriinae: Lupropini. Synonymy: Kaszab (1976: 453).

*Spinosdara* Bouchard & Bousquet, **new subgenus** [F]. Type species: *Osdarabiroi* Kaszab, 1939, by **present designation**. Status: valid subgenus of *Osdara* Walker, 1858 in Stenochiinae: Cnodalonini. Note: Kaszab (1941a: 33) introduced the new subgenus name *Spinosdara* for three nominal species, but unfortunately did not designate a type species; the subgenusSpinosdara, which has been treated as valid since 1941, is therefore unavailable (ICZN 1999, Article 13.3); we hereby make the name available by selecting *Osdarabiroi* Kaszab, 1939 as type species and referring to Kaszab (1941a: 33) for the character states that characterise and differentiate *Spinosdara*.

*Spinostatira* Pic, 1918b: 22 [F]. Type species: *Statiraspinipes* Pic, 1918, by subsequent designation (Borchmann 1936: 247). Status: valid subgenus of *Statira* Lepeletier & Audinet-Serville, 1828 in Lagriinae: Lagriini: Statirina.

*Splenoodescelis* Egorov, 2004: 593 [F]. Type species: *Platyscelisturkestanica* Seidlitz, 1893, by original designation. Status: valid subgenus of *Oodescelis* Motschulsky, 1845 in Blaptinae: Platyscelidini.

*Splichalia* Reitter, 1913: 664 [F]. Type species: *Splichaliatigrinella* Reitter, 1913, by monotypy. Status: valid genus in Lagriinae: Lagriini: Statirina.

*Spongesmia* Bouchard & Bousquet, **new subgenus** [F]. Type species: *Adesmianassata* Erichson, 1843, by **present designation**. Status: valid subgenus of *Adesmia* Fischer, 1822 in Pimeliinae: Adesmiini. Note: Koch (1944b: 149) introduced the new subgenus name *Spongesmia* for four nominal species, but unfortunately did not designate a type species; the subgenusSpongesmia, which has been treated as valid since 1944, is therefore unavailable (ICZN 1999, Article 13.3); we hereby make the name available by selecting *Adesmianassata* Erichson, 1843 as type species and referring to Koch (1944b: 149) for the character states that characterise and differentiate *Spongesmia*.

*Spongesmima* Koch, 1944b: 157 [F]. Type species: *Adesmiascrobipennis* Haag-Rutenberg, 1875, by monotypy. Status: junior synonym of *Renatiella* Koch, 1944 in Pimeliinae: Adesmiini. Synonymy: Penrith (1979: 27).

*Spyrathus* Kraatz, 1865: 6, 9 [M]. Type species: *Spyrathusindicus* Kraatz, 1865, by monotypy. Status: valid genus in Pimeliinae: Erodiini.

*Srilanka* Kaszab, 1980b: 319 [F]. Type species: *Srilankamirabilis* Kaszab, 1980, by original designation. Status: valid genus in Stenochiinae: Cnodalonini. Note: *Srilanka* was used earlier by Kaszab (1979a: 107) without a description, a definition, or a bibliographic reference to such a published statement (ICZN 1999, Article 13.1) and is therefore unavailable from that date.

*Staius* Fairmaire, 1896b: 359 [M]. Type species: *Staiusmiricornis* Fairmaire, 1896, by monotypy. Status: valid genus and subgenus in Lagriinae: Lagriini: Statirina.

*Stalagmoptera* Solsky, 1876: 286 [F]. Type species: *Stalagmopteratuberculatocostata* Solsky, 1876, by subsequent designation (Gebien 1937a: 821). Status: valid genus in Pimeliinae: Pimeliini.

*Statira* Lepeletier & Audinet-Serville, 1828: 479 [F]. Type species: *Statiraagroides* Lepeletier & Audinet-Serville, 1828, by subsequent designation (Blanchard 1844: pl. 53bis). Status: valid genus and subgenus in Lagriinae: Lagriini: Statirina.

*Statiropsis* Borchmann, 1912b: 389 [F]. Type species: *Statiropsisaenea* Borchmann, 1912, by monotypy. Status: valid genus in Lagriinae: Lagriini: Statirina.

*Stegastopsis* Kraatz, 1865: 80, 176 [F]. Type species: *Stegastopsisbabylonica* Kraatz, 1865, by monotypy. Status: valid genus and subgenus in Pimeliinae: Tentyriini.

*Steira* Westwood, 1837: pl. 176 [F]. Type species: *Steiracostata* Westwood, 1837, by monotypy. Status: senior synonym of *Stips* Koch, 1950 in Pimeliinae: Adelostomini. Note: junior homonym of *Steira* Eschscholtz, 1825 [Mollusca].

*Stemmatoderus* Agassiz, 1846b: 351 [M]. Type species [automatic]: *Stemmoderussingularis* Spinola, 1842, by monotypy. Status: junior synonym of *Stemmoderus* Spinola, 1842 in Tenebrioninae: Amarygmini. Note: unjustified emendation of *Stemmoderus* Spinola, 1842, not in prevailing usage.

*Stemmoderus* Spinola, 1842: pl. 91 (p. 1) [M]. Type species: *Stemmoderussingularis* Spinola, 1842, by monotypy. Status: valid genus in Tenebrioninae: Amarygmini.

*Stenadelium* Watt, 1992: 32 [N]. Type species: *Stenadeliumstriatum* Watt, 1992, by original designation. Status: valid genus in Lagriinae: Adeliini.

*Stene* Stephens, 1829: 19 [F]. Type species: **fixed herein** (ICZN 1999, Article 70.3) as *Colydiumcastaneum* Herbst, 1797, misidentified as *Tenebrioferrugineus* Fabricius, 1781 in the original designation by monotypy in Stephens (1829). Status: junior synonym of *Tribolium* W.S. MacLeay, 1825 in Tenebrioninae: Triboliini. Synonymy: Shuckard (1840: vii). Note: the type species “*Tenebrioferrugineus* Fabricius” was first established by monotypy; as noted by C.O. Waterhouse (1896: 230) and Blair (1913: 223) the *Tenebrioferrugineus* Fabricius of authors, including Stephens (1829: 19, as “*ferruginea*, Oliv.”), was misidentified; Blair (1913: 223) noted that the species the authors referred to is in fact *Colydiumcastaneum* Herbst, 1797; we follow currently accepted concepts (e.g., Bousquet et al. 2018: 224) and fix the type species according to the requirements of Article 70.3.2 (ICZN 1999); the nominal species *Tenebrioferrugineus* Fabricius, 1781 is a valid species in the genus *Tribolioides* Blair, 1913 [Coleoptera: Cucujidae].

*Steneleodes* Blaisdell, 1909: 409 [M]. Type species: *Eleodeslongicollis* J.L. LeConte, 1851, by subsequent designation (Bousquet et al. 2018: 166). Status: valid subgenus in *Eleodes* Eschscholtz, 1829 in Blaptinae: Amphidorini.

*Stenerophlina* Reitter, 1906b: 130 [F]. Type species: *Omophlinahauseri* Reitter, 1894, by monotypy. Status: valid genus in Alleculinae: Cteniopodini.

*Stenerula* Fairmaire, 1875: 42 [F]. Type species: *Stenerulasubopaca* Fairmaire, 1875, by monotypy. Status: valid genus in Alleculinae: incertae sedis.

*Steneryx* Reitter, 1890b: 256 [M]. Type species: *Cisteladejeanii* Faldermann, 1836, by monotypy. Status: valid genus in Alleculinae: Cteniopodini.

*Stenethmus* Gebien, 1937b: 41 [M]. Type species: *Psammodestentyriniformis* Hesse, 1935, by original designation. Status: valid genus in Pimeliinae: Sepidiini: Oxurina.

*Steneucyrtus* Fairmaire, 1896a: 31 [M]. Type species: *Steneucyrtuspexicollis* Fairmaire, 1896, by monotypy. Status: valid genus in Stenochiinae: Cnodalonini.

*Stenholma* Solier, 1835b: 253, 412 [F]. Type species: *Stenholmatentyrioides* Solier, 1835, by monotypy. Status: junior synonym of *Melaphorus* Guérin-Méneville, 1834 in Pimeliinae: Evaniosomini. Synonymy: Solier (1835b: 412).

*Stenillus* Blair, 1927: 245 [M]. Type species: *Stenillusmonticola* Blair, 1927, by monotypy. Status: valid subgenus of *Pseudethas* Fairmaire, 1896 in Pimeliinae: Stenosini: Dichillina.

*Stenocara* Solier, 1835b: 512, 553 [N]. Type species: *Pimelialongipes* Fabricius, 1775, by subsequent designation (Desmarest 1860: 141). Status: valid genus and subgenus in Pimeliinae: Adesmiini.

*Stenocephalus* Agassiz, 1846b: 71, 351 [M]. Type species [automatic]: *Cephalostenusdejeanii* Solier, 1838 (= *Scauruselegans* Brullé, 1832), by subsequent designation (Hope 1841: 115). Status: junior synonym of *Cephalostenus* Solier, 1838 in Tenebrioninae: Scaurini. Note: unjustified emendation of *Cephalostenus* Solier, 1838; junior homonym of *Stenocephalus* Tschudi, 1838 [Amphibia].

*Stenocera* Agassiz, 1846b: 74, 351 [F]. Type species [automatic]: *Cerostenadeplanata* Solier, 1836, by **present designation**. Status: junior synonym of *Psectrascelis* Solier, 1836 in Pimeliinae: Nycteliini. Note: unjustified emendation of *Cerostena* Solier, 1836, not in prevailing usage; junior homonym of *Stenocera* Brullé, 1834 [Coleoptera: Carabidae].

*Stenochia* W. Kirby, 1819a: 423 [F]. Type species: *Stenochiarufipes* W. Kirby, 1819, by subsequent designation (Hope 1841: 133). Status: junior synonym of *Strongylium* W. Kirby, 1819 in Stenochiinae: Stenochiini. Synonymy: Latreille (1829b: 683).

*Stenochidus* J.L. LeConte, 1862: 244 [M]. Type species: *Stenochiagracilis* J.L. LeConte, 1851, by subsequent designation (R. Lucas 1920: 608). Status: valid genus in Alleculinae: Alleculini: Alleculina.

*Stenochinus* Motschulsky, 1860a: 102 [M]. Type species: *Stenochinusreticulatus* Motschulsky, 1860, by monotypy. Status: valid genus in Stenochiinae: Cnodalonini.

*Stenodesia* Reitter, 1916a: 4 [F]. Type species: *Stenocaraglobulum* Haag-Rutenberg, 1875, by monotypy. Status: valid genus in Pimeliinae: Adesmiini.

*Stenogena* Fairmaire, 1895a: 33 [F]. Type species: *Stenogenamadecassa* Fairmaire, 1895, by monotypy. Status: valid genus in Alleculinae: Alleculini: Alleculina. Note: placed in the subfamily Alleculinae by Chatanay (1915a: 526).

*Stenogenomorpha* Pic, 1919b: 5 [F]. Type species: *Stenogenomorphaimpressa* Pic, 1919, by monotypy. Status: valid genus in Alleculinae: incertae sedis.

*Stenogonopus* Gebien, 1938b: 91 [M]. Type species: *Tenebrioplumosus* Thunberg, 1787, by original designation. Status: valid genus in Blaptinae: Platynotini: Platynotina.

*Stenohelops* Reitter, 1922a: 22 [M]. Type species: *Isopedusplicatulus* Kraatz, 1880, by subsequent designation (Gebien 1943: 423). Status: valid genus and subgenus in Tenebrioninae: Helopini: Helopina. Note: the First Revisers (*Stenohelops* Reitter, 1922 versus *Gunarellus* Reitter, 1922) are Nabozhenko et al. (2020b: 297).

*Stenolagria* Merkl, 1987: 124, 157 [F]. Type species: *Stenolagriamatthewsi* Merkl, 1987, by original designation. Status: valid genus in Lagriinae: Lagriini: Lagriina.

*Stenolamus* Gebien, 1920: 107 [M]. Type species: *Stenolamussulciceps* Gebien, 1920, by subsequent designation (Gebien 1938a: 397). Status: valid genus in Blaptinae: incertae sedis. Note: this taxon is placed in “Blaptinae incertae sedis” based on comments in Kamiński et al. (2021b: 150).

†*Stenolassus* Nabozhenko, Chigray & Bukejs, 2020a: 519 [M]. Type species: *Nalassusklebsi* Nabozhenko, Perkovsky & Chernei, 2016, by original designation. Status: valid subgenus of *Stenohelops* Reitter, 1922 in Tenebrioninae: Helopini: Helopina. Note: described from Eocene Baltic amber.

*Stenomacidius* Seidlitz, 1895: 791 [M]. Type species: *Hedyphanesacutangulus* Seidlitz, 1895, by subsequent designation (G.S. Medvedev 1990: 242). Status: junior synonym of *Cylindrinotus* Faldermann, 1837 in Tenebrioninae: Helopini: Cylindrinotina. Synonymy: Nabozhenko (2006: 799).

*Stenomaleis* Español, 1957b: 21, 34 [M]. Type species: *Stenohelopsardoini* Español, 1957 (= *Helopsprotensulus* Seidlitz, 1896), by original designation. Status: junior synonym of *Helopelius* Reitter, 1922 in Tenebrioninae: Helopini: Helopina. Synonymy: Nabozhenko et al. (2020b: 293).

*Stenomax* Allard, 1876a: 4 [M]. Type species: *Tenebriolanipes* Linnaeus, 1771 (= *Tenebrioaeneus* Scopoli, 1763), by subsequent designation (Español and Comas 1989: 166, 170). Status: valid genus and subgenus in Tenebrioninae: Helopini: Cylindrinotina.

*Stenomorpha* Solier, 1836: 407, 487 [F]. Type species: *Stenomorphablapsoides* Solier, 1836, by subsequent designation (Desmarest 1860: 150). Status: valid genus and subgenus in Pimeliinae: Asidini.

*Stenopalorus* Blair, 1930: 135 [M]. Type species: *Palorushypophloeoides* Blair, 1930, by monotypy. Status: junior synonym of *Palorus* Mulsant, 1854 in Tenebrioninae: Palorini. Synonymy: Halstead (1967a: 114, 115).

*Stenophanes* Solsky, 1876: 294 [M]. Type species: *Hedyphanesmesostenus* Solsky, 1871, by original designation. Status: valid genus in Stenochiinae: Cnodalonini.

*Stenophloeus* Blair, 1921: 1 [M]. Type species: *Hypophlaeusfilum* Fairmaire, 1893, by subsequent designation (Löbl et al. 2008b: 312). Status: valid subgenus of *Corticeus* Piller & Mitterpacher, 1783 in Diaperinae: Hypophlaeini.

*Stenopsis* Rafinesque, 1815: 113 [F]. Type species [automatic]: *Pimeliareflexa* Fabricius, 1775, by subsequent designation (Latreille 1810: 429). Status: junior synonym of *Akis* Herbst, 1799 in Pimeliinae: Akidini. Note: unnecessary replacement name for *Akis* Herbst, 1799.

*Stenoscapha* Bates, 1873b: 237 [F]. Type species: *Stenoscaphatibialis* Bates, 1873, by monotypy. Status: valid genus in Diaperinae: Diaperini: Diaperina.

*Stenoscapha* Fairmaire, 1885b: 234 [F]. Type species: *Stenoscaphaspissicornis* Fairmaire, 1885, by monotypy. Status: senior synonym of *Brachypophlaeus* Fairmaire, 1897 in Tenebrioninae: Ulomini. Note: junior homonym of *Stenoscapha* Bates, 1873 [Coleoptera: Tenebrionidae: Diaperinae: Diaperini: Diaperina].

*Stenosethas* Kaszab, 1975a: 11 [M]. Type species: *Stenosethascarinipennis* Kaszab, 1975, by original designation. Status: valid genus in Pimeliinae: Stenosini: Stenosina.

*Stenosida* Solier, 1835b: 253, 281 [F]. Type species: *Stenosidatenuicollis* Solier, 1835 (= *Tageniastriatopunctata* Wiedemann, 1821), by monotypy. Status: valid genus in Pimeliinae: Tentyriini.

*Stenosides* Solier, 1836: 406, 484 [M]. Type species: *Stenosidesgraciliformis* Solier, 1836, by monotypy. Status: valid subgenus of *Pelecyphorus* Solier, 1836 in Pimeliinae: Asidini.

*Stenosidops* Koch, 1940b: 733 [M]. Type species: *Tageniasabulosa* Guérin-Méneville, 1849, by monotypy. Status: valid subgenus of *Stenosis* Herbst, 1799 in Pimeliinae: Stenosini: Stenosina.

*Stenosis* Herbst, 1799: 160 [F]. Type species: **fixed herein** (ICZN 1999, Article 70.3) as *Tageniaintermedia* Solier, 1838, misidentified as *Pimeliaangustata* Fabricius, 1775 in the subsequent designation in Lacordaire (1859a). Status: valid genus and subgenus in Pimeliinae: Stenosini: Stenosina. Note: the type species “*Pimeliaangustata* Fabricius” (as *angustata* Herbst) was selected by Lacordaire (1859a: 102) as the type species; Baudi de Selve (1875: 67) first noted that *Pimeliaangustata* Fabricius of Herbst (1799) was misidentified and corresponded to *Tageniaintermedia* Solier, 1838; we follow currently accepted concepts (e.g., Löbl et al. 2008b: 179) and fix the type species according to the requirements of Article 70.3.2 (ICZN 1999); the nominal species *Pimeliaangustata* Fabricius, 1775 is a valid species in the genus *Mesostena* Eschscholtz, 1831 [Coleoptera: Tenebrionidae].

*Stenothesilea* Kulzer, 1951a: 131, 134 [F]. Type species: *Stenothesileacylindriformis* Kulzer, 1951, by monotypy. Status: valid genus in Stenochiinae: Cnodalonini.

*Stenotrichus* J.L. LeConte, 1862: 239 [M]. Type species: *Amphidorarufipes* J.L. LeConte, 1851, by original designation. Status: junior synonym of *Helops* Fabricius, 1775 in Tenebrioninae: Helopini: Helopina. Synonymy: Aalbu et al. (2002: 496).

*Steptochora* Koch, 1952b: 10 [F]. Type species: *Eurychorasetosula* Fairmaire, 1887, by original designation. Status: valid genus in Pimeliinae: Adelostomini.

*Sterces* Champion, 1891: 640 [M]. Type species: *Stercesviolaceipennis* Champion, 1891, by **present designation**. Status: junior synonym of *Alcyonotus* Pascoe, 1882 in Stenochiinae: Cnodalonini. Synonymy: Champion (1894b: lxiii).

*Steriphanides* Casey, 1907: 515 [M]. Type species: *Emmenastusstolidus* Champion, 1892, by monotypy. Status: valid genus in Pimeliinae: Edrotini.

*Steriphanus* Casey, 1907: 289 [M]. Type species: *Emmenastusconicicollis* Casey, 1890, by original designation. Status: valid genus in Pimeliinae: Edrotini.

*Sternocnera* Skopin, 1964: 394 [F]. Type species: *Sternocneralindti* Skopin, 1964, by original designation. Status: valid genus in Pimeliinae: Pimeliini.

*Sternodes* Fischer von Waldheim, 1837: 10 [M]. Type species: *Sternodeskarelini* Fischer von Waldheim, 1837 (= *Tenebriocaspicus* Pallas, 1781), by monotypy. Status: valid genus in Pimeliinae: Pimeliini.

*Sternomaia* Kulzer, 1952: 731 [F]. Type species: *Sternomaiacoeruleovirens* Kulzer, 1952, by original designation. Status: valid genus in Stenochiinae: Cnodalonini.

*Sternoplax* Frivaldszky, 1890: 207 [F]. Type species: *Trigonoscelisszechenyii* Frivaldszky, 1890, by monotypy. Status: valid genus and subgenus in Pimeliinae: Pimeliini.

*Sternotrigon* Skopin, 1973: 109, 110 [M]. Type species: *Trigonoscelissetosa* Bates, 1879, by original designation. Status: valid genus in Pimeliinae: Pimeliini.

*Stethasida* Casey, 1912: 78, 203 [F]. Type species: *Pelecyphorusmuricatulus* J.L. LeConte, 1851, by original designation. Status: valid subgenus of *Stenomorpha* Solier, 1836 in Pimeliinae: Asidini.

*Stethotrypes* Gebien, 1914c: 26 [M]. Type species: *Stethotrypesbicornutus* Gebien, 1914, by subsequent designation (Gebien 1940: 431). Status: valid genus in Diaperinae: Leiochrinini.

*Sthenoboea* Champion, 1885: 112 [F]. Type species: *Sthenoboeaapicalis* Champion, 1885, by monotypy. Status: valid genus in Stenochiinae: Cnodalonini.

*Stibia* Horn, 1870: 258, 260 [F]. Type species: *Stibiapuncticollis* Horn, 1870, by monotypy. Status: valid genus in Pimeliinae: Edrotini.

*Stictodera* Casey, 1907: 289, 352 [F]. Type species: *Emmenastuspinguis* J.L. LeConte, 1866, by original designation. Status: valid genus in Pimeliinae: Edrotini.

*Stictodere* Gebien, 1928: 101 [F]. Type species: *Stictoderesubseriata* Gebien, 1928, by subsequent designation (Gebien 1937a: 571). Status: valid genus in Pimeliinae: Epitragini.

*Stictoderia* Gebien, 1937a: 571 [F]. Type species [automatic]: *Stictoderesubseriata* Gebien, 1928, by subsequent designation (Gebien 1937a: 571). Status: junior synonym of *Stictodere* Gebien, 1928 in Pimeliinae: Epitragini. Note: unnecessary replacement name for *Stictodere* Gebien, 1928, which is not a homonym of *Stictodera* Casey, 1907 [Coleoptera: Tenebrionidae: Pimeliinae: Edrotini].

*Stierlinius* Forel, 1893: 167 [M]. Type species [automatic]: *Dolichoderusacuminatus* Klug, 1833, by monotypy. Status: junior synonym of *Macellocerus* Solier, 1848 in Tenebrioninae: Toxicini: Nycteropina. Note: replacement name for *Dolichoderus* Klug, 1833.

*Stigmatoma* J.L. LeConte, 1862: 244 [F]. Type species: *Cistelafraterna* Say, 1824, by monotypy. Status: junior synonym of *Ernocharis* C.G. Thomson, 1859 in Alleculinae: Alleculini: Mycetocharina. Synonymy: Seidlitz (1896: 147).

*Stilbocistela* Borchmann, 1932a: 319 [F]. Type species: *Stilbocistelaluzonica* Borchmann, 1932, by original designation. Status: valid genus in Alleculinae: Alleculini: Alleculina. Note: genus described as new again by Borchmann (1932b: 125).

*Stips* Koch, 1950a: 66 [F]. Type species [automatic]: *Steiracostata* Westwood, 1837, by monotypy. Status: valid genus in Pimeliinae: Adelostomini. Note: replacement name for *Steira* Westwood, 1837.

*Stipsostoma* Koch, 1952b: 34, 105 [N]. Type species: *Steirasculpta* Gebien, 1920, by original designation. Status: valid genus in Pimeliinae: Adelostomini.

*Stira* Agassiz, 1846b: 350, 353 [F]. Type species [automatic]: *Steiracostata* Westwood, 1837, by monotypy. Status: senior synonym of *Stips* Koch, 1950 in Pimeliinae: Adelostomini. Note: unjustified emendation of *Steira* Westwood, 1837 (and *Steira* Eschscholtz, 1825 in Mollusca), not in prevailing usage; we act as First Revisers and treat *Stira* Agassiz, 1846 [Coleoptera] as a junior homonym of *Stira* Agassiz, 1846 [Mollusca].

*Stizopus* Erichson, 1843: 245 [M]. Type species: *Stizopuslaticollis* Erichson, 1843, by monotypy. Status: valid genus in Blaptinae: Opatrini: Stizopodina.

*Stomion* G.R. Waterhouse, 1845a: 27 [N]. Type species: *Stomiongalapagoense* G.R. Waterhouse, 1845, by subsequent designation (Gebien 1937a: 590). Status: valid genus in Pimeliinae: Edrotini.

*Stomium* Agassiz, 1846b: 354 [N]. Type species [automatic]: *Stomiongalapagoense* G.R. Waterhouse, 1845, by subsequent designation (Gebien 1937a: 590). Status: junior synonym of *Stomion* G.R. Waterhouse, 1845 in Pimeliinae: Edrotini. Note: unjustified emendation of *Stomion* G.R. Waterhouse, 1845, not in prevailing usage.

*Stomylus* Fåhraeus, 1870: 300 [M]. Type species: *Stomylusbicolor* Fåhraeus, 1870, by subsequent designation (R. Lucas 1920: 616). Status: valid genus in Diaperinae: Diaperini: Diaperina.

*Stonavus* Reitter, 1904: 161 [M]. Type species: *Penthicusalaiensis* Reitter, 1896, by subsequent designation (Iwan and Löbl 2007: 734). Status: junior synonym of *Penthicus* Faldermann, 1836 in Blaptinae: Opatrini: Opatrina. Synonymy: Iwan and Löbl (2007: 734).

*Storthephora* Mäklin, 1875: 658 [F]. Type species: *Storthephoradenticollis* Mäklin, 1875, by subsequent designation (Bousquet and Bouchard 2014: 26). Status: junior synonym of *Paratenetus* Spinola, 1845 in Lagriinae: Goniaderini. Synonymy: Champion (1893b: 47).

*Storthocnemis* Karsch, 1881: 47 [F]. Type species: *Storthocnemissteckeri* Karsch, 1881, by original designation. Status: valid genus in Pimeliinae: Pimeliini.

*Stratodemus* Gebien, 1921b: 98 [M]. Type species: *Stratodemusheraldicus* Gebien, 1921, by monotypy. Status: valid genus in Lagriinae: Pycnocerini.

*Strepsius* Fairmaire, 1896b: 351 [M]. Type species: *Strepsiusspretulus* Fairmaire, 1896, by monotypy. Status: valid genus in Stenochiinae: Cnodalonini.

*Stridigula* Koch, 1954a: 60 [F]. Type species: *Stridigulaarguta* Koch, 1954, by original designation. Status: valid genus in Blaptinae: Platynotini: Eurynotina.

*Stridulomus* Koch, 1955a: 37 [M]. Type species: *Psammodessulcicollis* Péringuey, 1885, by monotypy. Status: valid genus in Pimeliinae: Sepidiini: Molurina.

*Strongyallecula* Pic, 1955: 186 [F]. Type species: *Strongyalleculabasilewskyi* Pic, 1955, by original designation. Status: valid genus in Alleculinae: incertae sedis.

*Strongylacanthus* Brèthes, 1925: 13 [M]. Type species: *Strongylacanthusperuvianus* Brèthes, 1925, by monotypy. Status: valid genus in Stenochiinae: Stenochiini.

*Strongylagria* Pic, 1915b: 4 [F]. Type species: *Strongylagriametallica* Pic, 1915, by monotypy. Status: valid genus in Lagriinae: Lagriini: Statirina.

*Strongyliastrum* Fairmaire, 1894a: 39 [N]. Type species: *Strongyliastrumbraetii* Fairmaire, 1894 (= *Strongyliumrufipenne* Kollar & Redtenbacher, 1844), by monotypy. Status: junior synonym of *Strongylium* W. Kirby, 1819 in Stenochiinae: Stenochiini. Synonymy: Gebien (1948: 519).

*Strongylium* W. Kirby, 1819a: 417 [N]. Type species: *Strongyliumchalconatum* W. Kirby, 1819, by monotypy. Status: valid genus and subgenus in Stenochiinae: Stenochiini. Note: nomen protectum (see Bouchard et al. 2005: 501); the First Reviser (*Strongylium* W. Kirby, 1819 versus *Stenochia* W. Kirby, 1819) is Latreille (1829b: 683); we recognize two valid subgenera in the diverse genus *Strongylium* W. Kirby, 1819, i.e., the recently described *Afrostrongylium* Robiche, 2019 and the nominotypical subgenus; however, much taxonomic research is needed to evaluate the possible validity of the many synonyms of the subgenusStrongylium W. Kirby, 1819.

*Strophia* Robiche, 2004b: 130 [F]. Type species: *Oncosomaertli* Gebien, 1910, by original designation. Status: senior synonym of *Strophiamixa* Robiche, 2005 in Blaptinae: Pedinini: Helopinina. Note: junior homonym of *Strophia* Meigen, 1832 [Lepidoptera] and *Strophia* Albers, 1850 [Mollusca].

*Strophiamixa* Robiche, 2005: 358 [F]. Type species [automatic]: *Oncosomaertli* Gebien, 1910, by original designation. Status: valid subgenus of *Amatodes* Dejean, 1834 in Blaptinae: Pedinini: Helopinina. Note: replacement name for *Strophia* Robiche, 2004.

*Stygohelops* Leo & Liberto, 2003: 299 [M]. Type species: *Probaticuskalavriticus* Schawaller, 2001, by original designation. Status: valid genus in Tenebrioninae: Helopini: Cylindrinotina.

*Styphacus* Fairmaire, 1901a: 71 [M]. Type species: *Styphacusdecorsii* Fairmaire, 1901, by subsequent designation (R. Lucas 1920: 619). Status: valid genus in Blaptinae: Platynotini: Platynotina.

*Styphloeus* Kaszab, 1941a: 4, 36 [M]. Type species: *Styphloeusindicus* Kaszab, 1941, by original designation. Status: valid genus in Stenochiinae: Cnodalonini.

*Styrax* Westwood, 1875: 227 [M]. Type species: *Styraxtricondyloides* Westwood, 1875, by monotypy. Status: junior synonym of *Strongylium* W. Kirby, 1819 in Stenochiinae: Stenochiini. Synonymy: Gebien (1948: 519).

*Styrus* Bates, 1873e: 348 [M]. Type species: *Styruselongatulus* Bates, 1873, by monotypy. Status: valid genus in Tenebrioninae: Heleini: Cyphaleina.

*Suarezius* Fairmaire, 1895a: 22 [M]. Type species: *Suareziusgibbosulus* Fairmaire, 1895, by monotypy. Status: valid genus in Stenochiinae: Cnodalonini.

*Subalphasida* Escalera, 1928: 136 [F]. Type species: *Asidaluctuosa* Boisduval, 1835, by original designation. Status: junior synonym of *Betasida* Reitter, 1917 in Pimeliinae: Asidini. Synonymy: Viñolas and Cartagena (2005: 282).

*Subpterocoma* Bouchard & Bousquet, **new replacement name** [F]. Type species [automatic]: *Pterocomatuberculata* Motschulsky, 1845, by subsequent designation (Skopin 1974b: 159). Status: valid subgenus of *Pterocoma* Dejean, 1834 in Pimeliinae: Pimeliini. Note: replacement name for *Pseudopimelia* Motschulsky, 1860.

*Sulcipectus* Louw, 1979: 99, 109 [N]. Type species: *Sulcipectusleve* Louw, 1979, by original designation. Status: valid genus in Pimeliinae: Sepidiini: Hypomelina.

*Sulcolagria* Pic, 1955: 181 [F]. Type species: *Sulcolagriasemiopaca* Pic, 1955, by original designation. Status: valid genus in Lagriinae: Lagriini: Lagriina.

*Sulcosis* Penrith, 1977: 19, 220 [F]. Type species: *Zophosisangolensis* Erichson, 1843, by original designation. Status: valid subgenus of *Zophosis* Latreille, 1802 in Pimeliinae: Zophosini.

*Sulpius* Fairmaire, 1906: 273 [M]. Type species: *Sulpiuspunctostriatus* Fairmaire, 1906, by monotypy. Status: valid genus in Blaptinae: Opatrini: Stizopodina.

*Sulpiusoma* Ferrer, 2006c: 81 [N]. Type species: *Sulpiusomamedquisti* Ferrer, 2006, by monotypy. Status: valid genus in Stenochiinae: Cnodalonini. Note: we act as First Revisers and reject the alternative original spelling *Sulpiosoma*, used by Ferrer (2006c: 79).

*Sumbawia* Gebien, 1925b: 441 [F]. Type species: *Sumbawiatetrops* Gebien, 1925, by monotypy. Status: valid genus in Tenebrioninae: Bolitophagini.

*Sundon* Pic, 1923d: 18 [N]. Type species: *Sundonatricorne* Pic, 1923, by monotypy. Status: valid genus in Stenochiinae: Cnodalonini.

*Syachis* Bates, 1879b: 467 [M]. Type species: *Syachishimalaicus* Bates, 1879, by subsequent designation (Gebien 1937a: 594). Status: valid genus in Pimeliinae: Tentyriini. Note: redescribed as new by Bates (1890: 55).

*Sycophantes* Kirsch, 1866: 198 [M]. Type species: *Sycophantesdentipes* Kirsch, 1866, by subsequent designation (Gebien 1928: 203). Status: valid genus in Stenochiinae: Cnodalonini.

*Sycophantomorphus* Pic, 1924b: 13 [M]. Type species: *Sycophantomorphusater* Pic, 1924, by monotypy. Status: valid genus in Stenochiinae: Cnodalonini.

*Syggona* Fåhraeus, 1870: 330 [F]. Type species: *Syggonaconcinna* Fåhraeus, 1870, by monotypy. Status: junior synonym of *Luprops* Hope, 1833 in Lagriinae: Lupropini. Synonymy: Ferrer (1995b: 62).

*Sylvanoplonyx* Bremer, 2010: 156, 157 [M]. Type species: *Sylvanoplonyxfemoralis* Bremer, 2010, by original designation. Status: valid genus in Tenebrioninae: Amarygmini.

*Sympetes* Pascoe, 1866a: 464 [M]. Type species: *Sympetesmacleayi* Pascoe, 1866 (= *Emcephalustricostellus* White, 1841), by subsequent designation (Gebien 1940: 1075). Status: valid genus in Tenebrioninae: Heleini: Heleina.

*Symphochora* Koch, 1952b: 12 [F]. Type species: *Eurychorahumerifera* Gebien, 1937, by original designation. Status: valid genus in Pimeliinae: Adelostomini.

*Symphoxycara* Koch, 1943a: 577 [N]. Type species: *Oxycarabreviusculum* Fairmaire, 1892, by monotypy. Status: valid subgenus of *Oxycara* Solier, 1835 in Pimeliinae: Tentyriini.

*Sympiezocnemis* Solsky, 1876: 290 [F]. Type species: *Sympiezocnemiskessleri* Solsky, 1876, by subsequent designation (Löbl et al. 2008b: 164). Status: junior synonym of *Pisterotarsa* Motschulsky, 1860 in Pimeliinae: Pimeliini. Synonymy: Heyden (1882: 140).

*Synallecula* Kolbe, 1883: 25 [F]. Type species: *Alleculalivida* Sahlberg, 1823, by subsequent designation (R. Lucas 1920: 621). Status: valid genus in Alleculinae: Alleculini: Alleculina. Note: raised from a subgenus of *Alogista* Fåhraeus, 1870 to valid genus by Novák (2017b: 130).

*Synatractus* W.J. MacLeay, 1887: 312 [M]. Type species: *Synatractusvariabilis* W.J. MacLeay, 1887, by monotypy. Status: junior synonym of *Casnonidea* Fairmaire, 1882 in Lagriinae: Lagriini: Statirina. Synonymy: Carter (1920b: 199), Merkl (1986: 188).

*Syncolydium* Kolbe, 1897a: 110 [N]. Type species: *Syncolydiumglabratum* Kolbe, 1897, by monotypy. Status: junior synonym of *Corticeus* Piller & Mitterpacher, 1783 in Diaperinae: Hypophlaeini. Synonymy: Bremer (1995: 3). Note: genus originally described in Tenebrionoidea: Colydiidae.

*Syngona* Rye, 1873: 293 [F]. Type species [automatic]: *Syggonaconcinna* Fåhraeus, 1870, by monotypy. Status: junior synonym of *Luprops* Hope, 1833 in Lagriinae: Lupropini. Note: unjustified emendation of *Syggona* Fåhraeus, 1870, not in prevailing usage.

*Synhimba* Koch, 1952d: 216 [N]. Type species: *Psammodescordiformis* Haag-Rutenberg, 1871, by original designation. Status: valid genus in Pimeliinae: Sepidiini: Oxurina.

*Synquadrideres* Iwan, 2003a: 182 [M]. Type species: *Synquadrideresnaivashaensis* Iwan, 2003, by original designation. Status: junior synonym of *Glyptopteryx* Gebien, 1910 in Blaptinae: Platynotini: Platynotina. Synonymy: Kamiński (2015a: 91).

*Syntyphlus* Koch, 1953e: 243 [M]. Type species: *Syntyphlussubterraneus* Koch, 1953, by original designation. Status: valid genus in Blaptinae: Opatrini: Stizopodina.

*Szentivanya* Kaszab, 1958: 1 [F]. Type species: *Szentivanyametasternalis* Kaszab, 1958, by original designation. Status: valid genus in Diaperinae: Gnathidiini: Gnathidiina.

*Tabarus* Gebien, 1921a: 317 [M]. Type species: *Tabarusinfernalis* Gebien, 1921, by subsequent designation (Gebien 1940: 1089). Status: valid genus in Stenochiinae: Cnodalonini.

*Taclamacanius* Ferrer & Yvinec, 2005: 121 [M]. Type species [automatic]: *Taklamakanialepetzi* Ferrer & Yvinec, 2004, by monotypy. Status: junior synonym of *Paranemia* Heyden, 1892 in Diaperinae: Phaleriini. Synonymy: G.S. Medvedev (2006: 557). Note: replacement name for *Taklamakania* Ferrer & Yvinec, 2004.

*Tactoderus* Fairmaire, 1892a: 112 [M]. Type species: *Tactoderussubopacus* Fairmaire, 1892 (= *Praogenagagatina* Mäklin, 1863), by monotypy. Status: junior synonym of *Praeugena* Laporte, 1840 in Tenebrioninae: Praeugenini. Synonymy: Gebien (1948: 544).

*Tadzhikistania* Bogatchev, 1960b: 35 [F]. Type species: *Tadzhikistaniamystacea* Bogatchev, 1960, by original designation. Status: valid genus in Pimeliinae: Pimeliini.

*Taenobates* Motschulsky, 1872: 25 [M]. Type species: *Tenebriosaperdoides* G.-A. Olivier, 1795, by original designation. Status: junior synonym of *Xylopinus* J.L. LeConte, 1862 in Stenochiinae: Cnodalonini. Synonymy: C.O. Waterhouse (1876: 288). Note: we act as First Revisers and reject the alternative original spelling *Taeniobates*, used by Motschulsky (1872: 32).

*Tagalinus* Kaszab, 1977a: 301, 333 [M]. Type species: *Ulomalifuanum* Montrouzier, 1860, by original designation. Status: valid genus in Phrenapatinae: Penetini.

*Tagalopsis* Kaszab, 1955a: 471, 475 [F]. Type species: *Tagalopsisszekessyi* Kaszab, 1955, by original designation. Status: valid genus in Phrenapatinae: Penetini.

*Tagalus* Gebien, 1914b: 388 [M]. Type species: *Tagalusimpressicollis* Gebien, 1914, by subsequent designation (Gebien 1940: 756). Status: junior synonym of *Dioedus* J.L. LeConte, 1862 in Phrenapatinae: Penetini. Synonymy: Kaszab (1977a: 314).

*Tagenesthes* Koch, 1941: 276, 282 [F]. Type species: *Asphaltesthesafrogermanica* Koch, 1941, by monotypy. Status: valid subgenus of *Asphaltesthes* Kraatz, 1865 in Pimeliinae: Tentyriini.

*Tagenia* Latreille, 1802: 170 [F]. Type species: *Tenebriofiliformis* Fabricius, 1792, by monotypy. Status: junior synonym of *Stenosis* Herbst, 1799 in Pimeliinae: Stenosini: Stenosina. Synonymy: G.-A. Olivier (1803: 355, through synonymy of the type species).

*Tagenodes* Haag-Rutenberg, 1876: 87 [M]. Type species: *Tagenodesmoufleti* Haag-Rutenberg, 1876, by monotypy. Status: junior synonym of *Rhammatodes* Haag-Rutenberg, 1876 in Pimeliinae: Tentyriini. Synonymy: Koch (1952a: 133).

†*Tagenopsis* Heer, 1864: 377 [F]. Type species: *Tagenopsisbrevicornis* Heer, 1864, by monotypy. Status: valid genus in Tenebrionidae: incertae sedis. Note: combined description of a new genus and a single new species (ICZN 1999, Article 12.2.6); described from Middle Miocene deposits (Germany).

*Tagenostola* Reitter, 1916d: 138, 151 [F]. Type species: *Stenosisturkestanica* Reitter, 1886, by subsequent designation (Gebien 1937a: 684). Status: valid genus in Pimeliinae: Stenosini: Stenosina.

*Tagona* Fischer, 1820: pl. 16 [F]. Type species: *Tagonaacuminata* Fischer, 1820, by subsequent designation (Hope 1841: 124). Status: valid genus in Blaptinae: Blaptini: Prosodina.

*Tagonoides* Fairmaire, 1886d: 343 [F]. Type species: *Tagonoidesdelavayi* Fairmaire, 1886, by subsequent designation (Blair 1923a: 283). Status: valid genus in Blaptinae: Blaptini: Gnaptorinina.

*Taichius* Ando, 1996: 196, 197 [M]. Type species: *Platycrepishemiceroides* Blair, 1929, by original designation. Status: valid genus in Stenochiinae: Cnodalonini.

*Taiwanocryphaeus* Masumoto, 1996b: 67 [M]. Type species: *Taiwanocryphaeusrhinoceros* Masumoto, 1996, by original designation. Status: valid genus in Tenebrioninae: Toxicini: Toxicina.

*Taiwanolagria* Masumoto, 1988b: 41 [F]. Type species: *Taiwanolagriamerkli* Masumoto, 1988, by original designation. Status: valid genus in Lagriinae: Lagriini: Statirina.

*Taiwanomenephilus* Masumoto, 1986b: 61 [M]. Type species: *Taiwanomenephiluschui* Masumoto, 1986, by original designation. Status: valid genus in Stenochiinae: Cnodalonini.

*Taiwanomenimus* Masumoto, Akita & Lee, 2019: 161 [M]. Type species: *Taiwanomenimusnakasatoi* Masumoto, Akita & Lee, 2019, by original designation. Status: valid genus in Diaperinae: Gnathidiini: Gnathidiina.

*Taiwanotagalus* Masumoto, 1982b: 143 [M]. Type species: *Taiwanotagalusklapperichi* Masumoto, 1982, by monotypy. Status: valid genus in Phrenapatinae: Penetini.

*Taiwanotrachyscelis* Masumoto, Akita & Lee, 2012: 29 [F]. Type species: *Taiwanotrachyscelischengi* Masumoto, Akita & Lee, 2012, by original designation. Status: valid genus in Diaperinae: Trachyscelini.

*Taklamakania* Ferrer & Yvinec, 2004: 42 [F]. Type species: *Taklamakanialepetzi* Ferrer & Yvinec, 2004, by monotypy. Status: junior synonym of *Paranemia* Heyden, 1892 in Diaperinae: Phaleriini. Synonymy: G.S. Medvedev (2006: 557). Note: junior homonym of *Taklamakania* Zhang, 1979 [Trilobita].

*Talanus* Jacquelin du Val, 1857: 156 [M]. Type species: *Talanuscribrarius* Jacquelin du Val, 1857, by monotypy. Status: valid genus in Stenochiinae: Talanini.

*Tamatasida* Koch, 1962a: 90, 145 [F]. Type species: *Scotinesthestuberculosa* Fairmaire, 1895, by original designation. Status: valid genus in Pimeliinae: Asidini.

*Tamdaous* Pic, 1923b: 11 [M]. Type species: *Tamdaousimpressus* Pic, 1923, by monotypy. Status: valid subgenus of *Oedemutes* Pascoe, 1860 in Stenochiinae: Cnodalonini.

*Tamena* Reitter, 1900c: 90, 143 [F]. Type species: *Psammocryptusrugiceps* Reitter, 1897, by monotypy. Status: valid genus in Pimeliinae: Tentyriini.

*Tanchirus* Fairmaire, 1897e: 224 [M]. Type species: *Tanchiruscompactus* Fairmaire, 1897, by monotypy. Status: valid genus in Stenochiinae: Cnodalonini.

*Tangiprosodes* G.S. Medvedev, 2005b: 84 [M]. Type species: *Prosodespygmaea* Kraatz, 1882, by original designation. Status: valid subgenus of *Prosodes* Eschscholtz, 1829 in Blaptinae: Blaptini: Prosodina. Note: originally described as a section within a subgenus.

*Tanychilus* Newman, 1838: 487 [M]. Type species: *Tanychilusstriatus* Newman, 1838, by subsequent designation (Cazurro Ruiz 1897a: 213). Status: valid genus in Alleculinae: Alleculini: Alleculina.

*Tanylypa* Pascoe, 1869: 152 [F]. Type species: *Tanylypamorio* Pascoe, 1869, by monotypy. Status: valid genus in Zolodininae.

*Tapenopsis* Solier, 1843: 5 [F]. Type species: *Tapenopsiscostata* Solier, 1843, by original designation. Status: valid genus in Pimeliinae: Leptodini.

*Taphrosoma* Kirsch, 1866: 195 [N]. Type species: *Taphrosomadohrnii* Kirsch, 1866, by monotypy. Status: valid genus in Stenochiinae: Cnodalonini.

*Tapinocomus* Gebien, 1928: 102 [M]. Type species: *Tapinocomussubnudus* Gebien, 1928, by monotypy. Status: valid genus in Pimeliinae: Epitragini.

*Tapinopsis* Agassiz, 1846b: 361 [F]. Type species [automatic]: *Tapenopsiscostata* Solier, 1843, by original designation. Status: junior synonym of *Tapenopsis* Solier, 1843 in Pimeliinae: Leptodini. Note: unjustified emendation of *Tapenopsis* Solier, 1843, not in prevailing usage.

*Taraxides* C.O. Waterhouse, 1876: 289 [M]. Type species: *Helopssinuatus* Fabricius, 1801 (= *Tenebriolaevigatus* Fabricius, 1781), by subsequent designation (Gebien 1941: 340). Status: valid genus in Stenochiinae: Cnodalonini. Note: the original combination of the accepted name of the type species, *Tenebriolaevigatus* Fabricius, 1781, is a junior primary homonym of *Tenebriolaevigatus* Linnaeus, 1767.

*Tarpela* Bates, 1870: 272 [F]. Type species: *Tarpelabrownii* Bates, 1870, by subsequent designation (Gebien 1943: 407). Status: valid genus in Tenebrioninae: Helopini: Helopina.

*Tarphiophasis* Wollaston, 1877: 227 [F]. Type species: *Tarphiophasistuberculata* Wollaston, 1877, by monotypy. Status: valid genus in Blaptinae: Opatrini: Ammobiina.

*Tarsocnodes* Gebien, 1920: 82 [M]. Type species: *Psammodesmolossus* Haag-Rutenberg, 1871, by original designation. Status: valid genus in Pimeliinae: Sepidiini: Molurina.

*Tarsosis* Gebien, 1920: 33, 34 [F]. Type species: *Stenocaradamarense* Péringuey, 1886, by monotypy. Status: valid subgenus of *Zophosis* Latreille, 1802 in Pimeliinae: Zophosini.

*Tauroceras* Hope, 1841: 130 [N]. Type species: *Tenebriocornutus* Fabricius, 1775, by original designation. Status: valid genus in Tenebrioninae: Centronopini.

*Tauroceropedus* Pic, 1913b: 4 [M]. Type species: *Tauroceropedusdifformipes* Pic, 1913, by subsequent designation (Gebien 1941: 344). Status: junior synonym of *Tauroceras* Hope, 1841 in Stenochiinae: Cnodalonini. Synonymy: Ferrer et al. (2005: 272).

*Taurohelops* Keskin & Nabozhenko, 2015: 84 [M]. Type species: *Stenomaxincultus* Allard, 1877, by original designation. Status: valid genus in Tenebrioninae: Helopini: Cylindrinotina.

*Taxes* Champion, 1895a: 226 [M]. Type species: *Taxesdepressus* Champion, 1895, by subsequent designation (R. Lucas 1920: 628). Status: valid genus in Alleculinae: Alleculini: Alleculina.

*Tearchus* Kraatz, 1880b: 110 [M]. Type species: *Tearchusannulipes* Kraatz, 1880, by monotypy. Status: valid genus in Stenochiinae: Cnodalonini.

*Tedinus* Casey, 1891: 153 [M]. Type species: *Tedinusangustus* Casey, 1891, by monotypy. Status: junior synonym of *Isomira* Mulsant, 1856 in Alleculinae: Alleculini: Gonoderina. Synonymy: Bousquet and Campbell (1991: 259).

*Telabis* Casey, 1890b: 331 [M]. Type species: *Eurymetoponlongipenne* Casey, 1890, by subsequent designation (Casey 1907: 288). Status: valid genus in Pimeliinae: Edrotini. Note: see Alonso-Zarazaga in Bousquet et al.(2018: 116) for comments on the gender of this genus-group name.

*Telacis* Poey, 1854: 322 [M]. Type species [automatic]: *Chrysomelasulphurea* Linnaeus, 1758, by subsequent designation (Latreille 1810: 429). Status: junior synonym of *Cteniopus* Solier, 1835 in Alleculinae: Cteniopodini. Note: replacement name for *Cistela* Fabricius, 1775, a senior synonym of *Cteniopus* Solier, 1835.

*Telaponium* Blaisdell, 1923: 209 [N]. Type species: *Telaponiumcastaneum* Blaisdell, 1923, by original designation. Status: valid genus in Pimeliinae: Edrotini.

*Telchis* Champion, 1886: 142 [M]. Type species: *Telchisclavicornis* Champion, 1886, by monotypy. Status: valid genus in Phrenapatinae: Penetini.

*Teles* Mulsant & Godart, 1876: 163 [M]. Type species: *Teleseutymi* Mulsant & Godart, 1876, by monotypy. Status: valid genus in Stenochiinae: Cnodalonini. Note: the alternative original spelling *Tales*, used by Mulsant and Godart (1876: 164), was rejected by Rye (1879: 62) who acted as the First Reviser.

*Telesicles* Champion, 1888: 450 [M]. Type species: *Telesiclescordatus* Champion, 1888, by monotypy. Status: valid genus in Alleculinae: Alleculini: Alleculina.

*Telethrus* Pascoe, 1882: 29 [M]. Type species: *Telethrusebeninus* Pascoe, 1882, by monotypy. Status: valid genus in Stenochiinae: Cnodalonini.

*Telleus* Fairmaire, 1904b: 465 [M]. Type species: *Telleuscrenatus* Fairmaire, 1904, by monotypy. Status: valid genus in Stenochiinae: Cnodalonini.

*Temnes* Champion, 1888: 410 [M]. Type species: *Temnescaeruleus* Champion, 1888, by monotypy. Status: valid genus in Alleculinae: Alleculini: Alleculina.

*Temnoaphelus* Ferrer, 1988: 377 [M]. Type species: *Temnoaphelushispidus* Ferrer, 1988, by original designation. Status: valid genus in Stenochiinae: Cnodalonini.

*Temnophthalmus* Gebien, 1921b: 62, 75 [M]. Type species: *Temnophthalmusscalaris* Gebien, 1921, by subsequent designation (Gebien 1941: 341). Status: valid genus in Stenochiinae: Cnodalonini.

*Teneatopus* Reitter, 1920a: 23 [M]. Type species: *Tenebrioatronitens* Fairmaire, 1891, by monotypy. Status: junior synonym of *Ariarathus* Fairmaire, 1891 in Tenebrioninae: Tenebrionini. Synonymy: Ardoin (1969b: 126).

*Tenebrio* Linnaeus, 1758: 417 [M]. Type species: *Tenebriomolitor* Linnaeus, 1758, by subsequent designation (Latreille 1810: 429). Status: valid genus and subgenus in Tenebrioninae: Tenebrionini.

*Tenebriocamaria* Pic, 1919b: 3 [F]. Type species: *Tenebriocamariaatra* Pic, 1919, by monotypy. Status: valid genus in Stenochiinae: Cnodalonini.

*Tenebriocephalon* Pic, 1925b: 10 [N]. Type species: *Tenebriocephalonpiceum* Pic, 1925, by monotypy. Status: valid genus in Pimeliinae: Ceratanisini.

*Tenebrioloma* Gebien, 1910c: 386 [N]. Type species: *Tenebriolomasemicostatum* Gebien, 1910, by monotypy. Status: junior synonym of *Tribolium* W.S. MacLeay, 1825 in Tenebrioninae: Triboliini. Synonymy: Halstead (1967b: 270).

*Tenebriomimus* Kolbe, 1901: 342 [M]. Type species: *Tenebriomimusadansoniarum* Kolbe, 1901 (= *Palembusocularis* Casey, 1891), by monotypy. Status: junior synonym of *Ulomoides* Blackburn, 1888 in Diaperinae: Diaperini: Diaperina. Synonymy: Gebien (1922b: 268, with *Martianus* Fairmaire, 1893, a junior synonym of *Ulomoides* Blackburn, 1888).

*Tenebrionellus* Crotch, 1874: 105 [M]. Type species [automatic]: *Tenebriomolitor* Linnaeus, 1758, by subsequent designation (Latreille 1810: 429). Status: junior synonym of *Tenebrio* Linnaeus, 1758 in Tenebrioninae: Tenebrionini. Note: unnecessary replacement name for *Tenebrio* Linnaeus, 1758.

†*Tenebrionites* Cockerell, 1920: 67 [M]. Type species: *Tenebrionitesanglicus* Cockerell, 1920, by monotypy. Status: valid genus in Tenebrionidae: incertae sedis. Note: combined description of a new genus and a single new species (ICZN 1999, Article 12.2.6); described from Middle Eocene deposits (United Kingdom).

*Tenebriopsis* Gebien, 1928: 170, 186 [F]. Type species: *Tenebriopsissubtilicostis* Gebien, 1928, by monotypy. Status: valid genus in Stenochiinae: Cnodalonini.

*Tenesis* Duvivier, 1892: 163 [M]. Type species: *Tenesisfemoratus* Duvivier, 1892, by monotypy. Status: valid genus in Stenochiinae: Cnodalonini.

*Tentyria* Latreille, 1802: 170 [F]. Type species: *Tentyrialigurica* Solier, 1835, by plenary powers (ICZN 2010a, Opinion 2244). Status: valid genus in Pimeliinae: Tentyriini. Note: name placed on the Official List of Generic Names in Zoology (ICZN 2010a, Opinion 2244).

*Tentyriina* Peyerimhoff, 1907: 31 [F]. Type species [automatic]: *Pimeliaorbiculata* Fabricius, 1775, by subsequent designation (Gebien 1937a: 624; see ICZN 2010a, Opinion 2244). Status: junior synonym of *Tentyrina* Reitter, 1900 in Pimeliinae: Tentyriini. Note: unjustified emendation of *Tentyrina* Reitter, 1900, not in prevailing usage.

*Tentyrina* Reitter, 1900c: 92, 166 [F]. Type species: *Pimeliaorbiculata* Fabricius, 1775, by subsequent designation (Gebien 1937a: 624; see ICZN 2010a, Opinion 2244). Status: valid genus in Pimeliinae: Tentyriini. Note: name placed on the Official List of Generic Names in Zoology (ICZN 2010a, Opinion 2244).

*Tentyriomorpha* Peyerimhoff, 1927: 52 [F]. Type species [automatic]: *Tentyromorphatelueti* Escalera, 1913, by monotypy. Status: junior synonym of *Tentyromorpha* Escalera, 1913 in Pimeliinae: Tentyriini. Note: unjustified emendation of *Tentyromorpha* Escalera, 1913, not in prevailing usage.

*Tentyriopsis* Gebien, 1928: 168, 174 [F]. Type species: **fixed herein** (ICZN 1999, Article 70.3) as *Tentyriopsispertyi* Gebien, 1940, misidentified as *Tentyriastriipuncta* Perty, 1830 in the original designation by monotypy in Gebien (1928). Status: valid genus in Stenochiinae: Cnodalonini. Note: the type species *Tentyriastriipuncta* Perty was first established by monotypy; Gebien (1940: 1091) reported that *Tentyriastriipuncta* Perty of Gebien (1928) was misidentified and proposed the name *Tentyriopsispertyi* for it; we follow currently accepted concepts (e.g., Blackwelder 1945: 533) and fix the type species according to the requirements of Article 70.3.2 (ICZN 1999); the nominal species *Tentyriastriipuncta* Perty, 1830 is also a valid species in the genus *Tentyriopsis* Gebien, 1928.

*Tentyrodera* Koch, 1943a: 572 [F]. Type species: *Microderamarginata* Baudi di Selve, 1874, by monotypy. Status: valid subgenus of *Microdera* Eschscholtz, 1831 in Pimeliinae: Tentyriini.

*Tentyromorpha* Escalera, 1913: 38 [F]. Type species: *Tentyromorphatelueti* Escalera, 1913, by monotypy. Status: valid subgenus of *Pachychila* Eschscholtz, 1831 in Pimeliinae: Tentyriini.

*Tentyronota* Reitter, 1900c: 93, 188 [F]. Type species: *Micipsarotundicollis* Kraatz, 1865, by monotypy. Status: valid genus in Pimeliinae: Tentyriini.

*Terametus* Motschulsky, 1869: 193 [M]. Type species: *Terametuscapicola* Motschulsky, 1869, by monotypy. Status: valid genus in Lagriinae: Lupropini.

*Teremenes* Carter, 1914b: 54 [M]. Type species: *Tenebriolongipennis* Hope, 1843, by **present designation**. Status: junior synonym of *Zophophilus* Fairmaire, 1881 in Stenochiinae: Cnodalonini. Synonymy: Carter (1930: 547).

*Termitonebria* Wasmann in Wasmann and Brauns, 1925: 105 [F]. Type species: *Termitonebriabraunsi* Wasmann, 1925 (= *Asyleptusfumosus* Péringuey, 1896), by monotypy. Status: junior synonym of *Asyleptus* Péringuey, 1896 in Tenebrioninae: Amarygmini. Synonymy: Schawaller and Bremer (2013: 81).

*Tessaromma* Boheman, 1858: 91 [N]. Type species: *Tessarommalugubre* Boheman, 1858, by subsequent designation (Aalbu and Triplehorn 1991: 170). Status: junior synonym of *Blapstinus* Dejean, 1821 in Blaptinae: Opatrini: Blapstinina. Synonymy: Gemminger in Gemminger and Harold (1870: 1923, with *Pedonoeces* G.R. Waterhouse, 1845, a junior synonym of *Blapstinus* Dejean, 1821), Aalbu and Triplehorn (1991: 170). Note: junior homonym of *Tessaromma* Newman, 1840 [Coleoptera: Cerambycidae].

*Tetragonomecus* Rye, 1880: 87 [M]. Type species [automatic]: *Tetragonomenessemiviridis* Chevrolat, 1878, by monotypy. Status: junior synonym of *Tetragonomenes* Chevrolat, 1878 in Stenochiinae: Cnodalonini. Note: unjustified emendation for *Tetragonomenes* Chevrolat, 1878, not in prevailing usage.

*Tetragonomenes* Chevrolat, 1878b: clii [M]. Type species: *Tetragonomenessemiviridis* Chevrolat, 1878, by monotypy. Status: valid genus in Stenochiinae: Cnodalonini.

*Tetranillus* Wasmann, 1899a: 167 [M]. Type species: *Tetranilluscostatus* Wasmann, 1899, by monotypy. Status: valid genus in Pimeliinae: Stenosini: Stenosina.

*Tetranosis* G.S. Medvedev, 1995a: 858 [F]. Type species: *Tetranosisclypeoloba* Koch, 1940, by original designation. Status: valid subgenus of *Microtelopsis* Koch, 1940 in Pimeliinae: Stenosini: Stenosina. Note: as pointed out by Aalbu et al. (2017: 326) this name was first introduced by Koch (1940b: 740); however, Koch did not designate a type species and therefore his name is unavailable; Löbl and Merkl (2003: 251) designated a type species for this genus but did not describe the genus-group taxon as new (ICZN 1999, Article 16.1); G.S. Medvedev (1995a: 858) was the first to provide a description of *Tetranosis* as well as fixing a type species for the genus, thereby making the name nomenclaturally available for the first time; we hereby act as First Revisers and select *Microtelopsis* Koch, 1940 as the valid name of the genus based on the Principle of Priority (ICZN 1999, Article 23.3.5).

*Tetraphyllus* Laporte & Brullé, 1831: 332, 404 [M]. Type species: *Tetraphylluslatreillei* Laporte & Brullé, 1831, by subsequent designation (Gebien 1935: 64). Status: valid genus in Stenochiinae: Cnodalonini. Note: nomenclatural stability in this genus is threatened by the discovery of an older type species designation (*Tetraphyllusformosus* Laporte & Brullé, 1831, by subsequent designation by Bates (1879a: 293), which is the type species of *Damatris* Laporte, 1840); we recommend that an application be submitted to the International Commission on Zoological Nomenclature to maintain the type species designation proposed by Gebien (1935: 64).

*Tetrethas* Koch, 1962a: 23, 145 [M]. Type species: *Anethasxylophilus* Koch, 1962, by original designation. Status: valid subgenus of *Anethas* Jakobson, 1924 in Pimeliinae: Stenosini: Stenosina.

*Tetromma* Dejean, 1834: 183 [N]. Type species: *Upisunicolor* Herbst, 1797, by subsequent designation (Löbl et al. 2008a: 43). Status: junior synonym of *Hyperops* Eschscholtz, 1831 in Pimeliinae: Tentyriini. Synonymy: Dejean (1836: 203).

*Texaponium* Thomas, 1984: 658 [N]. Type species: *Cryptadiustriplehorni* Berry, 1974, by original designation. Status: valid genus in Pimeliinae: Edrotini.

*Thaioblaps* Masumoto, 1989: 187 [F]. Type species: *Thaioblapspunneeae* Masumoto, 1989, by original designation. Status: valid genus in Blaptinae: Blaptini: Blaptina.

*Thalpobia* Fairmaire, 1871a: 385 [F]. Type species: *Thalpobialaevipennis* Fairmaire, 1871, by monotypy. Status: valid genus in Pimeliinae: Tentyriini.

*Thalpophila* Solier, 1835b: 253, 370 [F]. Type species: *Akisabbreviata* Fabricius, 1801, by subsequent designation (Chevrolat 1849: 544). Status: senior synonym of *Thalpophilodes* Strand, 1942 in Pimeliinae: Tentyriini. Note: junior homonym of *Thalpophila* Hübner, 1820 [Lepidoptera].

*Thalpophilodes* Strand, 1942: 391 [M]. Type species [automatic]: *Akisabbreviata* Fabricius, 1801, by subsequent designation (Chevrolat 1849: 544). Status: valid genus in Pimeliinae: Tentyriini. Note: replacement name for *Thalpophila* Solier, 1835.

*Tharsus* J.L. LeConte, 1862: 233 [M]. Type species: *Tharsusseditiosus* J.L. LeConte, 1862, by monotypy. Status: junior synonym of *Metaclisa* Jacquelin du Val, 1861 in Tenebrioninae: Metaclisini. Synonymy: Steiner (2016: 538).

*Thaumatoblaps* Kaszab & G.S. Medvedev, 1984: 79 [F]. Type species: *Thaumatoblapsmarikovskiji* Kaszab & G.S. Medvedev, 1984, by original designation. Status: valid genus in Blaptinae: Blaptini: Blaptina.

*Theatetes* Champion, 1888: 420 [M]. Type species: *Theatetesbasicornis* Champion, 1888, by monotypy. Status: valid genus in Alleculinae: Alleculini: Alleculina.

*Thecacerus* Lacordaire, 1859b: 420 [M]. Type species: *Cnodalonnodosum* Gray, 1831, by monotypy. Status: valid genus in Stenochiinae: Cnodalonini.

*Theresea* Pic, 1917d: 12 [F]. Type species: *Thereseadiversipennis* Pic, 1917, by monotypy. Status: valid genus in Stenochiinae: Stenochiini. Note: Masumoto (1999b: 130) transferred this genus from Cnodalonini.

*Thesilea* Haag-Rutenberg, 1878: 103 [F]. Type species: *Thesileaversicolor* Haag-Rutenberg, 1878, by subsequent designation (R. Lucas 1920: 638). Status: valid genus in Stenochiinae: Cnodalonini.

*Thettea* Bates, 1879a: 290 [F]. Type species: *Thetteatenuitarsis* Bates, 1879, by monotypy. Status: valid genus in Stenochiinae: Cnodalonini.

*Thinobatis* Eschscholtz, 1831: 5, 8 [F]. Type species: *Thinobatisferruginea* Eschscholtz, 1831, by monotypy. Status: valid genus in Pimeliinae: Thinobatini.

*Thoracon* Gistel, 1848a: xi [N]. Type species [automatic]: *Silphasabulosa* Linnaeus, 1758, by subsequent designation (Latreille 1810: 429). Status: junior synonym of *Opatrum* Fabricius, 1775 in Blaptinae: Opatrini: Opatrina. Note: unnecessary replacement name for *Opatrum* Fabricius, 1775.

*Thoracophorus* Hope, 1841: 188 [M]. Type species: *Thoracophoruswalckenaerii* Hope, 1841, by original designation. Status: senior synonym of *Cardiothorax* Motschulsky, 1860 in Lagriinae: Adeliini. Synonymy: Motschulsky (1860a: 67). Note: junior homonym of *Thoracophorus* Motschulsky, 1837 [Coleoptera: Staphylinidae].

*Thoracostira* Borchmann, 1936: 240, 508 [F]. Type species: *Statirasculpta* Kirsch, 1873, by original designation. Status: valid genus in Lagriinae: Lagriini: Statirina.

*Thorictophasis* Koch, 1950c: 80 [F]. Type species: *Caenocrypticusdeserticus* Koch, 1950, by original designation. Status: junior synonym of *Caenocrypticus* Gebien, 1920 in Pimeliinae: Caenocrypticini. Synonymy: Endrödy-Younga (1996: 13).

*Thorictosoma* Lea, 1919: 257 [N]. Type species: *Thorictosomaectatommae* Lea, 1919, by original designation. Status: valid genus in Pimeliinae: Cnemeplatiini: Thorictosomatina.

*Thornella* Novák, 2019e: 83 [F]. Type species: *Alleculaholomelaena* Fairmaire, 1894, by original designation. Status: valid subgenus of *Upinella* Mulsant, 1856 in Alleculinae: Alleculini: Alleculina.

*Thoseus* Pic, 1925b: 9 [M]. Type species: *Thoseusrufus* Pic, 1925, by monotypy. Status: valid genus in Lagriinae: Belopini.

*Thraustocolus* Kraatz, 1866: 414 [M]. Type species [automatic]: *Calobamonleptoderus* Kraatz, 1865, by monotypy. Status: valid genus & subgenus in Pimeliinae: Tentyriini. Note: replacement name for *Calobamon* Kraatz, 1865.

*Threnus* Motschulsky, 1870: 404 [M]. Type species: *Threnusniger* Motschulsky, 1870, by original designation. Status: senior synonym of *Argoporis* Horn, 1870 in Tenebrioninae: Cerenopini. Synonymy: Aalbu et al. (1995: 483). Note: see the entry for *Argoporis* Horn, 1870 regarding the priority of these two names.

*Thriptera* Solier, 1836: 9, 48 [F]. Type species: *Thripteramaillei* Solier, 1836 (= *Pimeliacrinita* Klug, 1830), by subsequent designation (Hope 1841: 118). Status: valid genus in Pimeliinae: Pimeliini.

*Thurea* Ferrer, 1998b: 151 [F]. Type species: *Thureapalmi* Ferrer, 1998, by original designation. Status: junior synonym of *Platycotylus* Olliff, 1883 in Tenebrioninae: Palorini. Synonymy: Schawaller (2014: 51).

*Thydemorphus* Pic, 1918b: 19 [M]. Type species: *Thydemorphuspilitarsis* Pic, 1918, by monotypy. Status: valid genus in Stenochiinae: Cnodalonini.

*Thydemus* Lewis, 1894: 475 [M]. Type species: *Scotaeuspurpurivittatus* Marseul, 1876, by original designation. Status: junior synonym of *Pseudonautes* Fairmaire, 1892 in Stenochiinae: Cnodalonini. Synonymy: Gebien (1914a: 58).

*Thylacoderes* Solier, 1843: 44, 123 [M]. Type species: *Thylacodereseumolpoides* Solier, 1843, by original designation. Status: valid genus in Pimeliinae: Praociini.

*Tibinella* Novák, 2019e: 91 [F]. Type species: *Upinellapahangica* Novák, 2019, by original designation. Status: valid subgenus of *Upinella* Mulsant, 1856 in Alleculinae: Alleculini: Alleculina.

​*Tibiocnodes* ​Gearner & Kamiński in Gearner et al., 2021: 8 [M]. Type species: *Psammodeslucidus* ​Fåhraeus, 1870, by original designation. Status: valid genus in Pimeliinae: Sepidiini: Molurina.

*Tidiguinia* Español, 1959: 249 [F]. Type species: *Tidiguiniabolivari* Español, 1959, by original designation. Status: valid genus in Blaptinae: Opatrini: Opatrina.

*Timeneca* Carter, 1914b: 78 [F]. Type species [automatic]: *Ctimenebreweri* Bates, 1873, by monotypy. Status: junior synonym of *Mitrothorax* Carter, 1914 in Tenebrioninae: Heleini: Cyphaleina. Synonymy: Matthews (1992: 456). Note: replacement name for *Ctimene* Bates, 1873, a senior synonym of *Mitrothorax* Carter, 1914.

*Timogebienus* Ardoin, 1963b: 308, 331 [M]. Type species: *Hoplonyxcollaris* Gebien, 1910, by monotypy. Status: valid genus in Tenebrioninae: Amarygmini.

*Timosmithus* Ardoin, 1974b: 457 [M]. Type species: *Timosmithusbasilewskyi* Ardoin, 1974, by monotypy. Status: valid genus in Pimeliinae: Stenosini: Stenosina.

*Tinophyllus* Fairmaire, 1869b: 234 [M]. Type species: *Tinophyllusgracilicornis* Fairmaire, 1869, by monotypy. Status: junior synonym of *Camariodes* Fairmaire, 1869 in Stenochiinae: Cnodalonini. Synonymy: Fairmaire (1886c: 75).

*Tisamenes* Champion, 1884: 64 [M]. Type species: *Tisamenestruquii* Champion, 1884, by monotypy. Status: valid subgenus of *Philolithus* Lacordaire, 1858 in Pimeliinae: Asidini.

*Titaena* Erichson, 1842a: 178 [F]. Type species: *Titaenacolumbina* Erichson, 1842, by subsequent designation (Chevrolat 1849: 594). Status: valid genus in Tenebrioninae: Titaenini.

*Tithassa* Pascoe, 1860b: 125 [F]. Type species: *Tithassacorynomelas* Pascoe, 1860, by monotypy. Status: valid genus in Lagriinae: Goniaderini.

*Tjikoraia* Pic, 1921d: 18 [F]. Type species: *Tjikoraiajavana* Pic, 1921, by subsequent designation (Riley 1923: 130). Status: junior synonym of *Menimus* Sharp, 1876 in Diaperinae: Gnathidiini: Gnathidiina. Synonymy: Kaszab (1979a: 81).

*Tlascalinus* Casey, 1907: 370 [M]. Type species: *Trimytisflohri* Champion, 1892, by monotypy. Status: valid genus in Pimeliinae: Edrotini.

*Toktokkus* Kamiński & Gearner in Kamiński et al., 2020: 10 [M]. Type species: *Toktokkustschinkeli* Kamiński & Gearner, 2020, by original designation. Status: valid genus in Pimeliinae: Sepidiini: Molurina.

*Tomogria* Merkl, 1988a: 135 [F]. Type species: *Tomogriaperlata* Merkl, 1988, by original designation. Status: valid genus in Lagriinae: Lagriini: Lagriina.

*Tonibiastes* Casey, 1895: 617 [M]. Type species: *Notibiuscostipennis* Horn, 1894, by original designation. Status: valid genus in Blaptinae: Opatrini: Blapstinina.

*Tonibius* Casey, 1895: 617 [M]. Type species: *Notibiussulcatus* J.L. LeConte, 1851, by subsequent designation (R. Lucas 1920: 644). Status: valid genus in Blaptinae: Opatrini: Blapstinina.

*Tonkinius* Fairmaire, 1903a: 13 [M]. Type species: *Tonkiniussculptilis* Fairmaire, 1903, by monotypy. Status: valid genus in Stenochiinae: Cnodalonini.

*Toreuma* Carter, 1913a: 84 [N]. Type species: *Toreumacupreum* Carter, 1913, by monotypy. Status: senior synonym of *Atoreuma* Gebien, 1941 in Tenebrioninae: Heleini: Cyphaleina. Note: junior homonym of *Toreuma* Haeckel, 1880 [Cnidaria].

*Toxicum* Latreille, 1802: 174 [N]. Type species: *Toxicumrichesianum* Latreille, 1802, by monotypy. Status: valid genus and subgenus in Tenebrioninae: Toxicini: Toxicina. Note: combined description of a new genus and a single new species (ICZN 1999, Article 12.2.6).

*Toxocnema* Fåhraeus, 1870: 304 [F]. Type species: *Toxocnemarufitarsis* Fåhraeus, 1870, by monotypy. Status: valid genus in Stenochiinae: Cnodalonini. Note: transferred from Tenebrioninae: Ulomini by Schawaller (2009b: 363).

*Trachasida* Reitter, 1917a: 41, 62 [F]. Type species: *Asidaruficornis* Solier, 1836, by subsequent designation (F. Soldati 2008: 33). Status: junior synonym of *Gracilasida* Escalera, 1905 in Pimeliinae: Asidini. Synonymy: Wilke (1922: 259, with *Planasida* Escalera, 1907, a junior synonym of *Gracilasida* Escalera, 1905).

*Tracheloeum* Hope, 1841: 116 [N]. Type species: *Tracheloeumlaticolle* Hope, 1841, by original designation. Status: valid subgenus of *Somaticus* Hope, 1841 in Pimeliinae: Sepidiini: Trachynotina. Note: combined description of a new genus and a single new species (ICZN 1999, Article 12.2.6).

*Trachelostenus* Solier, 1851: 255 [M]. Type species: *Trachelostenusinaequalis* Solier, 1851, by monotypy. Status: valid genus in Tenebrioninae: Trachelostenini. Note: the family-group name based on this genus was downgraded from the family Trachelostenidae (within the superfamily Tenebrionoidea) to the tribe Trachelostenini (within the tenebrionid subfamily Tenebrioninae) by Matthews and Lawrence (2015: 290).

*Trachyderes* Koch, 1955a: 112 [M]. Type species: *Trachynotusbipunctatus* Haag-Rutenberg, 1873, by original designation. Status: valid subgenus of *Somaticus* Hope, 1841 in Pimeliinae: Sepidiini: Trachynotina.

*Trachyderma* Latreille, 1828: 576 [N]. Type species: *Pimeliahispida* Fabricius, 1775 (= *Tenebriohispidus* Forskål, 1775), by subsequent designation (P.H. Lucas 1839: 50). Status: valid genus and subgenus in Pimeliinae: Pimeliini. Note: gender of genus corrected from feminine to neuter by Löbl and Smetana (2011: 33).

*Trachydermum* Gistel, 1848a: xi [N]. Type species [automatic]: *Pimeliahispida* Fabricius, 1775 (= *Tenebriohispidus* Forskål, 1775), by subsequent designation (P.H. Lucas 1839: 50). Status: junior synonym of *Trachyderma* Latreille, 1828 in Pimeliinae: Pimeliini. Note: unnecessary replacement name for *Trachyderma* Latreille, 1828.

*Trachymetus* Reitter, 1904: 51, 76 [M]. Type species: *Pachypteruselongatus* Mulsant & Rey, 1859, by monotypy. Status: junior synonym of *Amblysphagus* Fairmaire, 1896 in Blaptinae: Opatrini: Neopachypterina. Synonymy: Kaszab (1975a: 27).

*Trachynotidus* Péringuey, 1899: 296 [M]. Type species: *Psammodesthoreyi* Haag-Rutenberg, 1871, by subsequent designation (Kamiński et al. 2019b: 18). Status: valid genus in Pimeliinae: Sepidiini: Hypomelina.

*Trachynotus* Latreille, 1828: 579 [M]. Type species: *Sepidium vittatum* Fabricius, 1781, by subsequent designation (Kamiński et al. 2019b: 93). Status: valid genus in Pimeliinae: Sepidiini: Trachynotina.

*Trachyscelis* Latreille, 1809: 379 [F]. Type species: *Trachyscelisaphodioides* Latreille, 1809, by monotypy. Status: valid genus in Diaperinae: Trachyscelini.

*Tragardhus* Koch, 1956a: 369 [M]. Type species: *Tragardhusgrandipleurum* Koch, 1956, by original designation. Status: valid genus and subgenus in Blaptinae: Dendarini: Melambiina.

*Trestonia* Rafinesque, 1815: 113 [F]. Type species [automatic]: *Toxicumrichesianum* Latreille, 1802, by monotypy. Status: junior synonym of *Toxicum* Latreille, 1802 in Tenebrioninae: Toxicini: Toxicina. Note: unnecessary replacement name for *Toxicum* Latreille, 1802.

*Triangulipenna* Louw, 1979: 100, 114 [F]. Type species: *Triangulipennalacuna* Louw, 1979, by original designation. Status: valid genus in Pimeliinae: Sepidiini: Hypomelina.

*Tribolium* W.S. MacLeay, 1825: 47 [N]. Type species: *Colydiumcastaneum* Herbst, 1797, by monotypy (see ICZN 1988, Opinion 1495). Status: valid genus and subgenus in Tenebrioninae: Triboliini. Note: placed on the Official List of Generic Names in Zoology (ICZN 1988, Opinion 1495).

*Trichamarygmus* Carter, 1913b: 46 [M]. Type species: *Trichamarygmuspilosus* Carter, 1913, by monotypy. Status: valid genus in Tenebrioninae: Amarygmini.

*Trichanemia* Ardoin, 1971: 361, 406 [F]. Type species: *Anemiaschmitzi* Ardoin, 1971, by monotypy. Status: valid subgenus of *Cheirodes* Gené, 1839 in Tenebrioninae: Melanimonini.

*Tricheleodes* Blaisdell, 1909: 138 [M]. Type species: *Eleodeshirsuta* J.L. LeConte, 1861, by subsequent designation (Johnston 2016: 666). Status: valid subgenus of *Eleodes* Eschscholtz, 1829 in Blaptinae: Amphidorini.

*Trichethmus* Gebien, 1937b: 45 [M]. Type species: *Trichethmuslobicollis* Gebien, 1937, by monotypy. Status: valid genus in Pimeliinae: Sepidiini: Trachynotina.

*Trichiasida* Casey, 1912: 77, 172 [F]. Type species: *Pelecyphorushirsutus* J.L. LeConte, 1851, by original designation. Status: valid subgenus of *Stenomorpha* Solier, 1836 in Pimeliinae: Asidini.

*Trichiotes* Casey, 1907: 432, 443 [M]. Type species: *Trichiotesseriatus* Casey, 1907, by original designation. Status: valid genus in Pimeliinae: Edrotini.

*Trichochianalus* Kaszab, 1940a: 149, 201 [M]. Type species: *Platynoscelismonticola* Kaszab, 1940, by original designation. Status: valid subgenus of *Bioramix* Bates, 1879 in Blaptinae: Platyscelidini. Note: the spellings *Trichochianalis* (p. 201 – also used in the directory of new taxa published in volume 30 of the journal *Mitteilungen der Münchner Entomologischen Gesellschaft* (p. xi)) and *Trichochianalus* (pp 125, 149, 201) were used in the original publication; Kaszab (1940b: 989) used the spelling *Trichochianalus* only and therefore acted as the First Reviser (ICZN 1999, Article 24.2.4).

*Trichodamatris* Chatanay, 1915a: 506, 524 [M]. Type species: *Porphyrhybaraffrayi* Fairmaire, 1884, by original designation. Status: valid genus in Stenochiinae: Cnodalonini.

*Trichoderulus* Blaisdell, 1923: 281 [M]. Type species: *Trichoderuluslongipilosus* Blaisdell, 1923 (= *Eleodestribulus* Thomas, 2005), by original designation. Status: junior synonym of *Pseudeleodes* Blaisdell, 1909 in Blaptinae: Amphidorini. Synonymy: Johnston (2016: 672).

*Tricholeipopleura* Kaszab, 1940a: 152, 223 [F]. Type species: *Platynoscelislucidicollis* Kraatz, 1882, by original designation. Status: valid subgenus of *Bioramix* Bates, 1879 in Blaptinae: Platyscelidini. Note: the spellings *Tricholeipopleura* (pp 131, 223, 225–228, also used in the directory of new taxa published in volume 30 of the journal *Mitteilungen der Münchner Entomologischen Gesellschaft* (p. xii)) and *Tricholeipoleura* (pp. 152) were used in the original publication; Kaszab (1940b: 989) used the spelling *Tricholeipopleura* only and therefore acted as the First Reviser (ICZN 1999, Article 24.2.4).

*Trichomyatis* Schuster in Reinig, 1931: 893 [F]. Type species: *Trichomyatisconradti* Schuster, 1931, by original designation. Status: valid genus in Blaptinae: Platyscelidini.

*Trichoodescelis* Kaszab, 1940b: 940, 954 [F]. Type species: *Platyscelisacutangula* Kraatz, 1884, by original designation. Status: junior synonym of *Longuloodescelis* Kaszab, 1940 in Blaptinae: Platyscelidini. Synonymy: Egorov (2004: 595).

*Trichoplatynoscelis* Kaszab, 1940b: 896 [F]. Type species: *Trichoplatynoscelispamirensis* Kaszab, 1940, by original designation. Status: junior synonym of *Trichomyatis* Schuster, 1931 in Blaptinae: Platyscelidini. Synonymy: Egorov (2004: 603). Note: this name was also used in several instances by Kaszab (1940a: 126, 132, 142, 143, 146); however, it is not available from that publication since there is no type species designated.

*Trichoplatyscelis* Reinig, 1931: 895 [F]. Type species: *Trichoplatyscelispamirensis* Reinig, 1931, by original designation. Status: valid subgenus of *Bioramix* Bates, 1879 in Blaptinae: Platyscelidini.

*Trichopodus* Mulsant & Rey, 1859c: 58, 59 [M]. Type species: *Trichopodusvalidus* Mulsant & Rey, 1859, by monotypy. Status: senior synonym of *Trichosternum* Wollaston, 1861 in Blaptinae: Opatrini: Opatrina. Synonymy: Bates (1872a: 98). Note: junior homonym of *Trichopodus* Lacepède, 1802 [Pisces].

*Trichosaragus* Blackburn, 1890: 1269 [M]. Type species: *Trichosaraguspilosellus* Blackburn, 1890, by monotypy. Status: valid genus in Tenebrioninae: Heleini: Heleina.

*Trichosphaena* Reitter, 1916c: 140, 145 [F]. Type species: *Sphenariaolgae* Semenov, 1889, by subsequent designation (Gebien 1937a: 577). Status: valid genus in Pimeliinae: Tentyriini.

*Trichosternum* Wollaston, 1861: 205 [N]. Type species: *Trichosternumstriatum* Wollaston, 1861, by monotypy. Status: valid genus in Blaptinae: Opatrini: Opatrina.

*Trichostethe* Koch, 1950b: 285 [F]. Type species: *Cyphostethepilosa* Koch, 1950, by original designation. Status: valid subgenus of *Cyphostethe* Marseul, 1866 in Pimeliinae: Tentyriini.

*Trichotenebrio* Ardoin, 1962a: 64 [M]. Type species: *Trichotenebrioatronitidus* Ardoin, 1962, by monotypy. Status: valid genus in Tenebrioninae: Tenebrionini.

*Trichotoides* Marcuzzi, 1954: 23 [M]. Type species: *Scapteshintoni* Kaszab, 1949, by monotypy. Status: junior synonym of *Ammodonus* Mulsant, 1859 in Blaptinae: Opatrini: Ammobiina. Synonymy: Ferrer and Moraguès (2001: 499).

*Trichoton* Hope, 1841: 111 [N]. Type species: *Trichotoncayennense* Hope, 1841, by original designation. Status: valid genus in Blaptinae: Opatrini: Blapstinina. Note: combined description of a new genus and a single new species (ICZN 1999, Article 12.2.6).

*Trichotrachys* Koch, 1955a: 201 [M]. Type species: *Trachynotussordidus* Gerstaecker, 1854, by original designation. Status: valid subgenus of *Somaticus* Hope, 1841 in Pimeliinae: Sepidiini: Trachynotina.

*Trichotrichus* Koch, 1955a: 108 [M]. Type species: *Trachynotuscrinitus* Haag-Rutenberg, 1873, by original designation. Status: valid subgenus of *Somaticus* Hope, 1841 in Pimeliinae: Sepidiini: Trachynotina.

*Trichotum* Agassiz, 1846b: 376 [N]. Type species [automatic]: *Trichotoncayennense* Hope, 1841, by original designation. Status: junior synonym of *Trichoton* Hope, 1841 in Blaptinae: Opatrini: Blapstinina. Note: unjustified emendation of *Trichoton* Hope, 1841, not in prevailing usage.

*Trichulodes* Carter, 1914a: 223 [M]. Type species: *Trichulodespunctatus* Carter, 1914, by monotypy. Status: junior synonym of *Pseudolyprops* Fairmaire, 1882 in Lagriinae: Goniaderini. Synonymy: Doyen et al. (1990: 231).

*Trientoma* Solier, 1835b: 253, 256 [F]. Type species: *Trientomavarvasi* Solier, 1835, by monotypy. Status: valid genus in Pimeliinae: Edrotini.

*Trigonocnera* Reitter, 1893: 202, 213 [F]. Type species: *Trigonoscelispseudopimelia* Reitter, 1889, by original designation. Status: valid genus in Pimeliinae: Pimeliini.

*Trigonopachys* Skopin, 1968b: 300 [M]. Type species: *Trigonopachysmichailovi* Skopin, 1968, by original designation. Status: valid genus in Pimeliinae: Pimeliini.

*Trigonopilus* Fairmaire, 1893b: 22 [M]. Type species: *Trigonopiluslaticeps* Fairmaire, 1893, by monotypy. Status: valid genus in Blaptinae: Opatrini: incertae sedis. Note: placed in Opatrini incertae sedis by Kamiński et al. (2021b: 151).

*Trigonopoda* Gebien, 1914a: 2 [F]. Type species: *Trigonopodacrassipes* Gebien, 1914, by monotypy. Status: valid genus in Blaptinae: Opatrini: Ammobiina.

*Trigonopus* Mulsant & Rey, 1853b: 105 [M]. Type species: *Trigonopuscapicola* Mulsant & Rey, 1853, by subsequent designation (Cazurro Ruiz 1897b: 539). Status: valid genus in Blaptinae: Platynotini: Platynotina.

*Trigonoscelis* Dejean, 1834: 179 [F]. Type species: *Pimelianodosa* Fischer, 1820, by subsequent designation (Hope 1841: 118). Status: valid genus and subgenus in Pimeliinae: Pimeliini.

*Trigonotarsus* Hope, 1843: 357 [M]. Type species: *Trigonotarsusaustralis* Hope, 1843, by original designation. Status: senior synonym of *Sobas* Pascoe, 1863 in Blaptinae: Opatrini: Opatrina. Note: junior homonym of *Trigonotarsus* Guérin-Méneville, 1838 [Coleoptera: Curculionidae].

*Trilobocara* Solier, 1851: 129 [N]. Type species: *Trilobocaraciliatum* Solier, 1851, by original designation. Status: valid genus in Pimeliinae: Trilobocarini.

*Trimytantron* Ardoin, 1977: 381 [N]. Type species: *Trimytantrondecui* Ardoin, 1977, by original designation. Status: valid genus in Pimeliinae: Edrotini.

*Trimytis* J.L. LeConte, 1851: 141 [F]. Type species: *Trimytispruinosa* J.L. LeConte, 1851, by monotypy. Status: valid genus in Pimeliinae: Edrotini.

*Triorophus* J.L. LeConte, 1851: 141 [M]. Type species: *Triorophuslaevis* J.L. LeConte, 1851, by subsequent designation (Casey 1907: 432). Status: valid genus in Pimeliinae: Edrotini.

*Triphalopsis* Blaisdell, 1923: 232 [F]. Type species: *Triphalopsispartida* Blaisdell, 1923, by original designation. Status: valid genus in Pimeliinae: Edrotini.

*Triphalopsoides* Doyen, 1990: 222 [M]. Type species: *Triphalopsoideslasiodorsa* Doyen, 1990, by monotypy. Status: valid genus in Pimeliinae: Edrotini.

*Triphalus* J.L. LeConte, 1866b: 104 [M]. Type species: *Triphalusperforatus* J.L. LeConte, 1866, by monotypy. Status: valid genus in Pimeliinae: Edrotini.

*Triplehornia* Matthews & Lawrence, 2005: 543 [F]. Type species: *Triplehorniametallica* Matthews & Lawrence, 2005, by original designation. Status: valid genus in Diaperinae: incertae sedis. Note: Matthews and Lawrence (2019: 651) mentioned that this genus belongs to an unnamed tribe within the subfamily Diaperinae.

*Tripolicryptus* Strand, 1929: 24 [M]. Type species [automatic]: *Brachycryptustripolitanus* Quedenfeldt, 1891, by monotypy. Status: valid genus in Alleculinae: Cteniopodini. Note: replacement name for *Brachycryptus* Quedenfeldt, 1891.

*Trisilus* Haag-Rutenberg, 1878: 101 [M]. Type species: *Trisilusfemoralis* Haag-Rutenberg, 1878, by monotypy. Status: junior synonym of *Cyphaleus* Westwood, 1841 in Tenebrioninae: Heleini: Cyphaleina. Synonymy: Matthews (1992: 490).

*Trogloderus* J.L. LeConte, 1879: 2 [M]. Type species: *Trogloderuscostatus* J.L. LeConte, 1879, by monotypy. Status: valid genus in Blaptinae: Amphidorini.

*Troglogeneion* Aalbu, 1985: 541 [N]. Type species: *Troglogeneionzapoteca* Aalbu, 1985, by original designation. Status: valid genus in Pimeliinae: Edrotini.

*Tromosternus* Harold, 1876: 130 [M]. Type species: *Tromosternushaagi* Harold, 1876 (= *Gnesishelopioides* Pascoe, 1866), by monotypy. Status: junior synonym of *Gnesis* Pascoe, 1866 in Stenochiinae: Cnodalonini. Synonymy: Lewis (1894: 476).

*Tropidopterus* Cazurro Ruiz, 1897b: 637 [M]. Type species: *Tropidopteruscarinatus* Cazurro Ruiz, 1897, by original designation. Status: junior synonym of *Adelium* W. Kirby, 1819 in Lagriinae: Adeliini. Synonymy: Doyen et al. (1990: 230). Note: Tropidoptère was first used by Blanchard (1845: 35) but it was not formed as a scientific name and is therefore unavailable (ICZN 1999, Article 1.1); the latinized form of this name, *Tropidopterus*, was used subsequently (e.g., Lacordaire, 1859b: 438) but was not made available to our knowledge until Cazurro Ruiz (1897b: 637) published a combined description of a new genus and single new species (ICZN 1999, Article 12.2.6); since we believe that the combined description by Cazurro Ruiz (1897b : 637) was based on the same concept as the unavailable name “Tropidoptère Blanchard” we follow the intentions of authors in recent literature (e.g., Doyen et al. 1990: 230; Matthews 1998: 777; Matthews and Lawrence 2019: 615) and treat *Tropidopterus* Cazurro Ruiz, 1897 as a junior synonym of *Adelium* W. Kirby, 1819.

*Tropitrachys* Koch, 1955a: 229 [M]. Type species: *Trachynotusperegrinator* Koch, 1953, by original designation. Status: valid subgenus of *Somaticus* Hope, 1841 in Pimeliinae: Sepidiini: Trachynotina.

*Truncatocamaria* Pic, 1922b: 27 [F]. Type species: *Camariaspinipes* Pic, 1917 (as “*Camariaspinifer*”), by monotypy. Status: junior synonym of *Camaria* Lepeletier & Audinet-Serville, 1828 in Stenochiinae: Cnodalonini. Synonymy: Gebien (1942a: 320), Masumoto (1993a: 147).

*Truncatoodescelis* Kaszab, 1940b: 942, 962 [F]. Type species: *Platyscelislongicollis* Kraatz, 1884, by original designation. Status: valid subgenus of *Oodescelis* Motschulsky, 1845 in Blaptinae: Platyscelidini.

*Tuberocnodes* ​Gearner & Kamiński in Gearner et al., 2021: 8 [M]. Type species: *Psammodeshumeralis* ​Haag-Rutenberg, 1871, by original designation. Status: valid genus in Pimeliinae: Sepidiini: Molurina.

*Tucumana* Gebien, 1911b: 604 [F]. Type species [automatic]: *Eusteniatenuimembris* Fairmaire, 1905, by monotypy. Status: valid genus in Alleculinae: Alleculini: Xystropodina. Note: replacement name for *Eustenia* Fairmaire, 1905; placed in Alleculinae by Chatanay (1915a: 526).

*Turcmenicola* Bogatchev, 1952: 44 [M]. Type species: *Turcmenicolajachontovi* Bogatchev, 1952, by monotypy. Status: valid subgenus of *Colposcelis* Dejean, 1834 in Pimeliinae: Tentyriini.

*Turkmenohelops* G.S. Medvedev, 1987: 98, 102 [M]. Type species: *Zophohelopsbalchanicus* G.S. Medvedev & Nepesova, 1985, by original designation. Status: valid genus in Tenebrioninae: Helopini: Cylindrinotina.

*Turkonalassus* Keskin, Nabozhenko & Alpagut-Keskin, 2017: 727 [M]. Type species: *Helopsadimonius* Allard, 1876, by original designation. Status: valid genus in Tenebrioninae: Helopini: Cylindrinotina.

*Tydeolus* Champion, 1884: 37 [M]. Type species: *Tydeolusatratus* Champion, 1884, by subsequent designation (W.F. Kirby 1885b: 80). Status: valid genus in Pimeliinae: Epitragini.

*Tylophloeus* Bremer, 1998: 10, 13 [M]. Type species: *Hypophlaeusflavipennis* Motschulsky, 1860, by original designation. Status: valid subgenus of *Corticeus* Piller & Mitterpacher, 1783 in Diaperinae: Hypophlaeini.

*Tyndarisus* Pascoe, 1869: 294 [M]. Type species: *Tyndarisuslongitarsus* Pascoe, 1869 (= *Lepispilusstygianus* Pascoe, 1869), by monotypy. Status: junior synonym of *Pachycoelia* Boisduval, 1835 in Tenebrioninae: Heleini: Cyphaleina. Synonymy: Carter (1926: 119, with *Lepispilus* Westwood, 1841, a junior synonym of *Pachycoelia* Boisduval, 1835).

*Tynteria* Reitter, 1897a: 301 [F]. Type species: *Pachychilahumerosa* Fairmaire, 1875, by monotypy. Status: junior synonym of *Oterophloeus* Desbrochers des Loges, 1881 in Pimeliinae: Tentyriini. Synonymy: Reitter (1900c: 89, 143).

*Tynthlobia* Fairmaire, 1888d: 261 [F]. Type species: *Tynthlobiaquadricostata* Fairmaire, 1888, by monotypy. Status: junior synonym of *Ethmus* Haag-Rutenberg, 1873 in Pimeliinae: Sepidiini: Trachynotina. Synonymy: Fairmaire (1891b: 250).

*Typhlophloeus* Jeannel & Paulian, 1945: 51 [M]. Type species: *Typhlophloeuschappuisi* Jeannel & Paulian, 1945, by original designation. Status: valid genus in Diaperinae: Hypophlaeini.

*Typhluloma* Lea, 1912: 475 [N]. Type species: *Typhlulomainops* Lea, 1912, by monotypy. Status: valid genus in Tenebrioninae: Ulomini.

*Typhlusechus* Linell, 1897: 154 [M]. Type species: *Typhlusechussingularis* Linell, 1897, by original designation. Status: valid genus in Pimeliinae: Stenosini: Typhlusechina.

*Typhobia* Pascoe, 1869: 279 [F]. Type species: *Typhobiafuliginea* Pascoe, 1869, by monotypy. Status: junior synonym of *Platydema* Laporte & Brullé, 1831 in Diaperinae: Diaperini: Diaperina. Synonymy: Champion (1886: 181).

*Tyrtaeus* Champion, 1913: 76 [M]. Type species: *Tyrtaeusrufus* Champion, 1913, by original designation. Status: valid genus in Diaperinae: Gnathidiini: Anopidiina.

*Ubangia* Gebien, 1914e: 54 [F]. Type species: *Ubangialatifrons* Gebien, 1914, by monotypy. Status: junior synonym of *Crypsinous* Fairmaire, 1891 in Tenebrioninae: Amarygmini. Synonymy: Ardoin (1964a: 839).

*Ucalegon* Champion, 1884: 65 [M]. Type species: *Ucalegonpulchellus* Champion, 1884, by monotypy. Status: valid subgenus of *Pelecyphorus* Solier, 1836 in Pimeliinae: Asidini.

*Udebra* Reitter, 1896a: 236 [F]. Type species: *Udebrahauseri* Reitter, 1896 (= *Erodiusfimbriatus* Ménétriés, 1849), by monotypy. Status: junior synonym of *Adavius* Mulsant & Rey, 1859 in Blaptinae: Opatrini: Ammobiina. Synonymy: Kaszab (1942: 28).

*Uenomisolampidius* Masumoto, 1996a: 36 [M]. Type species: *Ueonomisolampidiusshunichii* Masumoto, 1996, by original designation. Status: valid genus in Stenochiinae: Cnodalonini.

*Uenostrongylium* Masumoto, 1999a: 123 [N]. Type species: *Cryptobateslaosensis* Pic, 1928, by original designation. Status: valid genus in Stenochiinae: Stenochiini.

*Uleda* Laporte, 1840: 220 [F]. Type species: *Ulomadiaperoides* Laporte, 1840, by monotypy. Status: valid genus in Tenebrioninae: Ulomini.

*Uloma* Dejean, 1821: 67 [N]. Type species: *Tenebrioculinaris* Linnaeus, 1758, by plenary powers (ICZN 1975, Opinion 1039). Status: valid genus and subgenus in Tenebrioninae: Ulomini. Note: placed on the Official List of Generic Names in Zoology (ICZN 1975, Opinion 1039). Note: the name *Uloma* is a Greek noun οὔλωμα (meaning cicatrisation) and the gender is neuter instead of the previously accepted feminine gender (e.g., ICZN 1975, Opinion 1039; see ICZN 2021, Official Correction 134).

*Ulomimus* Bates, 1873a: 201 [M]. Type species: *Ulomimusindicus* Bates, 1873, by monotypy. Status: valid genus in Tenebrioninae: Ulomini. Note: unjustified emendation of the original spelling *Ulomimimus*, introduced by Rye (1875: 288), in prevailing usage and treated as a justified emendation (ICZN 1999, Article 33.2.3.1).

*Ulomina* Baudi di Selve, 1876a: 112 [F]. Type species: *Ulominacarinata* Baudi di Selve, 1876, by monotypy. Status: valid genus in Tenebrioninae: Palorini. Note: redescribed as new by Baudi di Selve (1876b: 235).

*Ulomoides* Blackburn, 1888: 274 [M]. Type species: *Ulomoideshumeralis* Blackburn, 1888, by monotypy. Status: valid genus in Diaperinae: Diaperini: Diaperina.

*Ulomoides* Escalera, 1927: 501 [M]. Type species [automatic]: *Crypticusviaticus* Fairmaire, 1851, by original designation. Status: senior synonym of *Platycrypticus* Español, 1952 in Diaperinae: Crypticini. Note: junior homonym of *Ulomoides* Blackburn, 1888 [Coleoptera: Tenebrionidae: Diaperinae: Diaperini: Diaperina].

*Ulomotypus* Broun, 1886: 841 [M]. Type species: *Ulomotypuslaevigatus* Broun, 1886, by monotypy. Status: valid genus in Tenebrioninae: Palorini.

*Ulosonia* Laporte, 1840: 220 [F]. Type species: *Ulomatricorne* Laporte, 1840 (= *Phaleriatricornis* Dalman, 1823), by subsequent designation (Gebien 1940: 786). Status: junior synonym of *Hypogena* Dejean, 1834 in Tenebrioninae: Triboliini. Synonymy: Jacquelin du Val (1857: 148).

*Ulus* Horn, 1870: 349, 358 [M]. Type species: *Blapstinuscrassus* J.L. LeConte, 1851, by subsequent designation (R. Lucas 1920: 665). Status: valid genus in Blaptinae: Opatrini: Blapstinina.

*Umslatus* Péringuey, 1899: 312 [M]. Type species: *Umslatusagilis* Péringuey, 1899, by monotypy. Status: valid genus in Tenebrioninae: Amarygmini.

*Uniungulum* Koch, 1962b: 113 [N]. Type species: *Uniungulumhoeschi* Koch, 1962, by original designation. Status: valid genus in Pimeliinae: Sepidiini: Hypomelina.

*Upembarus* Koch, 1956a: 220 [M]. Type species: *Upembarussaegeri* Koch, 1956, by original designation. Status: valid genus and subgenus in Blaptinae: Platynotini: Platynotina.

*Upinella* Mulsant, 1857: 17 [F]. Type species: *Alleculaaterrima* Rosenhauer, 1847, by monotypy. Status: valid genus and subgenus in Alleculinae: Alleculini: Alleculina.

*Upis* Fabricius, 1792: 515 [F]. Type species: *Attelabusceramboides* Linnaeus, 1758, by monotypy. Status: valid genus in Stenochiinae: Cnodalonini.

*Uptona* G.S. Medvedev & Lawrence, 1986: 582 [F]. Type species: *Uptonapallida* G.S. Medvedev & Lawrence, 1986, by original designation. Status: valid genus in Diaperinae: Hyociini: Uptonina.

*Uria* Gistel, 1848a: x [F]. Type species [automatic]: *Tenebriocadaverinus* Fabricius, 1792, by subsequent designation (Westwood 1838: 32). Status: junior synonym of *Phaleria* Latreille, 1802 in Diaperinae: Phaleriini. Note: unnecessary replacement name for *Phaleria* Latreille, 1802.

*Uriela* Reitter, 1887b: 518 [F]. Type species: *Podhomalafausti* Kraatz, 1881, by monotypy. Status: junior synonym of *Podhomala* Solier, 1836 in Pimeliinae: Pimeliini. Synonymy: Sénac (1888: lv).

*Urielina* Reitter, 1888: 331 [F]. Type species: *Podhomalanitida* Baudi di Selve, 1876, by monotypy. Status: valid subgenus of *Podhomala* Solier, 1836 in Pimeliinae: Pimeliini.

*Uroblaps* Motschulsky, 1860c: 530 [F]. Type species: *Blaps producta* Brullé, 1832 (= *Blaps lusitanica* Herbst, 1799), by subsequent designation (Nabozhenko 2008: 36). Status: junior synonym of *Blaps* Fabricius, 1775 in Blaptinae: Blaptini: Blaptina. Synonymy: Gemminger in Gemminger and Harold (1870: 1860)

*Uroplatopsis* Champion, 1889: 53 [F]. Type species: *Uroplatopsisimitator* Champion, 1889, by subsequent designation (R. Lucas 1920: 666). Status: valid genus in Lagriinae: Lagriini: Statirina.

*Uroprosodes* Reitter, 1909a: 119 [M]. Type species: *Prosodescostifera* Kraatz, 1886, by original designation. Status: valid subgenus of *Prosodes* Eschscholtz, 1829 in Blaptinae: Blaptini: Prosodina.

*Uyttenboogaartia* Koch, 1943a: 592, 595 [F]. Type species: *Hegetercribricollis* Brullé, 1839, by original designation. Status: valid genus in Pimeliinae: Tentyriini.

*Uzagaria* Ancey, 1881: 509 [F]. Type species: *Uzagariapubens* Ancey, 1881, by monotypy. Status: junior synonym of *Emmalus* Erichson, 1843 in Blaptinae: Opatrini: Ammobiina. Synonymy: Gebien (1910b: 306, as *Emmallus*).

†*Vabole* Alekseev & Nabozhenko, 2015: 128 [F]. Type species: *Vaboletriplehorni* Alekseev & Nabozhenko, 2015, by original designation. Status: valid genus in Tenebrioninae: Palorini. Note: described from Eocene Baltic amber.

*Vacronus* Casey, 1907: 501, 508 [M]. Type species: *Vacronustenuicornis* Casey, 1907, by original designation. Status: junior synonym of *Alaephus* Horn, 1870 in Pimeliinae: Vacronini. Synonymy: Doyen and Lawrence (1979: 350).

*Vadalus* Mulsant & Rey, 1853b: 150 [M]. Type species: *Pedinuspunctulatus* Mulsant & Rey, 1853, by monotypy. Status: junior synonym of *Pedinus* Latreille, 1797 in Blaptinae: Pedinini: Pedinina. Synonymy: Gemminger in Gemminger and Harold (1870: 1918), Kamiński and Iwan (2017: 599).

*Valdivium* Matthews, 1998: 709, 721 [N]. Type species: *Adeliumsulcatulum* Fairmaire & Germain, 1860, by original designation. Status: valid genus in Lagriinae: Adeliini.

*Vaniosus* Kulzer, 1956b: 896 [M]. Type species: *Vaniosusparadoxus* Kulzer, 1956, by original designation. Status: valid genus in Pimeliinae: Evaniosomini.

*Vansonium* Koch, 1950b: 354 [N]. Type species: *Vansoniumbushmanicum* Koch, 1950, by original designation. Status: valid genus in Pimeliinae: Cryptochilini: Vansoniina.

*Varogeton* Bremer, 2014a: 37, 80 [M]. Type species: *Dietysussubannulipes* Pic, 1923, by original designation. Status: valid subgenus of *Amarygmus* Dalman, 1823 in Tenebrioninae: Amarygmini.

*Vernayella* Koch, 1958: 129 [F]. Type species: *Vernayellanoctivaga* Koch, 1958, by original designation. Status: valid subgenus of *Caenocrypticus* Gebien, 1920 in Pimeliinae: Caenocrypticini.

*Vieta* Laporte, 1840: 196 [F]. Type species: *Sepidium vestitum* Guérin-Méneville, 1831, by subsequent designation (Hope 1841: 116). Status: valid genus in Pimeliinae: Sepidiini: Sepidiina.

*Vietnalia* Novák, 2021: 440 [F]. Type species: *Vietnaliacatcatica* Novák, 2021, by original designation. Status: valid genus in Alleculinae: Alleculini: Alleculina.

*Vietomorpha* Fairmaire, 1887a: 186 [F]. Type species: *Vietomorphafoveipennis* Fairmaire, 1887, by monotypy. Status: valid genus in Pimeliinae: Sepidiini: Sepidiina.

*Viettagona* G.S. Medvedev & Merkl, 2003: 317, 328 [F]. Type species: *Viettagonavietnamensis* G.S. Medvedev & Merkl, 2003, by original designation. Status: valid genus in Blaptinae: Blaptini: Gnaptorinina.

*Villiersia* Gridelli, 1951: 219, 227 [F]. Type species: *Tenebrioclypealis* Gebien, 1920, by original designation. Status: senior synonym of *Gridellia* Kammerer, 2006 in Tenebrioninae: Tenebrionini. Note: junior homonym of *Villiersia* d’Orbigny, 1837 [Mollusca].

*Viriathus* Fairmaire, 1902b: 339 [M]. Type species: *Viriathusstrigipennis* Fairmaire, 1902, by monotypy. Status: valid genus in Alleculinae: Alleculini: Gonoderina.

*Vizcainyx* Aalbu & Smith, 2020: 198 [M]. Type species: *Vizcainyxandrewsi* Aalbu & Smith, 2020, by original designation. Status: valid genus in Pimeliinae: Edrotini.

*Vutsimus* Péringuey, 1899: 308 [M]. Type species: *Vutsimuspraetorius* Péringuey, 1899, by subsequent designation (Gebien 1943: 922). Status: valid genus in Tenebrioninae: Amarygmini.

*Wahlbergylium* Ferrer, 2011: 141 [N]. Type species: *Wahlbergyliumviklundi* Ferrer, 2011, by original designation. Status: junior synonym of *Argutiolana* Robiche, 2001 in Stenochiinae: Cnodalonini. Synonymy: Robiche (2019a: 97).

*Wallardilagria* Pic, 1910: 74 [F]. Type species: *Heterogriapallidicolor* Pic, 1910, by monotypy. Status: junior synonym of *Xanthalia* Fairmaire, 1894 in Lagriinae: Lagriini: Statirina. Synonymy: Merkl (2004: 285).

*Warchalowskiellus* Iwan, 1998a: 60 [M]. Type species: *Trigonopuslongulus* Mulsant & Rey, 1853, by original designation. Status: junior synonym of *Schelodontes* Koch, 1956 in Blaptinae: Platynotini: Platynotina. Synonymy: Iwan and Kamiński (2014: 171).

*Waterhousia* Skopin, 1973: 109, 110 [F]. Type species: *Trigonoscelislongipes* C.O. Waterhouse, 1889, by original designation. Status: valid genus in Pimeliinae: Pimeliini.

*Wattadelium* Emberson, 2000: 24 [N]. Type species [automatic]: *Edalusopacus* Broun, 1893, by subsequent designation (R. Lucas 1920: 255). Status: valid genus in Lagriinae: Adeliini. Note: replacement name for *Edalus* Broun, 1893.

*Wattiana* Matthews & Lawrence, 2005: 537 [F]. Type species: *Wattianagreensladei* Matthews & Lawrence, 2005, by original designation. Status: valid genus in Pimeliinae: Cnemeplatiini: Thorictosomatina.

*Wattius* Kaszab, 1982b: 50 [M]. Type species: *Calymmuscucullatus* Pascoe, 1871, by original designation. Status: valid genus in Tenebrioninae: Toxicini: Dysantina.

*Weisea* Semenov, 1891: 370 [F]. Type species: *Weiseasabulicola* Semenov, 1891, by monotypy. Status: valid genus in Blaptinae: Opatrini: Ammobiina.

*Wolladrus* Iwan & Kamiński, 2016: 483 [M]. Type species [automatic]: *Hadrusalpinus* Wollaston, 1854, by subsequent designation (R. Lucas 1920: 313). Status: valid genus in Blaptinae: Opatrini: Opatrina. Note: replacement name for *Hadrus* Wollaston, 1854.

*Xanthalia* Fairmaire, 1894f: 395 [F]. Type species [automatic]: *Xanthiacurticollis* Fairmaire, 1893, by monotypy. Status: valid genus in Lagriinae: Lagriini: Statirina. Note: replacement name for *Xanthia* Fairmaire, 1893; **new placement** [OM], previously included in Lagriinae: Lagriini: Lagriina.

*Xanthia* Fairmaire, 1893b: 31 [F]. Type species: *Xanthiacurticollis* Fairmaire, 1893, by monotypy. Status: senior synonym of *Xanthalia* Fairmaire, 1894 in Lagriinae: Lagriini: Statirina. Note: junior homonym of *Xanthia* Hübner, 1813 [Lepidoptera].

*Xanthicles* Champion, 1886: 231 [M]. Type species: *Xanthiclescaraboides* Champion, 1886, by subsequent designation (Gebien 1941: 815). Status: valid genus in Lagriinae: Goniaderini.

*Xanthobates* Gebien, 1928: 170, 185 [M]. Type species: *Xanthobatesflavus* Gebien, 1928, by monotypy. Status: valid genus in Stenochiinae: Cnodalonini.

*Xanthohelops* Nabozhenko, 2006: 822 [M]. Type species: *Xanthohelopskarakumicus* Nabozhenko & G.S. Medvedev, 2006, by original designation. Status: valid genus in Tenebrioninae: Helopini: Cylindrinotina.

*Xanthomus* Mulsant, 1854: 302 [M]. Type species: *Helopspallidus* Curtis, 1830, by monotypy. Status: valid genus in Tenebrioninae: Helopini: Cylindrinotina.

*Xanthothopeia* Mäklin, 1867: 223 [F]. Type species: *Xanthothopeiarufipennis* Mäklin, 1867, by monotypy. Status: junior synonym of *Strongylium* W. Kirby, 1819 in Stenochiinae: Stenochiini. Synonymy: Gebien (1948: 519).

*Xanthothopia* Gemminger in Gemminger and Harold, 1870: 2001 [F]. Type species [automatic]: *Xanthothopeiarufipennis* Mäklin, 1867, by monotypy. Status: junior synonym of *Strongylium* W. Kirby, 1819 in Stenochiinae: Stenochiini. Note: unjustified emendation of *Xanthothopeia* Mäklin, 1867, not in prevailing usage.

*Xantusiella* Kaszab, 1941a: 4, 18 [F]. Type species: *Xantusiellacrenulata* Kaszab, 1941, by original designation. Status: valid genus in Stenochiinae: Cnodalonini.

*Xenius* Champion, 1886: 224 [M]. Type species: *Xeniusscabripennis* Champion, 1886, by monotypy. Status: valid genus in Stenochiinae: Cnodalonini.

*Xenocera* Borchmann, 1936: 18, 116 [F]. Type species: *Lagriocerafeai* (as “*feae*”) Borchmann, 1911, by original designation. Status: senior synonym of *Xenocerogria* Merkl, 2007 in Lagriinae: Lagriini: Lagriina. Note: junior homonym of *Xenocera* Broun, 1881 [Coleoptera: Ptinidae].

*Xenocerogria* Merkl, 2007: 269 [F]. Type species [automatic]: *Lagriocerafeai* (as “*feae*”) Borchmann, 1911, by original designation. Status: valid genus in Lagriinae: Lagriini: Lagriina. Note: replacement name for *Xenocera* Borchmann, 1936.

*Xenogena* Borchmann, 1936: 22, 211 [F]. Type species: *Adynatacrinita* Borchmann, 1915, by original designation. Status: valid genus in Lagriinae: Lagriini: Lagriina.

*Xenogloeus* Wollaston, 1861: 251 [M]. Type species: *Xenogloeuspolitus* Wollaston, 1861, by monotypy. Status: valid genus in Tenebrioninae: Triboliini.

*Xenolagria* Merkl, 1987: 124, 126 [F]. Type species: *Lagriatincta* Blackburn, 1889, by original designation. Status: valid genus in Lagriinae: Lagriini: Lagriina.

*Xenostethus* Bates, 1868: 321 [M]. Type species: *Xenostethuslacordairii* Bates, 1868, by monotypy. Status: valid genus in Lagriinae: Lagriini: Statirina.

*Xenostira* Borchmann, 1921: 217, 221 [F]. Type species: *Xenostiragiraffa* Borchmann, 1921, by original designation. Status: valid subgenus of *Statira* Lepeletier & Audinet-Serville, 1828 in Lagriinae: Lagriini: Statirina.

*Xenotermes* Wasmann, 1896: 616 [M]. Type species: *Xenotermesfeai* (as “*feae*”) Wasmann, 1896, by monotypy. Status: valid genus in Tenebrioninae: Rhysopaussini.

*Xenus* Péringuey, 1899: 255 [M]. Type species: *Xenustricorniger* Péringuey, 1899, by monotypy. Status: senior synonym of *Aphrotus* Péringuey, 1904 in Pimeliinae: Epitragini. Note: junior homonym of *Xenus* Kaup, 1829 [Aves].

*Xerolinus* Ivie & Hart, 2016: 470 [M]. Type species: *Diastolinussallei* Mulsant & Rey, 1859, by original designation. Status: valid genus in Blaptinae: Opatrini: Blapstinina.

*Xyloborus* Motschulsky, 1858a: 64 [M]. Type species: *Xyloboruscrenipennis* Motschulsky, 1858, by monotypy. Status: senior synonym of *Rhipidandrus* J.L. LeConte, 1862 in Tenebrioninae: Bolitophagini. Synonymy: Merkl and Kompantzeva (1996: 91). Note: although Schwarz and Barber (1914: 175) pointed out that the name *Xyloborus* was made available for the first time by Motschulsky (1858: 64) there are few occurrences of this name in the literature; reversal of precedence cannot be used to conserve usage of the broadly used name *Rhipidandrus* J.L. LeConte, 1862 since *Xyloborus* was used as valid after 1899 (e.g., Swezey 1942: 167); an application to the ICZN is necessary to conserve usage *Rhipidandrus* J.L. LeConte, 1862; the older names *Xyloborus* Kirby & Spence, 1828 and *Xyloborus* Dejean, 1834 [Coleoptera] are unavailable because they were published before 1931 without a description, a definition or an indication (ICZN 1999, Article 12.1).

*Xylochus* Broun, 1880: 396 [M]. Type species: *Xylochustibialis* Broun, 1880, by subsequent designation (Watt 1992: 24). Status: valid genus in Alleculinae: Alleculini: incertae sedis.

*Xylopinus* J.L. LeConte, 1862: 230 [M]. Type species: *Tenebrioanthracinus* Knoch, 1801 (= *Tenebriosaperdoides* G.-A. Olivier, 1795), by subsequent designation (Gebien 1941: 336). Status: valid genus in Stenochiinae: Cnodalonini.

*Xysta* Eschscholtz, 1829: 9 [F]. Type species: *Eleodesgravida* Eschscholtz, 1829, by subsequent designation (Hope 1841: 124). Status: senior synonym of *Steneleodes* Blaisdell, 1909 in Blaptinae: Amphidorini. Synonymy: Smith and Johnston in Bousquet et al. (2018: 166). Note: junior homonym of *Xysta* Meigen, 1824 [Diptera].

*Xystronia* Solier, 1835a: 238 [F]. Type species: *Xystroniacoerulea* Solier, 1835, by monotypy. Status: valid subgenus of *Lystronychus* Latreille, 1829 in Alleculinae: Alleculini: Xystropodina.

*Xystropus* Solier, 1835a: 241 [M]. Type species: *Xystropuspilosus* Solier, 1835, by monotypy. Status: valid genus in Alleculinae: Alleculini: Xystropodina. Note: see Bousquet et al. (2015: 139) for comments regarding the species originally included in this genus.

*Yamatotakeru* Ando, 2015: 385 [M]. Type species: *Ischnodactylusloripes* Lewis, 1894, by original designation. Status: valid genus in Diaperinae: Diaperini: Diaperina.

†*Yantaroxenos* Nabozhenko, Kirejtshuk & Merkl, 2016c: 564 [M]. Type species: *Yantaroxenoscolydioides* Nabozhenko, Kirejtshuk & Merkl, 2016, by original designation. Status: valid genus in Lagriinae: Belopini. Note: described from Eocene Baltic amber.

*Yarranum* Matthews, 1998: 708, 734 [N]. Type species: *Seirotranacrenicollis* Pascoe, 1869, by original designation. Status: valid genus in Lagriinae: Adeliini.

*Zabroideus* Fairmaire, 1894c: 219 [M]. Type species: *Zabroideuspinguis* Fairmaire, 1894, by monotypy. Status: valid genus in Stenochiinae: Cnodalonini.

*Zadenos* Laporte, 1840: 210 [M]. Type species: *Opatrumlongipalpe* Wiedemann, 1823, by monotypy. Status: valid subgenus of *Selenepistoma* Dejean, 1834 in Blaptinae: Dendarini: Melambiina. Note: this name was recently treated as a valid genus (Kamiński 2015: 531); however, the older available genus name *Selenepistoma* Dejean, 1834 has priority and therefore Zadenos is downgraded to a valid subgenus of Dejean’s name; **new status**.

*Zaleucus* Champion, 1892: 491 [M]. Type species [automatic]: *Zamolxisdilatatus* Champion, 1884, by monotypy. Status: valid subgenus of *Pelecyphorus* Solier, 1836 in Pimeliinae: Asidini. Note: replacement name for *Zamolxis* Champion, 1884.

*Zambesmia* Bouchard & Bousquet, **new subgenus** [F]. Type species: *Macropodachiyakensis* Kuntzen, 1916, by **present designation**. Status: valid subgenus of *Adesmia* Fischer, 1822 in Pimeliinae: Adesmiini. Note: Koch (1944b: 149) introduced the new subgenus name *Zambesmia* for three nominal species, but unfortunately did not designate a type species; the subgenusZambesmia, which has been treated as valid since 1944, is therefore unavailable (ICZN 1999, Article 13.3); we hereby make the name available by selecting *Macropodachiyakensis* Kuntzen, 1916 as type species and referring to Koch (1944b: 149) for the character states that characterise and differentiate *Zambesmia*.

*Zamolxis* Champion, 1884: 70 [M]. Type species: *Zamolxisdilatatus* Champion, 1884, by monotypy. Status: senior synonym of *Zaleucus* Champion, 1892 in Pimeliinae: Asidini. Note: junior homonym of *Zamolxis* Stål, 1865 [Hemiptera].

*Zarudnionymus* Semenov-Tjan-Shansky & Bogatchev, 1947: 175 [M]. Type species: *Zarudnionymuspersis* Semenov-Tjan-Shansky & Bogatchev, 1947 (= *Adelostomagrande* Haag-Rutenberg, 1879), by monotypy. Status: valid subgenus of *Adelostoma* Duponchel, 1827 in Pimeliinae: Adelostomini.

*Zeadelium* Watt, 1992: 32 [N]. Type species: *Adeliumlentum* Broun, 1880, by original designation. Status: valid genus in Lagriinae: Adeliini.

*Ziaelas* Fairmaire, 1892e: cx [M]. Type species: *Ziaelasinsolitus* Fairmaire, 1892, by monotypy. Status: valid genus in Tenebrioninae: Amarygmini.

*Zidalus* Mulsant & Rey, 1853b: 71 [M]. Type species: *Opatrinuscorvinus* Mulsant & Rey, 1853, by monotypy. Status: valid genus in Blaptinae: Platynotini: Platynotina.

*Zizu* Novák, 2019b: 186 [M]. Type species: *Zizukejvali* Novák, 2019, by original designation. Status: valid genus in Alleculinae: Alleculini: Alleculina.

*Zodinus* Mulsant & Rey, 1853b: 90 [M]. Type species: *Opatrinusservus* Mulsant & Rey, 1853, by subsequent designation (Koch 1956a: 93). Status: junior synonym of *Zidalus* Mulsant & Rey, 1853 in Blaptinae: Platynotini: Platynotina. Synonymy and First Reviser action (*Zodinus* Mulsant & Rey, 1853 versus *Zidalus* Mulsant & Rey, 1853) is Iwan (1995b: 362).

*Zolodinus* Blanchard, 1853: 159 [M]. Type species: *Zolodinuszelandicus* Blanchard, 1853, by monotypy. Status: valid genus in Zolodininae.

*Zomedes* Watt, 1992: 25 [M]. Type species: *Zomedesborealis* Watt, 1992, by original designation. Status: valid genus in Alleculinae: Alleculini: incertae sedis.

*Zophius* Dejean, 1834: 189 [M]. Type species: *Helopsrufopictus* Wiedemann, 1823, by monotypy. Status: valid genus in Stenochiinae: Cnodalonini.

*Zophobas* Dejean, 1834: 204 [M]. Type species: *Helopsmorio* Fabricius, 1777 (= *Tenebrioatratus* Fabricius, 1775), by subsequent designation (Motschulsky 1872: 26). Status: valid genus and subgenus in Tenebrioninae: Tenebrionini. Note: according to Matthews and Lawrence (2019: 628) more research is needed to establish the correct placement of this genus within the subfamily Tenebrioninae.

*Zophodes* Fåhraeus, 1870: 298 [M]. Type species: *Zophodestristis* Fåhraeus, 1870, by monotypy. Status: valid genus in Blaptinae: Platynotini: Platynotina.

*Zophohelops* Reitter, 1902a: 221 [M]. Type species [automatic]: *Euryhelopstiro* Reitter, 1902, by subsequent designation (Gebien 1943: 424). Status: valid genus and subgenus in Tenebrioninae: Helopini: Cylindrinotina. Note: replacement name for *Euryhelops* Reitter, 1902.

*Zophondrus* Nabozhenko, 2014: 240 [M]. Type species: *Zophohelopsiranensis* Nabozhenko, 2014, by original designation. Status: valid subgenus of *Zophohelops* Reitter, 1902 in Tenebrioninae: Helopini: Cylindrinotina.

*Zophophilus* Fairmaire, 1881b: 359 [M]. Type species: *Zophophiluscurticornis* Fairmaire, 1881, by monotypy. Status: valid genus in Stenochiinae: Cnodalonini.

*Zophoserodius* Reitter, 1914a: 58, 60 [M]. Type species: *Erodiuszophosoides* Allard, 1865, by subsequent designation (Löbl et al. 2008a: 43). Status: valid subgenus of *Erodius* Fabricius, 1775 in Pimeliinae: Erodiini.

*Zophosis* Latreille, 1802: 167 [F]. Type species: *Erodiustestudinarius* Fabricius, 1781, by monotypy. Status: valid genus and subgenus in Pimeliinae: Zophosini.

*Zophosodactylus* Koch, 1962b: 146 [M]. Type species: *Protodactylussanctaemariae* Koch, 1962, by original designation. Status: junior synonym of *Protodactylus* Koch, 1952 in Pimeliinae: Zophosini. Synonymy: Penrith (1981c: 129).

*Zoutpansbergia* Koch, 1956a: 388 [F]. Type species: *Zoutpansbergiaserricostata* Koch, 1956, by monotypy. Status: valid genus in Blaptinae: Dendarini: Melambiina. Note: combined description of new genus-group taxon and new species (ICZN 1999, Article 13.4).

*Zuercheria* Reitter, 1908: 134 [F]. Type species: *Zuercheriamatthiesseni* Reitter, 1908, by subsequent designation (Löbl et al. 2008a: 43). Status: junior synonym of *Strongylium* W. Kirby, 1819 in Stenochiinae: Stenochiini. Synonymy: Gebien (1911b: 590).

*Zygas* Pascoe, 1866a: 487 [M]. Type species: *Eurychoracimicoides* Quensel, 1806, by original designation. Status: junior synonym of *Lycanthropa* J. Thomson, 1860 in Pimeliinae: Adelostomini. Synonymy: Haag-Rutenberg (1875b: 61–64).

*Zypoetes* Champion, 1893a: 532 [M]. Type species: *Zypoetesepieroides* Champion, 1893, by monotypy. Status: valid genus in Phrenapatinae: Penetini.

## Appendix 1

List of unavailable genus-group names in Tenebrionidae Latreille, 1802. Each entry includes the name, its usage in the literature (or one example when the name was used more than once), and the reason for treating it as unavailable. See the Methods section for additional information. The incorrect subsequent spellings below are not in prevailing usage.

*Acanthodactylus*; Escalera, 1914: 347; incorrect subsequent spelling for *Acontodactylus* Desbrochers des Loges, 1894.

*Acanthopus*; Latreille, 1829a: 38; incorrect subsequent spelling for *Accanthopus* Dejean, 1821.

*Achaemenus*; G.S. Medvedev and Iwan, 2006: 618; incorrect subsequent spelling for *Achaemenes* Bogatchev, 1949.

*Acilagria*; Borchmann, 1916a: 90; published before 1931 without a description, a definition, or an indication.

*Acmaeus*; Gebien, 1910b: 304; incorrect subsequent spelling for *Acmoeus* Fåhraeus, 1870.

*Acrothymus*; Gemminger in Gemminger and Harold, 1870: 2009; incorrect subsequent spelling for *Arcothymus* Pascoe, 1866.

*Acthosus*; Carter, 1906: 251; incorrect subsequent spelling for *Achthosus* Pascoe, 1863.

*Acysba*; Villa and Villa, 1833: 18; published before 1931 without a description, a definition, or an indication.

*Adosagria*; Pic, 1955: 179, 180; incorrect subsequent spelling for *Adosogria* Borchmann, 1936.

*Aediotorix*; Gebien, 1905: 258; incorrect subsequent spelling for *Aediatorix* Bates, 1868.

*Aemyone*; Ferrer and Delatour, 2007: 275, 276; incorrect subsequent spelling for *Aemymone* Bates, 1868.

*Afghanopachya*; Anonymous in Staff of BIOSIS, 1981: 283; incorrect subsequent spelling for *Afghanopachys* Kwieton, 1978.

*Afghanprosodes*; Schuster, 1936: 197; published after 1930 without a type species designation.

*Agapetus*; Dejean, 1834: 212; published before 1931 without a description, a definition, or an indication.

*Agroecus*; Chatanay, 1915: 508; incorrect subsequent spelling for *Agraecus* Fairmaire, 1900.

*Agymnonix*; Kaszab, 1980b: 320; incorrect subsequent spelling for *Agymnonyx* Gebien, 1921.

*Akixa*; Guérin-Méneville, 1834: 13; incorrect subsequent spelling for *Akis* Herbst, 1799.

*Alcione*; Reitter, 1897a: 300; incorrect subsequent spelling for *Alcinoe* Ménétriés, 1849.

*Alcmaeonis*; Blackburn, 1893b: 133; incorrect subsequent spelling for *Alcmeonis* Bates, 1868.

*Alcyone*; Kraatz, 1865: 80; incorrect subsequent spelling for *Alcinoe* Ménétriés, 1849.

*Aletha*; Poole and Gentili, 1996: 425; incorrect subsequent spelling for *Alethia* Champion, 1888.

*Alienolonyx*; Bremer, 2019: 60; alternative original spelling of *Alienoplonyx* Bremer, 2019, herein rejected.

*Allegoria*; Gemminger in Gemminger and Harold, 1870: 1959; incorrect subsequent spelling for *Alegoria* Laporte, 1840.

*Allolagria*; Sharp, 1922: 144; incorrect subsequent spelling for *Allogria* Borchmann, 1916.

*Alphitoplogus*; Motschulsky, 1873: 474; incorrect subsequent spelling for *Alphitophagus* Stephens, 1832.

*Amacarus*; Dejean, 1834: 212; published before 1931 without a description, a definition, or an indication.

*Amarigmus*; Hope, 1841: 75; incorrect subsequent spelling for *Amarygmus* Dalman, 1823.

*Amathodes*; Erichson, 1845a: 114; incorrect subsequent spelling for *Amatodes* Dejean, 1834.

*Amblychara*; Reitter, 1900c: 94; incorrect subsequent spelling for *Amblycara* Fairmaire, 1893.

*Amblycyphrus*; Aalbu et al. 1995: 481; incorrect subsequent spelling for *Amblycyphus* Motschulsky, 1870.

*Ammophthorus*; Jacquelin du Val, 1861: 288; incorrect subsequent spelling for *Ammophtorus* Lacordaire, 1859.

*Amophorus*; Dejean, 1836: 203; incorrect subsequent spelling for *Ammophorus* Guérin-Méneville, 1831.

*Amozoum*; Reitter, 1914a: 45, 50; incorrect subsequent spelling for *Ammozoum* Semenov, 1891.

*Amphitrix*; Koch, 1956a: 49; incorrect subsequent spelling for *Amphithrix* Español, 1953.

*Amphydora*; Dejean, 1834: 210; incorrect subsequent spelling for *Amphidora* Eschscholtz, 1829.

*Amphysus*; Dejean, 1834: 189; published before 1931 without a description, a definition, or an indication.

*Amplypteraca*; Mas-Peinado et al., 2018: 531, 543; alternative original spelling of *Amblypteraca* Mas-Peinado, Buckley, Ruiz & García-París, 2018, herein rejected.

*Amyone*; Ferrer and Delatour, 2007: 279; incorrect subsequent spelling for *Aemymone* Bates, 1868.

*Anadesis*; Laporte, 1840: 185; incorrect subsequent spelling for *Anodesis* Solier, 1834.

*Anaemia*; Horn, 1870: 377; incorrect subsequent spelling for *Anemia* Laporte, 1840.

*Anarmastodera*; Schawaller, 2010: 285; incorrect subsequent spelling for *Anarmostodera* Fairmaire, 1897.

*Anchophtalmus*; Lesne, 1922: 703; incorrect subsequent spelling for *Anchophthalmus* Gerstaecker, 1854.

*Aniosis*; Ferreira, 1967: 741; incorrect subsequent spelling for *Anisosis* Deyrolle, 1867.

*Anisochara*; Löbl et al. 2008b: 309; incorrect subsequent spelling for *Anisocara* Gebien, 1925.

*Anisocheira*; Dejean, 1834: 197; published before 1931 without a description, a definition, or an indication.

*Anisocrepis*; Dejean, 1834: 198; published before 1931 without a description, a definition, or an indication.

*Anodesia*; Agassiz, 1846a: 11; incorrect subsequent spelling for *Anodesis* Solier, 1834.

*Anoedus*; Blanchard, 1845: 35; incorrect subsequent spelling for *Anaedus* Blanchard, 1842.

*Anomaearthrum*; Aalbu et al., 2002: 501; incorrect subsequent spelling for *Anomoearthrum* Mäklin, 1867.

*Anomalipes*; Guérin-Méneville, 1831b: pl. 29; incorrect original spelling, emended to *Anomalipus* Guérin-Méneville, 1831, under prevailing usage.

*Anthcarohelops*; Nabozhenko, 2019: 7; incorrect subsequent spelling for *Anthracohelops* Haupt, 1950.

*Antocera*; Ragusa, 1898: 121; incorrect subsequent spelling for *Autocera* Wollaston, 1857.

*Apatocerus*; Schawaller, 2013: 68; incorrect subsequent spelling for *Apistocerus* Fairmaire, 1899.

*Apaxo*; Fairmaire, 1877b: 167; incorrect subsequent spelling for *Anaxo* Bates, 1868.

*Aphthora*; Champion, 1895b: 148; incorrect subsequent spelling for *Aphtora* Bates, 1872.

*Aplocheirus*; Macquart, 1850: 191; published before 1931 without a description, a definition or an indication.

*Archeoglenes*; Doyen and Lawrence, 1979: 333; incorrect subsequent spelling for *Archaeoglenes* Broun, 1893.

*Arcolymus*; Gebien, 1942a: 745; incorrect subsequent spelling for *Arcothymus* Pascoe, 1866.

*Argasidius*; Gebien, 1937a: 674; incorrect subsequent spelling for *Argasidus* Péringuey, 1899.

*Argenis*; Burmeister, 1875: 463; incorrect subsequent spelling for *Aryenis* Bates, 1868.

*Arrhenoplitis*; Gemminger in Gemminger and Harold, 1870: 1949; incorrect subsequent spelling for *Arrhenoplita* W. Kirby, 1837.

*Arthrocomus*; Gebien, 1911b: 614; incorrect subsequent spelling for *Arthroconus* Solier, 1851.

*Arthrodactyla*; Laporte, 1840: 211; incorrect subsequent spelling for *Athrodactyla* Klug, 1833.

*Ascalabos*; Fairmaire, 1894f: 395; incorrect subsequent spelling for *Ascalabus* Fairmaire, 1893.

*Asdidius*; Rye, 1870: 268; incorrect subsequent spelling for *Asididius* Fairmaire, 1869.

*Asidax*; Gistel, 1848b: 149; incorrect subsequent spelling for *Asida* Latreille, 1802.

*Aspicephalus*; Motschulsky, 1839: 63; incorrect original spelling, emended to *Aspidocephalus* Motschulsky, 1839, under prevailing usage.

*Aspidus*; Gebien, 1910b: 298; incorrect subsequent spelling for *Aspidius* Mulsant & Rey, 1859.

*Astenochirus*; R. Lucas, 1920: 123; incorrect subsequent spelling for *Asthenochirus* Fairmaire, 1885

*Astenorhinus*; Fairmaire, 1894g: 664; incorrect subsequent spelling for *Asthenochirus* Fairmaire, 1885.

*Asthenorhinus*; Kolbe, 1897b: 611; incorrect subsequent spelling for *Asthenochirus* Fairmaire, 1885.

*Asyleplus*; Gebien, 1943: 922; incorrect subsequent spelling for *Asyleptus* Péringuey, 1896.

*Batulinus*; Papp, 1961: 105; incorrect subsequent spelling for *Batulius* J.L. LeConte, 1851.

*Beplegenes*; Carter, 1926: 128; incorrect subsequent spelling for *Blepegenes* Pascoe, 1868.

*Biophanes*; Marseul, 1857: 117; incorrect subsequent spelling for *Bioplanes* Mulsant, 1854.

*Blapicauda*; Ren et al., 2000: 48; published after 1930 without a type species designation.

*Blapiplanula*; Ren et al., 2000: 49; published after 1930 without a type species designation.

*Blapsibreva*; Ren et al., 2000: 49; published after 1930 without a type species designation.

*Blaptinus*; Latreille, 1829a: 21; incorrect subsequent spelling for *Blapstinus* Dejean, 1821.

*Blaptyscelis*; Koch, 1965: 127; published after 1930 without a type species designation.

*Blaptyscellis*; Koch in Pierre, 1962: 213; published after 1930 without a type species designation.

*Brachicula*; Fairmaire, 1906: 278; alternative original spelling of *Brachycula*, rejected by Bousquet et al. (2015: 143).

*Brachydium*; Kaszab, 1939b: 187; incorrect subsequent spelling for *Brachyidium* Fairmaire, 1883.

*Brachypophloeus*; Alluaud, 1899: 342; incorrect subsequent spelling for *Brachypophlaeus* Fairmaire, 1897.

*Bradytes*; Dejean, 1834: 182; published before 1931 without a description, a definition, or an indication.

*Bucerus*; Dejean, 1834: 203; published before 1931 without a description, a definition, or an indication.

*Bysacnus*; Fairmaire, 1897f: 124; incorrect subsequent spelling for *Byzacnus* Pascoe, 1866.

*Cacloplesia*; Pic, 1935: 97; incorrect subsequent spelling for *Cacoplesia* Fairmaire, 1898.

*Calliosis*; Deyrolle, 1867: 81; original spelling of *Calosis*, corrected in the same work.

*Calobomon*; Reitter, 1900c: 93; incorrect subsequent spelling for *Calobamon* Kraatz, 1865.

*Calobosca*; Champion, 1895b: 170; incorrect subsequent spelling for *Calabosca* Fairmaire, 1894.

*Calymmaphorus*; Solier, 1841a: 209, 245; incorrect original spelling for *Calymmophorus* Solier, 1841.

*Calyptosis*; Maseul, 1857: 111; incorrect original spelling for *Calyptopsis* Solier, 1835.

*Camariocropteron*; Gebien, 1942a: 332; incorrect subsequent spelling for *Camariocropterum* Pic, 1920.

*Camarioides*; Seidlitz, 1900: 277; incorrect original spelling for *Camariodes* Fairmaire, 1869.

*Cameria*; Laporte, 1840: 231; incorrect subsequent spelling for *Camaria* Lepeletier & Audinet-Serville, 1828.

*Campanotiphilus*; Carter, 1926: 148; incorrect subsequent spelling for *Camponotiphilus* Lea, 1914.

*Caragonia*; Wollaston, 1861: 206; first published as a synonym and not treated before 1961 as an available name and adopted as the name of a taxon or treated as a senior homonym.

*Caribanoisis*; Nabozhenko et al., 2016d: 569 [header]; as pointed out by Bousquet et al. (2018: 136), this is an error by the publisher for *Caribanosis* Nabozhenko, Kirejtshuk, Merkl, Varela, Aalbu & Smith, 2016.

*Cataphronetes*; Imhoff, 1856: 239; incorrect subsequent spelling for *Cataphronetis* P.H. Lucas, 1846.

*Catonus*; Kaszab, 1968: 395; incorrect subsequent spelling for *Catomus* Allard, 1876.

*Centronipus*; Macquart, 1850: 188; incorrect subsequent spelling for *Centronopus* Solier, 1848.

*Ceracostira*; Pic, 1955: 185; incorrect subsequent spelling for *Coracostira* Fairmaire, 1899.

*Chardiochianalus*; Kaszab, 1940a: 202; alternative original spelling of *Cardiochianalus* Kaszab, 1940, rejected by Kaszab (1975a: 4).

*Charinotus*; Dejean, 1834: 206; published before 1931 without a description, a definition or an indication.

*Charistela*; Kolbe, 1897b: 620; incorrect subsequent spelling for *Caristela* Fairmaire, 1894.

*Charitotheca*; Agassiz, 1846: 78; proposed as an unjustified emendation for the unavailable name *Chariotheca* Dejean, 1834; published before 1931 without a description, a definition, or an indication.

*Chatanayas*; Anonymous in Commonwealth Institute of Entomology, 1960: 393; incorrect subsequent spelling for *Chatanayus* Ardoin, 1957.

*Chelenodus*; Berthold, 1827: 369; published before 1931 without a description, a definition, or an indication.

*Chemolamus*; Fairmaire, 1895b: 447, 448; incorrect subsequent spelling for *Chemolanus* Bates, 1879.

*Chilenodus*: Agassiz, 1846a: 35; proposed as a replacement name for the unavailable name *Chelenodus* Berthold, 1827; published before 1931 without a description, a definition, or an indication.

*Chiron*; Villa and Villa, 1833: 18; first published as a synonym and not treated before 1961 as an available name and adopted as the name of a taxon or treated as a senior homonym.

*Cholopus* Agassiz, 1846b: 83; proposed as a replacement name for the unavailable name *Cholipus* Dejean, 1834; published before 1931 without a description, a definition, or an indication.

*Chorodes*; Cazurro Ruiz, 1895: 730; incorrect subsequent spelling for *Chaerodes* White, 1846.

*Chremolamus*; Fairmaire, 1884d: 74; incorrect subsequent spelling for *Chemolanus* Bates, 1879.

*Chromomoea*; Carter, 1915a: 59; incorrect subsequent spelling for *Chromomaea* Pascoe, 1866.

*Cichillus*; Bertkau, 1890: 257; incorrect subsequent spelling for *Dichillus* Jacquelin du Val, 1860.

*Cilibe*; Dejean, 1836: 208; incorrect subsequent spelling for *Celibe* Boisduval, 1835.

*Clinocramon*; Péringuey, 1904: 297; incorrect subsequent spelling for *Clinocranion* Solier, 1843.

*Clinocranium*; Agassiz, 1846a: 39; incorrect subsequent spelling for *Clinocranion* Solier, 1843.

*Cnecozochara*; Ogloblin and Znojko, 1950: 57; incorrect subsequent spelling for *Cnecosochara* Reitter, 1913.

*Cnodalium*; Gray, 1831: pl. 80; incorrect subsequent spelling for *Cnodalon* Latreille, 1797.

*Cnodulon*; Fabricius, 1801a: xxi; incorrect subsequent spelling for *Cnodalon* Latreille, 1797.

*Cnudalon*; Latreille, 1816a: 306; incorrect subsequent spelling for *Cnodalon* Latreille, 1797.

*Coedius*; Blanchard, 1845: 13; incorrect original spelling of *Caedius* Blanchard, 1845.

*Coelecnemis*; Imhoff, 1856: 238; incorrect subsequent spelling for *Coelocnemis* Mannerheim, 1843.

*Colposcytis*; Reitter, 1897a: 298; incorrect subsequent spelling for *Colposcythis* Reitter, 1889.

*Colposphaena*; Koch, 1950b: 283; incorrect subsequent spelling for *Colposphena* Semenov, 1889.

*Comphonota*; Marseul, 1857: 112; incorrect subsequent spelling for *Camphonota* Solier, 1836.

*Coriogeton*; Pic, 1915e: 20; incorrect subsequent spelling for *Cyriogeton* Pascoe, 1871.

*Coronus*; Dejean, 1834: 192; published before 1931 without a description, a definition or an indication.

*Corypera*; Fauvel, 1904: 190; incorrect subsequent spelling for *Coripera* Pascoe, 1866.

*Coscinopter*; Allard, 1876a: 15; original spelling corrected to *Coscinoptilix* in the same work.

*Coscinoptilax*; Blackwelder, 1945: 542; incorrect subsequent spelling for *Coscinoptilix* Allard, 1876.

*Cossiphus*; Laporte, 1833b: 34; incorrect subsequent spelling for *Cossyphus* G.-A. Olivier, 1791.

*Costatostira*; Pic, 1954: 232; incorrect subsequent spelling for *Costatosora* Pic, 1934.

*Crirasida*; Gebien, 1937a: 726; incorrect subsequent spelling for *Cribrasida* Reitter, 1917.

*Cripticopsis*; Español, 1955: 19; incorrect subsequent spelling for *Crypticopsis* Antoine, 1945.

*Cripticus*; Español, 1955: 9; incorrect subsequent spelling for *Crypticus* Latreille, 1816.

*Crypsinon*: Fairmaire, 1894f: 395; incorrect subsequent spelling for *Crypsinous* Fairmaire, 1891.

*Cryptochyle*; Latreille, 1829a: 7; incorrect subsequent spelling for *Cryptochile* Latreille, 1828.

*Ctenoposomus*; Reitter, 1906b: 135; alternative original spelling of *Ctenioposomus* Reitter, 1906, rejected by Bousquet et al. (2015: 140).

*Curismosphena*; Gebien, 1921b: 3; incorrect subsequent spelling for *Curimosphena* Gebien, 1920.

*Cymathotes*; Blanchard, 1845: 33; incorrect subsequent spelling for *Cymatothes* Dejean, 1834.

*Cyphagenia*; Laporte, 1840: 191; incorrect subsequent spelling for *Cyphogenia* Solier, 1837.

*Cyphoglossa*; Silbermann, 1838: 32; incorrect subsequent spelling for *Cryptoglossa* Solier, 1837.

*Cypobiestes*; Gebien, 1942a: 756; incorrect subsequent spelling for *Cybopiestes* Reitter, 1917.

*Deilognatha*; Sahlberg, 1903: 26; incorrect subsequent spelling for *Dailognatha* Steven, 1828.

*Delanurops*; Iablokoff-Khnzorian, 1964: 309; incorrect subsequent spelling for *Delonurops* Reitter, 1922.

*Dendronomus*; Dejean, 1834: 205; published before 1931 without a description, a definition or an indication.

*Derilis*; Motschulsky, 1872: 27; alternative original spelling of *Deriles* Motschulsky, 1872, herein rejected.

*Diaphanidius*; Semenov-Tjan-Shansky and Bogatchev, 1940: 208; incorrect subsequent spelling for *Diaphanidus* Reitter, 1900.

*Diatopsis*; Laporte, 1840: 242; incorrect subsequent spelling for *Dietopsis* Solier, 1835.

*Dichroma*; Gemminger in Gemminger and Harold, 1870: 1916; incorrect subsequent spelling for *Dichromma* Mulsant & Rey, 1855.

*Dichtrethus*; Péringuey, 1892a: 52; incorrect subsequent spelling for *Distretus* Haag-Rutenberg, 1871.

*Dimonus*; Reitter, 1914c: 389; incorrect subsequent spelling for *Dymonus* Solier, 1843.

*Diodoeus*; Kaszab, 1955: 477; incorrect subsequent spelling for *Dioedus* J.L. LeConte, 1862.

*Diodontus*; Laporte, 1840: 185; incorrect subsequent spelling for *Diodontes* Solier, 1834.

*Diphyrhynchus*; Fairmaire, 1849: 445; incorrect original spelling for *Diphyrrhynchus* Fairmaire, 1849.

*Diplocyrthus*; Escalera, 1914: 355; incorrect original spelling for *Diplocyrtus* Quedenfeldt, 1887.

*Dirosus*; Dallas, 1866: 470; incorrect original spelling for *Dirosis* Miller, 1858.

*Dissonus*; Marseul, 1863: 181; incorrect original spelling for *Dissonomus* Jacquelin du Val, 1861.

*Dolichopterus*; Gemminger in Gemminger and Harold, 1870: 2028; first published as a synonym and not treated before 1961 as an available name and adopted as the name of a taxon or treated as a senior homonym.

*Doryacus*; Gebien, 1938a: 73; incorrect subsequent spelling for *Doryagus* Pascoe, 1887.

*Dromeus*; Reitter, 1900c: 186; first published as a synonym and not treated before 1961 as an available name and adopted as the name of a taxon or treated as a senior homonym.

*Dyla*; Marseul, 1857: 116; incorrect subsequent spelling for *Dila* Fischer von Waldheim, 1844.

*Dzungaropterocoma*; Löbl et al., 2008b: 166; incorrect subsequent spelling for *Dzhungaropterocoma* Skopin, 1974.

*Ebertia*; Kaszab, 1970d: 429; incorrect subsequent spelling for *Ebertius* Jedlička, 1965.

*Ecoptostira*; Borchmann, 1936: 236; alternative original spelling of *Eccoptostira* Borchmann, 1936, herein rejected.

†*Edromus*; Haupt, 1950: 120; alternative original spelling of †*Eodromus* Haupt, 1950, herein rejected.

*Edrotoporus*; Champion, 1895b: 93; incorrect subsequent spelling for *Edrotopus* Haag-Rutenberg, 1877.

*Elanophorus*; Dahl, 1823: 41; incorrect subsequent spelling for *Elenophorus* Dejean, 1821; published in a work suppressed for the purposes of zoological nomenclature (ICZN 1964, Opinion 710).

*Eleutheris*; Dejean, 1834: 206; published before 1931 without a description, a definition or an indication.

*Ellidonius*; Gebien, 1937a: 743; incorrect subsequent spelling for *Ellidoneus* Wilke, 1922.

*Elodona*; Erichson, 1845b: 256; incorrect subsequent spelling for *Eledona* Latreille, 1797.

*Emallodera*; Burmeister, 1875: 467; incorrect subsequent spelling for *Emalodera* Blanchard, 1842.

*Emalodera*; Blanchard, 1842: pl. 13; incorrect original spelling, emended to *Emmallodera* Blanchard, 1842, under prevailing usage.

*Emmalodera*; Champion, 1895b: 46; incorrect subsequent spelling for *Emalodera* Blanchard, 1842.

*Emsipara*; Reitter, 1916b: 10; incorrect subsequent spelling for *Emypsara* Pascoe, 1866.

*Encyrtus*; Fairmaire, 18cix; incorrect subsequent spelling for *Eucyrtus* Lacordaire, 1859.

*Endomoderes*; Silbermann, 1838: 28; incorrect subsequent spelling for *Entomoderes* Solier, 1836.

*Endothyna*; Gebien, 1939: 470; incorrect subsequent spelling for *Endothina* Carter, 1924.

*Entelogonus*; Iablokoff-Khnzorian, 1964: 304; incorrect subsequent spelling for *Eutelogonus* Reitter, 1922.

*Enthriptera*; Reitter, 1893: 203; alternative original spelling of *Euthriptera* Reitter, 1893, herein rejected.

*Entomoderon*; Silbermann, 1838: 30; incorrect subsequent spelling for *Entomoderes* Solier, 1836.

*Eocoptostira*; Neave, 1950: 86; incorrect subsequent spelling for *Eccoptostira* Borchmann, 1936.

*Epaerops*; Champion, 1895b: 92; incorrect subsequent spelling for *Epairops* Fåhraeus, 1870.

*Epeirops*; Bertkau, 1876: 78; incorrect subsequent spelling for *Epairops* Fåhraeus, 1870.

*Ephicerus*; Macquart, 1850: 186; published before 1931 without a description, a definition or an indication.

*Epicamptus*; Dejean, 1834: 198; published before 1931 without a description, a definition or an indication.

*Epipedonata*; Solier, 1836: 342; alternative original spelling of *Epipedonota* Solier, 1836, rejected by Solier (1851: 157).

*Erganna*; Oustalet, 1898: 972; incorrect subsequent spelling for *Ergenna* Fairmaire, 1897.

*Eriodontes*; Gebien, 1937a: 546; incorrect subsequent spelling for *Erodiontes* Reitter, 1914

*Eschaptoporis*; Seidlitz, 1908: 336; incorrect subsequent spelling for *Eschatoporis* Blaisdell, 1906.

*Etaceta*; Ferrer and Delatour, 2007: 275; incorrect subsequent spelling for *Etazeta* Fairmaire, 1889.

*Eucirtus*; Schoenfeldt, 1897: 126; incorrect subsequent spelling for *Eucyrtus* Lacordaire, 1859.

*Eudustomus* Kolbe, 1889: 127; incorrect subsequent spelling for *Endustomus* Brême, 1842.

*Euganodia*; Seidlitz, 1900: 277; incorrect subsequent spelling for *Enganodia* Fairmaire, 1898.

*Eulimenes*; Lacordaire, 1859: 467; first published as a synonym and not treated before 1961 as an available name and adopted as the name of a taxon or treated as a senior homonym.

*Eunotus*; Dejean, 1834: 198; published before 1931 without a description, a definition or an indication.

*Euoplus*; Bertkau, 1879: 502; incorrect subsequent spelling for *Evoplus* J.L. LeConte, 1866.

*Euplopus*; Ragusa, 1898: 127; incorrect subsequent spelling for *Enoplopus* Solier, 1848.

*Eurhyzodina*; Bremer and Lillig, 2014: 145; incorrect subsequent spelling for *Eurhysodina* Wasmann, 1921.

*Eurichora*; G.-A. Olivier, 1795: 2; incorrect subsequent spelling for *Eurychora* Thunberg, 1789.

*Euripimelia*; Chernei, 2005: 104; incorrect subsequent spelling for *Eurypimelia* Reitter, 1915.

*Eurygonus*; Lacordaire, 1859a: 217; incorrect subsequent spelling for *Eurygona* Laporte, 1840.

*Eusemostene*; Gebien, 1940: 764; published after 1930 without a type species designation (see Bousquet et al. 2018: 224).

*Eutychus* Péringuey, 1908: 405; incorrect subsequent spelling of *Eutichus* Haag-Rutenberg, 1875.

*Eutypodera*; Pic, 1907b: 148; incorrect subsequent spelling for *Entypodera* Gerstaecker, 1871.

*Evaniosamus*; Laporte, 1840: 192; incorrect subsequent spelling for *Evaniosomus* Guérin-Méneville, 1834.

*Evrychora*; Thunberg, 1789: 9; incorrect original spelling for *Eurychora* Thunberg, 1789.

*Evtelus*; Fåhraeus, 1870: 304; incorrect original spelling for *Eutelus* Solier, 1843.

*Exangelutus*; Kulzer, 1964: 274; incorrect subsequent spelling for *Exangeltus* Blackburn, 1897.

*Excavatoprostenus*; Pic, 1944: 8; published after 1930 without a type species designation (see Bousquet et al. 2015: 139).

*Falsostenochirus*; Gebien, 1943: 924; incorrect subsequent spelling for *Falsastenochirus* Pic, 1938.

*Fatuellus*; Gistel, 1850: 409; proposed as a replacement name for the unavailable name *Lateranus* Sturm, 1843; published before 1931 without a description, a definition, or an indication.

*Galymmaphorus*; Solier, 1841a: 247; alternative original spelling of *Calymmophorus* Solier, 1841, herein rejected.

*Gampsia*; Gistel, 1848a: 126; incorrect subsequent spelling for *Campsia* Lepeletier & Audinet-Serville, 1828.

*Gasthrhaema*; Reitter, 1906b: 118; incorrect subsequent spelling for *Gastrhaema* Jacquelin du Val, 1863.

*Gentianidis*; Gemminger in Gemminger and Harold, 1870: 2028; incorrect subsequent spelling for *Gentinadis* Laporte, 1840.

*Glyptophrinus*; Lesne, 1922: 688; incorrect subsequent spelling for *Glyptophrynus* Fairmaire, 1899.

*Glyptoteryx*; Koch, 1956a: 216; incorrect subsequent spelling for *Glyptopteryx* Gebien, 1910.

*Gnata*; Dahl, 1823: 41; published in a work suppressed for the purposes of zoological nomenclature (ICZN 1964, Opinion 710).

*Gonyodera*; Perty, 1832: 63; alternative original spelling of *Goniadera*, rejected by Perty (1833: 14).

*Gymnogathus*; Kaszab, 1978: 52; incorrect subsequent spelling for *Gymnognathus* Solier, 1851.

*Hadrobates*; Seidlitz, 1905: 275; incorrect subsequent spelling for *Habrobates* Semenov, 1903.

*Halamobia*; Reitter, 1911: 329; incorrect subsequent spelling for *Halammobia* Semenov, 1901.

*Harotoma*; Kulzer 1951b: 563; incorrect subsequent spelling for *Horatoma* Solier, 1841.

*Harpiscius*; Reitter, 1914b: 370; incorrect subsequent spelling for *Herpiscius* Solier, 1838.

*Heiliofugus*; Laporte, 1840: 200; incorrect subsequent spelling for *Heliofugus* Guérin-Méneville, 1831.

*Helaeus*; Kirby, 1819b: 467; incorrect subsequent spelling for *Helea* Latreille, 1804.

*Heleus*; Latreille, 1817: 261; incorrect subsequent spelling for *Helea* Latreille, 1804.

*Heliopathes*; Mulsant, 1854: 157; incorrect subsequent spelling for *Heliopates* Dejean, 1834.

*Heliophugus*; Lacordaire, 1859b: 744; incorrect subsequent spelling for *Heliofugus* Guérin-Méneville, 1831.

*Heliosthraema*; Reitter, 1890a: 34; incorrect original spelling, emended to *Heliostrhaema* Reitter, 1890 under prevailing usage.

*Helisteres* Gemminger in Gemminger and Harold, 1870: 2008; incorrect subsequent spelling for *Heliosteres* Hope, 1841.

*Hemicyclys*; Imhoff, 1856: 243; incorrect subsequent spelling for *Hemicyclus* Westwood, 1841.

*Hemisphaeresmia*; Koch, 1944b: 157; published after 1930 without a type species designation.

*Hemitrichesthes*; Gebien, 1911b: 620; incorrect subsequent spelling for *Hemitrichestes* Reitter, 1904.

*Hemosodes*; Seidlitz, 1909: 298; incorrect subsequent spelling for *Hemasodes* Casey, 1907.

*Hepadrinus*; Luederwaldt, 1929: 48; incorrect subsequent spelling for *Opatrinus* Dejean, 1821.

*Heteroblaps*; Kolbe, 1928: 202; published before 1931 without a description, a definition, or an indication.

*Hexagonocheilus*; Solier, 1851: 168; incorrect original spelling for *Hexagonochilus* Solier, 1851.

*Heydyphanes*; Kraatz in Heyden and Kraatz, 1882: 332; incorrect subsequent spelling for *Hedyphanes* Fischer von Waldheim, 1820.

*Hipponomus*; Iablokoff-Khnzorian, 1964: 303; incorrect subsequent spelling for *Hipponome* Laporte, 1840.

*Hipsosoma*; Marseul, 1887: 307; incorrect subsequent spelling for *Hypsosoma* Ménétriés, 1854.

*Holania*; Fauvel, 1904: 186; incorrect subsequent spelling for *Holaniara* Fairmaire, 1871.

*Hologlyptus*; J.L. LeConte, 1866a: 59; incorrect original spelling for *Ologlyptus* Lacordaire, 1858.

*Homalopus*; Solier, 1836: 78; name not used as valid when proposed.

*Homoeotrysis*; Haag-Rutenberg, 1879b: 136; incorrect subsequent spelling for *Homotrysis* Pascoe, 1866.

*Hovamarygmus*; Gebien, 1943: 925; incorrect subsequent spelling for *Hovarygmus* Fairmaire, 1898.

*Hybraenia*; Pascoe 1866c: 497; incorrect subsequent spelling for *Hybrenia* Pascoe, 1866.

*Hylobates*; Dejean, 1834: 204; published before 1931 without a description, a definition, or an indication.

*Hylocurus*; Dejean, 1834: 203; published before 1931 without a description, a definition, or an indication.

*Hymenophorus*; Mulsant, 1852: 68; original spelling of *Hymenorus* Mulsant, 1852, corrected in the same work.

*Hyonthis*; Reitter, 1897a: 297; incorrect subsequent spelling for *Hionthis* Miller, 1861.

*Hypophloeus*; Panzer, 1795: 339; incorrect subsequent spelling for *Hypophlaeus* Fabricius, 1790.

*Hypsoderes*; Dejean, 1834: 195; published before 1931 without a description, a definition, or an indication.

*Iophon*; Champion, 1895a: 224; incorrect original spelling for *Jophon* Champion, 1895.

*Iphicerus*; Dejean, 1834: 203; published before 1931 without a description, a definition, or an indication.

*Iphtinus*; Reiche, 1854: 16; incorrect original spelling for *Iphthinus* Dejean, 1834.

*Ipitragus*; Macquart, 1850: 186; incorrect original spelling for *Epitragus* Latreille, 1802.

*Isomera*; Fairmaire and Coquerel, 1866: 46; incorrect subsequent spelling for *Isomira* Mulsant, 1856.

*Isophon*; Doyen et al., 1990: 235; incorrect subsequent spelling for *Jophon* Champion, 1895.

*Jintainum*; Iwan and Löbl, 2008: 267; incorrect subsequent spelling for *Jintaium* Ren, 1999.

*Lachnoderus*; Gebien, 1910b: 301; incorrect subsequent spelling for *Lachnoderes* Mulsant & Rey, 1859.

*Lachnogyia*; Reitter, 1904: 34; incorrect subsequent spelling for *Lachnogya* Ménétriés, 1849.

*Lachriomus*; Solsky, 1881: 50; incorrect subsequent spelling for *Lechriomus* Morawitz, 1865.

*Lasiotata*; Laporte, 1840: 181; incorrect subsequent spelling for *Lasiostola* Dejean, 1834.

*Lathelicus*; Heyden et al., 1883: 133; incorrect subsequent spelling for *Latheticus* C.O. Waterhouse, 1880.

*Lateranus*; Sturm, 1843: 147; published before 1931 without a description, a definition, or an indication.

*Leaena*; Dahl, 1823: 41; incorrect subsequent spelling for *Laena* Dejean, 1821; published in a work suppressed for the purposes of zoological nomenclature (ICZN 1964, Opinion 710).

*Leichrodes*; Pic, 1934d: 84; incorrect subsequent spelling for *Leiochrodes* Westwood, 1883.

*Leiochbrota*; Kirby, 1885a: 90; incorrect subsequent spelling for *Leiochrota* Westwood, 1883.

*Leiochritina*; Neervoort van de Poll, 1886: 34; incorrect subsequent spelling for *Leiochrotina* Westwood, 1883.

*Leiochrodomorphus*; Kaszab, 1946a: 31; incorrect subsequent spelling for *Leichrodomorphus* Pic, 1921.

*Leporina*; Reitter, 1920a: 18; first published as a synonym and not treated before 1961 as an available name and adopted as the name of a taxon or treated as a senior homonym.

*Leptynoderus*; Curtis, 1844: 221; incorrect subsequent spelling for *Leptynoderes* Solier, 1838.

*Leucolephus*; Medvedev, 1973: 644; incorrect subsequent spelling for *Leucolaephus* P.H. Lucas, 1859.

*Leucoloephus*; P.H. Lucas, 1859: xxii; incorrect original spelling of *Leucolaephus* P.H. Lucas, 1859.

*Liochrinus*; Fairmaire, 1893b: 25; incorrect subsequent spelling for *Leiochrinus* Westwood, 1883.

*Listronychus*; Laporte, 1840: 244; incorrect subsequent spelling for *Lystronychus* Latreille, 1829.

*Loboderus*; Gemminger, 1870: 1925; incorrect subsequent spelling for *Lobodera* Mulsant & Rey, 1859.

*Lobophilus*; Pic, 1911a: 183; incorrect subsequent spelling for *Lophophyllus* Fairmaire, 1887.

*Longicerenopus*; Berry, 1975: 929; published after 1930 without a type species designation (see Bousquet et al. 2018: 181).

*Lutelus*; Solier, 1843: 4; alternative original spelling of *Eutelus* Solier, 1843, herein rejected.

*Lyprocaulus*; Pic, 1934d: 84; incorrect subsequent spelling for *Leprocaulus* Fairmaire, 1896.

*Lyprochelida*; Gebien, 1904b: 20; incorrect subsequent spelling for *Lyprochelyda* Fairmaire, 1899.

*Lyproehelyda*; Gebien, 1921b: 238; incorrect subsequent spelling for *Lyprochelyda* Fairmaire, 1899.

*Lyprops*; Hope, 1841: 133; incorrect subsequent spelling for *Luprops* Hope, 1833.

*Lystronichus*; Latreille, 1829a: 41; incorrect original spelling for *Lystronychus* Latreille, 1829.

*Machlopis*; Reitter, 1900c: 191; incorrect subsequent spelling for *Machlopsis* Pomel, 1871.

*Macrophthalmus*; Lacordaire, 1859b: 732; incorrect subsequent spelling for *Macrophtalmus* Montrouzier, 1855.

*Macrotis*; Dejean, 1834: 186; published before 1931 without a description, a definition, or an indication.

*Marcuzzichoton*; Ferrer and Moraguès, 2001: 500, 517; published after 1930 without a type species designation (see Kamiński et al. 2019c: 361).

*Medaris*; Kolbe, 1898: 598; incorrect subsequent spelling for *Mederis* Motschulsky, 1872.

*Megagrius*; Laporte, 1840: 183; incorrect subsequent spelling for *Megagenius* Solier, 1835.

*Megliphus*; Motschulsky, 1872: 41; alternative original spelling of *Meglyphus* Motschulsky, 1872, herein rejected.

*Meladiesa*; Gebien, 1937a: 807; incorrect subsequent spelling for *Meladiesia* Reitter, 1909.

*Melanchrus*; Dejean, 1834: 185; published before 1931 without a description, a definition or an indication.

*Melanchrus*; Lillig and Pavlíček, 2003: 62; incorrect subsequent spelling for *Melancrus* Reiche & Saulcy, 1857.

*Metopocerus*; Dejean, 1834: 190; published before 1931 without a description, a definition, or an indication.

*Metriopa*; Hope, 1841: 118; incorrect subsequent spelling for *Metriopus* Solier, 1835.

*Micispa*; Motschulsky, 1858b: 189; incorrect subsequent spelling for *Micipsa* P.H. Lucas, 1855.

*Micrantherus*; Bertkau, 1876: 78; incorrect subsequent spelling for *Micrantereus* Solier, 1848.

*Micretyche*; Carter, 1926: 134; incorrect subsequent spelling for *Micrectyche* Bates, 1873.

*Micreuphloeus*; Gebien, 1911a: 526; incorrect subsequent spelling for *Micreuphlaeus* Fairmaire, 1897.

*Microblattelus*; Bremer, 2014: 181; incorrect subsequent spelling for *Microblattellus* Ferrer, 2006.

*Microperithrix*; Pierre, 1972: 951; name published after 1930 without a description, a definition or a bibliographic reference to such a published statement.

*Microperitrix*; Reymond, 1954: 45; name published after 1930 without a description, a definition or a bibliographic reference to such a published statement.

*Microps*; Dahl, 1823: 46; published in a work suppressed for the purposes of zoological nomenclature (ICZN 1964, Opinion 710).

*Microsomira*; Anonymous, 1931: 204; incorrect subsequent spelling for *Micrisomira* Pic, 1930.

*Miladion*; Reitter, 1887a: 385; alternative original spelling of *Myladion* Reitter, 1887, rejected by Reitter (1896b: 166).

*Milaris*; Motschulsky, 1872: 23; incorrect subsequent spelling for *Mylaris* Pallas, 1781.

*Mimoborchmannia*; Borchmann, 1936: 490; incorrect subsequent spelling for *Mimoborchmania* Pic, 1934.

*Minantereus*; Desmarest, 1860: 168; incorrect subsequent spelling for *Micrantereus* Solier, 1848.

*Mitrogenius*; Solier, 1836: 330; alternative original spelling of *Mitragenius* Solier, 1836, rejected by Solier (1851: 152).

*Monogolesthes*; Seidlitz, 1906: 268; incorrect subsequent spelling for *Mongolesthes* Reitter, 1904.

*Morocaula*; Sharp, 1899: 144; incorrect subsequent spelling for *Morocaulus* Fairmaire, 1899.

*Mosostena*; Marseul, 1863: 170; incorrect subsequent spelling for *Mesostena* Eschscholtz, 1831.

*Myaladion*; Kaszab, 1960a: 161; incorrect subsequent spelling for *Myladion* Reitter, 1887.

*Myaldion*; Kaszab, 1960a: 161; incorrect subsequent spelling for *Myladion* Reitter, 1887.

*Mycetocharus*; Stephens, 1832b: 27; incorrect subsequent spelling for *Mycetochares* Latreille, 1829.

*Mycetophylla*; Sturm, 1843: 166; incorrect subsequent spelling for *Mycetophila* Gyllenhal, 1810.

*Myria*; Gemminger in Gemminger and Harold, 1870: 1963; first published as a synonym and not treated before 1961 as an available name and adopted as the name of a taxon or treated as a senior homonym.

*Myrmecixenus*; Erichson, 1844: 287; incorrect subsequent spelling for *Myrmechixenus* Chevrolat, 1835.

*Neoandrosus*; Pic, 1921: 12; alternative original spelling of *Neandrosus* Pic, 1921, herein rejected.

*Nesiotaurus*; Fairmaire, 1897b: 385; incorrect subsequent spelling for *Nesotaurus* Fairmaire, 1896.

*Nilion*; Latreille, 1802: 179; incorrect original spelling of *Nilio* Latreille, 1802.

*Nocturna*; Voet, 1806: 81; published in a work that did not include consistent application of binominal nomenclature.

*Nodosogilium*; Pic, 1951: 12; alternative original spelling of *Nodosogylium* Pic, 1951, herein rejected.

*Nonpenicillus*; Ren, Wang and Yu, 2000: 50; published after 1930 without a type species designation.

*Notha*; Dejean, 1834: 182; published before 1931 without a description, a definition or an indication.

*Notiolosthus*; Motschulsky, 1872: 34; alternative original spelling of *Notiolesthus*, herein rejected.

*Notoprateus*; Gebien, 1941: 821; incorrect subsequent spelling for *Notoprataeus* Carter, 1924.

*Nyctipathes*; Marseul, 1857: 116; incorrect subsequent spelling for *Nyctipates* Gebler, 1841.

*Nystagmus*; Gistel, 1848a: 125; proposed as a replacement name for the unavailable name *Bucerus* Dejean, 1834; published before 1931 without a description, a definition or an indication.

*Odontomoplus*; Ragusa, 1898: 200; incorrect subsequent spelling for *Odontomophlus* Seidlitz, 1896.

*Oedemetus*; Bertkau, 1876: 79; incorrect subsequent spelling for *Oedemutes* Pascoe, 1860.

*Ogcoosoma*; Westwood, 1844: 227; incorrect subsequent spelling for *Oncosoma* Westwood, 1843.

*Ogcosoma*; Westwood, 1843: 121; incorrect original spelling, emended to *Oncosoma* Westwood, 1843 under prevailing usage.

*Ohionthis*; Reitter, 1900c: 89, 139; incorrect subsequent spelling for *Ohyonthis* Reitter, 1898.

*Oleroscelis*; Gemminger in Gemminger and Harold, 1870: 1812; incorrect subsequent spelling for *Oteroscelis* Solier, 1835.

*Olisthoena*; Fairmaire, 1849: 451; incorrect subsequent spelling for *Olisthaena* Erichson, 1842.

*Omalois*; Allard, 1876a: 4; incorrect original spelling of *Omaleis* Allard, 1876.

*Omalus*; Allard, 1876a: 4; original spelling of *Omalois* Allard, 1876, corrected in the same work.

*Omocrates*; Mulsant, 1854: 150; original spelling of *Olocrates*, corrected in the same work.

*Onymachris*; Champion, 1895b: 9; incorrect subsequent spelling for *Onymacris* Allard, 1885.

*Opatrinops*; Reitter, 1904: 73; first published as a synonym and not treated before 1961 as an available name and adopted as the name of a taxon or treated as a senior homonym.

*Ophtalmosis*; Deyrolle, 1867: 229; original spelling of *Ophthalmosis*, corrected in the same work.

*Opiestus*; Chevrolat, 1833b: 30; alternative original spelling of *Oopiestus*, rejected by Chevrolat (1847a: 118).

*Opisidus*; Masters, 1872: 182; incorrect subsequent spelling for *Ospidus* Pascoe, 1866.

*Oplochirus*; Bertkau, 1876: 78; incorrect subsequent spelling for *Oplocheirus* Klug, 1835.

*Oplomerus*; Dejean, 1834: 206; published before 1931 without a description, a definition, or an indication.

*Opsidus*; Masters, 1887: 328; incorrect subsequent spelling for *Ospidus* Pascoe, 1866.

*Orchrotus*; Cazurro Ruiz, 1894a: 63; incorrect subsequent spelling for *Oochrotus* Lucas, 1852.

*Orientacara*; Penrith, 1986: 294; incorrect subsequent spelling for *Orientocara* Koch, 1952.

*Orthogonoderus*; Germain, 1855: 402, 403; incorrect subsequent spelling for *Orthogonoderes* Solier, 1841.

*Oxipistoma*; Koch, 1940a: 259; alternative original spelling of *Oxypistoma*, also proposed after 1930 without type species designation.

*Pachynoscelis*; Heyden, 1882: 144; published without a description, a definition, or an indication.

*Paecilesthus*; Dejean, 1834: 207; incorrect original spelling of *Poecilesthus* Dejean, 1834.

*Paleosclerum*; Nabozhenko, 2019: 6; incorrect subsequent spelling for *Palaeosclerum* Nabozhenko & Kirejtshuk, 2017.

*Paracupezus*; Riley, 1923: 129; incorrect subsequent spelling for *Pareupezus* Kolbe, 1889.

*Parapiophorus*; Nabozhenko, 2019: 8; incorrect subsequent spelling for *Paropiophorus* Haupt, 1950.

*Parenicmosoma*; Ardoin, 1959a: 61; published after 1930 without a type species designation.

*Pectenepitragus*; Gebien, 1937a: 570; incorrect subsequent spelling for *Pectinepitragus* Pic, 1927.

*Pectophegoneus*; Anonymous in Staff of the Zoological Society of London, 1972: 507; incorrect subsequent spelling for *Pectphegoneus* Freude, 1968.

*Pedirus*; Doyen et al., 1990: 237; incorrect subsequent spelling for *Pediris* Motschulsky, 1872.

*Pelecipalpus*; Kaszab, 1982b: 48; incorrect subsequent spelling for *Pelecypalpus* Hinton, 1947.

*Penthapyllus*; Ragusa, 1898: 123; incorrect subsequent spelling for *Pentaphyllus* Dejean, 1821.

*Penticinus*; Reitter, 1896b: 171; alternative original spelling of *Penthicinus* Reitter, 1896, rejected by Reitter (1904: 135, 170).

*Penticus*; Medvedev and Iwan, 2007: 613; incorrect subsequent spelling for *Penthicus* Faldermann, 1836.

*Periclytus*; Bertkau, 1876: 78; incorrect subsequent spelling for *Proselytus* Fåhraeus, 1870.

*Periseptus*; Fairmaire, 1887a: 176; incorrect subsequent spelling for *Peristeptus* Haag-Rutenberg, 1875.

*Petrobius*; Brullé, 1832: 202; incorrect subsequent spelling for *Gnaptor* Brullé, 1831.

*Phalera*; Dejean, 1821: 68; incorrect subsequent spelling for *Phaleria* Latreille, 1802.

*Phanerentoma*; Lacordaire, 1859a: 195; incorrect subsequent spelling for *Phanerotoma* Solier, 1843.

*Philammus*; Escalera 1914: 339; incorrect subsequent spelling for *Philhammus* Fairmaire, 1871.

*Philax*; Dejean, 1834: 192; incorrect subsequent spelling for *Phylax* Brullé, 1832.

*Philethus*; Macquart, 1850: 182; incorrect subsequent spelling for *Phyletes* Redtenbacher, 1845.

*Philoscotus*; Dejean, 1834: 186; published before 1931 without a description, a definition or an indication.

*Phlaegmatus*; Dejean, 1834: 208; published before 1931 without a description, a definition or an indication.

*Phloeotribon*; Gerstaecker, 1866: 427; incorrect subsequent spelling for *Phaeotribon* Kraatz, 1865.

*Phrepates*; Solier, 1834: 488; incorrect subsequent spelling for *Phrenapates* Gray, 1831.

*Phylacoprosodes*; Schuster, 1934: 75; replacement name for *Aulonoscelis* Reitter, 1909, which is not an available name.

*Phylas*; Berthold, 1827: 368; incorrect subsequent spelling for *Phylan* Sturm, 1826.

*Phylethus*; Chevrolat, 1847b: 57; incorrect subsequent spelling for *Phyletes* Redtenbacher, 1845.

*Phyletus*; Marseul, 1863: 184; incorrect subsequent spelling for *Phyletes* Redtenbacher, 1845.

*Phylhammus*; Español, 1963b: 188; incorrect subsequent spelling for *Philhammus* Fairmaire, 1871.

*Phymatesthes*; Gebien, 1928: 189, 191; incorrect subsequent spelling for *Phymatestes* Pascoe, 1866.

*Phymathodes*; Blanchard, 1845: 39; incorrect subsequent spelling for *Phymatodes* Dejean, 1834.

*Phymatisoma*; Laporte and Brullé, 1831: 332, 408; incorrect original spelling, emended to *Phymatosoma* Laporte & Brullé, 1831 under prevailing usage.

*Phythora*; Marquet, 1897: 162; incorrect subsequent spelling for *Phtora* Germar, 1836.

*Phytophilus*; Guérin-Méneville, 1838: 99; incorrect subsequent spelling for *Phitophilus* Guérin-Méneville, 1831.

*Phyxelius*; Philippi, 1864: 348; first published as a synonym and not treated before 1961 as an available name and adopted as the name of a taxon or treated as a senior homonym.

*Piazomera*; Gebien, 1910a: 154; incorrect subsequent spelling for *Piesomera* Solier, 1843.

*Piezomera*; Gemminger in Gemminger and Harold, 1870: 1898; incorrect subsequent spelling for *Piesomera* Solier, 1843.

*Piliobola*; Hope, 1841: 112; published before 1931 without a description, a definition, or an indication.

*Pimelionotus*; Ardoin, 1962b: 969; published after 1930 without a type species designation.

*Pineta*; Desmarest, 1860: 159; incorrect subsequent spelling for *Peneta* Lacordaire, 1859.

*Plaesia*; Fairmaire, 1875: 43; incorrect subsequent spelling for *Plesia* Klug, 1833.

*Platomodes*; Dallas, 1866: 473; incorrect subsequent spelling for *Platamodes* Ménétriés, 1849.

*Platysum*; Mulsant and Rey, 1859a: 73, 81; original spelling of *Platynosum*, corrected in the same work.

*Plectrascelis*; Silbermann, 1838: 28; incorrect subsequent spelling for *Psectrascelis* Solier, 1836.

*Podhamala*; Motschulsky, 1860c: 535; incorrect subsequent spelling for *Podhomala* Solier, 1836.

*Polpagenia*; Laporte, 1840: 183; incorrect subsequent spelling for *Polpogenia* Solier, 1836.

*Polycoelagastridion*; Kaszab, 1942: 14; incorrect subsequent spelling for *Polycoelogastridion* Reichardt, 1936.

*Polycoelogastridium*; Gebien, 1938a: 428; incorrect subsequent spelling for *Polycoelogastridion* Reichardt, 1936.

*Porphyrrhyba*; Champion, 1895b: 195; incorrect subsequent spelling for *Porphyrhyba* Fairmaire, 1877.

*Porphyruba*; Bertkau, 1890: 260; incorrect subsequent spelling for *Porphyrhyba* Fairmaire, 1877.

*Porphyryba*; Fairmaire, 1894b: 144; incorrect subsequent spelling for *Porphyrhyba* Fairmaire, 1877.

*Prachoma*; Laporte, 1840: 195; incorrect subsequent spelling for *Prochoma* Solier, 1835.

*Praosis*; Laporte, 1840: 186; incorrect subsequent spelling for *Praocis* Eschscholtz, 1829.

*Praygena*; Bertkau, 1876: 78; incorrect subsequent spelling for *Praeugena* Laporte, 1840.

*Priopus*; Hope, 1841: 73; alternative original spelling of *Prioscelis*, rejected by Westwood (1844: 211).

*Procris*; Curtis, 1844: 221; incorrect subsequent spelling for *Praocis* Eschscholtz, 1829.

*Prodhomala*; Marseul, 1857: 112; incorrect subsequent spelling for *Podhomala* Solier, 1836.

*Proplatamodes*; Kaszab, 1960a: 16; first published as a synonym and not treated before 1961 as an available name and adopted as the name of a taxon or treated as a senior homonym.

*Prorythinota*; Ferrer et al., 2016: 160; incorrect subsequent spelling for *Prorhytinota* Koch, 1943, an unavailable name.

*Prothraustocolus*; Kaszab, 1979b: 278; incorrect subsequent spelling for *Prothraustocola* Kaszab, 1957.

*Psammeticus*; Guérin-Méneville, 1831b: pl. 28bis; incorrect subsequent spelling for *Psammetichus* Latreille, 1828.

*Psammodius*; Berthold, 1827: 366; incorrect subsequent spelling for *Psammodes* W. Kirby, 1819.

*Psaryphys*; Cazurro Ruiz, 1895: 498; incorrect subsequent spelling for *Psaryphis* Erichson, 1843.

*Pseudammobius*; Franz, 1996: 71; new nominal genus published in a combined description with a single included new nominal species not marked by “gen. nov., sp. nov.” or an equivalent expression (Löbl et al. 2008a: 45).

*Pseudelops*; Fauvel, 1904: 190; incorrect subsequent spelling for *Pseudhelops* Guérin-Méneville, 1841.

*Pseudemallus*; Edwards and Vevers, 1975: 281; incorrect subsequent spelling for *Pseudemmallus* Koch, 1956.

*Pseudoammobius*; Franz, 1996: 131; new nominal genus published in a combined description with a single included new nominal species not marked by “gen. nov., sp. nov.” or an equivalent expression (see Löbl et al. 2008a: 45).

*Pseudobax*; Gebien, 1942a: 308; incorrect subsequent spelling for *Pseudabax* Kraatz, 1880.

*Pseudocylibe*; Kaszab, 1982b: 291; alternative original spelling of *Pseudocylibe* Kaszab, 1982, herein rejected.

*Pseudocyrtus*; Pic 1916: 14; alternative original spelling of *Pseudeucyrtus* Pic, 1916, herein rejected.

*Pseudoderilis*; Gebien, 1941: 343; incorrect subsequent spelling for *Pseudoderiles* Gebien, 1928.

*Pseudohelops*; Cazurro Ruiz, 1895: 506; incorrect subsequent spelling for *Pseudhelops* Guérin-Méneville, 1841.

*Pseudonantes*; Pic, 1925b: 7; incorrect subsequent spelling for *Pseudonautes* Fairmaire, 1892.

*Pseudostena*; Gemminger in Gemminger and Harold, 1870: 1958; incorrect subsequent spelling for *Pseudostene* Wollaston, 1861.

*Pseudotrichoplatycelis*; Kaszab, 1960a: 83; alternative original spelling of *Pseudotrichoplatyscelis* Kaszab, 1960, herein rejected.

*Pseudotrichoplatynoscelis*; Kaszab, 1960a: 82; alternative original spelling of *Pseudotrichoplatyscelis* Kaszab, 1960, herein rejected.

*Psilioloba*; Mulsant and Rey, 1853b: 20; incorrect subsequent spelling of *Pilioloba* Erichson in Agassiz, 1846.

*Pyganisia*; Hope, 1841: 133; incorrect subsequent spelling for *Pyanisia* Laporte, 1840.

*Raibosceles*; Allard, 1876a: 5; incorrect original spelling for *Raiboscelis* Allard, 1876.

*Reichardtia*; Kaszab, 1982c: 79; incorrect subsequent spelling for *Reichardtiella* Kaszab, 1942.

*Reiterella*; Seidlitz, 1905: 276; incorrect subsequent spelling for *Reitterella* Semenov, 1891.

*Rhamnatodes* Champion, 1895b: 20; incorrect subsequent spelling for *Rhammatodes* Haag-Rutenberg, 1876.

*Rhicnodes*; Gebien, 1941: 817; incorrect subsequent spelling for *Rhicnodus* Fairmaire, 1892.

*Rhytimorpha*; Koch, 1943: 888; alternative original spelling of *Rhydimorpha* Koch, 1943, herein rejected.

*Rophobas*; Motschulsky, 1872: 36; alternative original spelling of *Rhophobas* Motschulsky, 1872, herein rejected.

*Rythinota*; Ferrer et al., 2016: 159; incorrect subsequent spelling for *Rhytinota* Eschscholtz, 1831.

*Rytinota*; Eschscholtz, 1831: 5, 7; incorrect original spelling, emended to *Rhytinota* Eschscholtz, 1831 under prevailing usage.

*Saragdonius*; Bertkau, 1876: 78; incorrect subsequent spelling for *Saragodinus* Bates, 1872.

*Sarathropus*; Heyden, 1895: 111; incorrect subsequent spelling for *Sarothropus* Kraatz, 1865.

*Saurothropus*; Dallas, 1866: 471; incorrect subsequent spelling for *Sarothropus* Kraatz, 1865.

*Scelidopsecta*; Kulzer, 1954b: 204; incorrect original spelling for *Scelidospecta* Kulzer, 1954.

*Sceloblaps*; Kolbe, 1928: 203; published before 1931 without a description, a definition or an indication.

*Schizomena*; Sharp, 1922: 142; incorrect subsequent spelling for *Schizomma* Gebien, 1921.

*Scytodonta*; Reitter, 1900c: 166; incorrect subsequent spelling for *Scythodonta* Reitter, 1894.

*Selenepistomus*; Mulsant and Rey, 1853b: 20; incorrect subsequent spelling for *Selenepistoma* Dejean, 1834.

*Selenoma*; Peña, 1966: 408; incorrect subsequent spelling for *Selenomma* Dejean, 1836.

*Sepidiostemis*; Reitter, 1914c: 382; incorrect subsequent spelling for *Sepidiostenus* Fairmaire, 1884.

*Sepidostenus*; Fairmaire, 1887a: 184; incorrect subsequent spelling for *Sepidiostenus* Fairmaire, 1884.

*Sericeus* Ragusa, 1898: 114; incorrect subsequent spelling for *Seriscius* Motschulsky, 1845.

*Solenopistoma*; Mulsant and Rey, 1854: 29; incorrect subsequent spelling for *Selenepistoma* Dejean, 1834.

*Solskia*; Semenov, 1891: 263; incorrect subsequent spelling for *Solskyia* Solsky, 1881.

*Solskya*; Semenov-Tjan-Shansky, 1908: 265; incorrect subsequent spelling for *Solskyia* Solsky, 1881.

*Somaticum*; Hope, 1841: 117; incorrect original spelling for *Somaticus* Hope, 1841.

*Spaerotidius*; Kaszab, 1941a: 39, 40; alternative original spelling of *Sphaerotidius* Kaszab, 1941, herein rejected.

*Sphaenariopsis*; Blair, 1935a: 103; incorrect original spelling for *Sphenariopsis* Kraatz, 1865.

*Sphingidophorus*; Pic, 1917: 1; incorrect original spelling for *Sphragidophorus* Champion, 1889.

*Sphoerotus*; Brême, 1842a: 106; incorrect original spelling for *Sphaerotus* W. Kirby, 1819.

*Spinanaedus*; Gebien, 1941: 826; incorrect original spelling for *Spinadaenus* Pic, 1921.

*Spinolystronychus*; Pic, 1944: 5; published after 1930 without a type species designation (see Bousquet et al. 2015: 139).

*Statyra*; Lacordaire, 1830b: 154; incorrect original spelling for *Statira* Lepeletier & Audinet-Serville, 1828.

*Stegatopsis*; Allard, 1884: 23; incorrect subsequent spelling for *Stegastopsis* Kraatz, 1865.

*Stenochara*; Hope, 1841: 70; incorrect subsequent spelling for *Stenocara* Solier, 1835.

*Stenogenius*; Solier, 1834: 520; first published as a synonym and not treated before 1961 as an available name and adopted as the name of a taxon or treated as a senior homonym.

*Sternodus*; Agassiz, 1846b: 353; incorrect subsequent spelling for *Sternodes* Fischer von Waldheim, 1837.

*Subtentyrina*; Koch, 1939: 260; published after 1930 without a type species designation.

*Subtentyrina*; Löbl and Merkl, 2003: 251; published after 1999 and not explicitly indicated as intentionally new.

*Sulpiosoma*; Ferrer, 2006c: 79; alternative original spelling of *Sulpiusoma* Ferrer, 2006, herein rejected.

*Symnetasida*; Ragusa, 1921: 100; incorrect subsequent spelling for *Gymnetasida* Reitter, 1917

*Syntractus*; Borchmann, 1909b: 2; incorrect subsequent spelling for *Synatractus* W.J. Macleay, 1887.

*Taeniobates*; Motschulsky, 1872: 32; alternative original spelling of *Taenobates* Motschulsky, 1872, herein rejected.

*Taganoides*; Kaszab, 1965: 109; incorrect subsequent spelling for *Tagonoides* Fairmaire, 1886.

*Tagaulus*; Hayashi, 1980: 19; incorrect subsequent spelling for *Tagalus* Gebien, 1914.

*Talpophila*; Macquart, 1850: 174; incorrect subsequent spelling for *Thalpophila* Solier, 1835.

*Tanchyrus*; Kaszab, 1941a: 2, 42; incorrect subsequent spelling for *Tanchirus* Fairmaire, 1897.

*Tanuria*; Lacordaire, 1859: 454; first published as a synonym and not treated before 1961 as an available name and adopted as the name of a taxon or treated as a senior homonym.

*Targesius*; Gistel, 1848: xi; proposed as a replacement name for the unavailable name *Melancrus* Dejean, 1834; published before 1931 without a description, a definition, or an indication.

*Tarsoconodes*; Sharp, 1922: 142; incorrect subsequent spelling for *Tarsocnodes* Gebien, 1920.

*Taurocerus*; Agassiz, 1846b: 362; incorrect subsequent spelling for *Tauroceras* Hope, 1841.

*Taxonema*; Bertkau, 1876: 78; incorrect subsequent spelling for *Toxocnema* Fåhraeus, 1870.

*Telleas*; Gebien, 1911a: 447; incorrect subsequent spelling for *Telleus* Fairmaire, 1904.

*Teneobrionites*; Bousquet et al. 2018: 381; incorrect subsequent spelling for *Tenebrionites*Cockerell 1920.

*Tentiria*; Dahl, 1823: 41; incorrect subsequent spelling for *Tentyria* Latreille, 1802; published in a work suppressed for the purposes of zoological nomenclature (ICZN 1964, Opinion 710).

*Tentyrionota*; Gridelli, 1937: 38; incorrect subsequent spelling for *Tentyronota* Reitter, 1900.

*Thecocerus*; Agassiz, 1846b: 368; proposed as an unjustified emendation for the unavailable name *Thecacerus* Dejean, 1834; published before 1931 without a description, a definition, or an indication.

*Thoracophora*; Imhoff, 1856: 242; incorrect subsequent spelling for *Thoracophorus* Hope, 1841.

*Thraucostolus*; Gebien, 1910a: 74; incorrect subsequent spelling for *Thraustocolus* Kraatz, 1866.

*Thryptera*; Gaubil, 1849: 213; incorrect subsequent spelling for *Thriptera* Solier, 1836.

*Thylacoderus*; Burmeister, 1875: 489; incorrect subsequent spelling for *Thylacoderes* Solier, 1843.

*Trachelaeum*; Agassiz, 1846b: 373; incorrect subsequent spelling for *Tracheloeum* Hope, 1841.

*Tracheloblaps*; Kolbe, 1928: 203; published before 1931 without a description, a definition or an indication.

*Trachinotus*; Gistel, 1848a: xi; incorrect subsequent spelling for *Trachynotus* Latreille, 1828.

*Treintoma*; Wolcott, 1950: 326; incorrect subsequent spelling for *Trientoma* Solier, 1835.

*Trelolosodis*; Cazurro Ruiz, 1897b: 427; incorrect subsequent spelling for *Scelosodis* Solier, 1835.

*Tribolocara*; Lacordaire, 1859a: 69, 72; incorrect subsequent spelling for *Trilobocara* Solier, 1851.

*Trichochianalis*; Kaszab, 1940a: 201; alternative original spelling of *Trichochianalus*, rejected by Ksazab (1940b: 989).

*Tricholeipoleura*; Kaszab, 1940a: 152; alternative original spelling of *Tricholeipopleura*, rejected by Kaszab (1940b: 989).

*Trichopodum*; Gebien, 1905: 253; incorrect subsequent spelling for *Trichopodus* Mulsant & Rey, 1859.

*Trichosphena*; Gebien, 1937a: 577; incorrect subsequent spelling for *Trichosphaena* Reitter, 1916.

*Trigonoides*; Fairmaire, 1901c: 267; incorrect subsequent spelling for *Tagonoides* Fairmaire, 1886.

*Trinobatis*; Laporte, 1840: 195; incorrect subsequent spelling for *Thinobatis* Eschscholtz, 1831.

*Trisilius*; Bertkau, 1879: 505; incorrect subsequent spelling for *Trisilus* Haag-Rutenberg, 1878.

*Trogloderes*; Gebien, 1938a: 64; incorrect subsequent spelling for *Trogloderus* J.L. LeConte, 1879.

*Ulomimimus*; Bates, 1873a: 201; incorrect original spelling, emended to *Ulomimus* Bates, 1873 under prevailing usage.

*Ulona*; Dahl, 1823: 43; incorrect subsequent spelling for *Uloma* Dejean, 1821; published in a work suppressed for the purposes of zoological nomenclature (ICZN 1964, Opinion 710).

*Umbraticus*; Voet, 1806: 81; published in a work that did not include consistent application of binominal nomenclature.

*Urosis*; Deyrolle, 1867: 81; alternative original spelling of *Anisosis* Deyrolle, 1867, rejected by Dallas (1868: 265–266).

*Usagaria*; Péringuey, 1892a: 56; incorrect subsequent spelling for *Uzagaria* Ancey, 1881.

*Usoma*; Berthold, 1827: 369; incorrect subsequent spelling for *Uloma* Dejean, 1821.

*Valeron*; Cazurro Ruiz, 1897b: 97; incorrect subsequent spelling for *Scleron* Hope, 1841.

*Verticiphloeus*; Bremer and Lillig, 2017b: 201; published after 1930 without a description, a definition, or a bibliographic reference to such a published statement.

*Victa*; Allard, 1870: 50; incorrect subsequent spelling for *Vieta* Laporte, 1840.

*Xanthomerus*; Escalera, 1914: 353; incorrect subsequent spelling for *Xanthomus* Mulsant, 1854.

*Xanthotopeia*; Fairmaire, 1894g: 671, 672; incorrect subsequent spelling for *Xanthothopeia* Mäklin, 1867.

*Xanthotopia*; Kolbe, 1897b: 619; incorrect subsequent spelling for *Xanthothopeia* Mäklin, 1867.

*Zaleucos*; Gebien, 1937a: 715; incorrect subsequent spelling for *Zaleucus* Champion, 1892.

*Zaphobas*; Papp, 1961: 128; incorrect subsequent spelling for *Zophobas* Dejean, 1834.

*Zarudnyonymus*; Kaszab 1981b: 367; incorrect subsequent spelling for *Zarudnionymus* Semenov-Tjan-Shansky & Bogatchev, 1947.

*Zioelas*; Gebien, 1943: 914; incorrect subsequent spelling for *Ziaelas* Fairmaire, 1892.

*Zophabas*; Motschulsky, 1872: 35; incorrect subsequent spelling for *Zophobas* Dejean, 1834.

*Zophelops*; Gebien, 1911a: 534; incorrect subsequent spelling for *Zophohelops* Reitter, 1902.

*Zophobius*; Dejean, 1834: 180; published before 1931 without a description, a definition, or an indication.

*Zypoetus*; Kaszab, 1977: 303; incorrect subsequent spelling for *Zypoetes* Champion, 1893.

## Appendix 2

Supporting references for conservation of *Cyphaleus* Westwood, 1841 over *Chrysobalus* Boisduval, 1835 through reversal of precedence (ICZN 1999, Article 23.9.2). To our knowledge *Chrysobalus* Boisduval, 1835 has not been used as a valid name after 1899.

1. Bouchard P, Bousquet Y, Davies AE, Alonso-Zarazaga MA, Lawrence JF, Lyal CHC, Newton AF, Reid CAM, Schmitt M, Ślipiński SA, Smith ABT (2011) Family-group names in Coleoptera (Insecta). ZooKeys 88: 1–972.
2. Bouchard P, Lawrence JF, Davies AE, Newton AF (2005) Synoptic classification of the world Tenebrionidae (Insecta: Coleoptera) with a review of family-group names. Annales zoologici (Warszawa) 55: 499–530.
3. Doyen JT, Matthews EG, Lawrence JF (1990) Classification and annotated checklist of the Australian genera of Tenebrionidae (Coleoptera). Invertebrate Taxonomy 3 [1989] (3): 229–260.
4. Hangay G, Zborowski P (2010) A guide to the beetles of Australia. CSIRO Publishing, Collingwood. x + 238 pp.
5. Kukalová-Peck J, Lawrence JF (1993) Evolution of the hind wing in Coleoptera. The Canadian Entomologist 125: 181–258.
6. Kukalová-Peck J, Lawrence JF (2004) Relationships among coleopteran suborders and major endoneopteran lineages: evidence from hind wing characters. European Journal of Entomology 101: 95–144.
7. Lawrence JF, Britton EB (1994) Australian beetles. Melbourne University Press, 192 pp.
8. Lawrence JF, Leschen RAB (2010) 11.12. Chalcodryidae Watt, 1974. Pages 567–571. In: Leschen, RAB, Beutel, RG, Lawrence, JF (Eds). Handbook of Zoology. A Natural History of the Phyla of the Animal Kingdom. Volume IV - Arthropoda: Insecta. Part 38. Coleoptera, Beetles. Volume 2: Systematics (Part 2). Walter de Gruyter, Berlin.
9. Lawrence JF, Newton Jr AF (1995) Families and subfamilies of Coleoptera (with selected genera, notes, references and data on family-group names). In: Pakaluk J, Slipinski SA (Eds) Biology, phylogeny, and classification of Coleoptera. Papers celebrating the 80 th birthday of Roy A. Crowson. Muzeum I Instytut Zoologii PAN, Warszawa, pp. 779–1006.
10. Lawrence JF, Slipinski A (2013) Australian beetles. Volume 1: morphology, classification and keys. CSIRO Publishing, Collingwood.
11. Leschen RAB, Escalona HE, Elgueta M (2016) Phylogeny of the Gondwanan beetle family Ulodidae (Tenebrionoidea). Zootaxa 4138: 441–473.
12. Matthews EG (1980) A guide to the genera of beetles of South Australia. Part 5. Polyphaga: Tenebrionoidea. South Australian Museum. vi + 67 pp.
13. Matthews EG (1992) Classification, relationships and distribution of the genera of Cyphaleini (Coleoptera: Tenebrionidae). Invertebrate Taxonomy 6: 437–522.
14. Matthews EG (2004) New synonymy and new names in Australian Tenebrionidae (Coleoptera). Transactions of the Royal Society of South Australia 128: 261.
15. Matthews EG, Bouchard P (2008) Tenebrionid Beetles of Australia: Description of tribes, keys to genera, catalogue of species. Commonwealth of Australia, Canberra.
16. Matthews EG, Lawrence JF (2019) In: Slipinski A, Lawrence JF (Eds) Australian beetles. Volume 2: Archostemata, Myxophaga, Adephaga, Polyphaga (part). CSIRO Publishing, Clayton South, pp. 582–661
17. Matthews EG, Lawrence JF, Bouchard P, Steiner Jr WE, Ślipiński SA (2010) 11.14. Tenebrionidae Latreille, 1802. Pages 574–659. In: Leschen, RAB, Beutel, RG, Lawrence, JF (Eds). Handbook of Zoology. A Natural History of the Phyla of the Animal Kingdom. Volume IV - Arthropoda: Insecta. Part 38. Coleoptera, Beetles. Volume 2: Systematics (Part 2). Walter de Gruyter, Berlin.
18. Matthews EG, Scupola A (2003) Entomological investigations in Australia by the Natural History Museum of Turin: Coleoptera, Tenebrionidae. Museo Regionale di Scienze Naturali, Monografie 35: 281–302.
19. Moore BP (1980) A guide to the beetles of southeastern Australia. Australian Entomological Press, Greenwich, Australia, 226 pp.
20. Naumann I (1993) CSIRO handbook of Australian insect names: common and scientific names for insects and allied organisms of economic and environmental importance. Sixth Edition. CSIRO Publishing, East Melbourne, Australia, 200 pp.
21. Steiner Jr WE (2009) Tenebrionidae of Australia. Descriptions of tribes. Keys to genera. Catalogue of species by E.G. Matthews and P. Bouchard. Australian Biological Resources Study, GPO Box 787, Canberra ACT 2601, Australia, 2008, pp. 398. Systematic Entomology 34: 198.
22. Stone c, Goodyer G, Sims K, Penman T, Carnegie A (2010) Beetle assemblages captured using static panel traps within New South Wales pine plantations. Australian Journal of Entomology 49: 304–316.
23. Williams G (2002) A taxonomic and biogeographic review of the invertebrates of the Central Eastern Rainforest Reserves of Australia (CERRA) World Heritage Area, and adjacent regions. Technical Reports of the Australian Museum 16: 1–208.
24. Williams G (2020) The invertebrate world of Australia’s subtropical rainforests. CSIRO Publishing, Clayton South, 385 pp.
25. Williams G, Evans T (1993) Hidden rainforests: subtropical rainforests and their invertebrate biodiversity. New South Wales University Press, 188 pp.
